# Research on the dynamic spillover of stock markets under COVID-19—Taking the stock markets of China, Japan, and South Korea as an example

**DOI:** 10.3389/fpubh.2022.1008348

**Published:** 2022-11-11

**Authors:** Baicheng Zhou, Qingshu Yin, Shu Wang, Tianye Li

**Affiliations:** ^1^School of Economics, Jilin University, Changchun, China; ^2^School of Business, Changchun Guanghua University, Changchun, China

**Keywords:** spillover effects, stock market, financial integration, SV-TVP-FAVAR, COVID-19

## Abstract

Examining stock market interactions between China (mainland China and Hong Kong), Japan, and South Korea, this study employs a framework that includes 239 economic variables to identify the spillover effects among these three countries, and empirically simulates the dynamic time-varying non-linear relationship between the stock markets of different countries. The findings are that in recent decades, China's stock market relied on Hong Kong's as a window to the exchange of price information with Japan and South Korea. More recently, the China stock market's spillover effect on East Asia has expanded. The spread of the crisis has strengthened co-movement between the stock markets of China, Japan, and South Korea.

## Introduction

**Figure 1:**
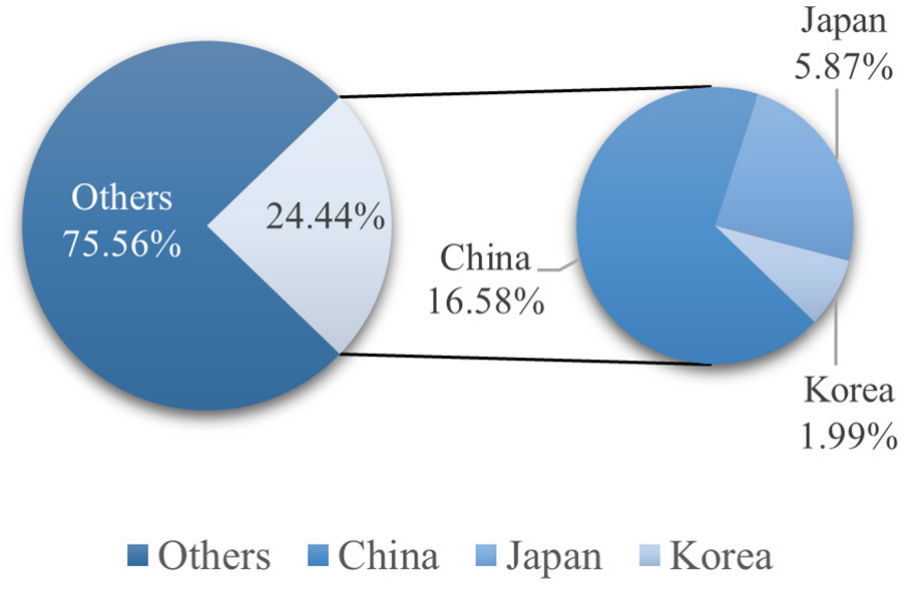
The relationship between the GDPs of China, Japan and South Korea, and the relationship between the total GDP of the three countries and total world GDP. The unit in the figure is 100 million U.S. dollars.

In recent years, the economic and social integration of East Asia has unprecedentedly deepened. Looking back on history, most of East Asia's economic integration has stemmed from crises (1–3). At the beginning of 2020, COVID-19 swept around the world. As the epidemic spreads across the earth, the outbreak of a major infectious disease has seriously impacted the world economy through complicated transmission channels. COVID-19 is one of the biggest crises the world economy has suffered in recent years (4–6). The stock market is an important aspect of economic integration (7, 8). In this study, we focus on the changes in from the stock markets in China, Japan and South Korea under the influence of the new coronavirus epidemic. China, Japan, and South Korea are geographical neighbors as well as close economic and trading partners; the three countries are particularly important in East Asia both politically and economically. Figure 1 shows the relationship between the total GDPs of China, Japan and South Korea, which together account for nearly one-quarter of the global economy. As these three countries represent three major world economies, their economic situation is closely associated with world economic development. To date, China is the largest economy that trades with Japan and South Korea. Japan and South Korea are China's second and third largest trade partners, respectively. In the past two decades, these countries have continuously promoted mutual trust and deepened cooperation and joint development. In terms of economic and trade exchanges, the three countries are significant trading partners. China, Japan, and South Korea have successively opened their financial markets, and their relationships are deepening daily. China, Japan, and South Korea belong to a common Confucian cultural circle in East Asia and the trading times of the stock markets coincide; investors are more inclined to allocate their assets to states with a higher degree of cultural intimacy when facing trade and financial frictions (9). Therefore, it is assumed that the financial interactions among China, Japan, and South Korea will be further enhanced as they become more integrated in various aspects. The disruption caused by the COVID-19 pandemic in 2020 has led to panic, which is likely to alter the world economy. Thus, we deem that this pandemic will further give rise to the fragmentation of globalization, and the regional financial cooperation will likely surpass that of prior global economic partnerships. Therefore, there is a great need to explore financial integration in East Asia.

The existing literature investigating the financial integration of East Asia considers numerous aspects and reaches some meaningful conclusions. Nevertheless, there are still various gaps in the literature that need to be addressed. Our article contributes to the literature in the following ways. The previous literature researching financial markets' connections usually utilizes a conventional econometric model to identify the spillover effects of markets. However, this approach considers the linkage between markets from only a single perspective, actually concealing a complex transmission mechanism and tending to rely on unreliable proxies, so the results deviate considerably from the economic reality. To avoid the above-mentioned problems, we construct a unified framework including a large number of factors involved in the interactions that occur between financial markets and rely on a more empirical method. Second, the prior literature using econometrics to empirically investigate inter-market interactions has somewhat identified the spillover effects between markets. Most of these studies indicate that the linkage between markets does not change over the long term or relies on specific time periods or zoning to identify the interactions. In reality, financial market integration is continuous and time-varying (10). The existing literature does not explore when and under what conditions spillovers occur between financial markets or the characteristics of these spillovers; furthermore, the literature has not comprehensively studied the dynamic evolution of the interactions between the financial markets of China, Japan, and South Korea to promote their financial linkage and cooperation among the three economies. In addition, the frequency of the release of macro data is completely different from that of financial market data. If the same frequency of data release were used to maintain consistency in the data, important high-frequency data would be lost. To address this gap, this paper constructs an econometric vector autoregression (VAR) model to identify the interactions among the stock markets during specific time periods, effectively capturing the financial market linkage among the three nations, which have diverse economic backgrounds. In addition, this study empirically identifies and untangles the evolution of these interactions. Finally, it is important to investigate whether heterogeneity exists in spillovers between financial markets that occur during unexpected external shocks. There has never been a large-scale infectious disease outbreak affecting China, Japan and South Korea on a large scale since the formation of a relatively mature stock market [neither the Severe Acute Respiratory Syndrome (SARS) virus in China in 2003 nor the Middle East Respiratory Syndrome (MERS)-related coronavirus in South Korea in 2015 led to such a pandemic]. Therefore, the impact of COVID-19 is unprecedented. Therefore, this article uses the time points of the Asian financial crisis in 1997 and the global economic crisis in 2008 to investigate changes in the interactions among multiple financial markets when the economy has experienced severe external shocks. Learning from this relevant experience can be helpful for understanding changes in the interactions among the stock markets under the massive impact of COVID-19 and provide an empirical foundation for China to deal with the financial integration of China, Japan, and South Korea in the post-epidemic era.

Our significant findings are as follows: First, our econometric models reveal that the influence of China's stock market on the surrounding markets is increasing; at present, China's revenue spillover effect on the Japanese and Korean stock markets is the same as that of Hong Kong stock market, but it is not greater than the spillover effect between the Japanese and South Korean stock markets. Second, when the economy encounters external shocks, the revenue spillover effect will deepen with the crisis but can vary. We find that severe shocks during a global economic crisis cut off the normal transmission channels that exist between financial markets, suggesting that we should focus on East Asia's regional economic integration from a dynamic and global perspective due to the unprecedented COVID-19 pandemic. In addition to learning from the experience but not copying it completely, financial integration in East Asia and China's positioning requires that attention be paid to the varying degree to which China's stock markets affect the stock markets of Japan and South Korea. Our research conclusions illustrate that China's stock market needs to be continuously developed, starting with higher-level systems and rules.

The rest of this paper is arranged in the following order: the second part reviews the related literature. The third part establishes a model that considers various factors and can capture changes that occur in the complex economic system and highlights the relevant assumptions. The fourth part analyses the relevant empirical results. The fifth part tests parameter stability. The sixth part summarizes the entire paper.

## Literature review

Fama (11) proposed the efficient market hypothesis (EMH) in 1970, arguing that a marketplace conforms to conditions such that the price of securities varies freely according to changes in information, fully disclosed relevant information and evenly distributed information. The market will no longer have external efficiency. In other words, a market that does not meet the efficiency conditions will exchange information and funds through different markets and a spillover effect will arise between financial markets (12–17). This investigation is chiefly carried out by considering two types of spillovers: return spillovers (18, 19) and volatility spillovers (20, 21). Research tends to show that stock market integration exists and is strengthened in peculiar periods (such as during the global economic crisis) (22–25). Due to accelerated financial integration, substantial investors no longer confine their asset portfolio to their domestic market. The frequent occurrence of cross-market investment has exposed the domestic financial market to a global perspective, which means that the domestic market can broaden its investment channel, lower market entry barriers, and enhance the stability of the domestic stock market (2, 26, 27) by introducing more diversified financing channels and investment sources. However, it is possible to augment the venture exposure of the domestic market (28, 29); thus, given the complex relationships that exist between financial systems, it is necessary for us to comprehensively consider the “dividends” and “challenges” of financial integration (30). Uddin et al. (31) evaluate the dependence dynamics among the affected countries and the global financial index using time-varying DCC-Student-t copula method, the conclusion can be used for reference to the mutual spillover between stock markets.

Few prior studies have focused on the financial integration of East Asia. The limited literature mostly deemed the degree of financial integration in East Asia to be far less than that seen globally (32–34). Both political conflicts and currency selection have hindered regional economic integration (35). Because of the significance and complexity of the stock market, we focus on the integration of stock markets in East Asia and specifically on the spillover effects among the stock markets of China, Japan, and South Korea. Wang and Li (8) verified the long-term and short-term cointegrated relationships among the stock markets of the three countries, finding that although the correlation between the stock markets of Japan and South Korea is higher than that between China and the other two countries, there is neither a short-term nor long-term cointegrated relationship between the stock markets of Japan and South Korea.

Moreover, the influence of China's stock market on East Asia has increased considerably. An (36) established a gravity model to verify that the degree of financial integration in East Asia is increasing annually, indicating that reducing East Asian nations' dependence on economies outside the domain is conducive to capital risk-sharing countries in East Asia. Burdekin and Siklos (37) and Huyghebaert and Wang (38) indicated that global factors could have a notable impact on Asian stock markets. Wu (39) further found that if international factors were excluded from the East Asian stock market interaction's influencing factors, the spillover effect between markets would be sharply decreased, which means that if we purely focus on the mutual spillovers among the stock markets, the contribution of the overall macro factors to the inter-market interactions will be ignored, which will lead to biased conclusions. Unfortunately, there is still no literature on the integration of complicated economic factors in the financial system into a unified time-varying framework to fit the spillover effect between the stock markets of China, Japan, and South Korea. This empirical research aims to fill this gap and provide policy recommendations for institutional financial and policy cooperation among East Asian economies.

## Methodology

A general linear VAR model can be written as follows (40):


(1) $$\begin{array}{l} Y_{t}=\Gamma \left(\text{L}\right)Y_{t-1}\text{+}\varepsilon_{t} \end{array}$$

where Γ(*L*) is a matrix of lagged polynomials, and ε_*t*_ ~ *N*(0, Ω_*t*_) is the disturbance in the model. This VAR model is not affected by endogenous relationships among variables and can identify the dynamic relationships that occur between variables in a multivariate time series; structural decomposition is carried out. However, the traditional linear model has several disadvantages. First, the conventional model is affected by a “curse of dimension” when dealing with high-dimensional variables. However, the interactions among the stock markets involve various influencing factors of the economic systems. Consequently, if the contribution of macro-and microelements to the spillover effects is not considered, the empirical results may not be accurate. Second, in the classical model, the variables must have linear relationships. The spillover effect between stock markets studied in this paper, which is more intricate and multivariate, obviously does not conform to this assumption. The linear model apparently cannot capture this subtle dynamic and time-varying aspect of the economic system.

To compensate for the shortcomings of the classical model, for problem one, this paper considers the economic factors that may be involved in the spillover effect of global stock markets in a unified analysis framework. At the same time, to avoid redundant influencing factors causing the econometric model to appear in the “curse of dimension,” referring to the practice of Bernanke et al. (41), many unobservable influencing factors are reflected in *U*_*t*_, so the classical model is extended to:


(2) $$\begin{array}{l} \left[\begin{array}{c} U_{t}\\ O_{t} \end{array}\right]=\Pi \left(\text{L}\right)\left[\begin{array}{c} U_{t-1}\\ O_{t-1} \end{array}\right]\text{+}\varepsilon_{t} \end{array}$$

where *O*_*t*_ is an m × 1 vector of observed variables. Estimating Equation (2) is difficult because *U*_*t*_ is too high and unobservable. Due to these unobservable variables, the standard method cannot be used to estimate the above formula. To solve this problem, the vector *X*_*t*_ with dimensions n × 1 is introduced as an information set to extract common latent factors, and the relationships between the information set and the observable and unobservable variables is as follows:


(3) $$\begin{array}{l} X_{t}=\Lambda^{O}O_{t}+\Lambda^{U}U_{t}+v_{t} \end{array}$$

Equation (3) is called the factor extraction equation. Where Λ^*O*^ and Λ^*U*^ are matrices of factor loadings and *v*_*t*_ is a vector of normally distributed random shocks. Thus, the impulse response functions (IRFs) can be written as follows:


(4) $$\begin{array}{l} {\hat{X}}_{t}=\left[\begin{array}{cc} {\hat{\Lambda }}^{U} & {\hat{\Lambda }}^{O} \end{array}\right]\left[\begin{array}{c} {Û}_{t}\\ O_{t} \end{array}\right]=\left[\begin{array}{cc} {\hat{\Lambda }}^{U} & {\hat{\Lambda }}^{O} \end{array}\right]\Psi \left(L\right)u_{t} \end{array}$$

where ${Û}_{t}={\hat{X}}_{t}-\beta_{O}O_{t}$,Ψ(*L*) = Π(*L*)^−1^.

For the second problem of the classical model, the original model parameters must be extended, and the invariant coefficient is rewritten as time varying:


(5) $$\begin{array}{l} \{\begin{array}{l} x_{it}={\tilde{\lambda }}_{i}^{O}O_{t}+{\tilde{\lambda }}_{i}^{U}U_{t}+\tau_{it}\\ \tau_{it}=b_{it}\tau_{t-1}+...+b_{it}\tau_{t-p}+\xi_{it} \end{array} \end{array}$$

${\tilde{\lambda }}_{i}^{O}$, ${\tilde{\lambda }}_{i}^{U}$ are the matrices of the factor loadings, respectively. *t* = 1, ..., *T*,τ_*t*_ ~ *N* (0, Ω_*t*_), *i* = 1, ..., *p*, ξ_*it*_ ~ *N* [0, exp (*h*_*it*_)]. The perturbation term in Equation (5) is set to conform to the random-walk form. Thus, the original formula is arranged as follows:


(6) $$\begin{array}{l} x_{t}=\lambda^{O}O_{t}+\lambda^{U}U_{t}+\Upsilon \left(L\right)x_{t}+\varepsilon_{t} \end{array}$$

where $\Upsilon \left(L\right)=diag\left(\rho_{11}L+...+\rho_{1w}L^{w},...,\rho_{n1}L+...+\rho_{nw}L^{w}\right)$, ε_*t*_ ~ *N* (0, *H*_*t*_), *H* = *diag* (exp (*h*_1*t*_), ..., exp (*h*_*nt*_)), $\left[\begin{array}{cc} \lambda^{O} & \lambda^{U} \end{array}\right]=\left(I_{n}-\Gamma \left(L\right)\right)\left[\begin{array}{cc} {\tilde{\lambda }}^{O} & {\tilde{\lambda }}^{U} \end{array}\right]$, and the residual term ε is in line with the innovative random walk $h_{it}=h_{it-1}+\eta_{t}^{h}$. The extended econometric model can comprehensively consider the economic variables involved in stock market interaction and capture the economic system's dynamic changes to better fit the stock markets.

As this paper measured the spillover effect between stock markets, the daily macro indicators are also added, so a model needs to be established to convert the data into a relationship compatible with the quarterly indicators. Shang et al. (42) and Shang et al. (43) provide a state space model for mixing SV-TVP-VAR, where $J_{t}^{\left(d\right)}$ is a potential daily indicator, $J_{t}^{\left(q\right)}$ is the quarterly economic variable, and $J_{t}^{\left(q\right)}=\mu \left(L\right)J_{t}^{\left(d\right)}$. Among these variables, L is a lagging operator, and is a high-level polynomial. The conversion relationship between quarterly indicators and monthly indicators is the same. The mixing SV-TVP-VAR model can be expressed in the following state space form:


(7) $$\begin{array}{l} \left(\begin{array}{c} x_{t,N}^{\left(q\right)}\\ O_{t,R}^{\left(q\right)}\\ J_{t,S}^{\left(d\right)} \end{array}\right)=\left(\begin{array}{ccc} {\tilde{\lambda }}_{N,SP}^{J} & {\tilde{\lambda }}_{N,R}^{O} & {\tilde{\lambda }}_{N,i}^{U}\\ 0_{R,SP} & I_{R,R} & 0_{R,i}\\ \prod_{S,SP} & 0_{S,R} & 0_{S,i} \end{array}\right)\left(\begin{array}{c} J_{t,S}^{\left(q\right)}\\ O_{t,R}^{\left(q\right)}\\ U_{t}^{\left(q\right)} \end{array}\right)+\left(\begin{array}{c} \mu_{x,t}^{\left(m\right)}\\ 0\\ 0 \end{array}\right) \end{array}$$

(8) $$\begin{array}{l} \left(\begin{array}{c} J_{t,S}^{\left(q\right)}\\ O_{t,R}^{\left(q\right)}\\ U_{t}^{\left(q\right)} \end{array}\right)=B_{t}^{\left(q\right)}\left(\begin{array}{c} J_{t-1,S}^{\left(q\right)}\\ O_{t-1,R}^{\left(q\right)}\\ U_{t-1}^{\left(q\right)} \end{array}\right)+\left(\begin{array}{c} \varepsilon_{j,t,S,SP}^{\left(q\right)}\\ \varepsilon_{O,t,R}^{\left(q\right)}\\ \varepsilon_{u,t}^{\left(q\right)} \end{array}\right) \end{array}$$

(9) $$\begin{array}{l} \left(\begin{array}{c} \mu_{t}^{\left(q\right)}\\ \varepsilon_{t}^{\left(q\right)} \end{array}\right)\sim WN\left[\begin{array}{cc} \left(\begin{array}{c} 0\\ 0 \end{array}\right) & \left(\begin{array}{cc} H_{t}^{\left(q\right)} & 0\\ 0 & \Omega_{t}^{\left(q\right)} \end{array}\right) \end{array}\right] \end{array}$$

Among them, N, R, and S represent the number of variables of economic variables, quarterly and daily macro indicators, respectively. SP represents the total number of potential daily indicators corresponding to all quarterly indicators, That is, $SP=\overset{S}{\underset{i=1}{\sum }}p_{i}$, *p*_*i*_ represents the number of potential daily indicators corresponding to the i-th quarter macro indicators. Π_*S,SP*_ represents the conversion relationship between quarterly metrics and potential daily metrics:


(10) $$\begin{array}{l} \Pi_{S,SP}=\left[\begin{array}{cccc} \Pi_{1,p1} & 0_{1,p2} & \cdots & 0_{1,ps}\\ 0_{1,p1} & \Pi_{1,p2} & \cdots & 0_{1,ps}\\ \vdots & \vdots & \ddots & \vdots \\ 0_{1,p1} & 0_{1,p2} & \cdots & \Pi_{1,\text{ps}} \end{array}\right] \end{array}$$

The conversion of quarterly indicators and monthly indicators is the same as above, and will not be repeated here.

In the parameter estimation part of the SV-TVP-VAR model, the two-step estimation method of Stock and Watson (44) is used to estimate the parameters of the unobservable part extracted from the background data set. The resulting vector is then used for Bayesian estimation along with other parameters to be estimated in the model. The relevant a priori parameters refer to the classic literature as follows: $\left[{\tilde{\lambda }}_{t}^{O},{\tilde{\lambda }}_{t}^{U}\right]\sim N\left(0_{1\times \left(k+i+1\right)},10I_{k+i+1}\right)$, Γ_*i*_(*L*) ~ *N* (0_1 × *q*_, 10*I*_q_), *h*_*i*0_ ~ *N* (0, 4), $\sigma_{h}^{-1}$ ~ *Gamma* (0.01, 0.01), where *i* = 1,…,*N*. *B*_0_ ~ *N (*$\hat{B},\hat{V}$*)*, *C*_0_ ~ *N* (0, 4*I*), logσ_0_ ~ *N* (0, 4*I*), $Q_{\sigma }^{-1}\sim \;W\left[0.005\times \left(\dim\left(B\right)+1\right)\times \hat{V},\left(\dim\left(B\right)+1\right)\right]$, $Q_{\sigma }^{-1}\sim \;W\left[0.005\times \left(\dim\left(C\right)+1\right)\times \hat{V},\left(\dim\left(C\right)+1\right)\right]$, $Q_{\sigma }^{-1}\sim \;W\left(0.0001\times \left(\dim\left(\sigma \right)+1\right)\times I,\left(\dim\left(\sigma \right)+1\right)\right)$. For B and C there are dim (*B*) = *m* × *m* × *p*, dim (*C*) = *m* (*m* – 1)/2. dim (σ)= *m*, for the lag term coefficient and the variable coefficient, we have ${\hat{V}}_{ij}=\frac{1}{c_{2}}$, ${\hat{V}}_{ij}=\frac{0.001s_{i}^{2}}{c^{2}s_{j}^{2}}$, *c* = 1, …, *p, p*$\left(J_{t}^{\theta }\;=\;1\right)$ = π_θ_ 1 − *p*$\left(J_{t}^{\theta }\;=\;0\right)$, π_θ_ ~ *Beta* (1,1), *E*(π_θ_) = 0.5, *std*(π_θ_)≅ 0.29, θ_*t*_ ∈ {*B*_*t*_, *C*_*t*_, log σ_*t*_}.

Objective differences exist between the stock markets of China, Japan, and South Korea. In the 1990s, Japan and South Korea began opening their capital markets. However, China's stock market is still in the opening process and has long relied on Hong Kong stock market as a medium for foreign exchanges. Although China's influence in East Asia has increased in recent years, its regional economic cooperation mainly focuses on trade, investment, and infrastructure. Does the spillover effect of China's stock market on the neighboring countries' stock markets exceed that between the stock markets of Japan and South Korea, which were more open before? Can China's capital opening process increase the influence of China's stock market on perimeter nations? To answer the above questions, the first set of hypotheses is put forward:

Hypothesis 1a: Due to the advancement of the opening process, the spillover effect of China's stock market on the Japanese and Korean stock markets is strengthened, although this effect is not as large as the spillover effect between the Japanese and Korean stock markets.

Hypothesis 1b: The opening of the financial market has significantly enhanced the influence of China's stock market; thus, China's stock market has a larger spillover effect on the stock markets of Japan and South Korea than the spillover effect between the stock markets of these two countries.

Hypothesis 1c: There is no obvious time-varying trend in the spillover effect of China's stock market on the other stock markets; specifically, the process of financial opening is not related to the spillover effect of China's stock market on different stock markets.

Although it is still debated whether a country's financial openness brings about a lower cost, multi-channel investment, and financing model or a more vulnerable financial environment exposed to the impact of global risks, the conclusion that external shocks will give rise to the aggravation of numerous studies has supported market spillovers. The outbreak and prevalence of COVID-19 in 2020 will have a large-scale external impact on the global capital market. Conflicts have accumulated in the world economic system in recent years, and the epidemic is undoubtedly a catalyst for anti-globalization. This paper applies the method of selecting particular time points to observe the spillover effects between the three stock markets during the Asian financial crisis and the global economic crisis to help us speculate on the possible impact patterns of COVID-19 on the stock market spillover effect. It is also worth noting that the epidemic's impact is not the traditional U.S. dollar liquidity crisis, which is different from the global economic crisis. The leverage of financial institutions broke down, resulting in a sharp decline in liquidity. The critical issue faced by the financial market in this epidemic is that they are incredibly pessimistic about economic prospects. Therefore, we cannot merely observe financial markets' experience, such as the 1997 and 2008 crises. It needs to be analyzed from a broader global economic perspective. According to the existing literature, investors prefer to withdraw their positions in multiple markets during the economic crisis, resulting in a “herding effect,” which intensifies the interaction between financial markets. However, this analysis is based on the premise that investors can freely enter and leave diverse financial markets. The stock markets between China, Japan, and South Korea do not meet this prerequisite. Therefore, it is difficult to accurately conclude whether the crisis shock will enhance the stock market spillover effect in China, Japan, and South Korea. According to the above contents, the second set of opposite hypotheses is proposed:

Hypothesis 2a: When the economy is affected by an external impact, the interactions among the stock markets of China, Japan, and South Korea will be enhanced.

Hypothesis 2b: When the economy suffers severe external shocks, the interactions among the stock markets of China, Japan, and South Korea will be weakened rather than strengthened.

In this paper, the above-mentioned hypotheses are successively verified by empirical testing. In addition, to offer policy suggestions for strengthening regional economic cooperation in East Asia in the post-epidemic era, we further explore the differences between the stock markets of China, Japan, and South Korea.

## Empirical analysis

### Data

**Figure 2:**
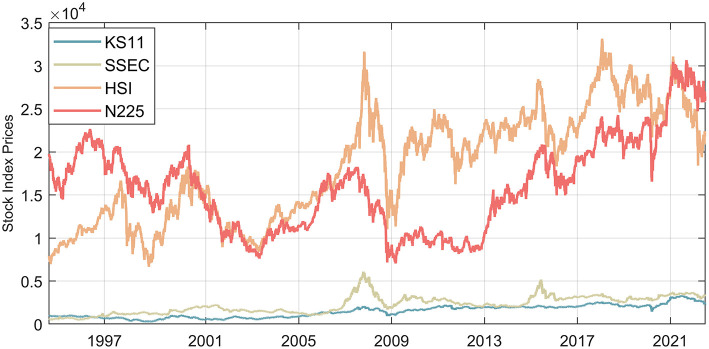
A chart of the historical trend of China's mainland stock market, China's Hong Kong stock market, Japan's stock market, and South Korea's stock market. Source: Author's own calculations. KS11 represents the Korea Composite Stock Price Index; SSEC represents the Shanghai Securities Composite Index; HSI represents the Hang Seng Index; N225 represents the Nikkei 225 Stock Index.

This study focuses on stock market linkages and what new role China should play in the financial integration of East Asia in the post-epidemic era, so we pay close attention to the exchange of price information between markets. This paper chooses a representative price series of different financial markets to test the cross-market return spillover and a composite index of the Shanghai stock exchange to represent the mainland stock market. Although China's stock market has been opening up, the mainland stock market is still relatively closed compared with more developed stock markets. Therefore, considering that Hong Kong is an important financial window for China, the Hong Kong stock market is also included in this paper's analysis through the Hang Seng Index. Moreover, this article chooses the Nikkei 225 index and the Korean composite index to represent the Japanese and Korean stock markets. In order to keep as much information from the high-frequency financial data as possible, all the stock market data in this paper use daily reports, as shown in Figure 2. The selected date range is from the first quarter of 1995 to the second quarter of 2022. All the return rates involved in this paper are logarithmic returns. All the data are from the wind database.

Considering that the stock markets of China, Japan and South Korea will not only affect each other, but also be affected by the return spillover of external financial markets, we further investigated. The commodity market, foreign exchange market, bond market and crude oil market are taken as the external financial markets under investigation. The statistical description of relevant data is shown in the Table 1. All the data are from the wind database.

**Table 1:** Statistical description of the main variables in the article.

**Variable**	**Mean**	**Std**.	**Median**	**Skew**.	**Kurt**.	**Min**.	**Max**.
SSEC	2,302.59	976.83	2,190.65	0.39	−0.17	5,367.82	546.62
SZI	7,740.47	4,185.29	8,280.61	0.20	−1.13	17,482.16	982.48
HIS	18,748.29	6,213.89	19,910.72	0.00	−1.21	31,255.88	7,806.48
N225	16,044.17	5,360.96	16,085.00	0.50	−0.35	29,001.71	7,924.67
KS11	1,516.66	712.77	1,638.50	0.18	−0.91	3,199.17	317.64
RJ/CRB	231.29	68.83	203.83	0.52	−0.67	425.29	124.86
USG10Y	3.69	1.62	3.57	0.20	−1.03	6.78	0.65
REER	110.59	9.54	111.88	−0.04	−0.71	133.03	90.76
CFD(OIL)	57.27	32.07	54.85	0.41	−0.97	122.79	11.56

Var., variable; Sam. Per., represents sample period; Max., maximum; Min., minimum; Std. Dev., standard deviation. SSEC represents the Shanghai Securities Composite Index; SZI represents the Shenzhen Component Index; KS11 represents the Korea Composite Stock Price Index; HSI represents the Hang Seng Index; N225 represents the Nikkei 225 Stock Index; RJ/CRB represents the RJ/CRB Commodity Price Index; USG10Y represents the U.S. 10-year Treasury yield; REER represents the Real effective exchange rate index of the US dollar; CFD(OIL) represents the Brent crude oil futures settlement price.

This paper analyses the interactions among the stock markets based on a broad perspective. It selects time series data constituted by 239 variables as the economic information set. Considering that many factors influence the economic system, we investigate the extraction mechanism, which makes it necessary to obtain considerable background data. The data with obvious seasonal factors are adjusted by the X-12 season. Some missing data are supplemented by the interpolation method. The non-stationary data tested by the augmented Dickey-Fuller (ADF) test are converted into static data by logarithmic transformation or the difference method due to space limitations, all data are not listed one by one but are retained for ready access, in the main text, we only list the ADF test results of the main financial market proxy variables and the results after stationary in Table 2. The first common factor F1 reflecting the macro-economic level is extracted from the industrial added value, GDP, and related macro-economic indexes reflecting the macro-economic fluctuation of China, Japan, and South Korea.

**Table 2:** ADF tests.

**Variable**	***T*-value**	***P*-value**	**Whether the data is a stationary series**
**The original data**			
SSEC	−0.131	0.602	0
SZI	−0.178	0.584	0
HIS	0.030	0.661	0
N225	0.521	0.827	0
KS11	0.898	0.900	0
RJ/CRB	0.234	0.735	0
USG10Y	−2.012	0.043	1
REER	0.466	0.813	0
CFD(OIL)	0.154	0.706	0
**Data after stationary processing**			
SSEC	−6.488	0.001	1
SZI	−6.510	0.001	1
HIS	−8.624	0.001	1
N225	−7.580	0.001	1
KS11	−6.901	0.001	1
RJ/CRB	−6.507	0.001	1
USG10Y	–		
REER	−9.277	0.001	1
CFD(OIL)	−7.697	0.001	1

1 represents the data as a stationary time series, and 0 represents the data with a unit root, which is an unstationary time series.

**Figure 3:**
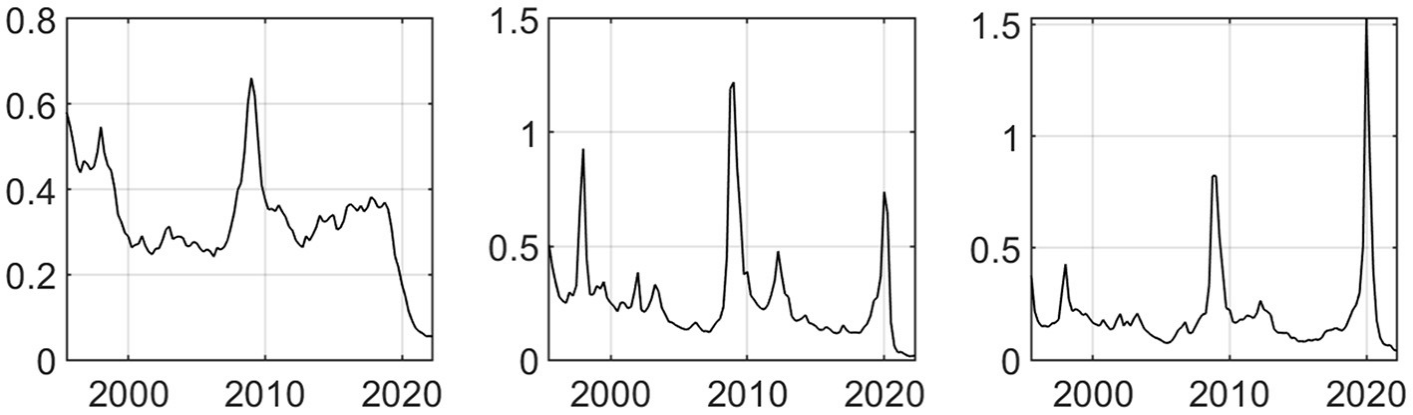
The desirable mean trend of the extracted non-observable common factors.

The data of the consumer price index, industrial producer price index, and purchasing power parity index of China, Japan, and South Korea are extracted as the second common factor F2 reflecting economic growth. The third common factor F3 is extracted from the data of money supply at all levels, interest rates of various maturities and exchange rates among countries. F1, F2, and F3 represent the trend of the extracted common factors, as shown in the Figure 3 below. It can be seen that the common factors have obvious responses during the global economic crisis and the impact of COVID-19 epidemic, and can contain most of the information of the 239 macroeconomic variables.

### Return spillovers among the stock markets of China, Japan, and South Korea

**Figure 4:**
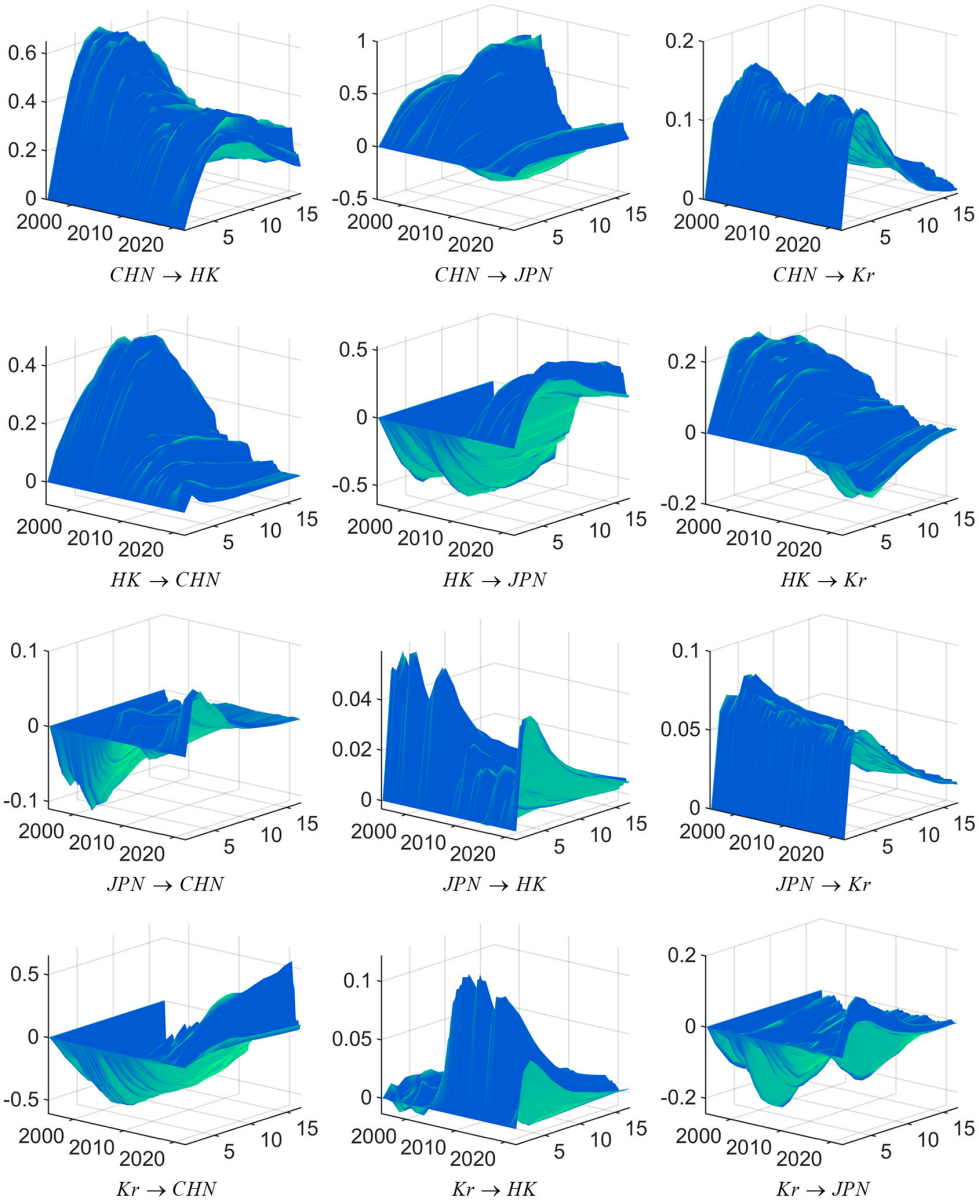
Three-dimensional impulse response graph of return spillovers between the stock markets of China, Japan, and South Korea. Source: Author's own calculations. CHN represents the China stock market, H.K. represents the Hong Kong stock market, JPN represents the Japan stock market, and Kr represents the South Korean stock market. Impulse response functions are to a 1 standard-deviation increase in the stock markets of China, Japan, or South Korea; each subfigure with the title of “*X* → *Y*” demonstrates the response of variable Y to a positive shock of variable X, wherein, X is an impulse variable, and Y is a response variable. One period in the figure denotes one season.

To verify hypotheses 1a, 1b, and 1c, a Markov chain Monte Carlo (MCMC) simulation based on the Bayesian framework is carried out. According to the model establishment and data selection analysis, it can be seen that $U_{t}={\left[F_{1},F_{2},F_{3}\right]}^{\prime }$, $J_{t}={\left[{\bar{Market\text{\_}China}}_{t},{\bar{Market\text{\_}HongKong}}_{t},{\bar{Market\text{\_}Japan}}_{t},{\bar{Market\text{\_}SouthKorea}}_{t}\right]}^{\prime }$ includes stock market data of China, Hong Kong, Japan and South Korea. After capturing the sophisticated and subtle time-varying effects in the economic system, we obtain the yield spillover's three-dimensional impulse response between China, Japan, and South Korea. The results are revealed in Figure 4 below.

We calculate the IRFs between the stock markets of China, Japan, and South Korea in a non-linear framework. These charts successively demonstrate the three-dimensional dynamic impulse response of other stock markets caused by the positive impact of one standard deviation of the China stock market's return rate, Hong Kong stock market, Japan stock market, and South Korean stock market. From the figures, we can gain the following comments. The first critical conclusion is that the Chinese mainland stock market has significantly increased the spillover effect on Japan's and Korea's stock markets. In contrast, China's Hong Kong stock market had a more substantial return spillover effect on the Japanese and Korean stock markets during the Asian financial crisis. In the fourth quarter of 2002, China began to implement the QFII system. The results of the three-dimensional impulse response diagram show that the spillover effect of China's stock market on Japan and South Korea has gradually increased since 2003. After the global economic crisis, China's stock market's spillover effect on other countries has further increased at an increasing rate. This process is synchronous with the gradual opening of China's stock market to the outside world.

The stock market of Japan and South Korea encountered an obvious shock and reached a short-term peak, according to the results for the second quarter in 2015. Generally, before China's stock market officially launched the QFII system in 2006, the Hong Kong stock market's influence on the Japanese and Korean stock markets was more significant than that of China's stock market. Nonetheless, the influence of China's mainland stock market strengthens rapidly as a result of the opening of the financial market; the spillover of the Chinese mainland stock market to the stock markets of Japan and Korea that occurred in recent years is similar to that of the Hong Kong stock market, and even has an increasing trend. Second, the extent of the response of China's stock market to a one-unit yield of the Japanese and Korean stock markets is gradually decreasing, which contrasts with the changing trend of the impact of China's stock market on Japan's and South Korea's stock markets; this result confirms that China's influence in the East Asian economic region is increasing annually. Third, an interesting phenomenon can be observed in the three-dimensional impulse response diagram of the return rate spillover effects among China, Japan, and South Korea. Due to the prevalence of the crisis, the interactions among the stock markets gradually increased. The normal transmission channel of price information between stock markets breaks down when a crisis occurs. It is difficult for investors to respond appropriately when a crisis occurs. Considering the reality of the stock markets in China, Japan, and South Korea, capital is not wholly free-flowing, especially when liquidity is sharply reduced and leverage density greatly decreases. Investors tend to withdraw their investment positions in different financial markets due to the time lag of the response, which leads to a more frequent exchange of price information in financial markets with the deepening of a crisis.

### The return spillovers among the stock markets of China, Japan, and South Korea at particular time points

**Figure 5:**
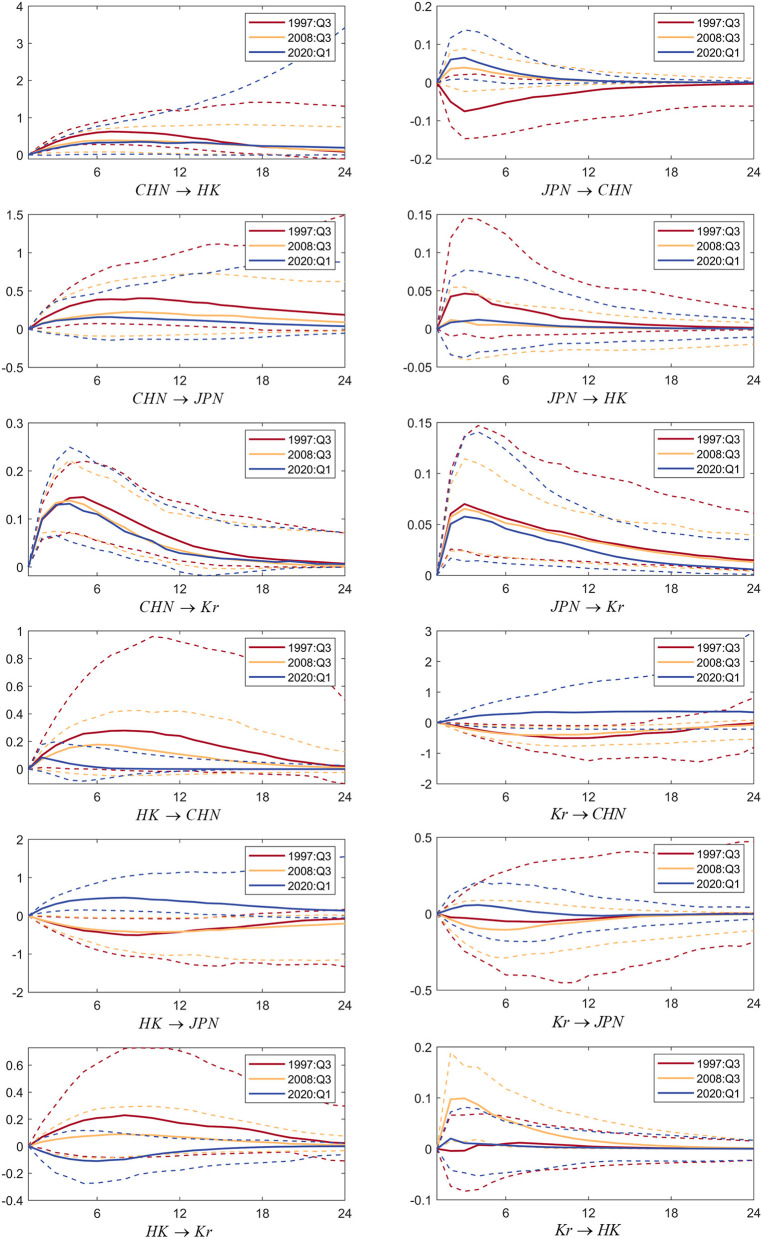
The return spillovers among the stock markets of China, Japan, and South Korea at particular time points. Sources: Author's own calculations. The upper and lower dotted lines represent the 10th and 90th of the distribution of the SV-TVP-FAVAR; The solid line represents the impulse response at different special periods.

The results shown in the graphs support Hypothesis 1a. The capital opening process coincides with an enhancement of the Chinese stock market's spillover effect on the Japanese and Korean stock markets. Despite the remarkable improvement in the opening degree and influence of China's stock market, its return spillover on the Japanese and Korean stock markets is still smaller than that between the Japanese and Korean stock markets. When the Asian financial crisis broke out, the opening degree of China's stock market was still small, and the linkage between China's stock market and Japan's and South Korea's stock markets was weak. China's influence on Japan's return and the South Korean stock market is completed through the Hong Kong stock market, which acts as a window. To further verify Hypothesis 2 regarding the change in the interaction between stock markets caused by acute external shocks, we choose typical time points from the third quarter of 1997, the third quarter of 2008 and the first quarter of 2020, corresponding to the Asian financial crisis, the outbreak of the global economic crisis, and the outbreak of COVID-19, respectively. This part still analyzes the mutual spillover between the stock markets of China, Japan and South Korea, so there are $J_{t}={\left[{\bar{Market\text{\_}China}}_{t},{\bar{Market\text{\_}HongKong}}_{t},{\bar{Market\text{\_}Japan}}_{t},{\bar{Market\text{\_}SouthKorea}}_{t}\right]}^{\prime }$. In this paper, we choose the best-lagged one-period model. The return spillovers among the stock markets of China, Japan, and South Korea at particular time points are shown in Figure 5.

The plots show that the mutual impact between the stock markets can be recovered within 2 years, and the measured value of the return spillover effect reaches the saturation state. Meanwhile, the following are observed. First, during the turbulent time, the Hong Kong stock market was impacted by the other stock markets, the magnitude of the impulse responses was similar, and the expected return spillover effect between the stock markets of Japan and South Korea showed the same characteristics, indicating that both the Hong Kong stock market of China and the stock markets of Japan and South Korea opened earlier; there was no marked difference in the degree of market openness during the crises. Notably, China's influence on East Asia's regional economy has strengthened, which has led to a decrease in the return spillover effect of the Japanese and Korean stock markets on China's stock market. Accordingly, the status of China's stock market has gradually changed from “passive receiver” to “active guide,” which is consistent with our conclusion that the linkage between China's stock market and Japan's and South Korea's stock markets in the period of global economic crisis was stronger than it was during the Asian financial crisis. During the COVID-19 outbreak, the effects of the shock in the stock markets of China, Japan and South Korea also showed different characteristics from past crises. Both the direction and magnitude of the response were different from previous crises, reminding us that the impact of the COVID-19 pandemic has brought more uncertainty to the economic system. The stock market linkage of China, Japan and South Korea also shows some new characteristics. Finally, the Chinese mainland stock market's return spillover effect on the Hong Kong stock market in the same period is larger than the return spillover of the Hong Kong stock market on the Chinese stock market. Similar phenomena also appear between the Chinese stock market and the Japanese and Korean stock markets, which can be explained from the same perspective.

### Interactions between the stock markets of China, Japan, and South Korea, and other financial markets

The last part of this paper provides a comprehensive evaluation of datasets containing abundant relevant economic variables and the return spillovers among the stock markets of China, Japan, and South Korea at particular time points, and some interesting conclusions are reached. However, considering that the stock market is one of the essential aspects of financial openness, its complexity and non-linearity are key challenges for this study. Therefore, it is necessary to further study the similarities and differences among the stock markets of China, Japan, and South Korea from the perspective of internal as well as external markets to provide the basis for restructuring the financial system in the East Asian economic region after the epidemic (45, 46). This section assesses the linkages that exist between the commodity markets and foreign exchange markets in China, Japan, and South Korea. The commodity price index generated by the Commodity Research Bureau (CRB) and the actual effective weighted exchange rate of the US dollar to major currencies are chosen as indicators. After adding external financial markets, the markets introduced in the model are $J_{t}={\left[{\bar{Market\text{\_}China}}_{t},{\bar{Market\text{\_}HongKong}}_{t},{\bar{Market\text{\_}Japan}}_{t},{\bar{Market\text{\_}SouthKorea}}_{t},{\bar{Market\text{\_}C}}_{t},{\bar{Market\text{\_}EF}}_{t},{\bar{Market\text{\_}B}}_{t},{\bar{Market\text{\_}CO}}_{t}\right]}^{\prime }$. The data were tested for stationarity, and some missing data were supplemented by interpolation. All data were obtained from the Wind database.

**Figure 6:**
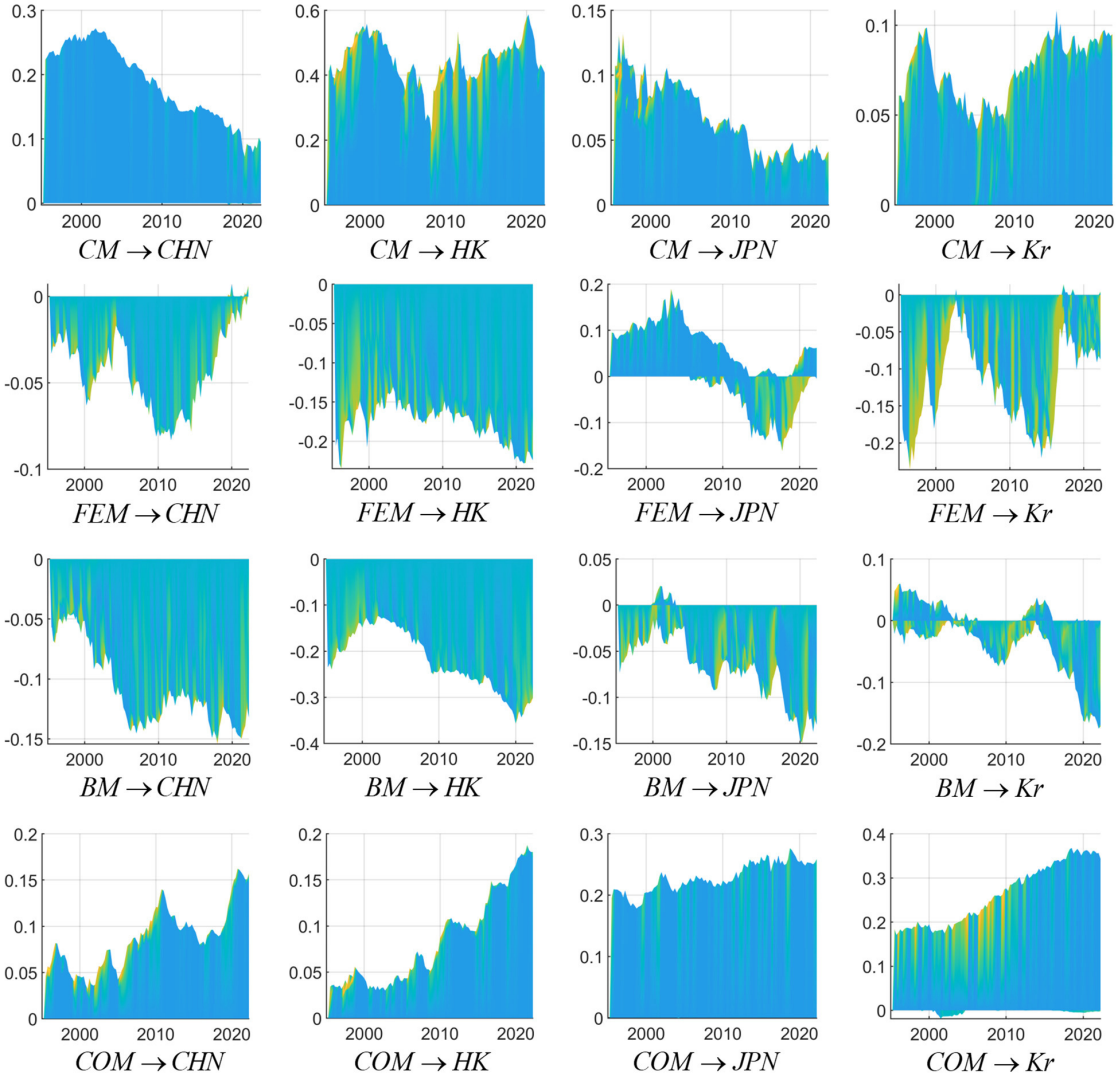
The interactions between the external financial markets and the stock markets of China, Hong Kong, Japan, and South Korea. Sources: Author's own calculations. CHN represents China stock market, H.K. represents Hong Kong stock market, JPN represents Japan stock market, and Kr represents South Korean stock market, CM represents commodity markets, FEM represents foreign exchange markets, BM represents bond markets and COM represents crude oil markets.

Figure 6 above demonstrates the interactions between the external financial market's impulse response and the stock markets of China, Hong Kong, Japan, and South Korea. The results show that the commodity market positively impacts China, Japan, and South Korea. Since the turn of the new century, commodities' impact on China's stock market returns has increased significantly. Around that time, China joined the World Trade Organization (WTO), and as generally seen in emerging economies, its demand for commodities soared. Then, the commodity market fell slowly and rose again. This market reached its peak during the global economic crisis, corresponding to the incredible increase in commodity prices that has occurred since 2002. During this period, the CRB commodity index increased by 112%, and the impact on China's stock market yield also reached a short-term peak. When the global financial crisis broke out in 2008, similar to the linkage between China, Japan, and South Korea, the commodity market's yield spillover effect on China's stock market is also obvious. The changes in other stock markets were caused by the shift in commodity market yield, indicating that the normal transmission channels among markets when the crisis occurred broke down. After the third quarter of 2008, because the crisis spread, the return spillover increased, and China's stock market rose again and reached a positive peak. Then, the commodity prices were no longer rising and falling sharply but turning into continuous fluctuations, and the effectiveness of China's macro policy regulation, the “financial firewall” played its due role, indicating that the stock market is no longer too sensitive to external shocks and the interaction between commodities and China's stock market. The market has declined slowly. The change in commodity market returns has an analogous impact on the stock markets of Hong Kong and South Korea because these stock markets are similar. After the Asian financial crisis, most Asian countries fell into recession. However, Hong Kong, South Korea, Singapore, and Taiwan implemented export-oriented strategies, persisted in developing the manufacturing and processing industries, achieved economic recovery relatively quickly, and blossomed into essential economies in Asia during a period of extreme growth. Thus, these economies were called the “four little dragons of Asia.” The Hong Kong and South Korean stock markets have a relatively consistent range and cycle, and both opened in the 1990s. The market value of the Hong Kong stock market is the highest among “The Four Tigers of Asia,” and “the commodity market” is more prominent. By observing the commodity market's return spillover to the Japanese stock market, we can find that the dynamic change trend after the global economic crisis is similar to that of China's stock market.

The exchange rate's spillover effect on the stock market is ultimately realized through the international flow of funds. This paper chooses the U.S. dollar's real effective weighted exchange rate to represent the foreign exchange using the direct pricing method. One unit of positive impulse represents an appreciation of the U.S. dollar and the devaluation of other countries' currencies, indicating that capital in the international market tends to flow back to the U.S., resulting in a reverse spillover between the exchange rate and the stock index. From a horizontal perspective, the Hong Kong stock market is most affected by a change in the U.S. dollar exchange rate related to Hong Kong's financial system. The Chinese mainland stock market has so far not fully opened its capital accounts. Hong Kong stocks are vital financial windows allowing the Chinese mainland stock market to communicate with other countries. When an appreciation of the dollar leads to a reflow of capital by a wide margin, the Hong Kong stock market is less stable than that of sovereign countries, and it is most affected by the foreign exchange's yield spillover effect. In terms of the timing of the dynamic change trend of the stock market caused by the price information transmission of the foreign exchange market, the maximum negative impact on the stock market in diverse regions appeared after the global financial crisis, indicating that the change in stock returns caused by changes in the foreign exchange market has a notable time lag. The interconnection of financial markets includes the interconnection between many financial markets and stock markets. Among them, the bond market spanning the inter-bank market and the exchange market is an important channel connecting the money market and the capital market, and it is also the difficulty and focus of promoting the interconnection of financial markets. In theory, the crude oil commodity market will affect the operating performance of listed companies in the relevant industrial chain and have an impact on the stock market, and the stock market, as a barometer of macroeconomic operations, can also affect the pricing of the crude oil market. On the other hand, in the context of the globalization of financial markets, the cross-market liquidity leads to a certain correlation between the two markets, especially when a global liquidity crisis emerges. Therefore, it is necessary to incorporate the interaction between the crude oil market and the stock markets of China, Japan and South Korea into the research framework. The spillover of the bond market and the crude oil market to the stock markets of China, Japan and South Korea is further discussed. It can be seen from the results that on the whole, the bond market has a negative reaction to the stock markets of the three countries, while the crude oil market has a positive reaction to the stock markets of the three countries.

### Robustness check

Previous sections presented conclusions regarding the return spillovers between the stock markets of China, Japan, and South Korea and the impact of other financial markets on these stock markets. To verify these conclusions, the robustness of the results must also be verified; when the model specifications change, the major conclusions noted in the paper should not vary significantly. Therefore, this section uses different methods to test the robustness of the above-mentioned findings to ensure that the conclusions are stable and reliable. To ensure the brevity of the article, all robustness test results are given in the Supplementary material.

#### Alternative VAR ordering

For our baseline model, we refer to the process proposed by Primiceri (47). To facilitate model estimation, Cholesky decomposition is used: the order of the proxy variables is $Y_{t}^{baseline}=\left[In{\left(China\right)}_{t}\;In{\left(HongKong\right)}_{t}\;In{\left(Japan\right)}_{t}\;In{\left(Korea\right)}_{t}\right]$. To ensure that the model can still obtain analogous results when changing the order of the proxy variables, we change the variable order of $Y_{t}^{baseline}$ and construct $Y_{t}^{alternative1}$. Thus, the order differs from the original. The relevant results of the original model are compared with those of the first and second alternative models with a changed order of variables. It can be seen that changing the order of variables will cause subtle changes in the empirical results but will not affect the main conclusions obtained in the article.

#### Alternative lag ordering

Another way to measure the robustness of the model is to vary the lag length of the model. In the preceding section, we choose the first-order lag model with the best fit. In this part, the lag length of the model is changed to the second order. Using empirical simulation, when the order of the surrogate variables remains unchanged, the model becomes a second-order lag, regardless of whether we obtain similar consequences of the return spillover effect between China, Japan, and South Korea. Due to space limitations, the test results are not listed. All the empirical results have been kept for reference.

#### Replace key variables

In order to further test the robustness of the model results, the SZI index is used as the proxy variable of the Chinese stock market, and the Chinese stock market is re-simulated with the Hong Kong stock market, the Japanese stock market, and the Korean stock market. The results show that the use of SZI does not affect the main conclusions of this paper. Furthermore, the relationship between Chinese stock market and external financial markets such as commodity market, foreign exchange market, bond market and crude oil market is re-simulated. It can be seen that the results obtained are still similar to those in the paper, which can prove the robustness of the model.

### Comments

The findings on the spillover effect of returns among the stock markets of China, Japan, and South Korea are supported by the results of the robustness test. The three different robustness tests gave the same result, that is the empirical conclusions are similar to the results of the original benchmark model. Specifically, the response amplitude of each model is analogous.

Moreover, the different models show that when the positive impact of one unit in the return rate of one country's stock market interacts with another stock market, the response value of the second period of the impact is the largest, and the impact effect decreases gradually. In the eighth period, the response value of most stock markets returns to zero, which indicates that the price information transmission between stock markets caused by a change in the return rates has only a short-term effect and no long-term effect. Our robustness test verifies the original assumption and enhances the credibility of the conclusion of this paper. That is, the influence of China's stock market on the stock markets of other countries in the East Asian economic region is increasing. Nevertheless, the spillover effect of China's stock market on the stock markets of Japan and South Korea is still smaller than that between Japan and South Korea. When severe external shocks occur, the price transmission between stock markets will lead to a different response than occurs in other periods.

## Conclusions

China, Japan, and South Korea are all located in the East Asia region and have close economic and trade ties as well as geographical proximity. However, due to various complicated factors, the East Asian Free Trade Area has not been fully realized. In 2020, COVID-19 suddenly swept the world, significantly affecting various countries' economies. Considering this background and the financial openness of China, Japan, and South Korea, in this paper, the stock market is chosen as the research object, the price information transmission among the three countries is analyzed and the possibility that further financial interconnections exist among the three countries is explored. Additionally, in this paper, the Asian financial crisis and the global economic crisis are selected as particular time points for the study. A framework containing numerous factors affecting stock market spillovers is employed as the benchmark, and this study assesses how the return spillovers among the Chinese, Japanese, and Korean stock markets change when severe external shocks occur, which has implications for the regional economic reconstruction of East Asia in the post-epidemic era.

This paper's main conclusions are as follows: First, the return spillover of China's stock market to stock markets in the surrounding areas is increasing due to the advancement of capital market openness. When China's stock market is relatively closed, the exchange of price information with the surrounding markets occurs mainly through the Hong Kong stock market. Various policies have been implemented to promote foreign investors' investment in China, and the influence of China's stock market has been dramatically strengthened. As a result, dependence on the “window” role of the Hong Kong stock market has been reduced, which indicates that the return spillover effect of China's stock market on the Japanese and South Korean markets has been almost equal to that of the Hong Kong stock market in recent years and shows an increasing trend. However, the return rate spillover from China's stock market to Japan's and South Korea's stock markets is still less than that between Japan's and South Korea's stock markets, which indicates that the future promotion of regional economic integration in East Asia requires China to further open its financial market on the premise of preventing systemic risks and promoting inter-regional capital circulation. Second, during the Asian financial crisis and the global economic crisis, the linkage between the markets deepened with the spread of the crises, which is consistent with the mainstream view. Due to the prevalence of the crisis, panic infected various markets, and the spillover effect was further enhanced. Third, to clarify how the stock markets of China, Japan, and South Korea interact with other financial markets beyond their mutual spillover effects and to provide a theoretical basis for the economic integration of East Asia, this paper further analyses the similarities and differences of the three nations' stock markets from the perspective of internal and external markets. The results demonstrate that the commodity market and crude oil market tends to have positive interactions with the three countries' stock markets, while the foreign exchange market and bond market has negative interactions. The Hong Kong stock market is most affected by external financial market shocks, which plays a key role in the strategic positioning of its financial window and external-oriented economy and its greater instability in the face of external shocks compared to the stock markets of sovereign countries.

In view of the conclusions on the stock markets of China, Japan and South Korea. First, it is increasingly urgent to strengthen regional monetary synergism, and for financial and economic stability, collaboration within the region of East Asia is essential. Cooperation within East Asia is essential to maintain financial and economic stability. The impact of COVID-19 has been unprecedented. The impact of the epidemic has put the world economy on a downward trend and brought unprecedented difficulties to global monetary and financial cooperation. In this context, strengthening regional monetary and financial cooperation is expected to revitalize and transcend economic globalization. Second, China needs to enhance its communication with other East Asian countries regarding market rules and systems. There are distinct differences in the stock return trends of China, Japan, and South Korea caused by the two financial crises considered in this paper. The global economic crisis impact is unprecedented; it destroyed the normal transmission channels that exist between financial markets. The effect of COVID-19 is also remarkable, and it is likely to accelerate the world economic recession. We need to learn from the impacts of previous financial crises on the financial market and consider the past as well as the current reality. Therefore, in the post-pandemic era, it is an important direction for East Asian regional economic cooperation to shift from the traditional negotiation of market access barriers to the construction of longer-term market rules and institutions. Finally, it is necessary to be aware that monetary and financial systems are highly dependent on institutions, and institutional defects cannot be addressed merely through technology. Thus, more effort needs to be exerted to promote regional financial cooperation by developing top-level institutions. Our observation of the linkages among the stock markets of China, Japan and South Korea and other financial markets indicates that there are still promising prospects for China's financial market and its advancement. The internationalization of a country's currency requires a developed financial market with breadth and depth to provide the foundation for the efficient allocation of resources. In addition, more secure and diversified financial products are needed so that investors can hedge their risks at a low cost and retain their earnings.

## Data availability statement

The original contributions presented in the study are included in the article/Supplementary material, further inquiries can be directed to the corresponding author.

## Author contributions

SW: conceptualization, methodology, software, formal analysis, data curation, writing—original draft preparation, writing—review, and editing. BZ, QY, and TL: validation. BZ: investigation, resources, supervision, project administration, and funding acquisition. QY: visualization. All authors contributed to the article and approved the submitted version.

## Funding

This work was funded by Philosophy and Social Science Research Innovation Team Project (Grant No. 2022CXTD04) and the National Natural Science Foundation of China (Grant No. 11901233).

## Conflict of interest

The authors declare that the research was conducted in the absence of any commercial or financial relationships that could be construed as a potential conflict of interest.

## Publisher's note

All claims expressed in this article are solely those of the authors and do not necessarily represent those of their affiliated organizations, or those of the publisher, the editors and the reviewers. Any product that may be evaluated in this article, or claim that may be made by its manufacturer, is not guaranteed or endorsed by the publisher.
