# Supplementary figures and images for: Research on the dynamic spillover of stock markets under COVID-19—Taking the stock markets of China, Japan, and South Korea as an example (part 1 of 3)
Source: Front Public Health. 2022 Nov 11;10:1008348. doi: 10.3389/fpubh.2022.1008348 (PMC9691647; doi:10.3389/fpubh.2022.1008348)

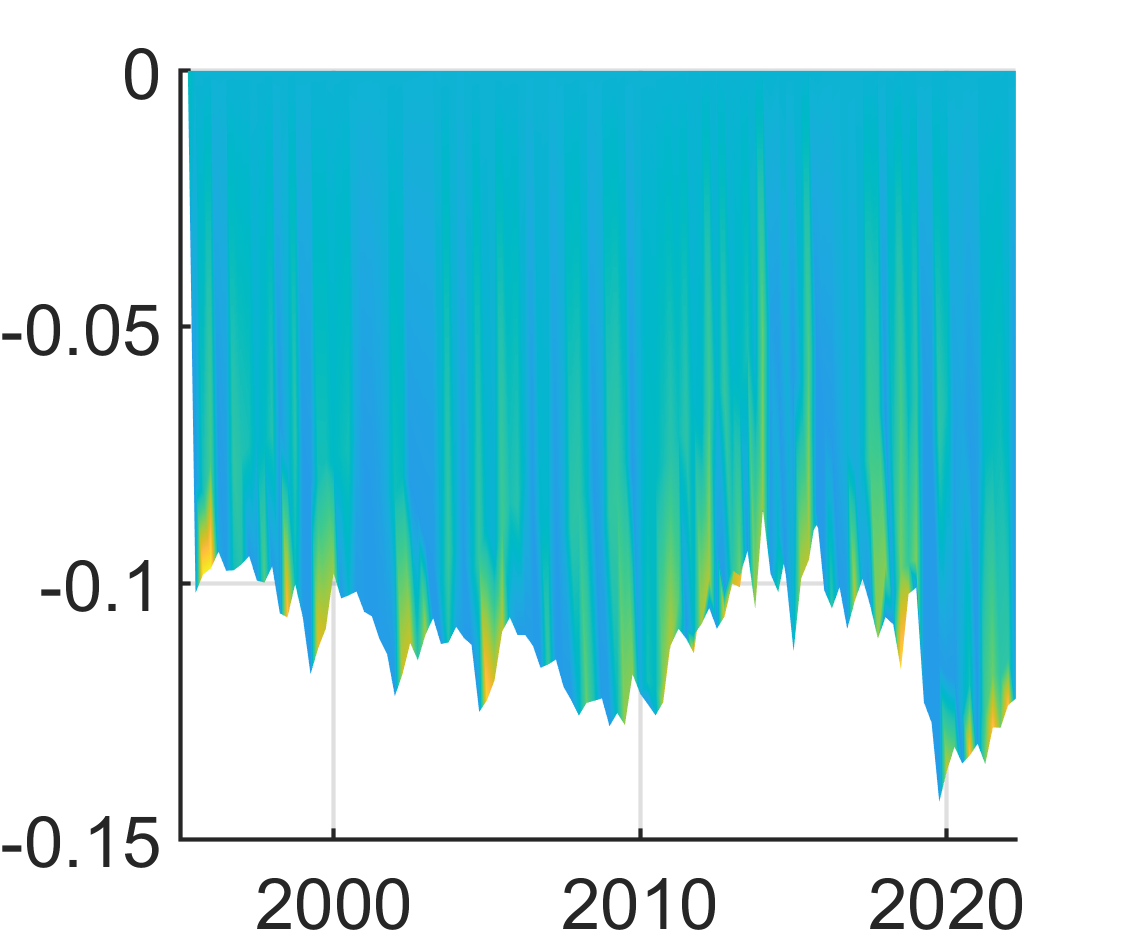

Supplement: Supplementary file 3 [file Data_Sheet_1.ZIP › BM_CHN_1 (1).tif]

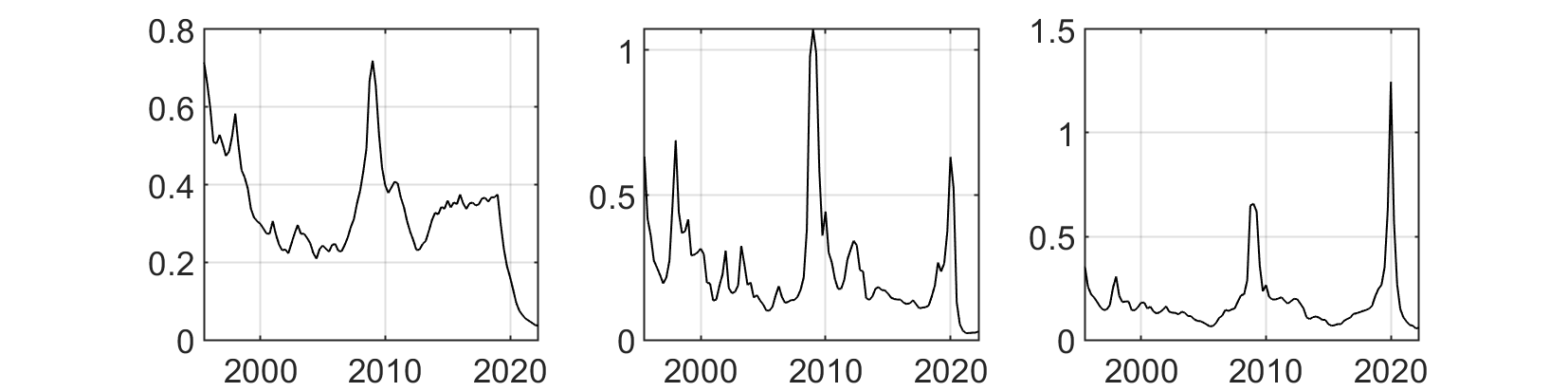

Supplement: Supplementary file 3 [file Data_Sheet_1.ZIP › BM_CHN_1 (2).tif]

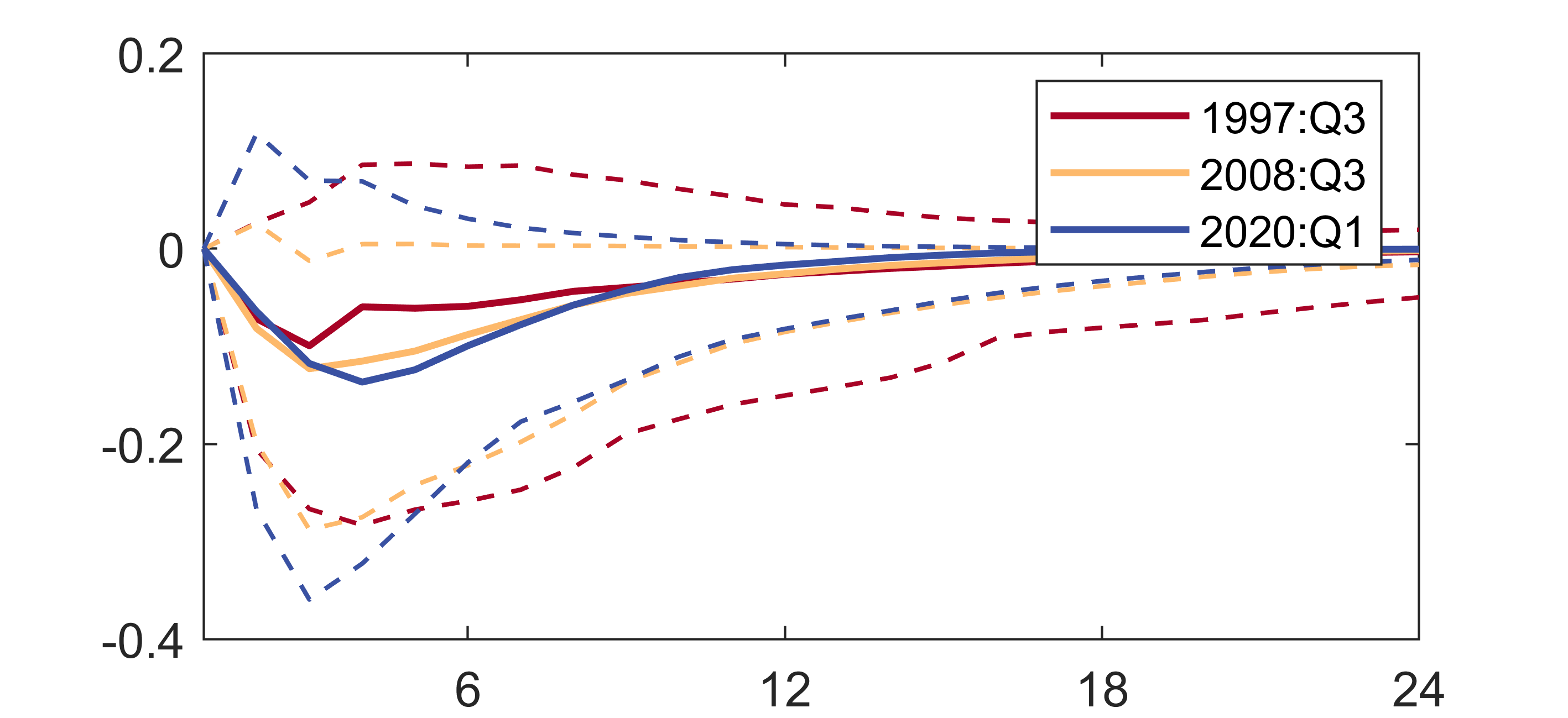

Supplement: Supplementary file 3 [file Data_Sheet_1.ZIP › BM_CHN_1 (3).tif]

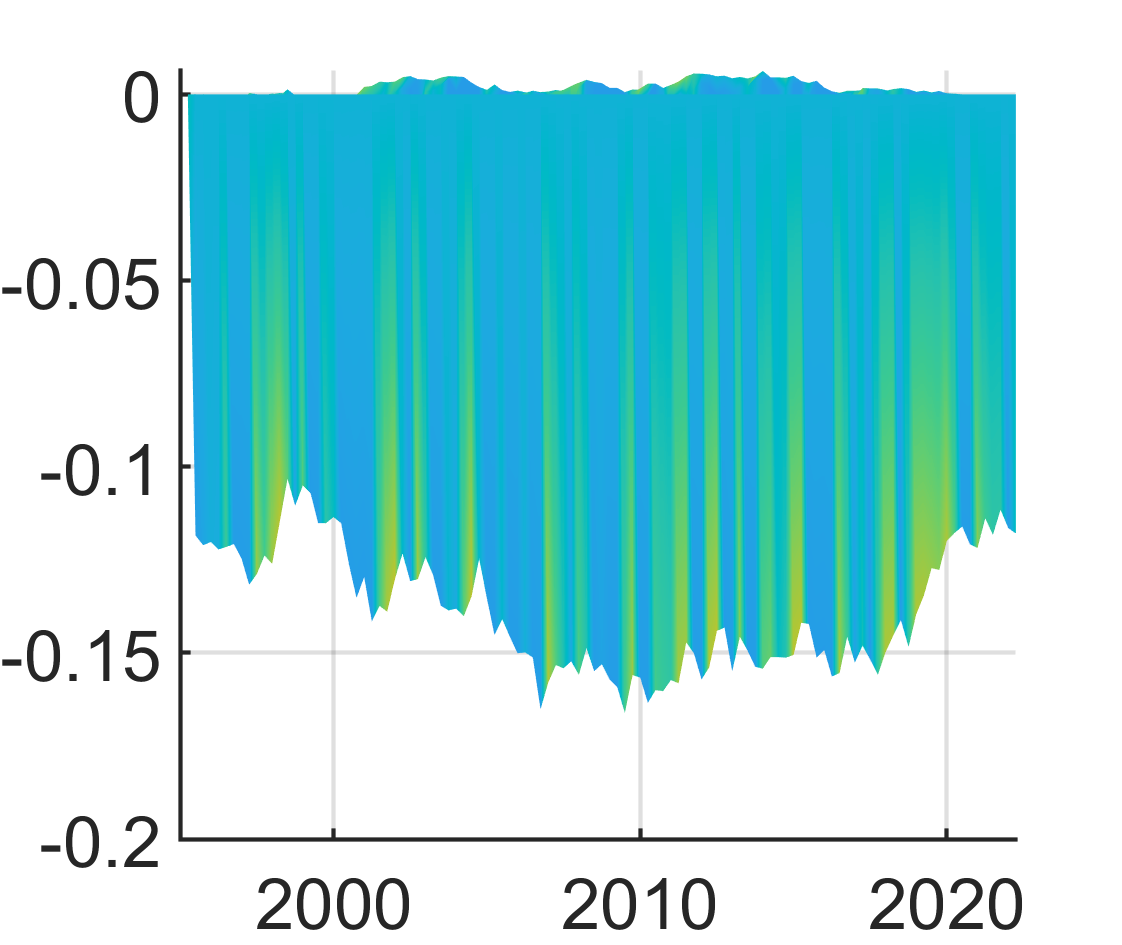

Supplement: Supplementary file 3 [file Data_Sheet_1.ZIP › BM_HK_1 (1).tif]

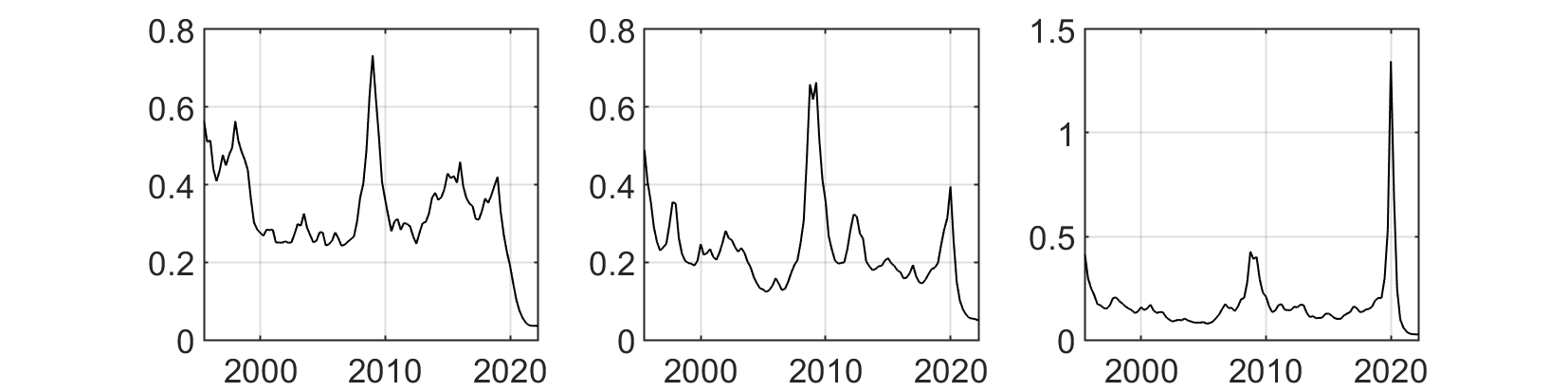

Supplement: Supplementary file 3 [file Data_Sheet_1.ZIP › BM_HK_1 (2).tif]

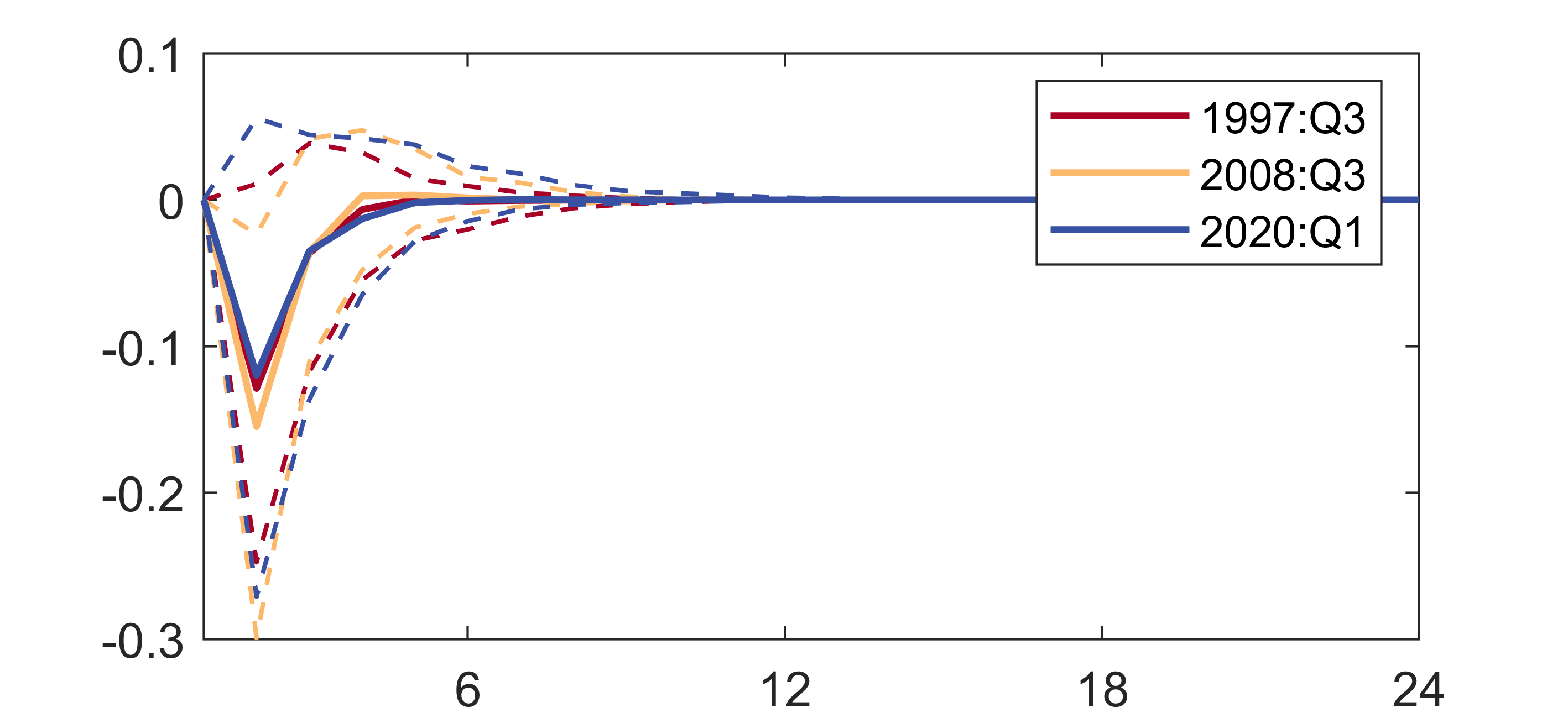

Supplement: Supplementary file 3 [file Data_Sheet_1.ZIP › BM_HK_1 (3).tif]

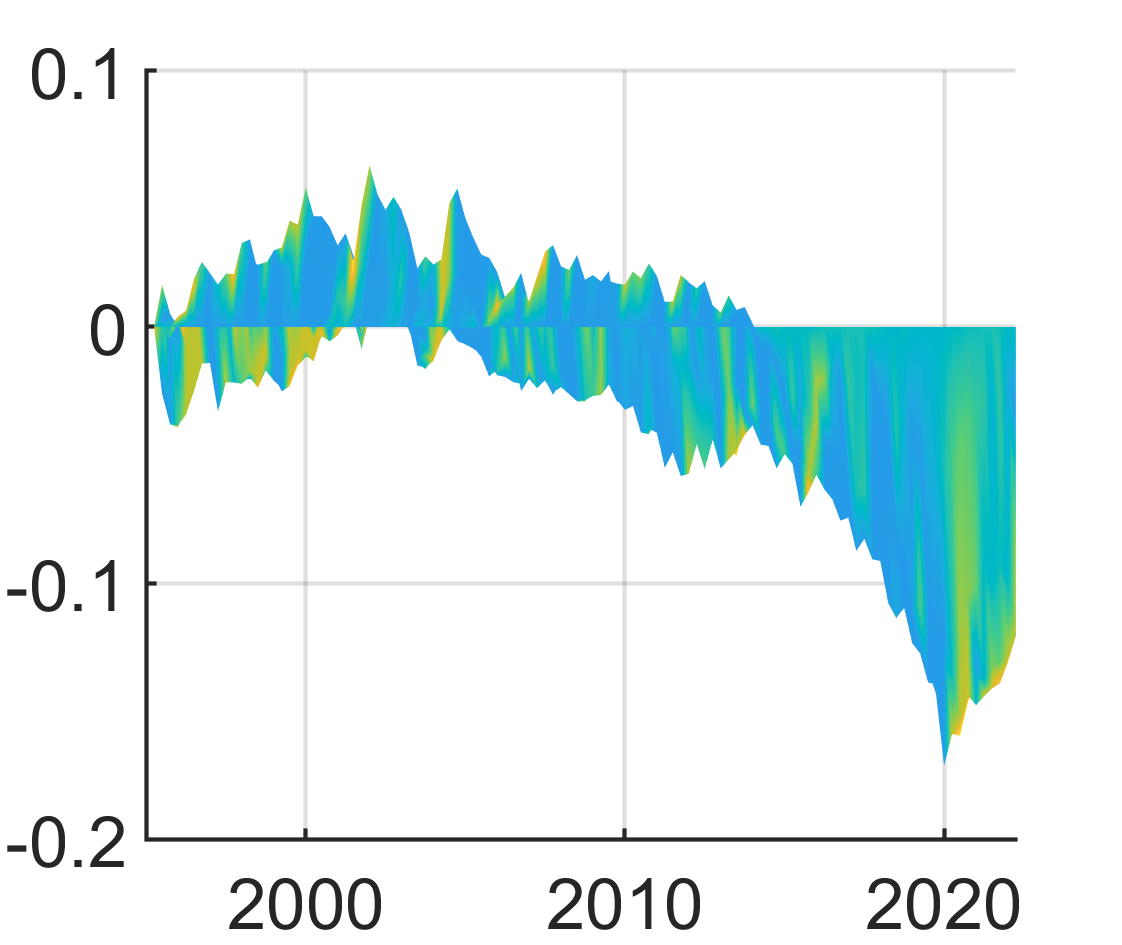

Supplement: Supplementary file 3 [file Data_Sheet_1.ZIP › BM_JPN_1 (1).tif]

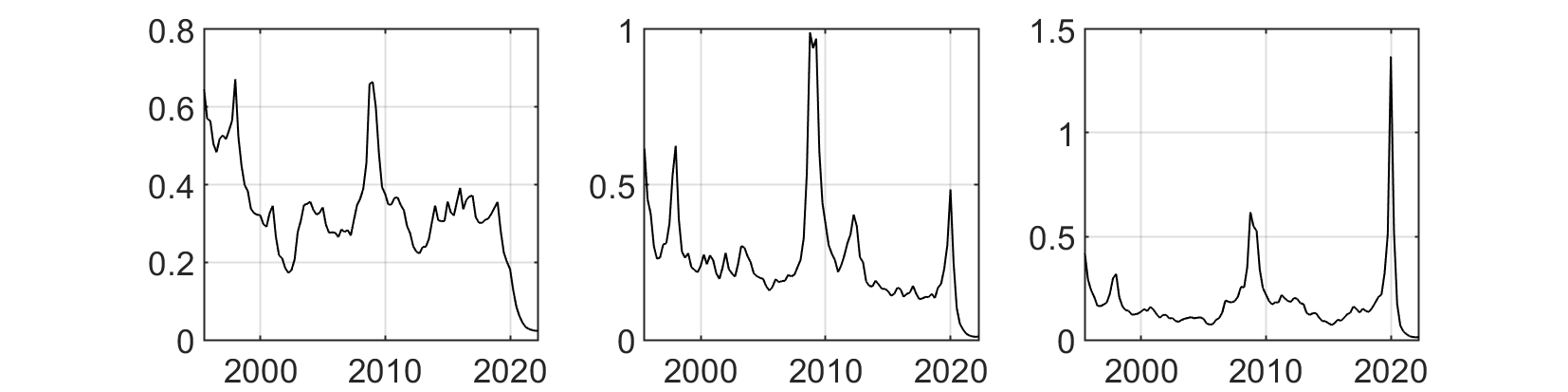

Supplement: Supplementary file 3 [file Data_Sheet_1.ZIP › BM_JPN_1 (2).tif]

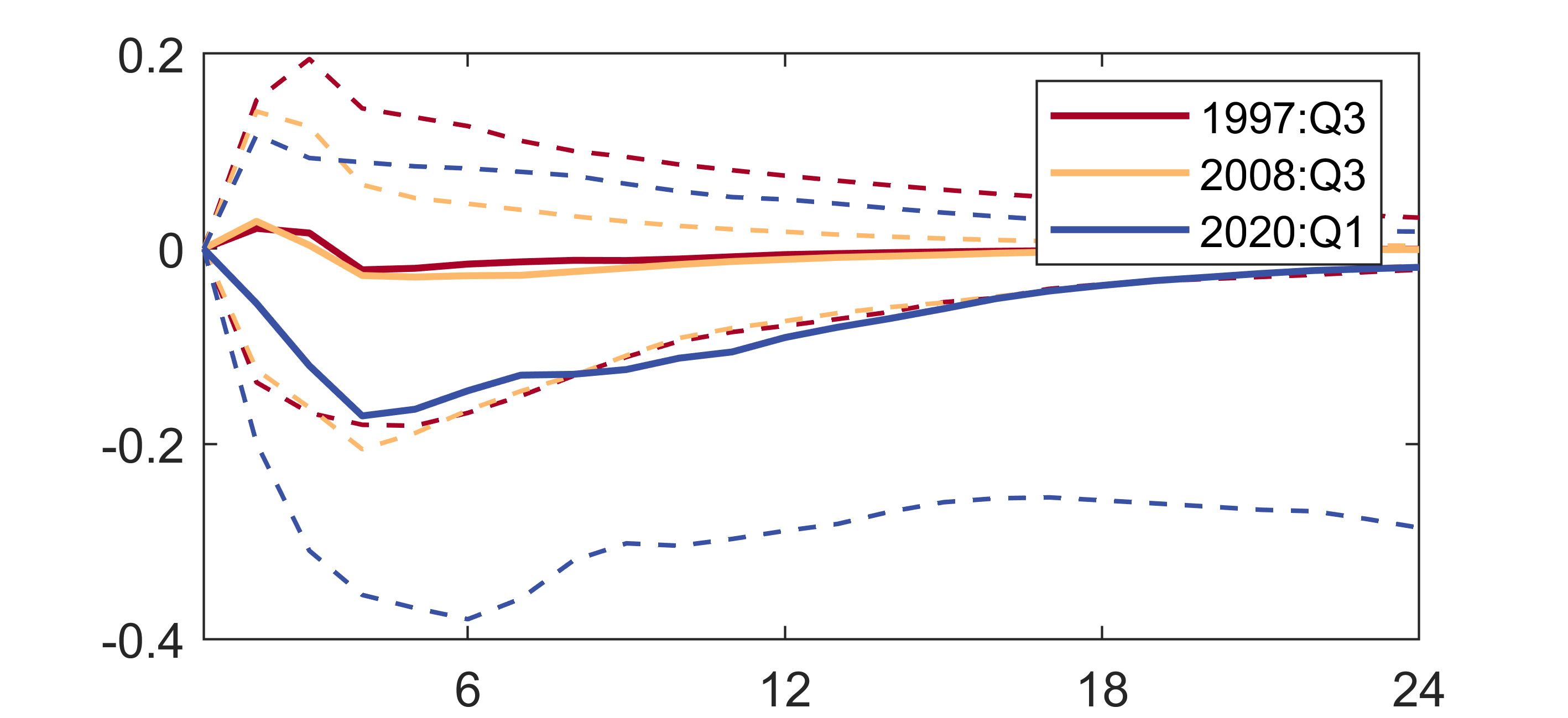

Supplement: Supplementary file 3 [file Data_Sheet_1.ZIP › BM_JPN_1 (3).tif]

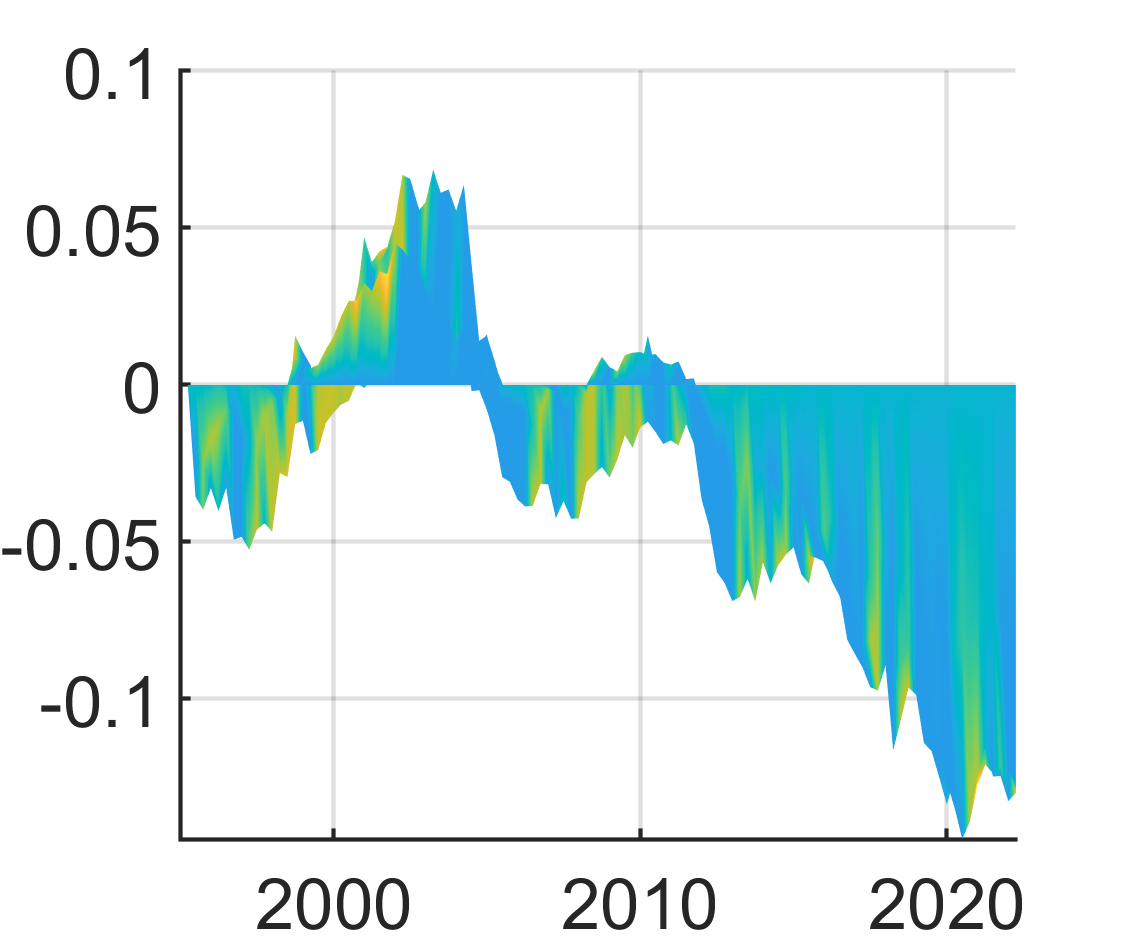

Supplement: Supplementary file 3 [file Data_Sheet_1.ZIP › BM_KR_1 (1).tif]

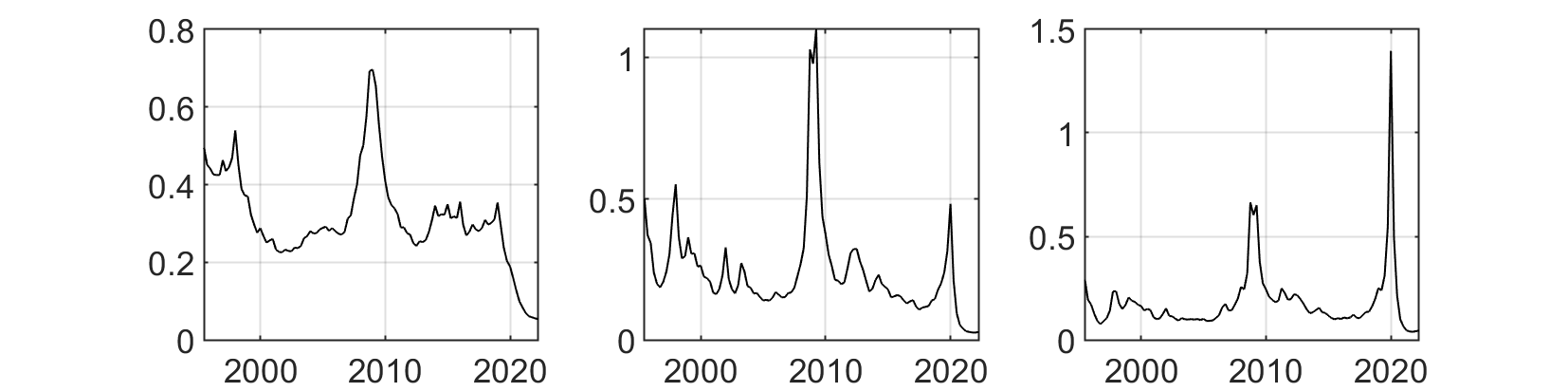

Supplement: Supplementary file 3 [file Data_Sheet_1.ZIP › BM_KR_1 (2).tif]

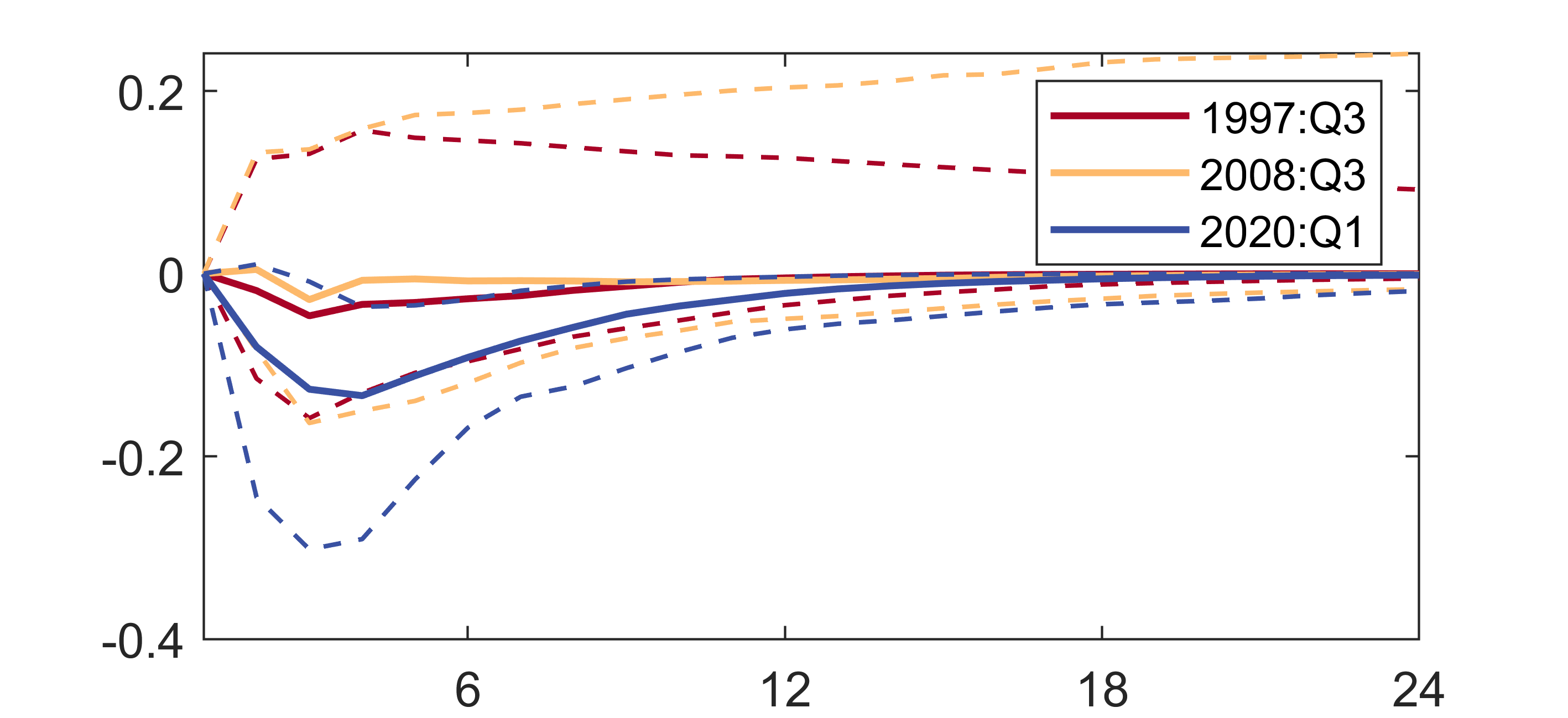

Supplement: Supplementary file 3 [file Data_Sheet_1.ZIP › BM_KR_1 (3).tif]

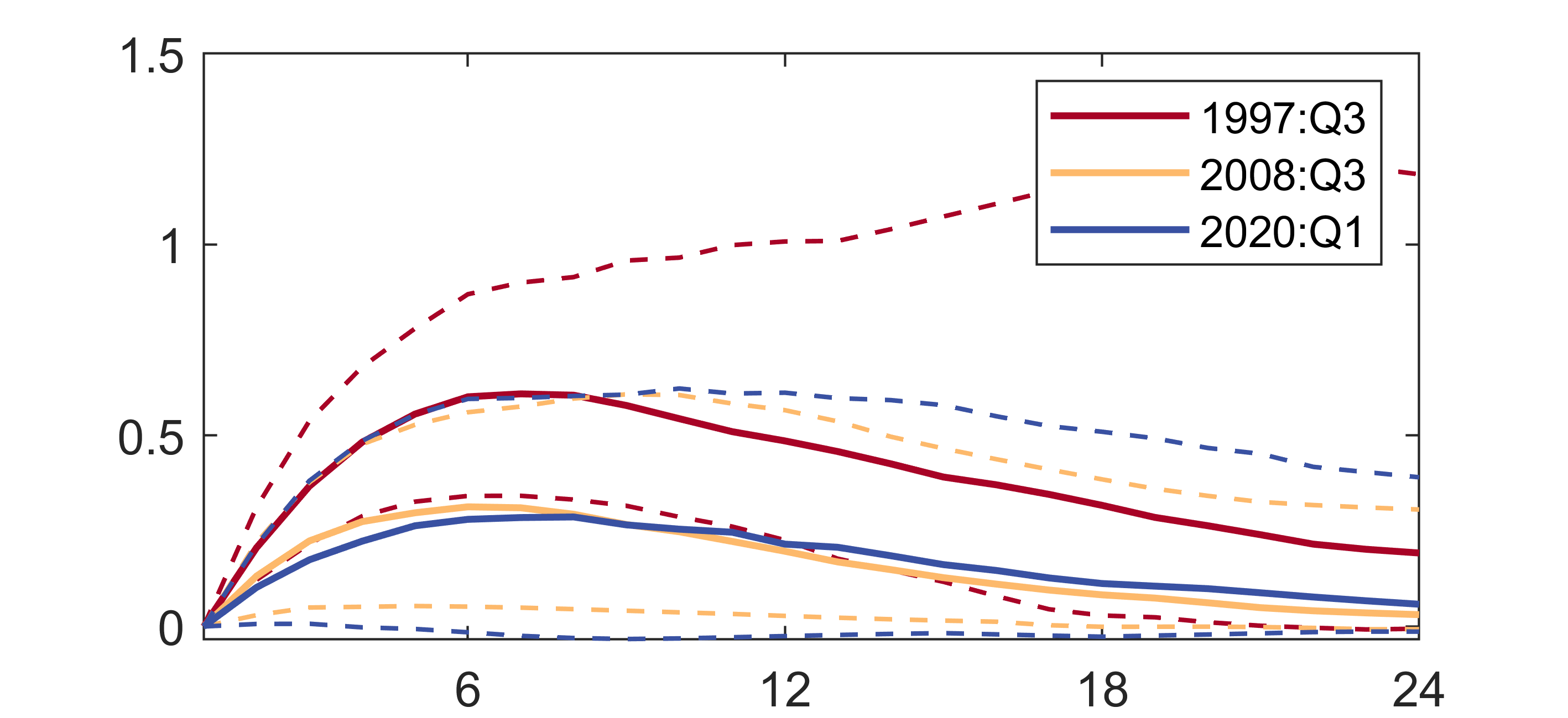

Supplement: Supplementary file 3 [file Data_Sheet_1.ZIP › CHN_HK_1 (1).tif]

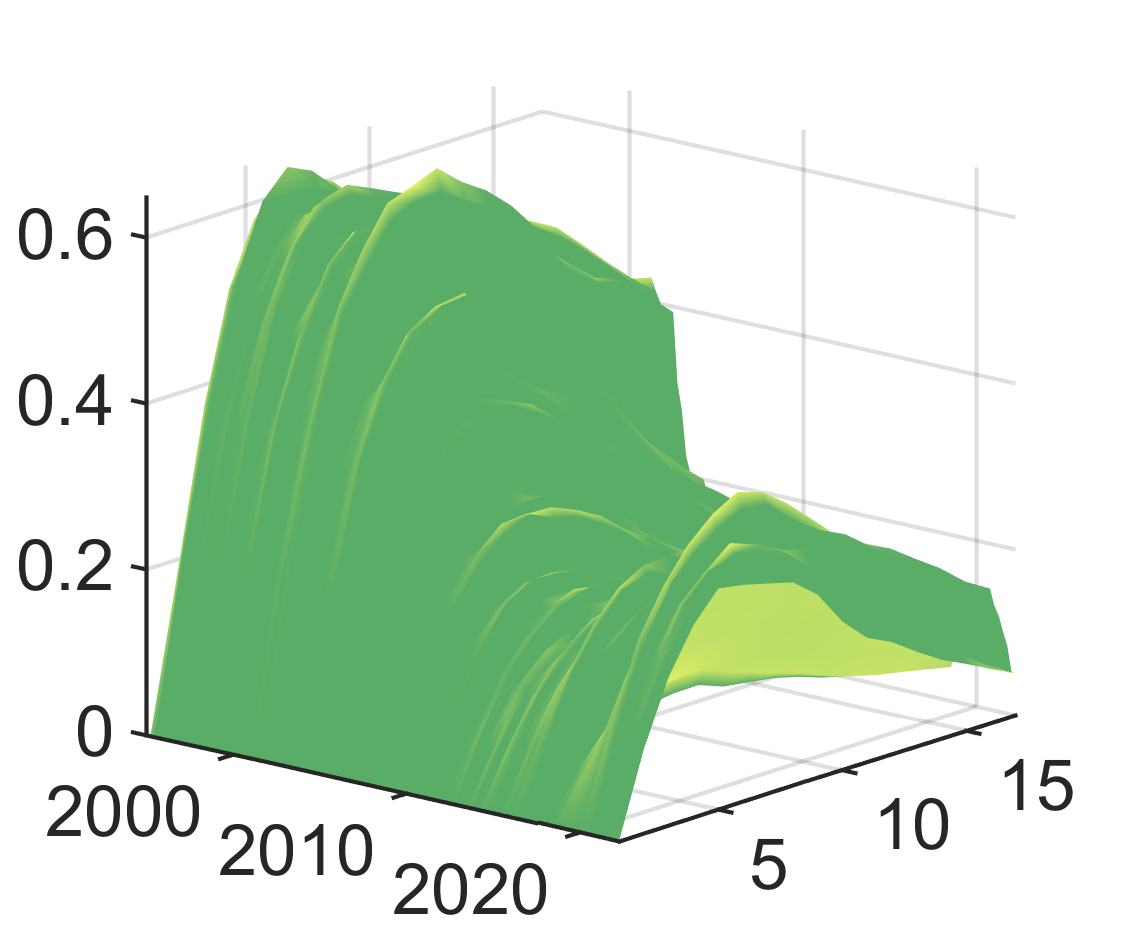

Supplement: Supplementary file 3 [file Data_Sheet_1.ZIP › CHN_HK_1 (2).tif]

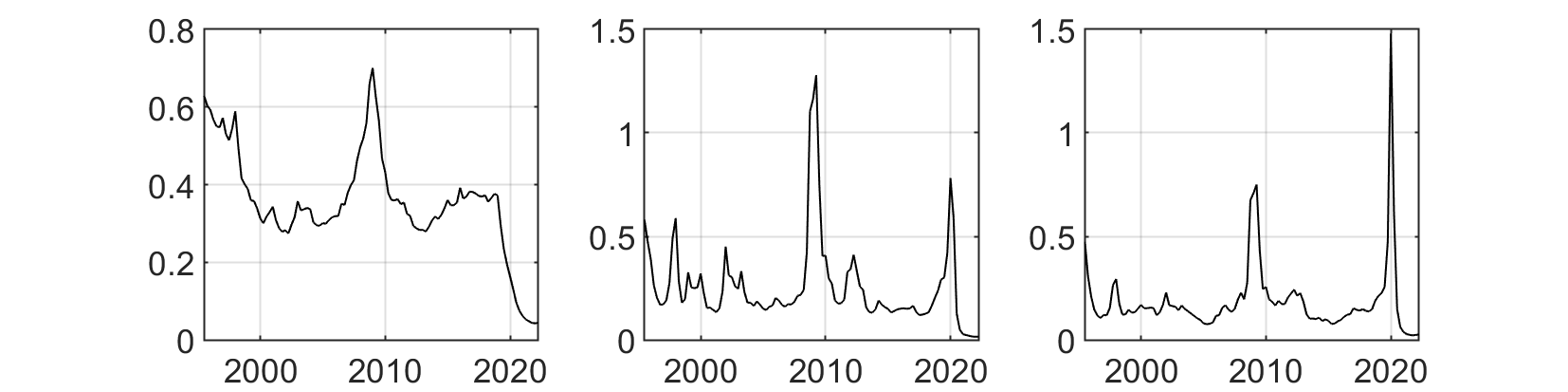

Supplement: Supplementary file 3 [file Data_Sheet_1.ZIP › CHN_HK_1 (3).tif]

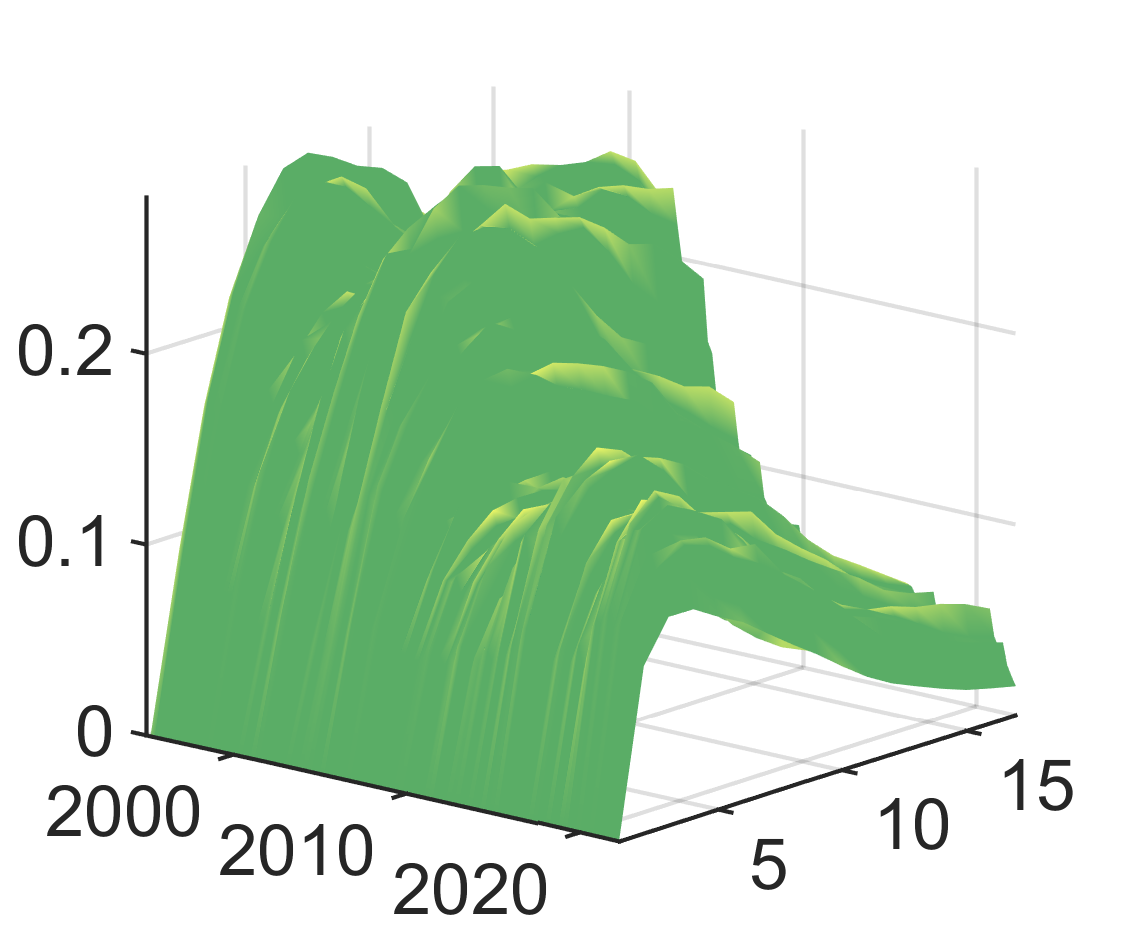

Supplement: Supplementary file 3 [file Data_Sheet_1.ZIP › CHN_JPN_1(1).tif]

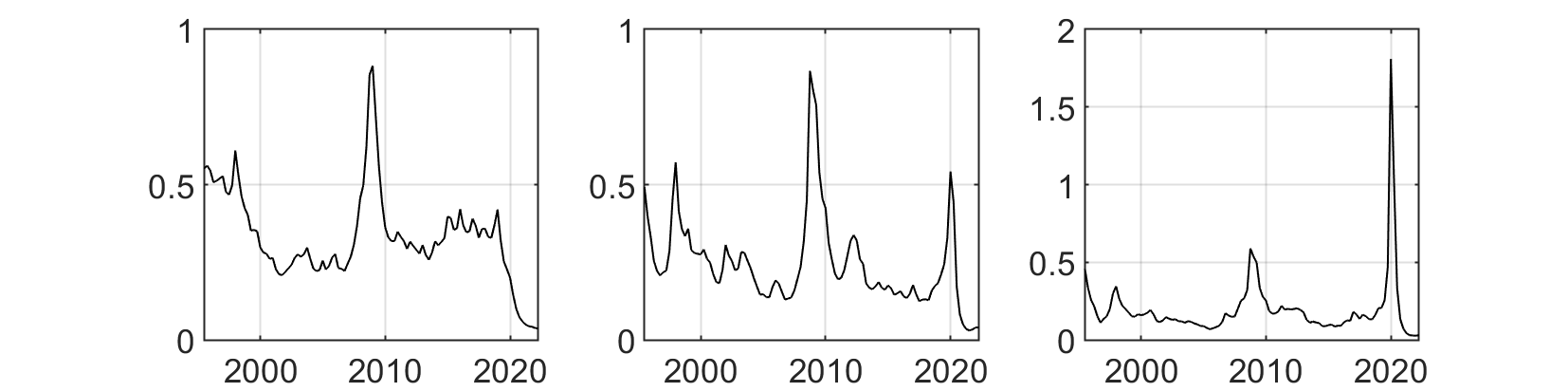

Supplement: Supplementary file 3 [file Data_Sheet_1.ZIP › CHN_JPN_1(2).tif]

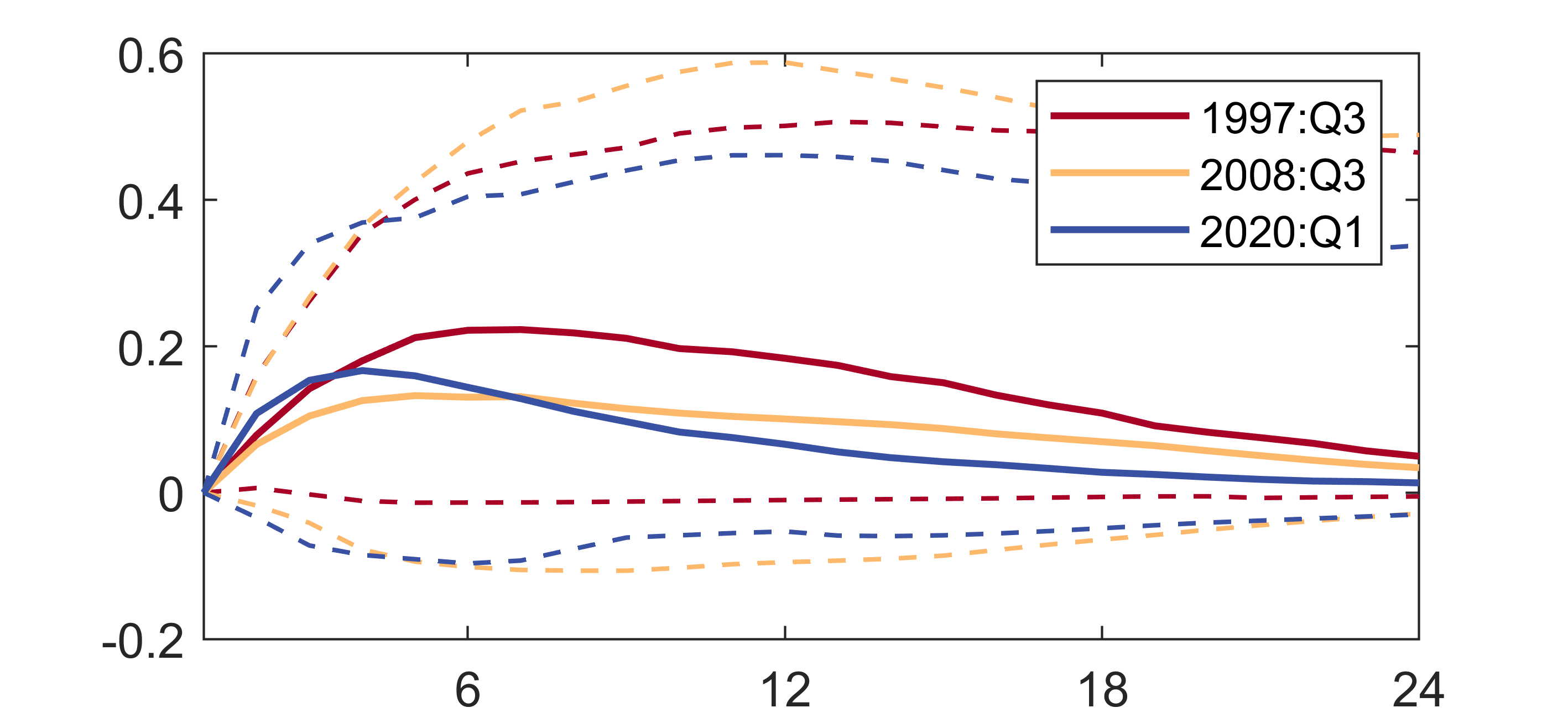

Supplement: Supplementary file 3 [file Data_Sheet_1.ZIP › CHN_JPN_1(3).tif]

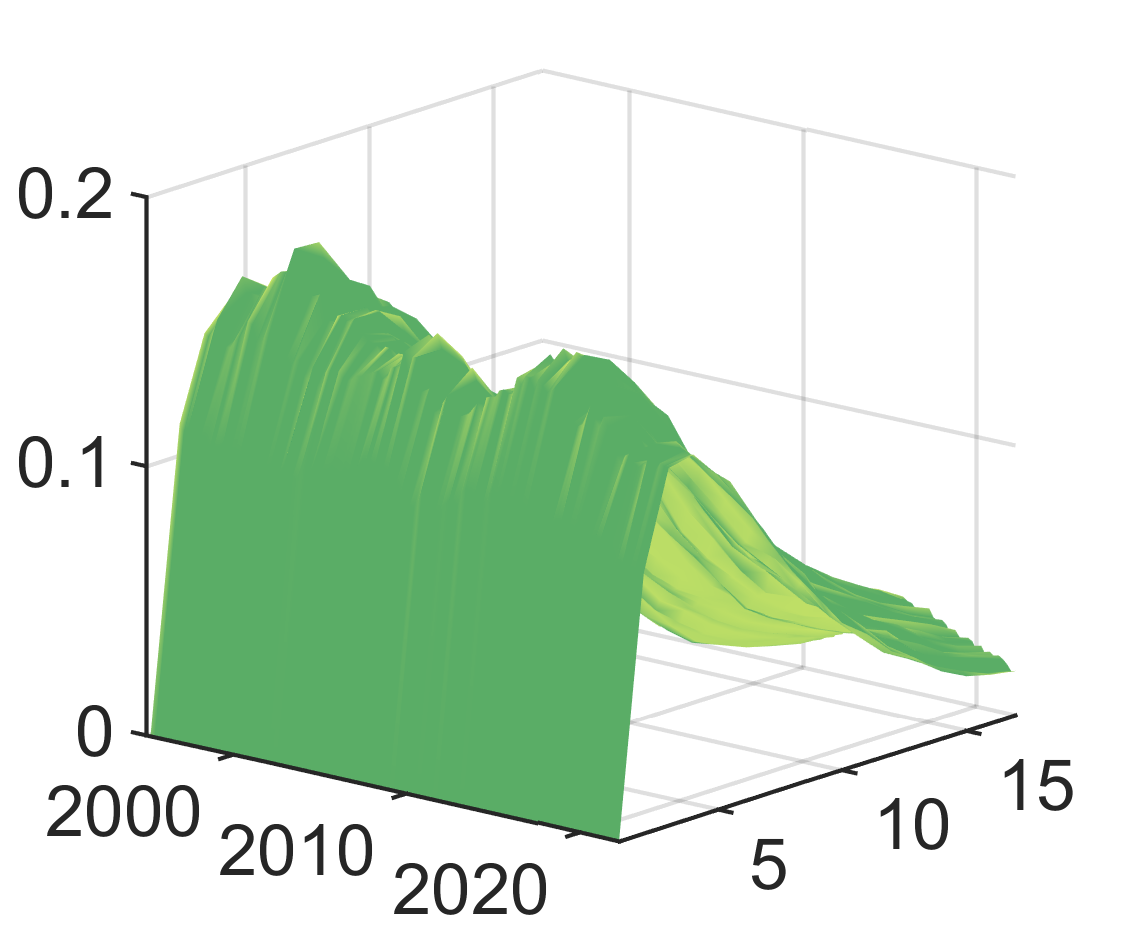

Supplement: Supplementary file 3 [file Data_Sheet_1.ZIP › CHN_KR_1 (1).tif]

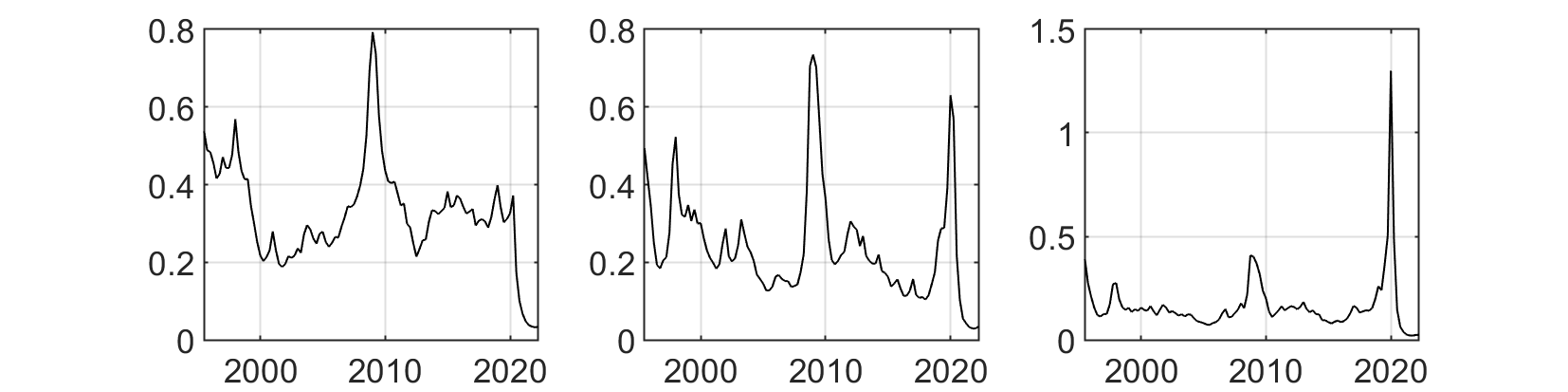

Supplement: Supplementary file 3 [file Data_Sheet_1.ZIP › CHN_KR_1 (2).tif]

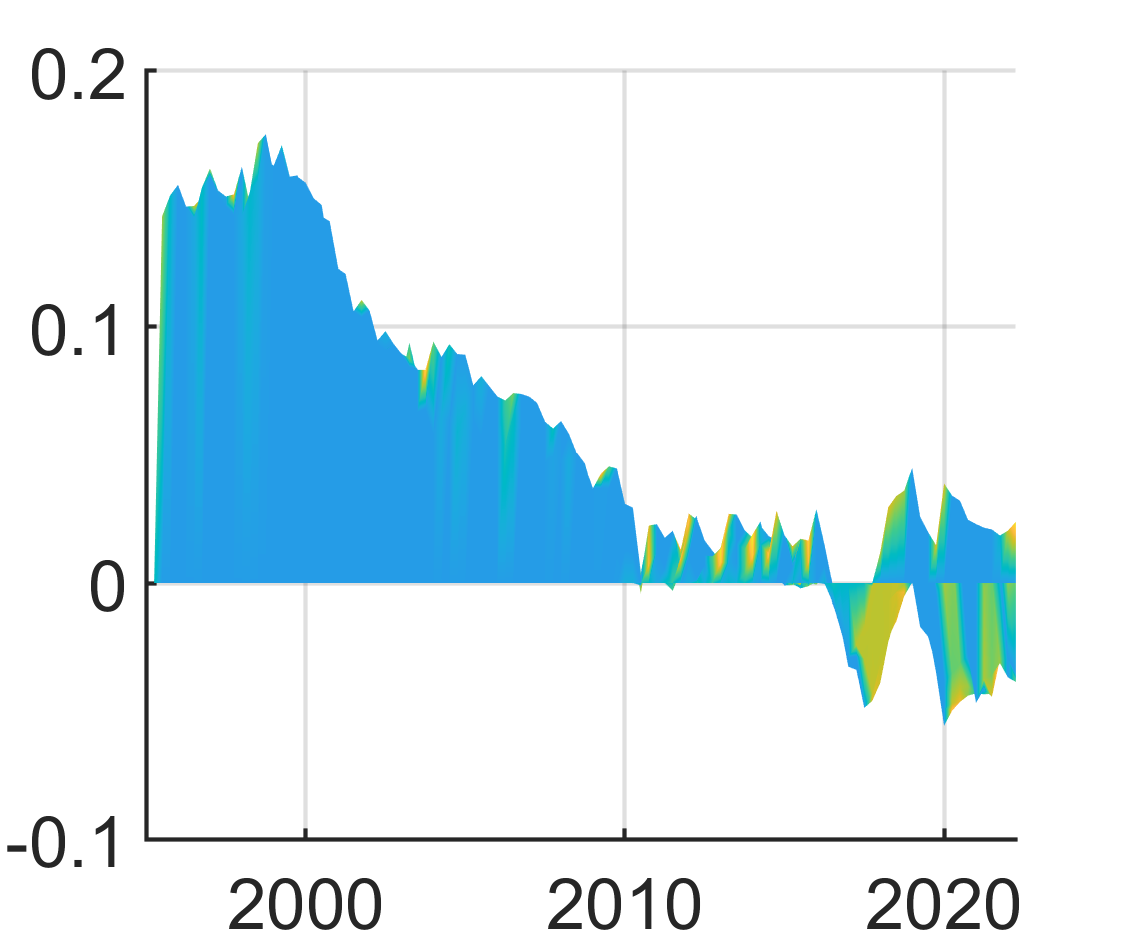

Supplement: Supplementary file 3 [file Data_Sheet_1.ZIP › CM_CHN_1 (1).tif]

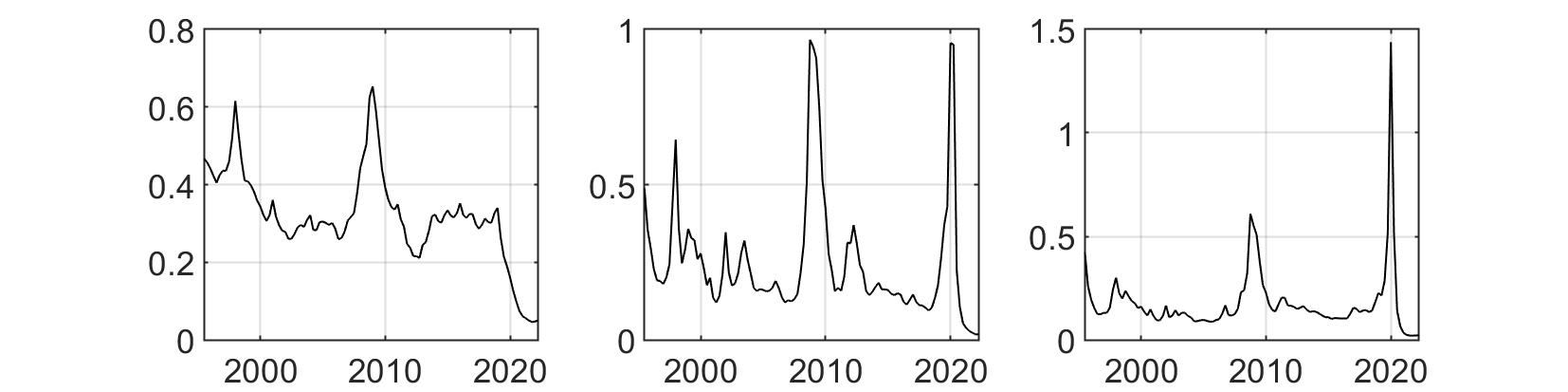

Supplement: Supplementary file 3 [file Data_Sheet_1.ZIP › CM_CHN_1 (2).tif]

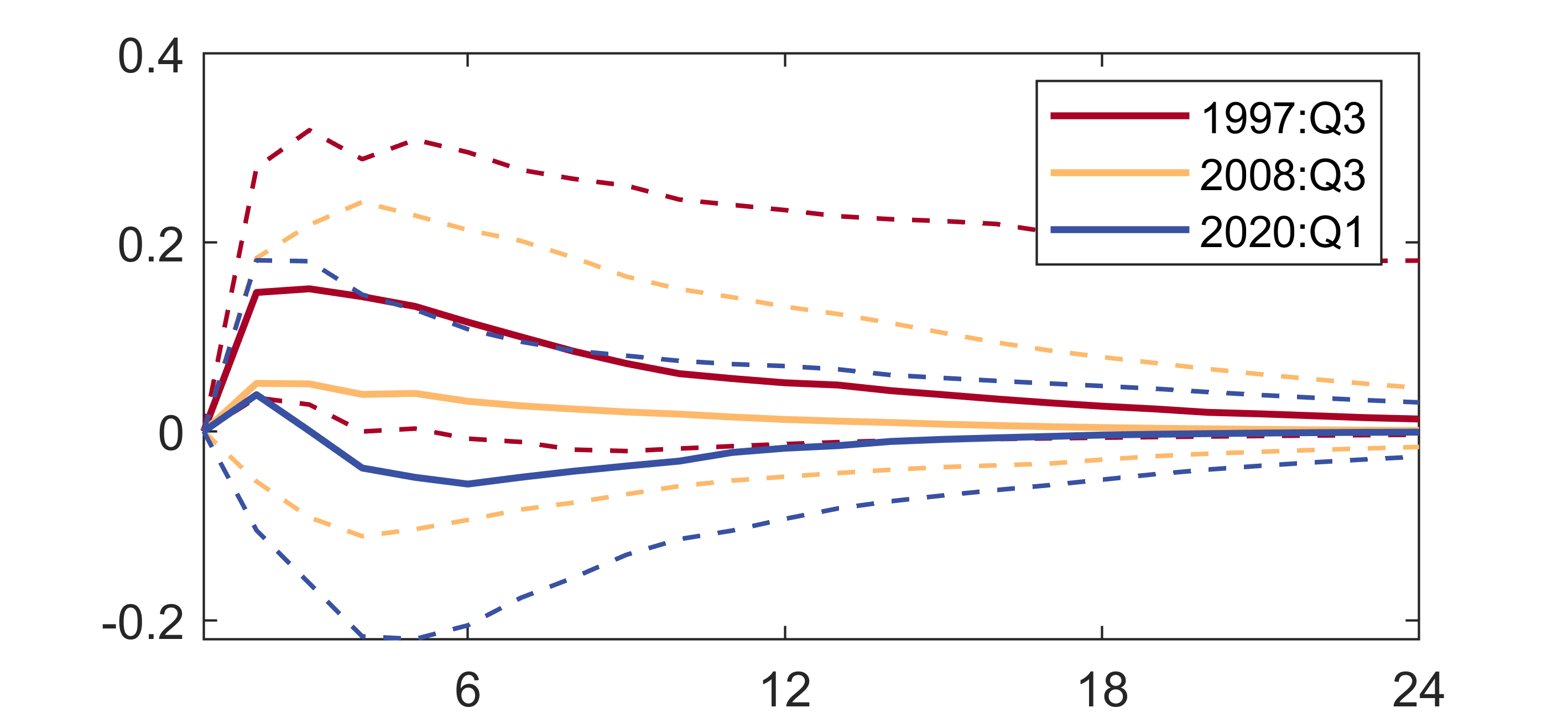

Supplement: Supplementary file 3 [file Data_Sheet_1.ZIP › CM_CHN_1 (3).tif]

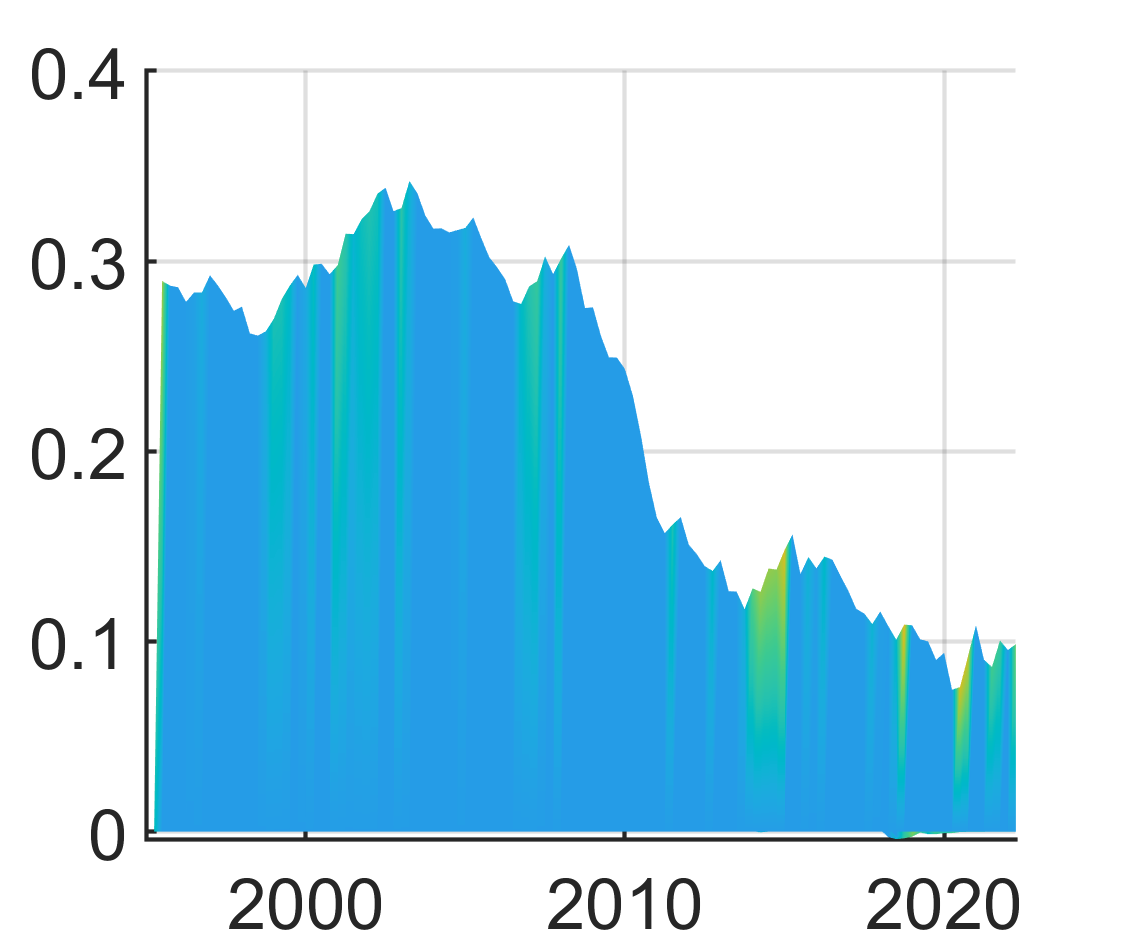

Supplement: Supplementary file 3 [file Data_Sheet_1.ZIP › CM_HK_1 (1).tif]

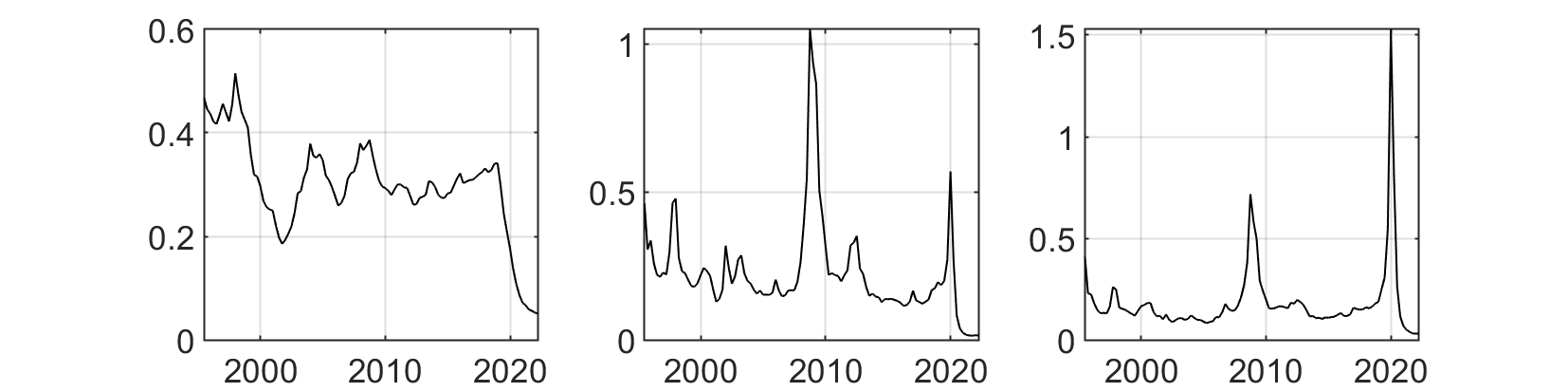

Supplement: Supplementary file 3 [file Data_Sheet_1.ZIP › CM_HK_1 (2).tif]

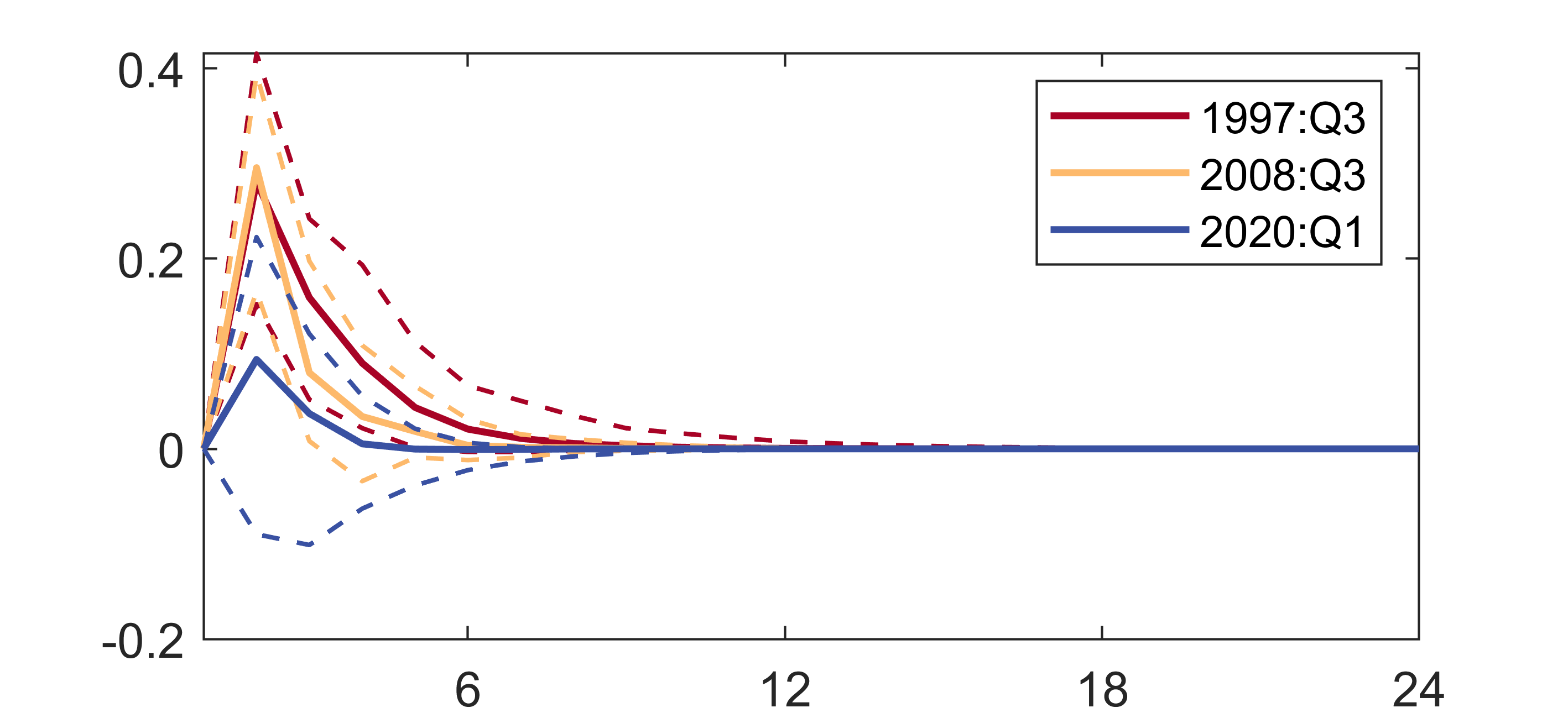

Supplement: Supplementary file 3 [file Data_Sheet_1.ZIP › CM_HK_1 (3).tif]

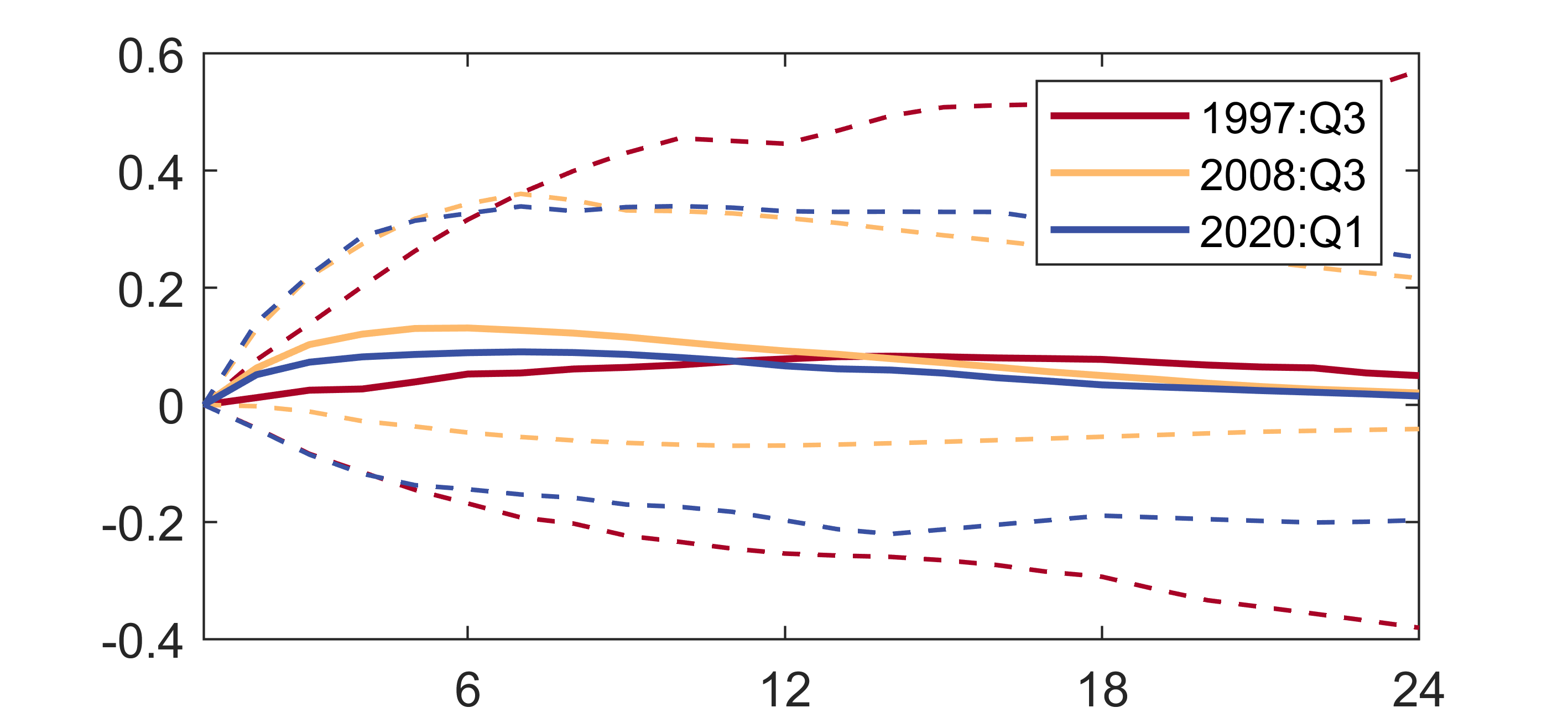

Supplement: Supplementary file 3 [file Data_Sheet_1.ZIP › CM_JPN_1 (3).tif]

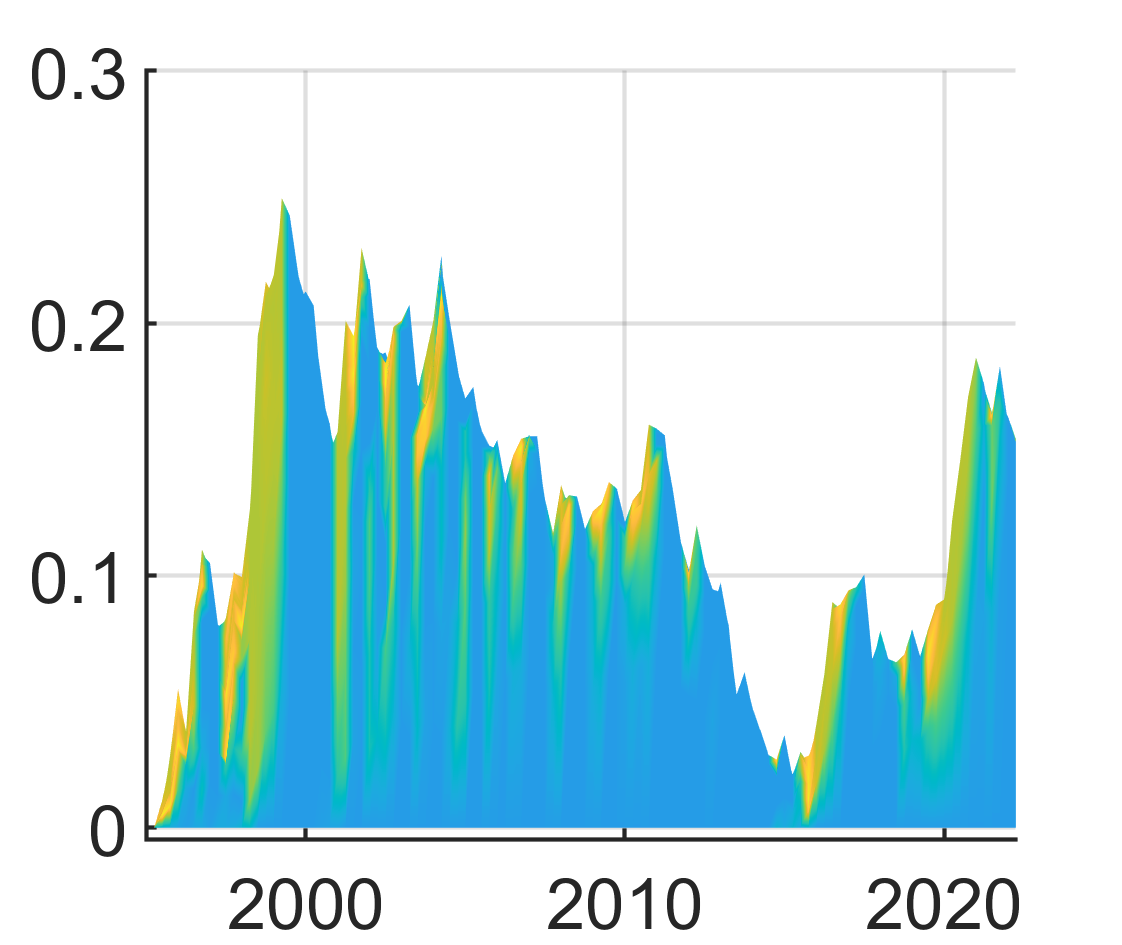

Supplement: Supplementary file 3 [file Data_Sheet_1.ZIP › CM_JPN_1 (4).tif]

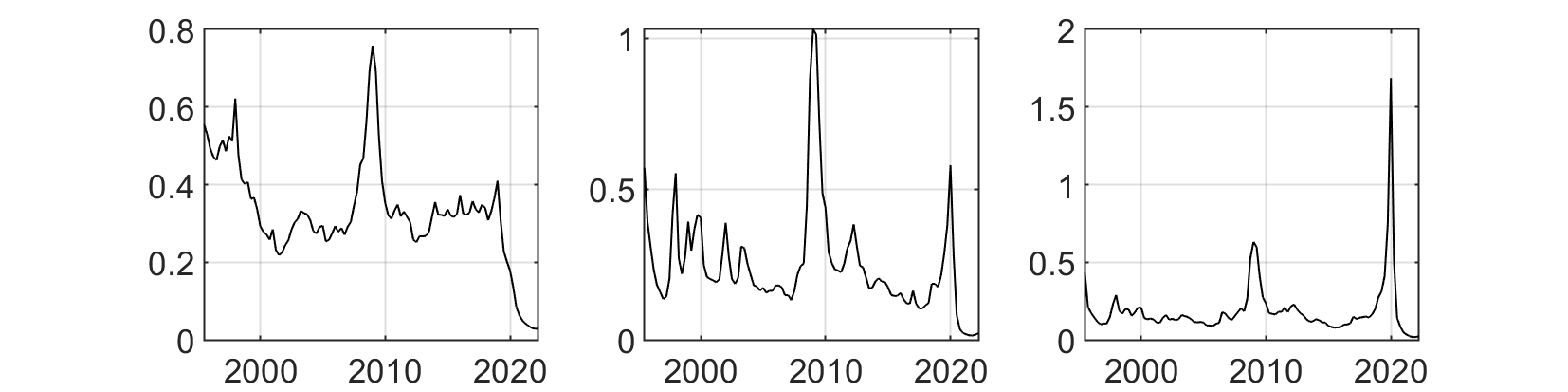

Supplement: Supplementary file 3 [file Data_Sheet_1.ZIP › CM_JPN_1 (5).tif]

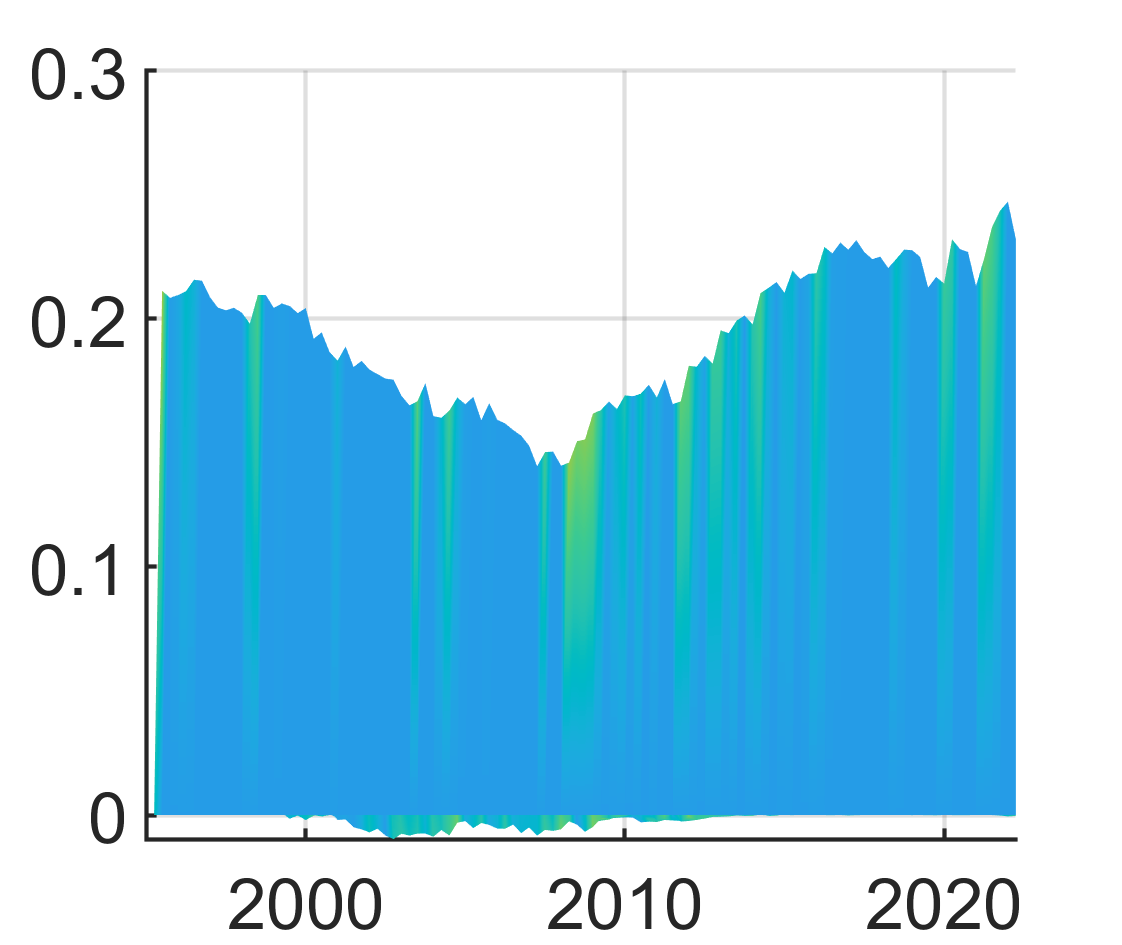

Supplement: Supplementary file 3 [file Data_Sheet_1.ZIP › CM_KR_1 (1).tif]

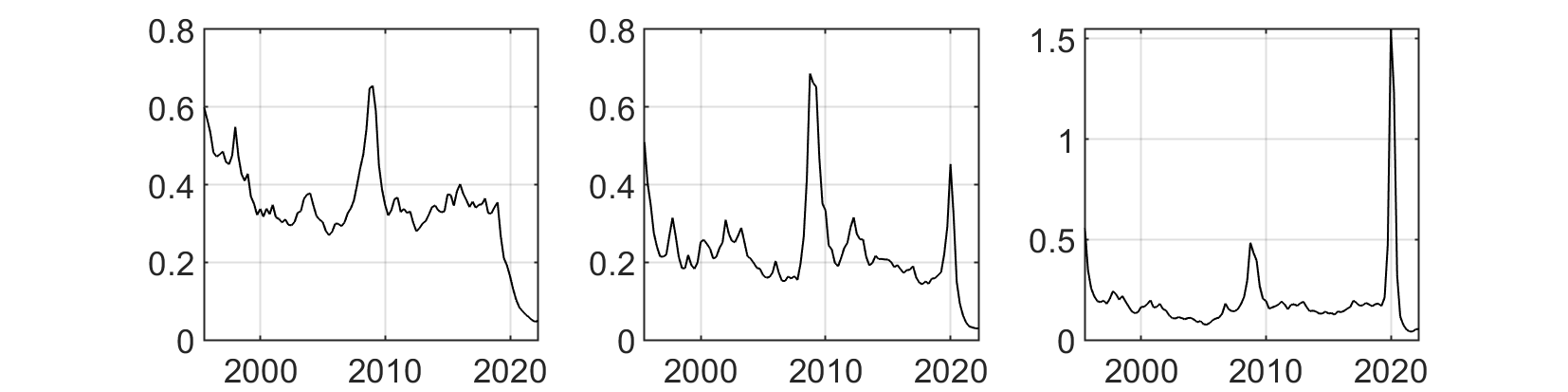

Supplement: Supplementary file 3 [file Data_Sheet_1.ZIP › CM_KR_1 (2).tif]

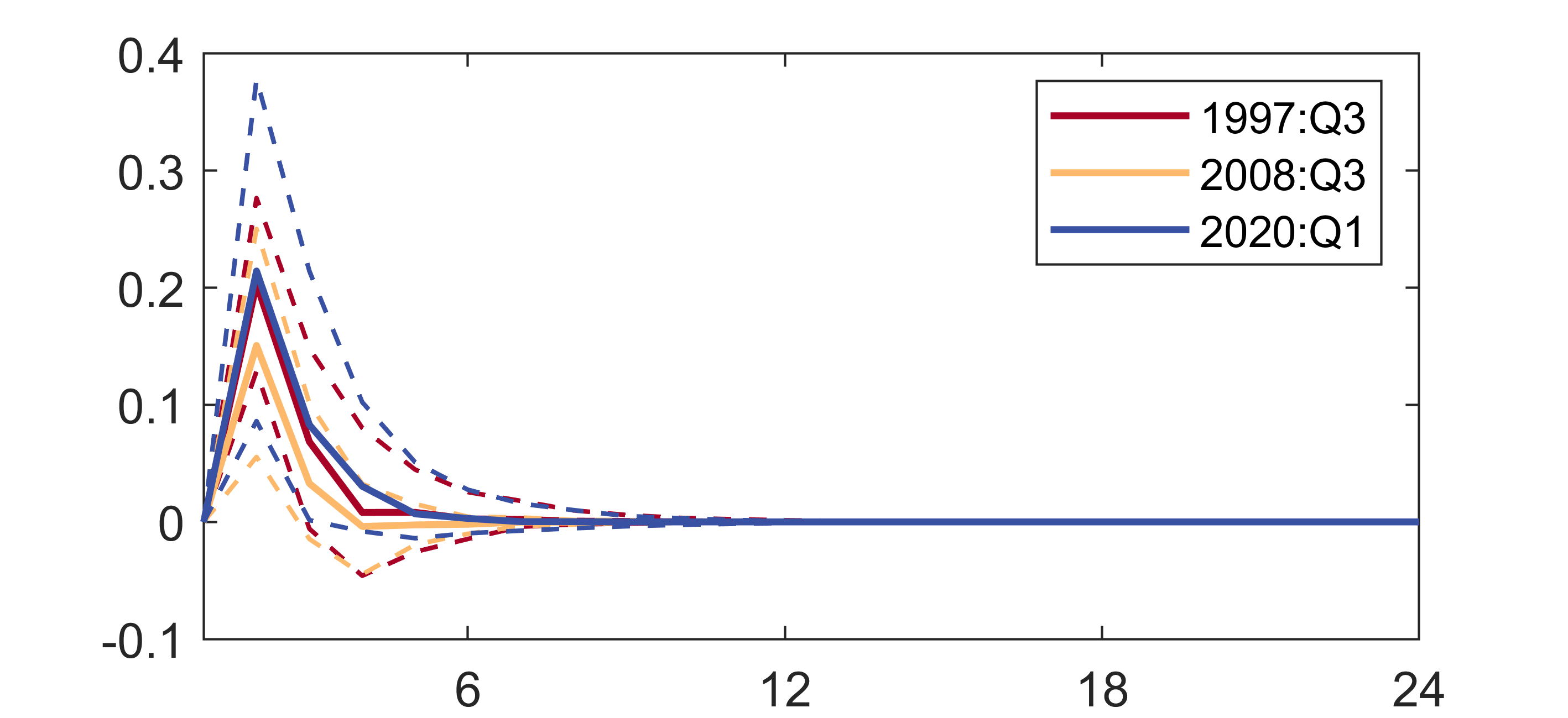

Supplement: Supplementary file 3 [file Data_Sheet_1.ZIP › CM_KR_1 (3).tif]

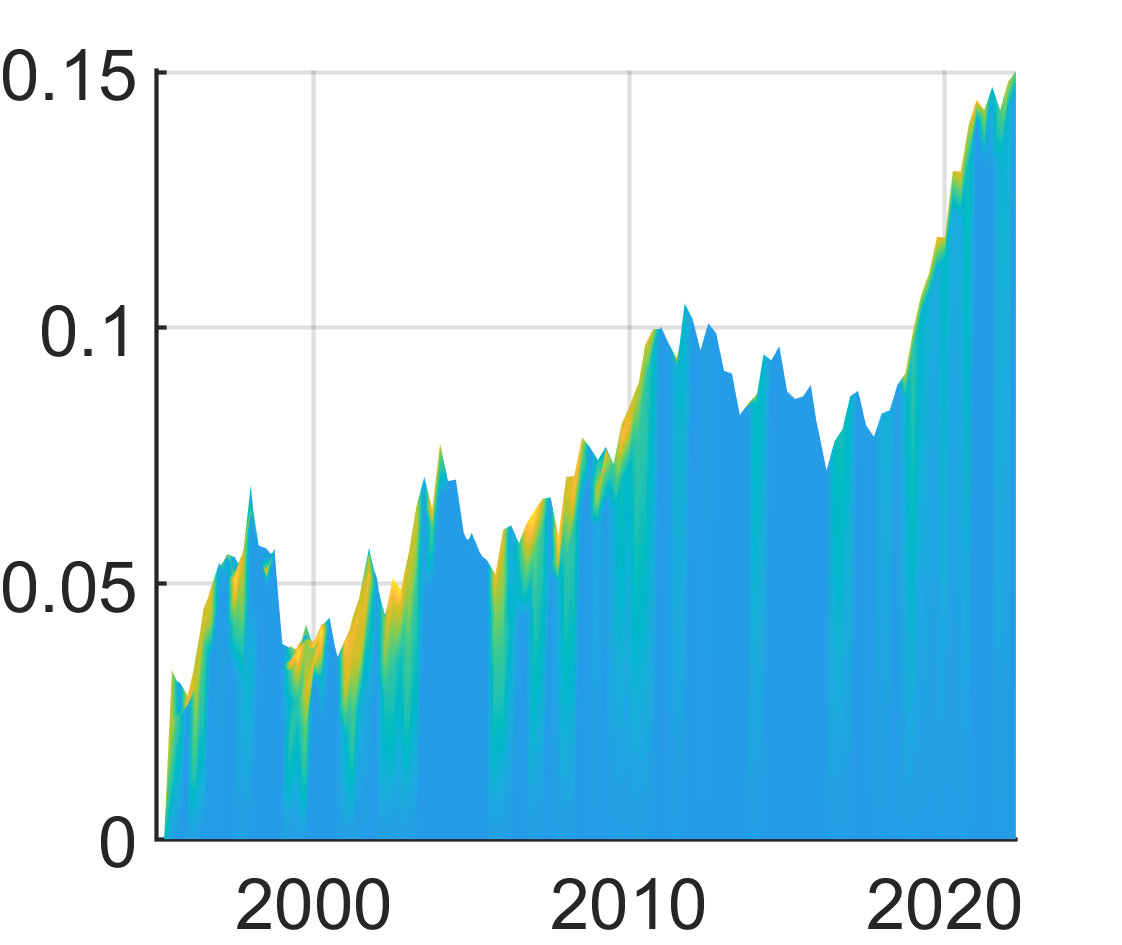

Supplement: Supplementary file 3 [file Data_Sheet_1.ZIP › COM_CHN_1 (1).tif]

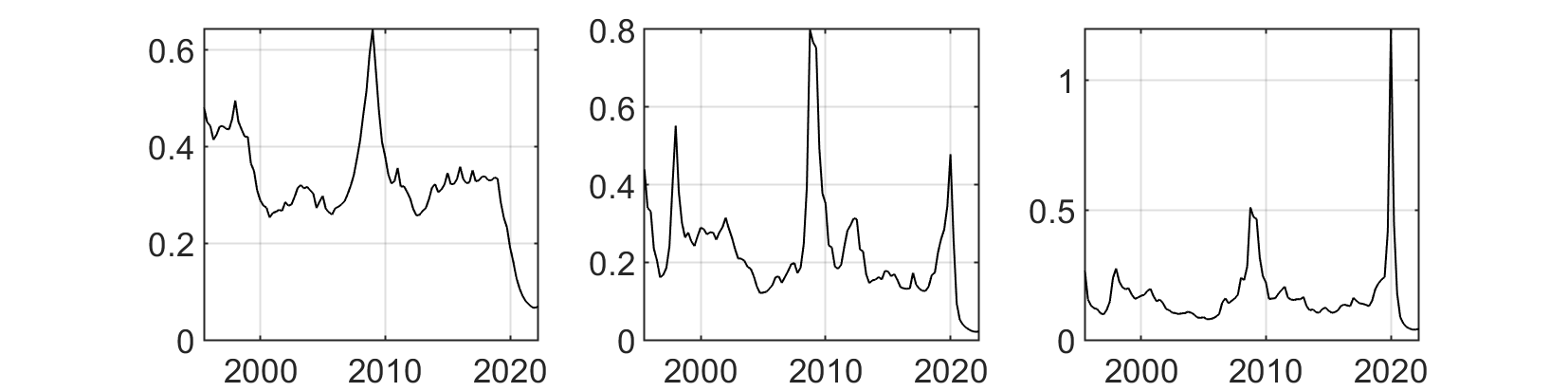

Supplement: Supplementary file 3 [file Data_Sheet_1.ZIP › COM_CHN_1 (2).tif]

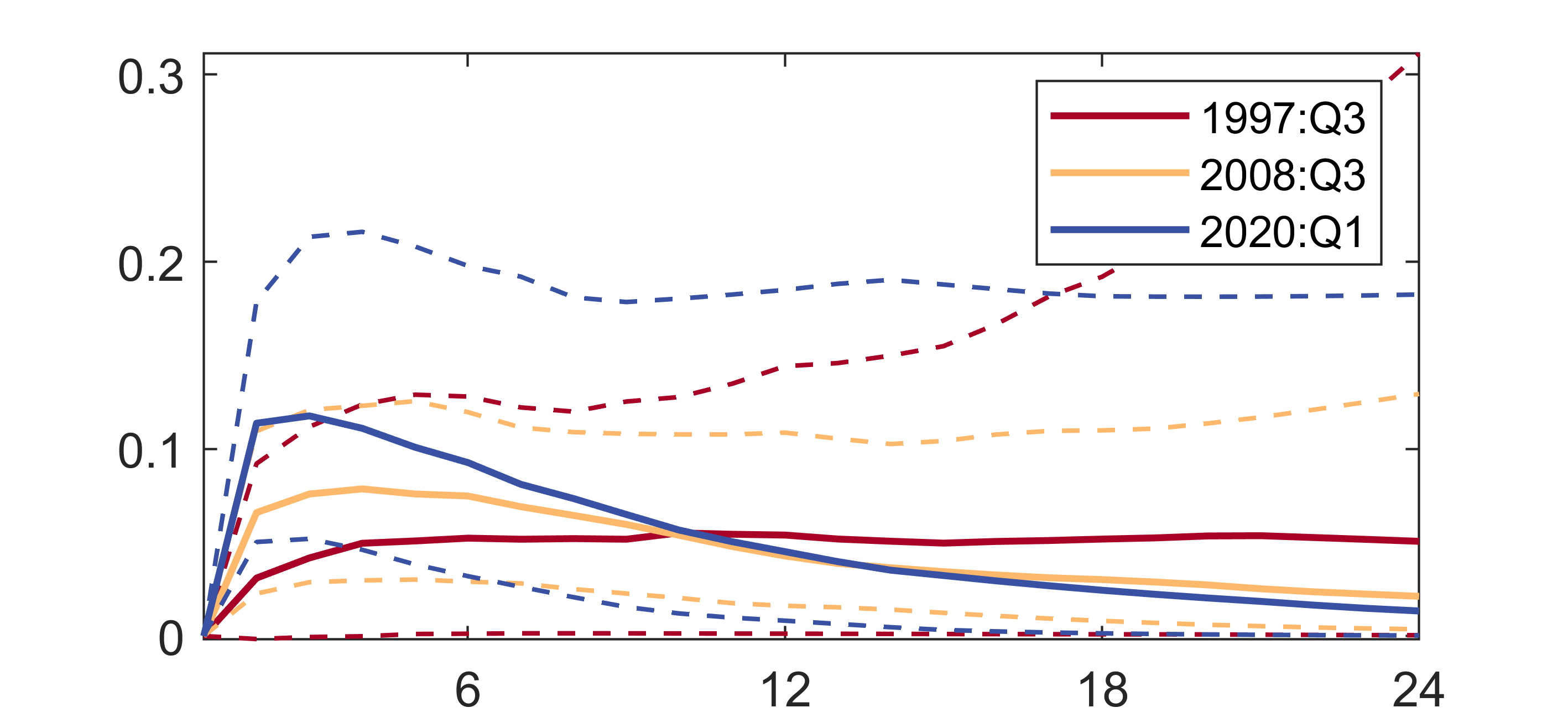

Supplement: Supplementary file 3 [file Data_Sheet_1.ZIP › COM_CHN_1 (3).tif]

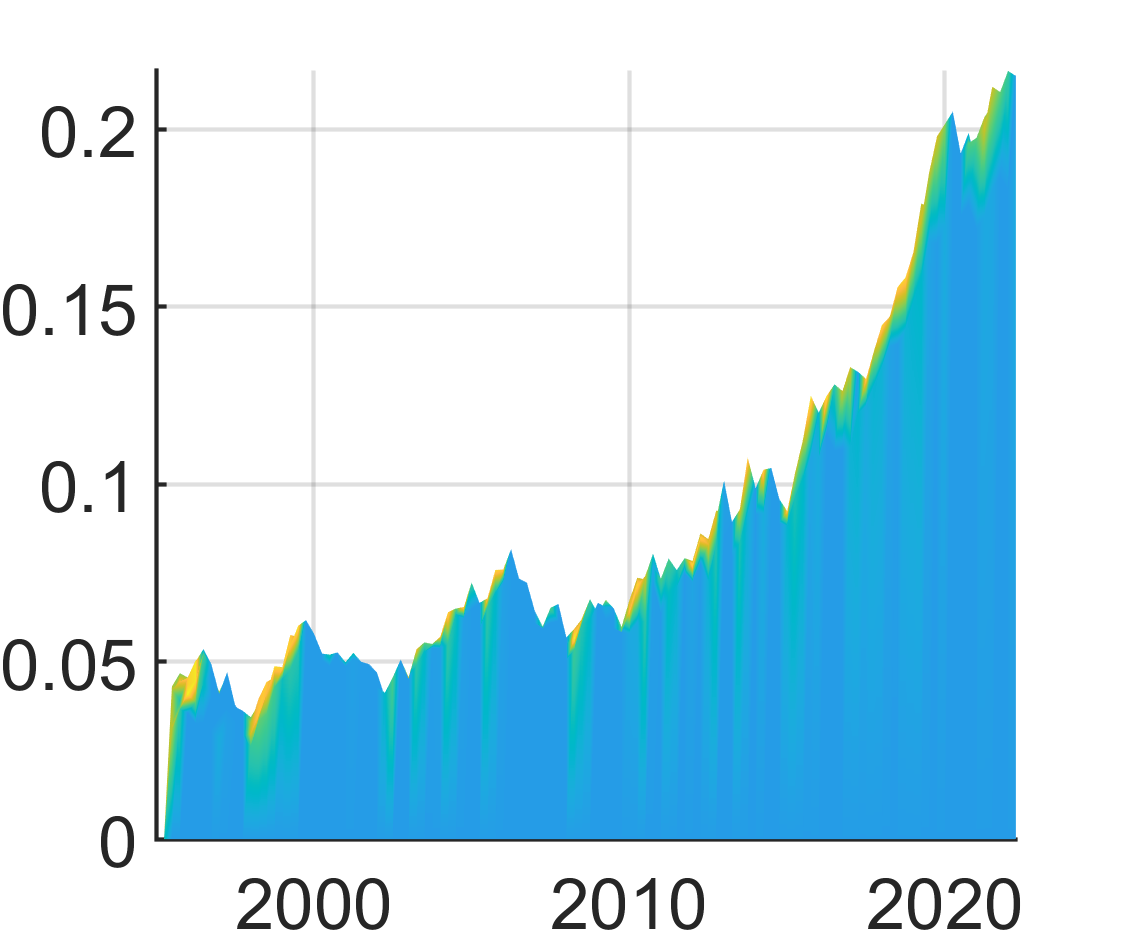

Supplement: Supplementary file 3 [file Data_Sheet_1.ZIP › COM_HK_1 (1).tif]

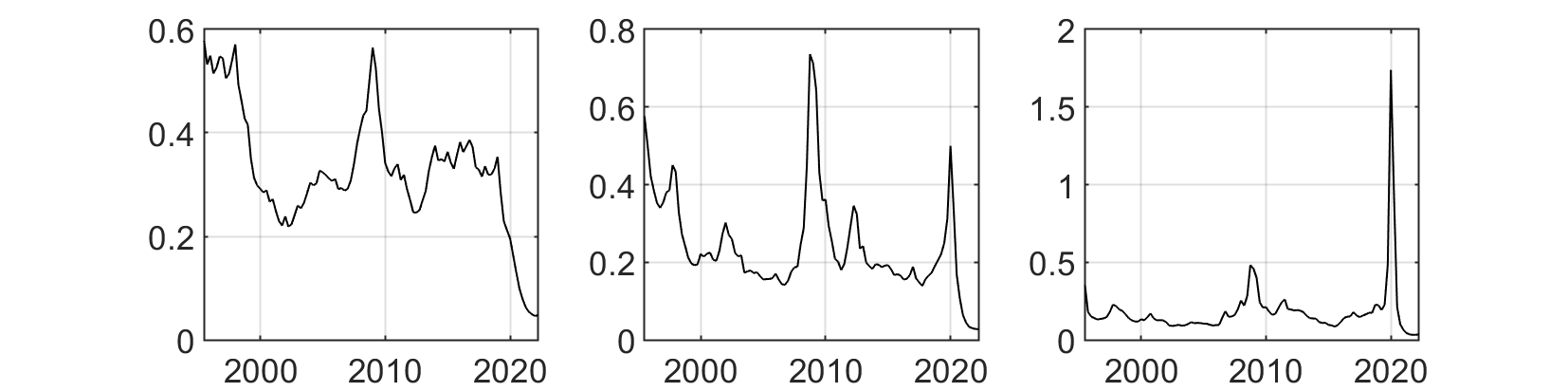

Supplement: Supplementary file 3 [file Data_Sheet_1.ZIP › COM_HK_1 (2).tif]

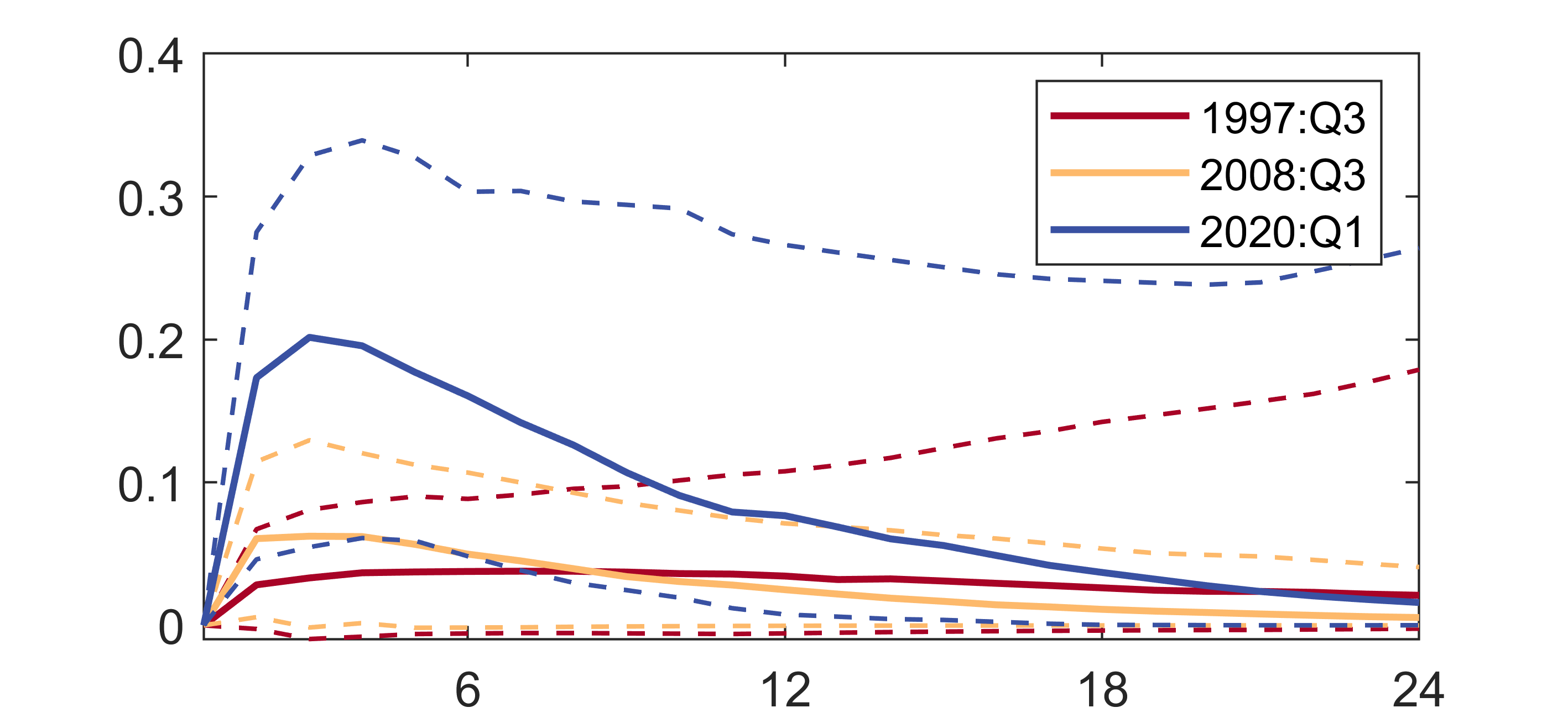

Supplement: Supplementary file 3 [file Data_Sheet_1.ZIP › COM_HK_1 (3).tif]

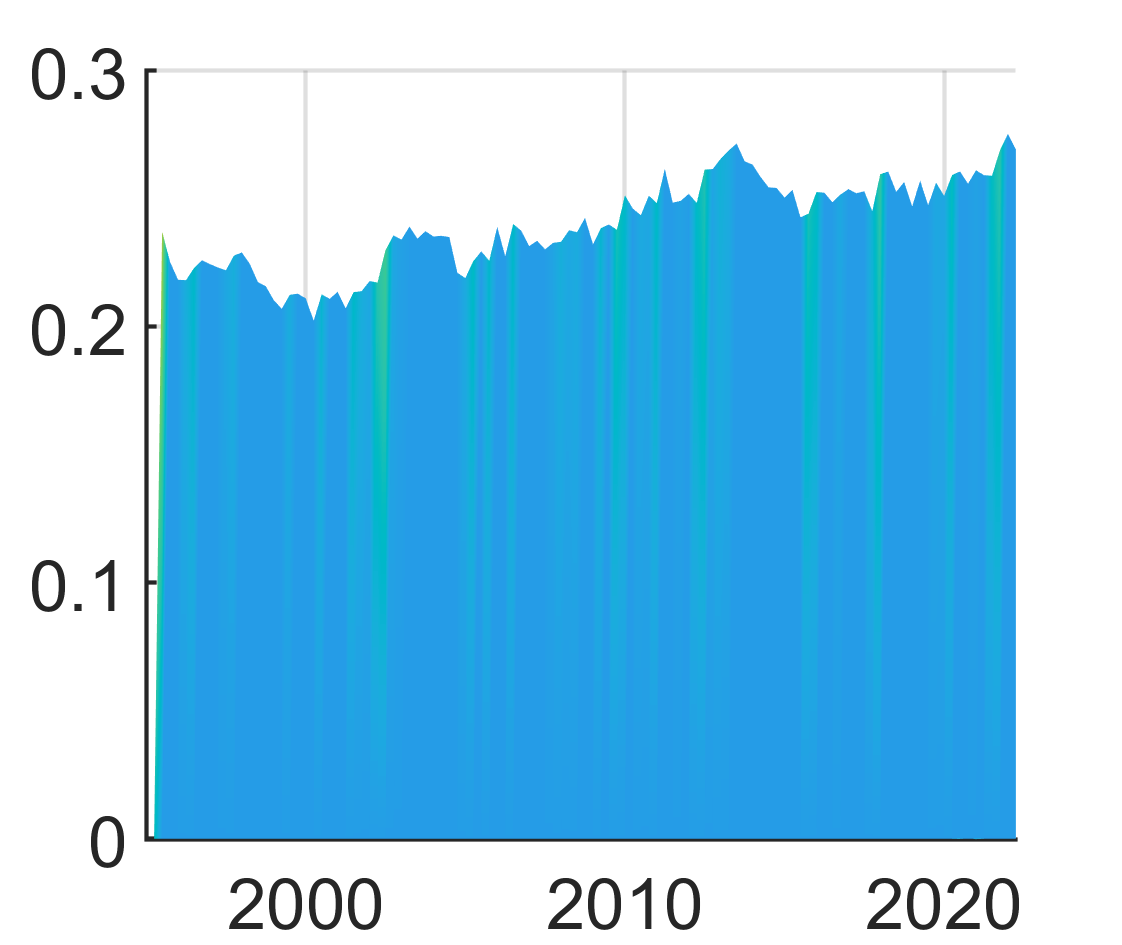

Supplement: Supplementary file 3 [file Data_Sheet_1.ZIP › COM_JPN_1 (1).tif]

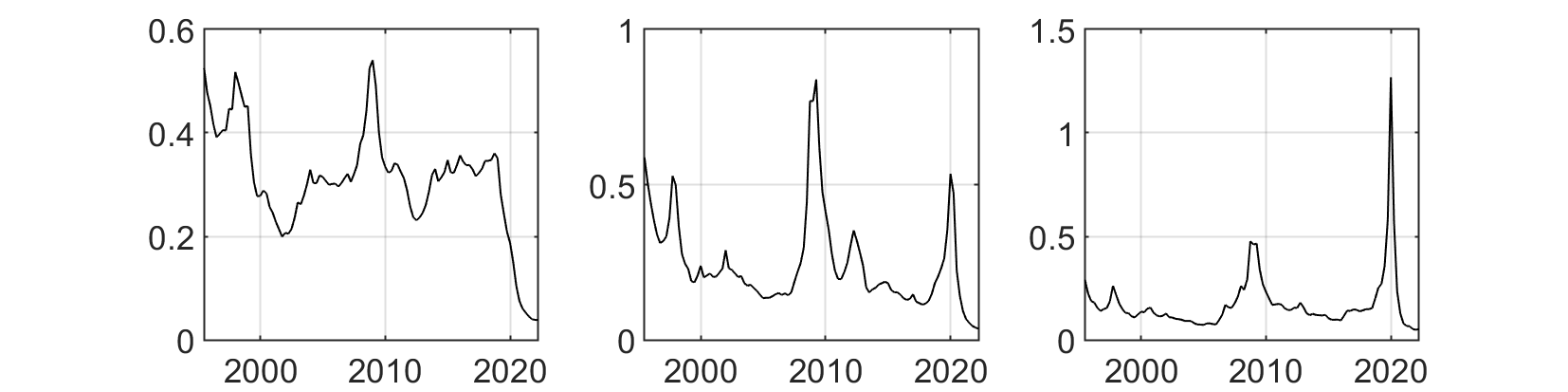

Supplement: Supplementary file 3 [file Data_Sheet_1.ZIP › COM_JPN_1 (2).tif]

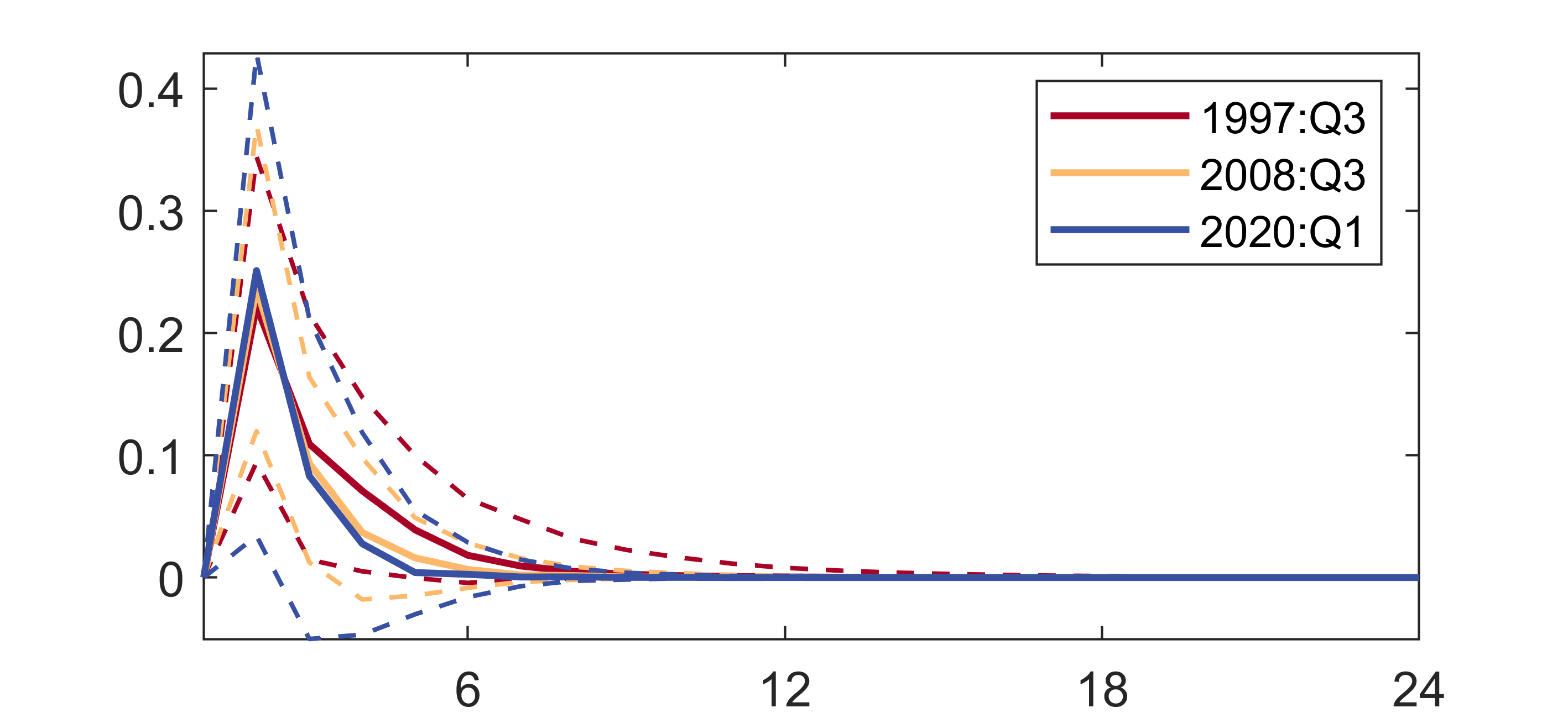

Supplement: Supplementary file 3 [file Data_Sheet_1.ZIP › COM_JPN_1 (3).tif]

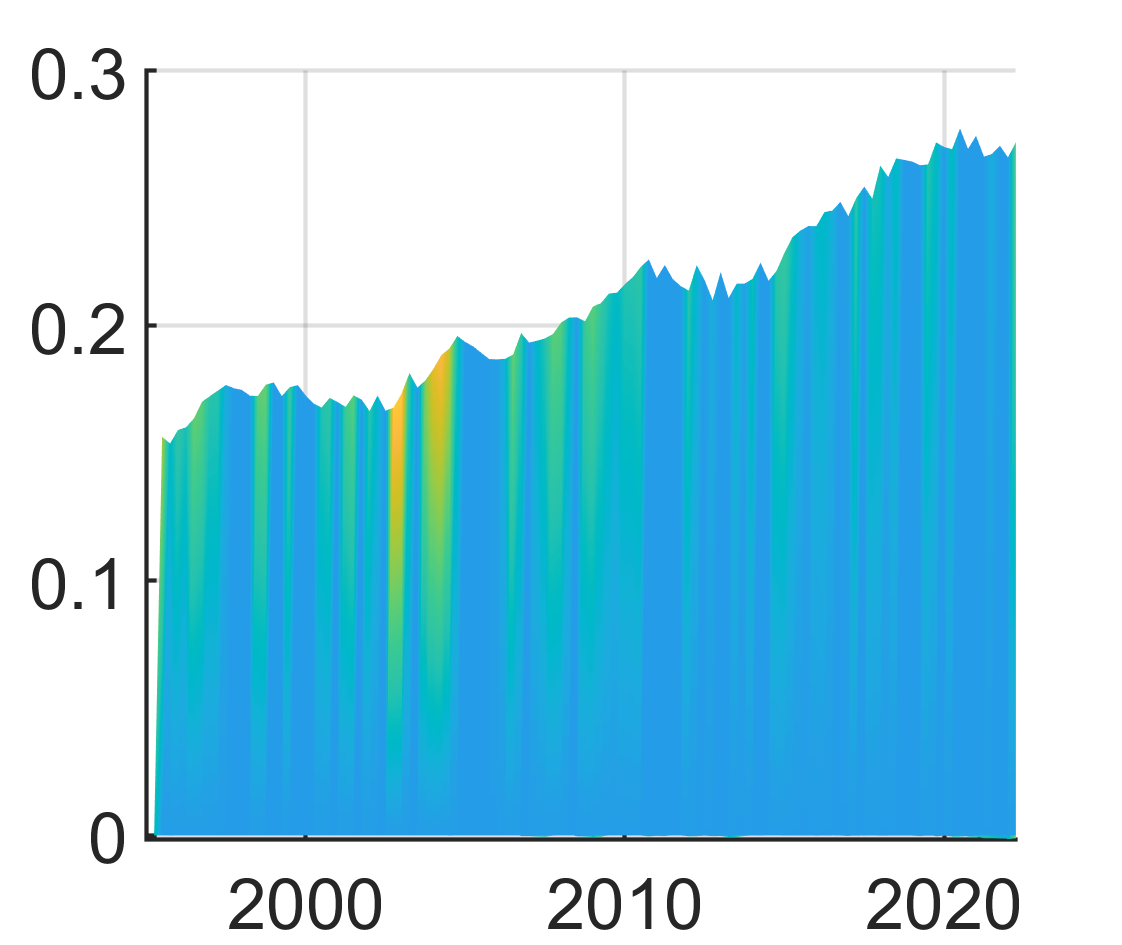

Supplement: Supplementary file 3 [file Data_Sheet_1.ZIP › COM_KR_1 (1).tif]

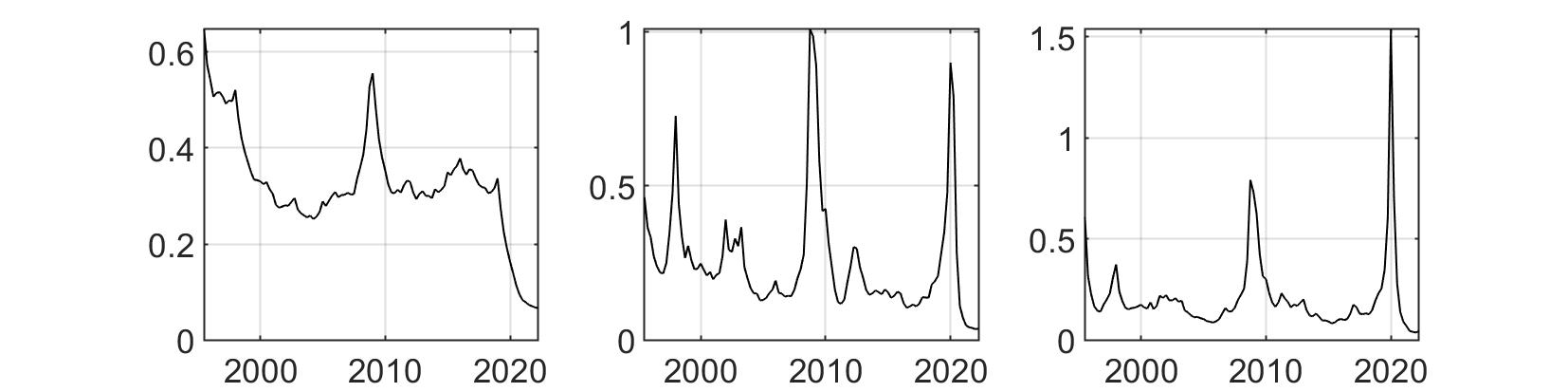

Supplement: Supplementary file 3 [file Data_Sheet_1.ZIP › COM_KR_1 (2).tif]

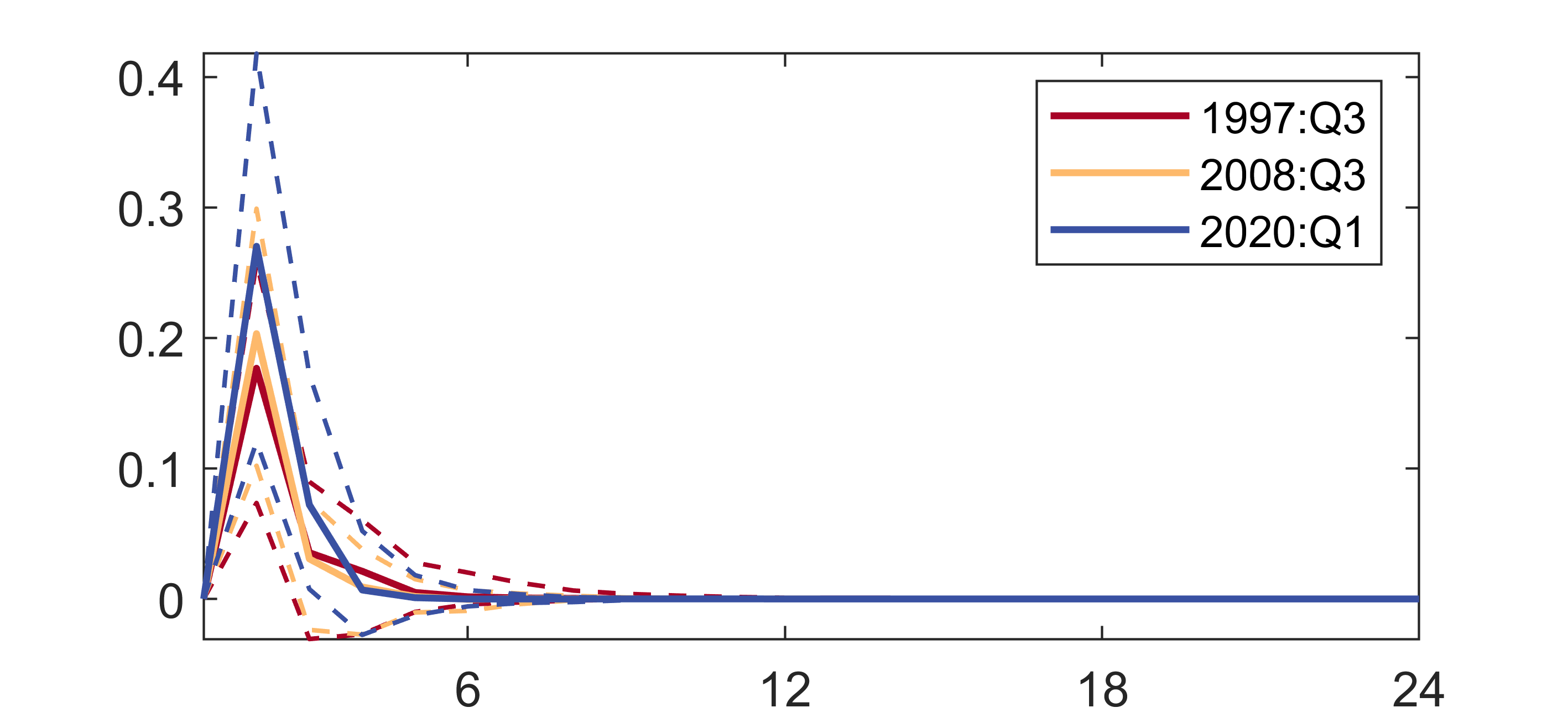

Supplement: Supplementary file 3 [file Data_Sheet_1.ZIP › COM_KR_1 (3).tif]

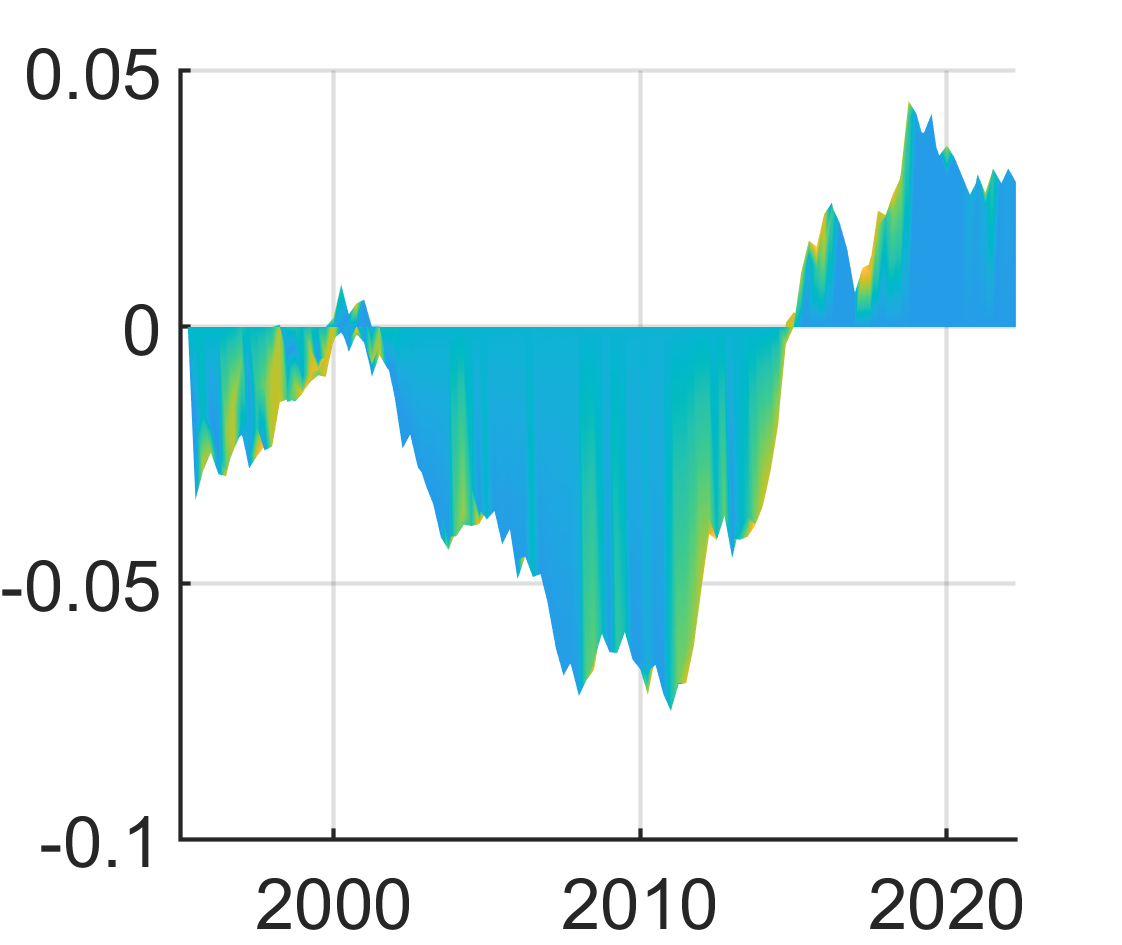

Supplement: Supplementary file 3 [file Data_Sheet_1.ZIP › FEM_CHN_1 (1).tif]

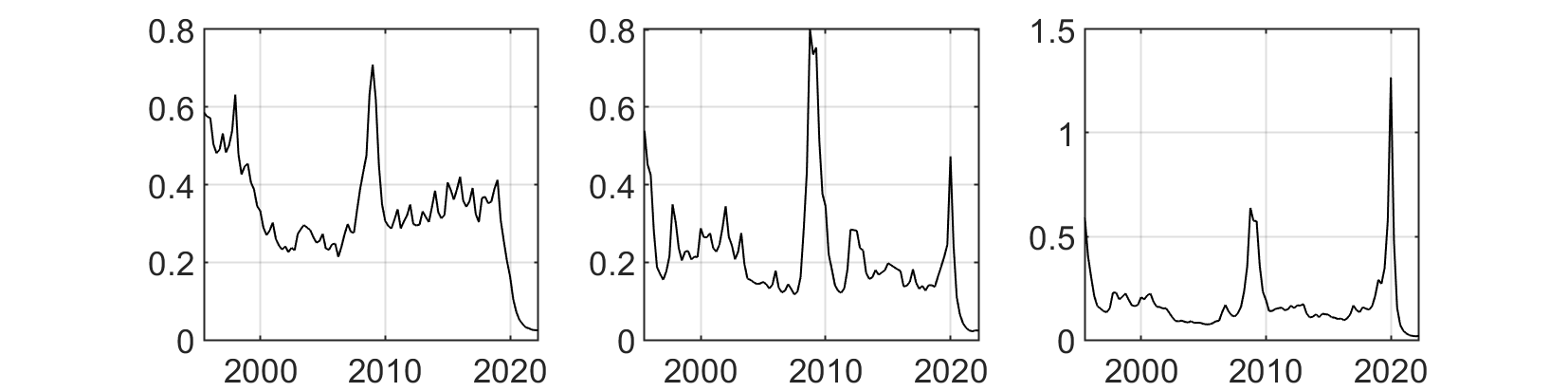

Supplement: Supplementary file 3 [file Data_Sheet_1.ZIP › FEM_CHN_1 (2).tif]

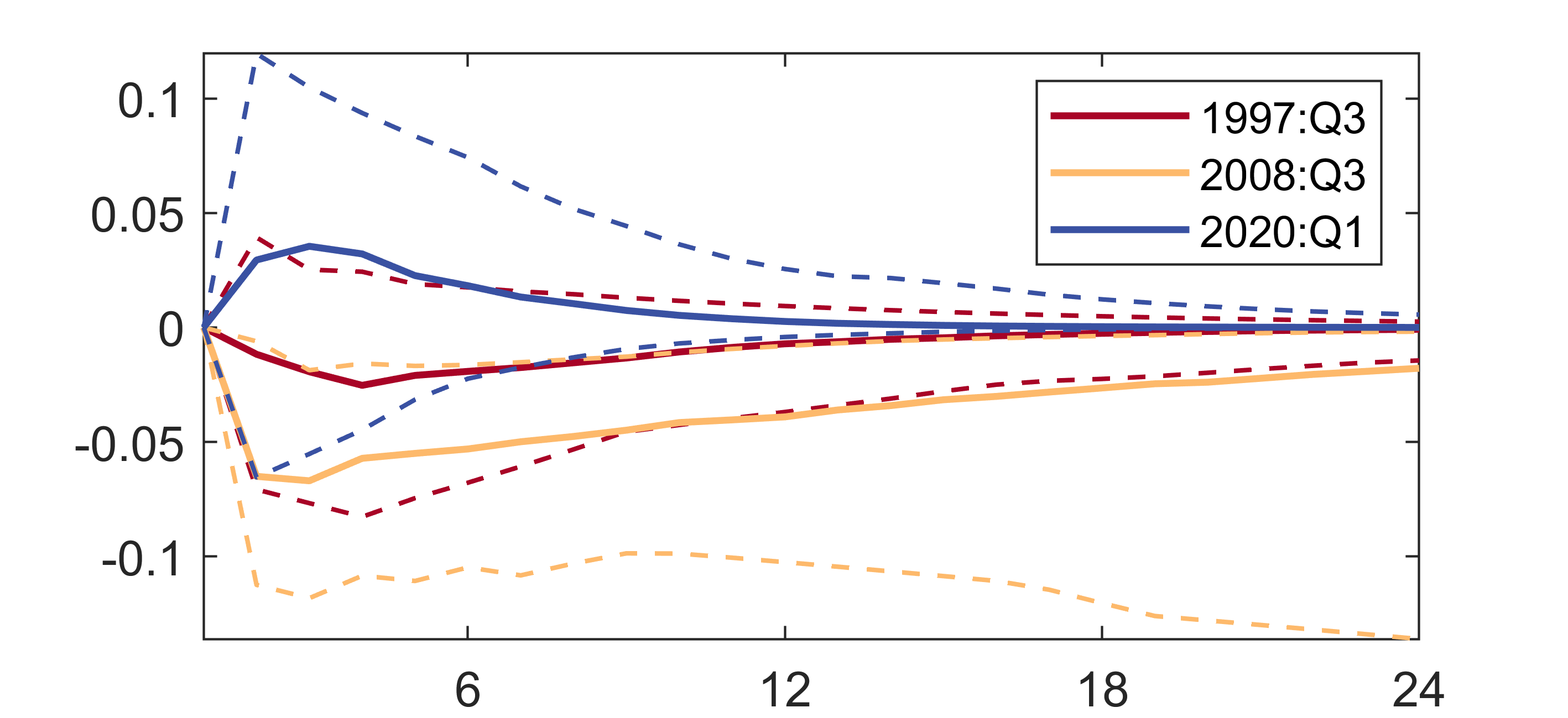

Supplement: Supplementary file 3 [file Data_Sheet_1.ZIP › FEM_CHN_1 (3).tif]

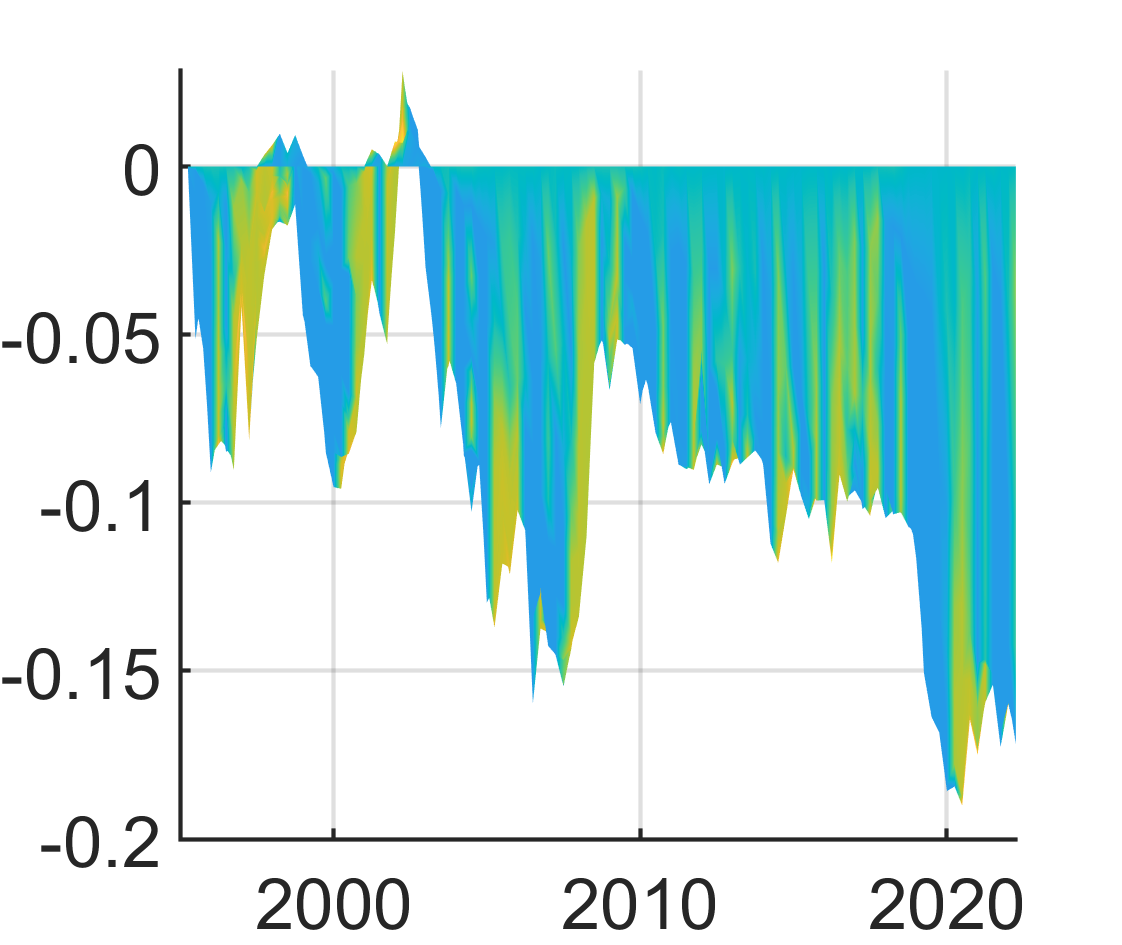

Supplement: Supplementary file 3 [file Data_Sheet_1.ZIP › FEM_HK_1 (1).tif]

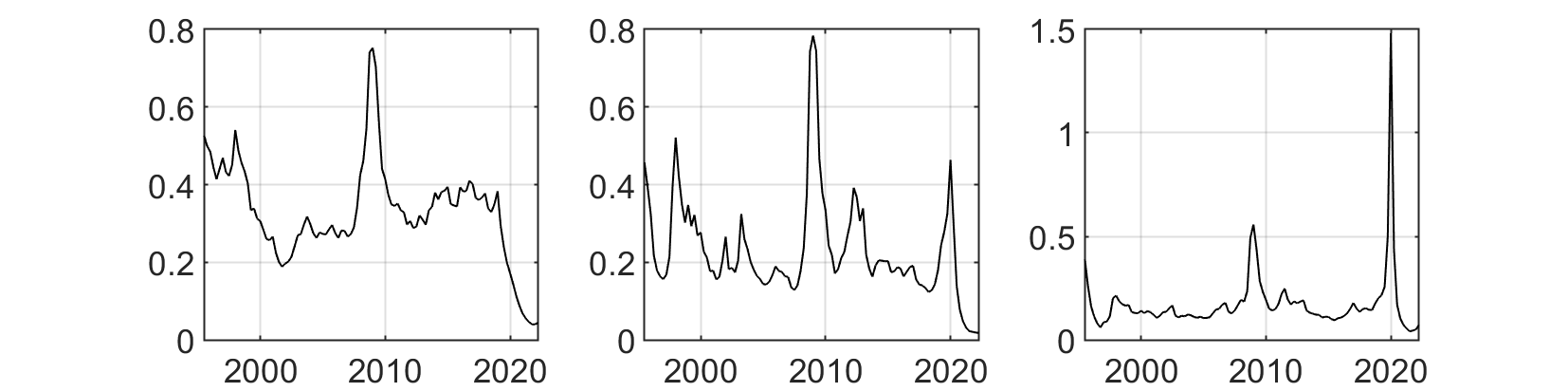

Supplement: Supplementary file 3 [file Data_Sheet_1.ZIP › FEM_HK_1 (2).tif]

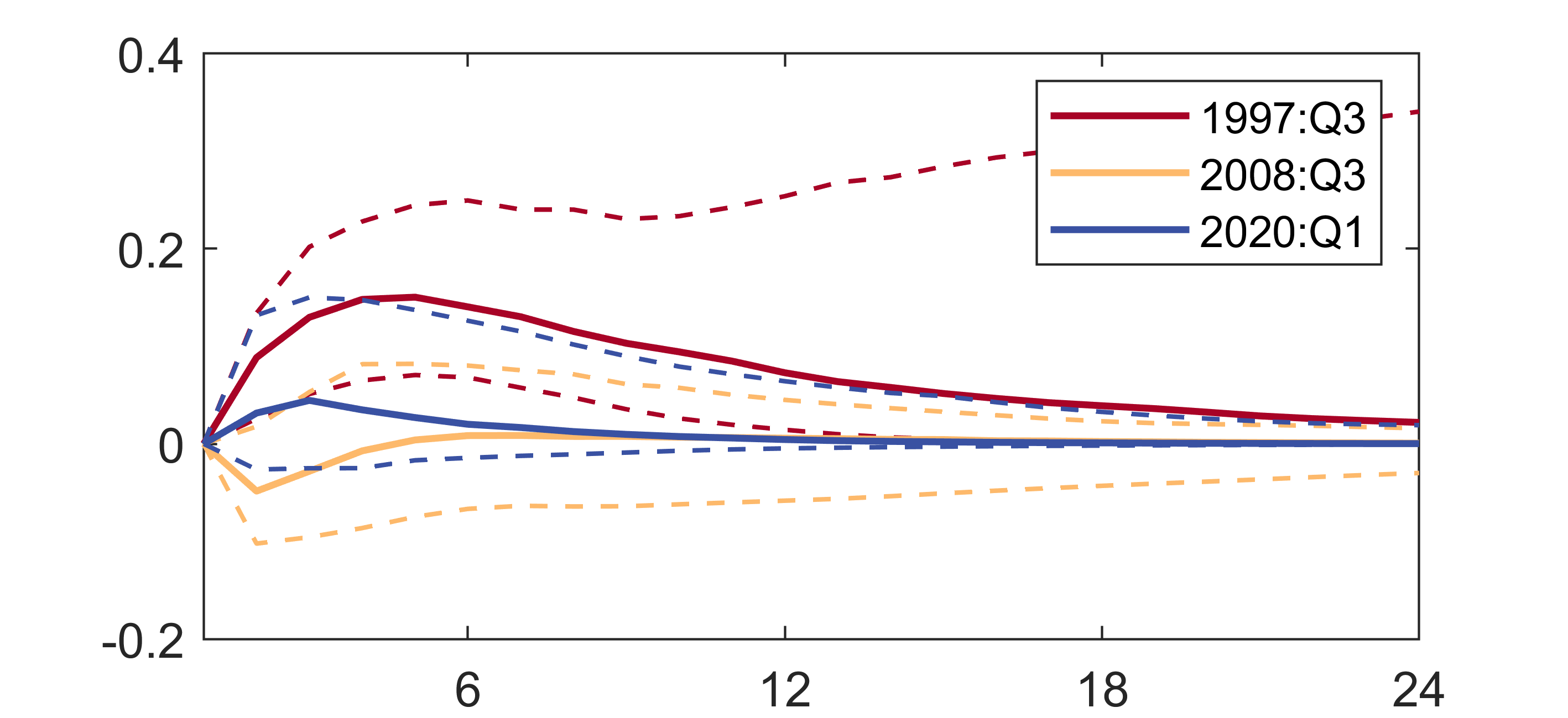

Supplement: Supplementary file 3 [file Data_Sheet_1.ZIP › FEM_JPN_1 (3).tif]

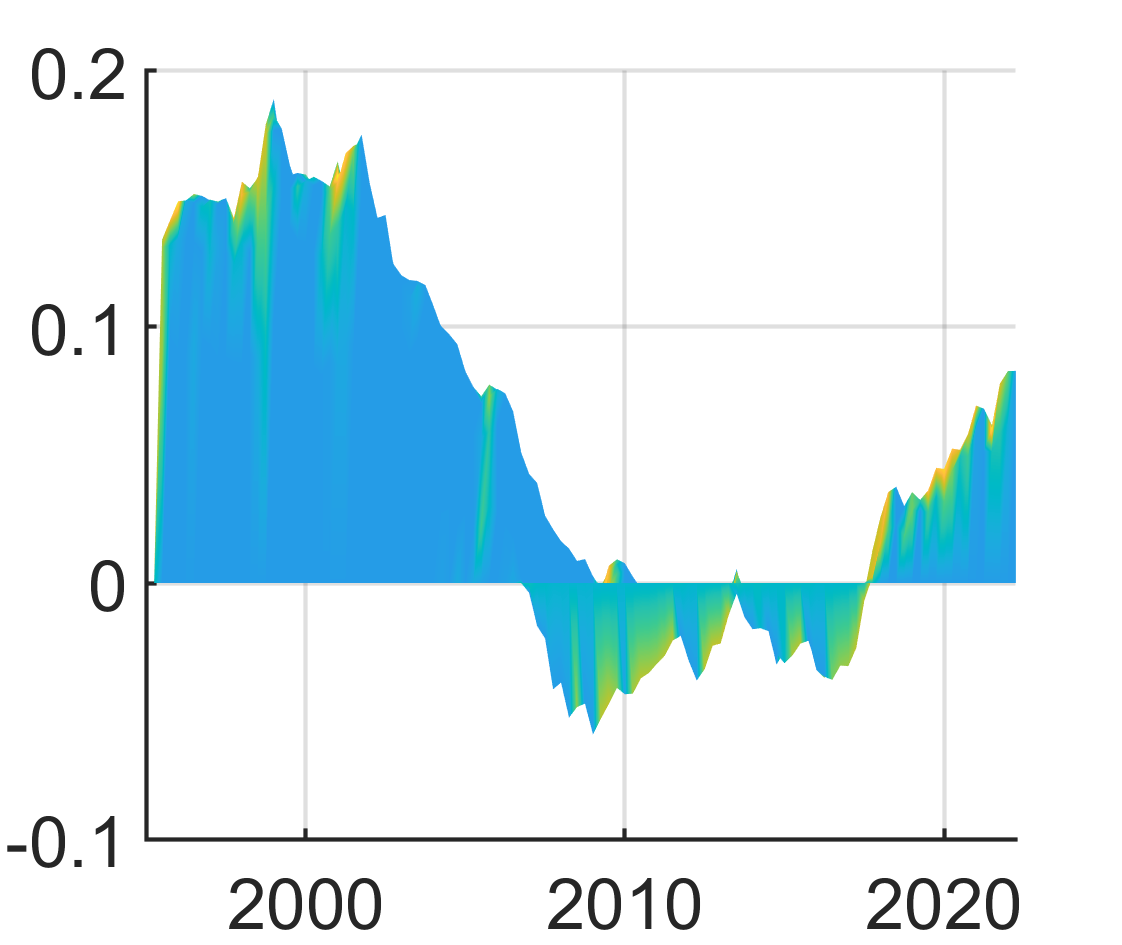

Supplement: Supplementary file 3 [file Data_Sheet_1.ZIP › FEM_JPN_1 (4).tif]

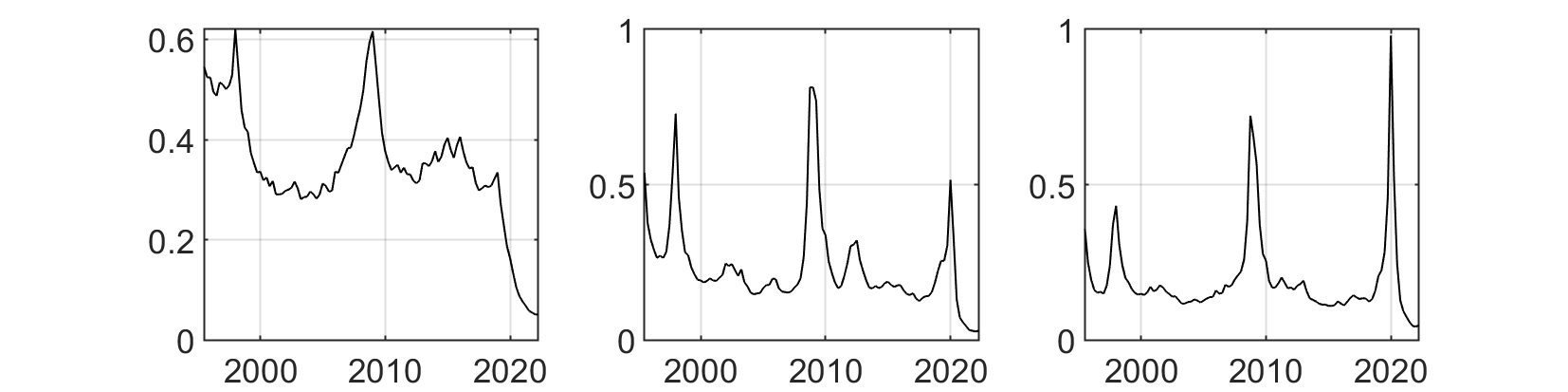

Supplement: Supplementary file 3 [file Data_Sheet_1.ZIP › FEM_JPN_1 (5).tif]

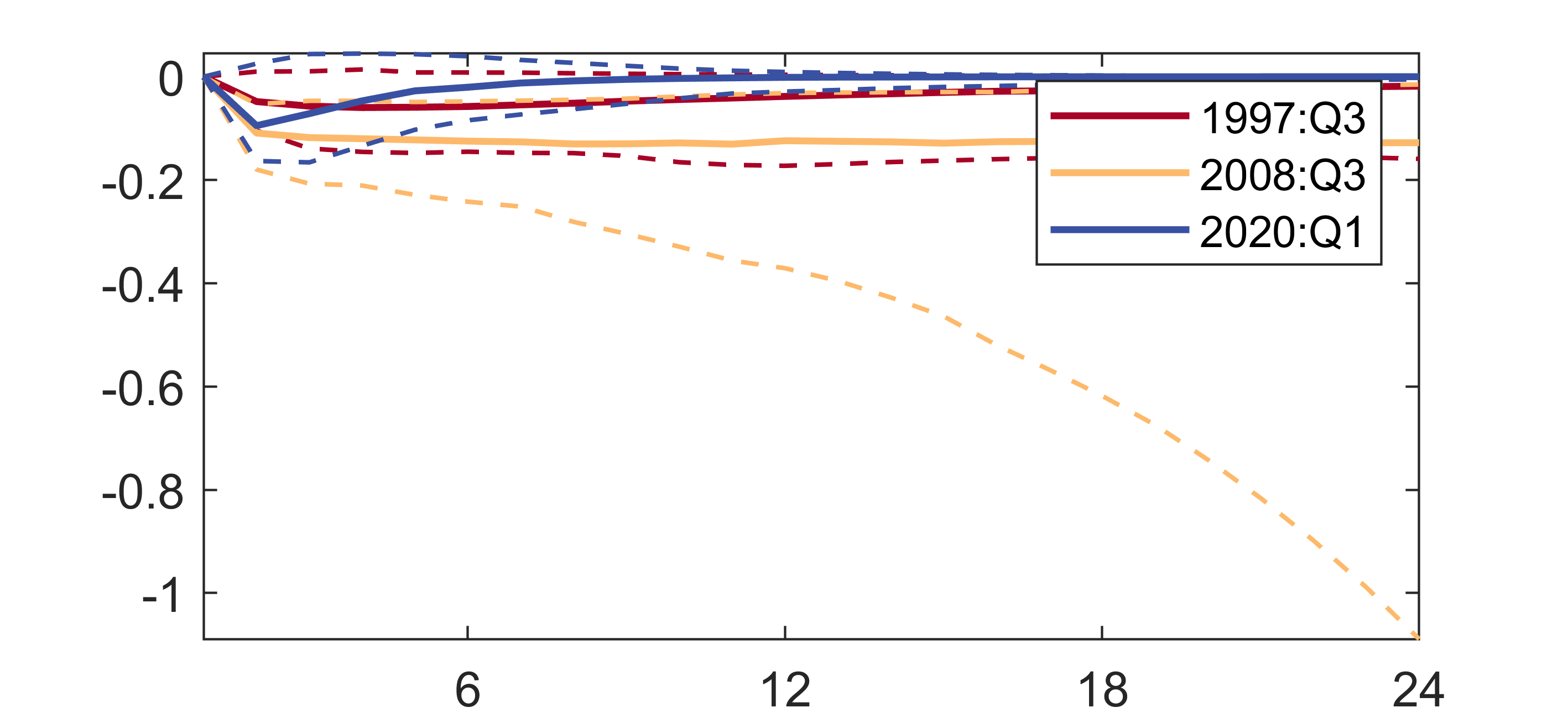

Supplement: Supplementary file 3 [file Data_Sheet_1.ZIP › FEM_KR_1 (3).tif]

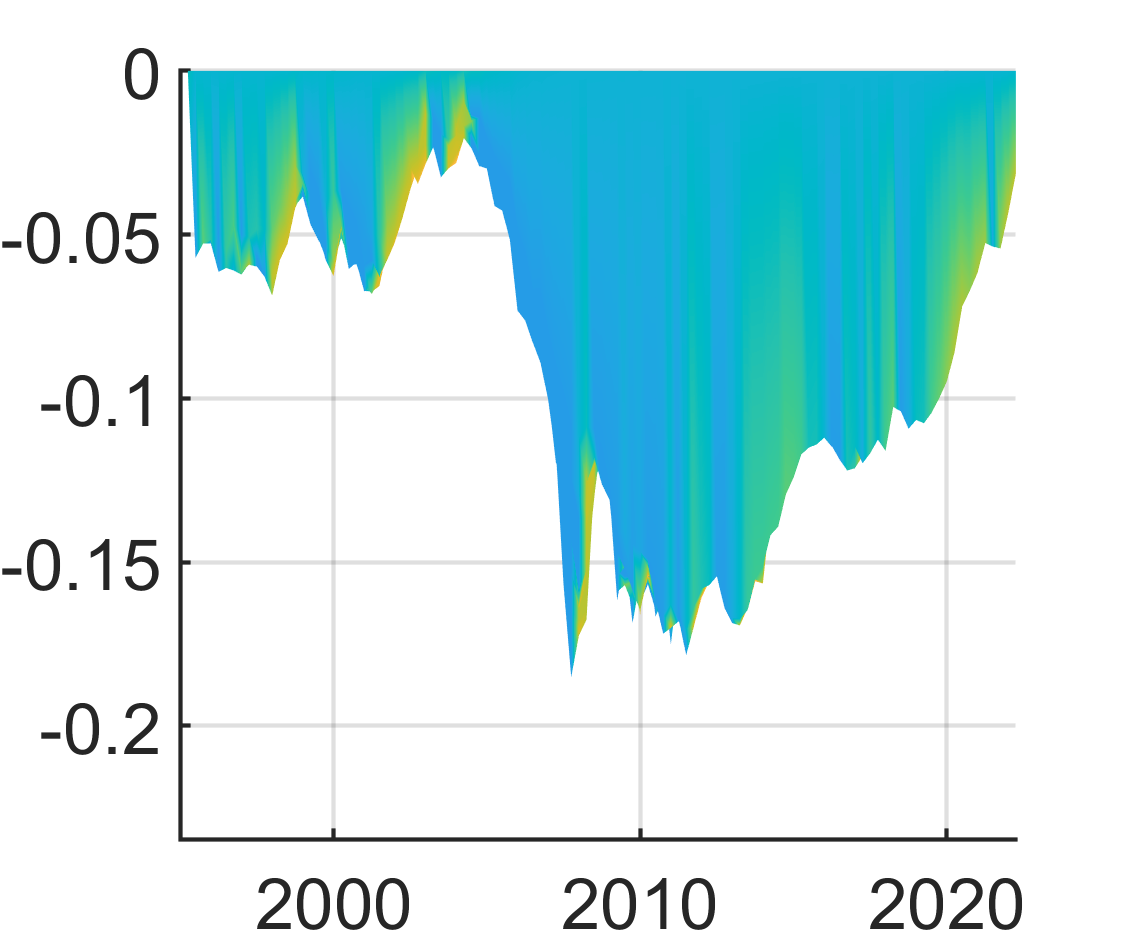

Supplement: Supplementary file 3 [file Data_Sheet_1.ZIP › FEM_KR_1 (4).tif]

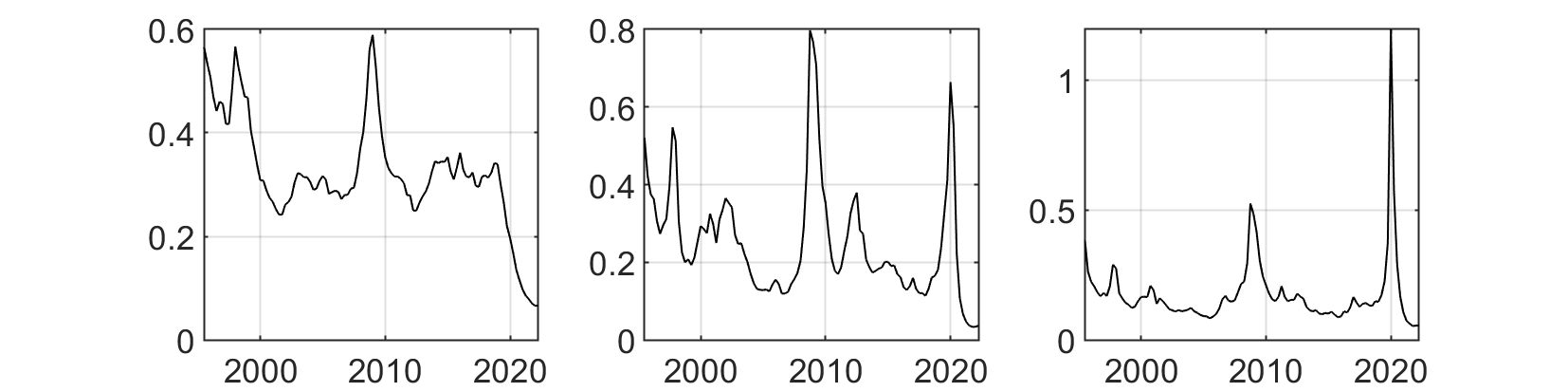

Supplement: Supplementary file 3 [file Data_Sheet_1.ZIP › FEM_KR_1 (5).tif]

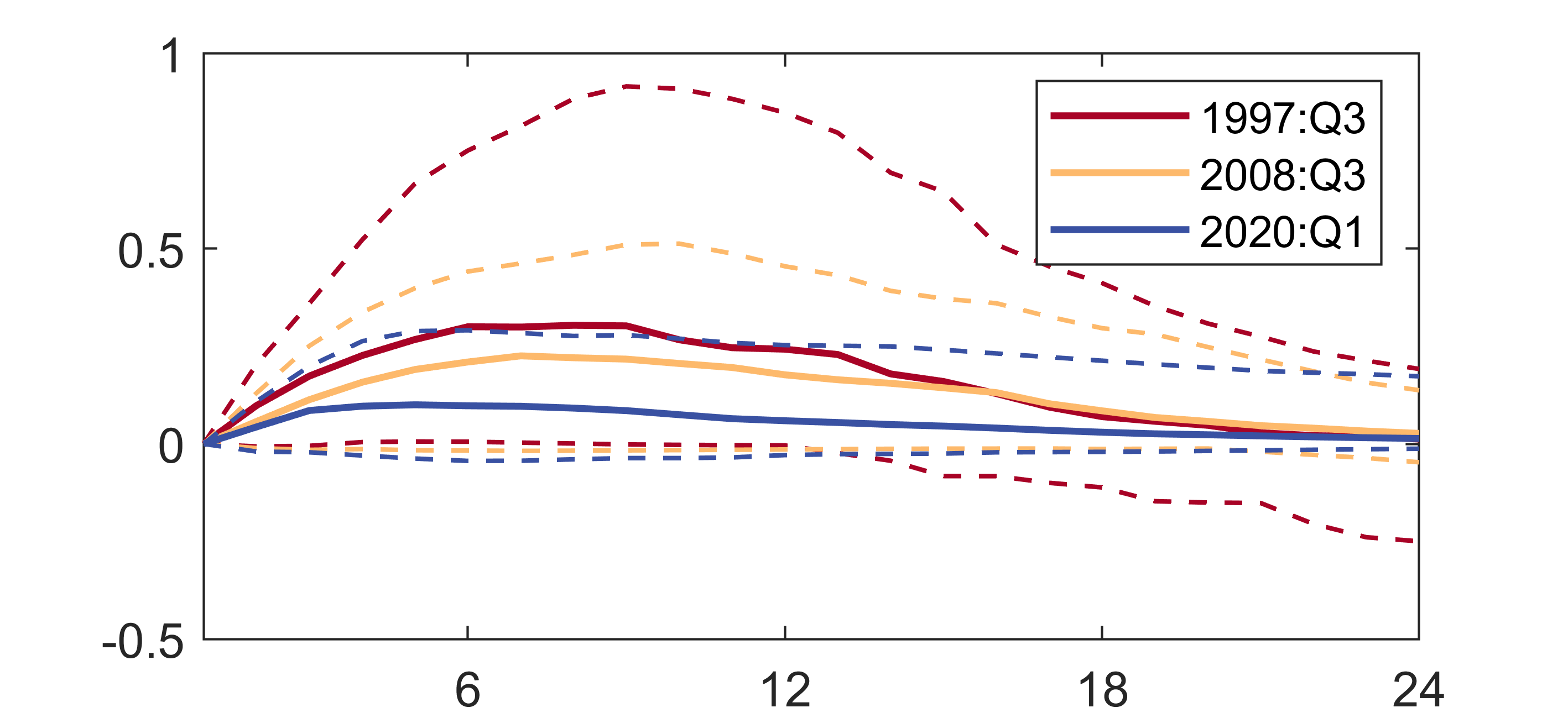

Supplement: Supplementary file 3 [file Data_Sheet_1.ZIP › HK_CHN_1 (1).tif]

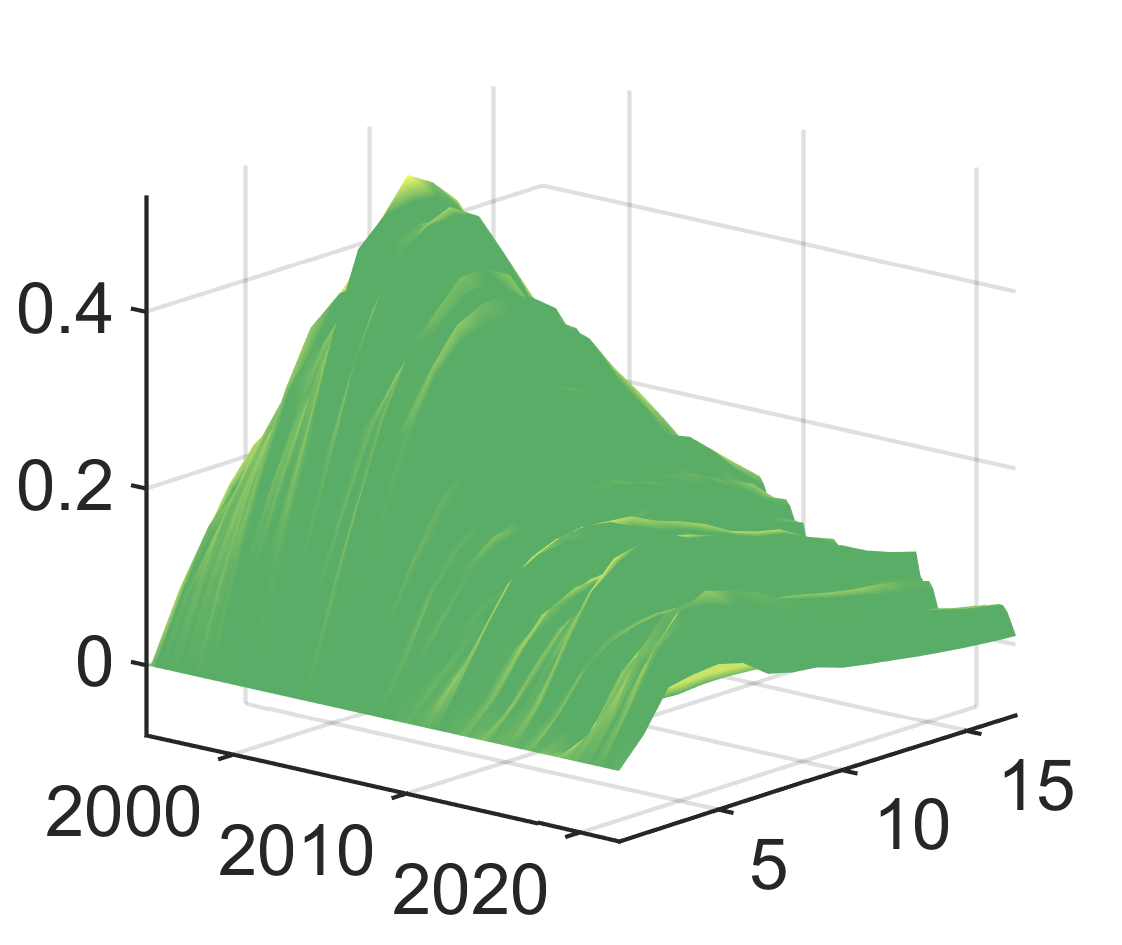

Supplement: Supplementary file 3 [file Data_Sheet_1.ZIP › HK_CHN_1 (2).tif]

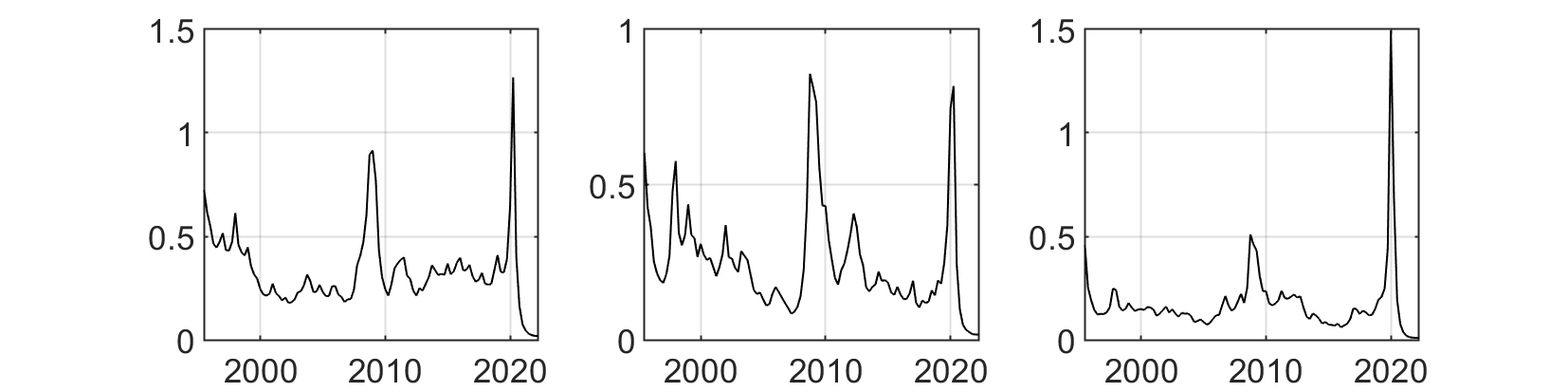

Supplement: Supplementary file 3 [file Data_Sheet_1.ZIP › HK_CHN_1 (3).tif]

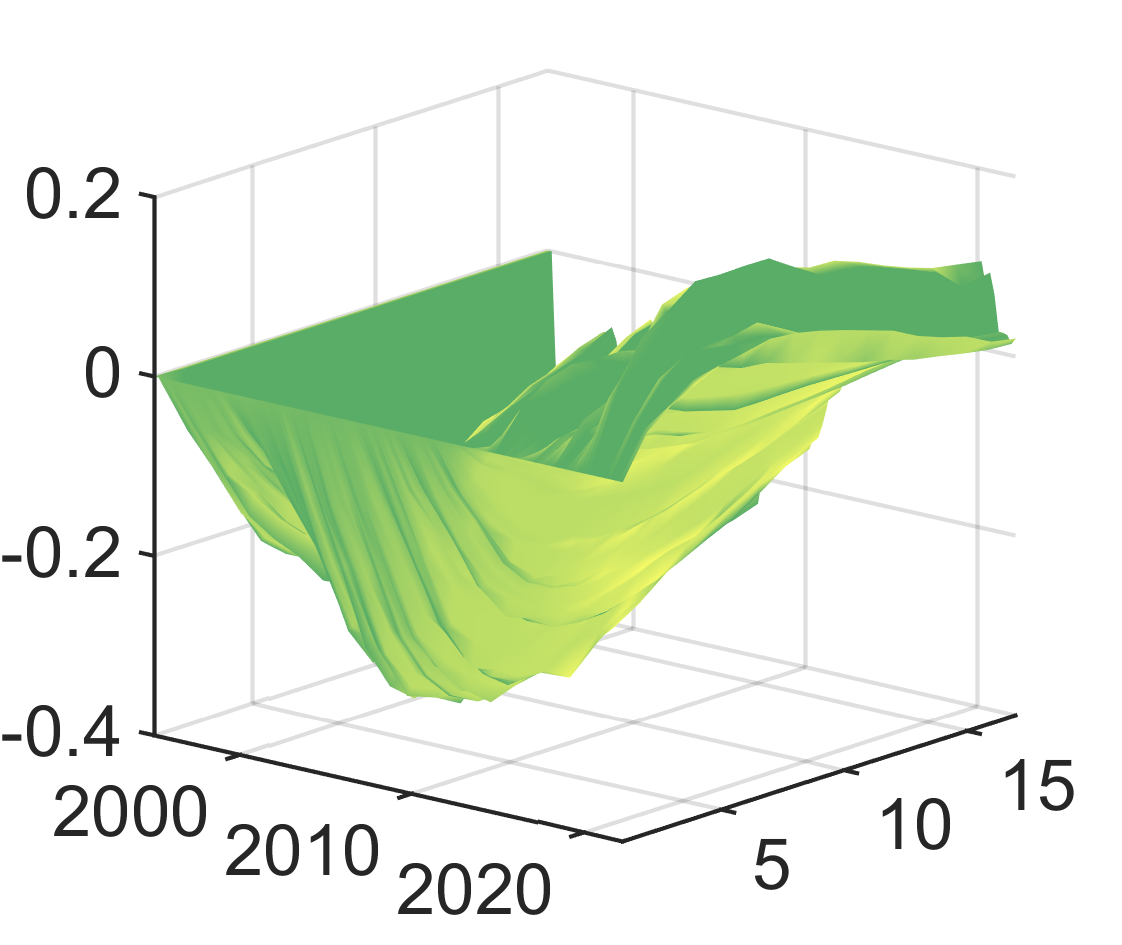

Supplement: Supplementary file 3 [file Data_Sheet_1.ZIP › HK_JPN_1 (1).tif]

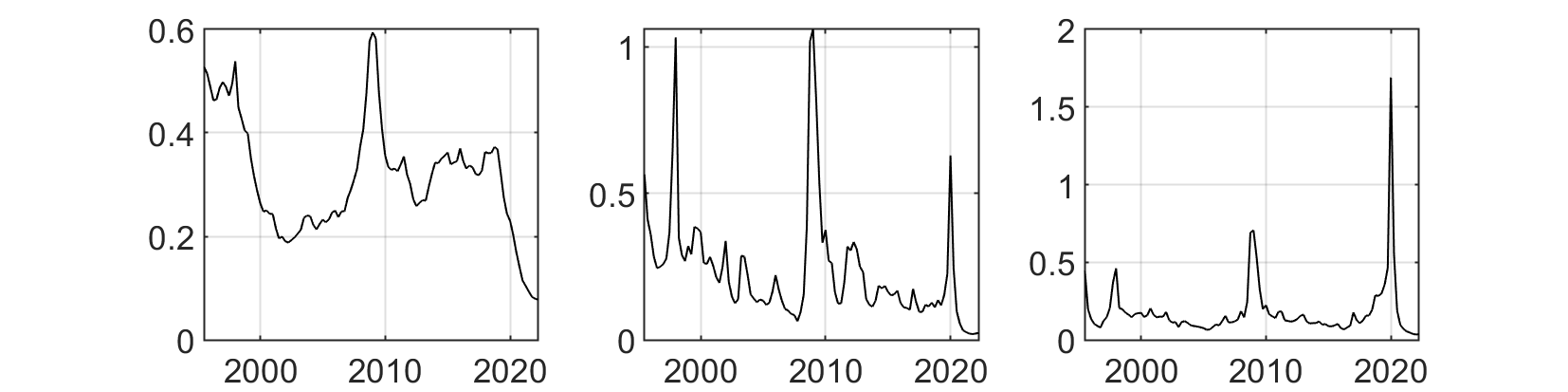

Supplement: Supplementary file 3 [file Data_Sheet_1.ZIP › HK_JPN_1 (2).tif]

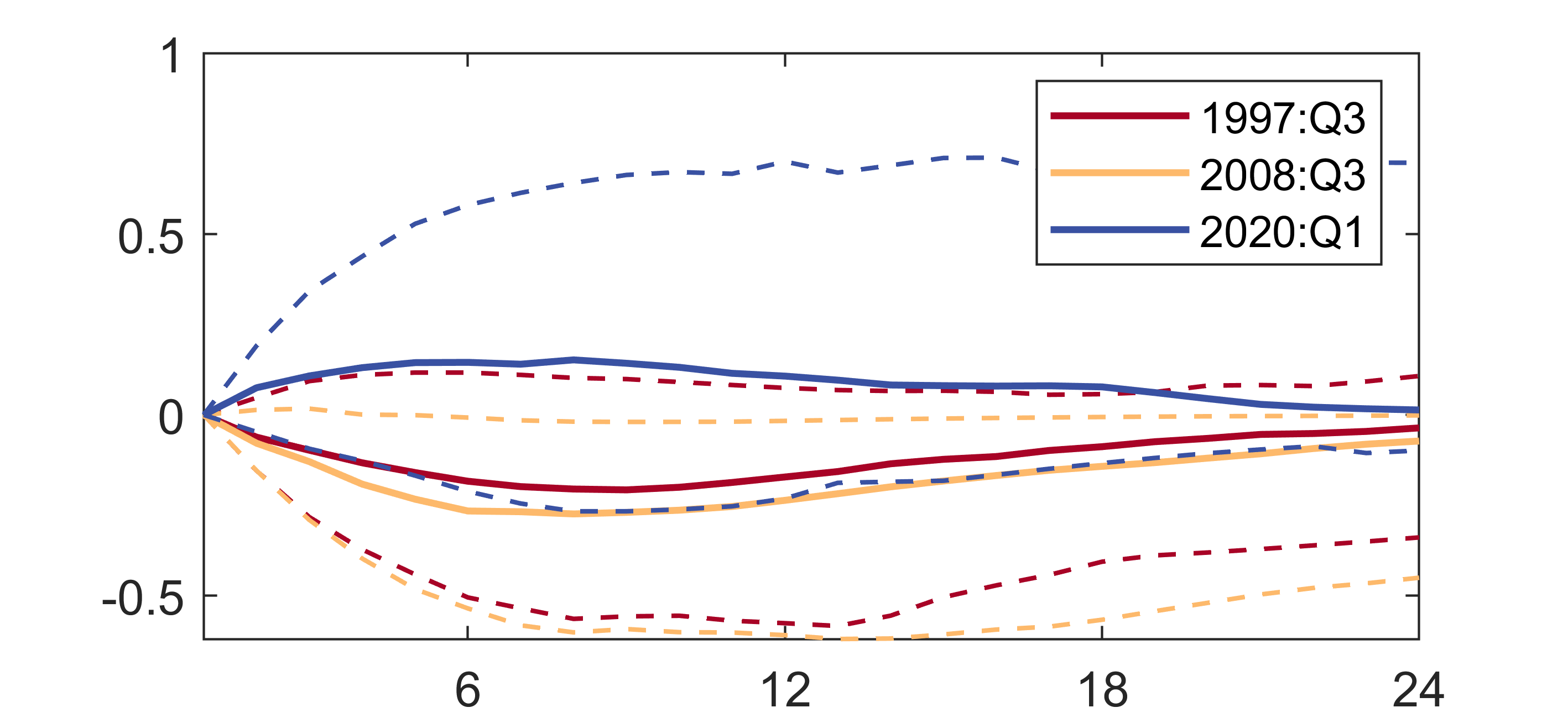

Supplement: Supplementary file 3 [file Data_Sheet_1.ZIP › HK_JPN_1 (3).tif]

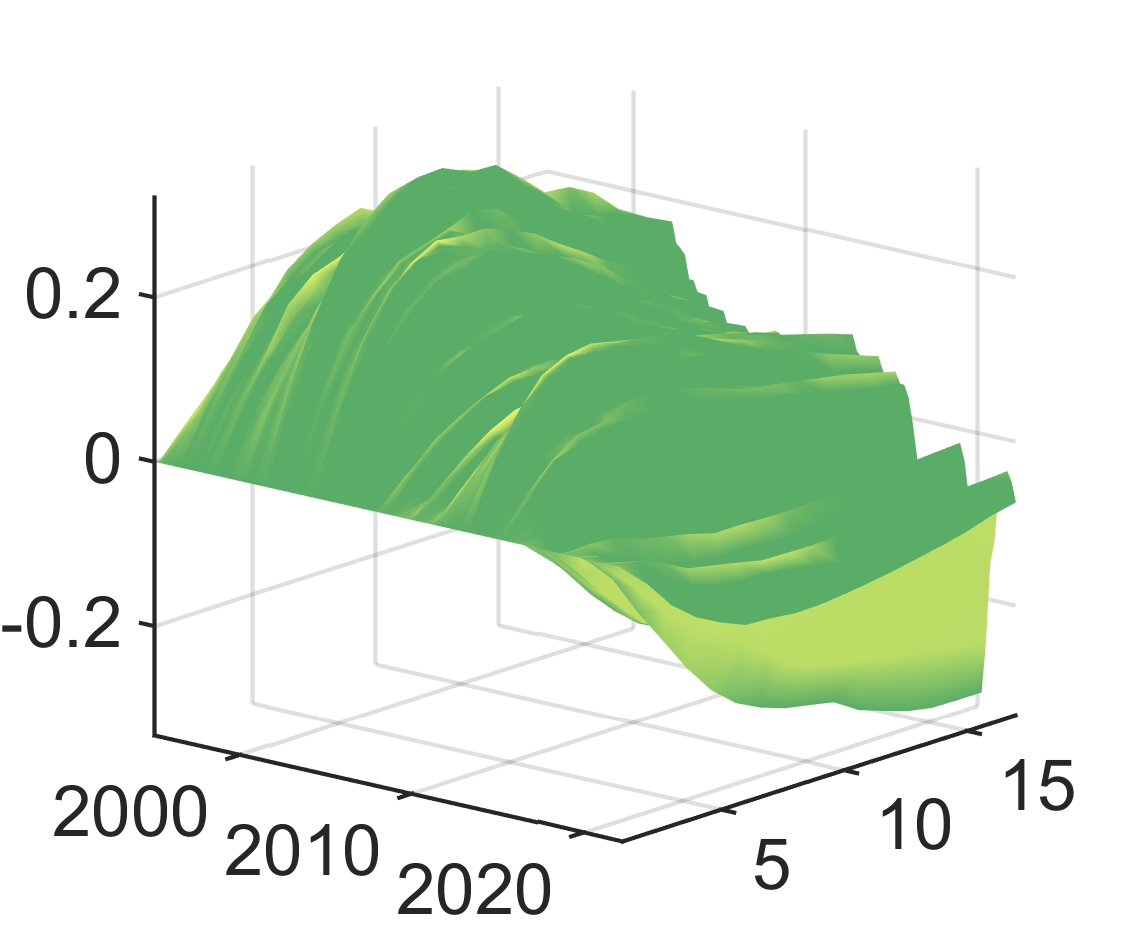

Supplement: Supplementary file 3 [file Data_Sheet_1.ZIP › HK_KR_1 (1).tif]

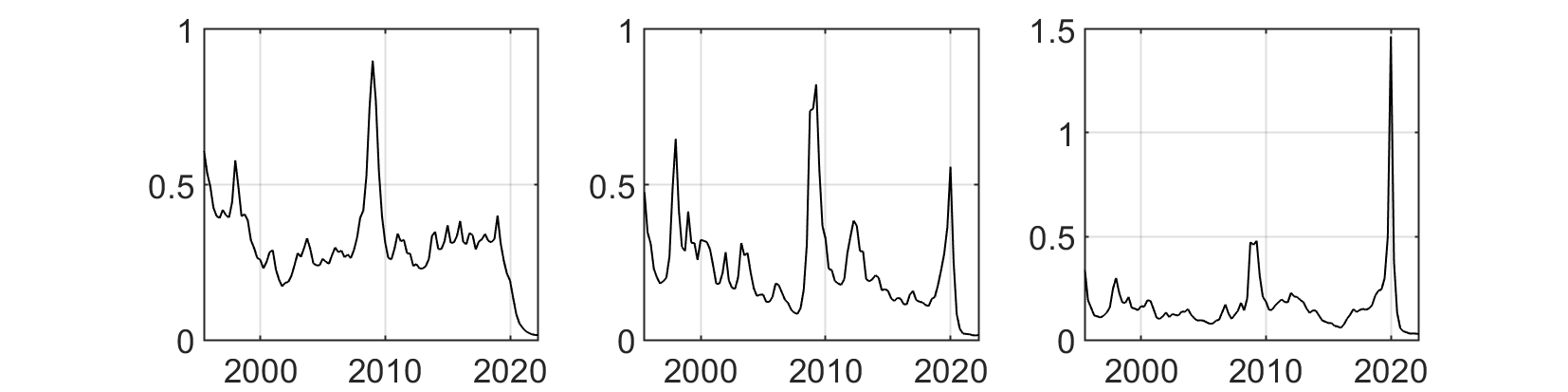

Supplement: Supplementary file 3 [file Data_Sheet_1.ZIP › HK_KR_1 (2).tif]

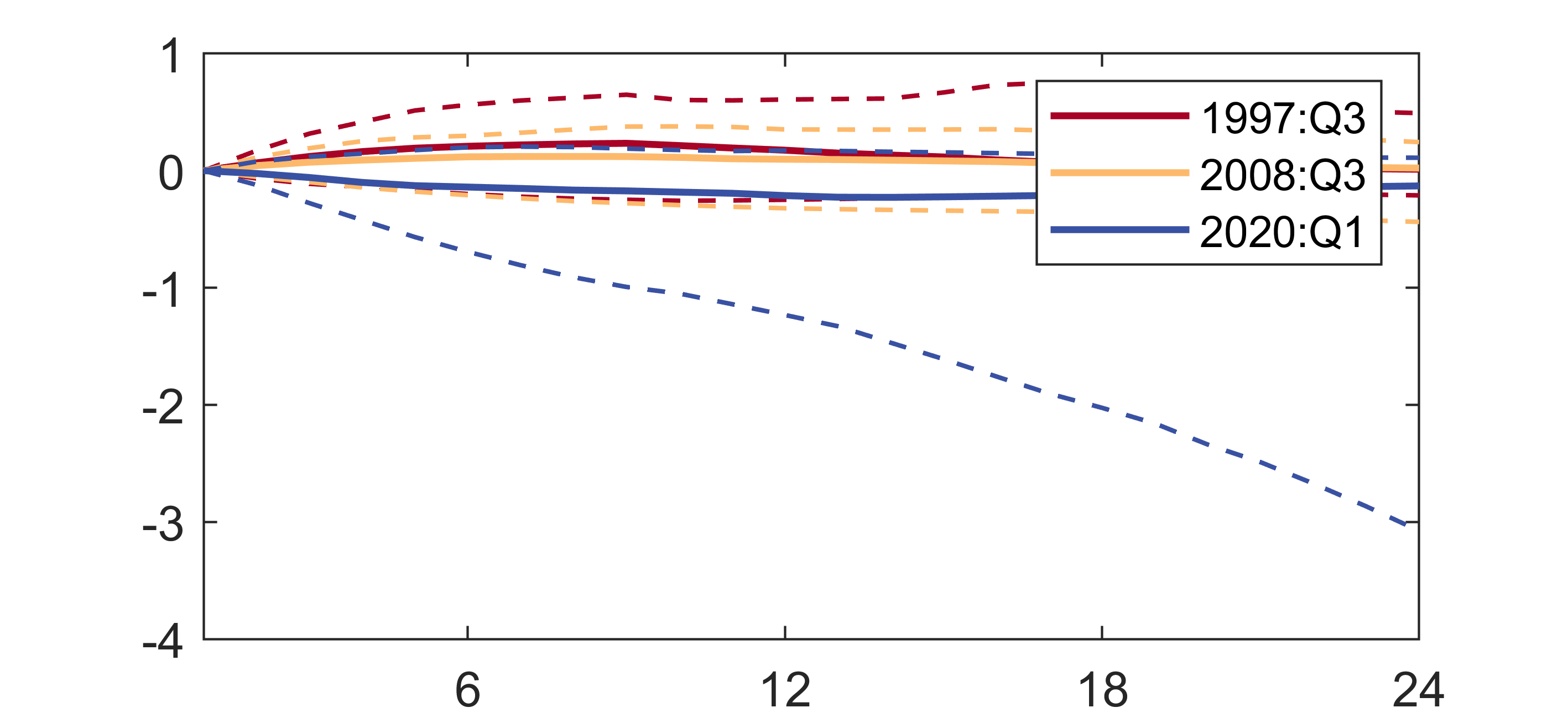

Supplement: Supplementary file 3 [file Data_Sheet_1.ZIP › HK_KR_1 (3).tif]

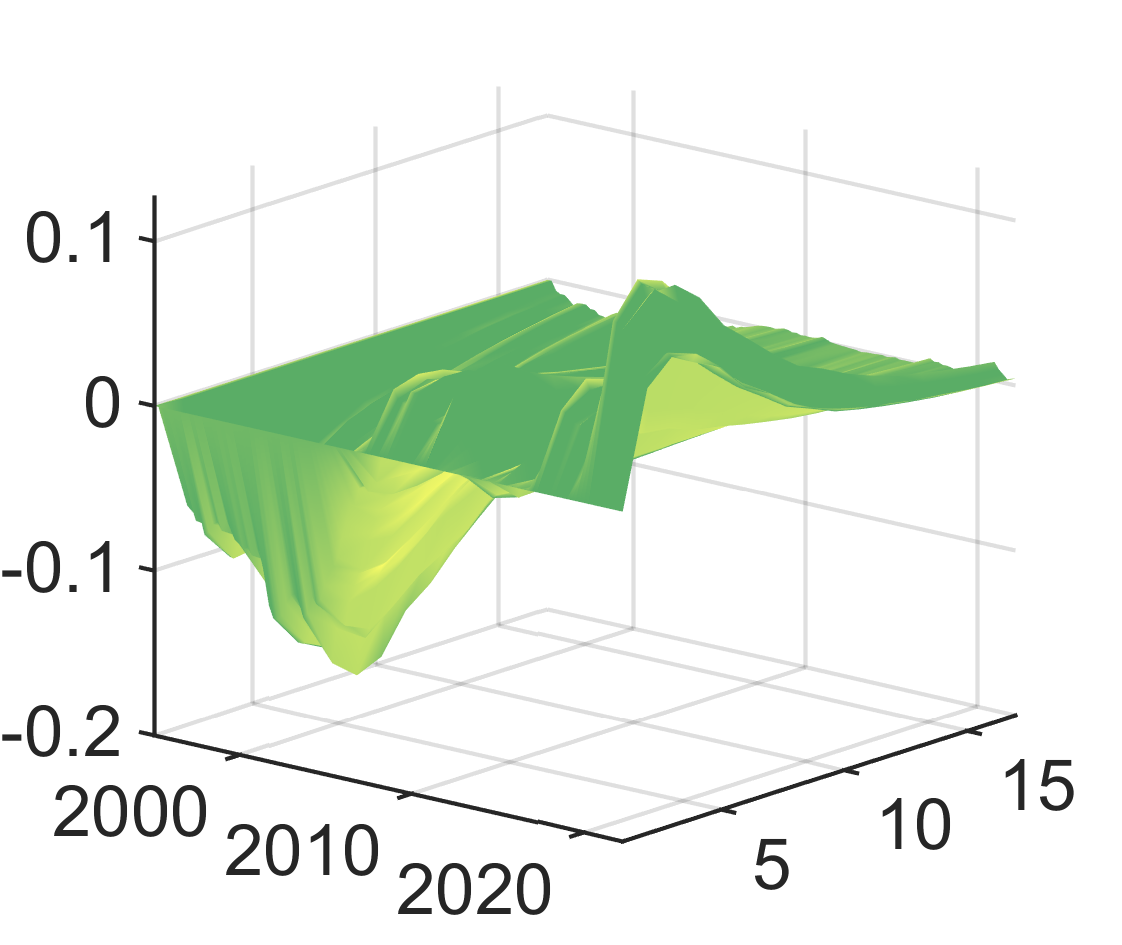

Supplement: Supplementary file 3 [file Data_Sheet_1.ZIP › JPN_CHN_1 (1).tif]

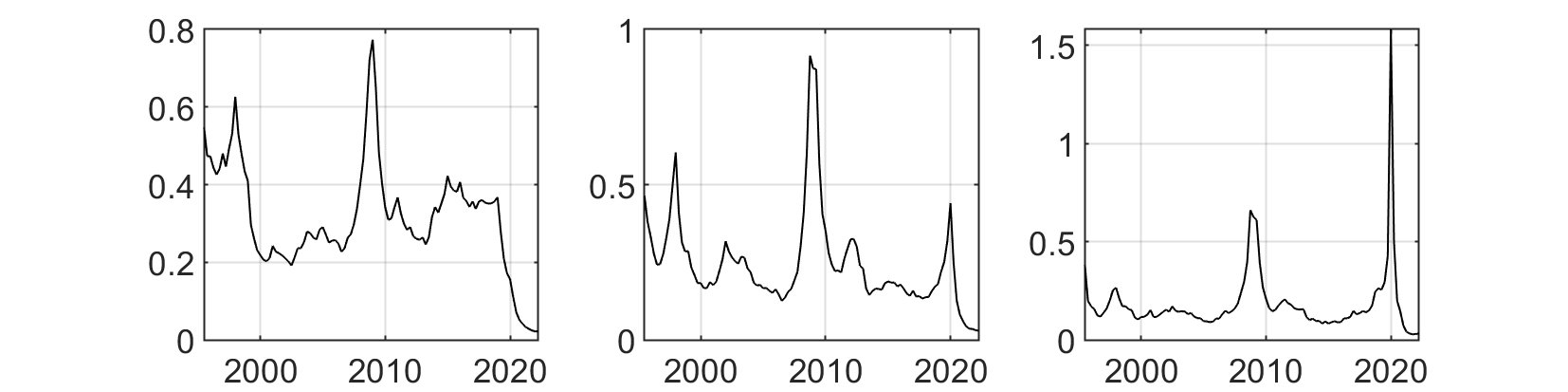

Supplement: Supplementary file 3 [file Data_Sheet_1.ZIP › JPN_CHN_1 (2).tif]

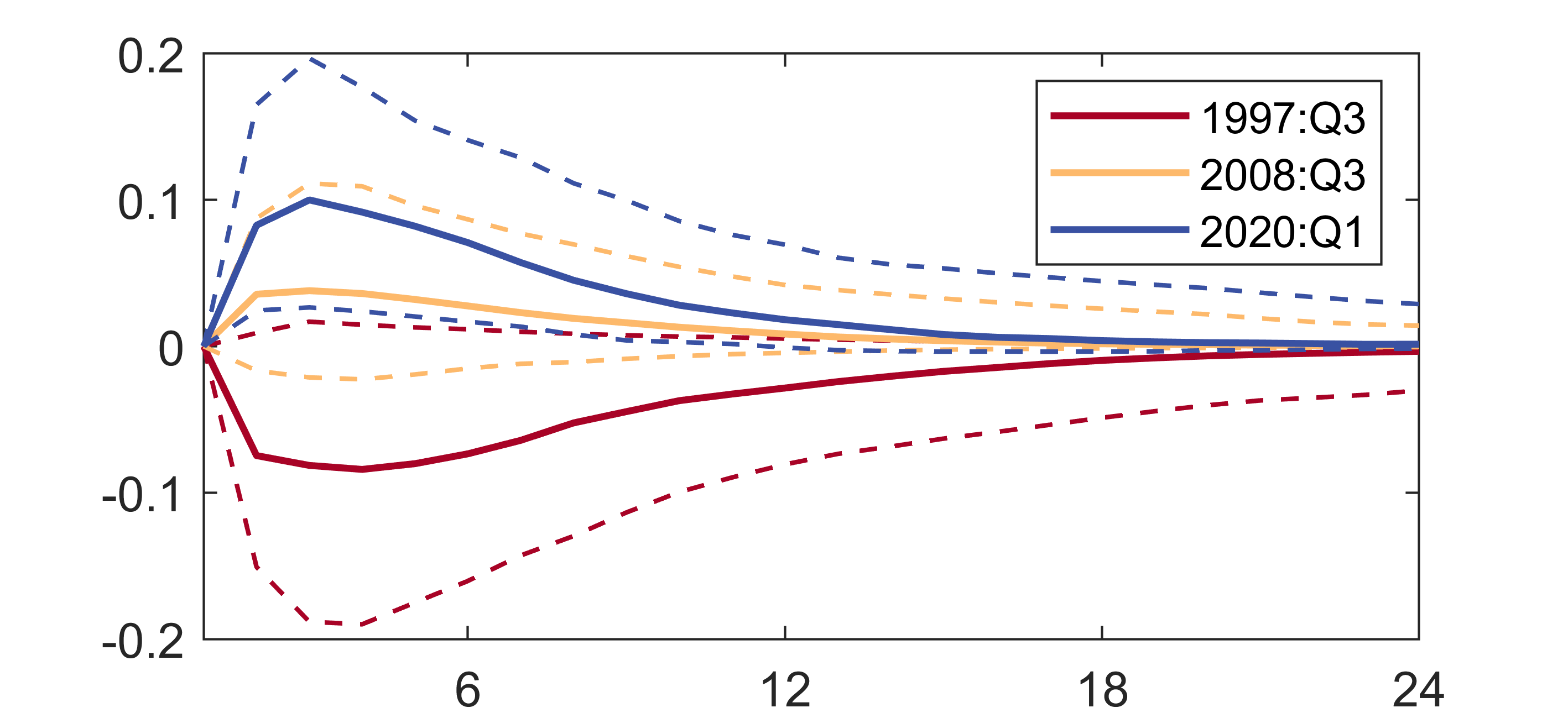

Supplement: Supplementary file 3 [file Data_Sheet_1.ZIP › JPN_CHN_1 (3).tif]

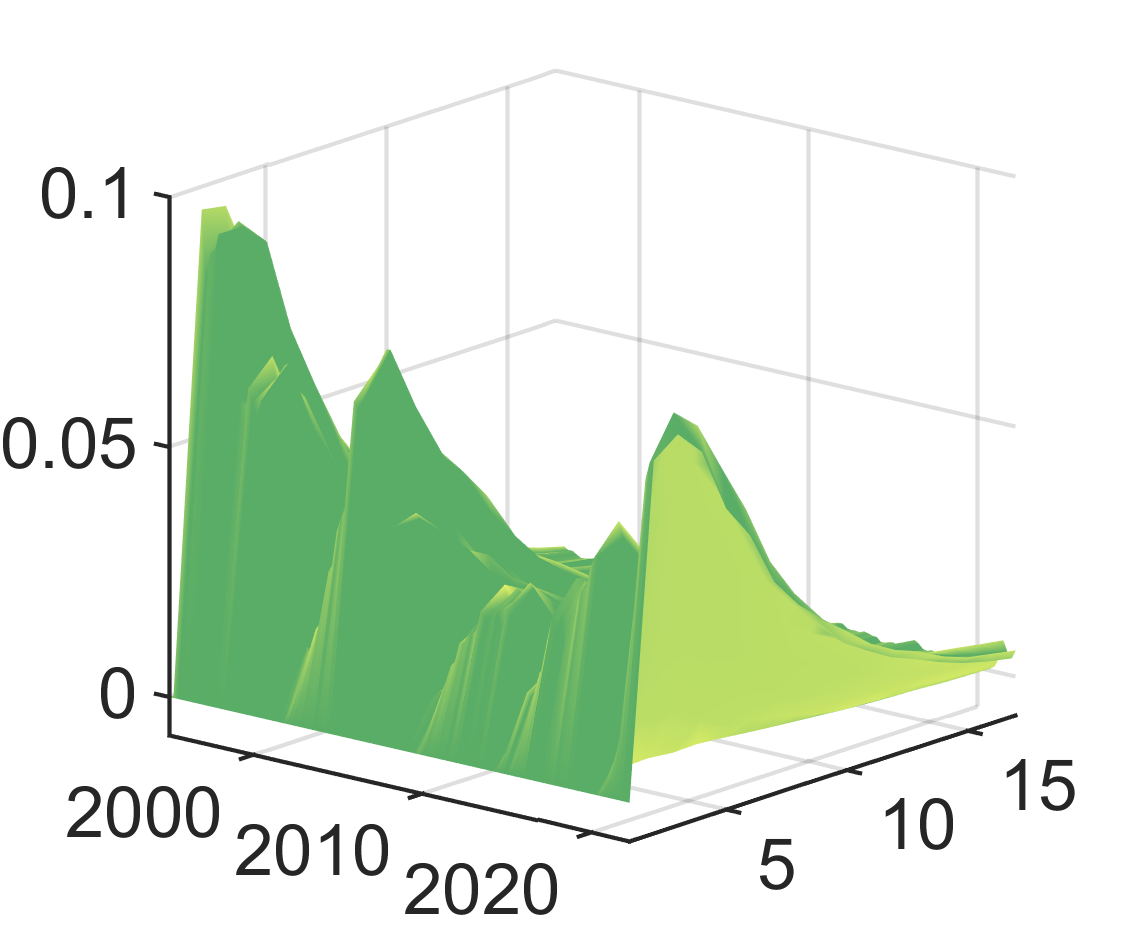

Supplement: Supplementary file 3 [file Data_Sheet_1.ZIP › JPN_HK_1 (1).tif]

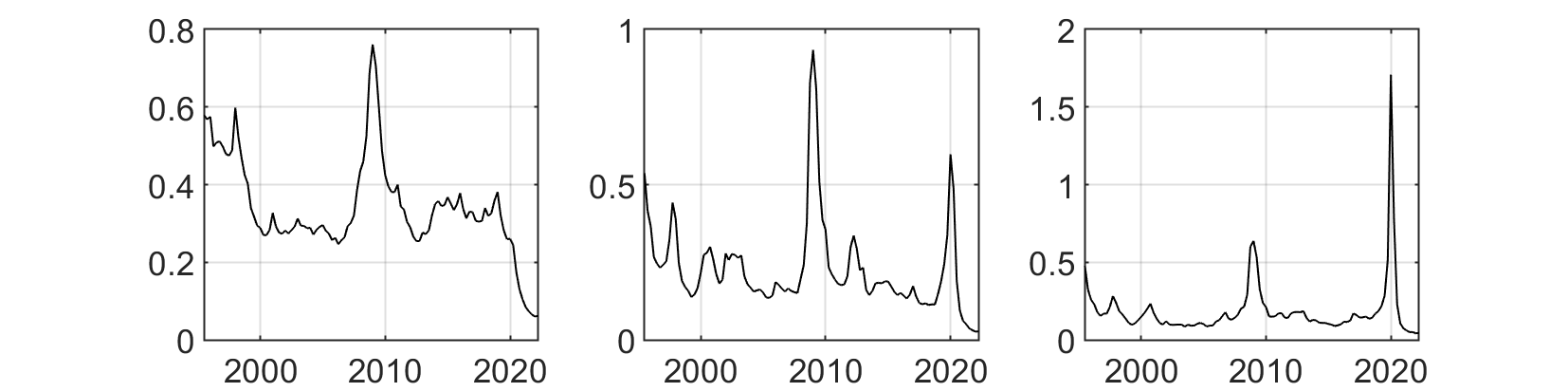

Supplement: Supplementary file 3 [file Data_Sheet_1.ZIP › JPN_HK_1 (2).tif]

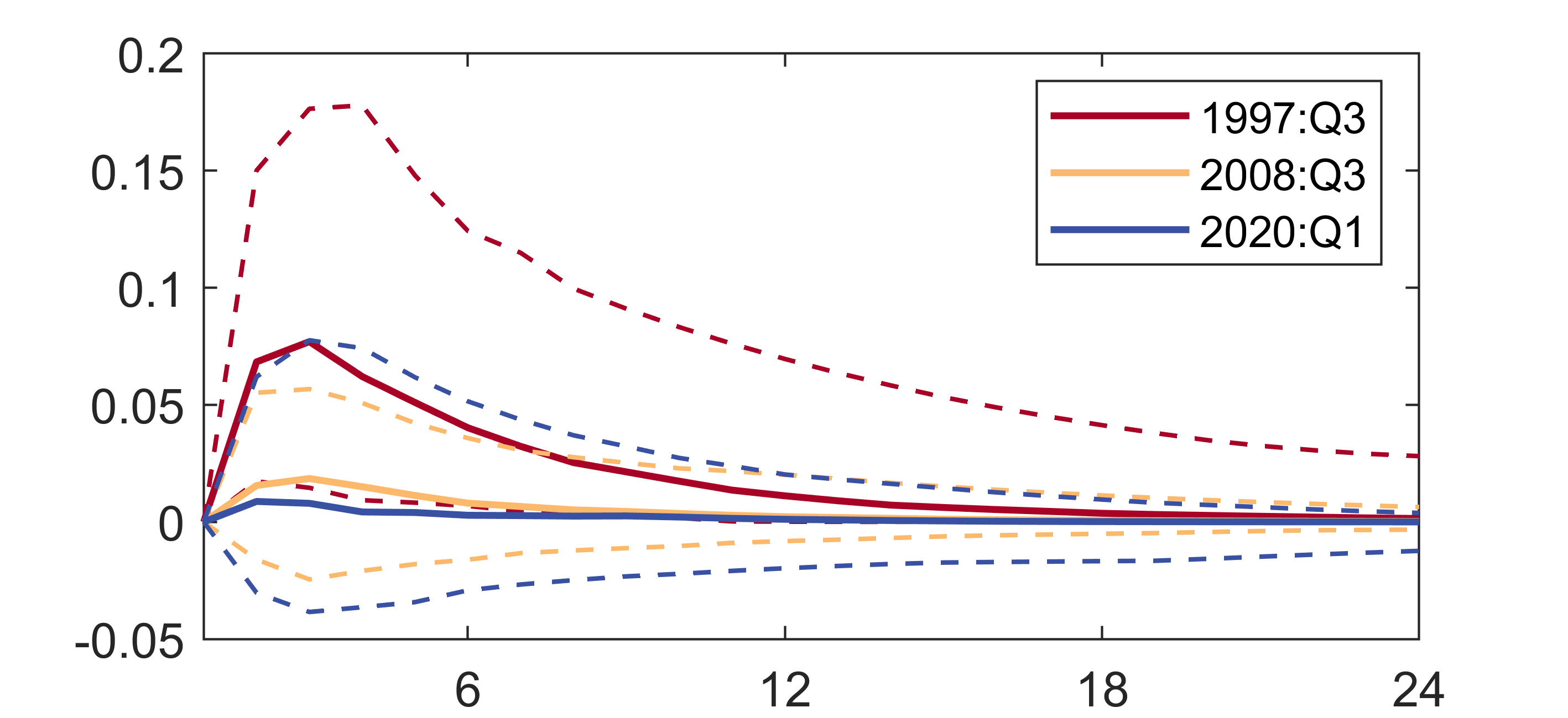

Supplement: Supplementary file 3 [file Data_Sheet_1.ZIP › JPN_HK_1 (3).tif]

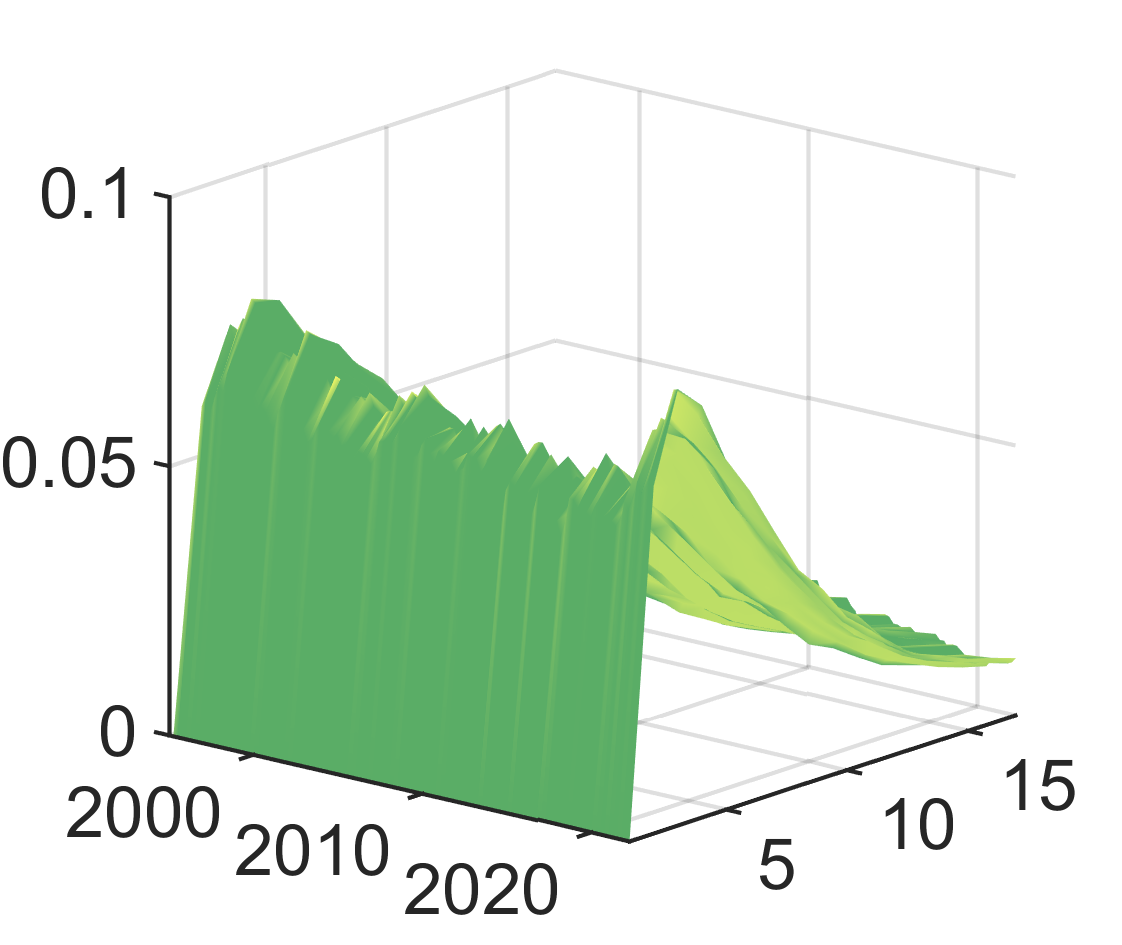

Supplement: Supplementary file 3 [file Data_Sheet_1.ZIP › JPN_KR_1 (1).tif]

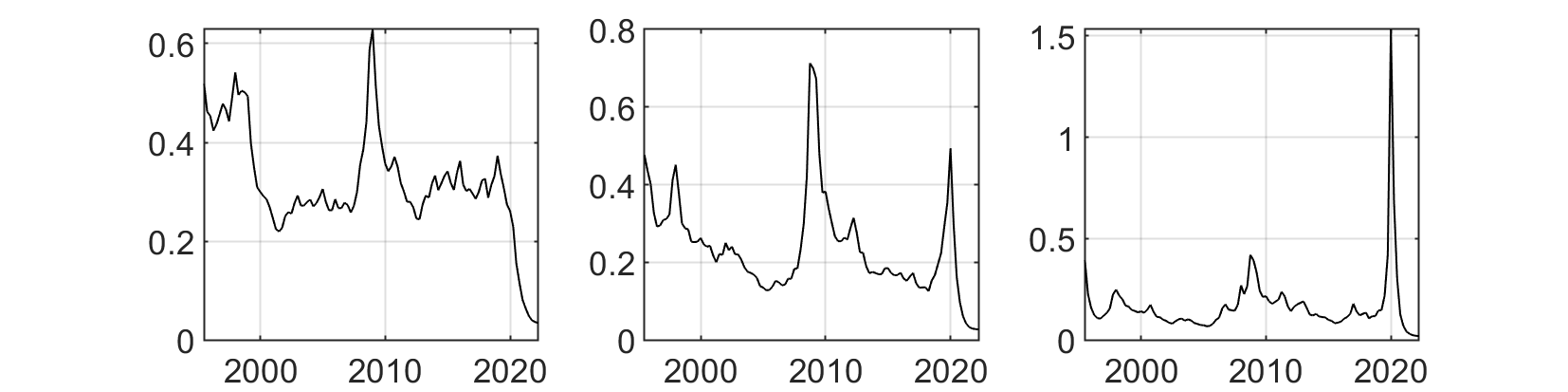

Supplement: Supplementary file 3 [file Data_Sheet_1.ZIP › JPN_KR_1 (2).tif]

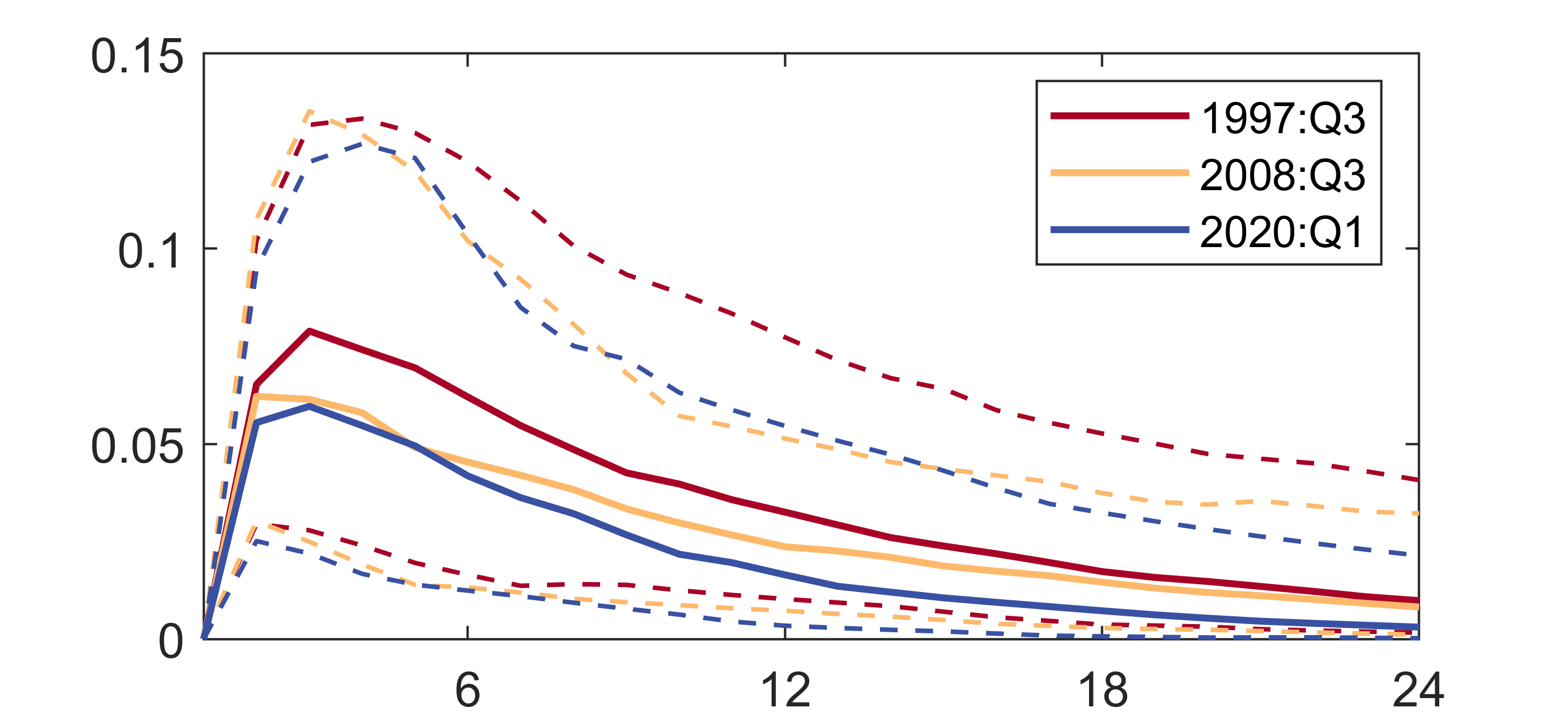

Supplement: Supplementary file 3 [file Data_Sheet_1.ZIP › JPN_KR_1 (3).tif]

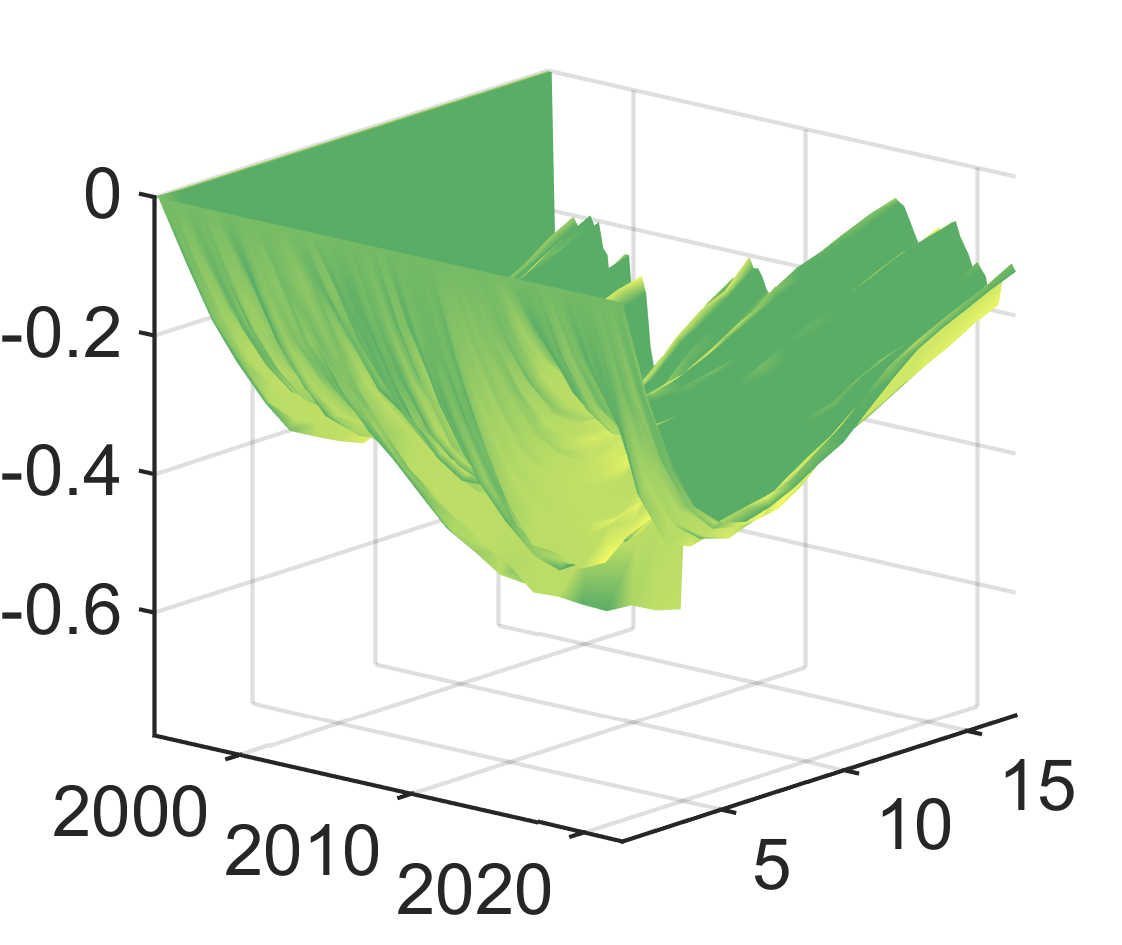

Supplement: Supplementary file 3 [file Data_Sheet_1.ZIP › KR_CHN_1 (1).tif]

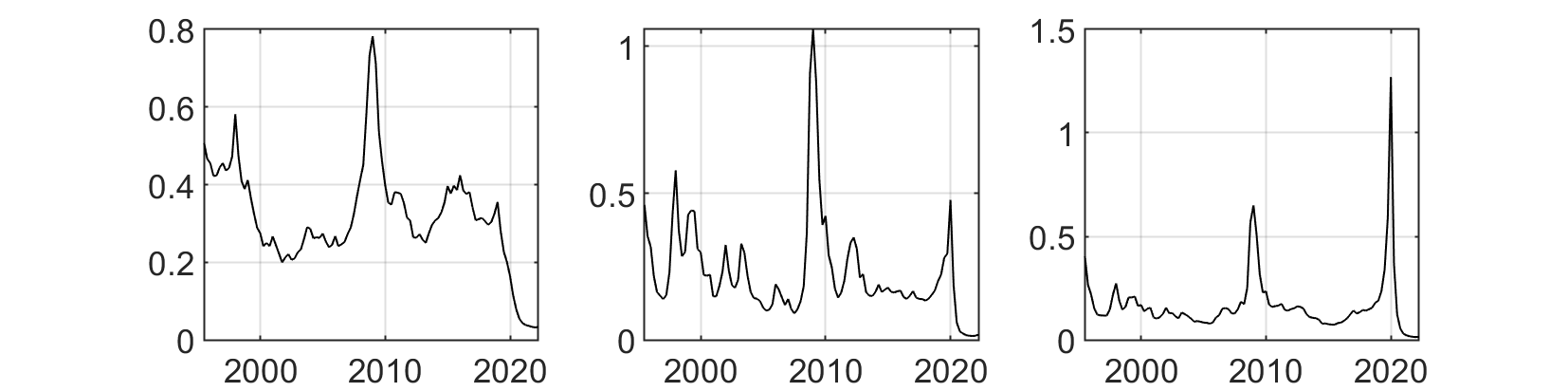

Supplement: Supplementary file 3 [file Data_Sheet_1.ZIP › KR_CHN_1 (2).tif]

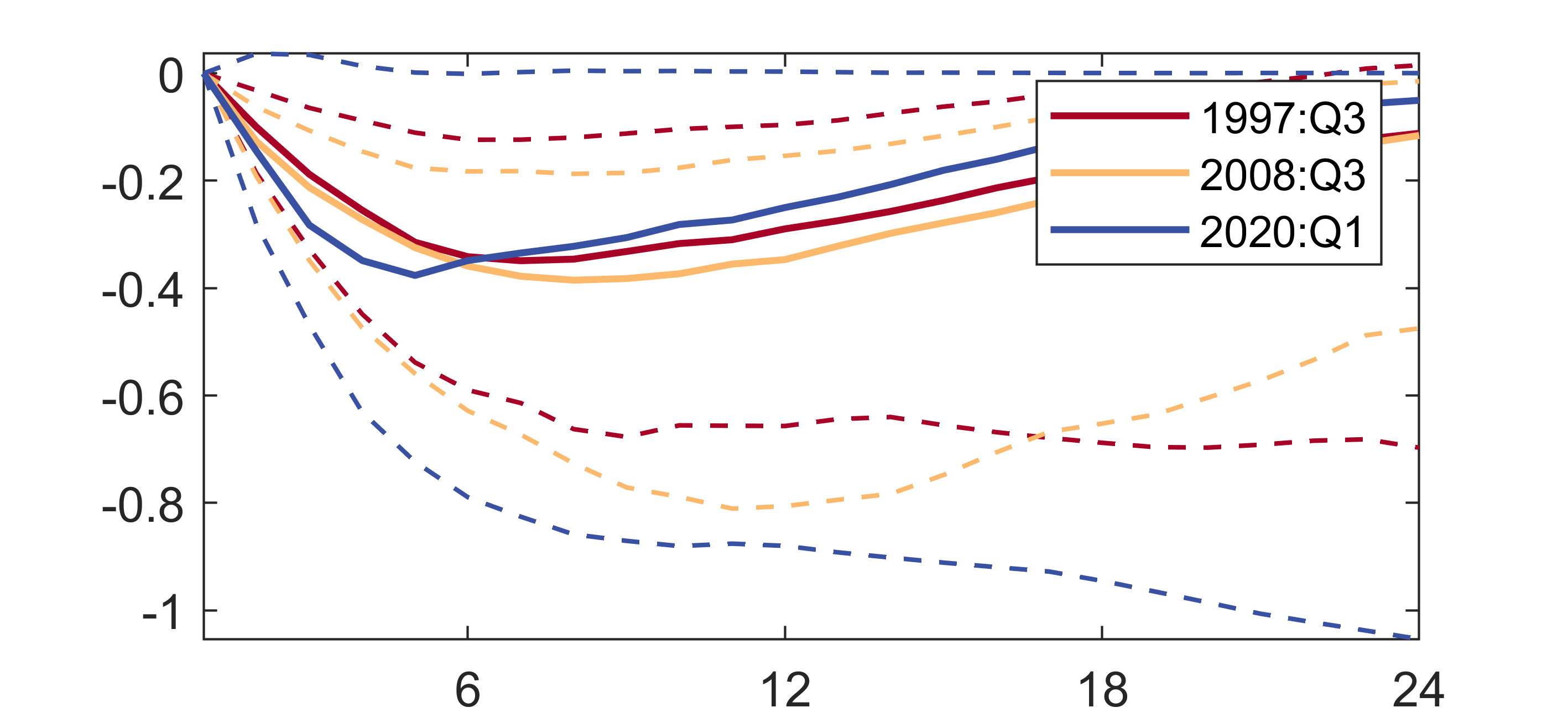

Supplement: Supplementary file 3 [file Data_Sheet_1.ZIP › KR_CHN_1 (3).tif]

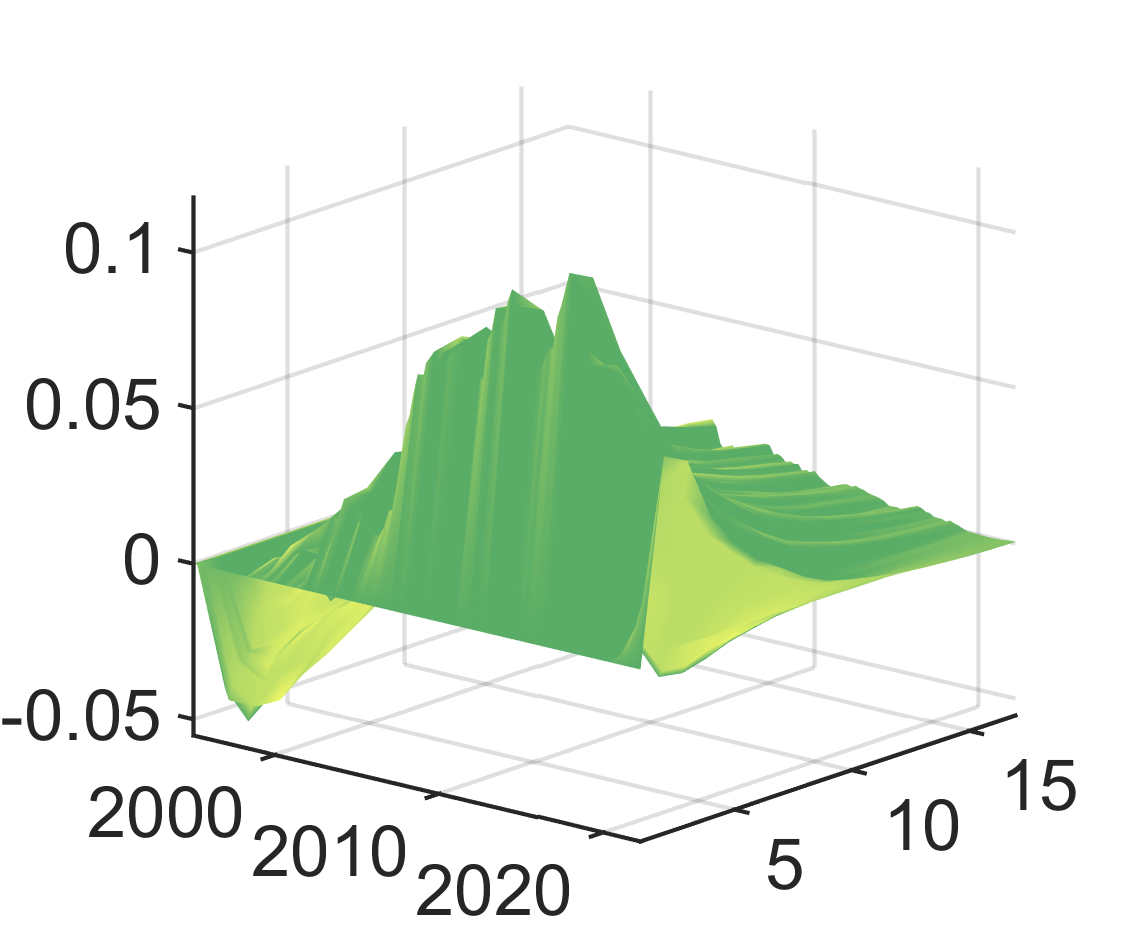

Supplement: Supplementary file 3 [file Data_Sheet_1.ZIP › KR_HK_1 (1).tif]

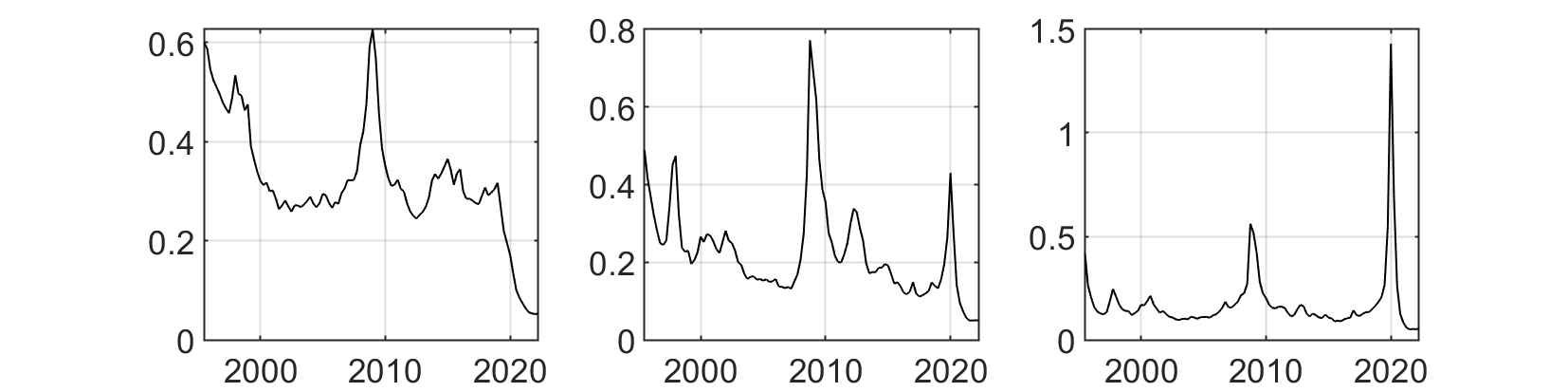

Supplement: Supplementary file 3 [file Data_Sheet_1.ZIP › KR_HK_1 (2).tif]

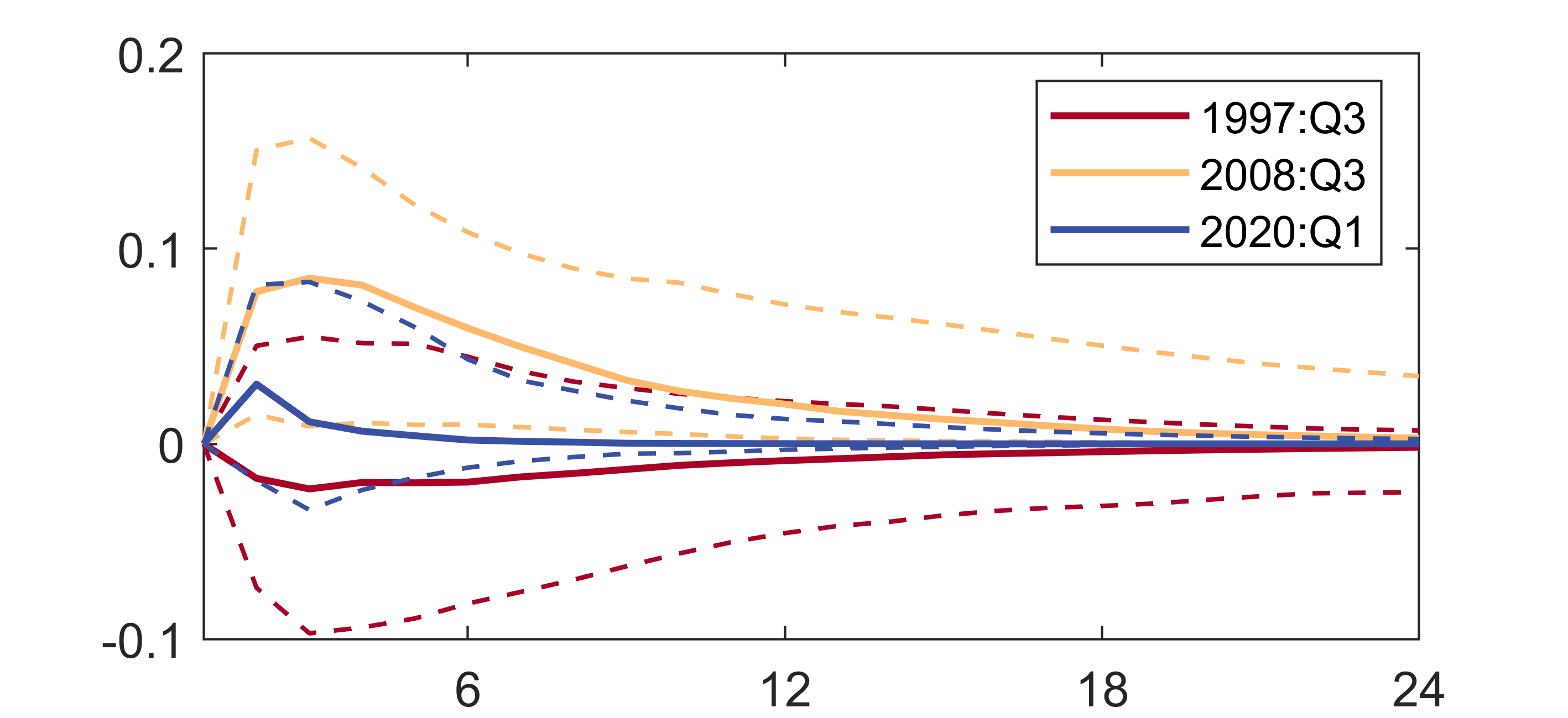

Supplement: Supplementary file 3 [file Data_Sheet_1.ZIP › KR_HK_1 (3).tif]

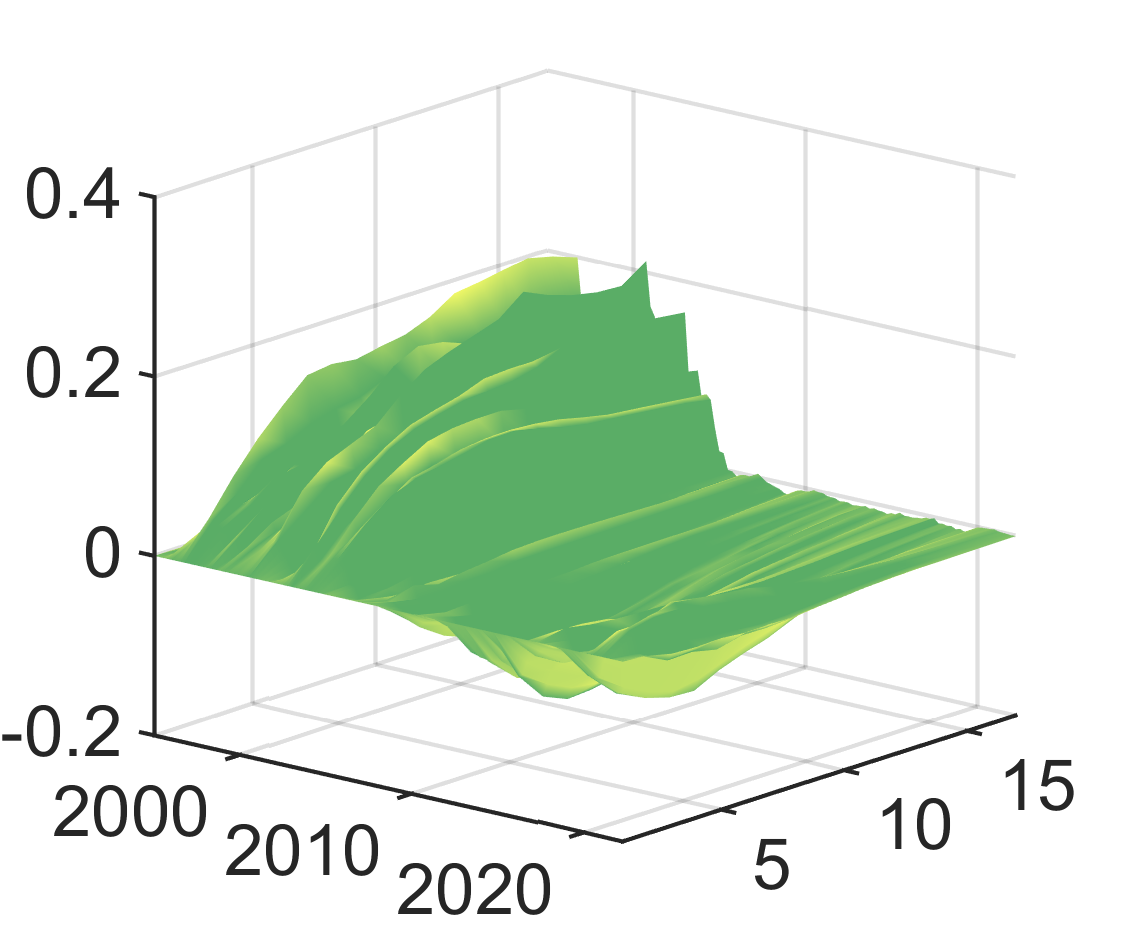

Supplement: Supplementary file 3 [file Data_Sheet_1.ZIP › KR_JPN_1 (1).tif]

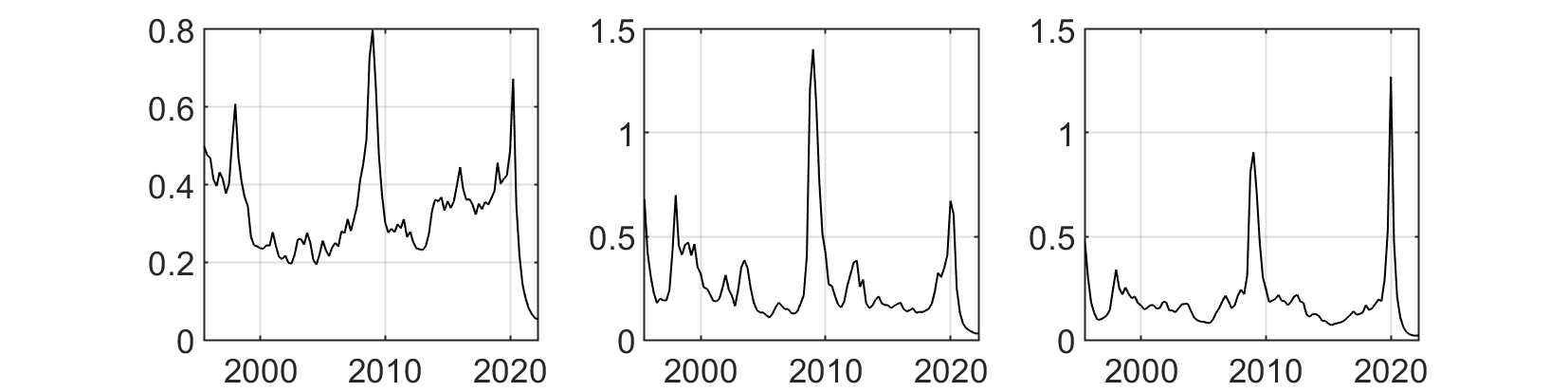

Supplement: Supplementary file 3 [file Data_Sheet_1.ZIP › KR_JPN_1 (2).tif]

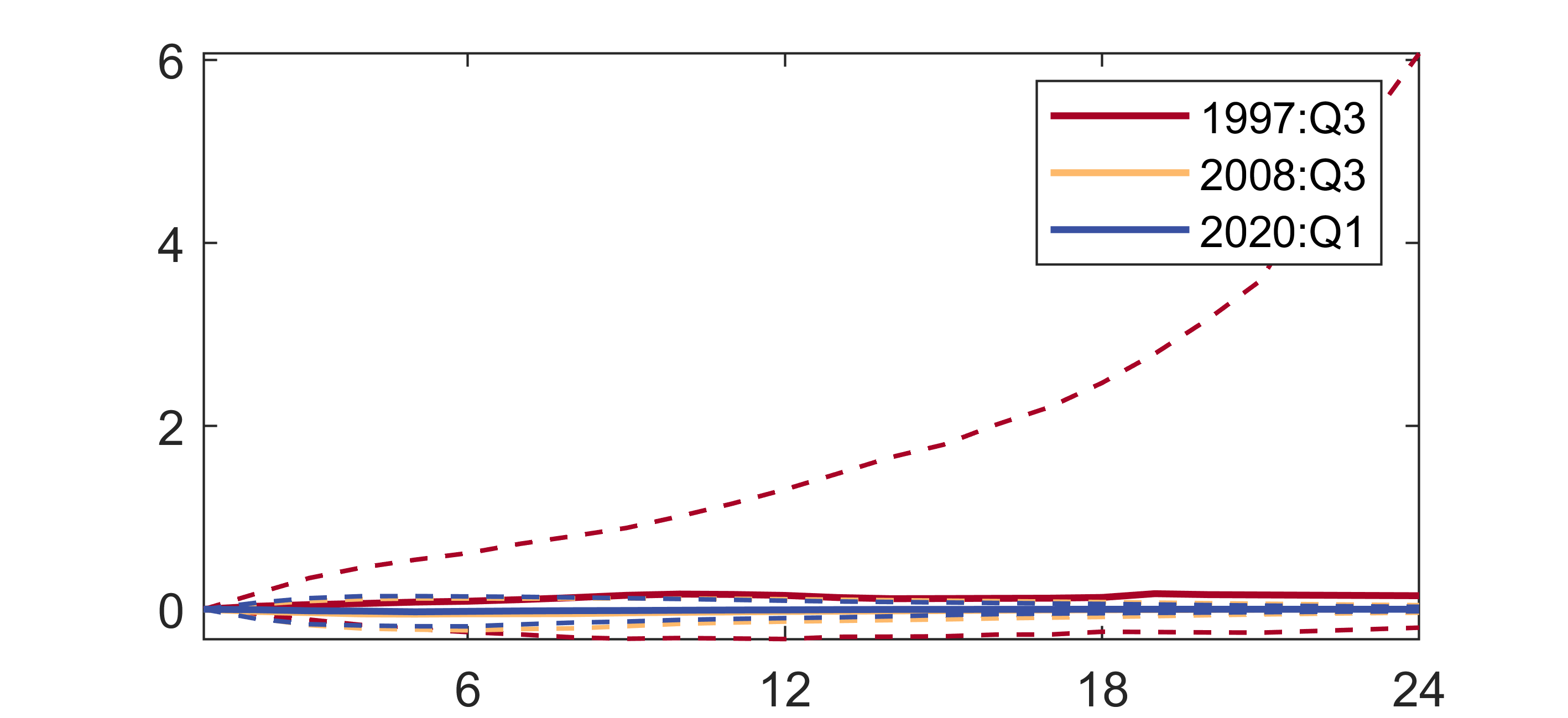

Supplement: Supplementary file 3 [file Data_Sheet_1.ZIP › KR_JPN_1 (3).tif]

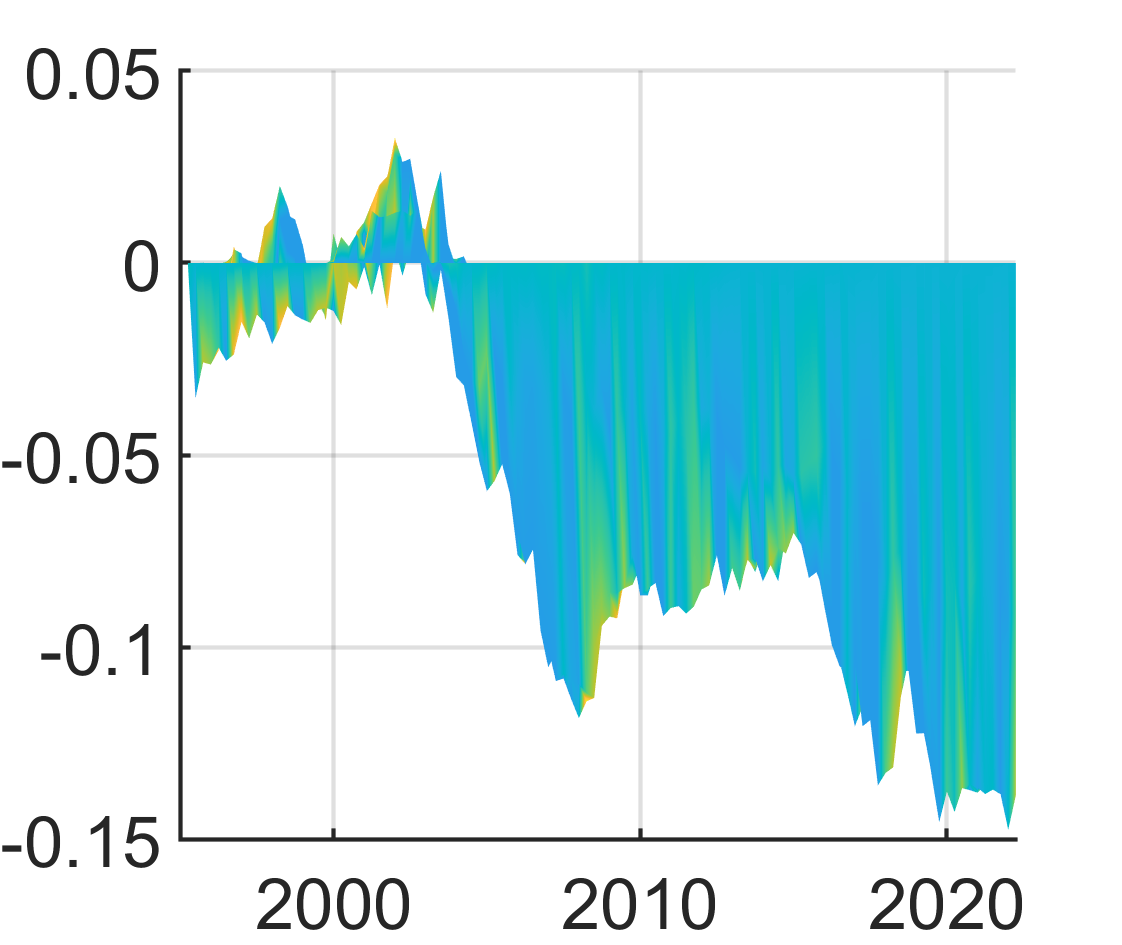

Supplement: Supplementary file 4 [file Data_Sheet_2.ZIP › BM_CHN_2 (1).tif]

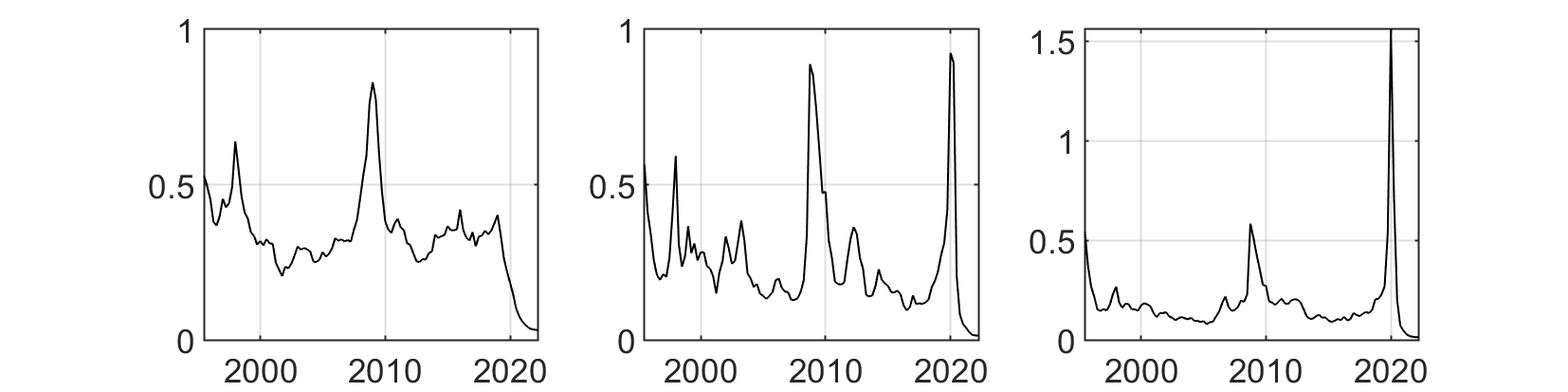

Supplement: Supplementary file 4 [file Data_Sheet_2.ZIP › BM_CHN_2 (2).tif]

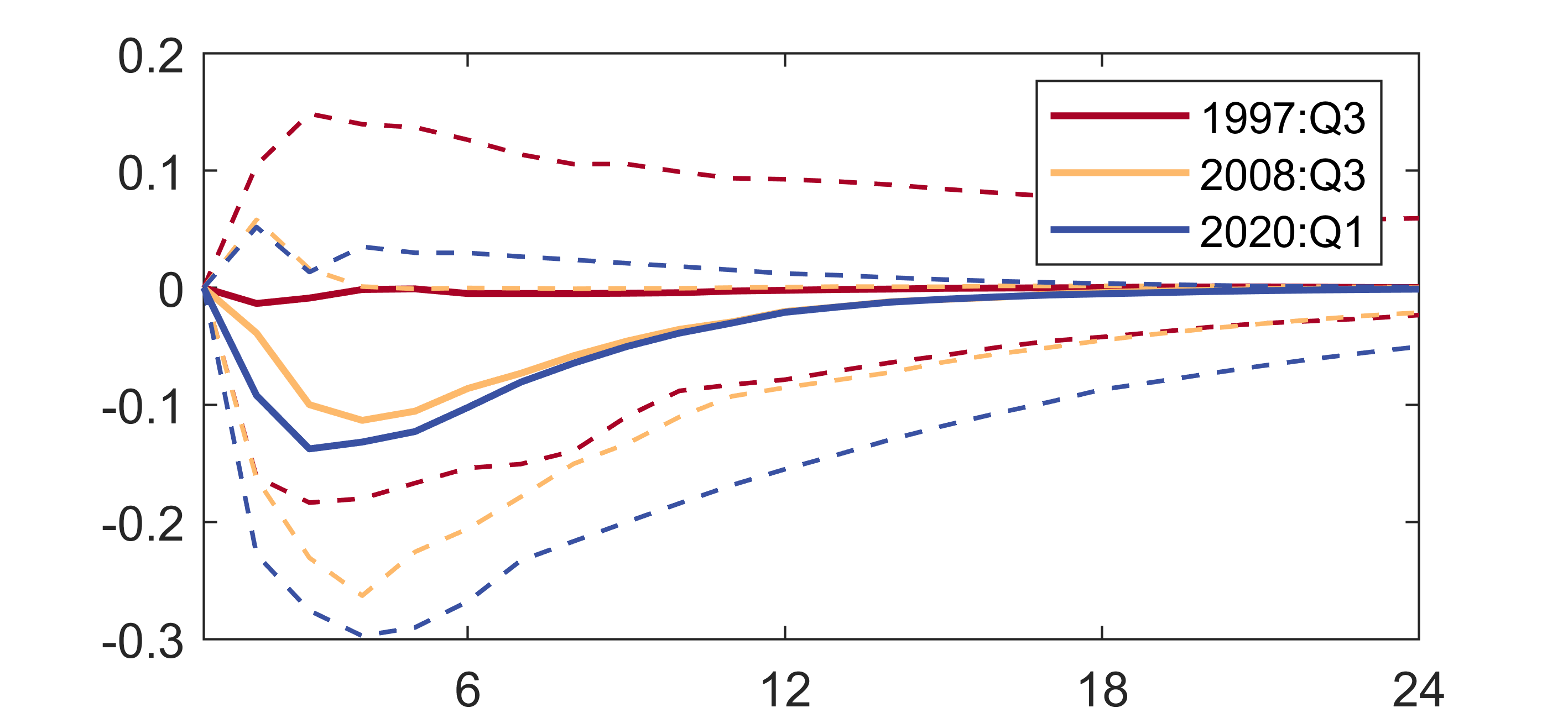

Supplement: Supplementary file 4 [file Data_Sheet_2.ZIP › BM_CHN_2 (3).tif]

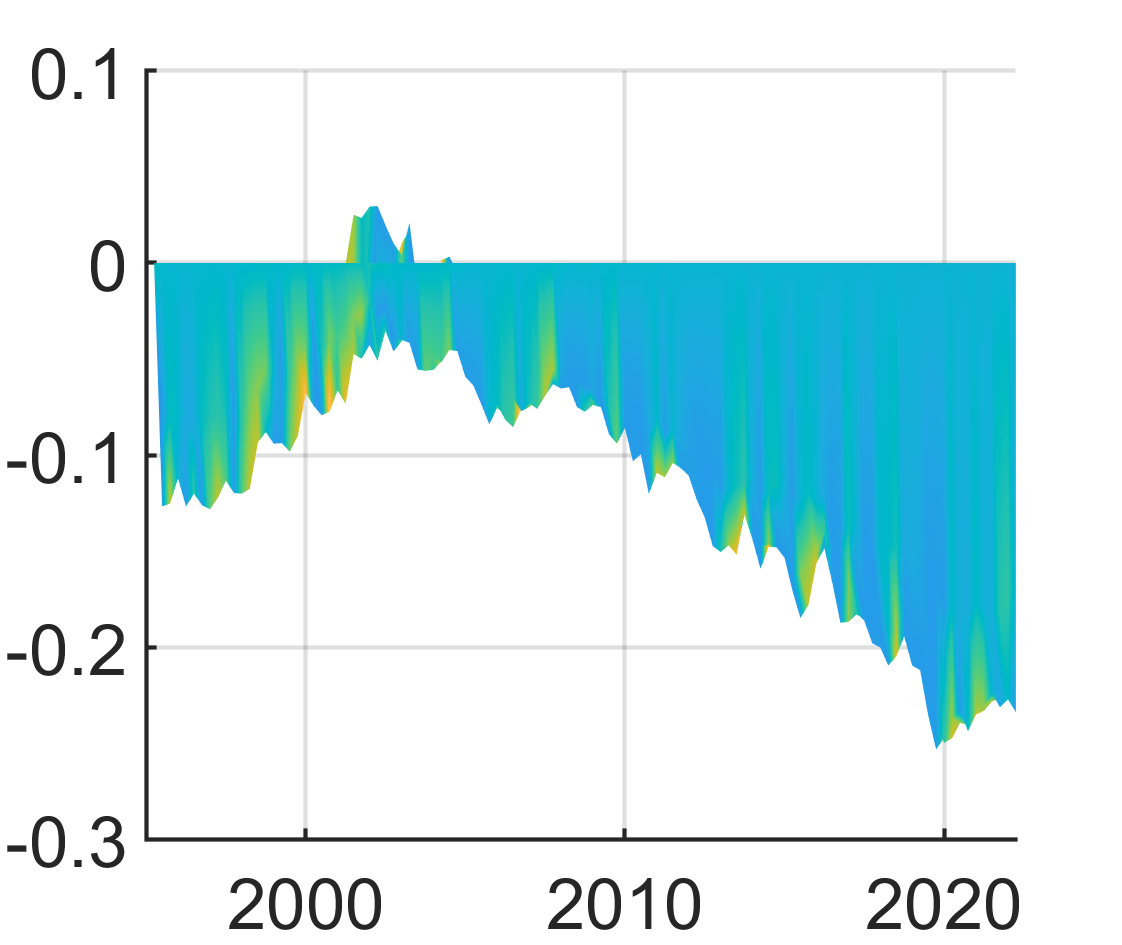

Supplement: Supplementary file 4 [file Data_Sheet_2.ZIP › BM_HK_2 (1).tif]

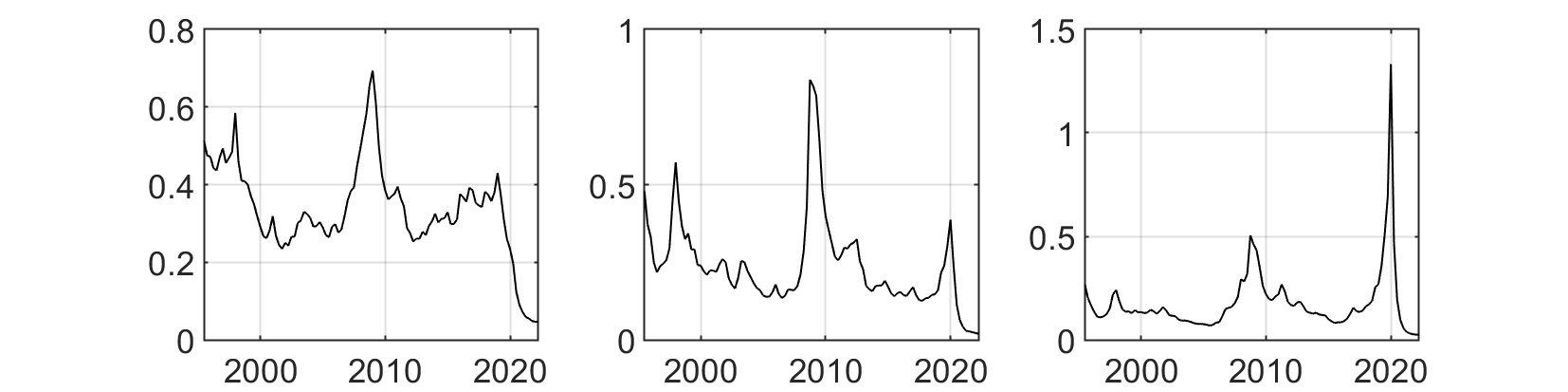

Supplement: Supplementary file 4 [file Data_Sheet_2.ZIP › BM_HK_2 (2).tif]

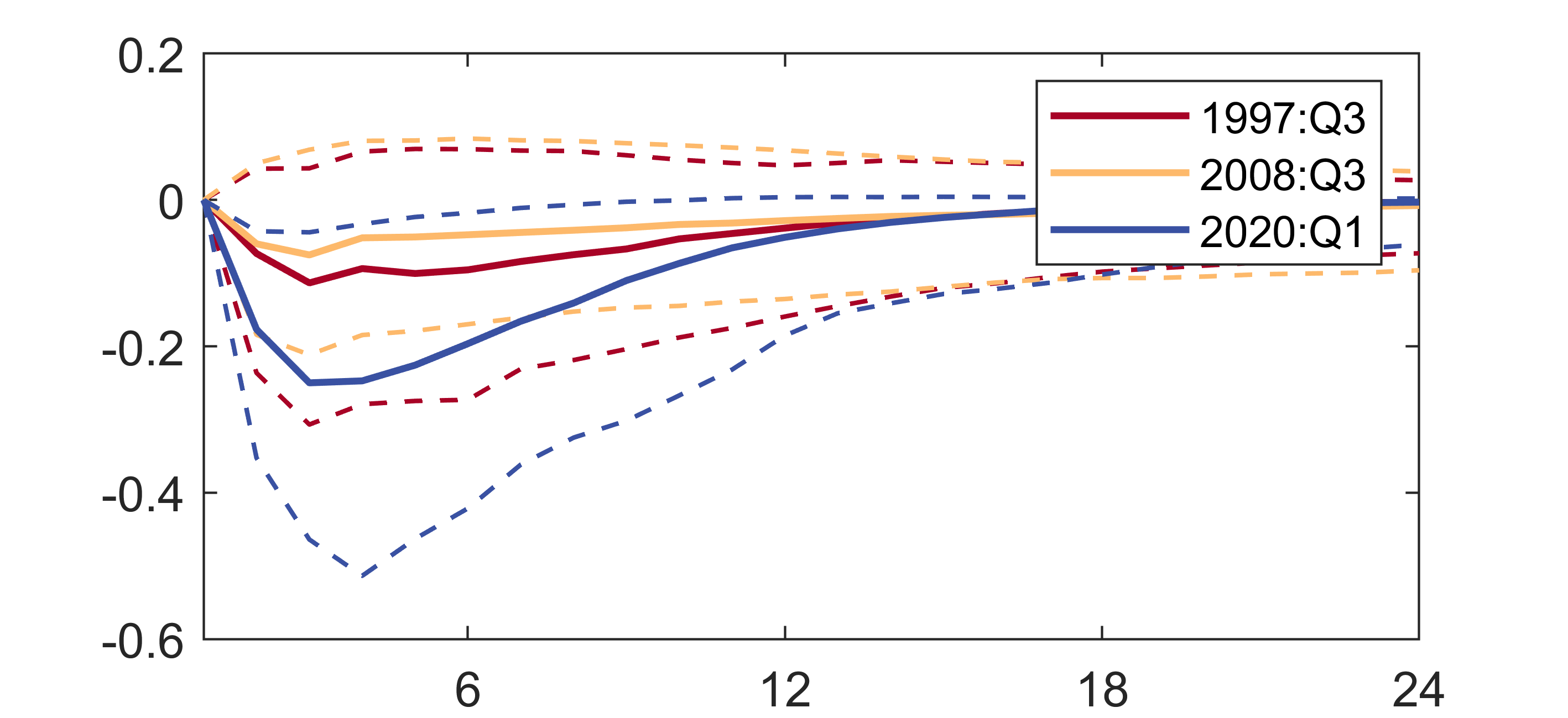

Supplement: Supplementary file 4 [file Data_Sheet_2.ZIP › BM_HK_2 (3).tif]

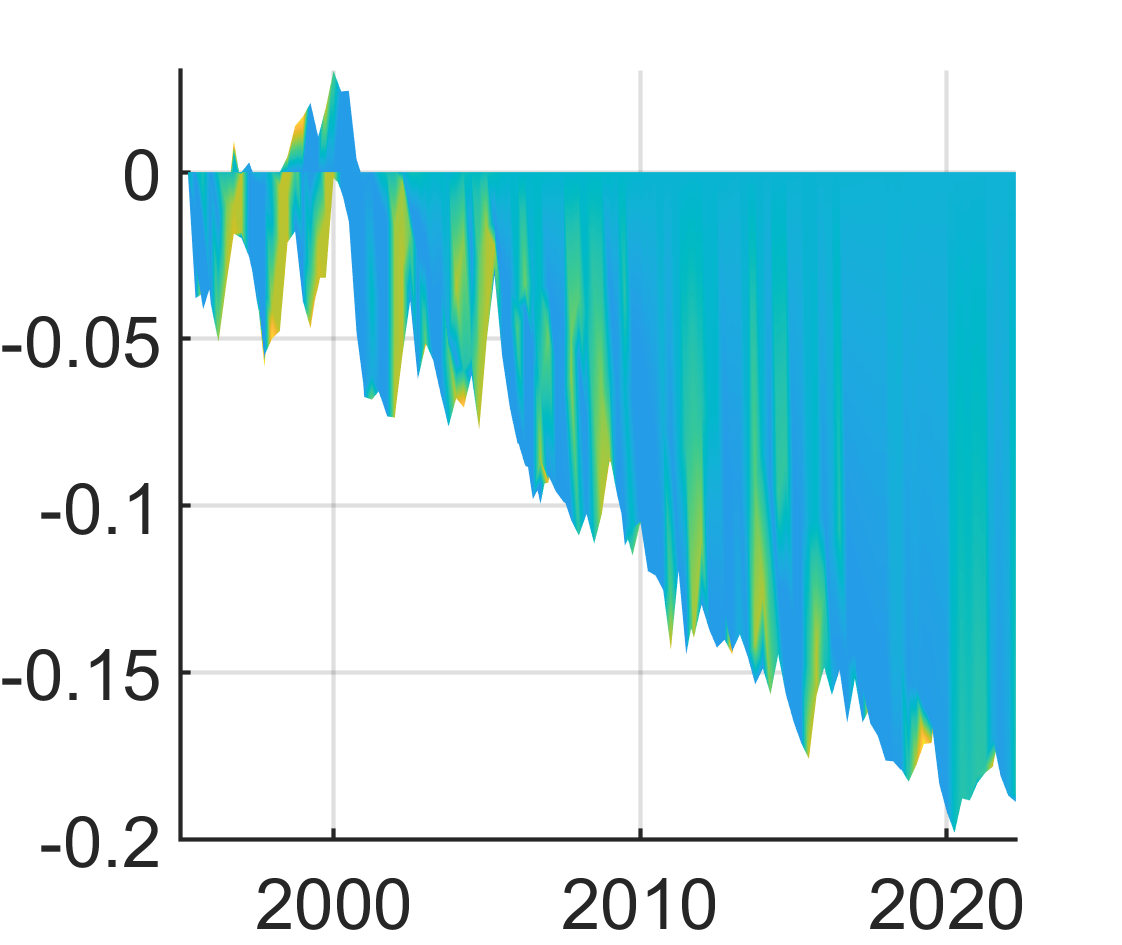

Supplement: Supplementary file 4 [file Data_Sheet_2.ZIP › BM_JPN_2 (1).tif]

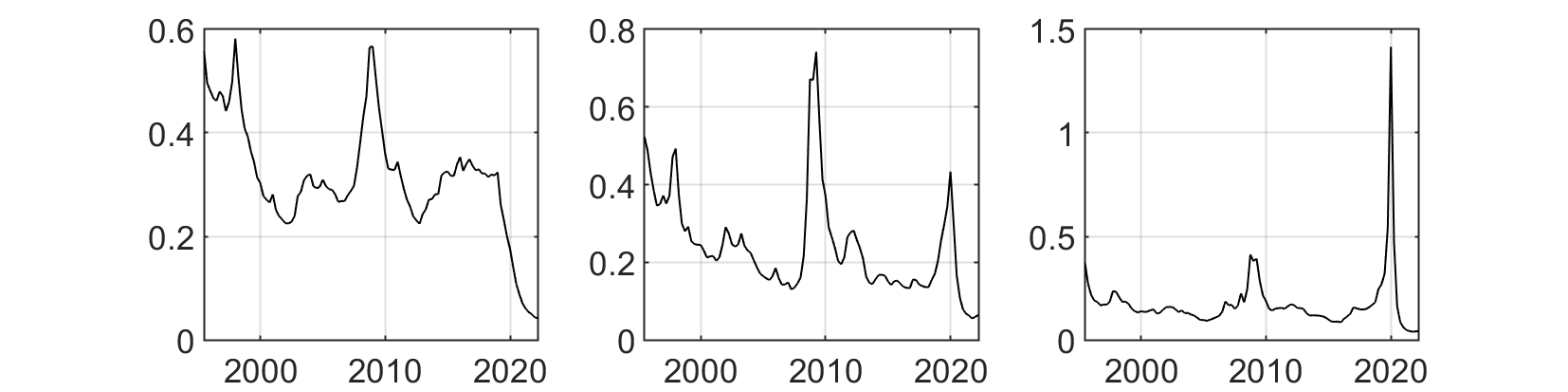

Supplement: Supplementary file 4 [file Data_Sheet_2.ZIP › BM_JPN_2 (2).tif]

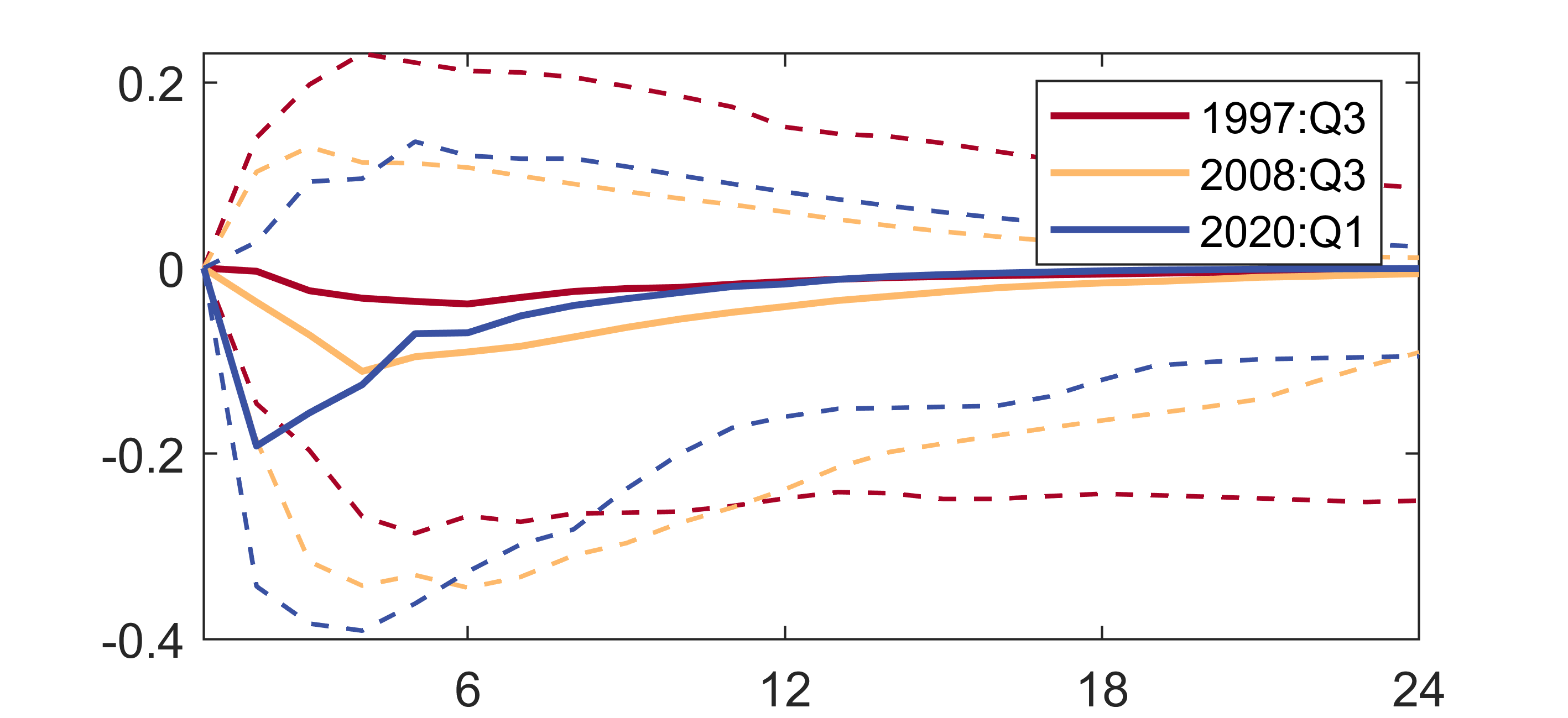

Supplement: Supplementary file 4 [file Data_Sheet_2.ZIP › BM_JPN_2 (3).tif]

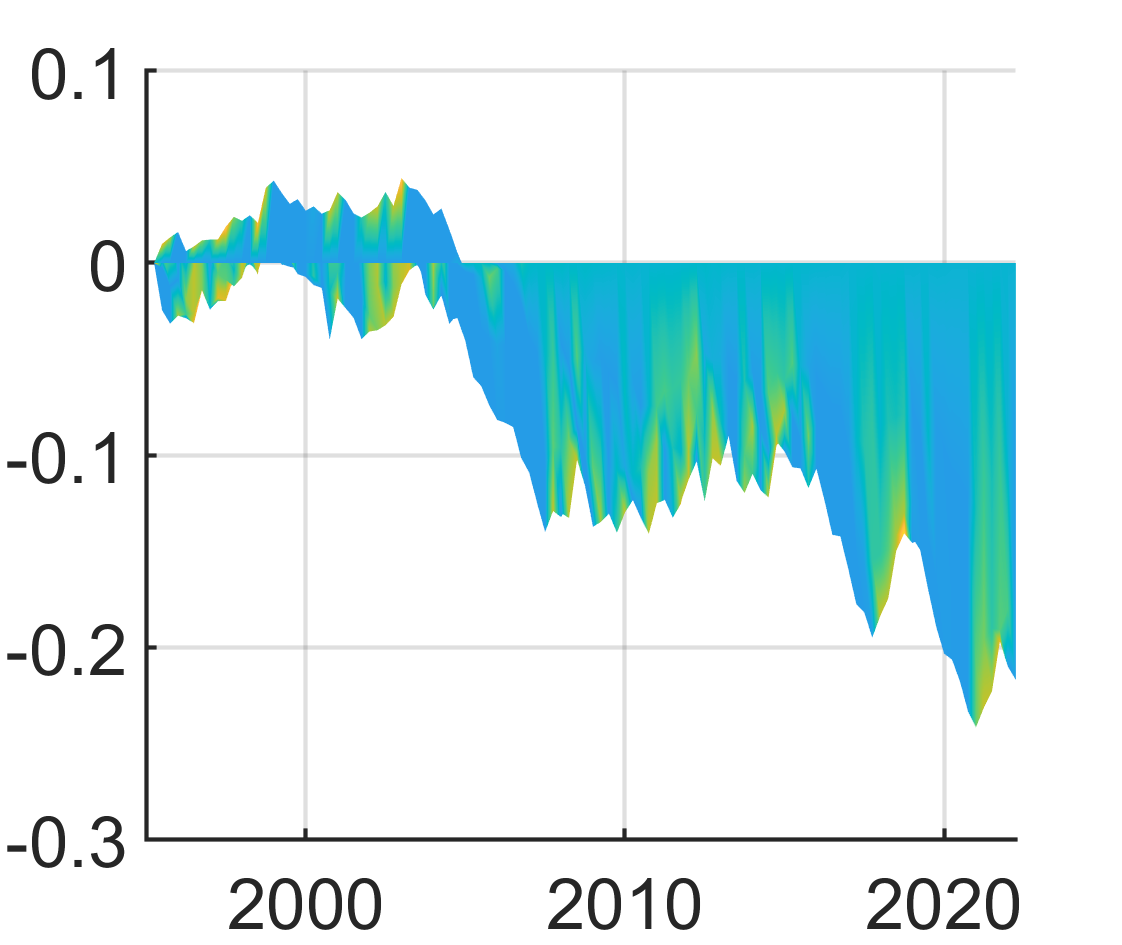

Supplement: Supplementary file 4 [file Data_Sheet_2.ZIP › BM_KR_2 (1).tif]

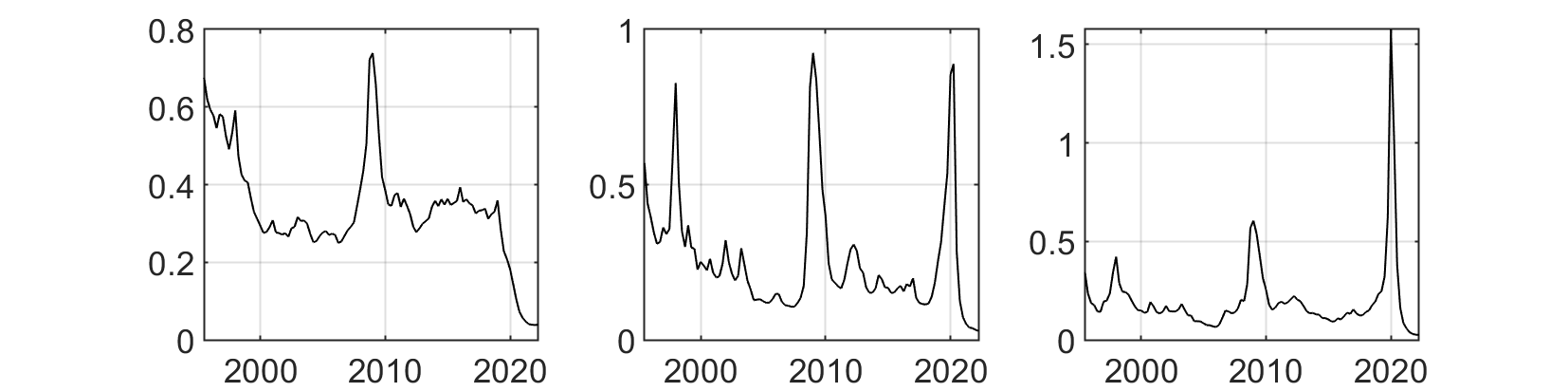

Supplement: Supplementary file 4 [file Data_Sheet_2.ZIP › BM_KR_2 (2).tif]

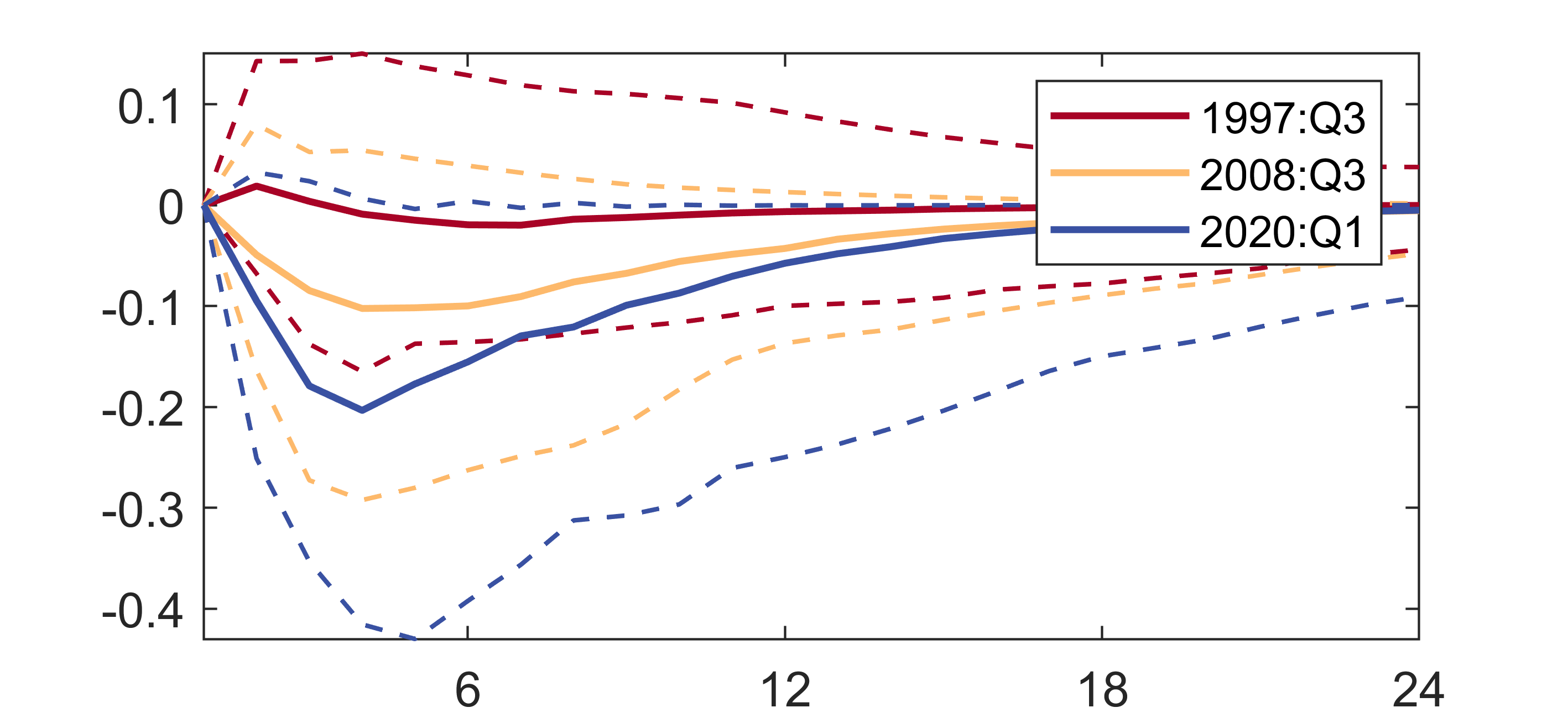

Supplement: Supplementary file 4 [file Data_Sheet_2.ZIP › BM_KR_2 (3).tif]

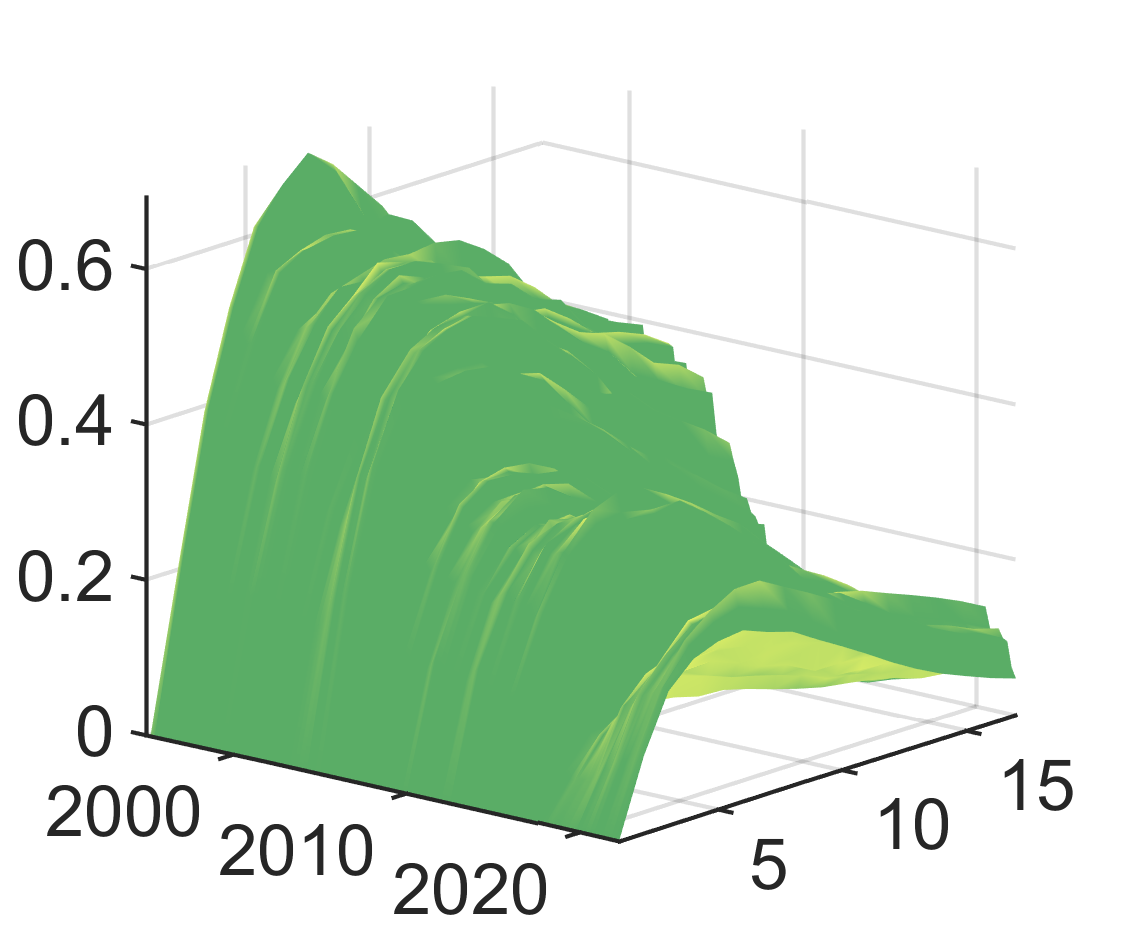

Supplement: Supplementary file 4 [file Data_Sheet_2.ZIP › CHN_HK_2 (1).tif]

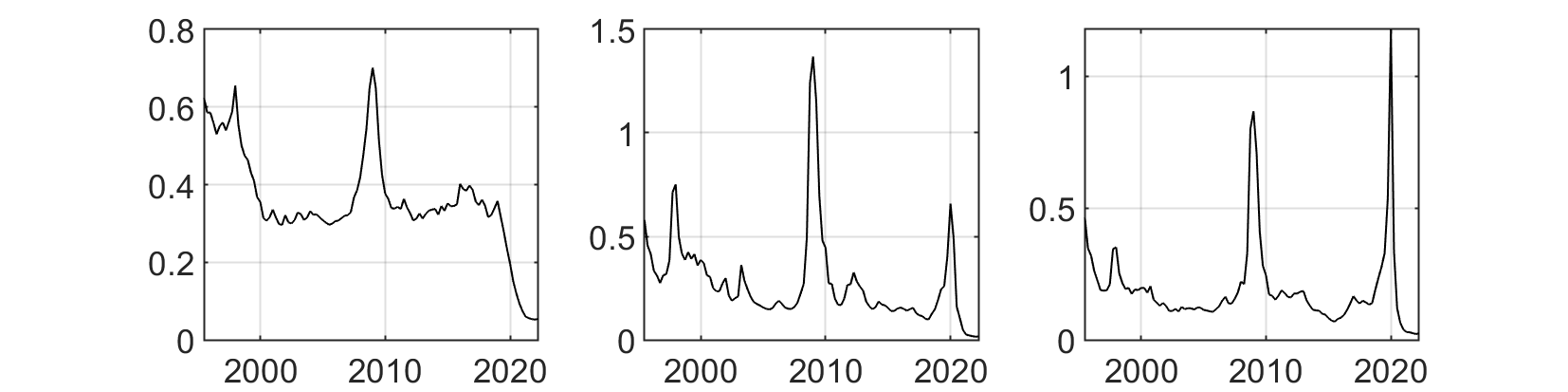

Supplement: Supplementary file 4 [file Data_Sheet_2.ZIP › CHN_HK_2 (2).tif]

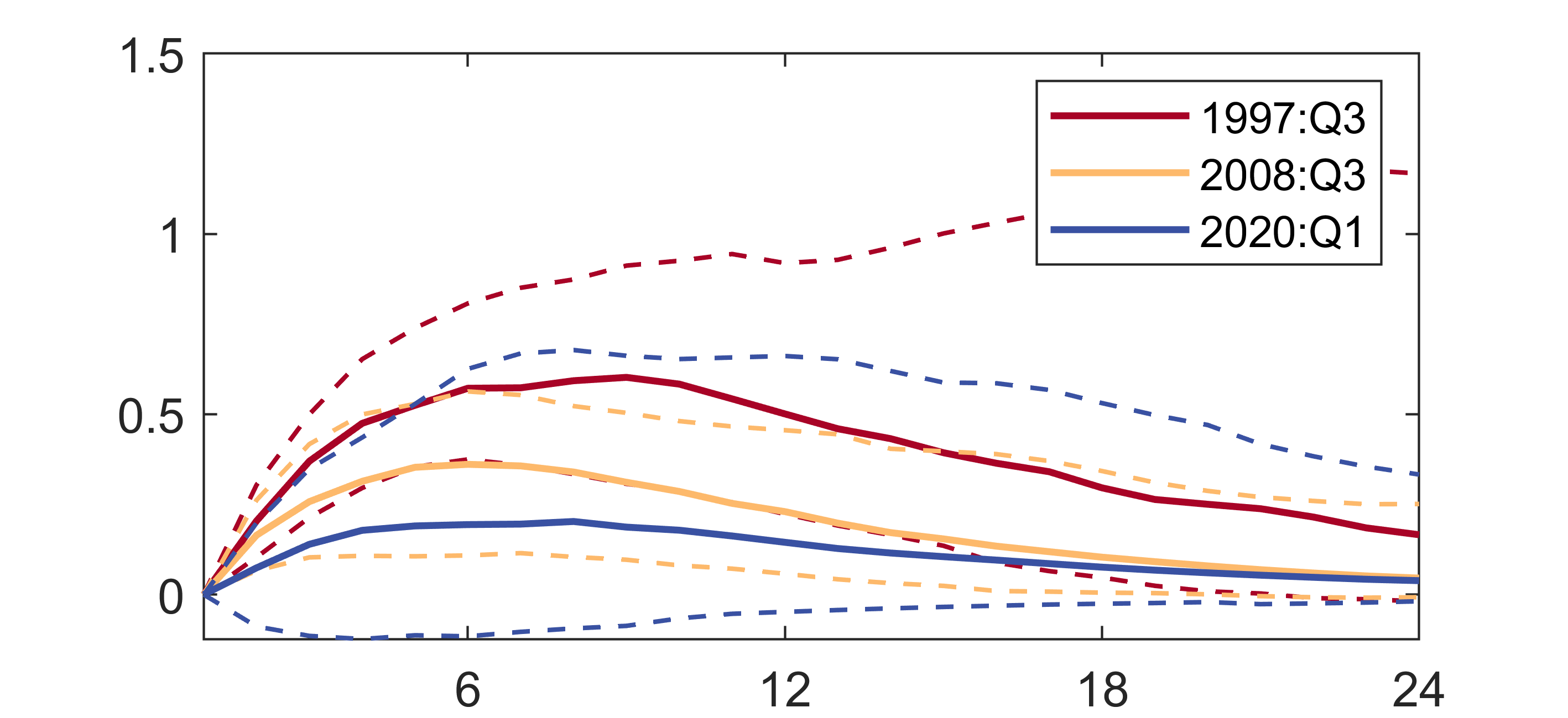

Supplement: Supplementary file 4 [file Data_Sheet_2.ZIP › CHN_HK_2 (3).tif]

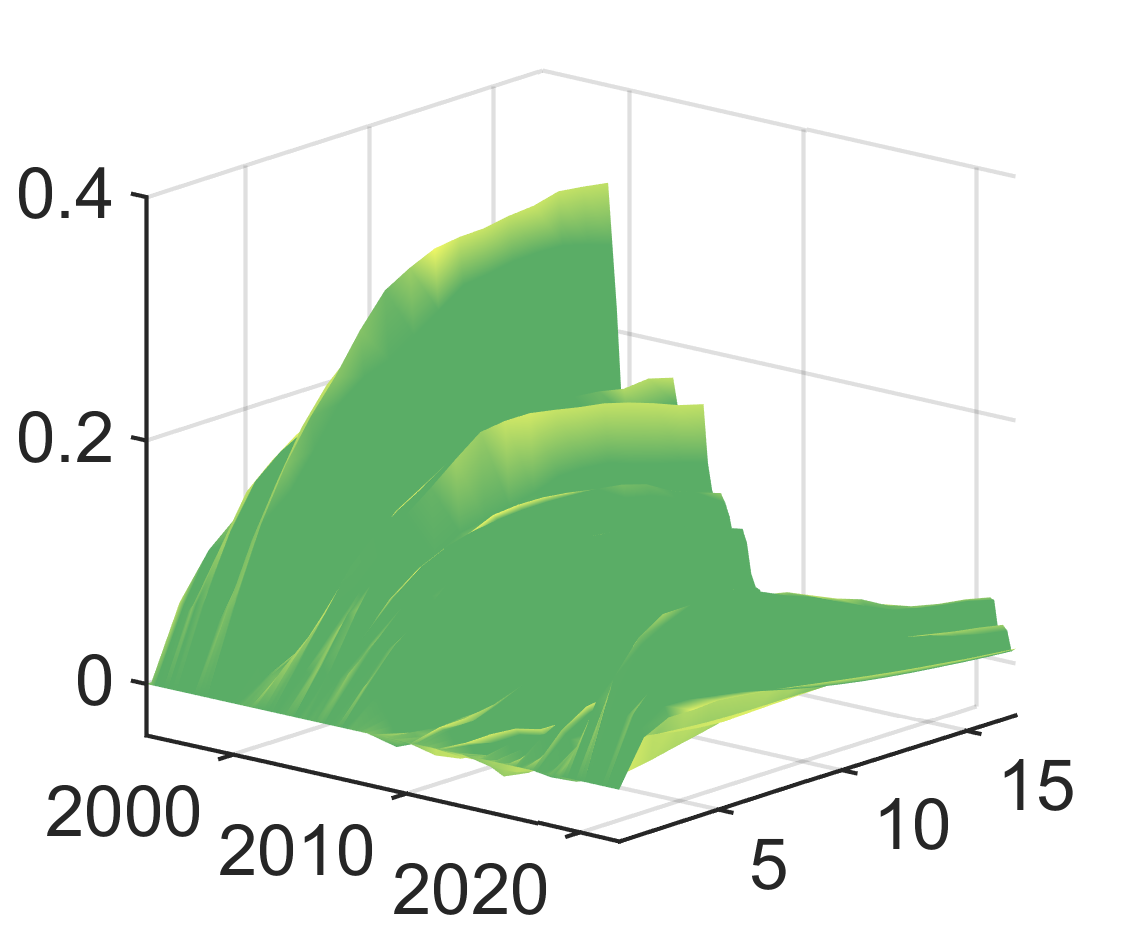

Supplement: Supplementary file 4 [file Data_Sheet_2.ZIP › CHN_JPN_2 (1).tif]

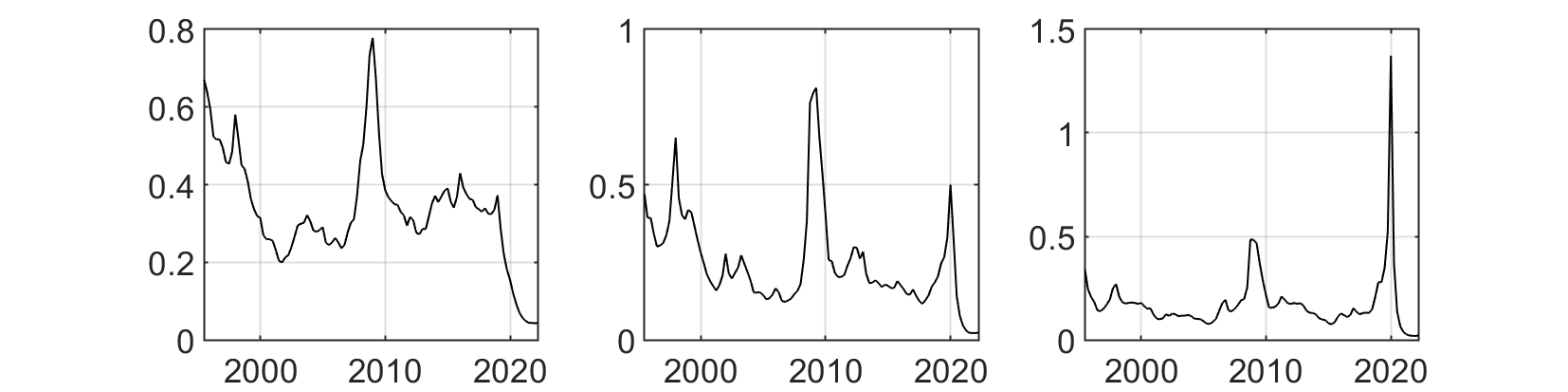

Supplement: Supplementary file 4 [file Data_Sheet_2.ZIP › CHN_JPN_2 (2).tif]

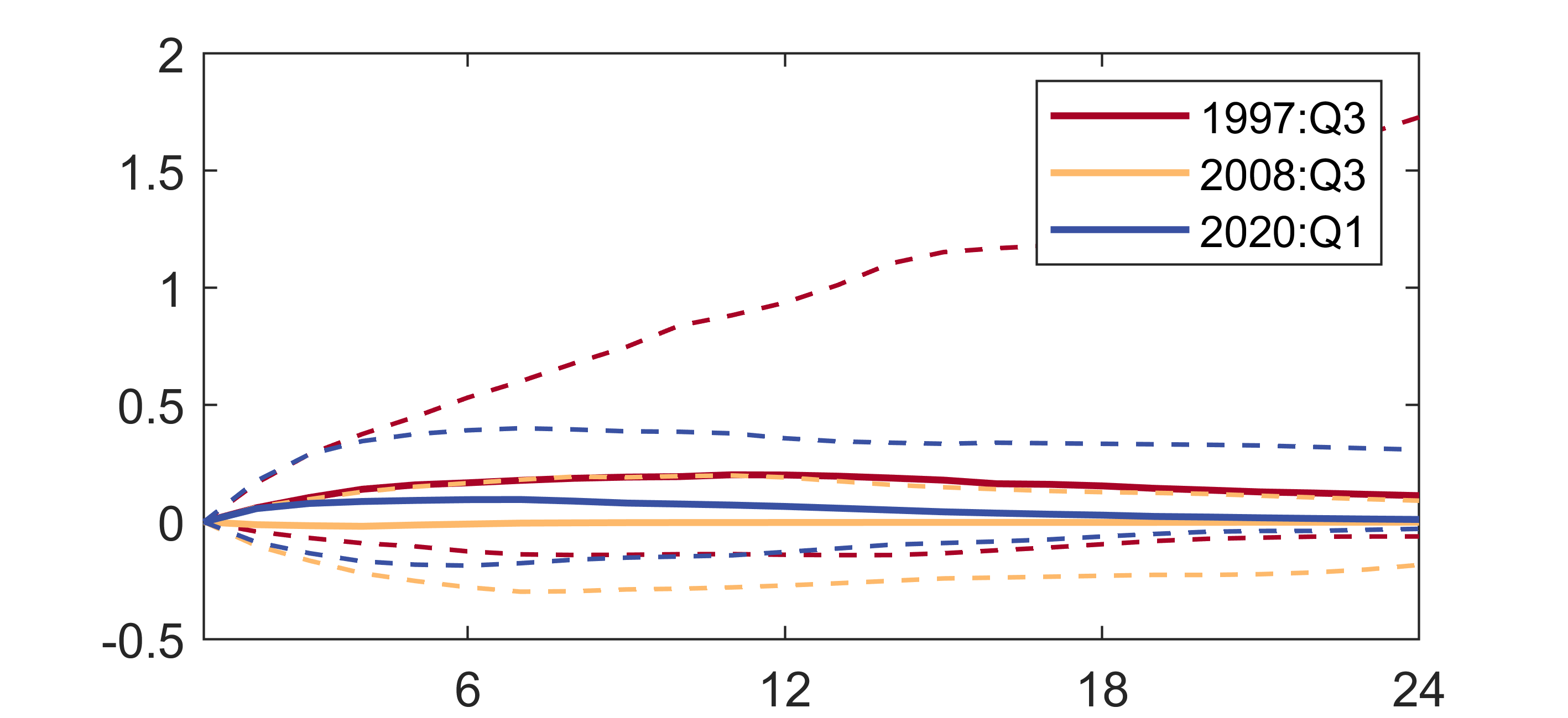

Supplement: Supplementary file 4 [file Data_Sheet_2.ZIP › CHN_JPN_2 (3).tif]
