# Supplementary figures and images for: Research on the dynamic spillover of stock markets under COVID-19—Taking the stock markets of China, Japan, and South Korea as an example (part 2 of 3)
Source: Front Public Health. 2022 Nov 11;10:1008348. doi: 10.3389/fpubh.2022.1008348 (PMC9691647; doi:10.3389/fpubh.2022.1008348)

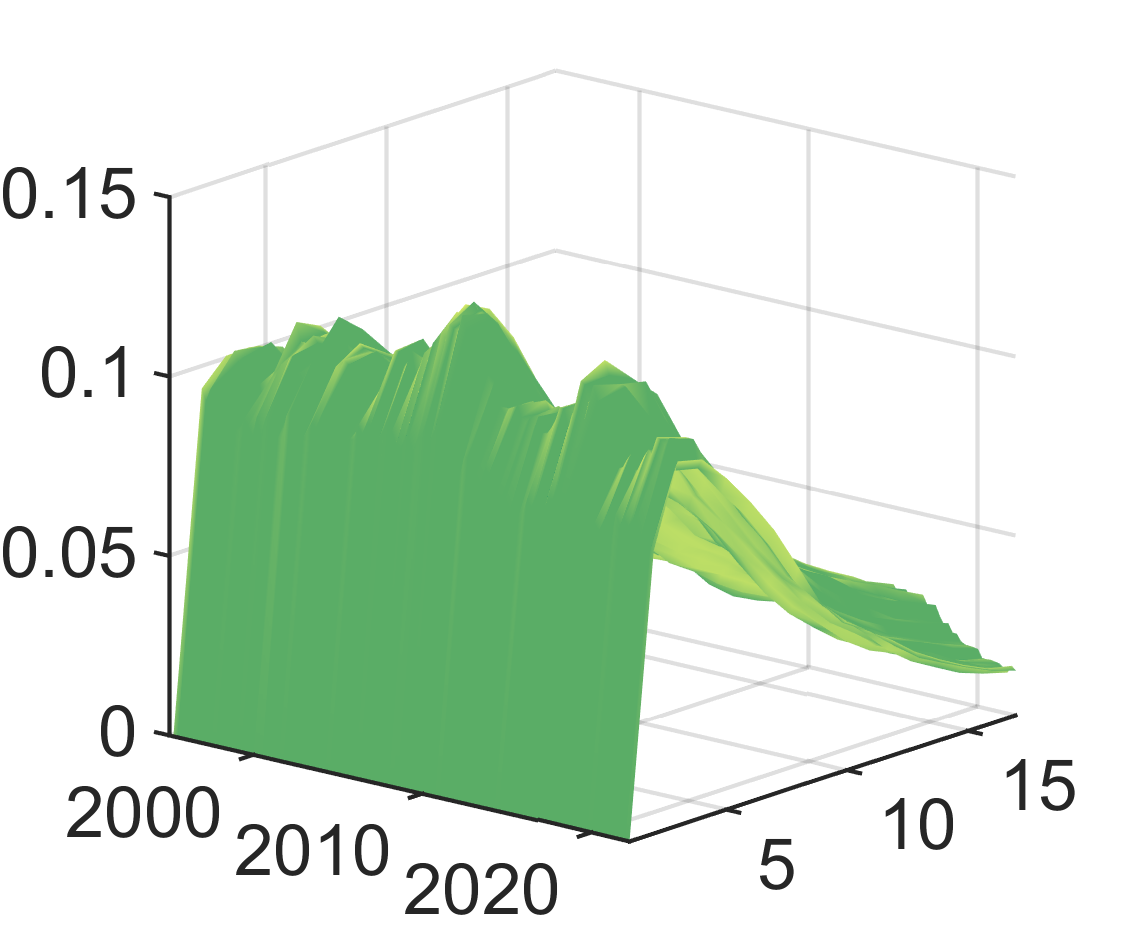

Supplement: Supplementary file 4 [file Data_Sheet_2.ZIP › CHN_KR_2 (1).tif]

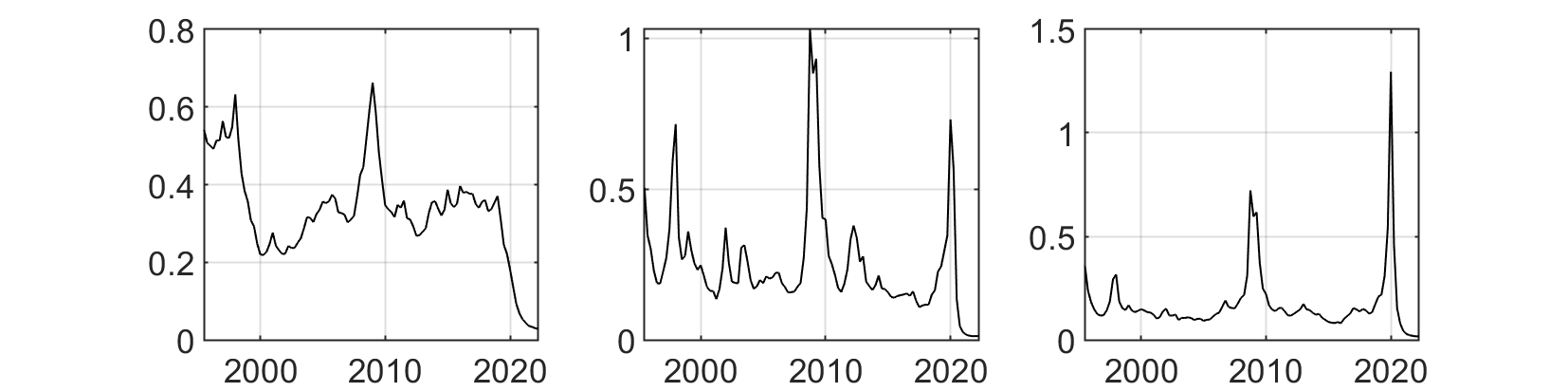

Supplement: Supplementary file 4 [file Data_Sheet_2.ZIP › CHN_KR_2 (2).tif]

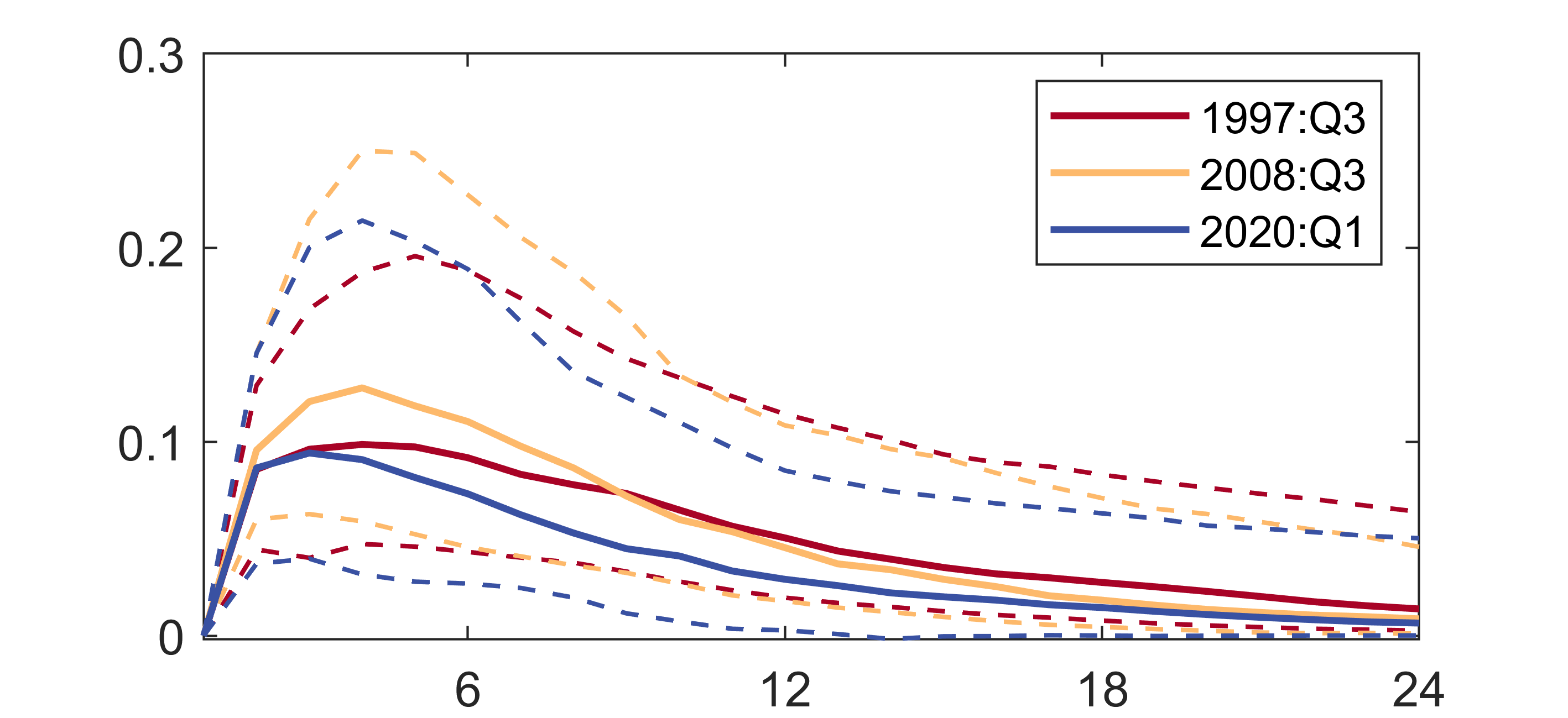

Supplement: Supplementary file 4 [file Data_Sheet_2.ZIP › CHN_KR_2 (3).tif]

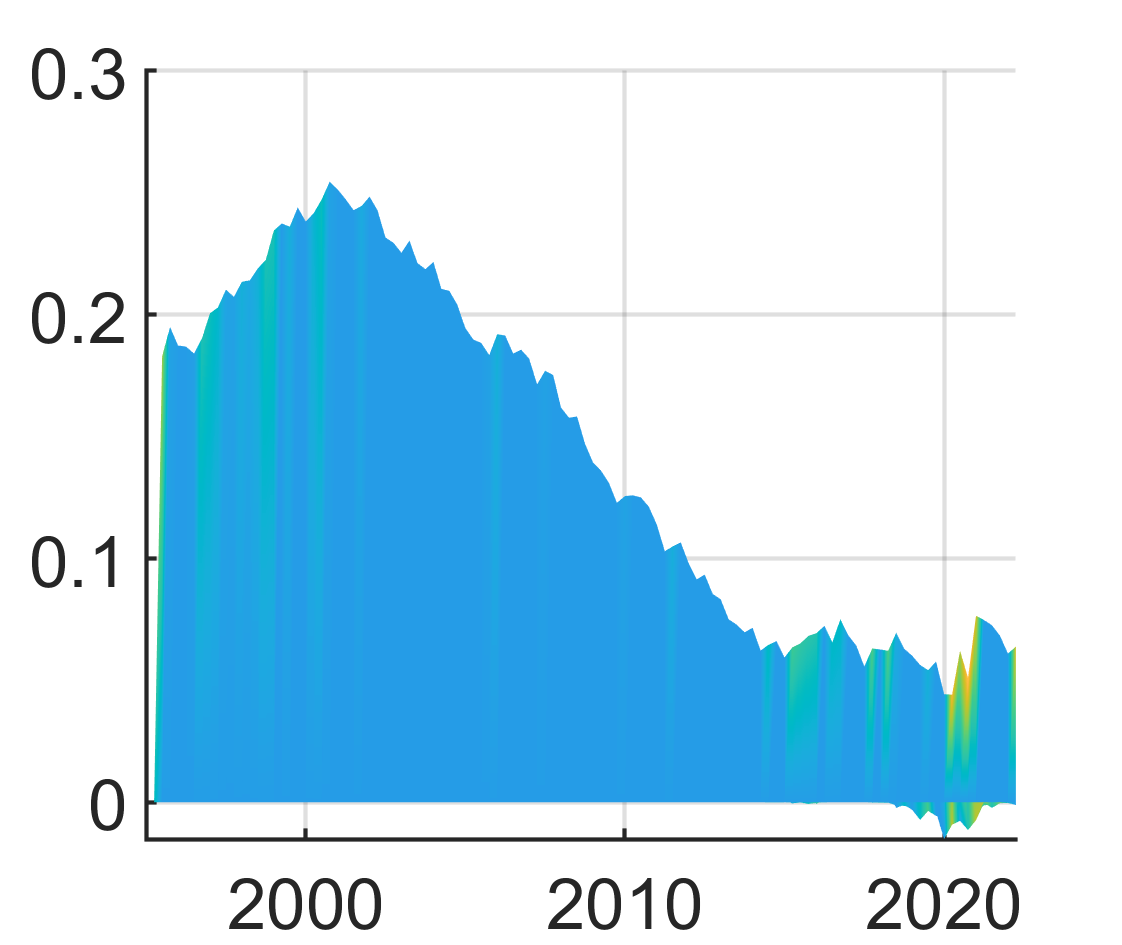

Supplement: Supplementary file 4 [file Data_Sheet_2.ZIP › CM_CHN_2 (1).tif]

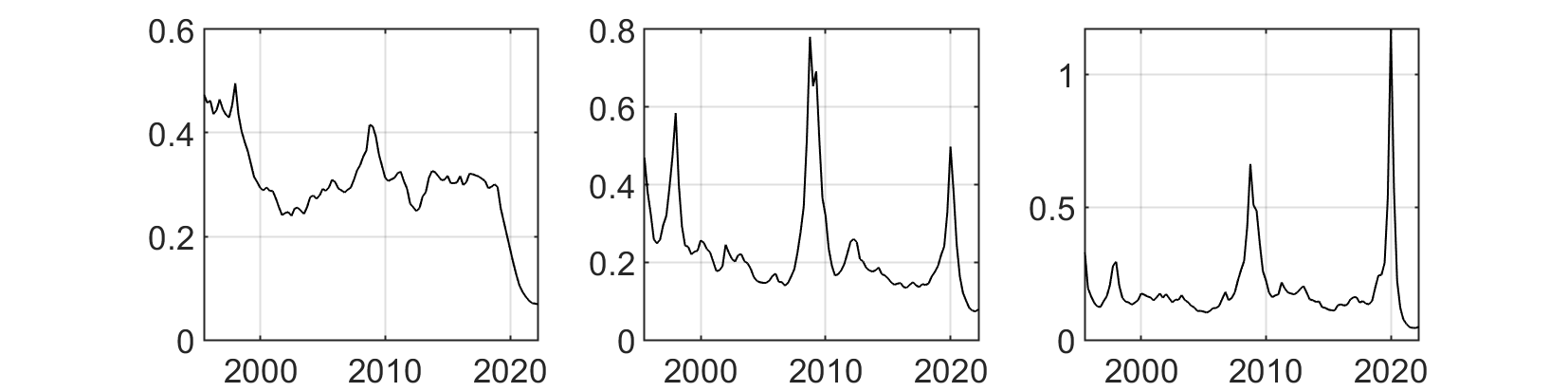

Supplement: Supplementary file 4 [file Data_Sheet_2.ZIP › CM_CHN_2 (2).tif]

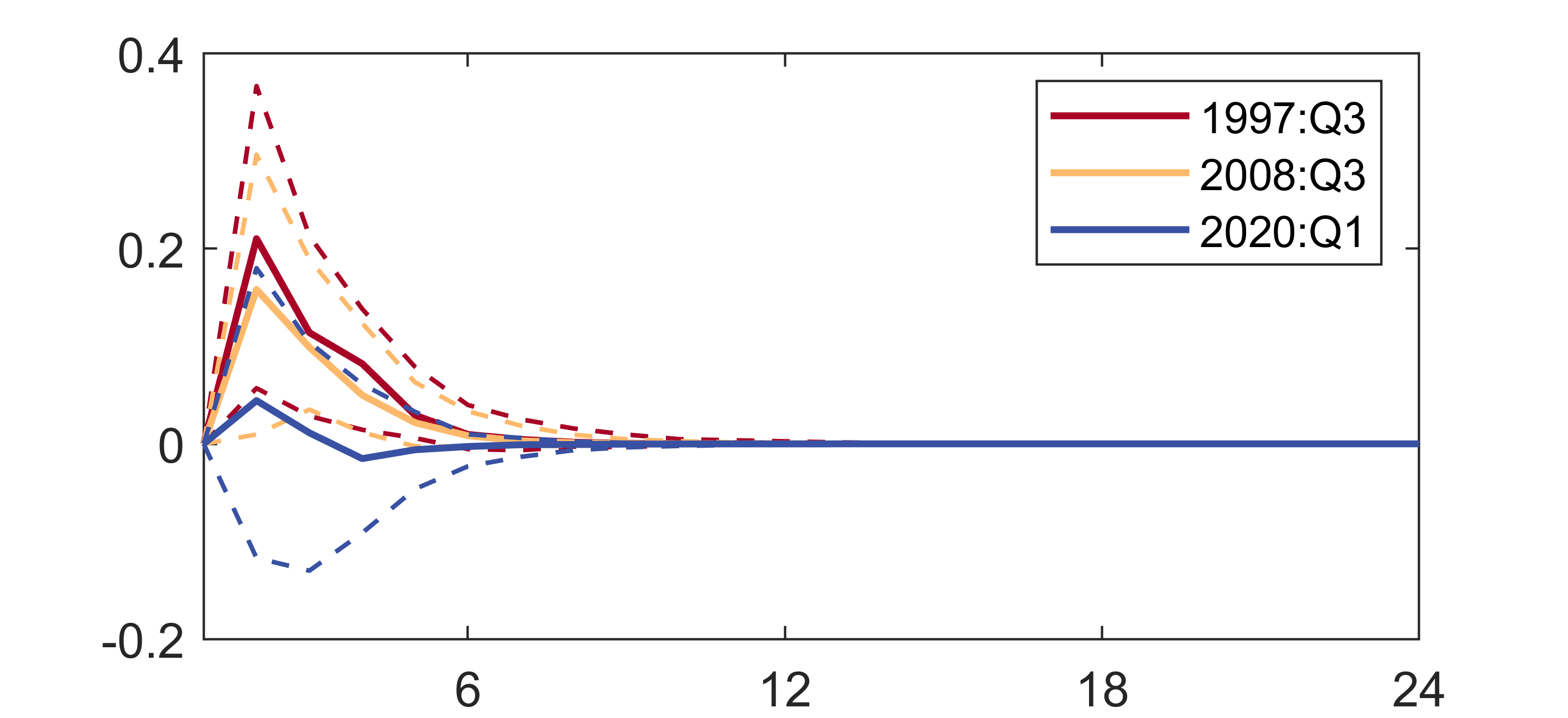

Supplement: Supplementary file 4 [file Data_Sheet_2.ZIP › CM_CHN_2 (3).tif]

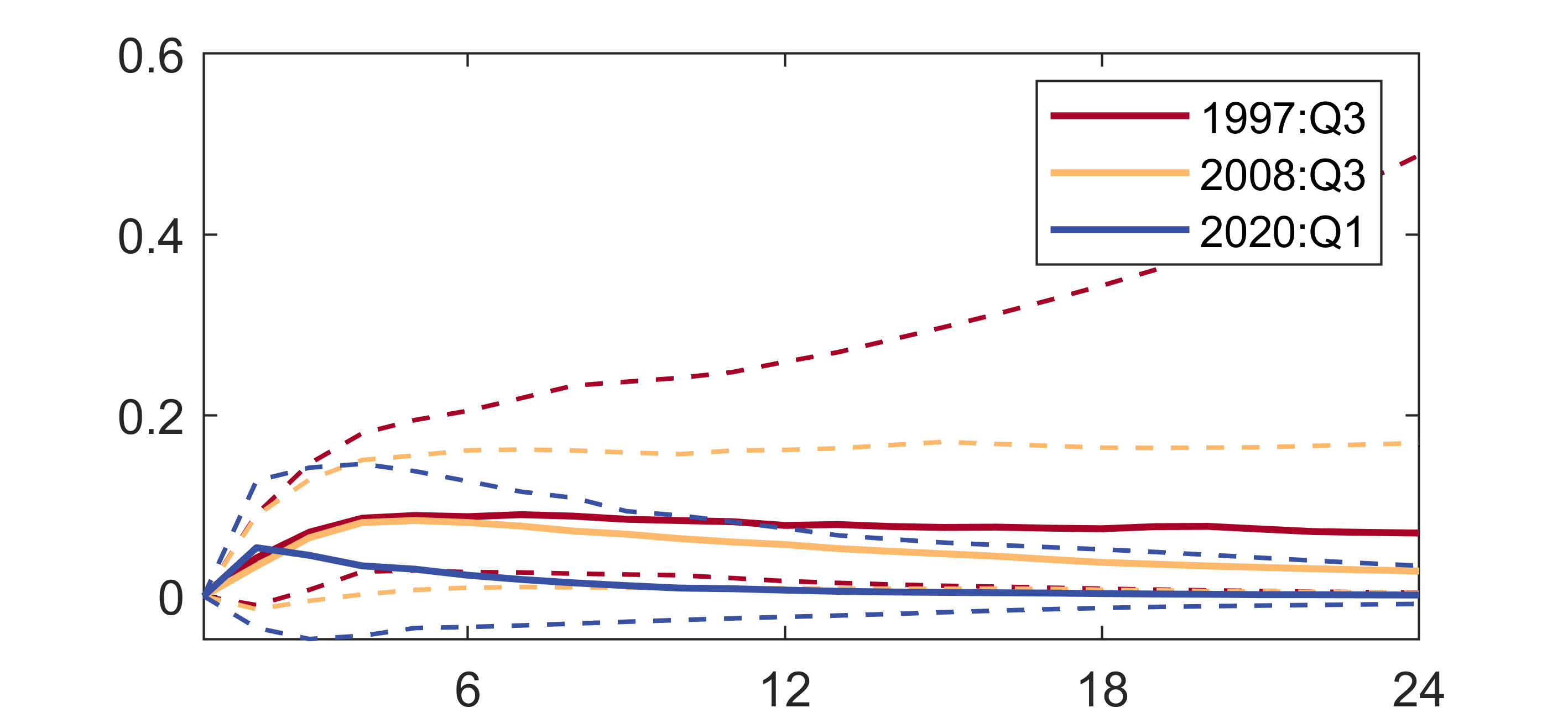

Supplement: Supplementary file 4 [file Data_Sheet_2.ZIP › CM_HK_2 (1).tif]

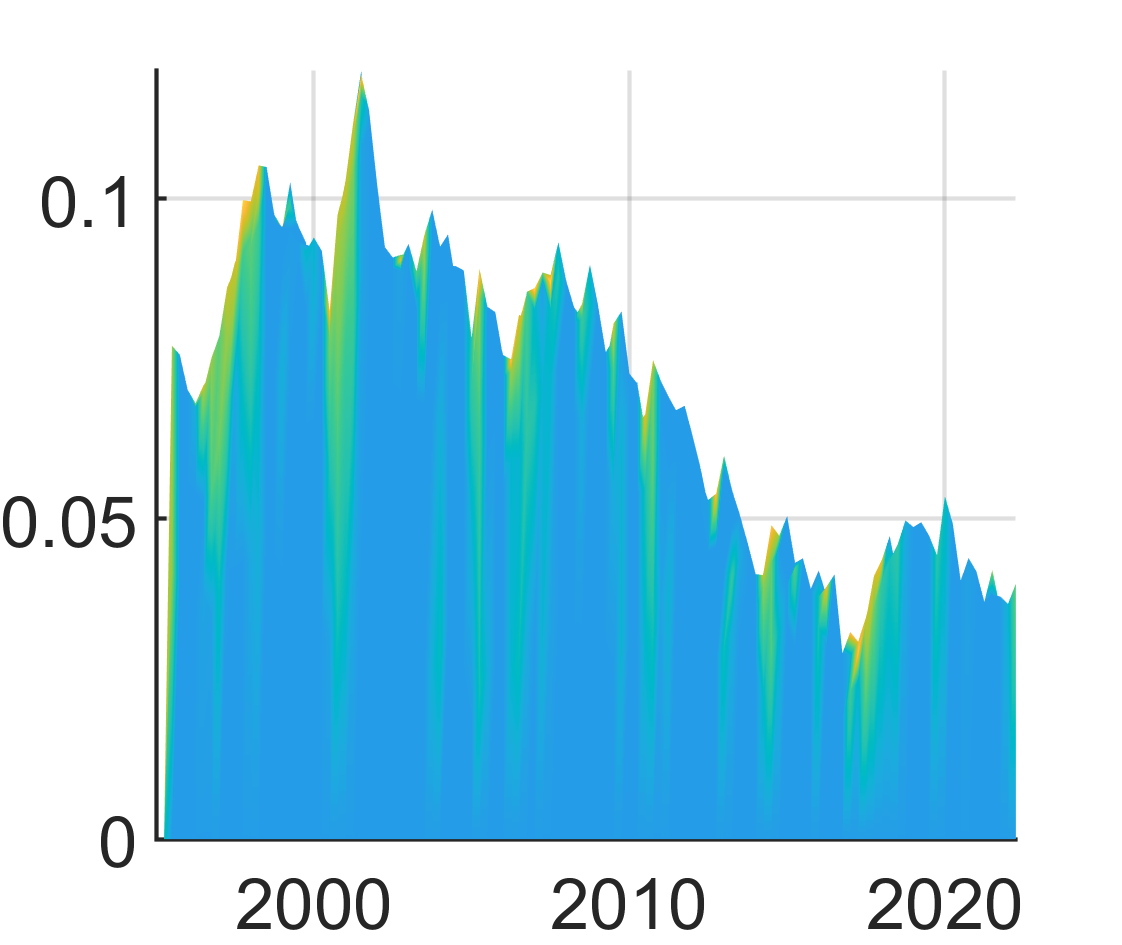

Supplement: Supplementary file 4 [file Data_Sheet_2.ZIP › CM_HK_2 (2).tif]

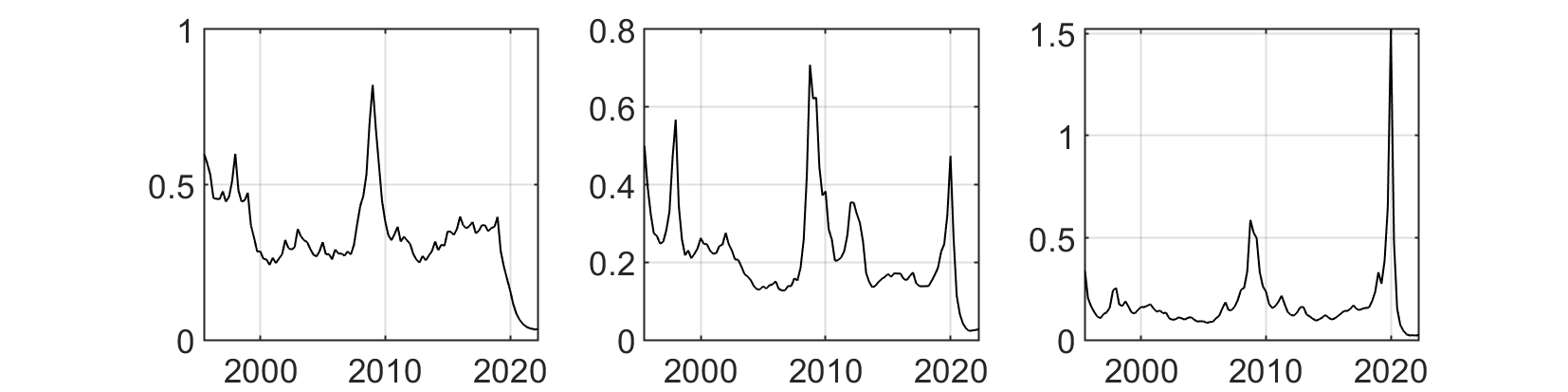

Supplement: Supplementary file 4 [file Data_Sheet_2.ZIP › CM_HK_2 (3).tif]

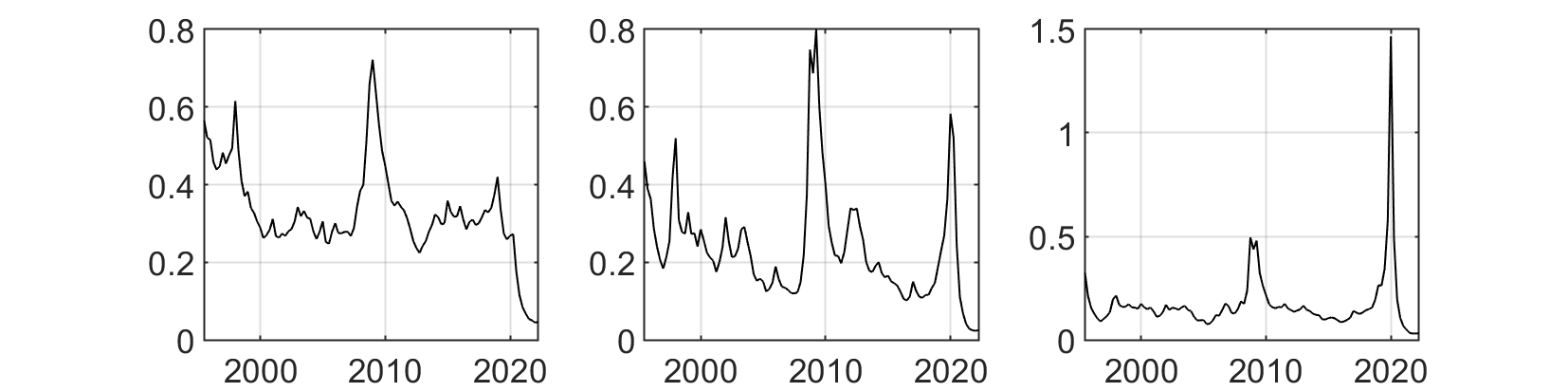

Supplement: Supplementary file 4 [file Data_Sheet_2.ZIP › CM_JPN_2 (1).tif]

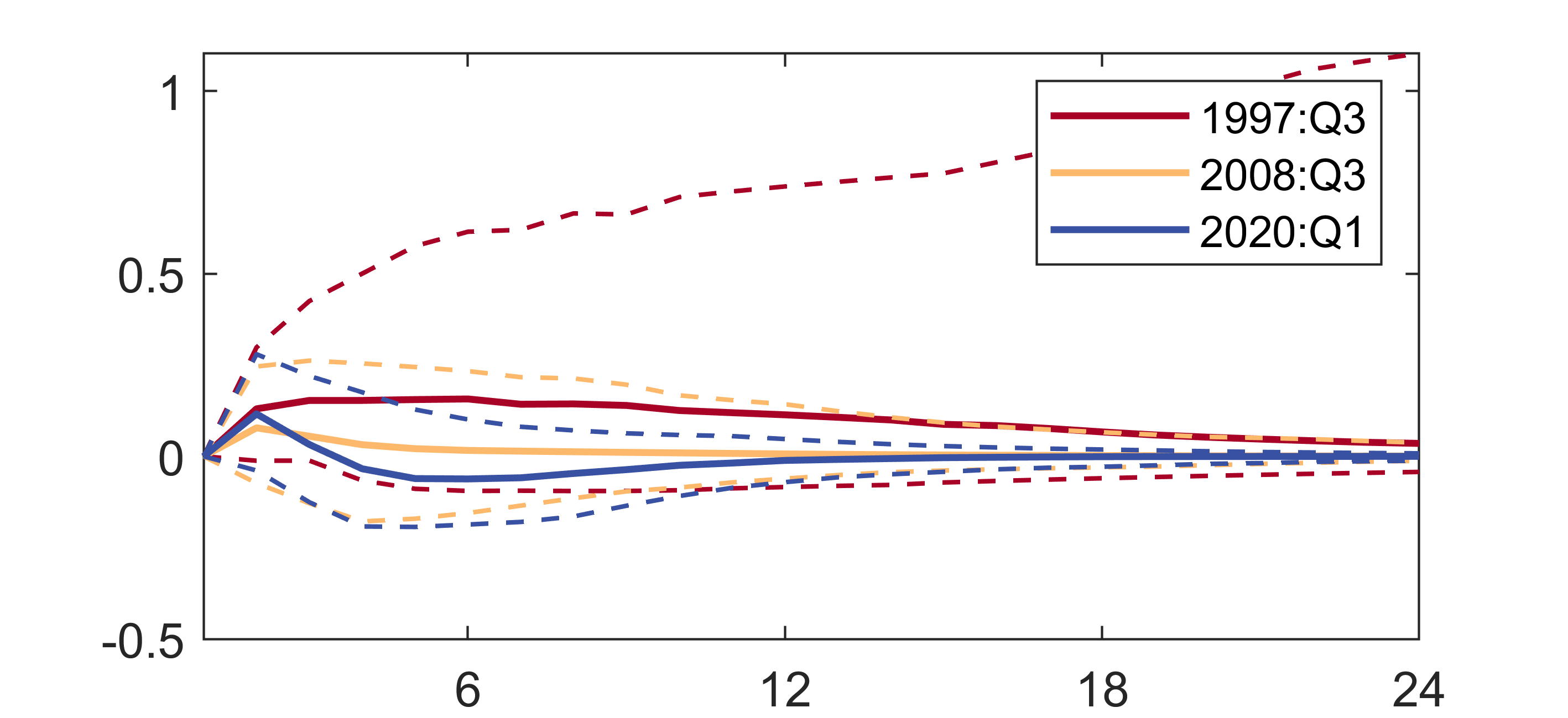

Supplement: Supplementary file 4 [file Data_Sheet_2.ZIP › CM_JPN_2 (2).tif]

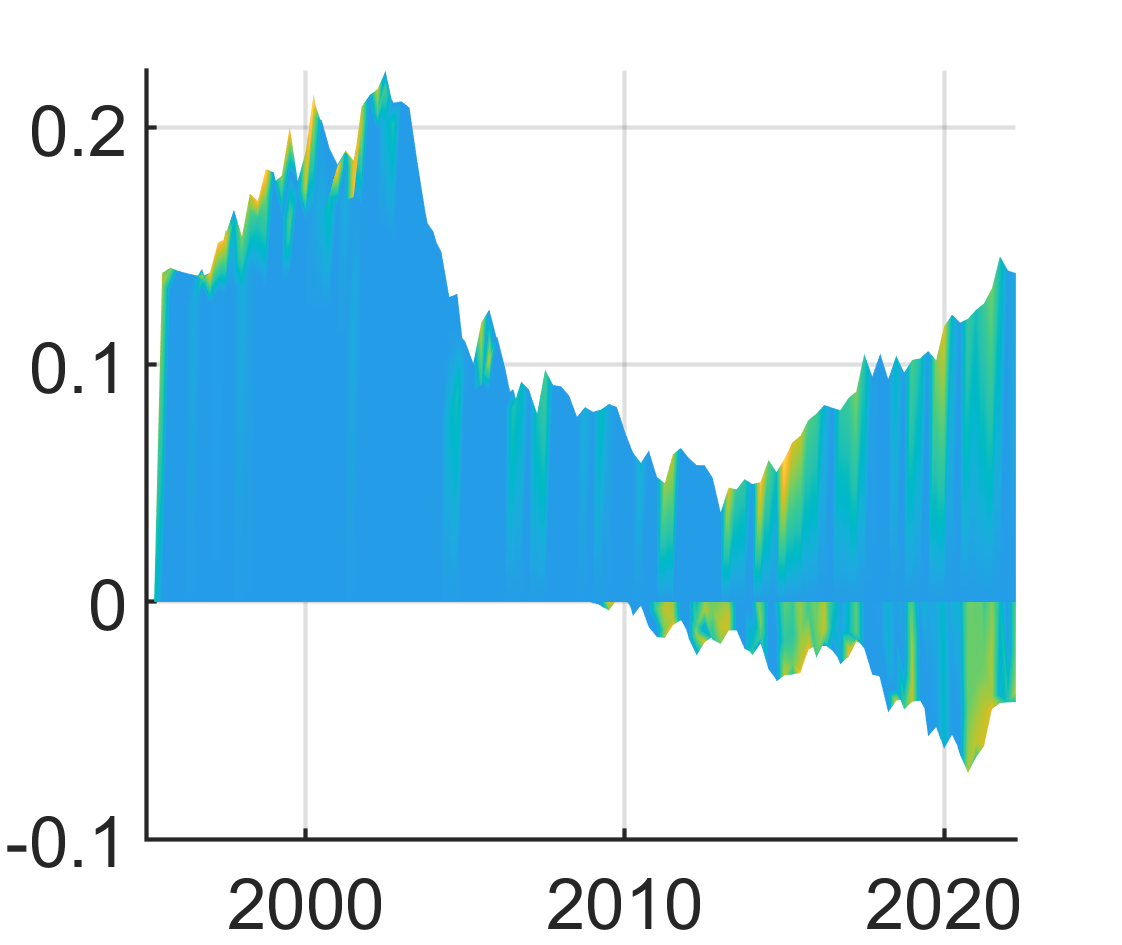

Supplement: Supplementary file 4 [file Data_Sheet_2.ZIP › CM_JPN_2 (3).tif]

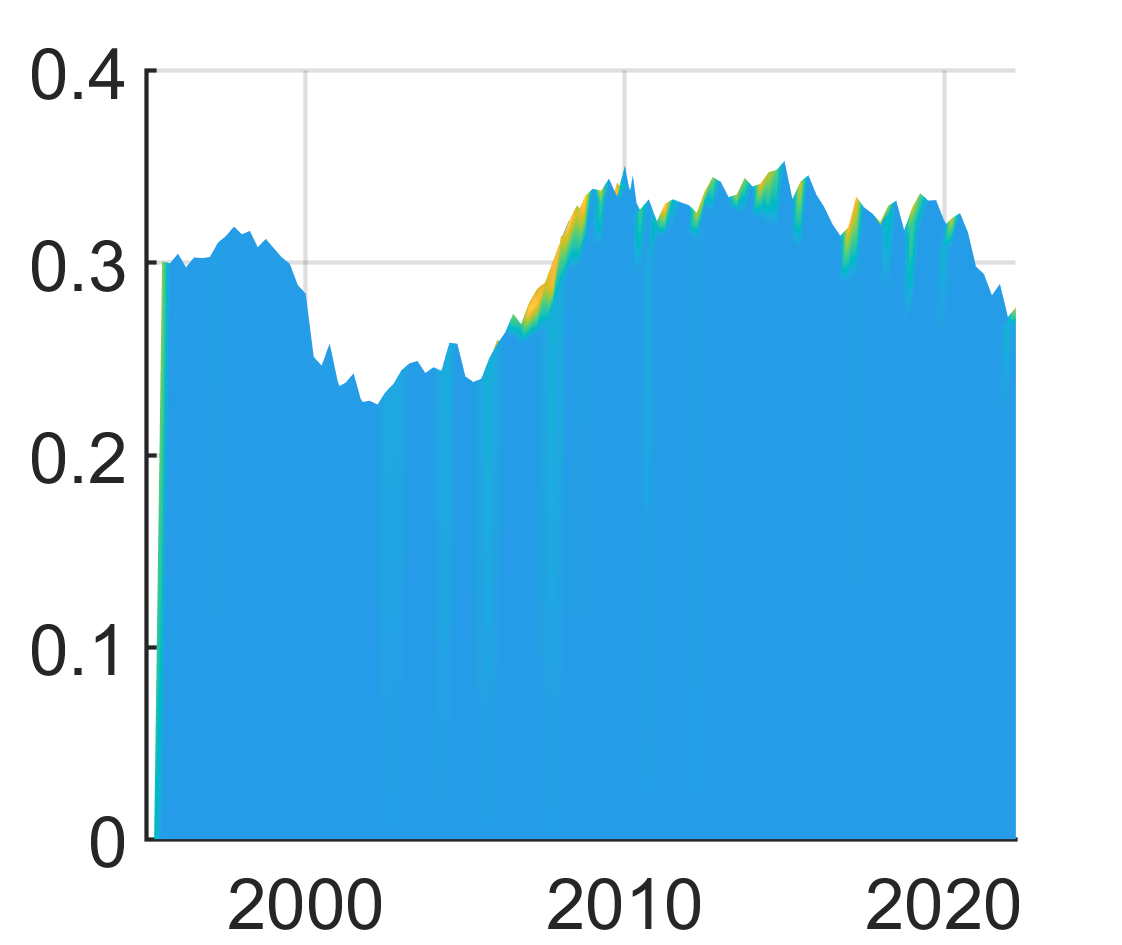

Supplement: Supplementary file 4 [file Data_Sheet_2.ZIP › CM_KR_2 (1).tif]

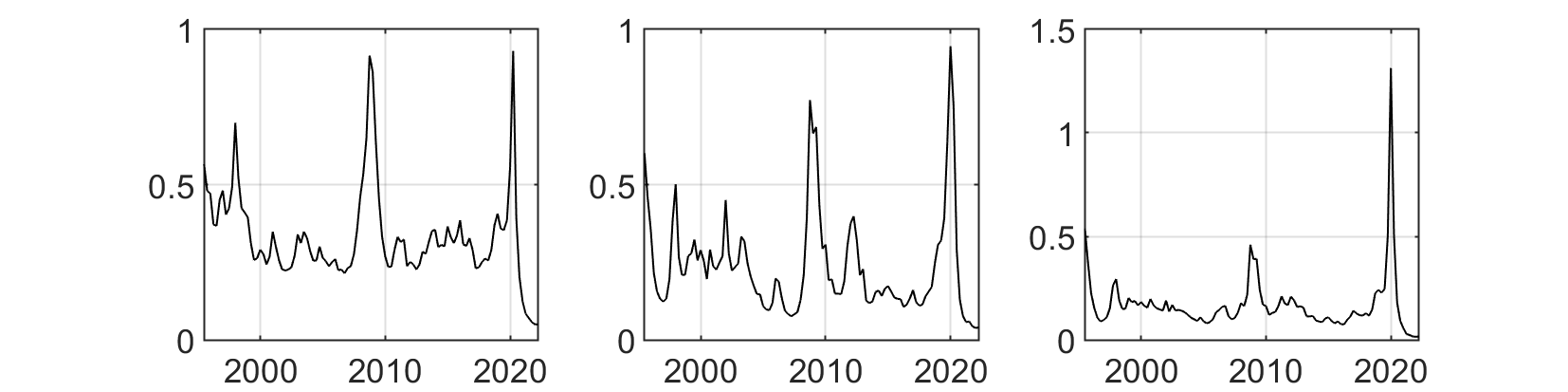

Supplement: Supplementary file 4 [file Data_Sheet_2.ZIP › CM_KR_2 (2).tif]

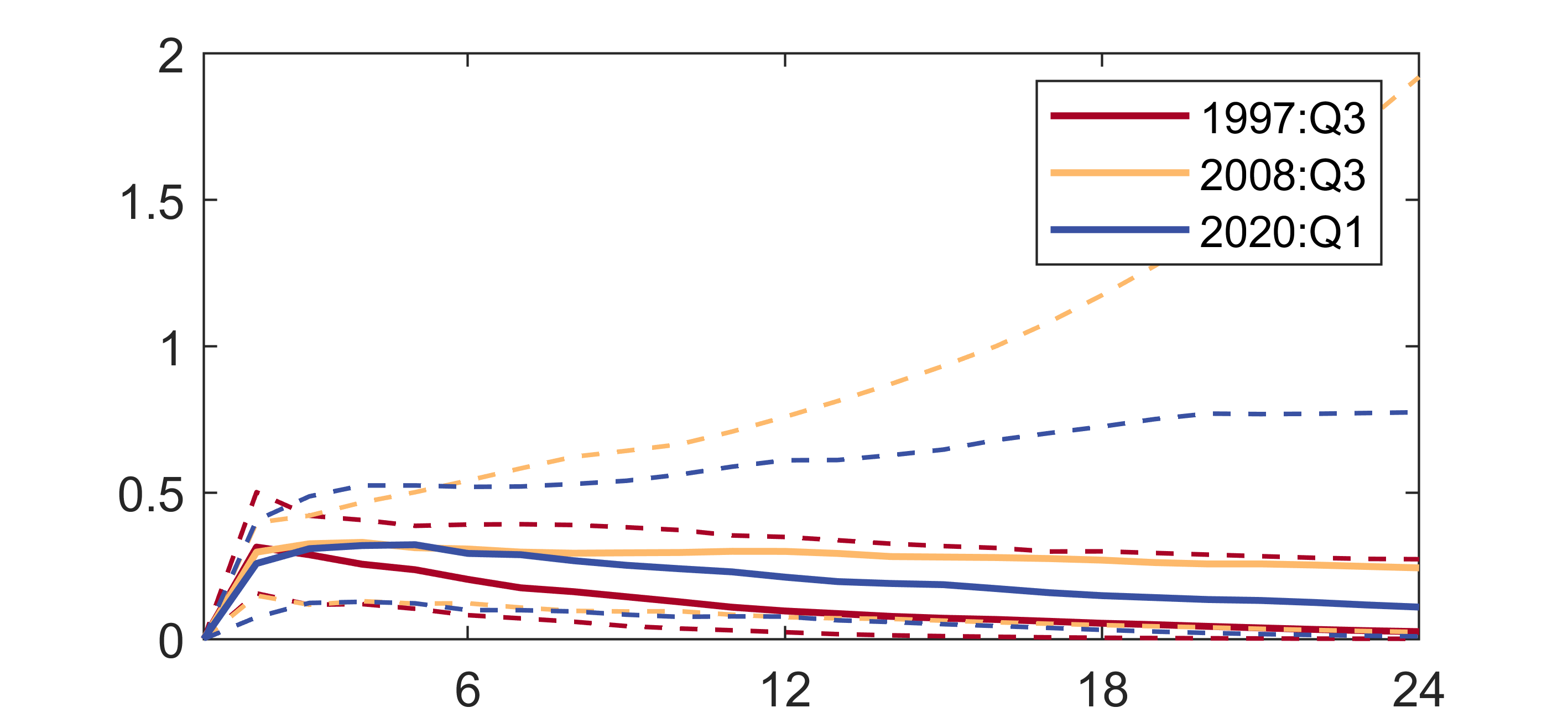

Supplement: Supplementary file 4 [file Data_Sheet_2.ZIP › CM_KR_2 (3).tif]

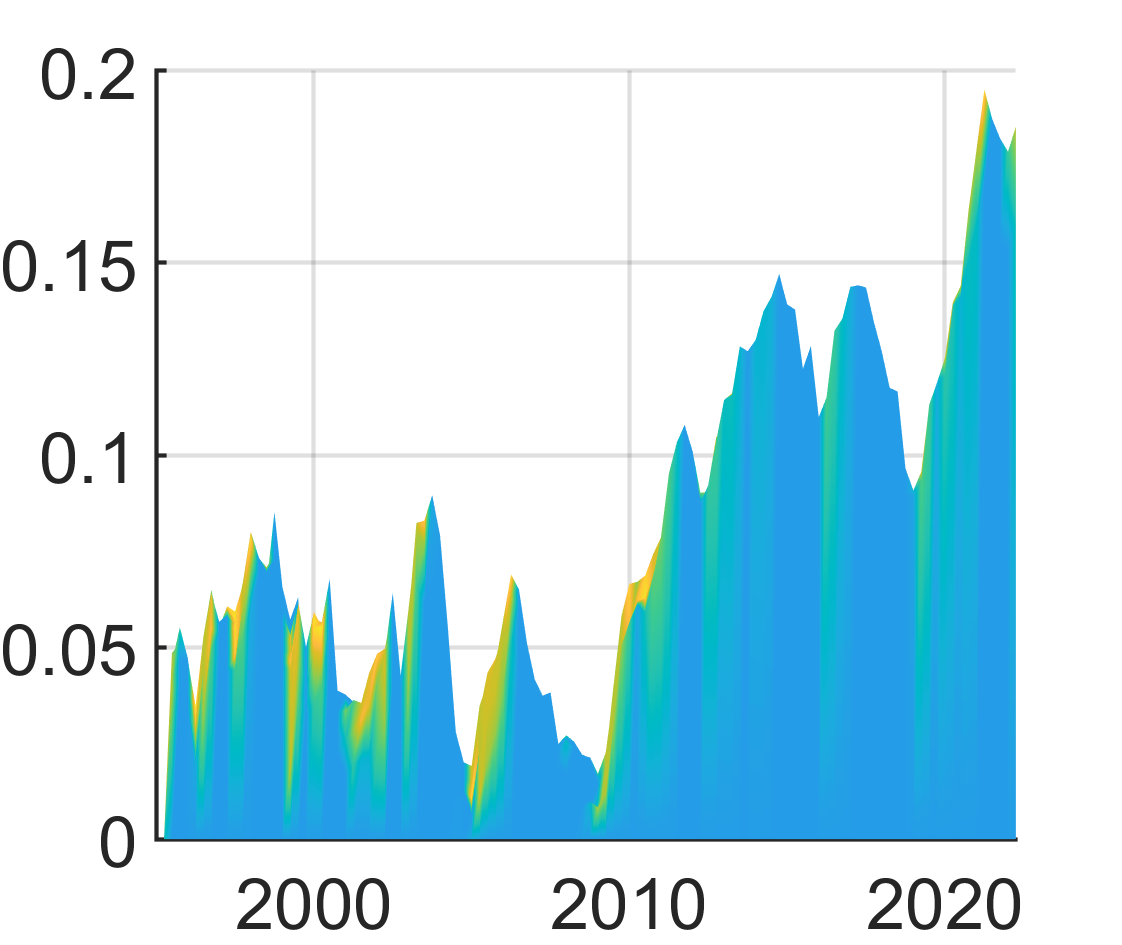

Supplement: Supplementary file 4 [file Data_Sheet_2.ZIP › COM_CHN_2 (1).tif]

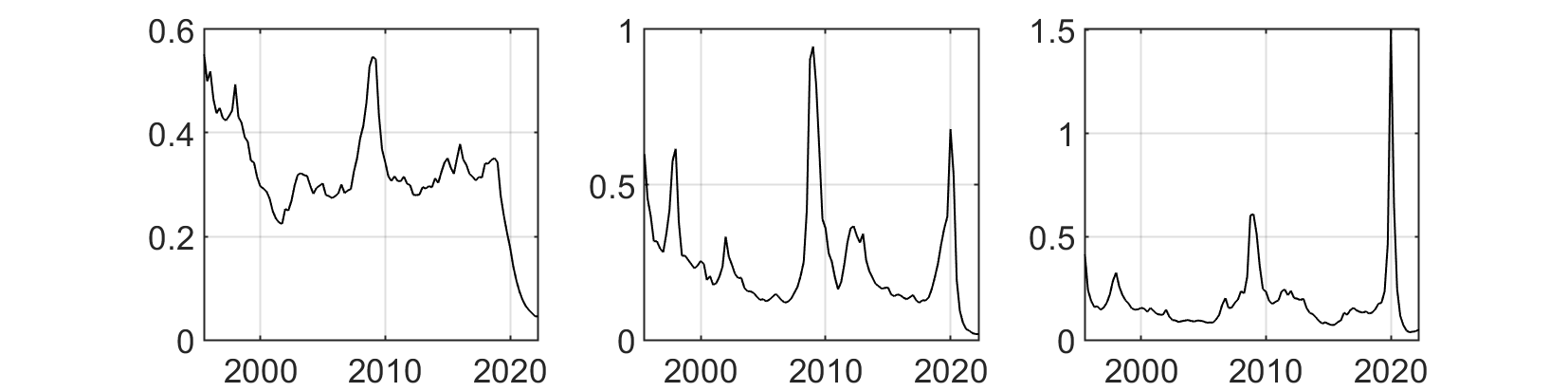

Supplement: Supplementary file 4 [file Data_Sheet_2.ZIP › COM_CHN_2 (2).tif]

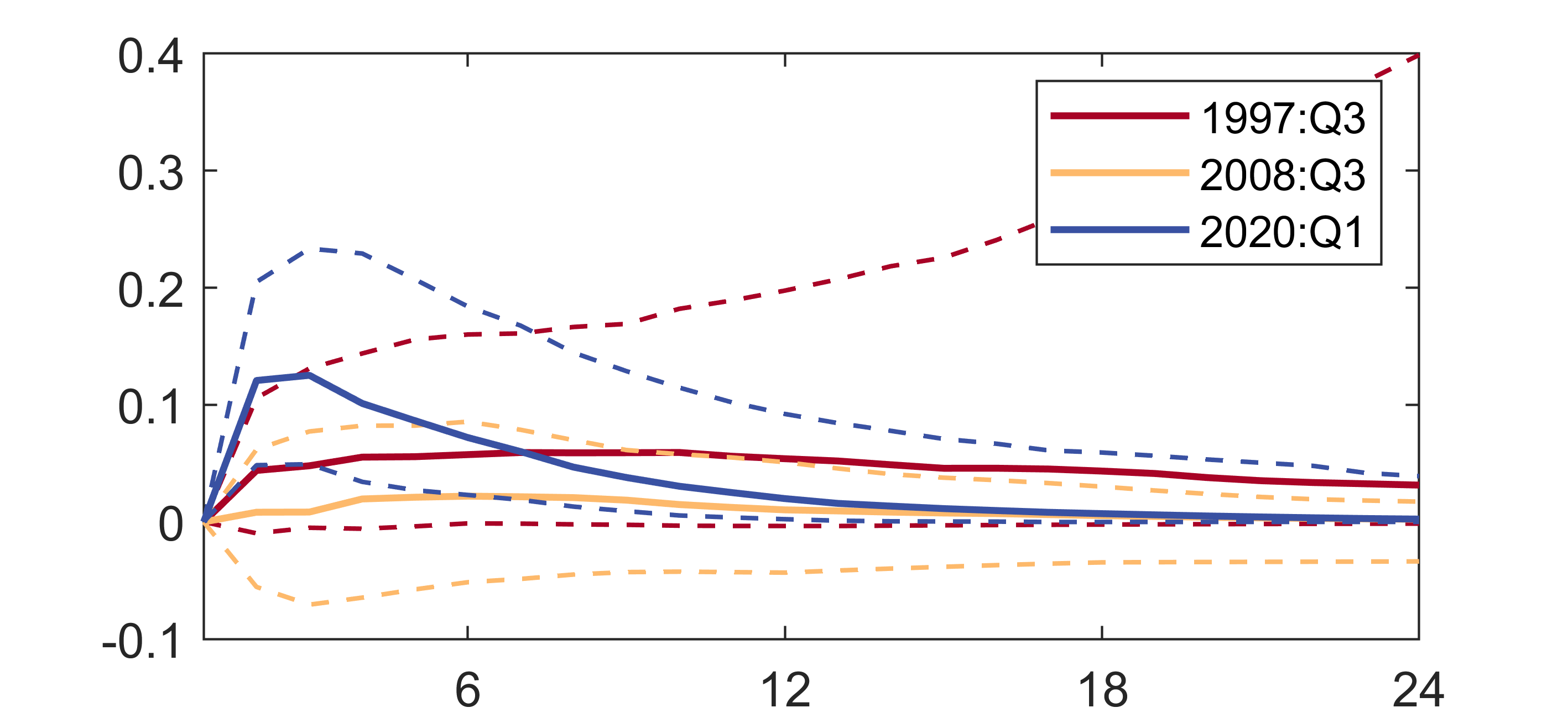

Supplement: Supplementary file 4 [file Data_Sheet_2.ZIP › COM_CHN_2 (3).tif]

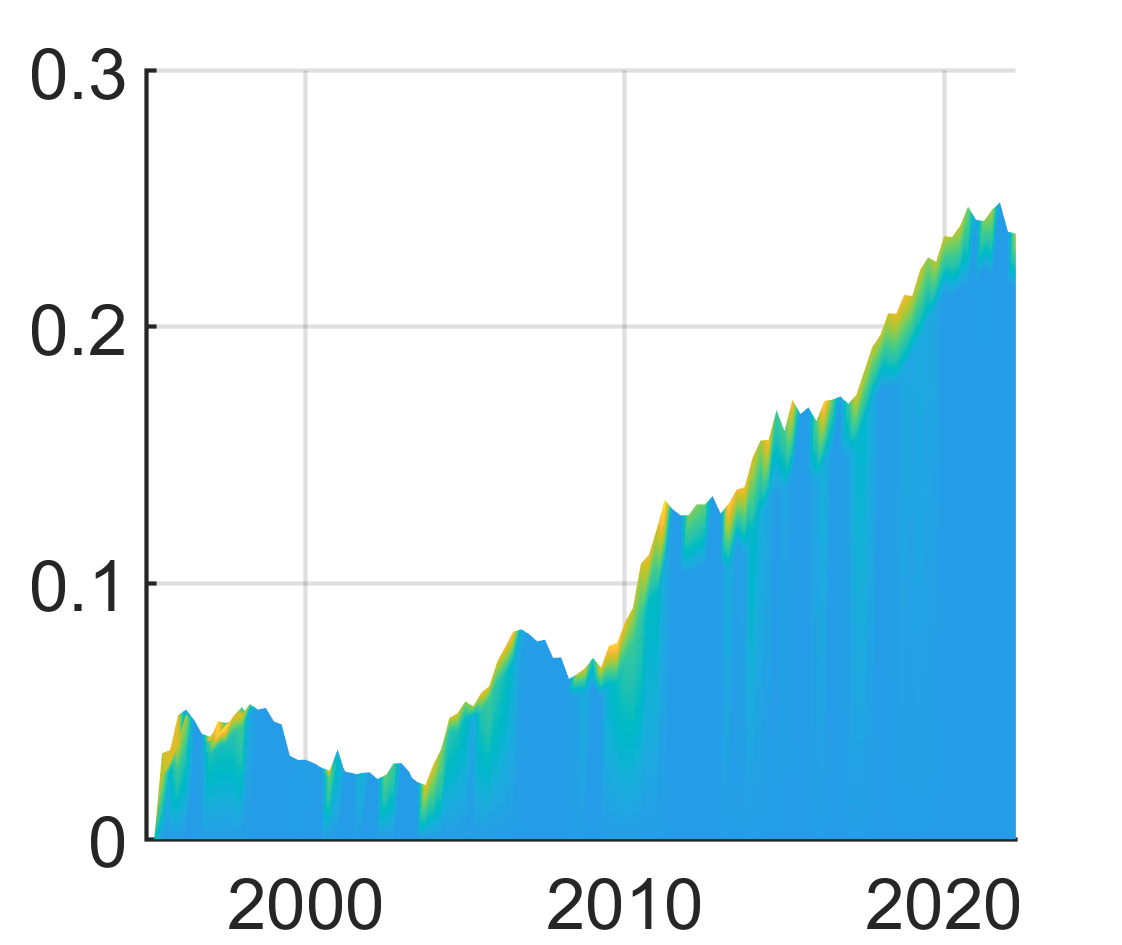

Supplement: Supplementary file 4 [file Data_Sheet_2.ZIP › COM_HK_2 (1).tif]

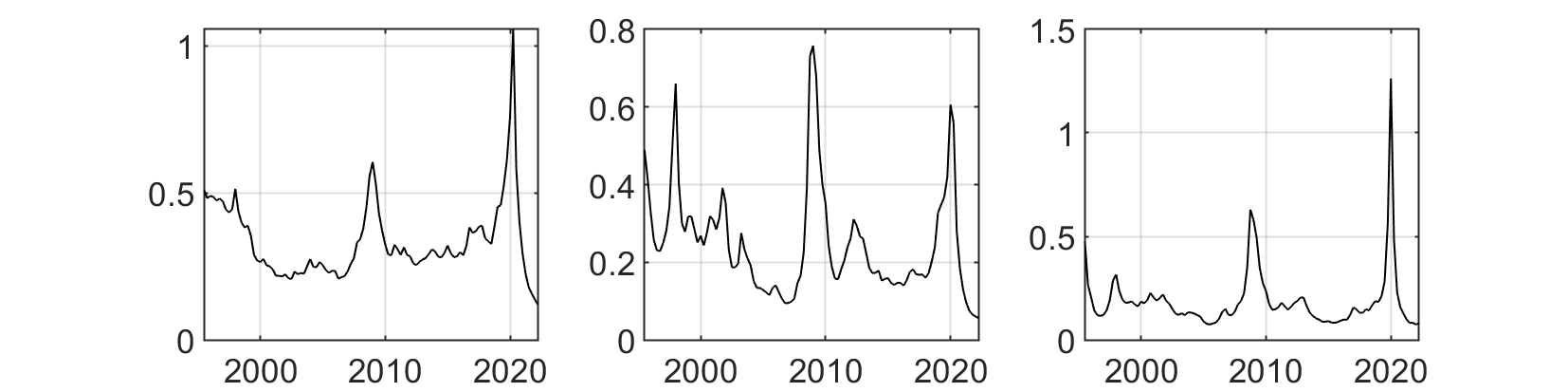

Supplement: Supplementary file 4 [file Data_Sheet_2.ZIP › COM_HK_2 (2).tif]

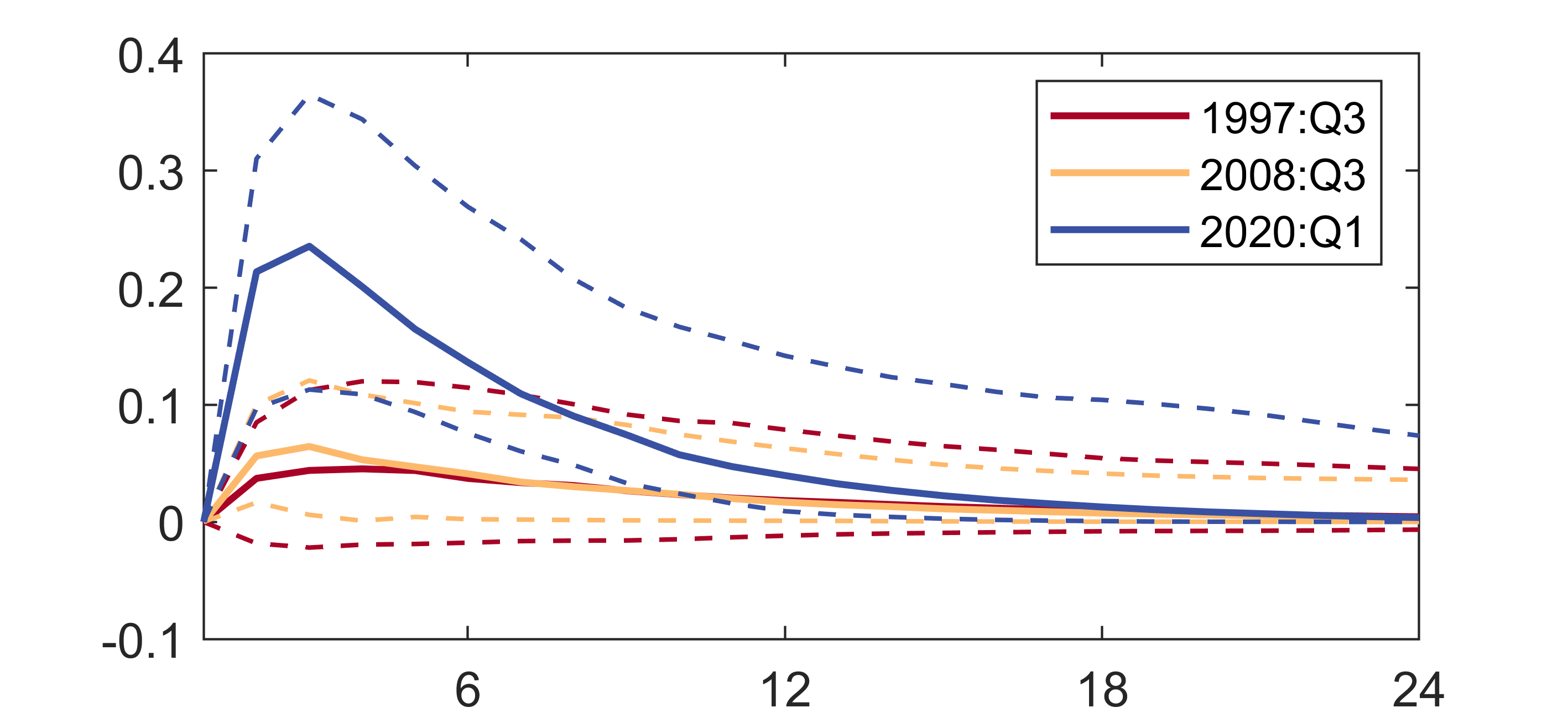

Supplement: Supplementary file 4 [file Data_Sheet_2.ZIP › COM_HK_2 (3).tif]

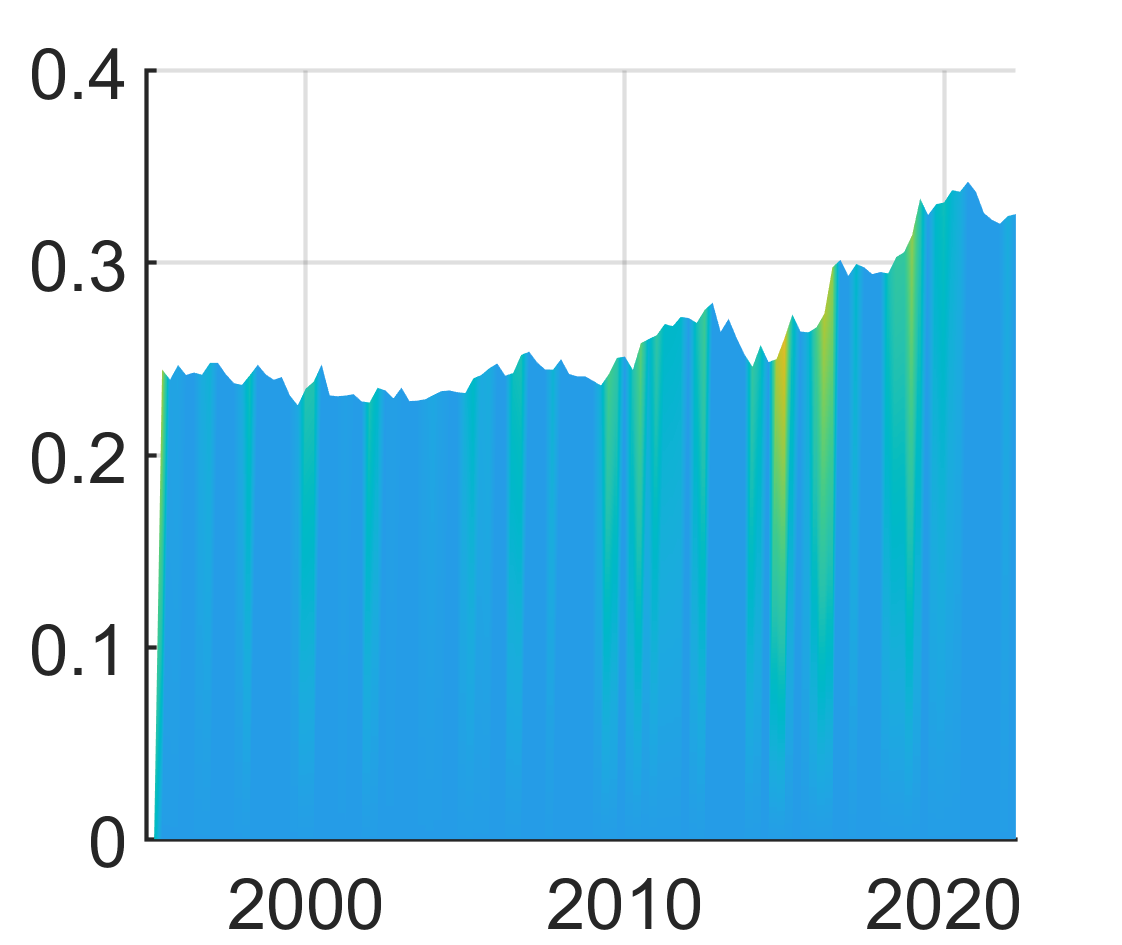

Supplement: Supplementary file 4 [file Data_Sheet_2.ZIP › COM_JPN_2 (1).tif]

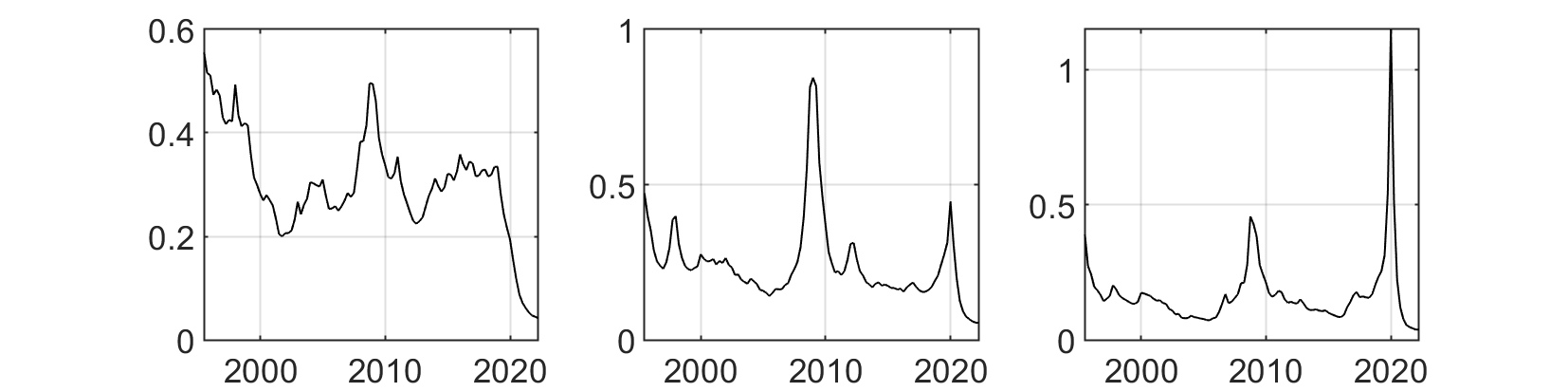

Supplement: Supplementary file 4 [file Data_Sheet_2.ZIP › COM_JPN_2 (2).tif]

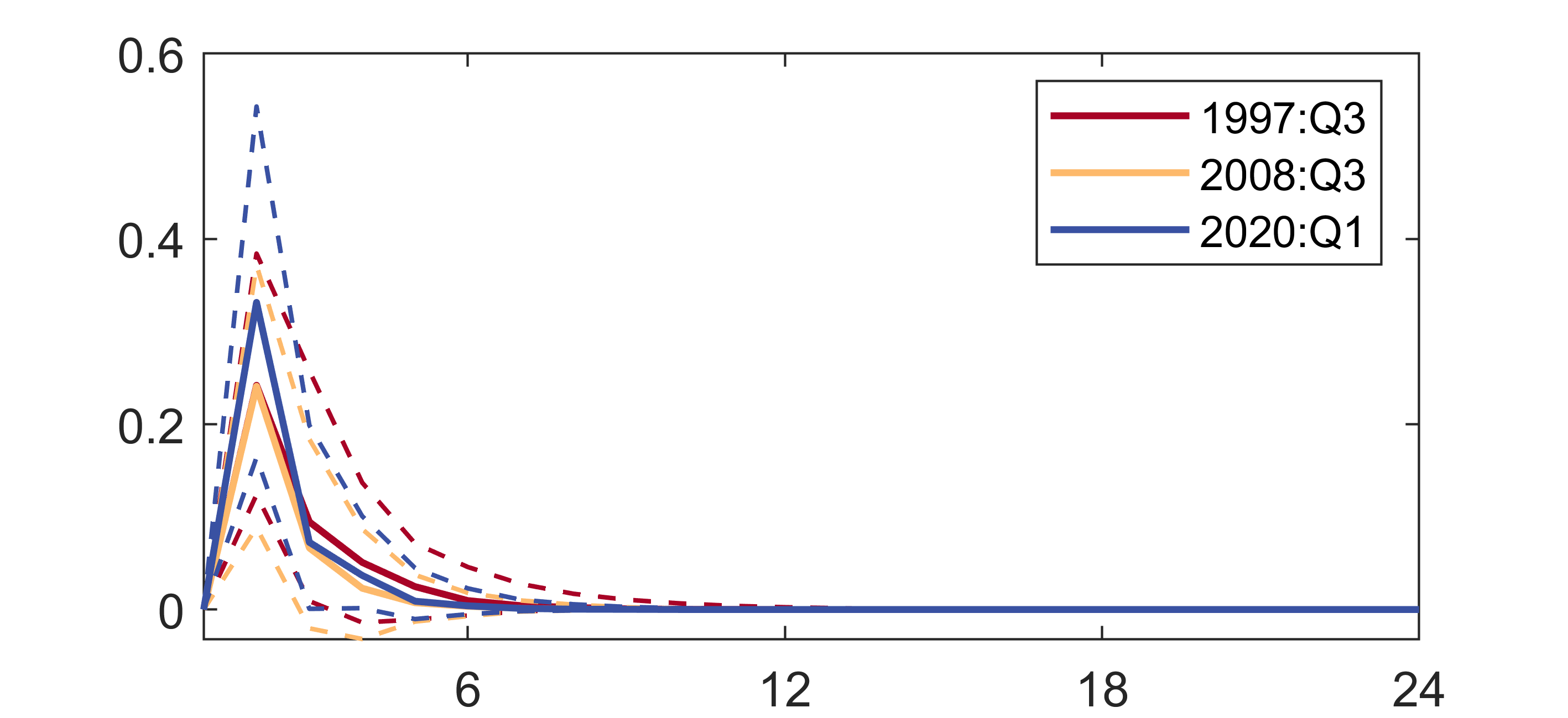

Supplement: Supplementary file 4 [file Data_Sheet_2.ZIP › COM_JPN_2 (3).tif]

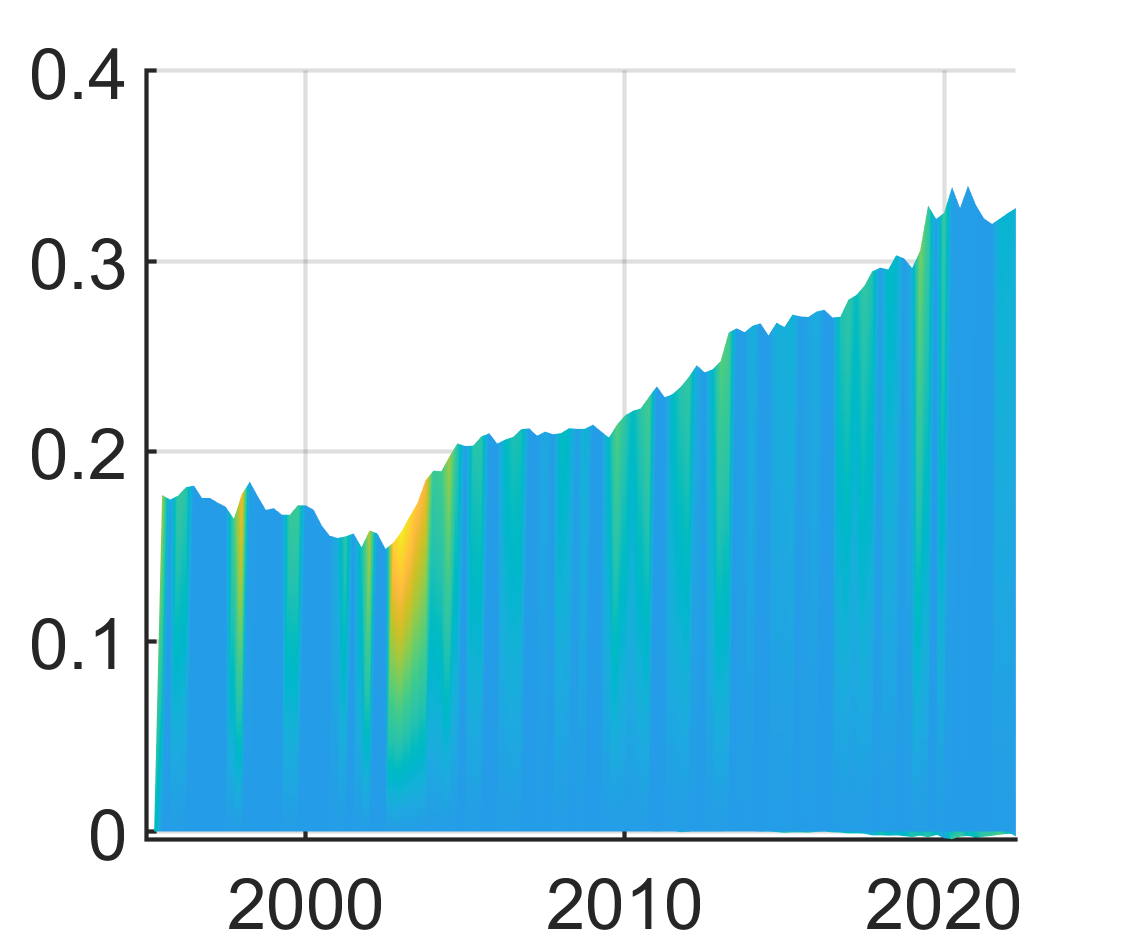

Supplement: Supplementary file 4 [file Data_Sheet_2.ZIP › COM_KR_2 (1).tif]

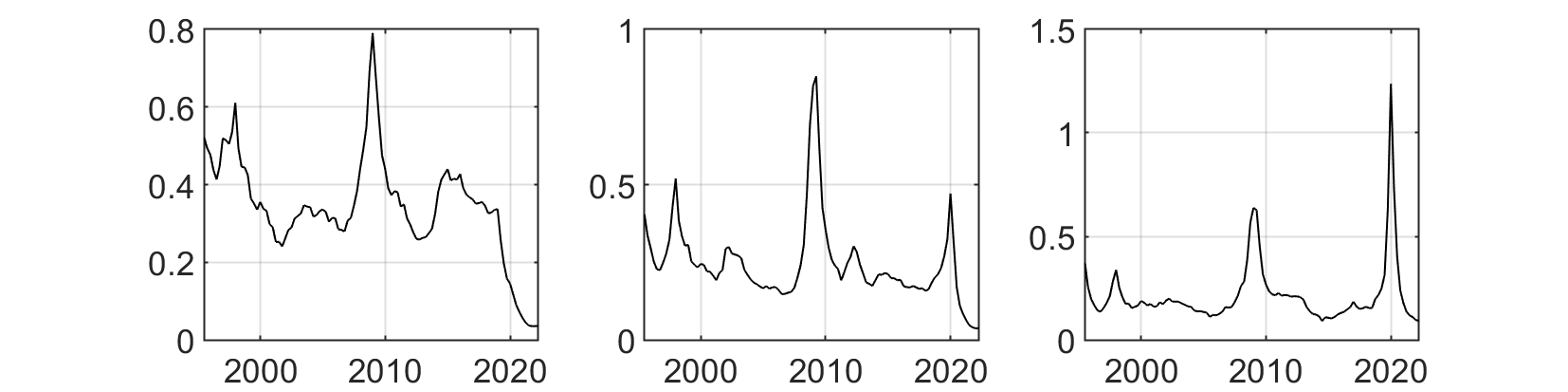

Supplement: Supplementary file 4 [file Data_Sheet_2.ZIP › COM_KR_2 (2).tif]

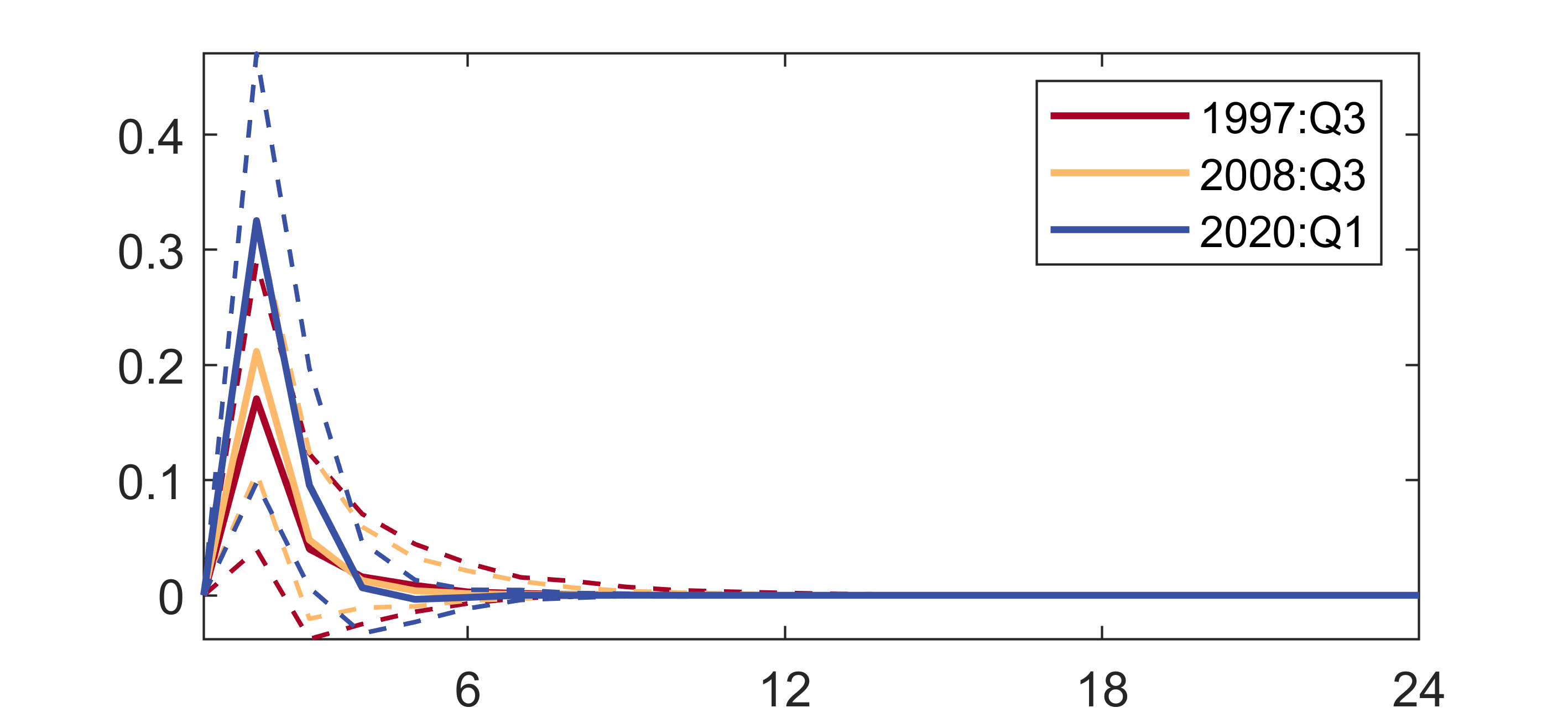

Supplement: Supplementary file 4 [file Data_Sheet_2.ZIP › COM_KR_2 (3).tif]

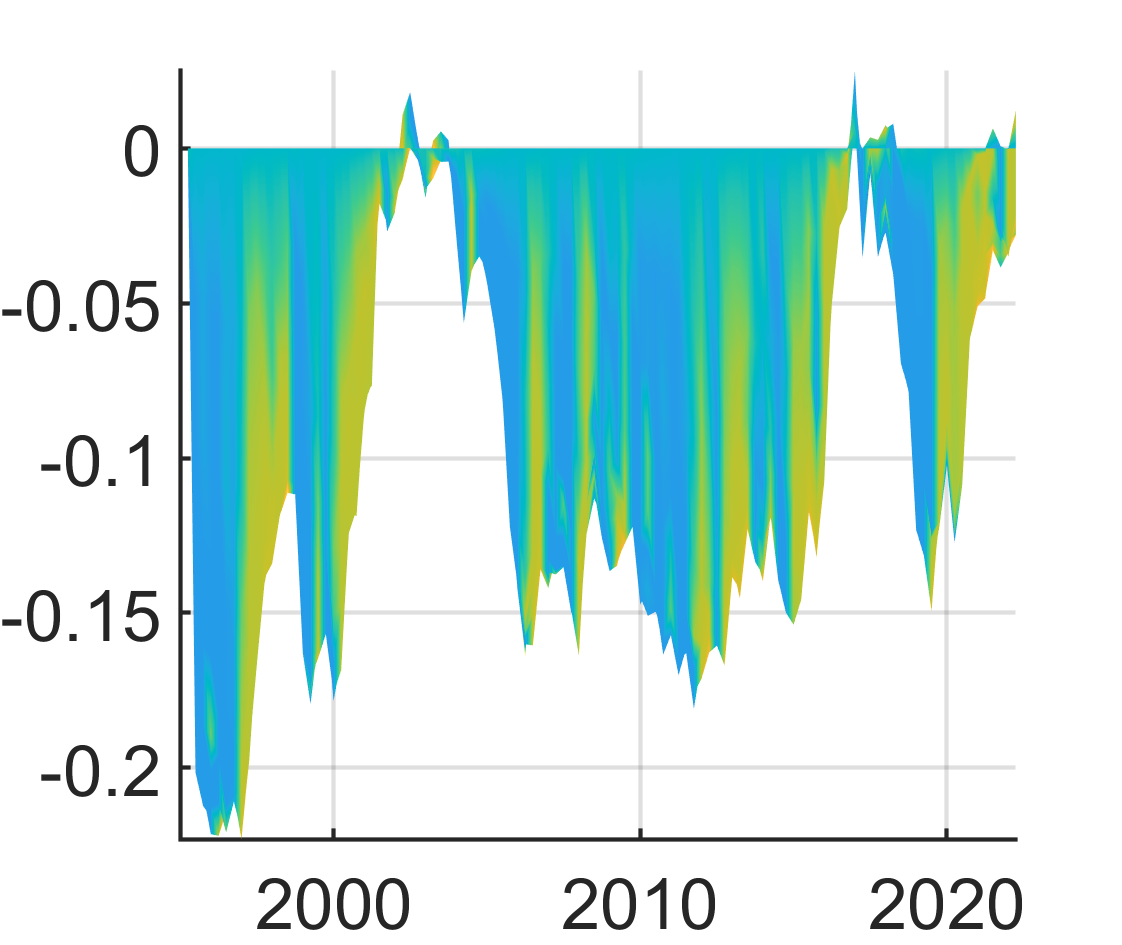

Supplement: Supplementary file 4 [file Data_Sheet_2.ZIP › FEM_CHN_2 (1).tif]

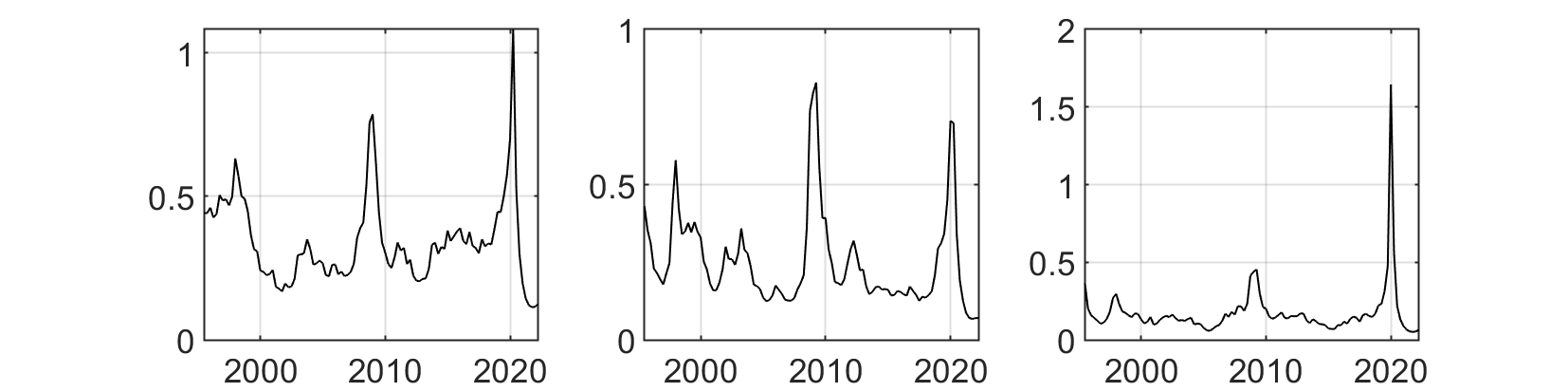

Supplement: Supplementary file 4 [file Data_Sheet_2.ZIP › FEM_CHN_2 (2).tif]

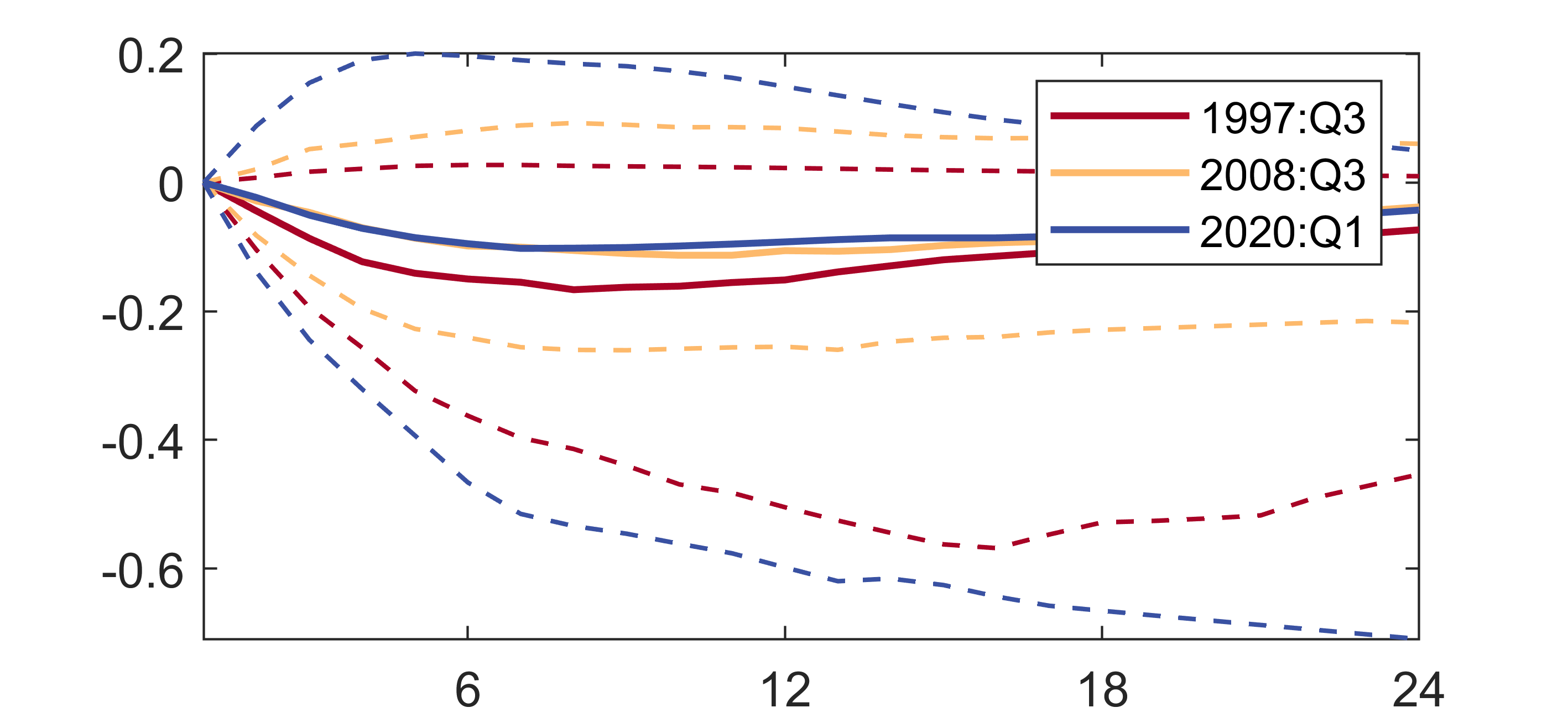

Supplement: Supplementary file 4 [file Data_Sheet_2.ZIP › FEM_CHN_2 (3).tif]

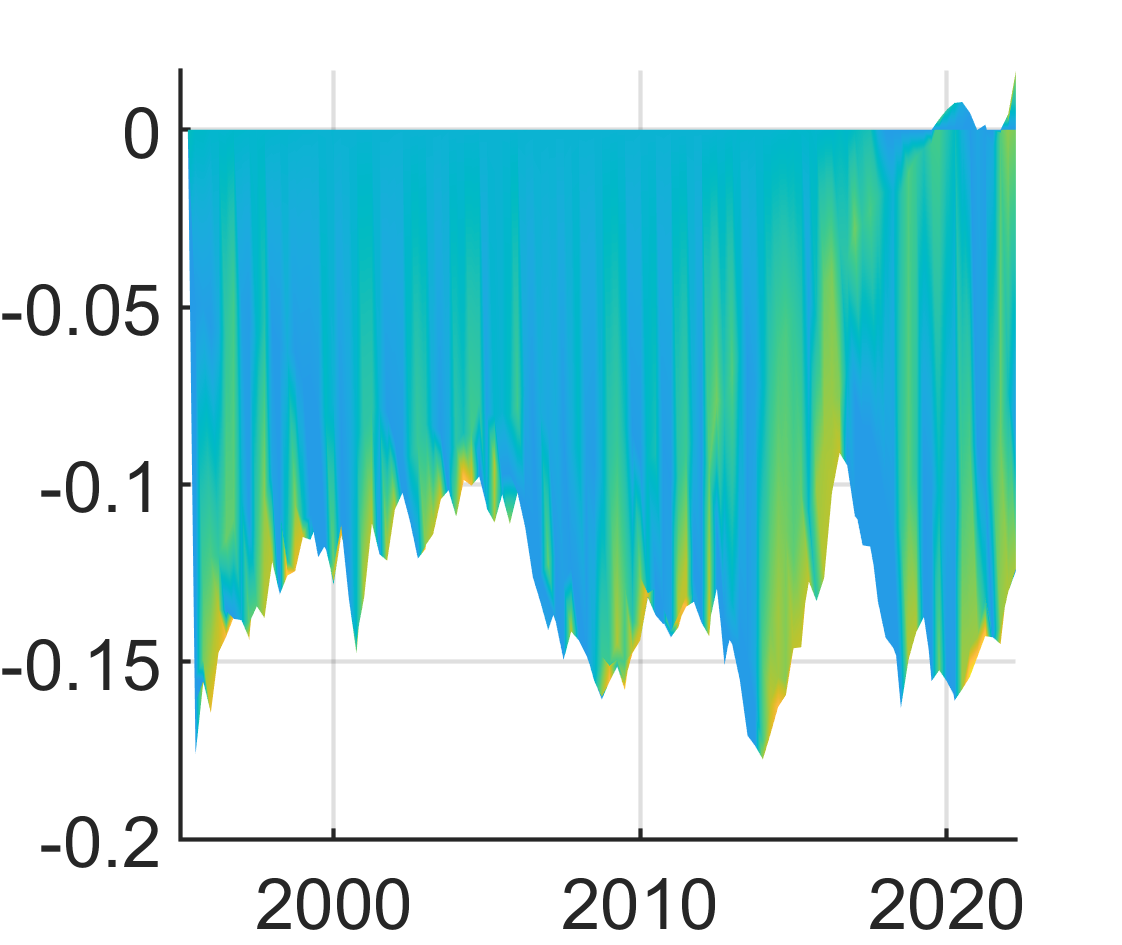

Supplement: Supplementary file 4 [file Data_Sheet_2.ZIP › FEM_HK_2 (1).tif]

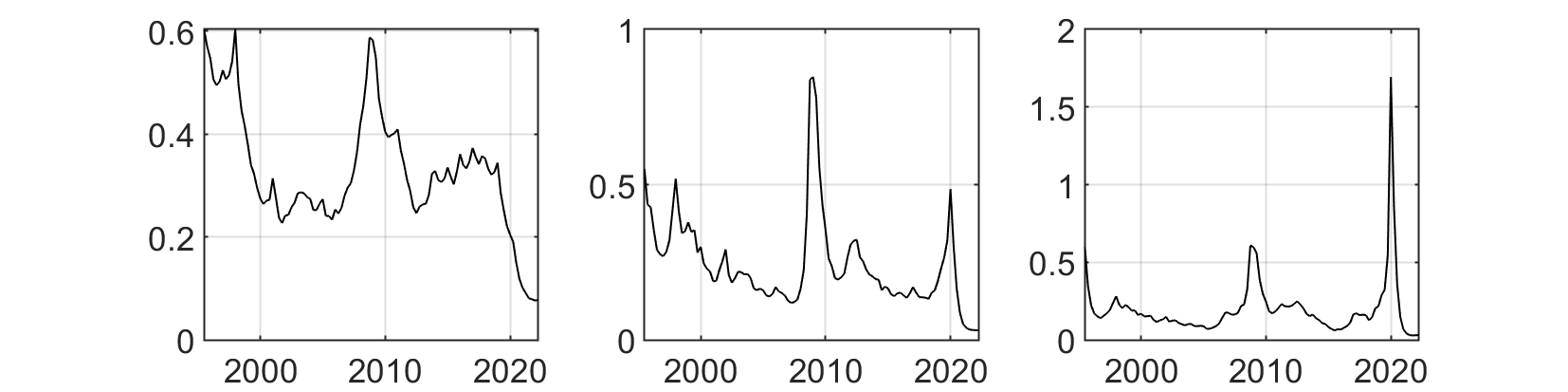

Supplement: Supplementary file 4 [file Data_Sheet_2.ZIP › FEM_HK_2 (2).tif]

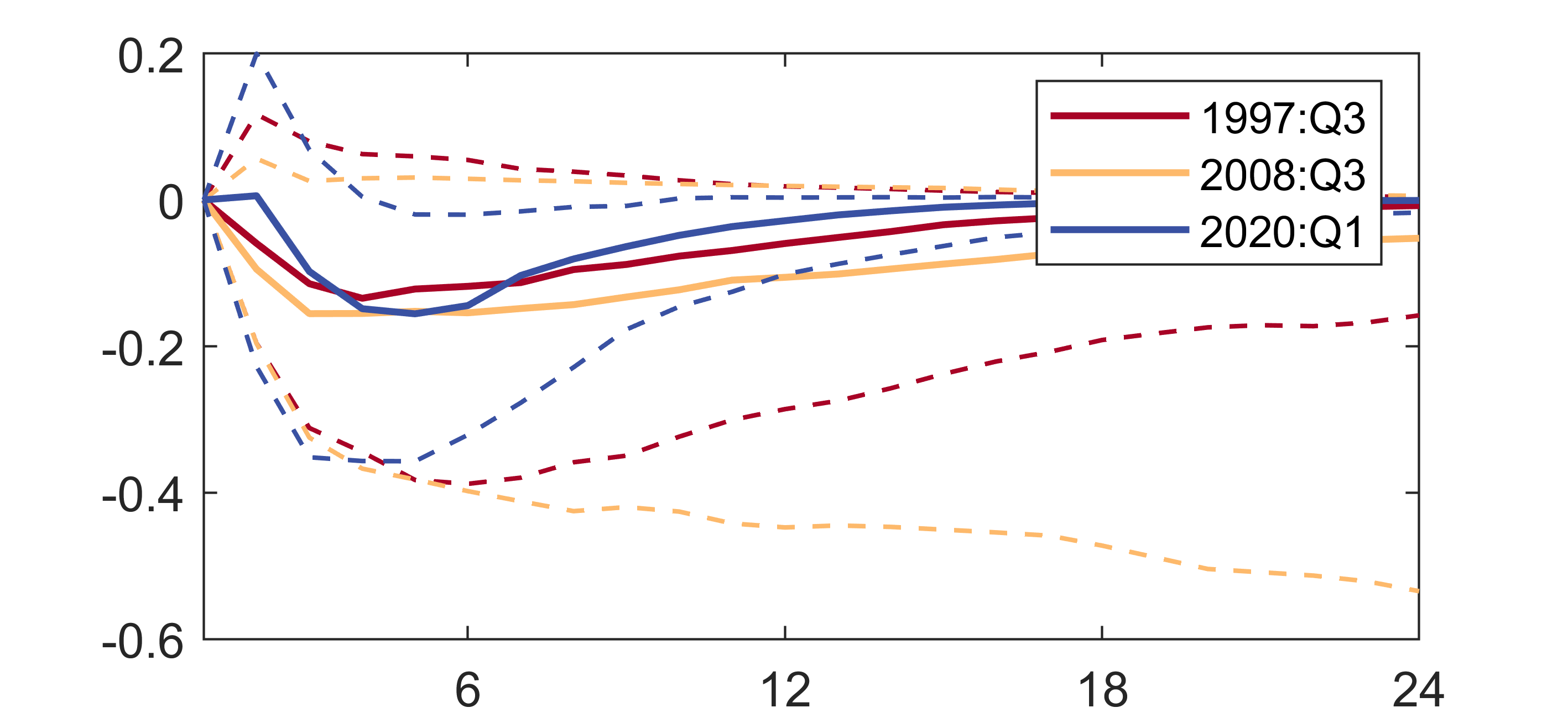

Supplement: Supplementary file 4 [file Data_Sheet_2.ZIP › FEM_HK_2 (3).tif]

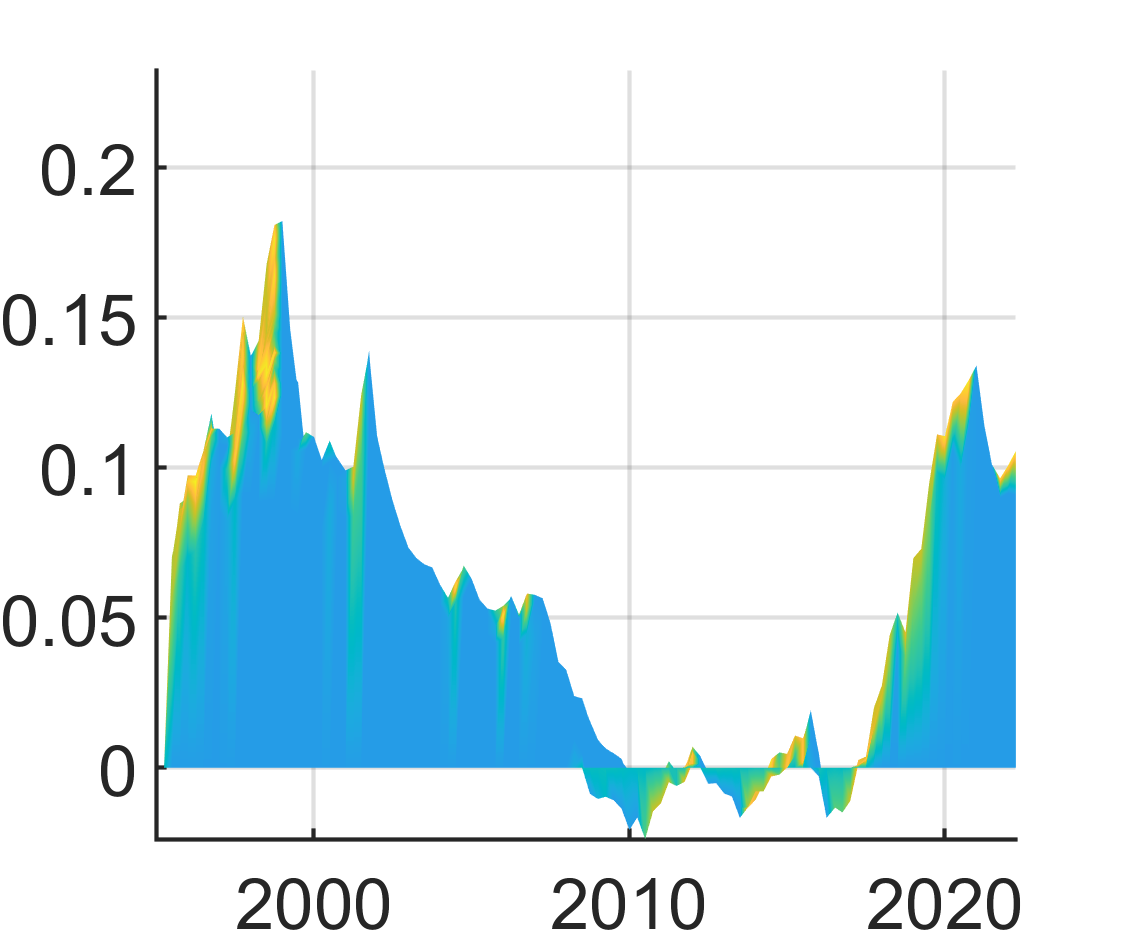

Supplement: Supplementary file 4 [file Data_Sheet_2.ZIP › FEM_JPN_2 (1).tif]

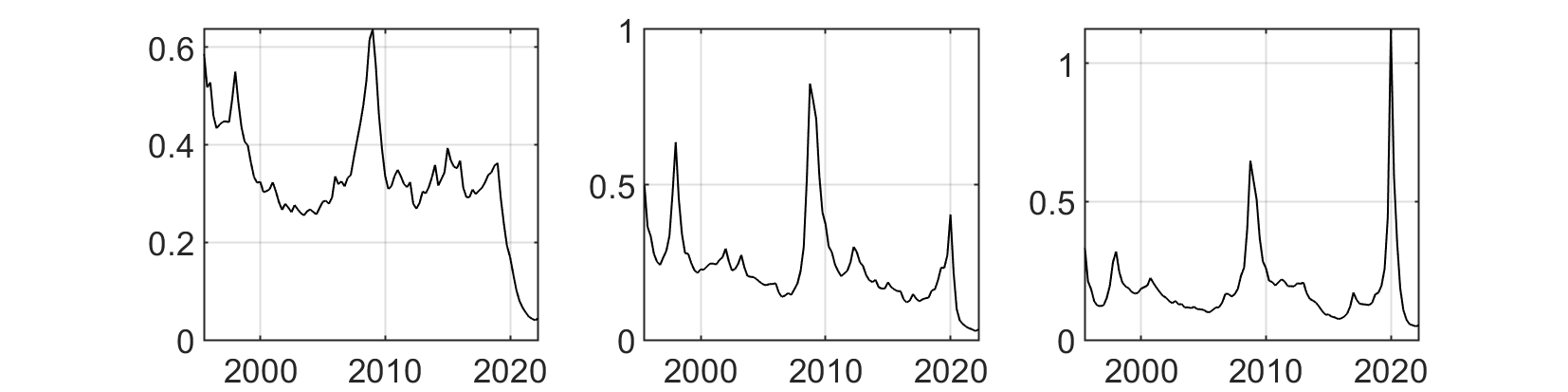

Supplement: Supplementary file 4 [file Data_Sheet_2.ZIP › FEM_JPN_2 (2).tif]

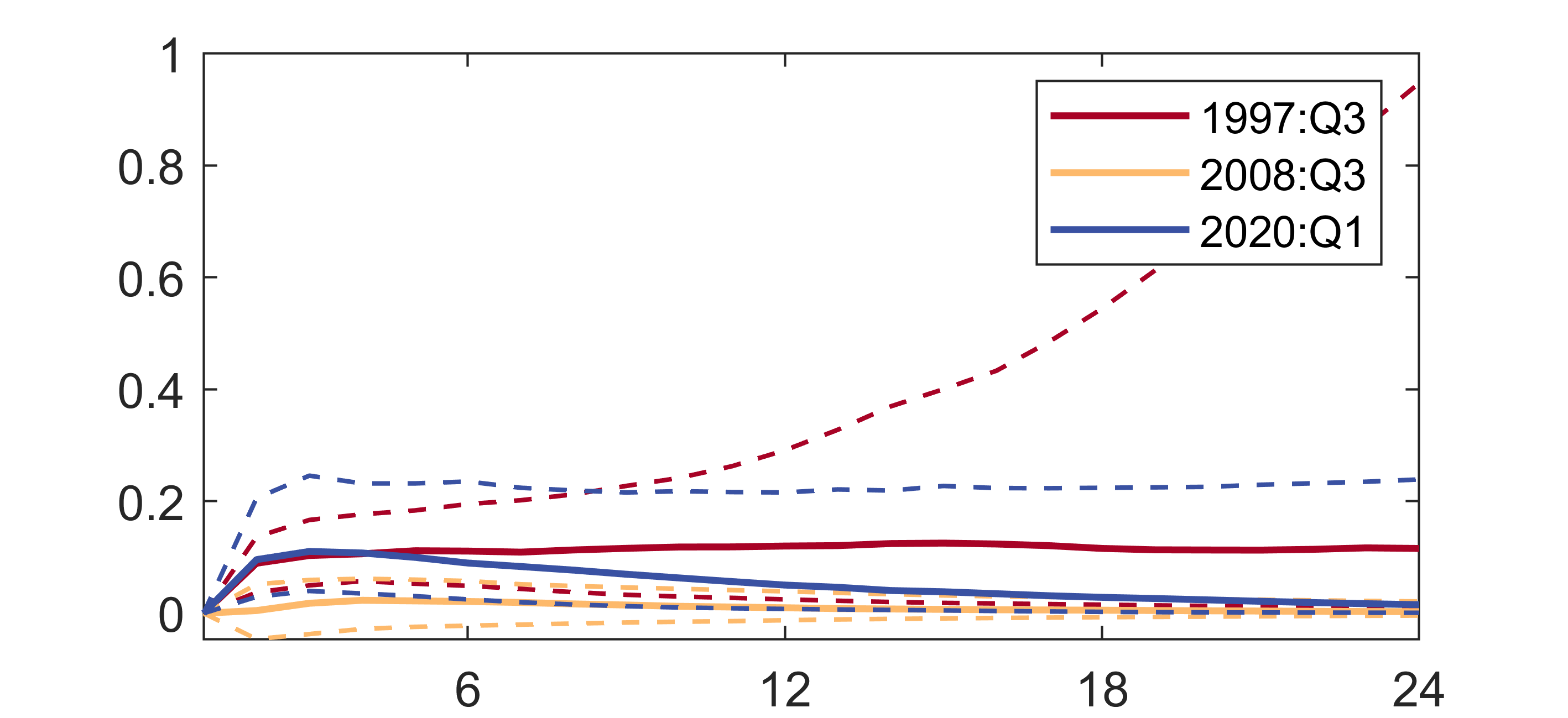

Supplement: Supplementary file 4 [file Data_Sheet_2.ZIP › FEM_JPN_2 (3).tif]

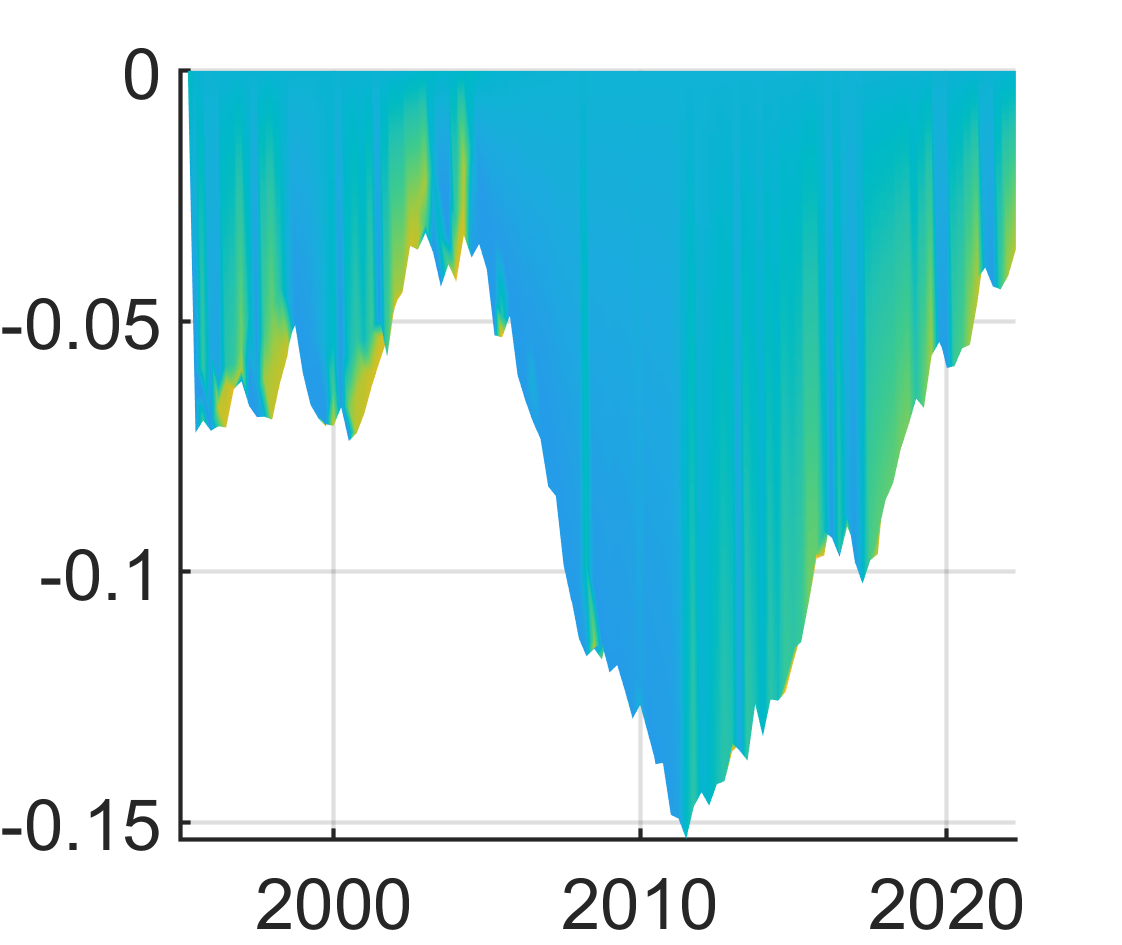

Supplement: Supplementary file 4 [file Data_Sheet_2.ZIP › FEM_KR_2 (1).tif]

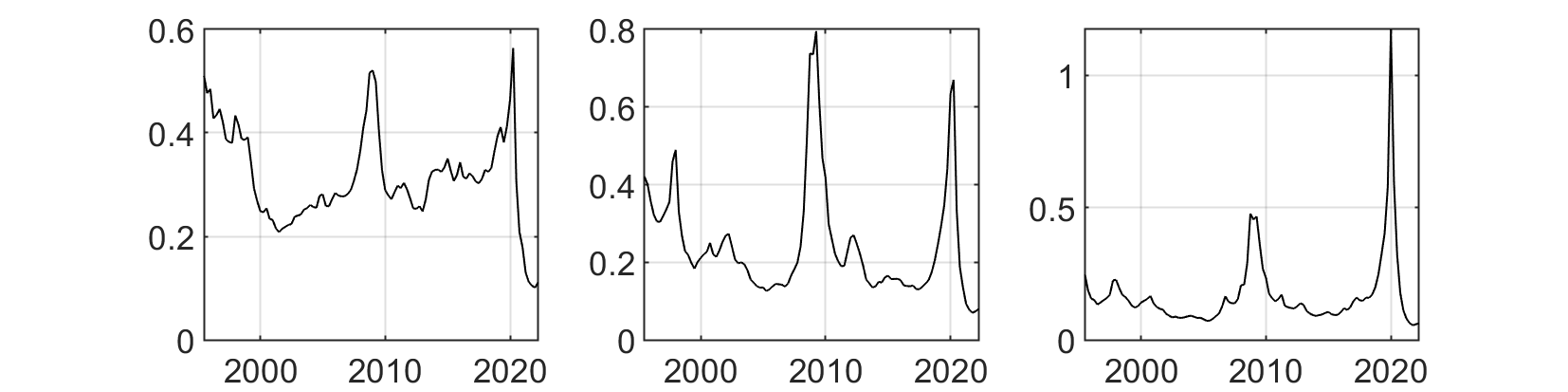

Supplement: Supplementary file 4 [file Data_Sheet_2.ZIP › FEM_KR_2 (2).tif]

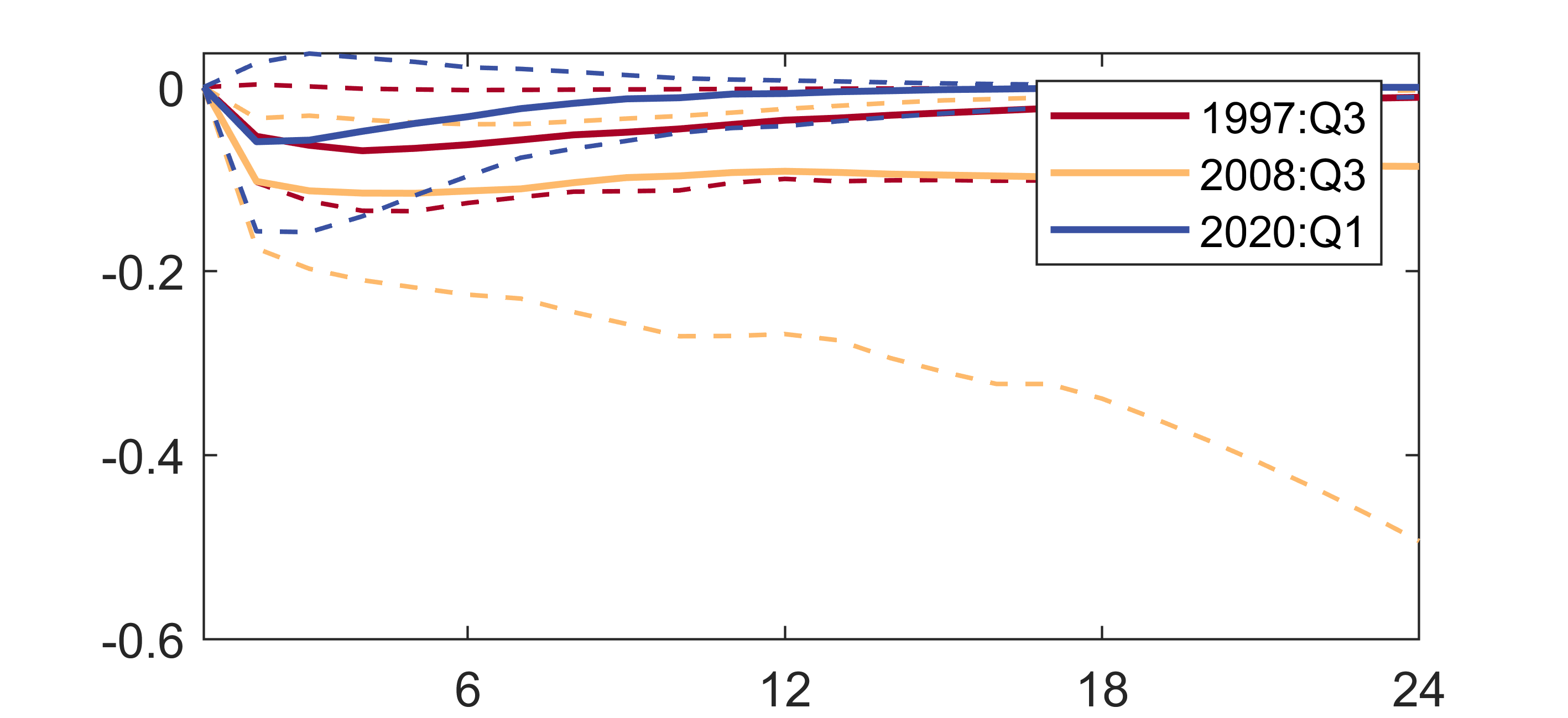

Supplement: Supplementary file 4 [file Data_Sheet_2.ZIP › FEM_KR_2 (3).tif]

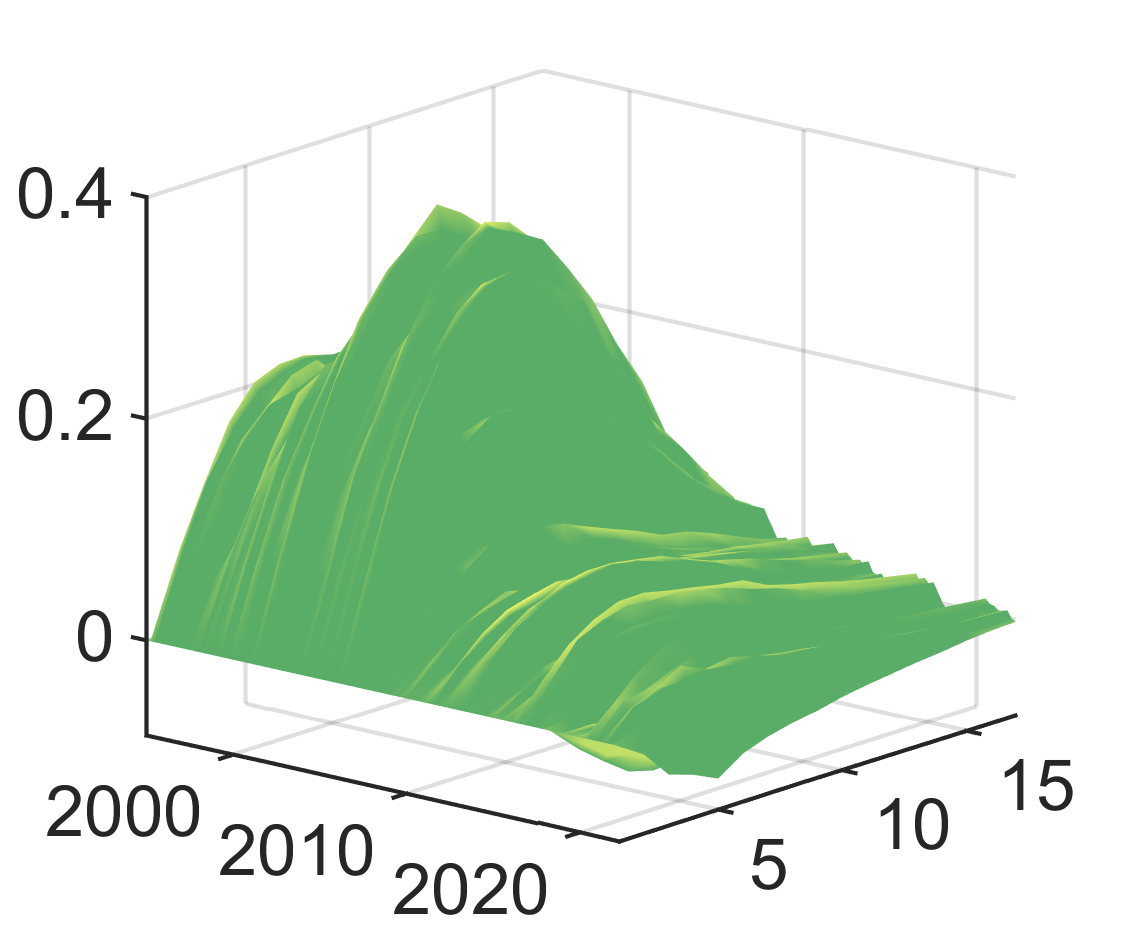

Supplement: Supplementary file 4 [file Data_Sheet_2.ZIP › HK_CHN_2 (1).tif]

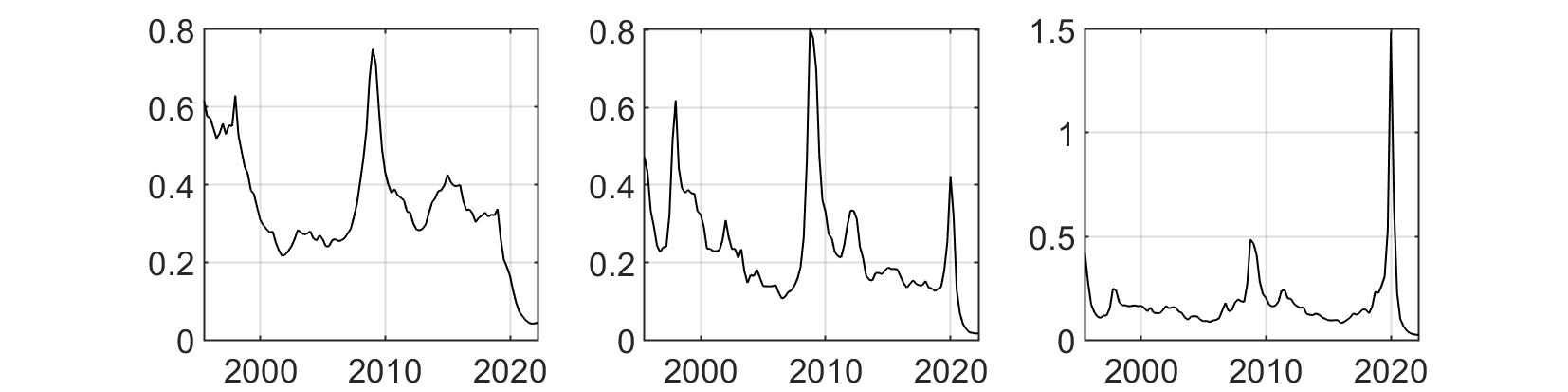

Supplement: Supplementary file 4 [file Data_Sheet_2.ZIP › HK_CHN_2 (2).tif]

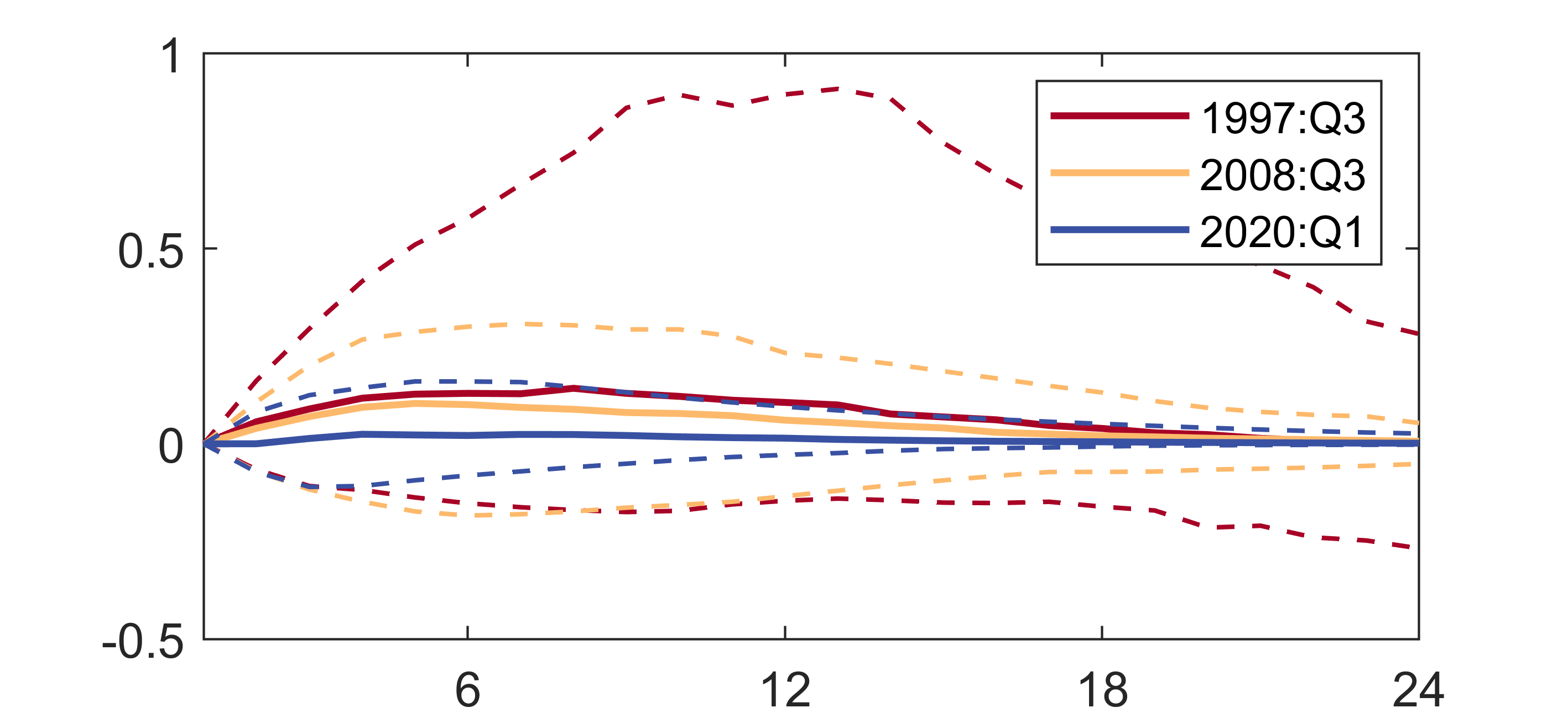

Supplement: Supplementary file 4 [file Data_Sheet_2.ZIP › HK_CHN_2 (3).tif]

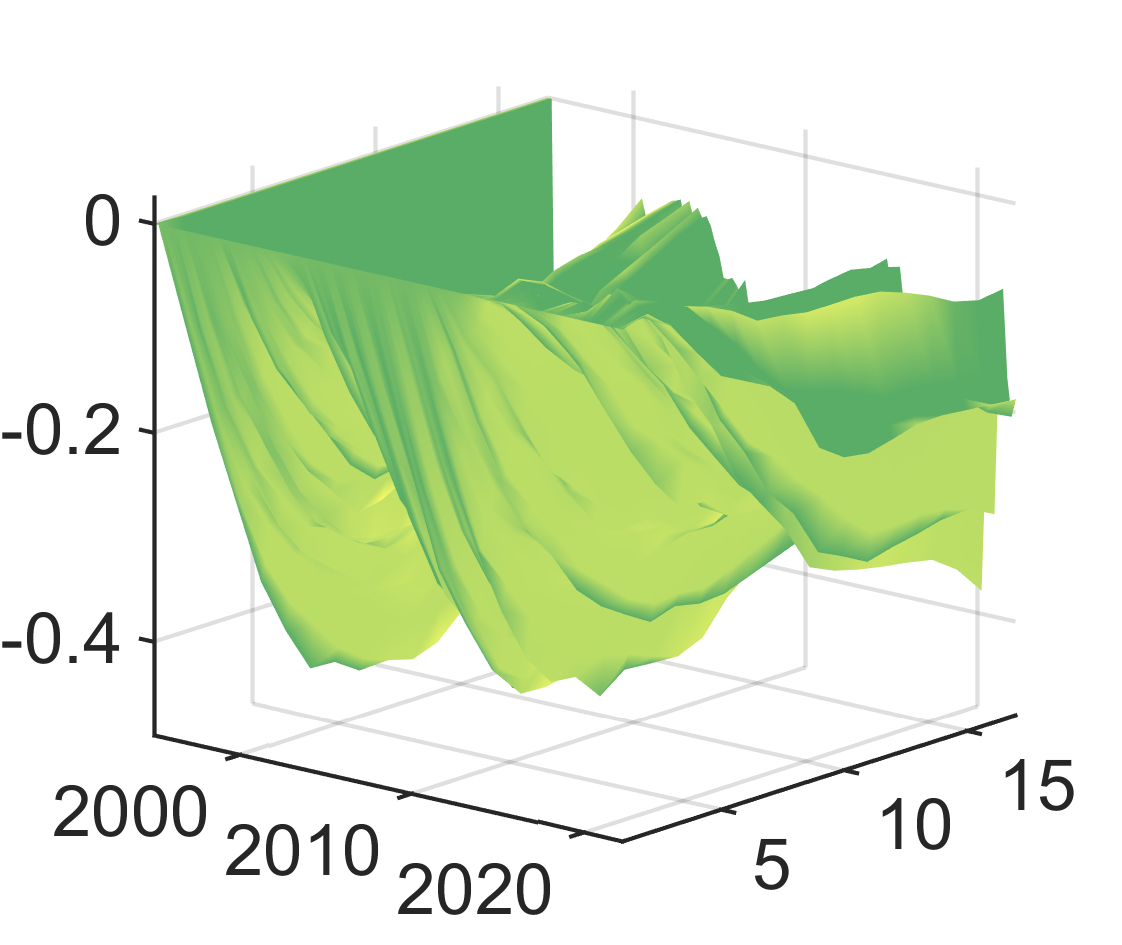

Supplement: Supplementary file 4 [file Data_Sheet_2.ZIP › HK_JPN_2 (1).tif]

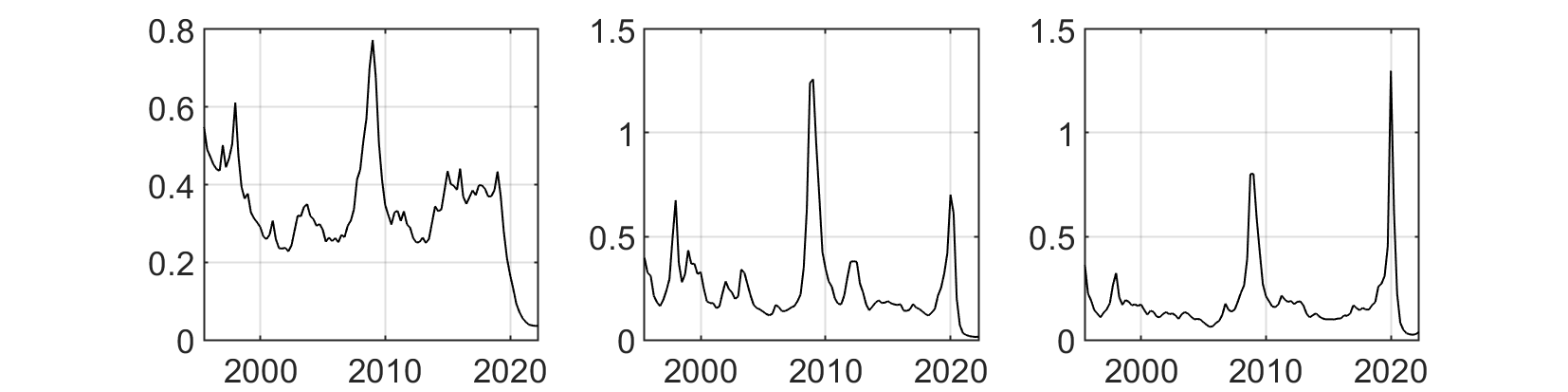

Supplement: Supplementary file 4 [file Data_Sheet_2.ZIP › HK_JPN_2 (2).tif]

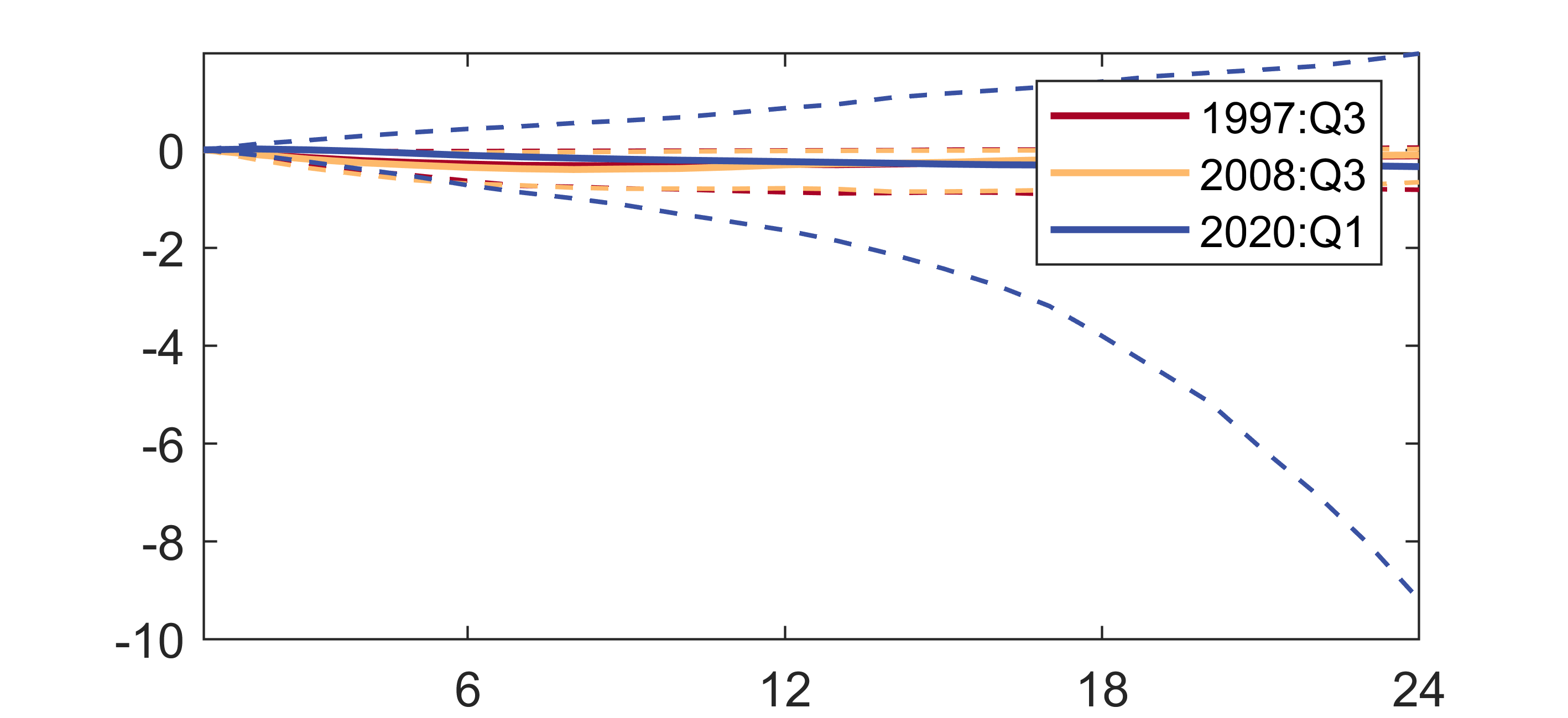

Supplement: Supplementary file 4 [file Data_Sheet_2.ZIP › HK_JPN_2 (3).tif]

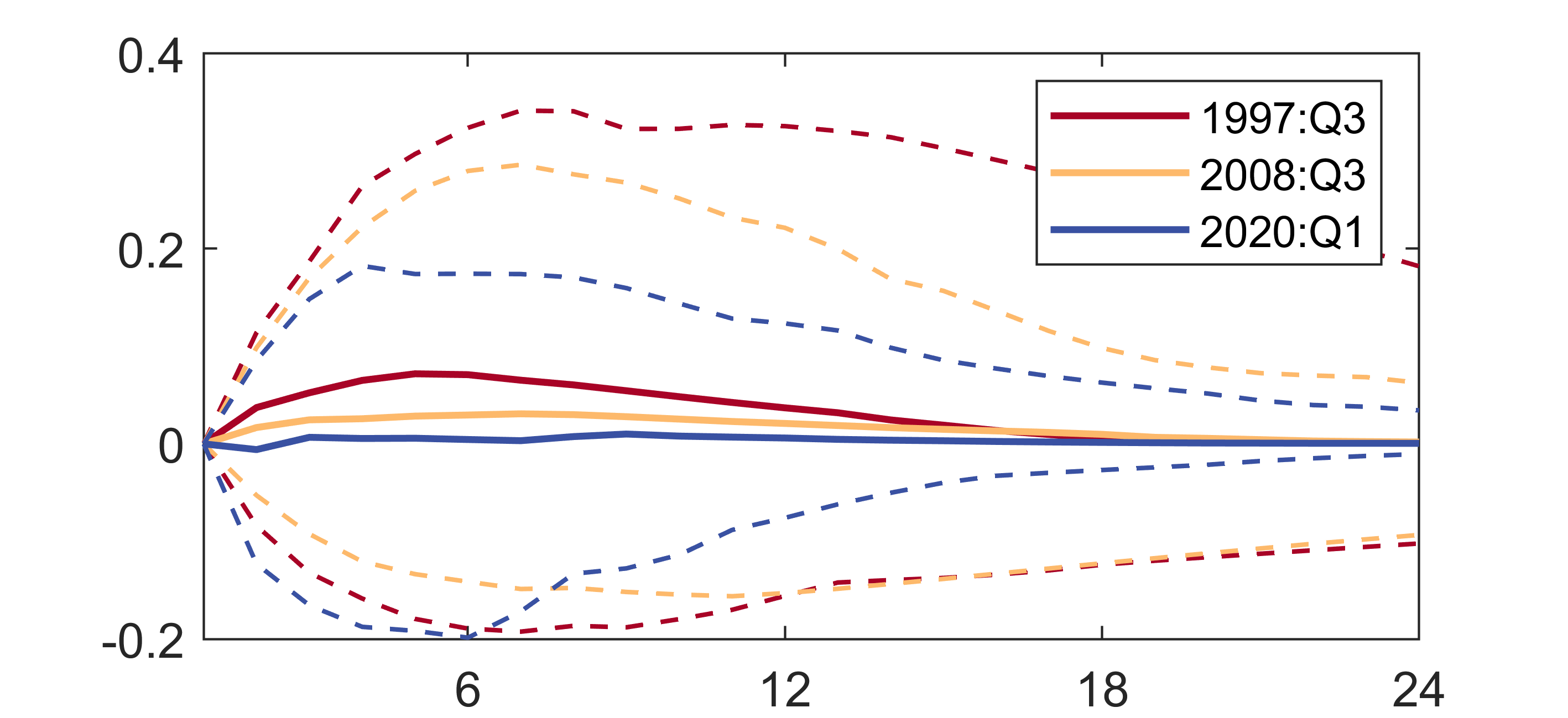

Supplement: Supplementary file 4 [file Data_Sheet_2.ZIP › HK_KR_2 (1).tif]

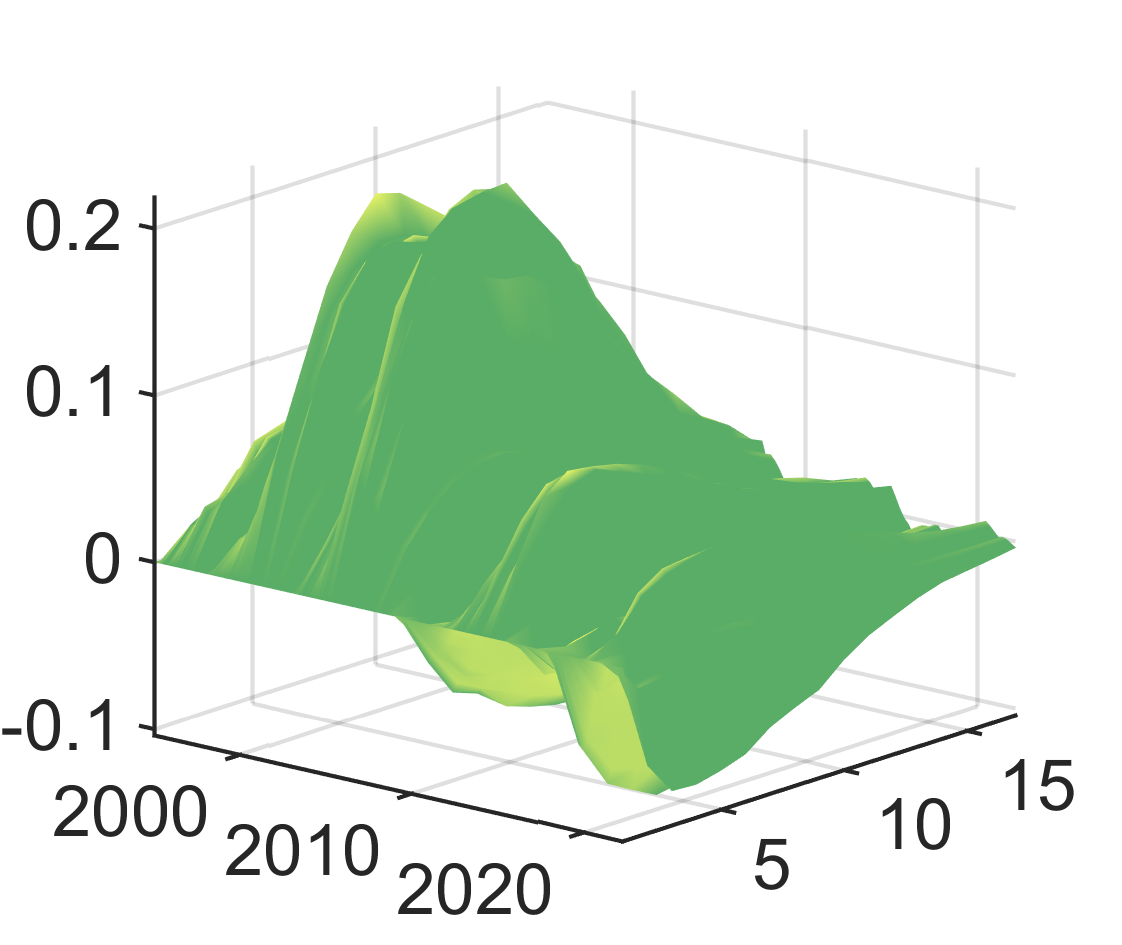

Supplement: Supplementary file 4 [file Data_Sheet_2.ZIP › HK_KR_2 (2).tif]

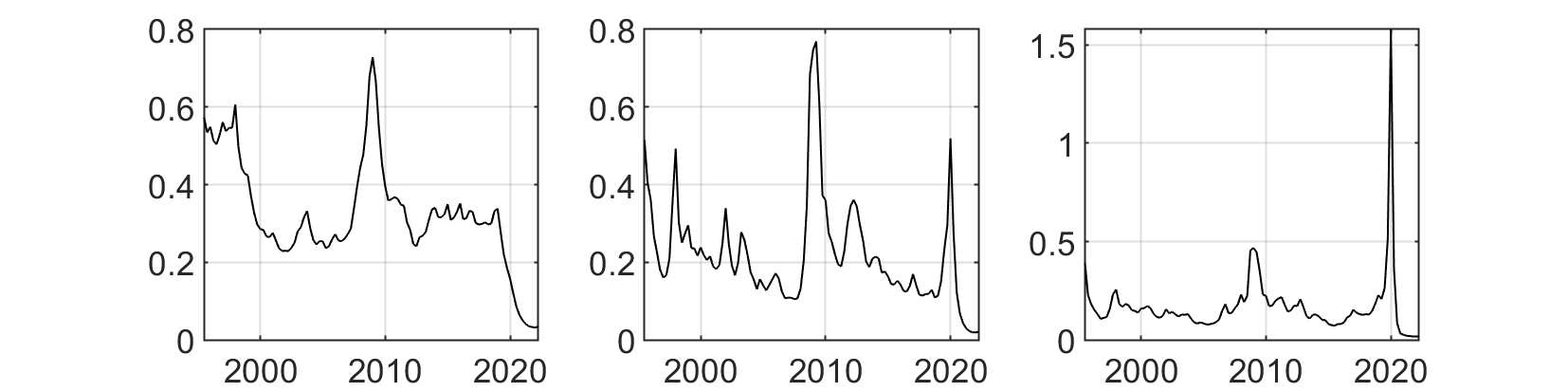

Supplement: Supplementary file 4 [file Data_Sheet_2.ZIP › HK_KR_2 (3).tif]

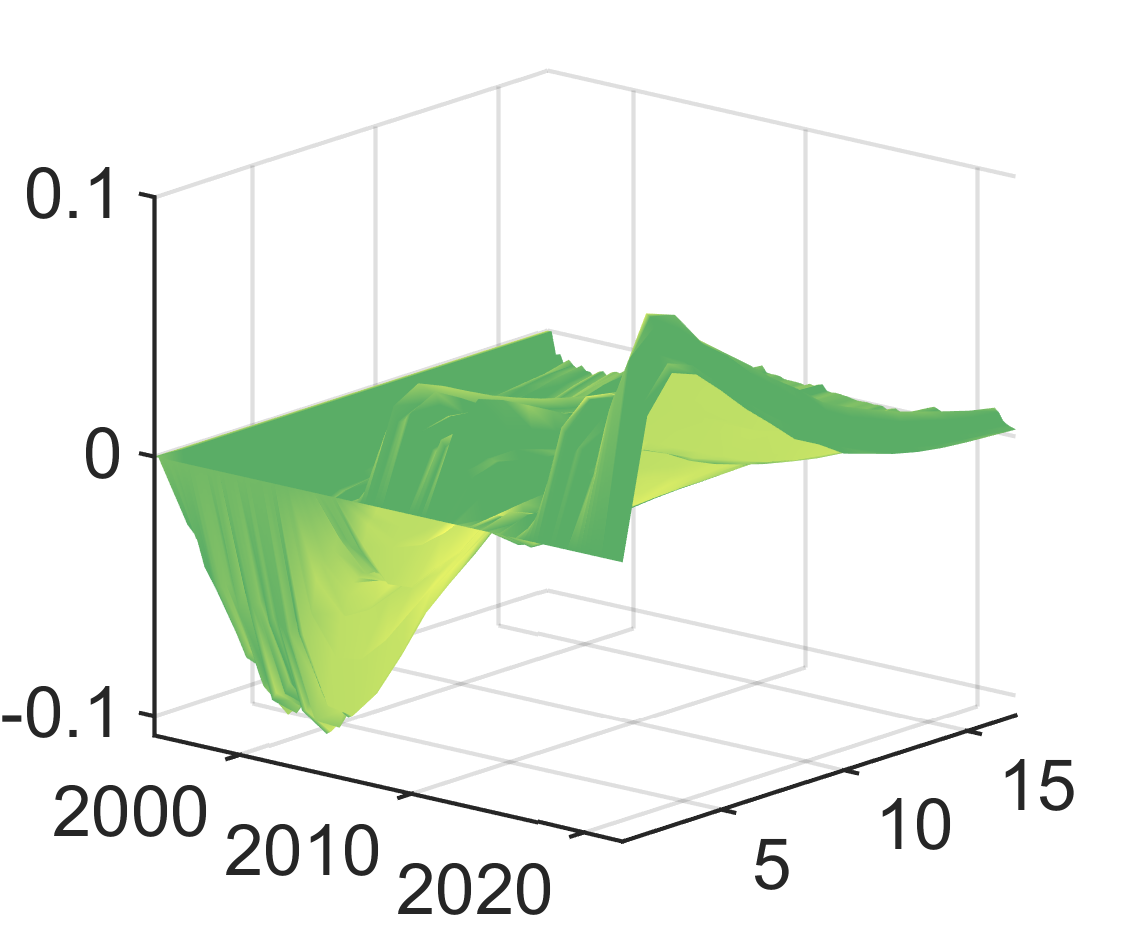

Supplement: Supplementary file 4 [file Data_Sheet_2.ZIP › JPN_CHN_2 (1).tif]

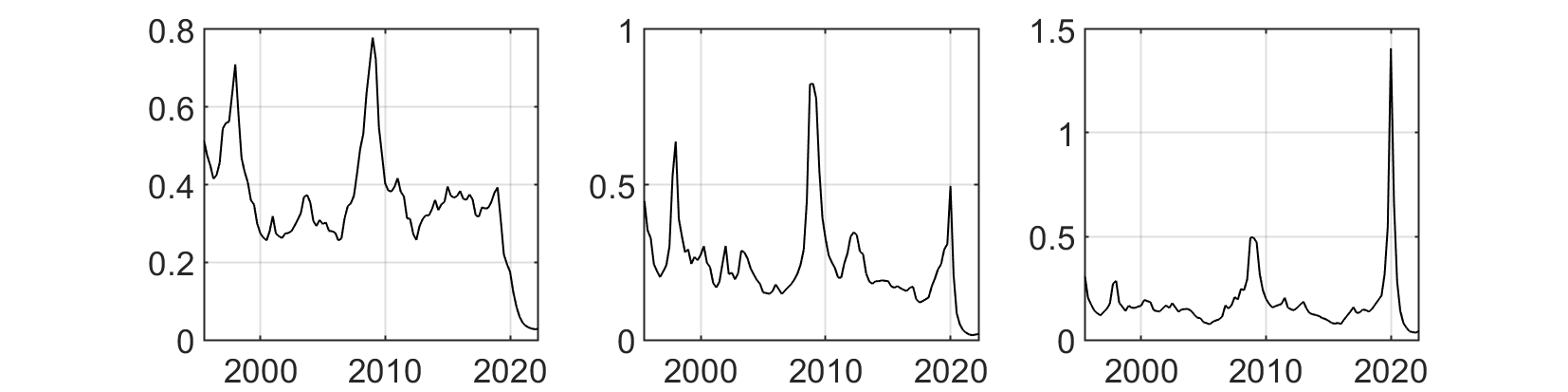

Supplement: Supplementary file 4 [file Data_Sheet_2.ZIP › JPN_CHN_2 (2).tif]

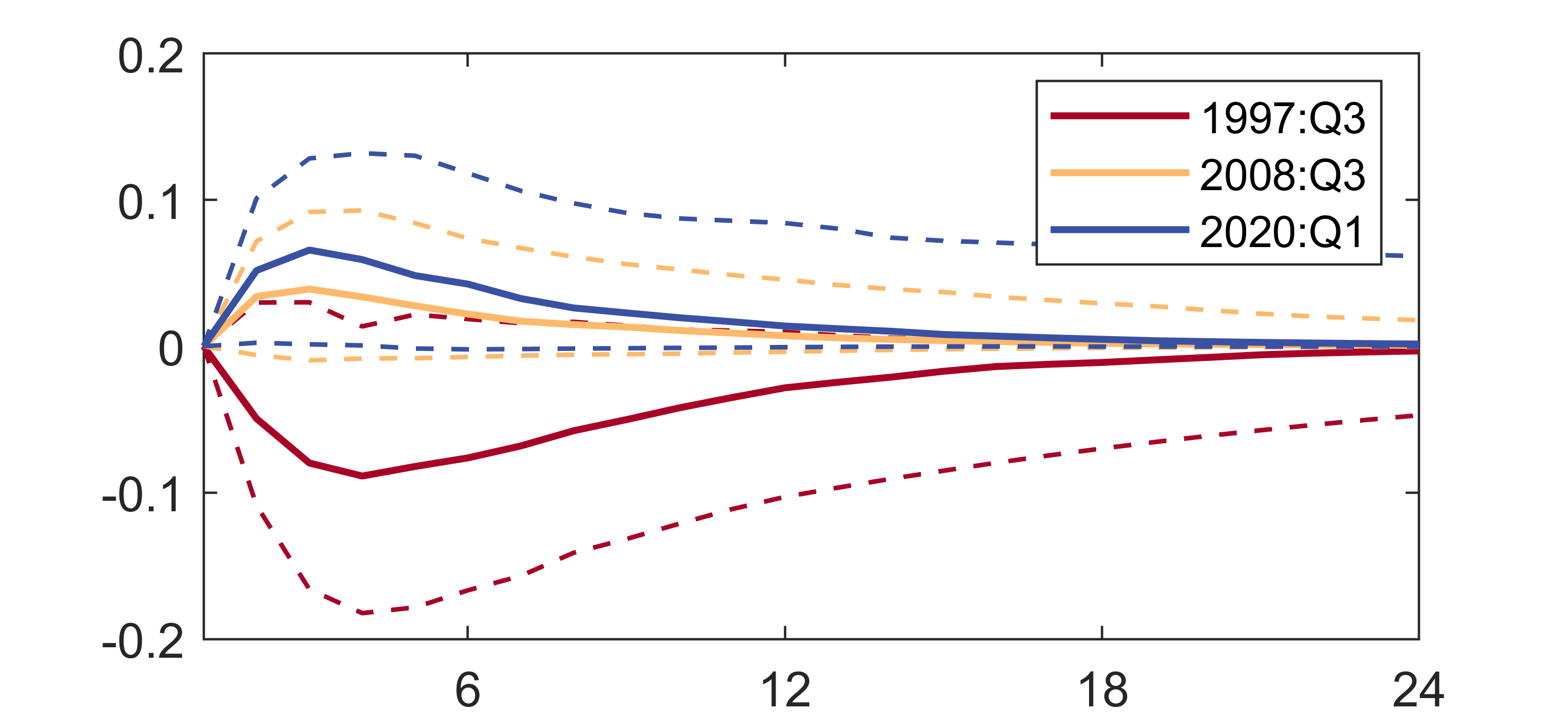

Supplement: Supplementary file 4 [file Data_Sheet_2.ZIP › JPN_CHN_2 (3).tif]

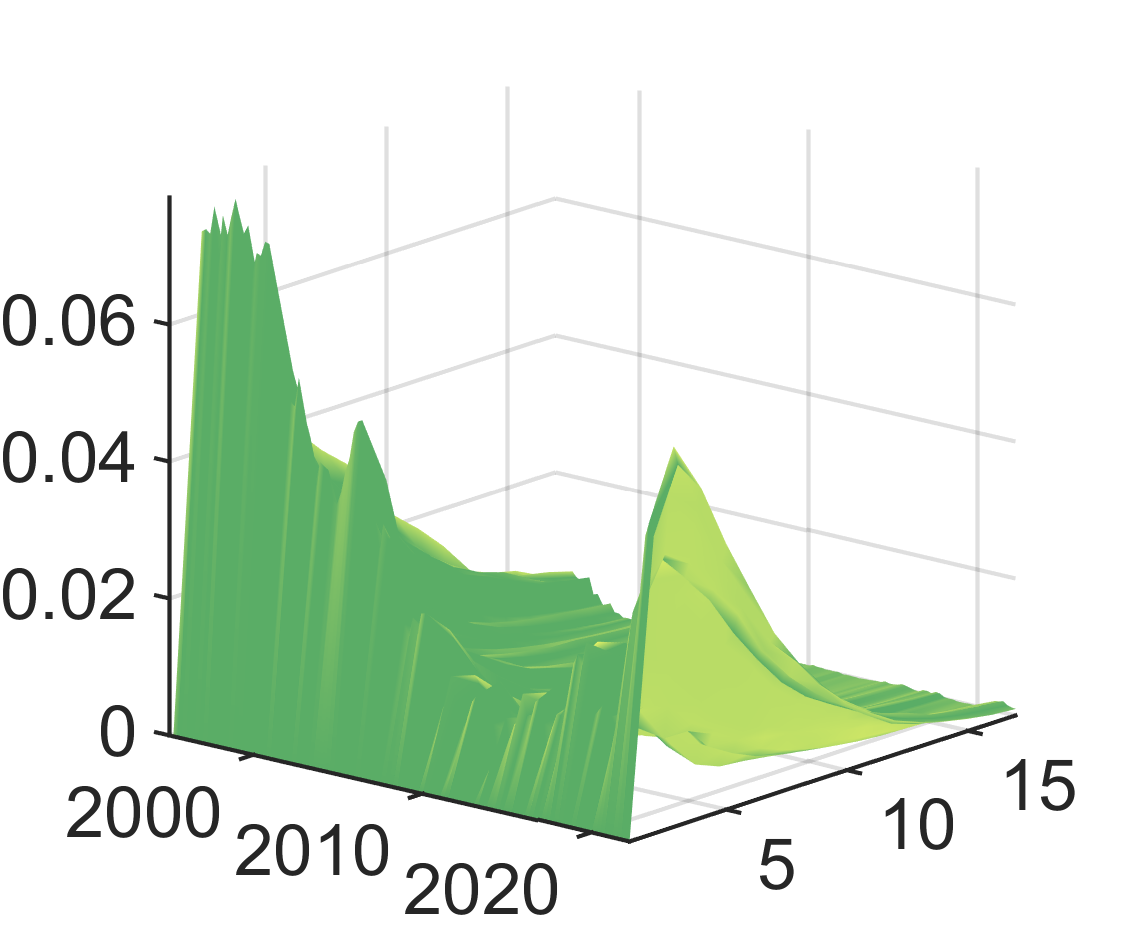

Supplement: Supplementary file 4 [file Data_Sheet_2.ZIP › JPN_HK_2 (1).tif]

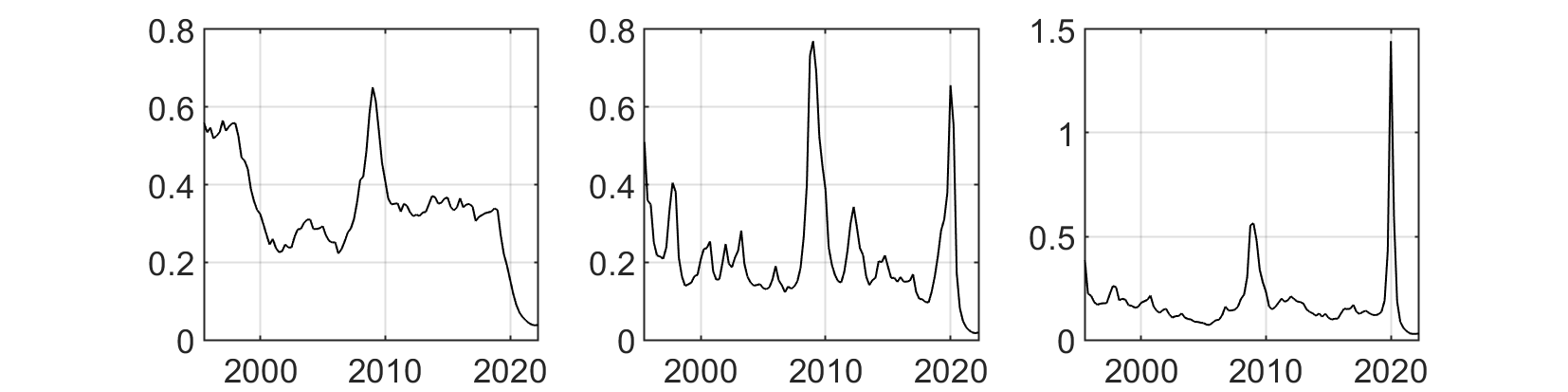

Supplement: Supplementary file 4 [file Data_Sheet_2.ZIP › JPN_HK_2 (2).tif]

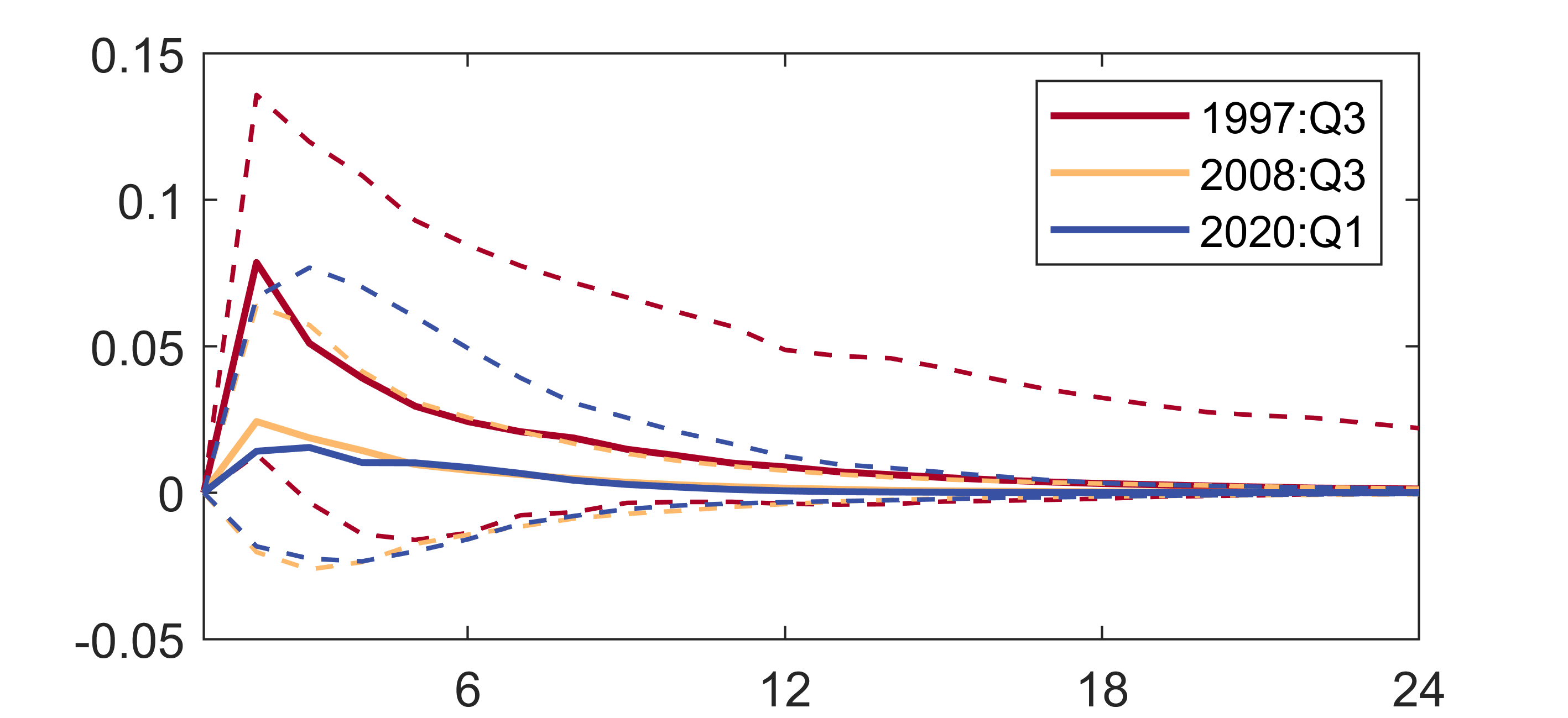

Supplement: Supplementary file 4 [file Data_Sheet_2.ZIP › JPN_HK_2 (3).tif]

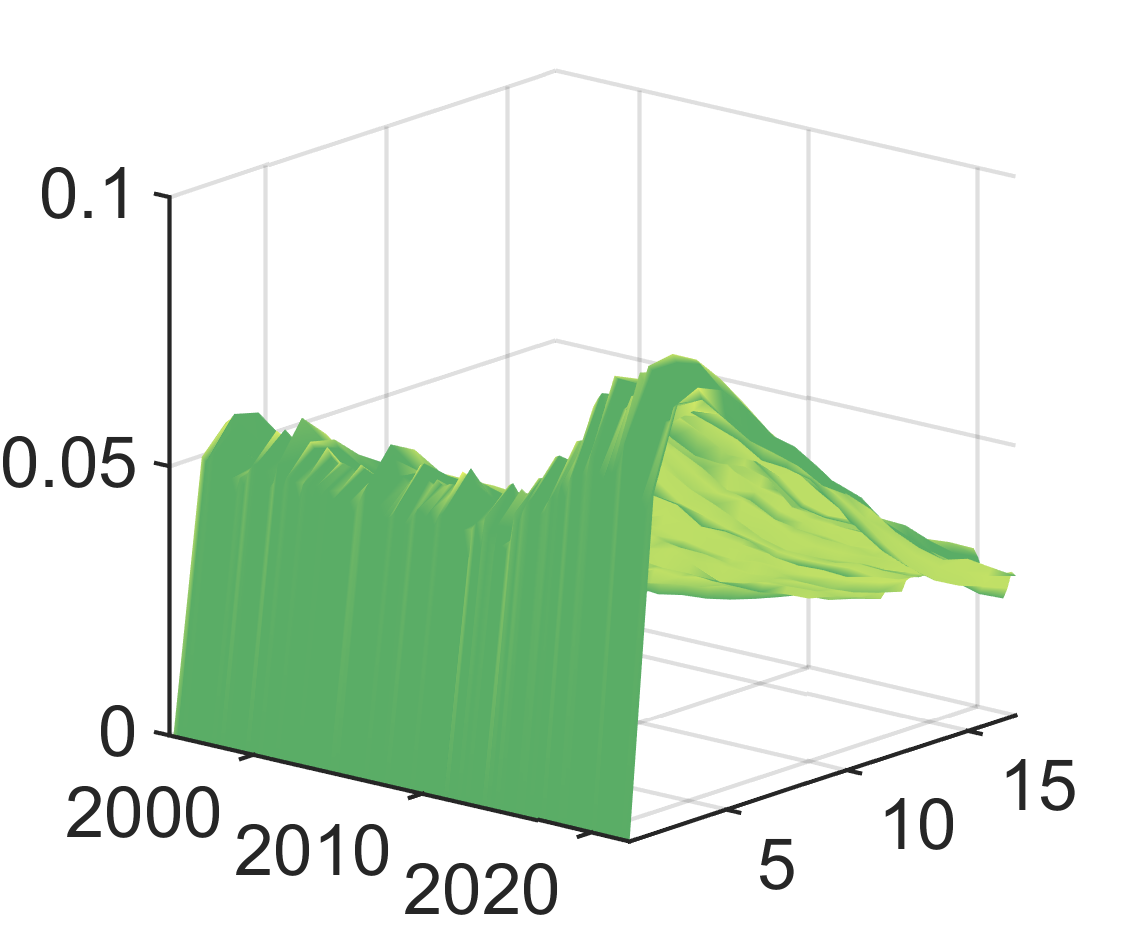

Supplement: Supplementary file 4 [file Data_Sheet_2.ZIP › JPN_KR_2 (1).tif]

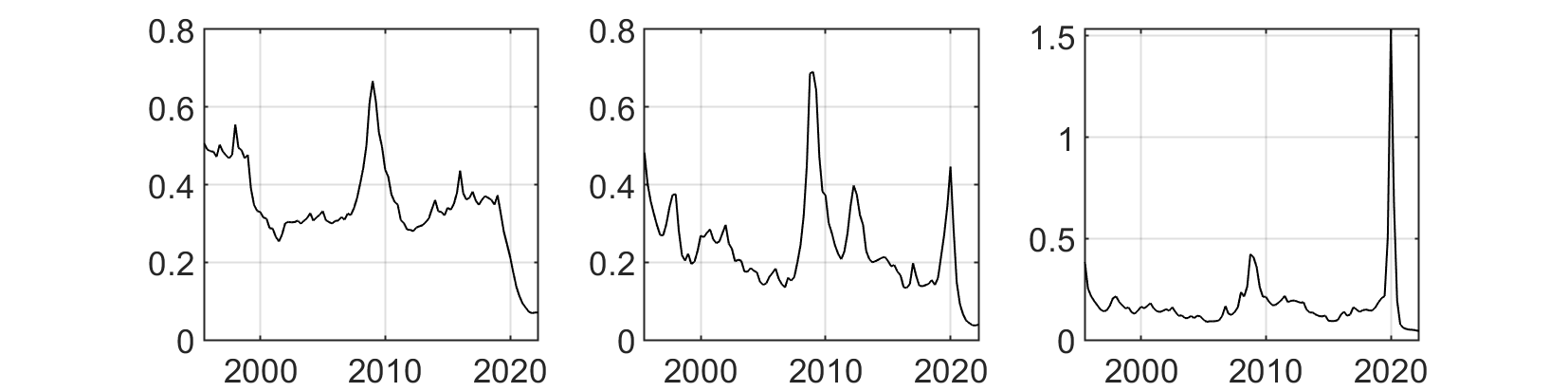

Supplement: Supplementary file 4 [file Data_Sheet_2.ZIP › JPN_KR_2 (2).tif]

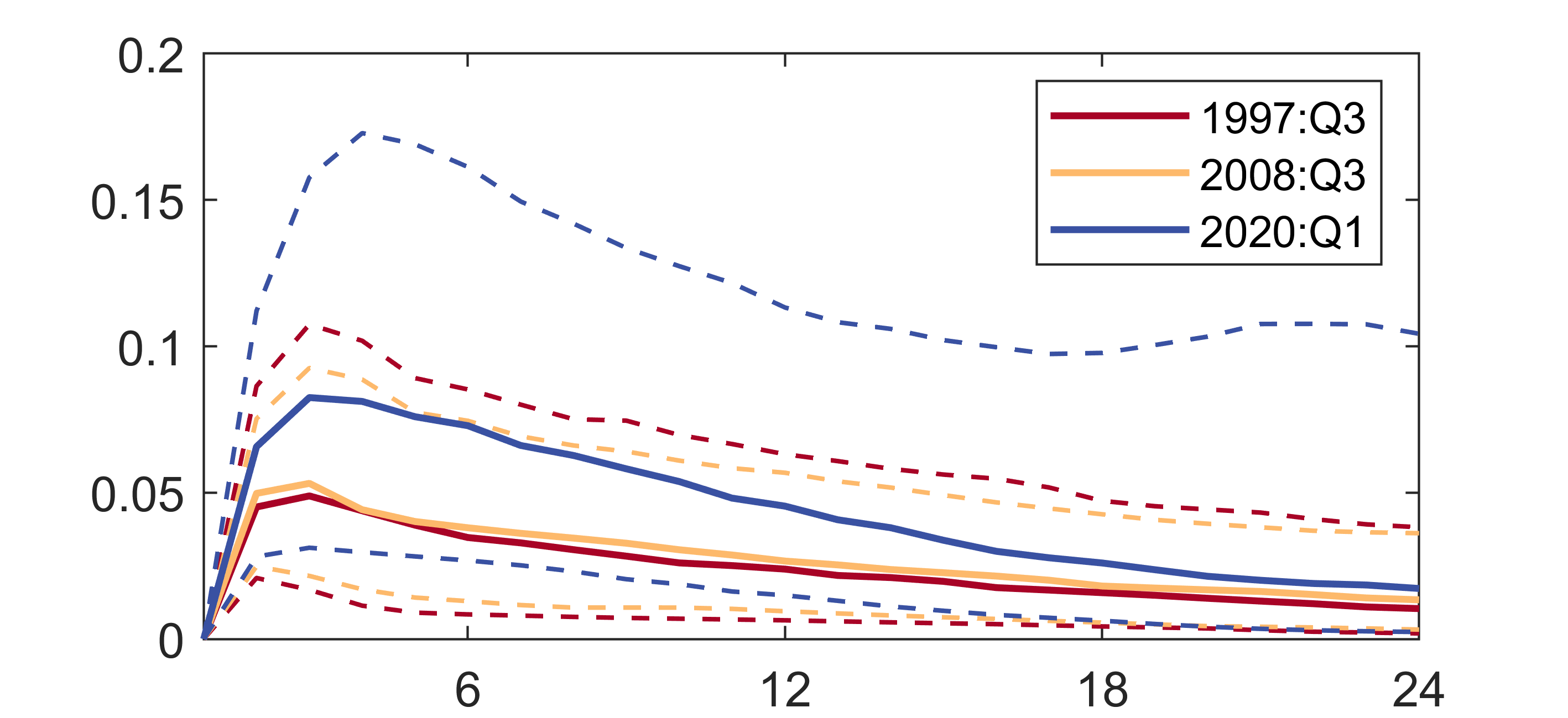

Supplement: Supplementary file 4 [file Data_Sheet_2.ZIP › JPN_KR_2 (3).tif]

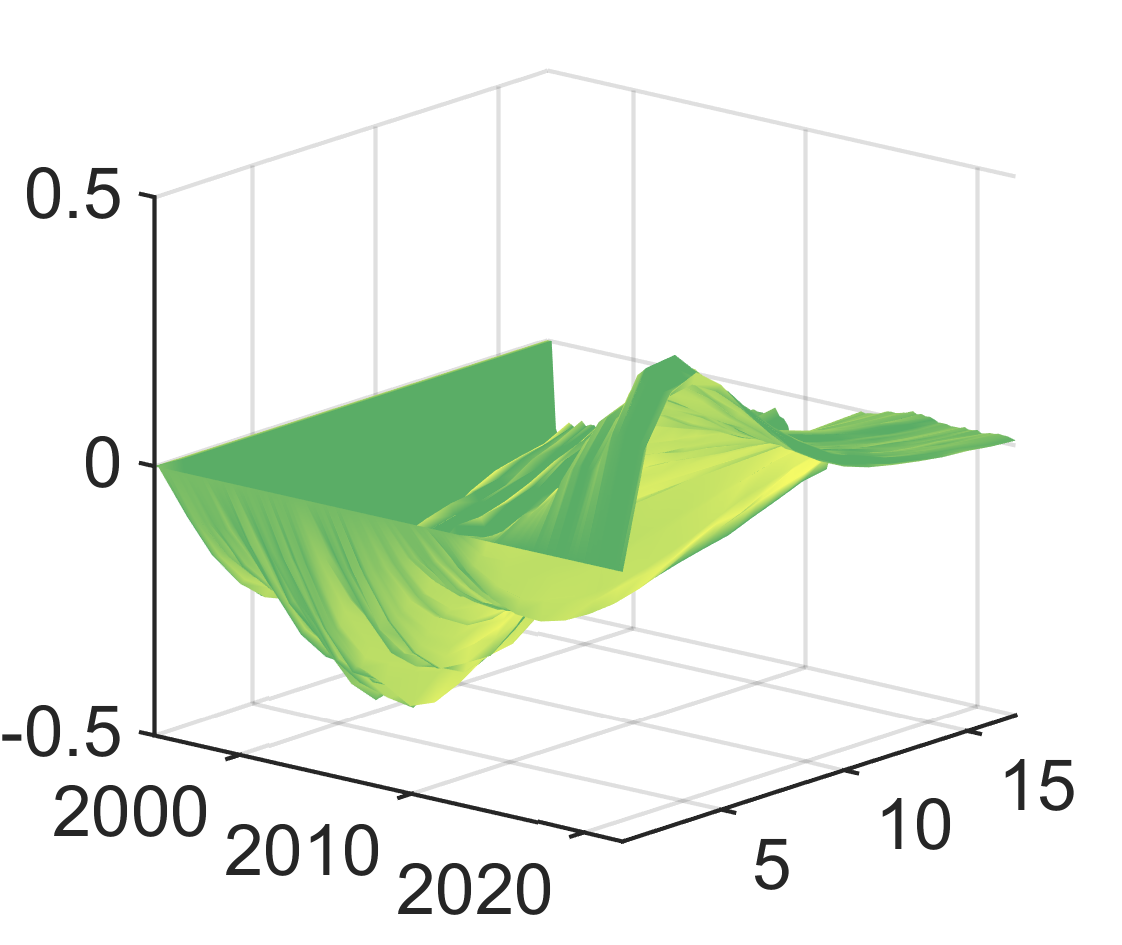

Supplement: Supplementary file 4 [file Data_Sheet_2.ZIP › KR_CHN_2 (1).tif]

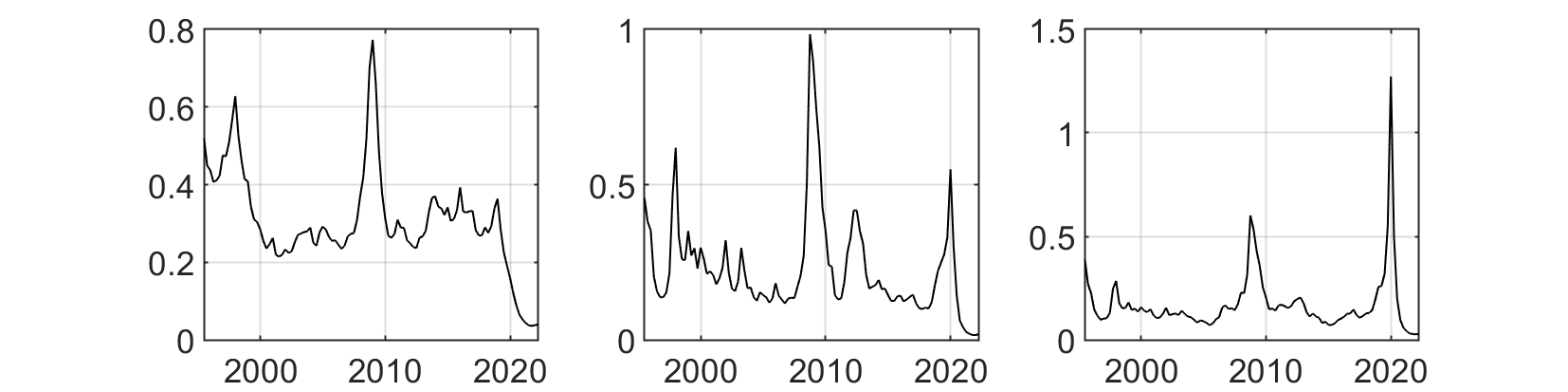

Supplement: Supplementary file 4 [file Data_Sheet_2.ZIP › KR_CHN_2 (2).tif]

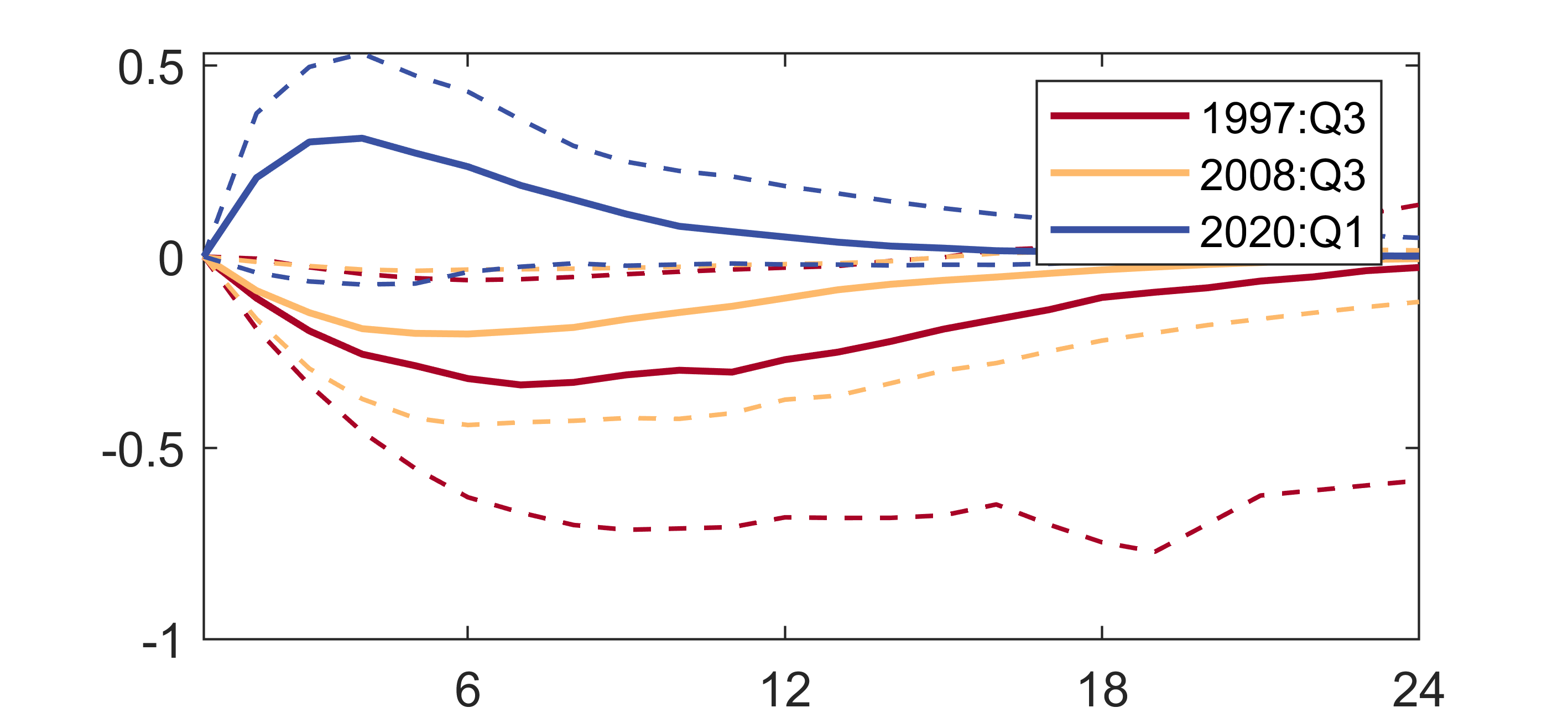

Supplement: Supplementary file 4 [file Data_Sheet_2.ZIP › KR_CHN_2 (3).tif]

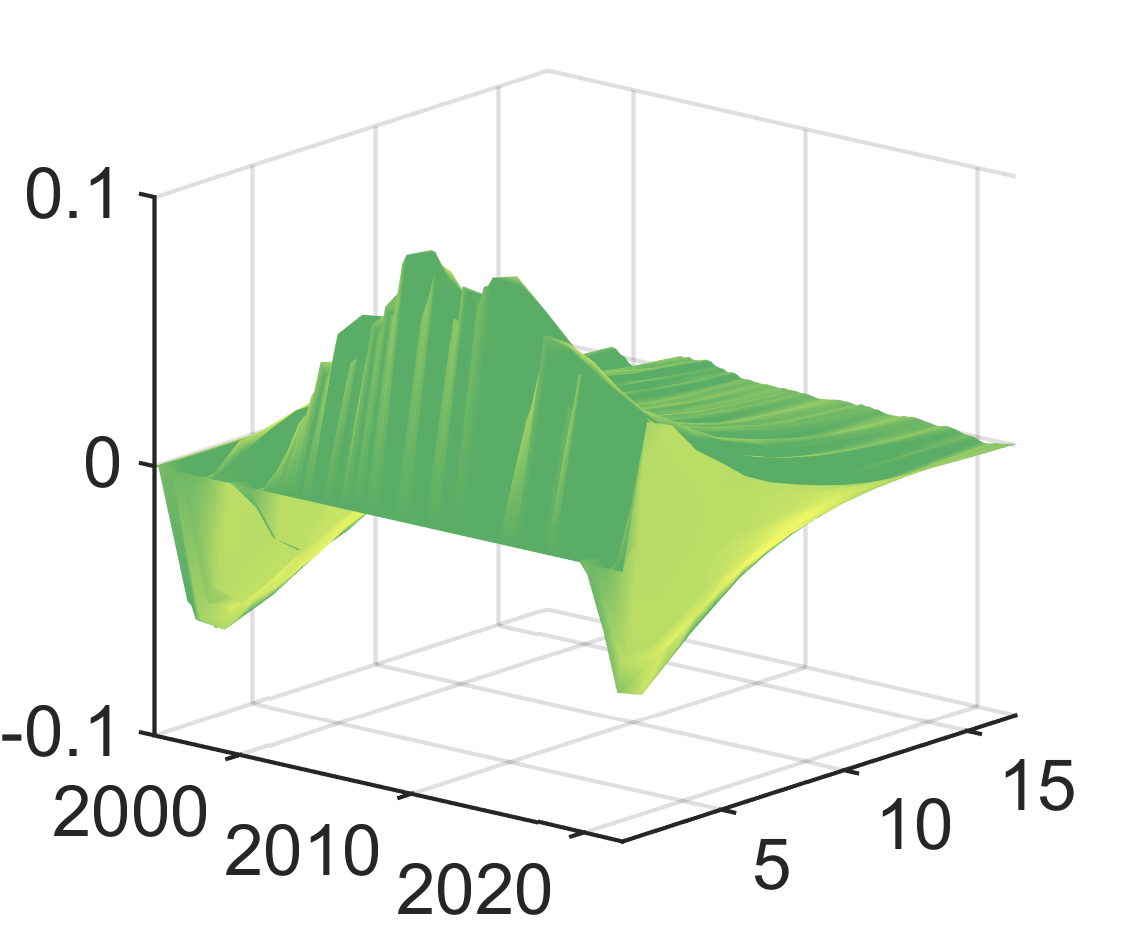

Supplement: Supplementary file 4 [file Data_Sheet_2.ZIP › KR_HK_2 (1).tif]

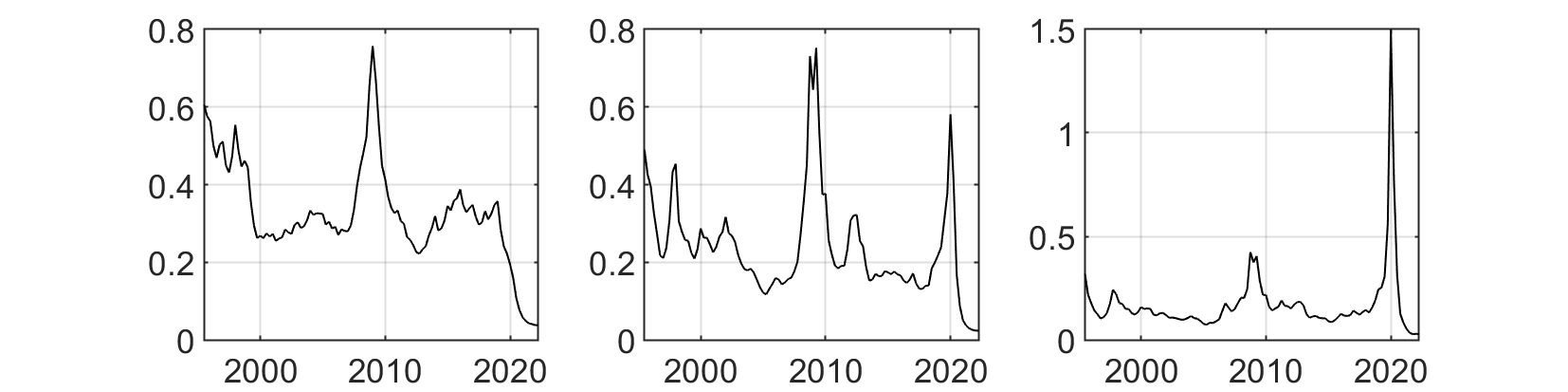

Supplement: Supplementary file 4 [file Data_Sheet_2.ZIP › KR_HK_2 (2).tif]

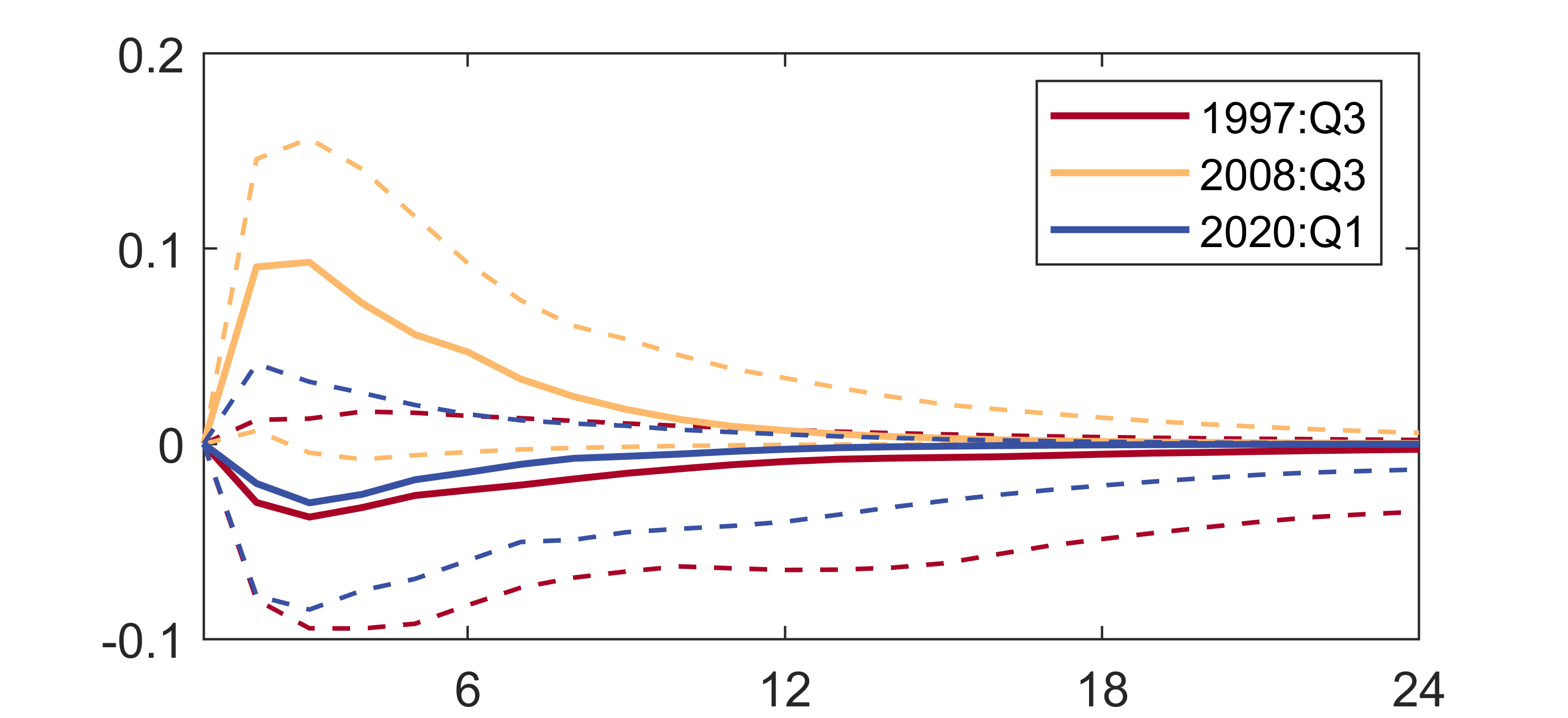

Supplement: Supplementary file 4 [file Data_Sheet_2.ZIP › KR_HK_2 (3).tif]

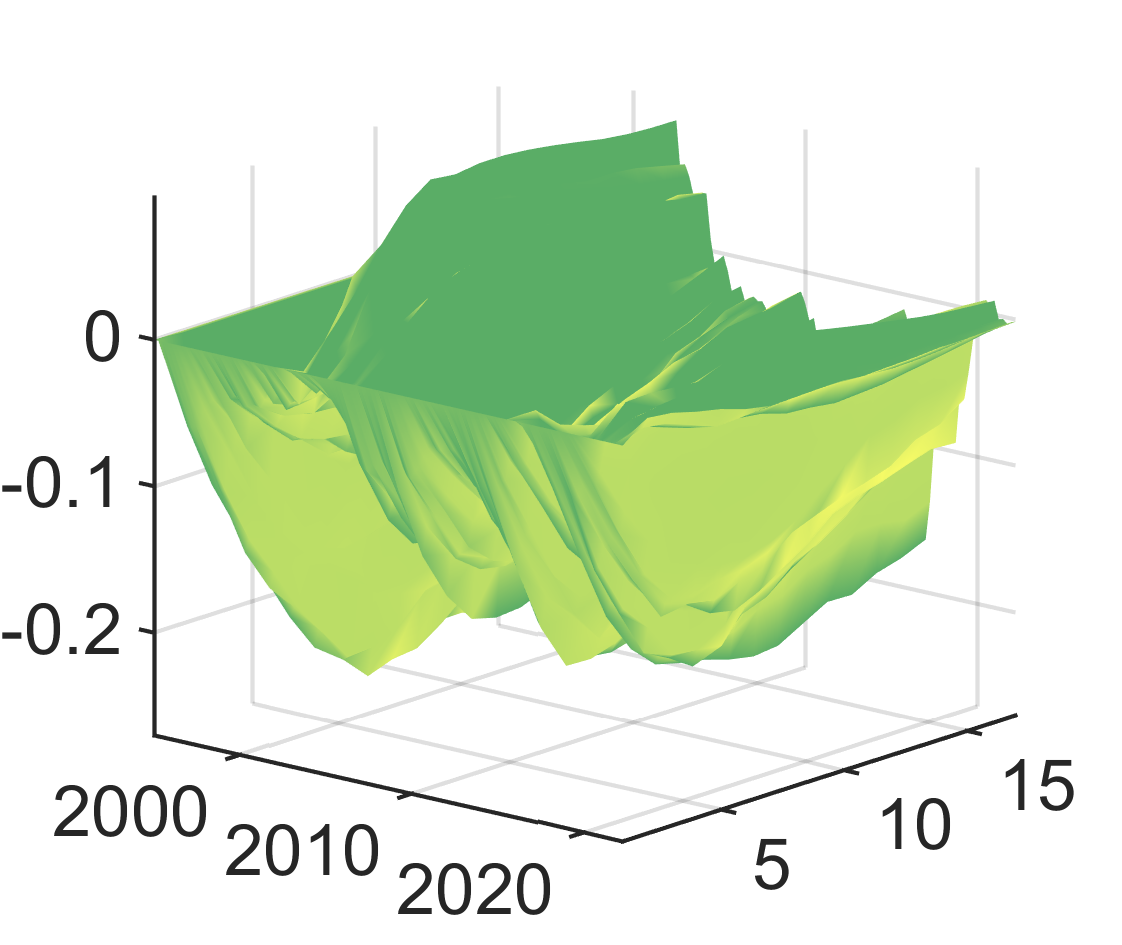

Supplement: Supplementary file 4 [file Data_Sheet_2.ZIP › KR_JPN_2 (1).tif]

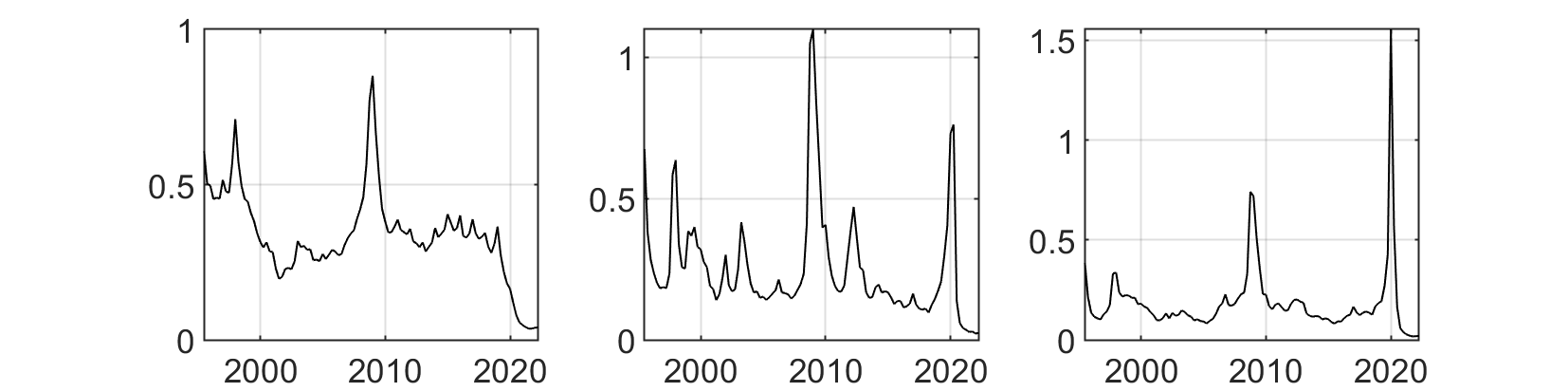

Supplement: Supplementary file 4 [file Data_Sheet_2.ZIP › KR_JPN_2 (2).tif]

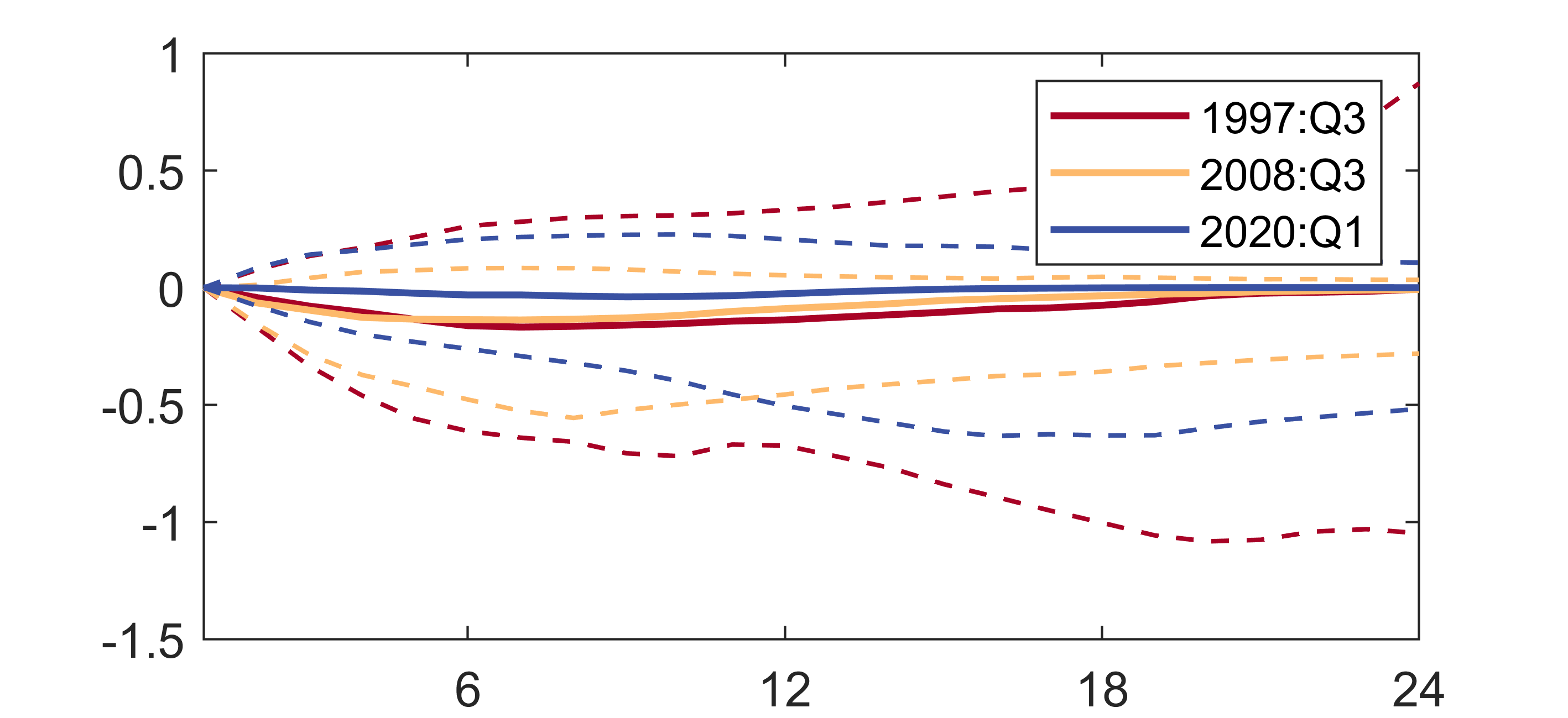

Supplement: Supplementary file 4 [file Data_Sheet_2.ZIP › KR_JPN_2 (3).tif]

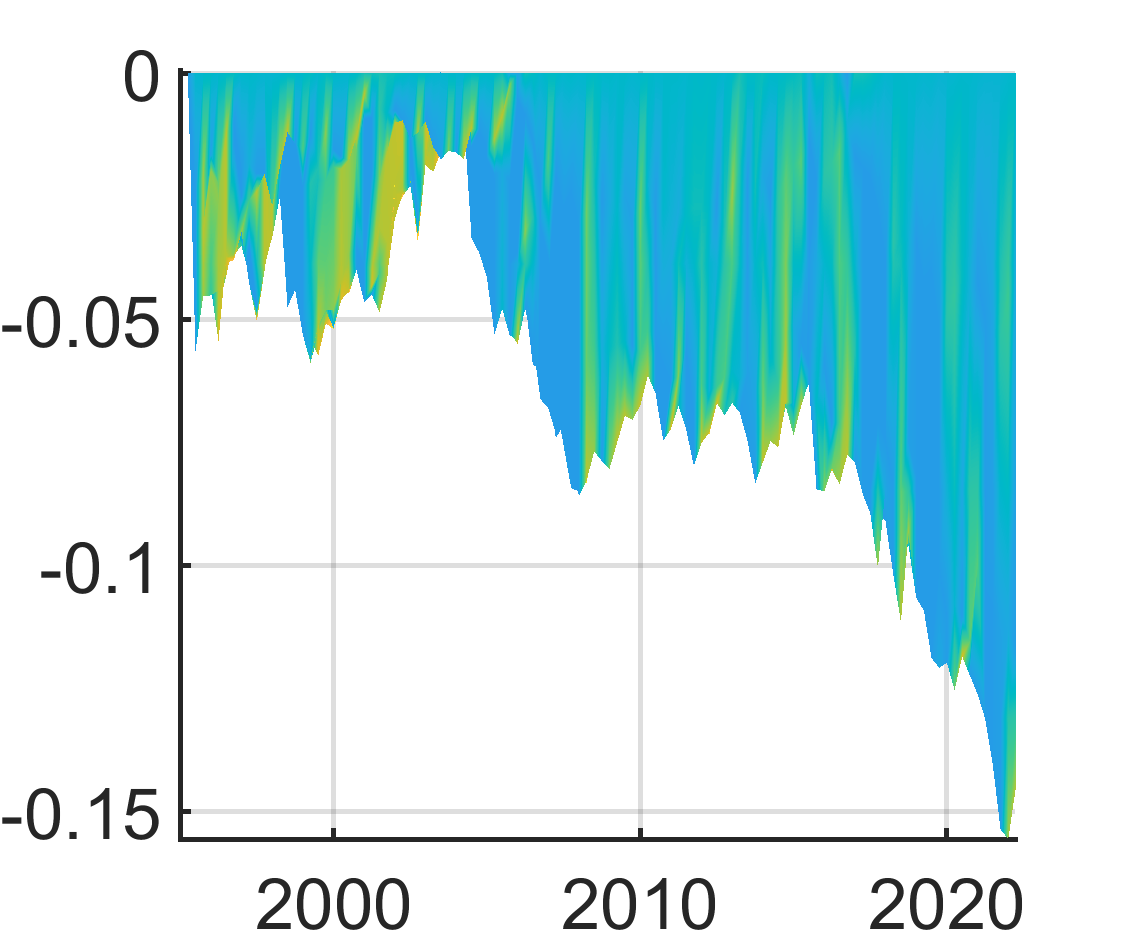

Supplement: Supplementary file 5 [file Data_Sheet_3.ZIP › BM_CHN_3 (1).tif]

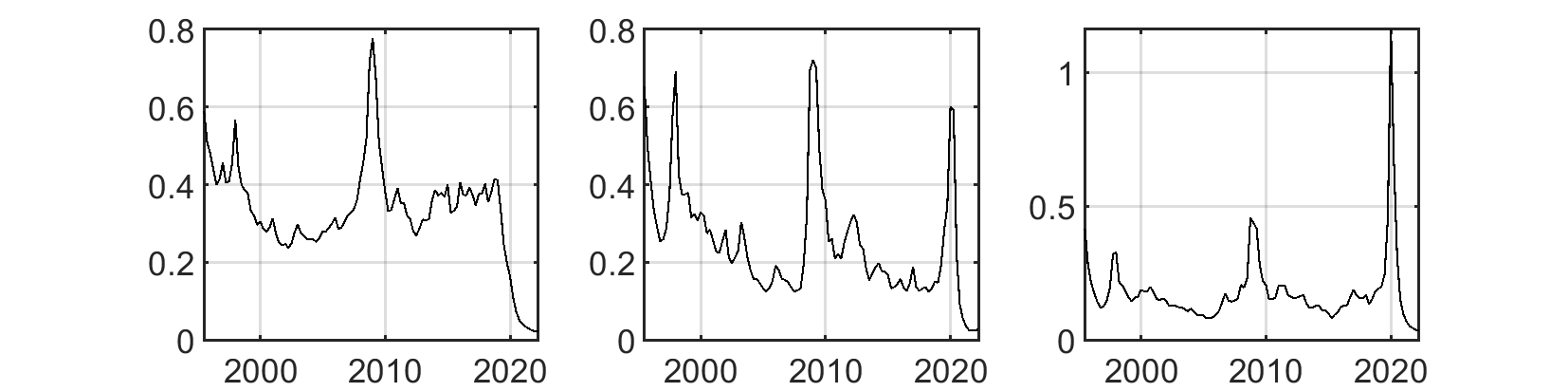

Supplement: Supplementary file 5 [file Data_Sheet_3.ZIP › BM_CHN_3 (2).tif]

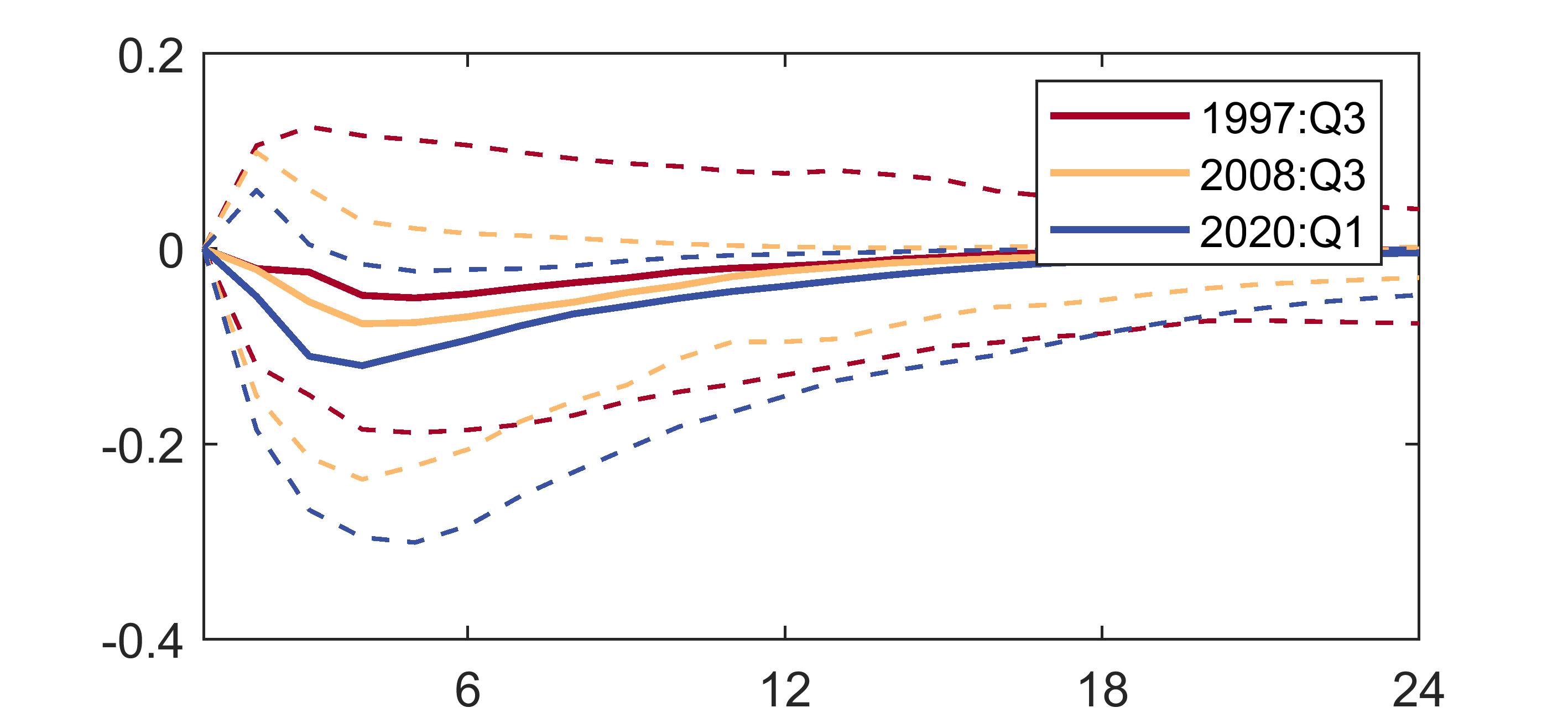

Supplement: Supplementary file 5 [file Data_Sheet_3.ZIP › BM_CHN_3 (3).tif]

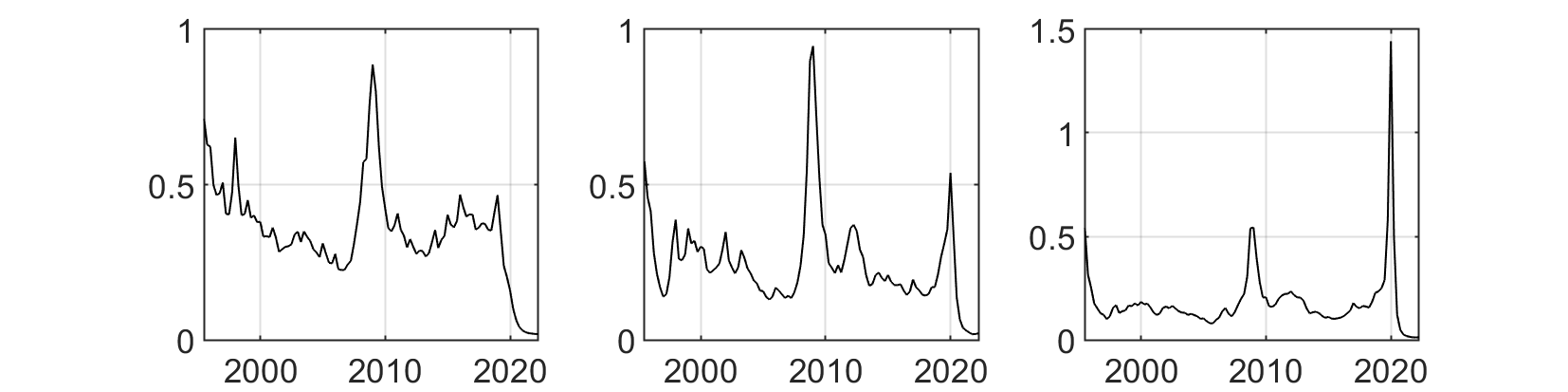

Supplement: Supplementary file 5 [file Data_Sheet_3.ZIP › CHN_HK_3 (2).tif]

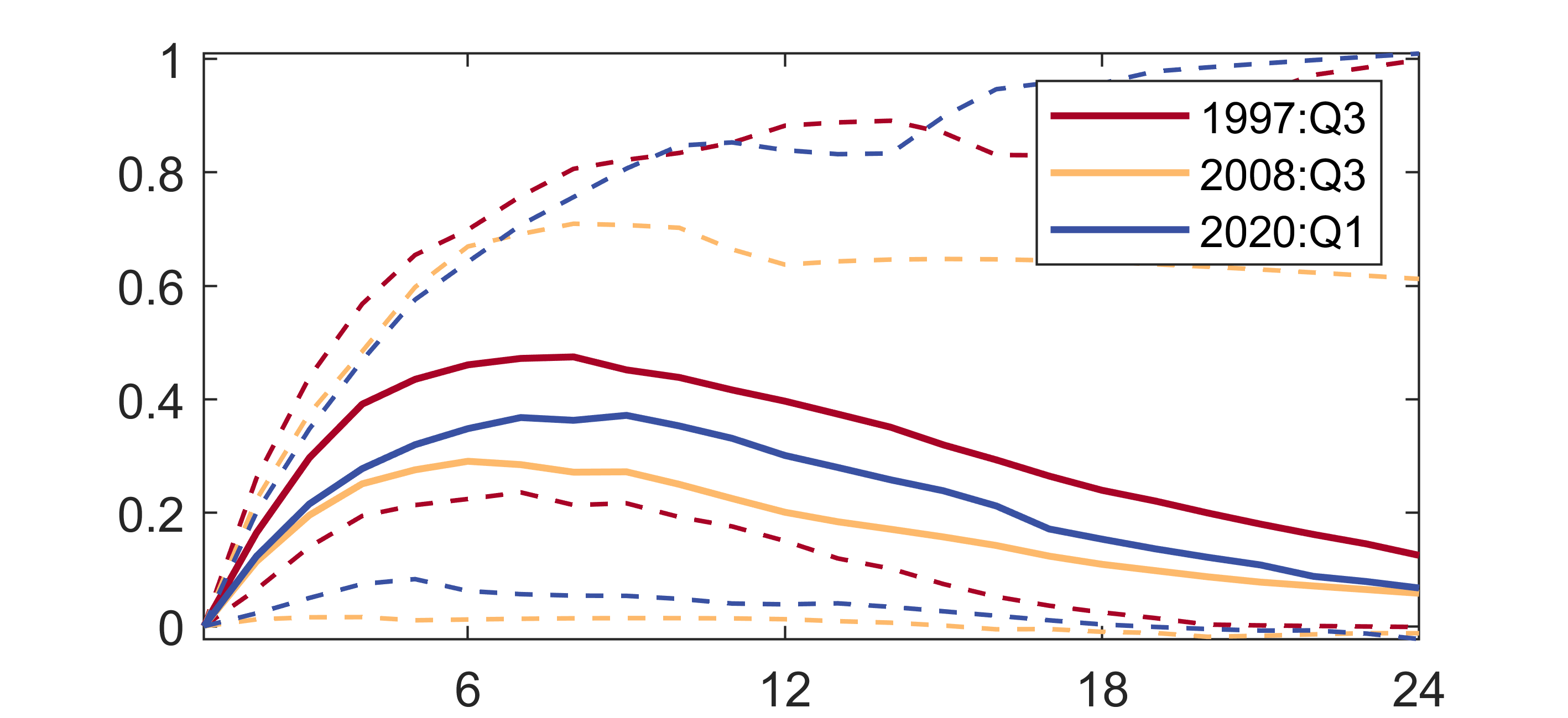

Supplement: Supplementary file 5 [file Data_Sheet_3.ZIP › CHN_HK_3 (3).tif]

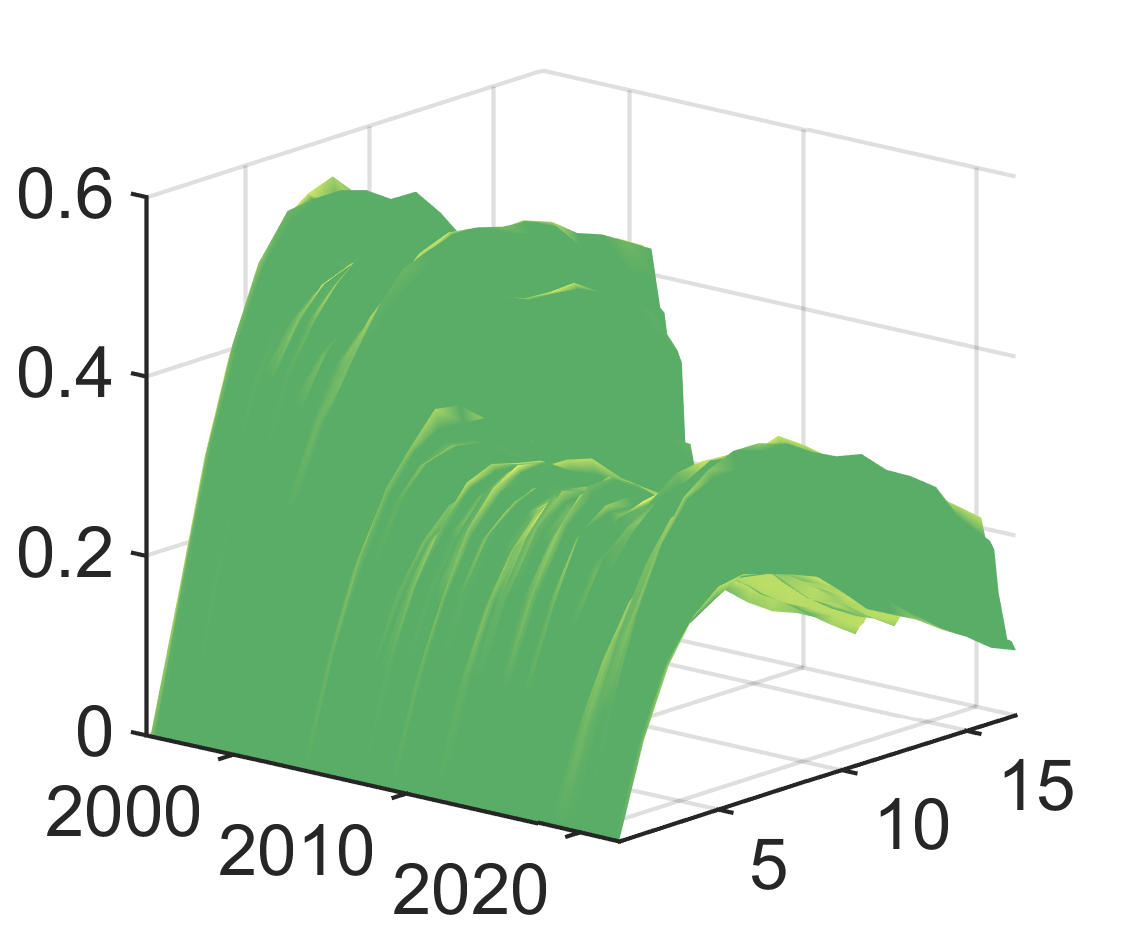

Supplement: Supplementary file 5 [file Data_Sheet_3.ZIP › CHN_HK_3 (4).tif]

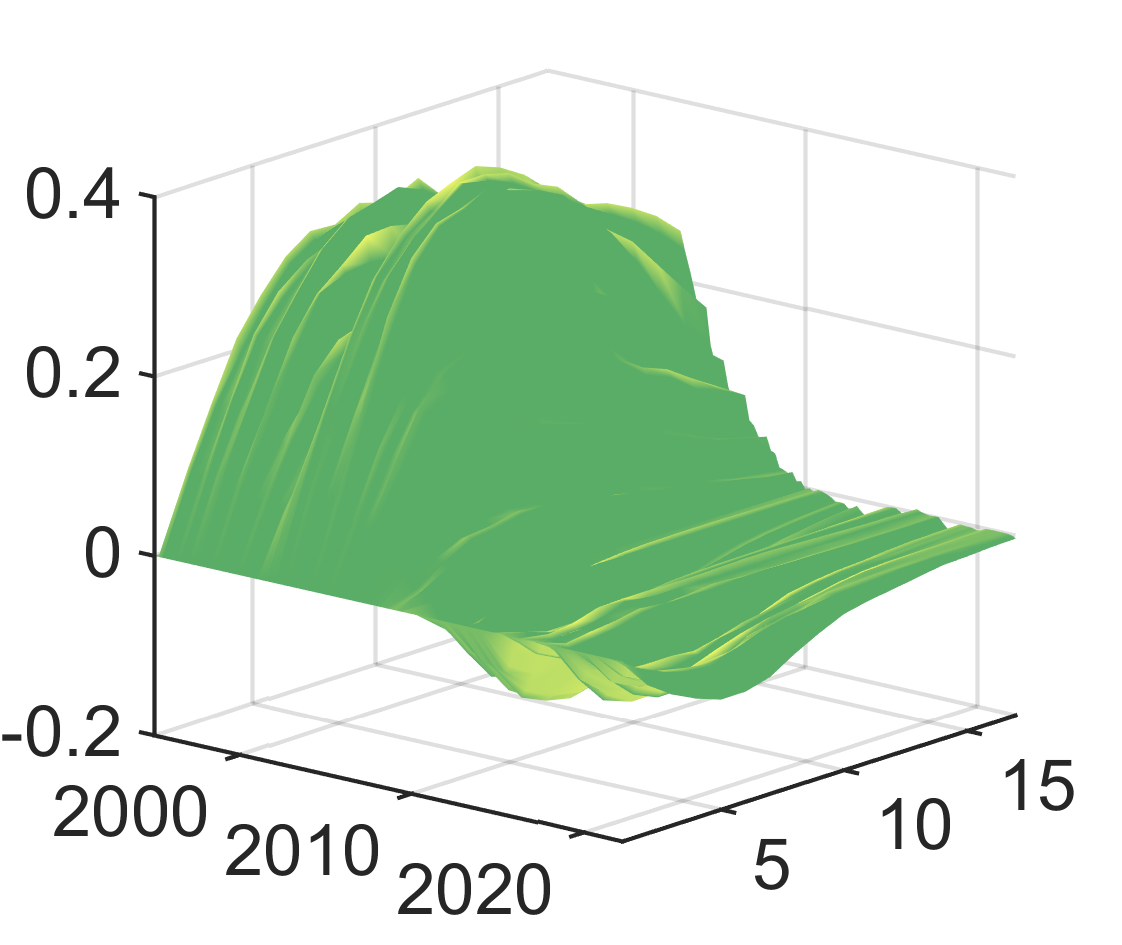

Supplement: Supplementary file 5 [file Data_Sheet_3.ZIP › CHN_JPN_3 (1).tif]

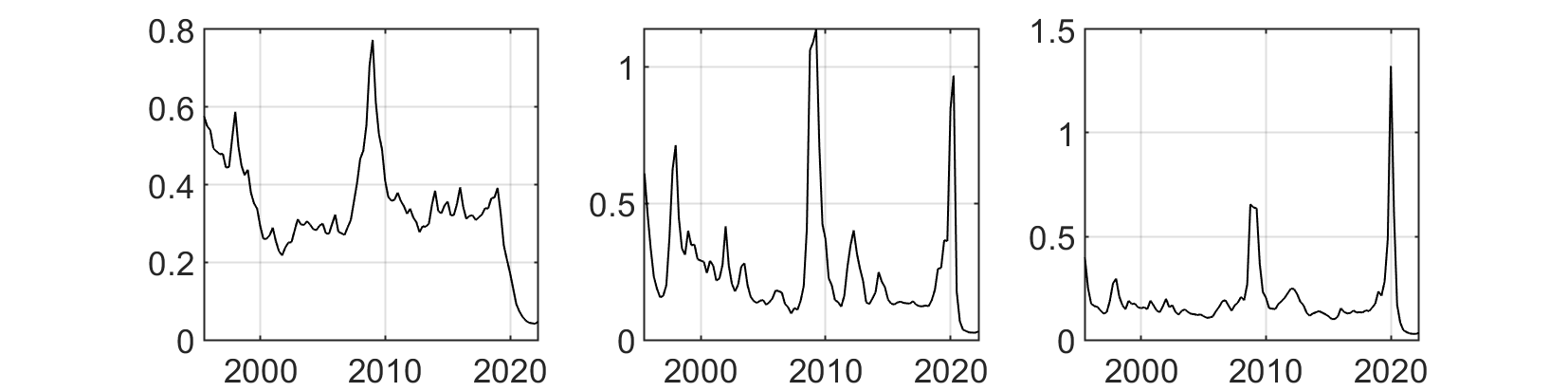

Supplement: Supplementary file 5 [file Data_Sheet_3.ZIP › CHN_JPN_3 (2).tif]

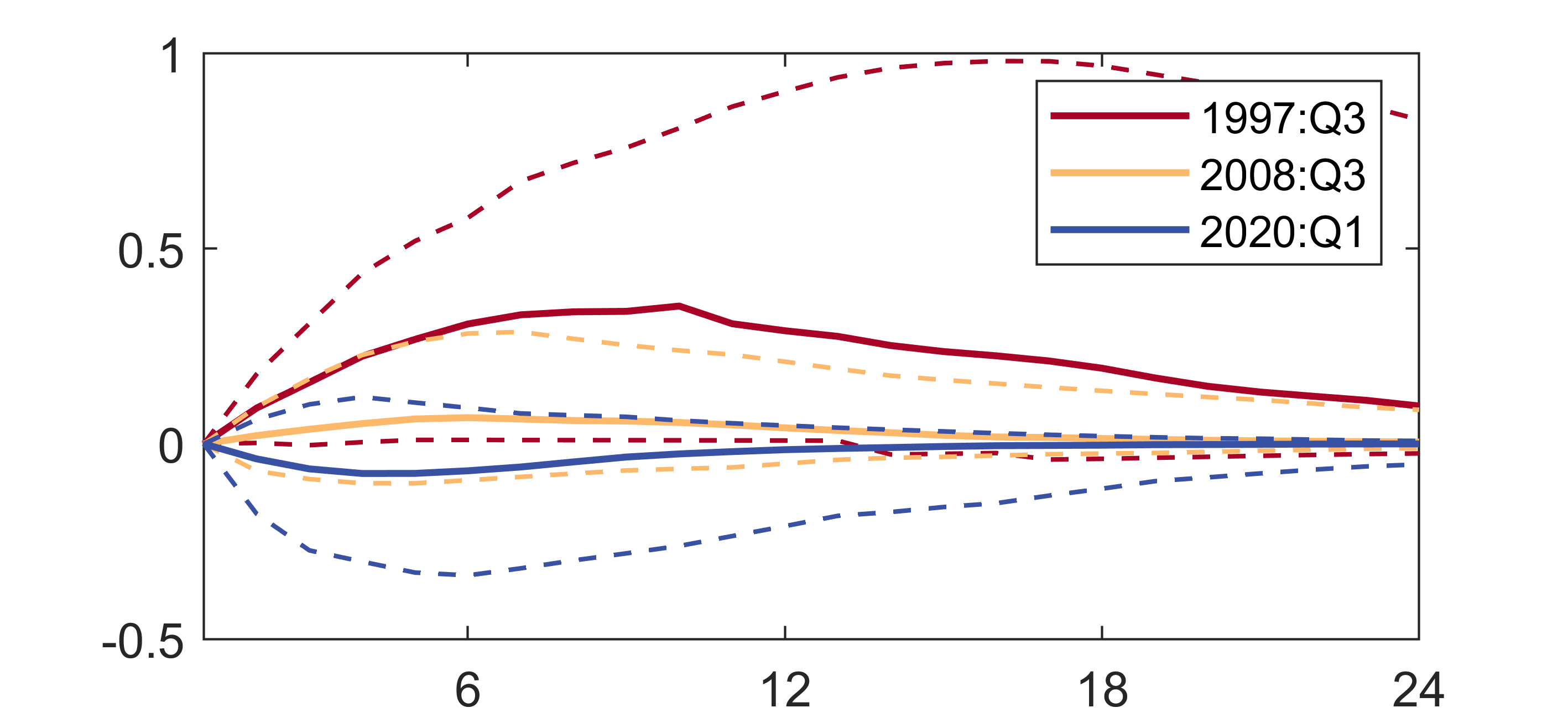

Supplement: Supplementary file 5 [file Data_Sheet_3.ZIP › CHN_JPN_3 (3).tif]

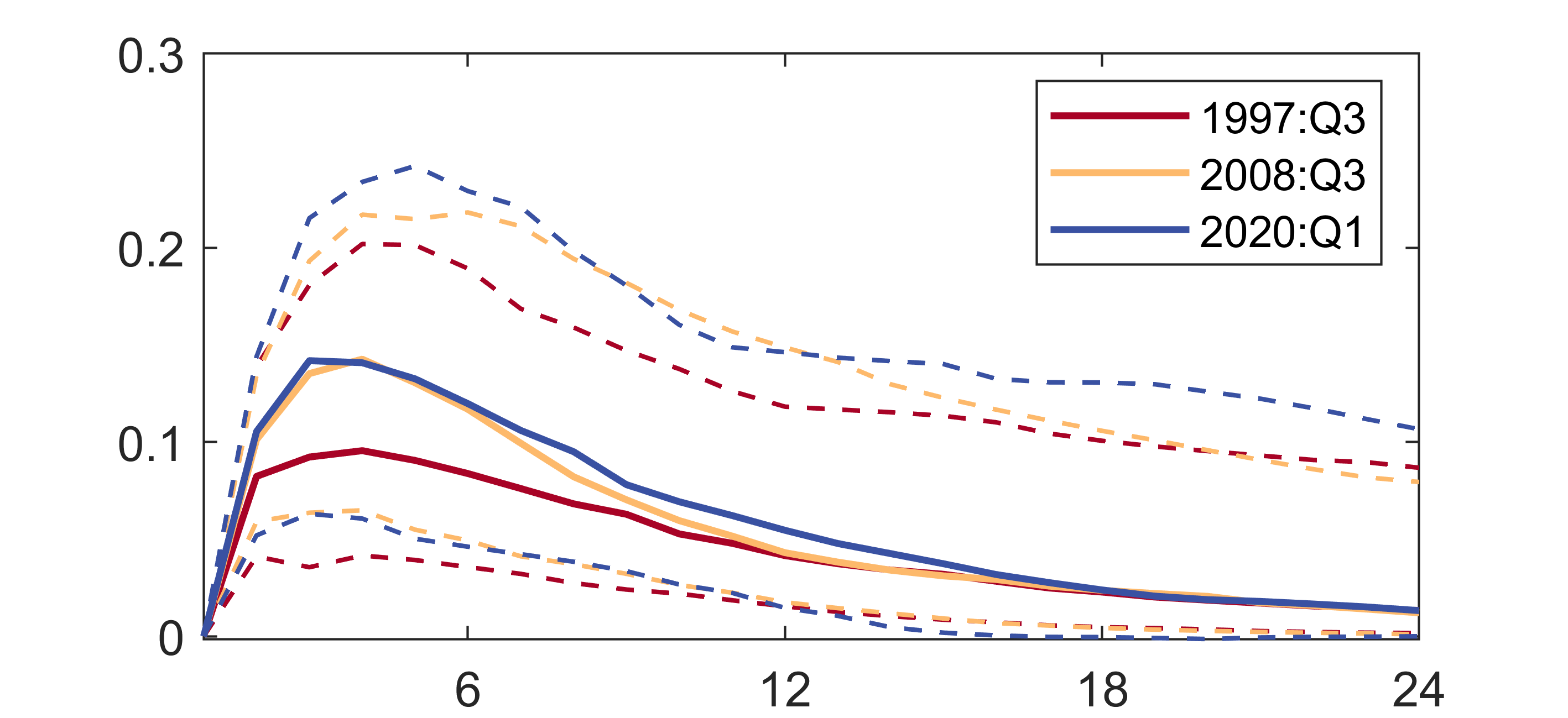

Supplement: Supplementary file 5 [file Data_Sheet_3.ZIP › CHN_KR_3 (1).tif]

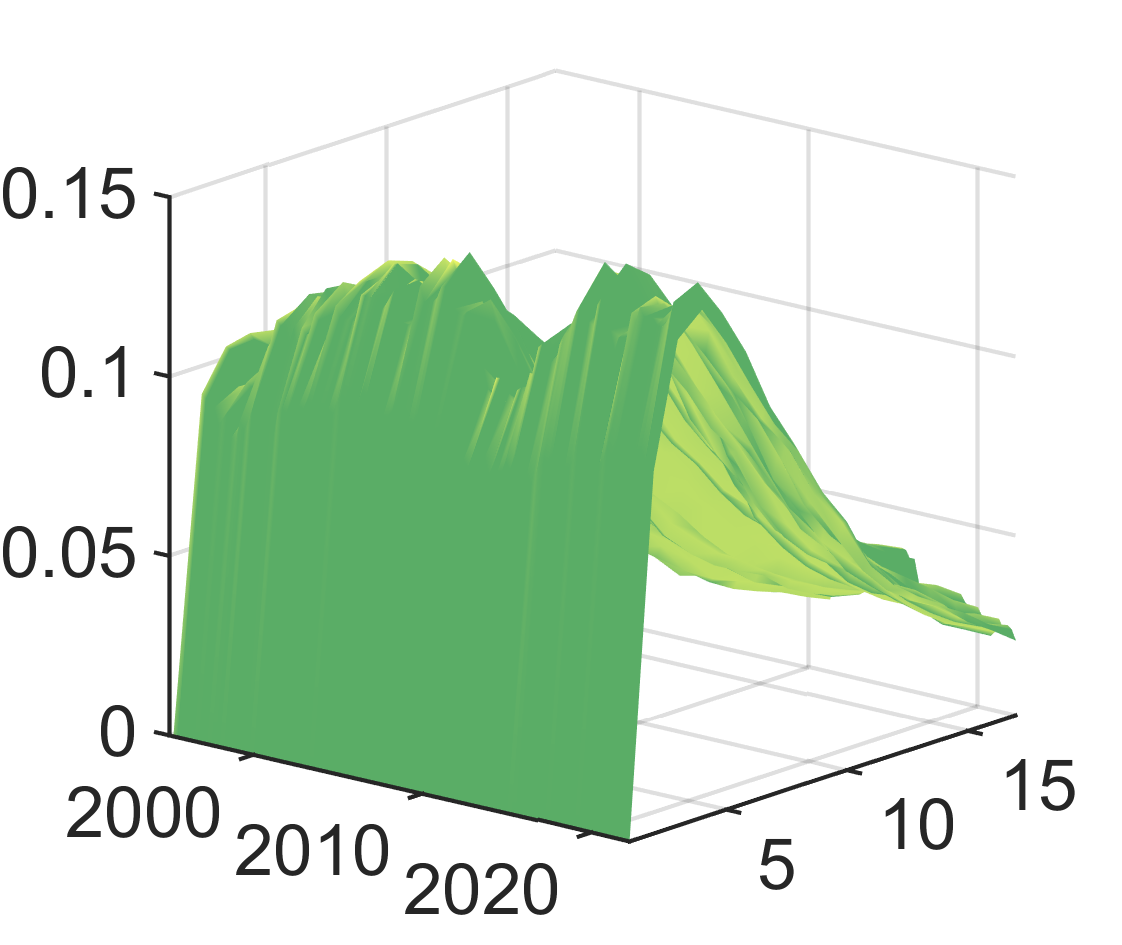

Supplement: Supplementary file 5 [file Data_Sheet_3.ZIP › CHN_KR_3 (2).tif]

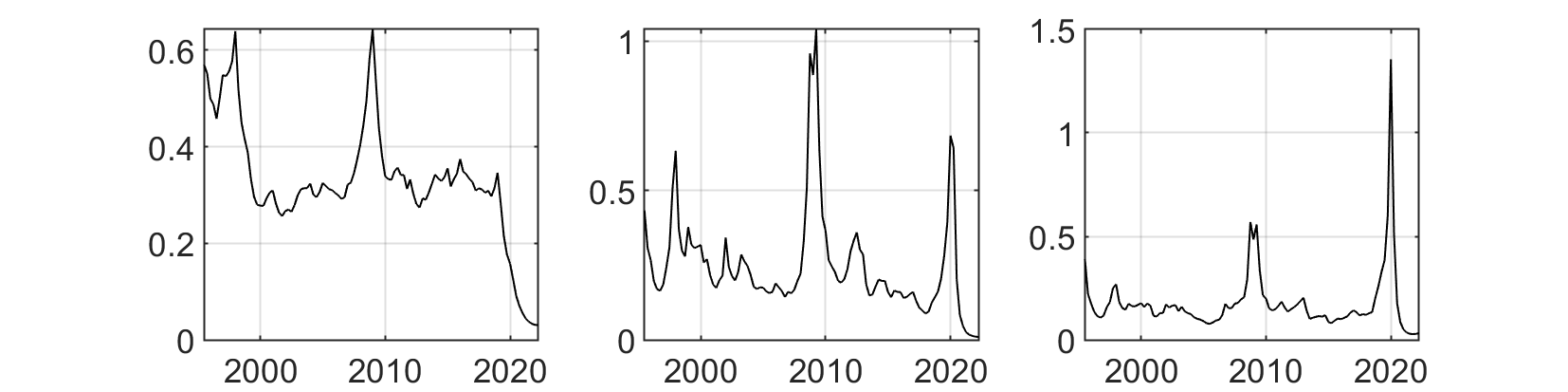

Supplement: Supplementary file 5 [file Data_Sheet_3.ZIP › CHN_KR_3 (3).tif]

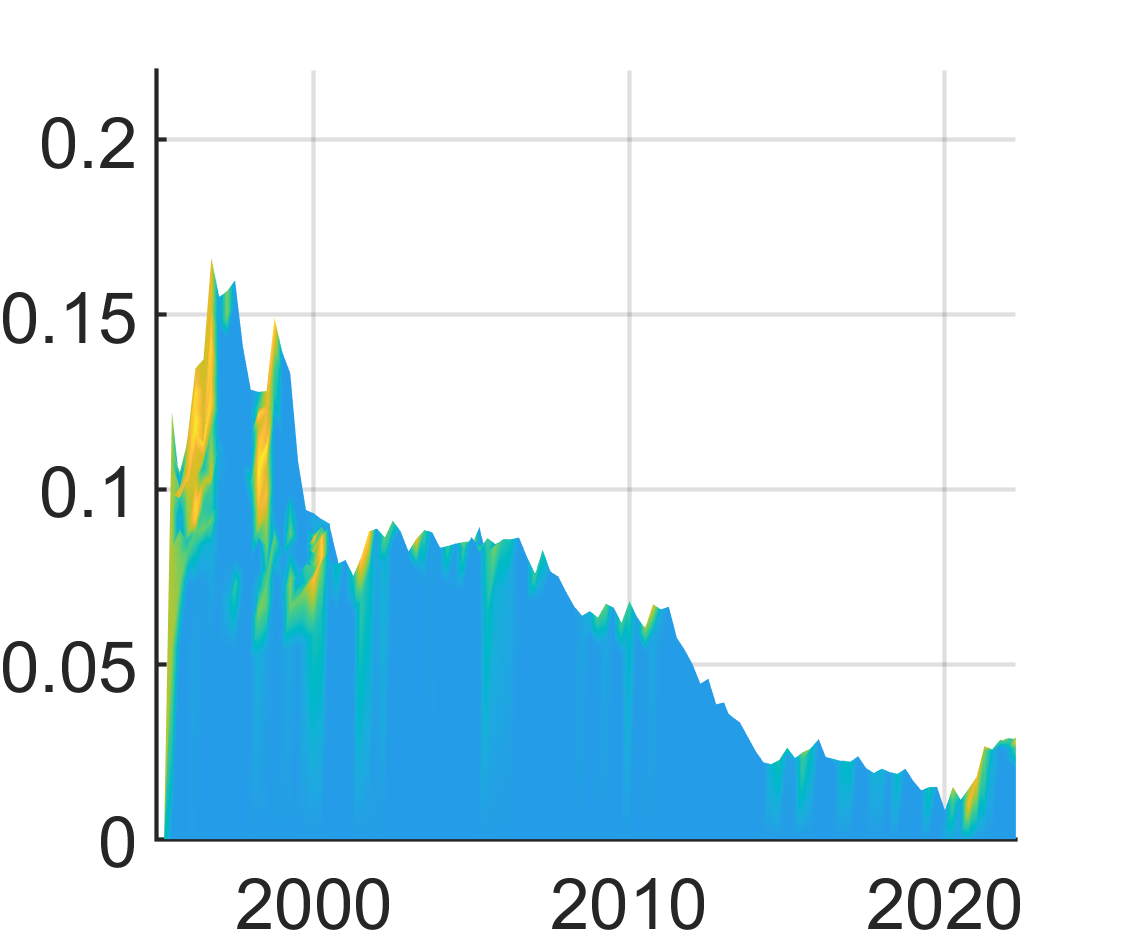

Supplement: Supplementary file 5 [file Data_Sheet_3.ZIP › CM_CHN_3 (1).tif]

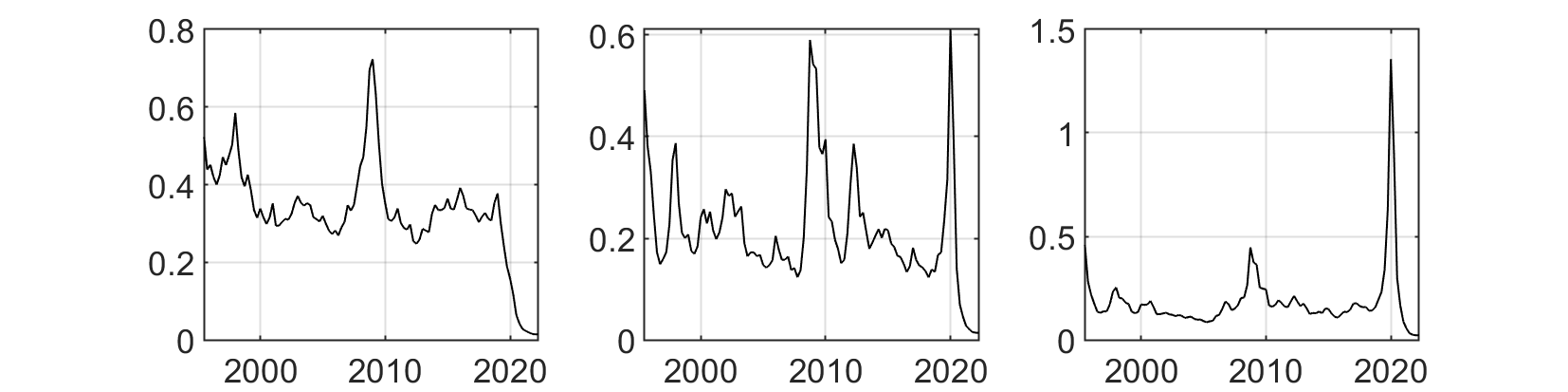

Supplement: Supplementary file 5 [file Data_Sheet_3.ZIP › CM_CHN_3 (2).tif]

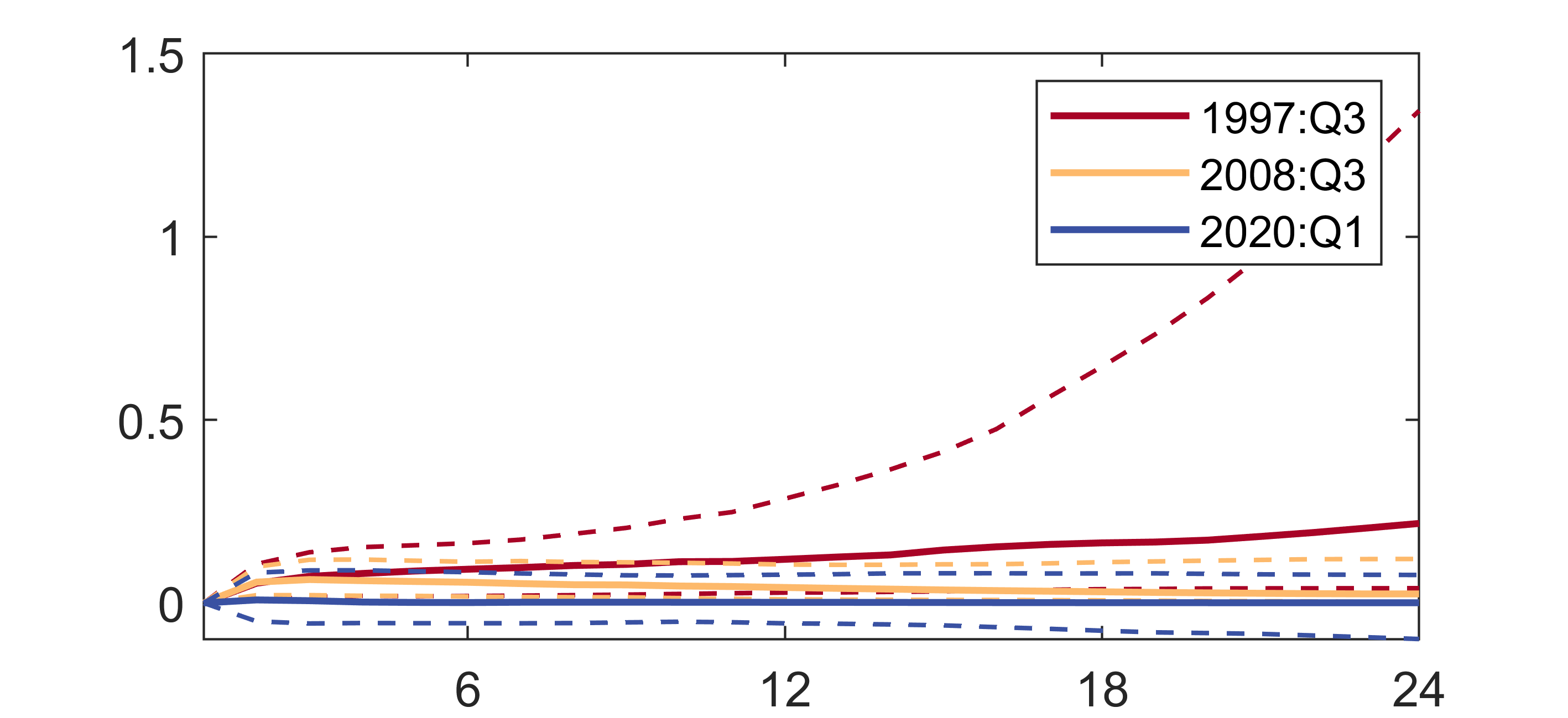

Supplement: Supplementary file 5 [file Data_Sheet_3.ZIP › CM_CHN_3 (3).tif]

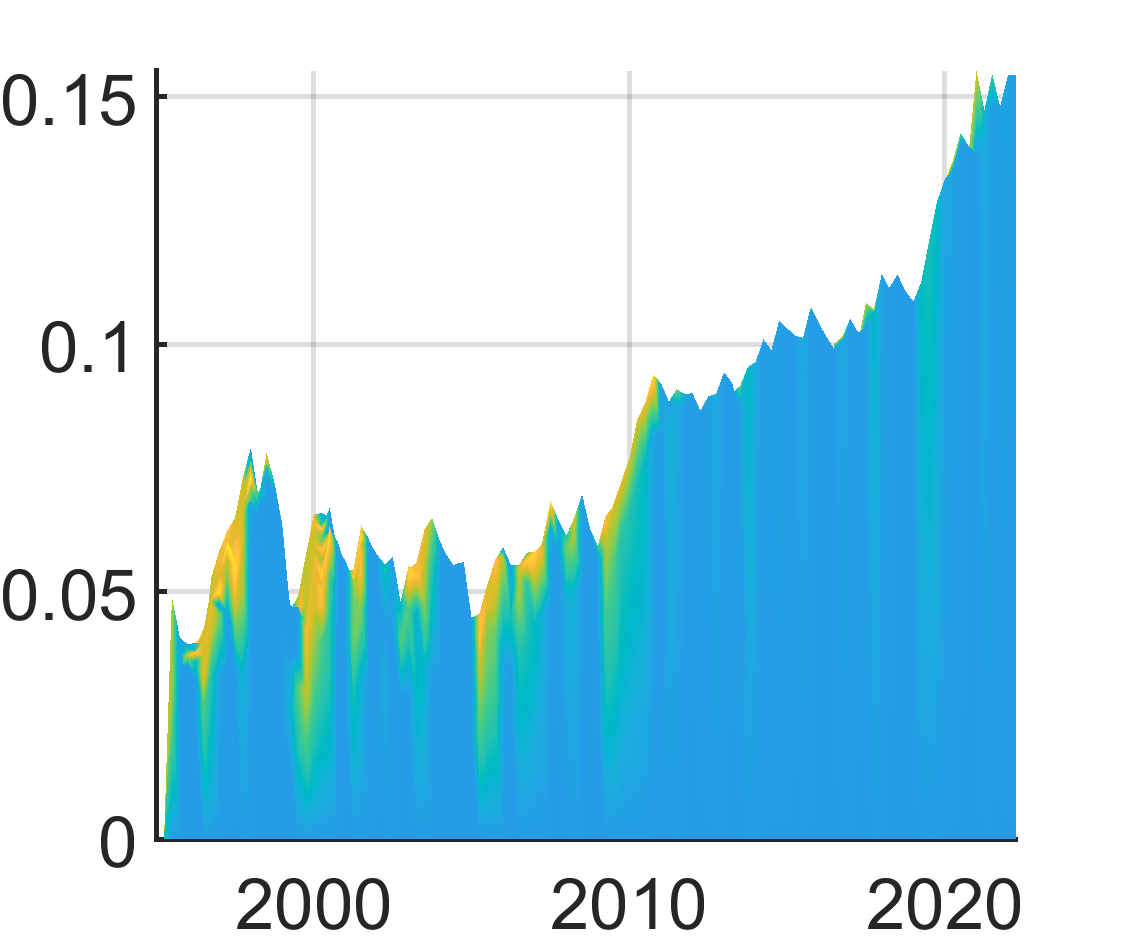

Supplement: Supplementary file 5 [file Data_Sheet_3.ZIP › COM_CHN_3 (1).tif]

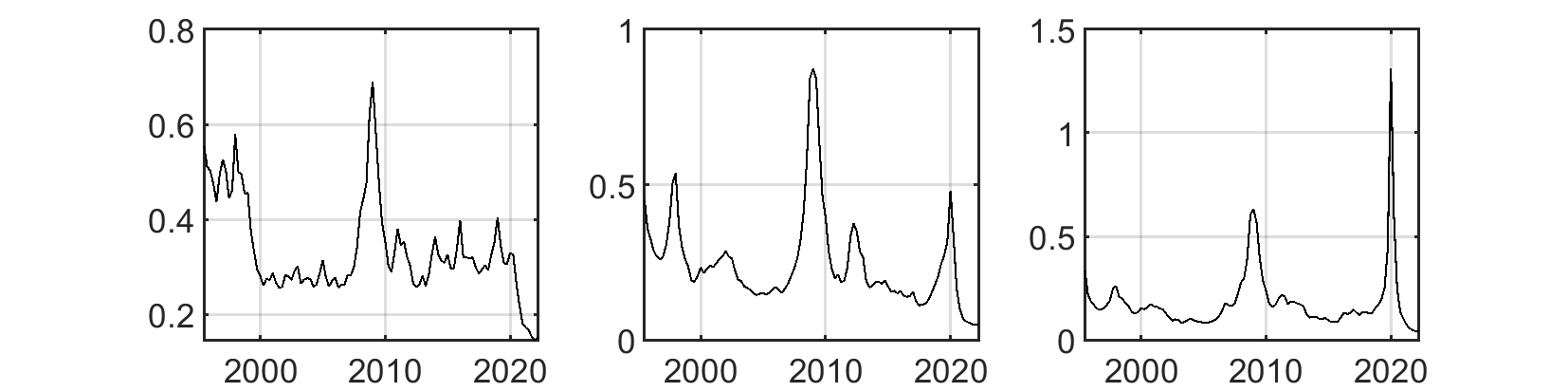

Supplement: Supplementary file 5 [file Data_Sheet_3.ZIP › COM_CHN_3 (2).tif]

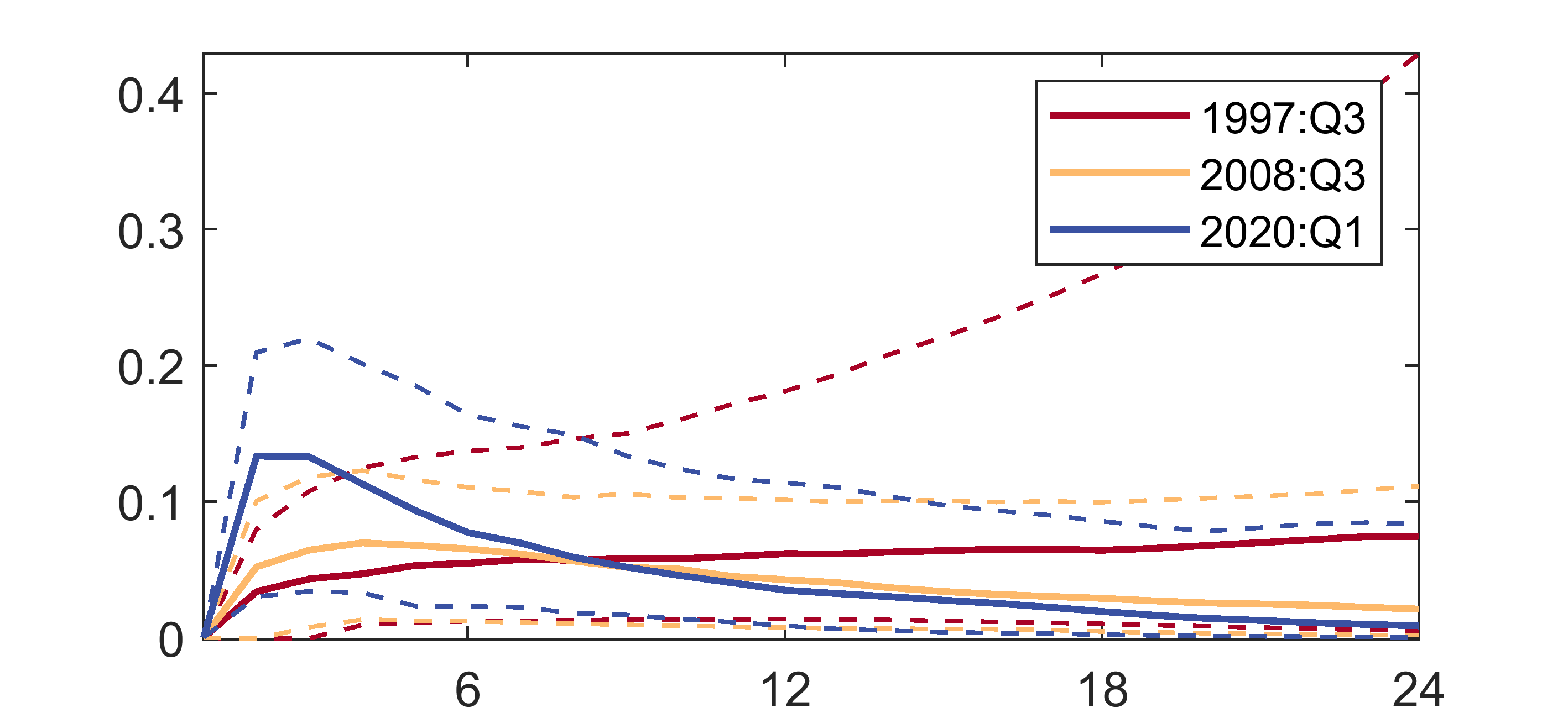

Supplement: Supplementary file 5 [file Data_Sheet_3.ZIP › COM_CHN_3 (3).tif]

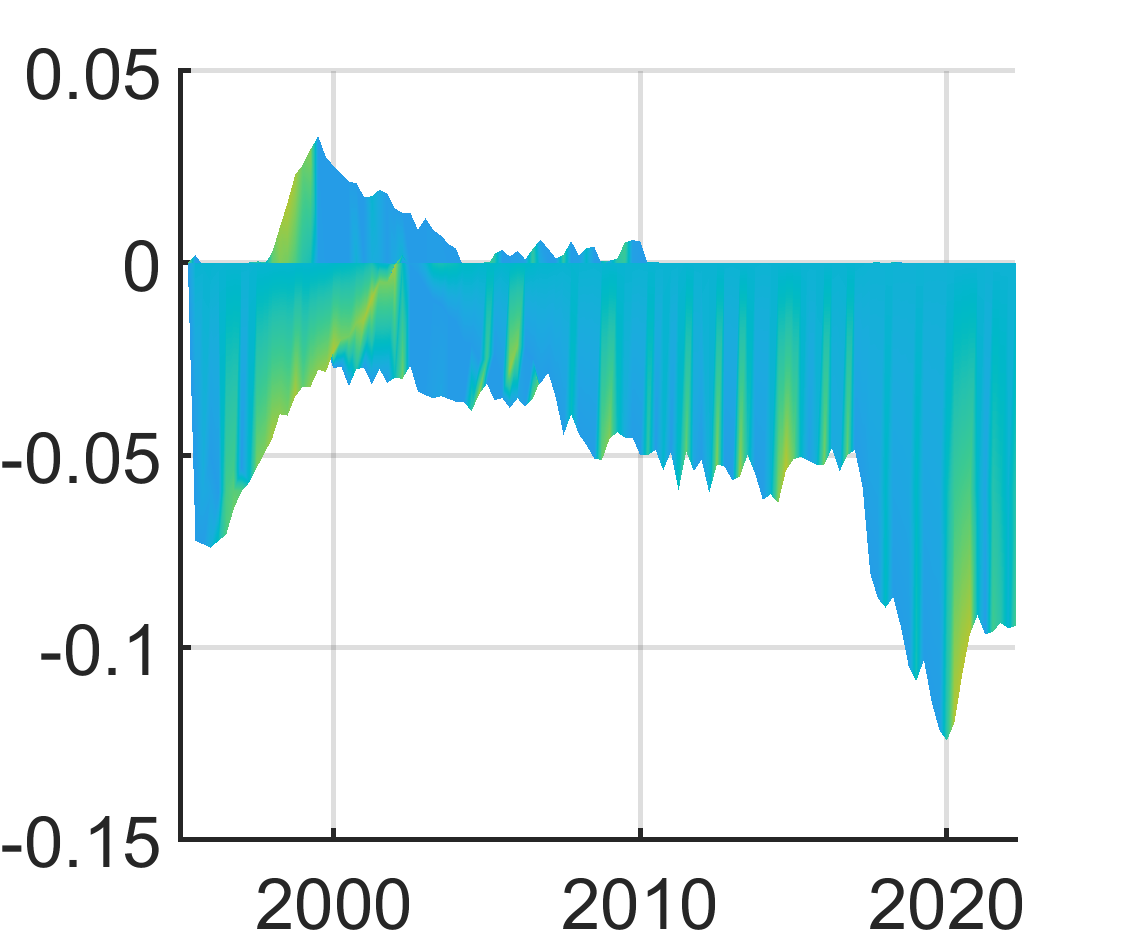

Supplement: Supplementary file 5 [file Data_Sheet_3.ZIP › FEM_CHN_3 (1).tif]

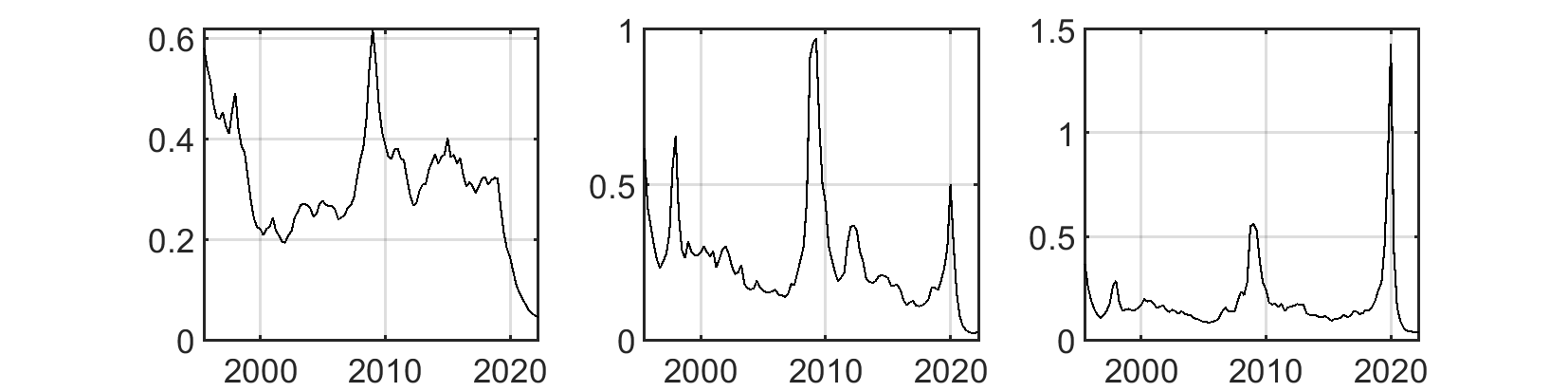

Supplement: Supplementary file 5 [file Data_Sheet_3.ZIP › FEM_CHN_3 (2).tif]

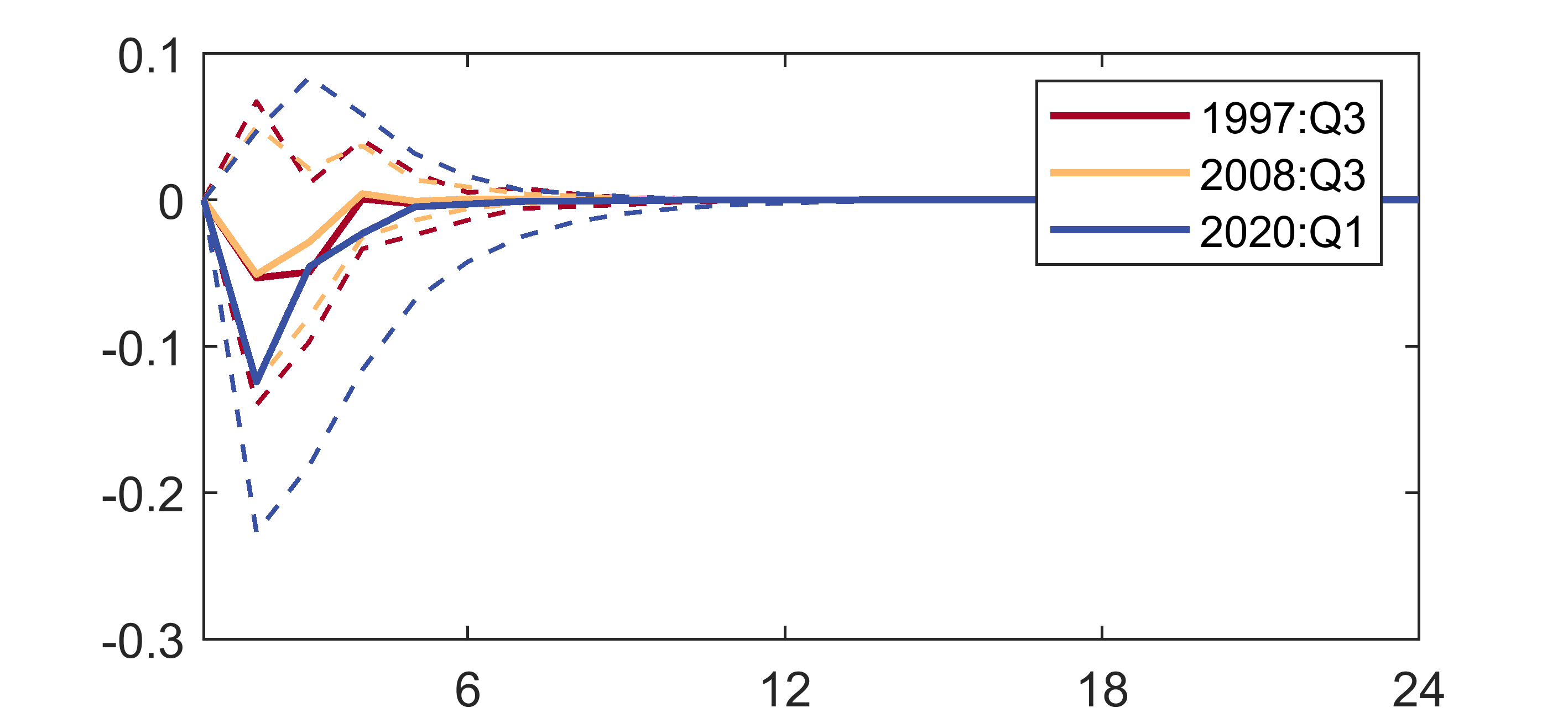

Supplement: Supplementary file 5 [file Data_Sheet_3.ZIP › FEM_CHN_3 (3).tif]

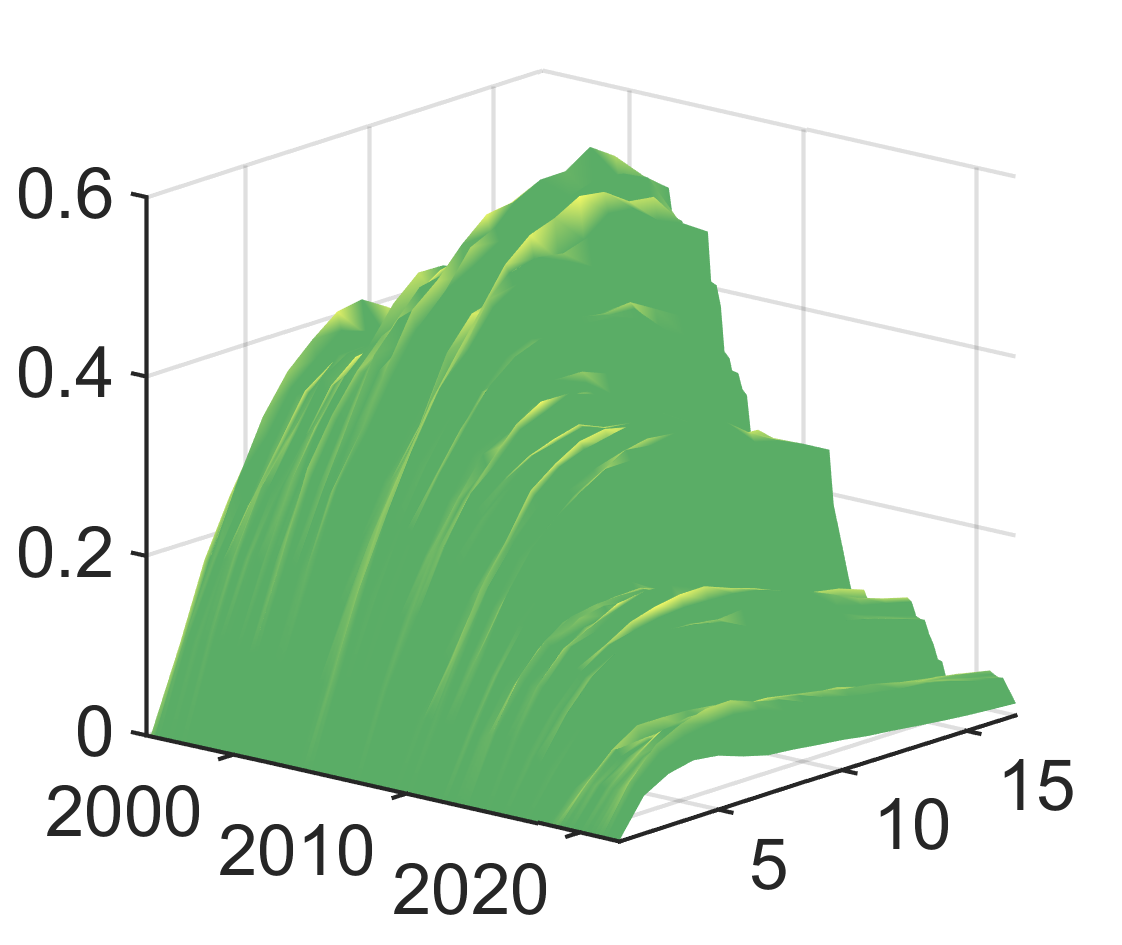

Supplement: Supplementary file 5 [file Data_Sheet_3.ZIP › HK_CHN_3(1).tif]

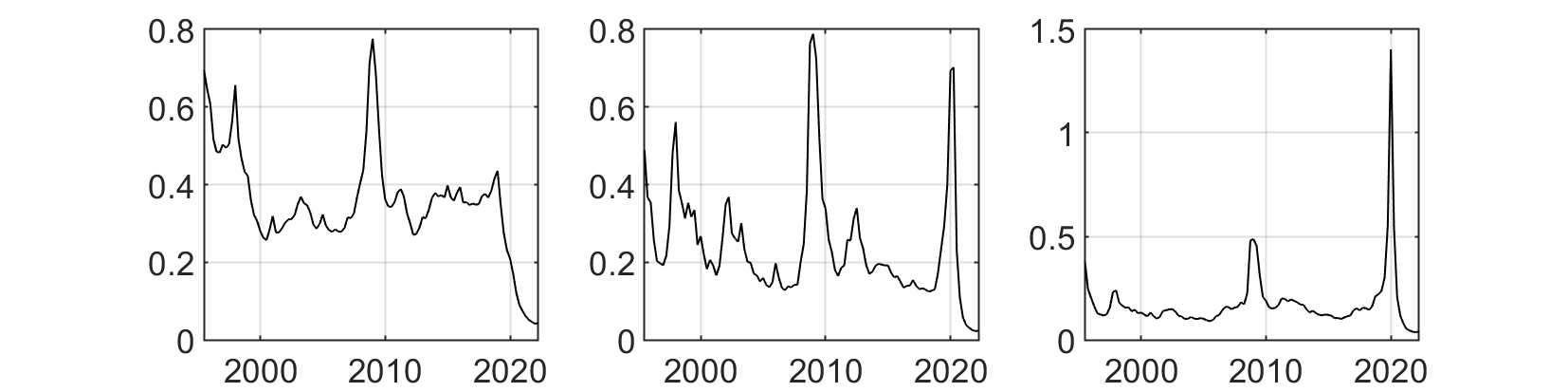

Supplement: Supplementary file 5 [file Data_Sheet_3.ZIP › HK_CHN_3(2).tif]

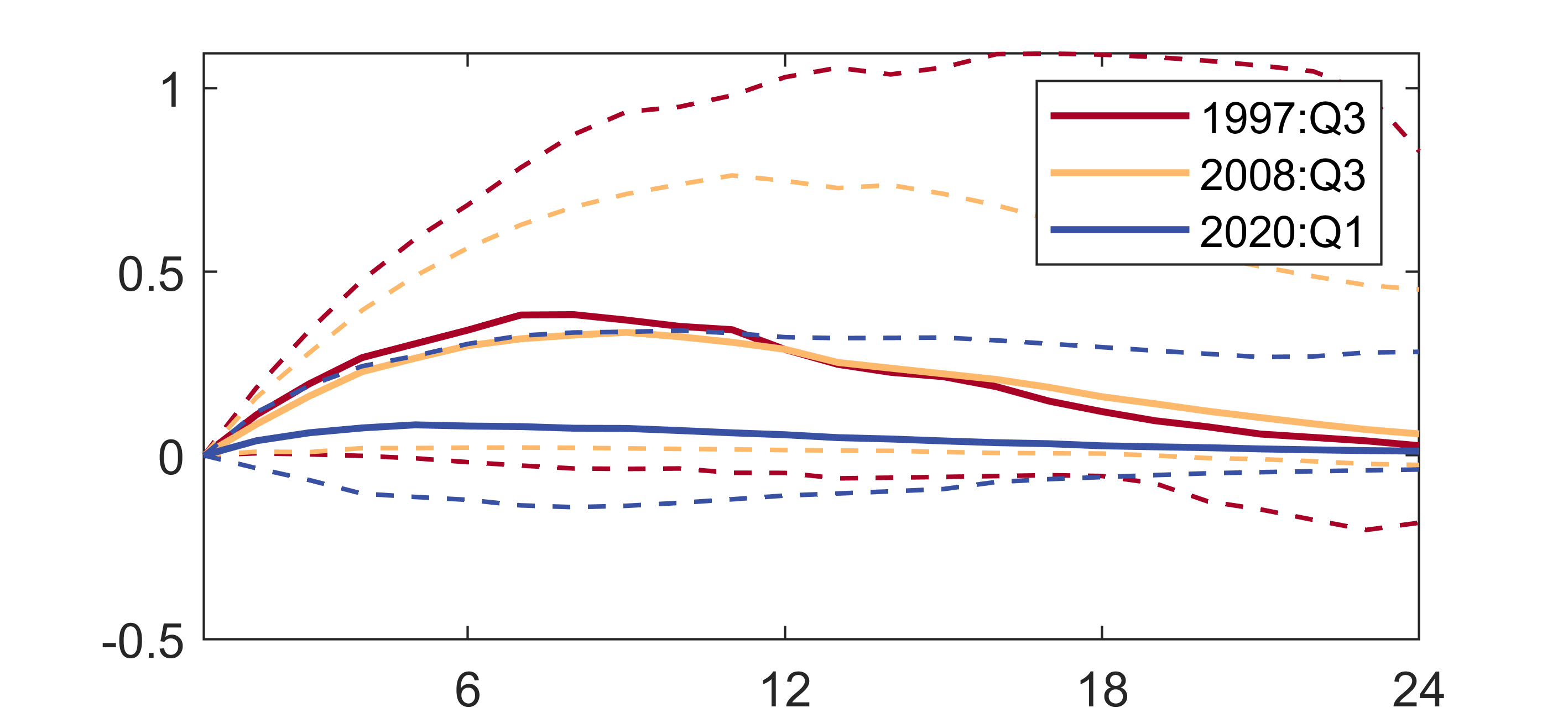

Supplement: Supplementary file 5 [file Data_Sheet_3.ZIP › HK_CHN_3(3).tif]

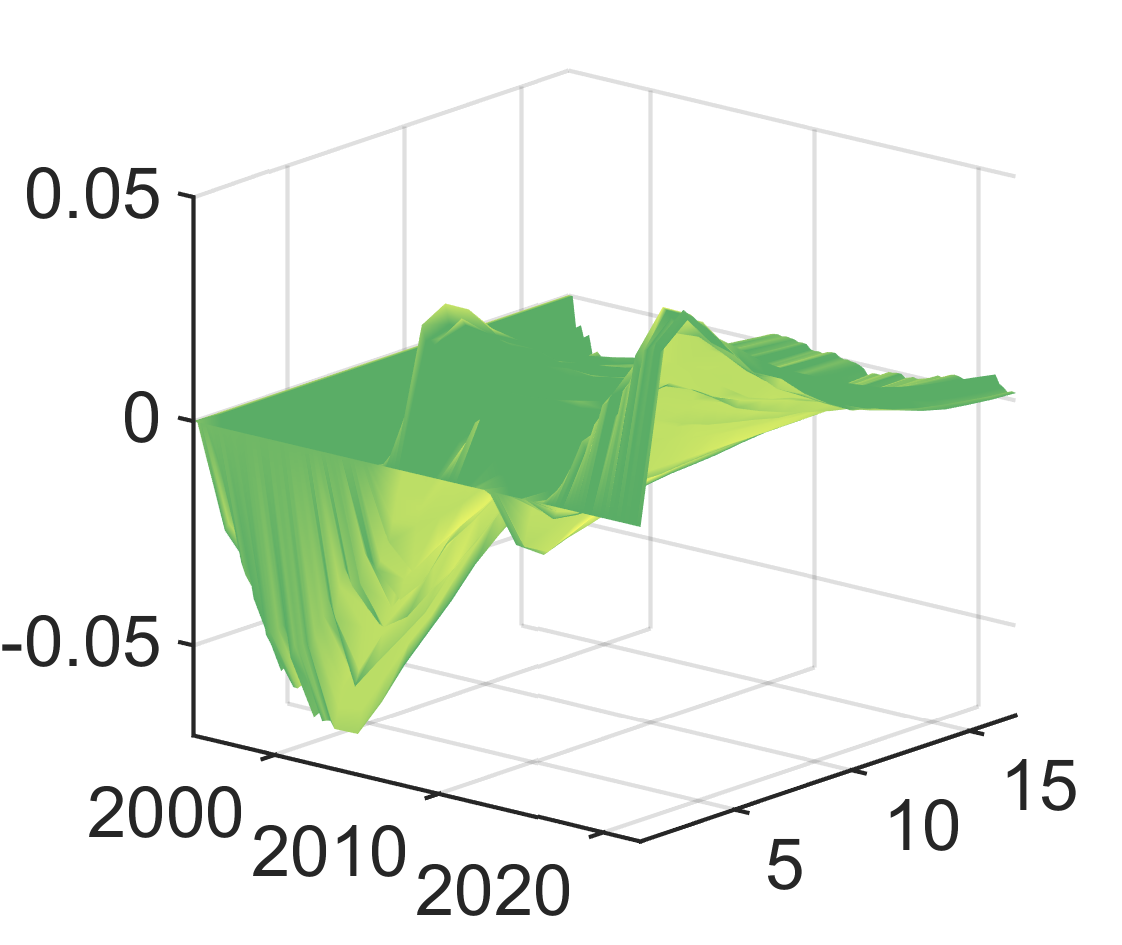

Supplement: Supplementary file 5 [file Data_Sheet_3.ZIP › JPN_CHN_3 (1).tif]

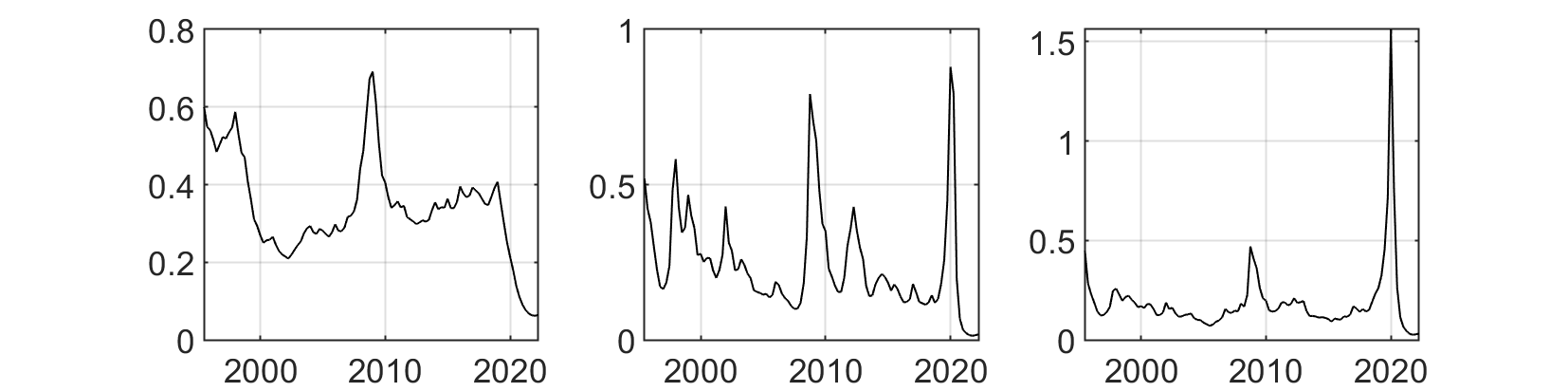

Supplement: Supplementary file 5 [file Data_Sheet_3.ZIP › JPN_CHN_3 (2).tif]

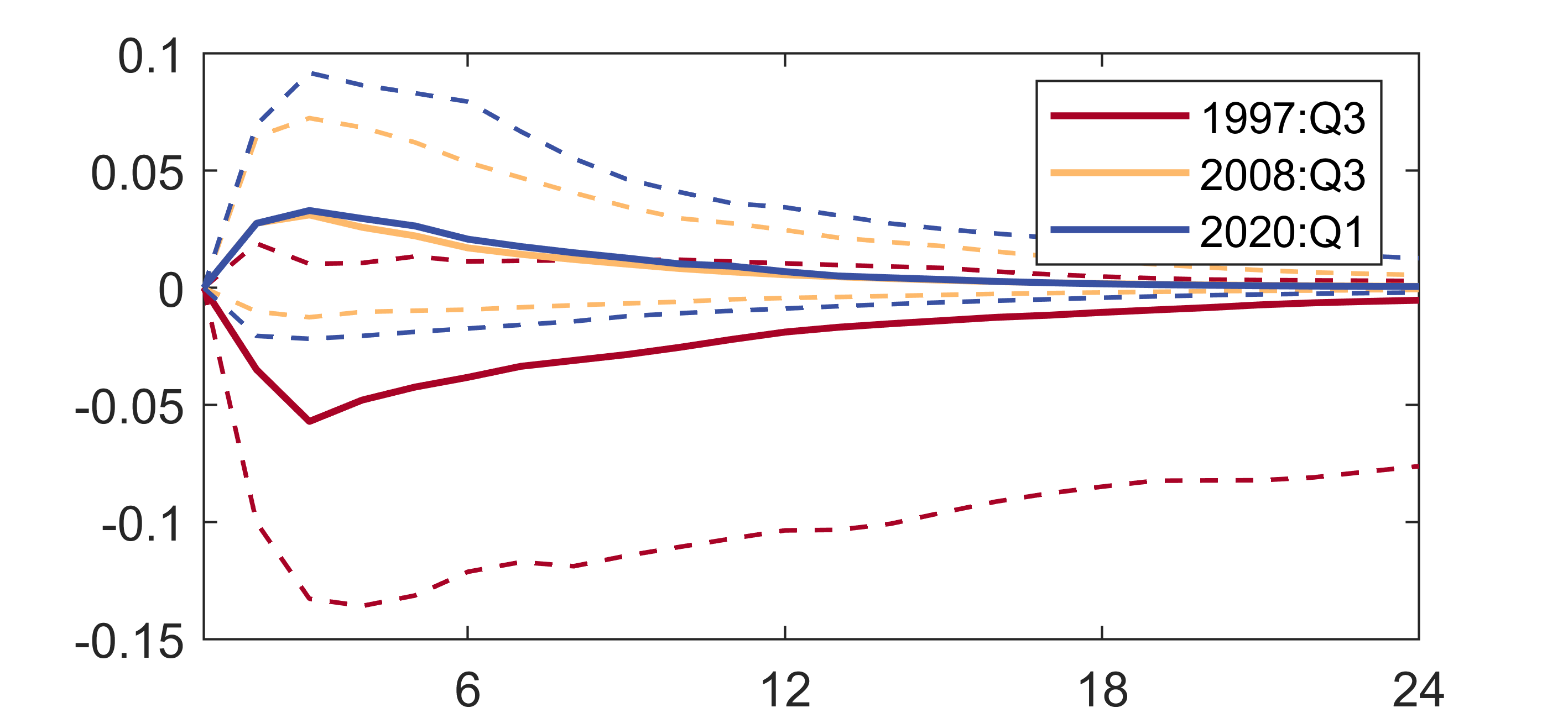

Supplement: Supplementary file 5 [file Data_Sheet_3.ZIP › JPN_CHN_3 (3).tif]

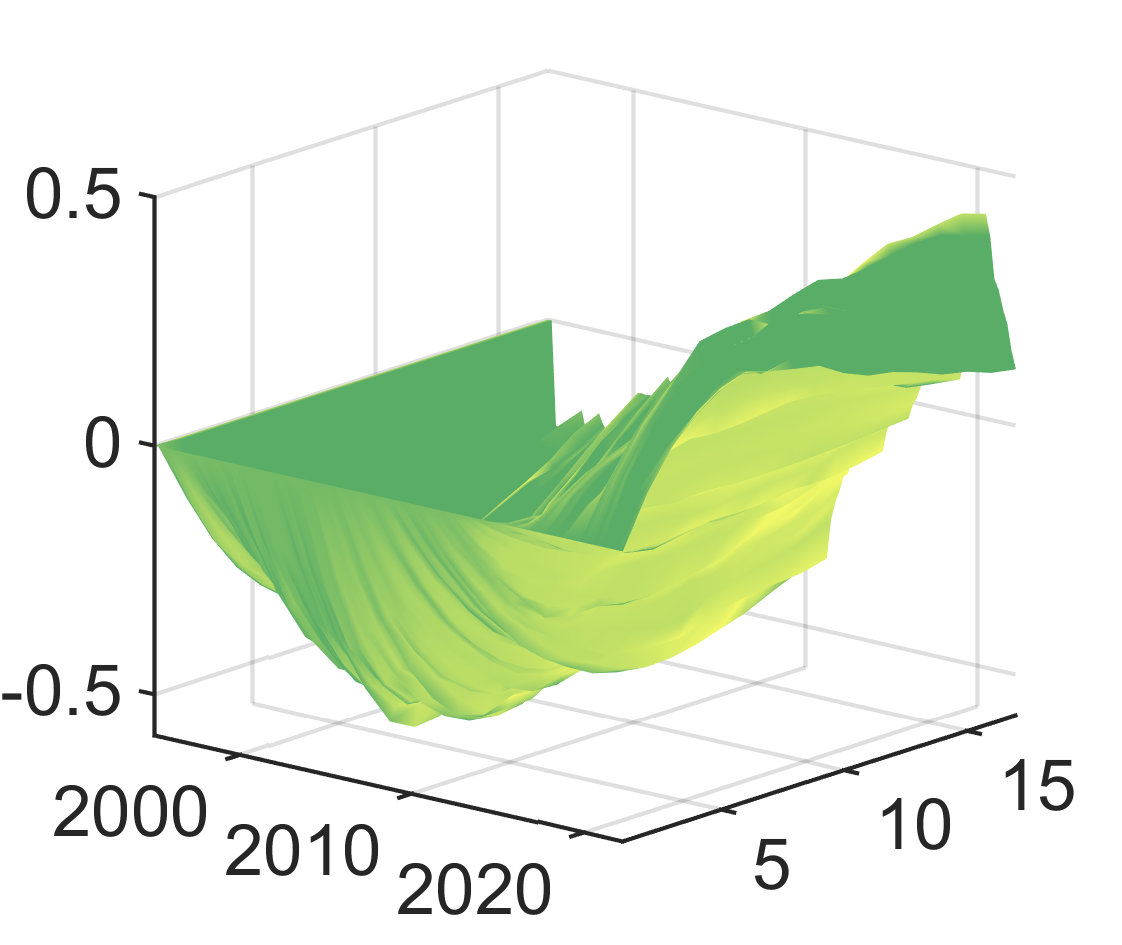

Supplement: Supplementary file 5 [file Data_Sheet_3.ZIP › KR_CHN_3 (1).tif]

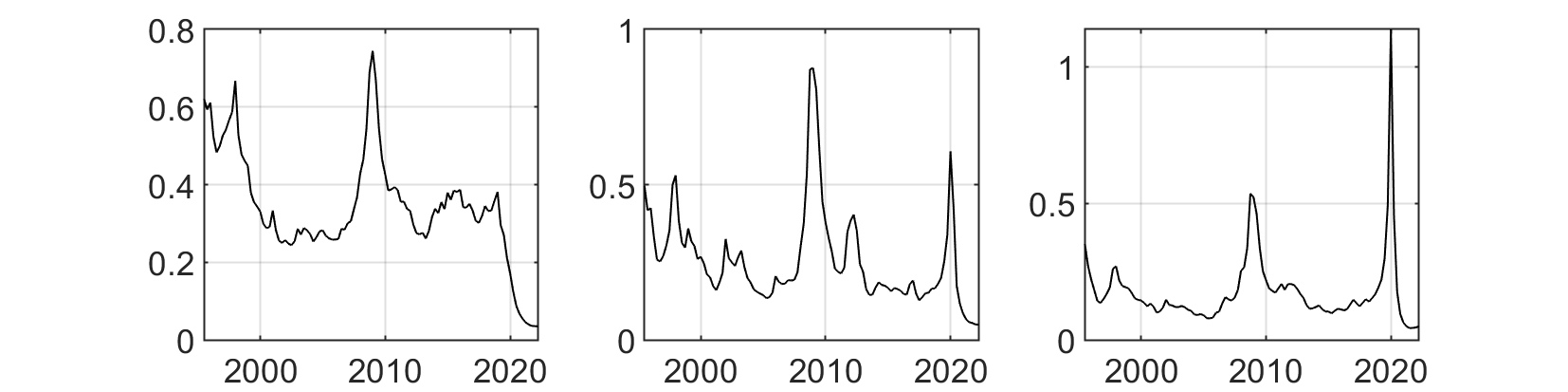

Supplement: Supplementary file 5 [file Data_Sheet_3.ZIP › KR_CHN_3 (2).tif]

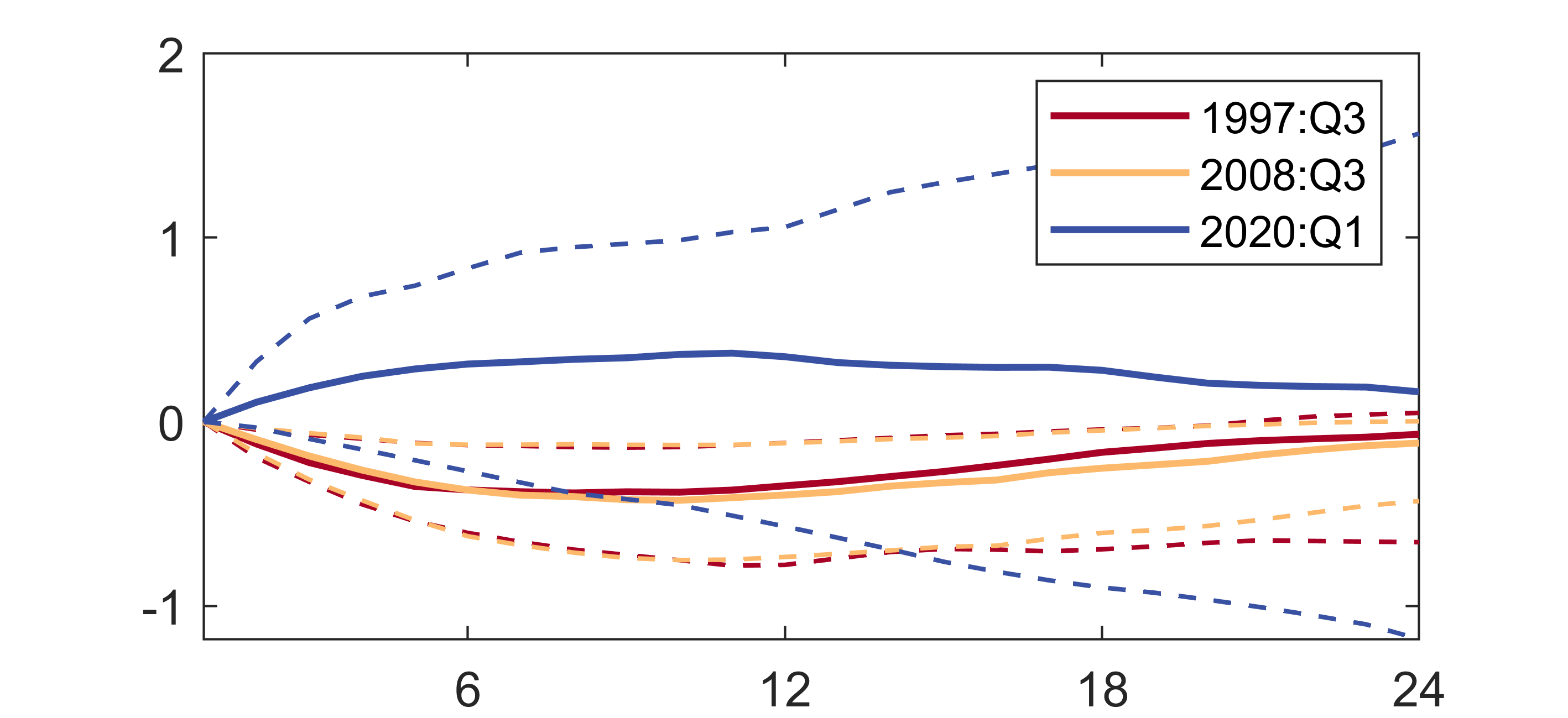

Supplement: Supplementary file 5 [file Data_Sheet_3.ZIP › KR_CHN_3 (3).tif]

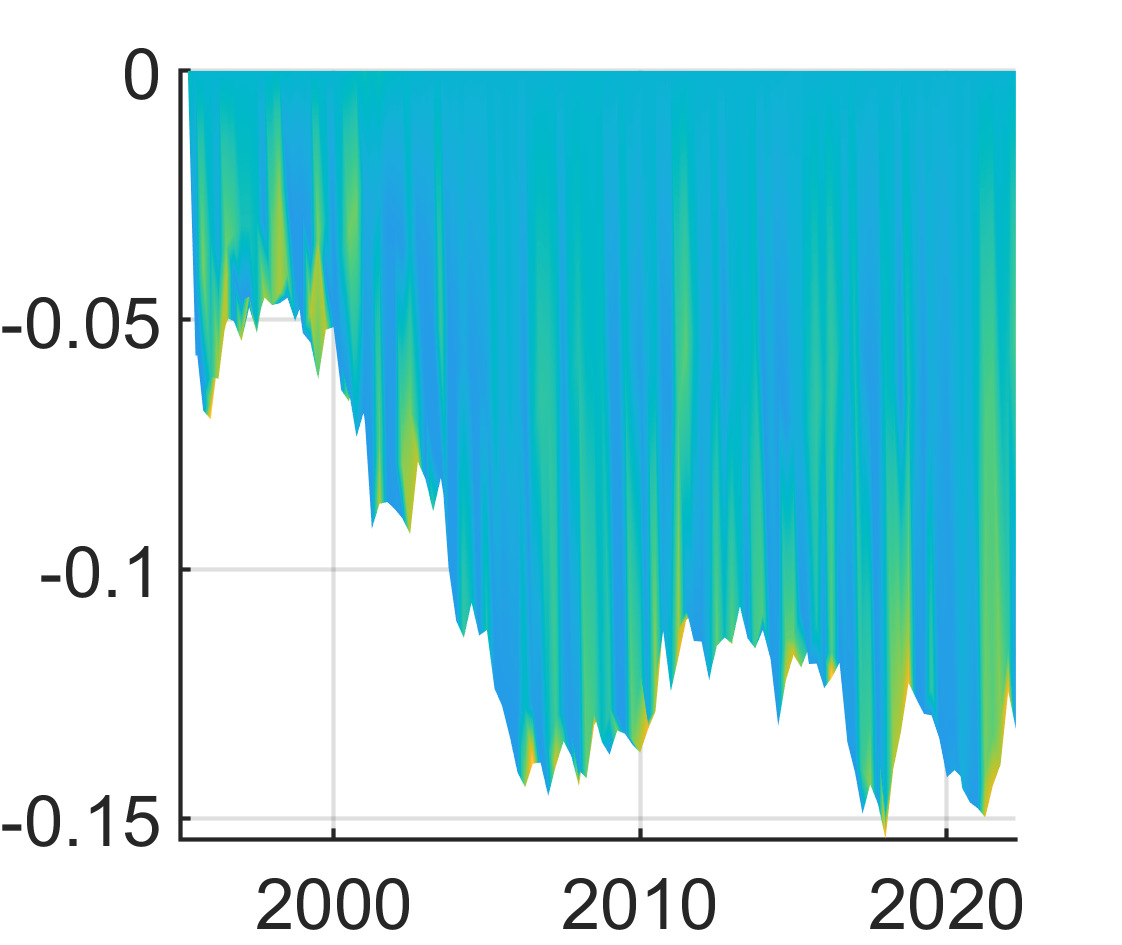

Supplement: Supplementary file 6 [file Data_Sheet_4.ZIP › BM_CHN (1).tif]

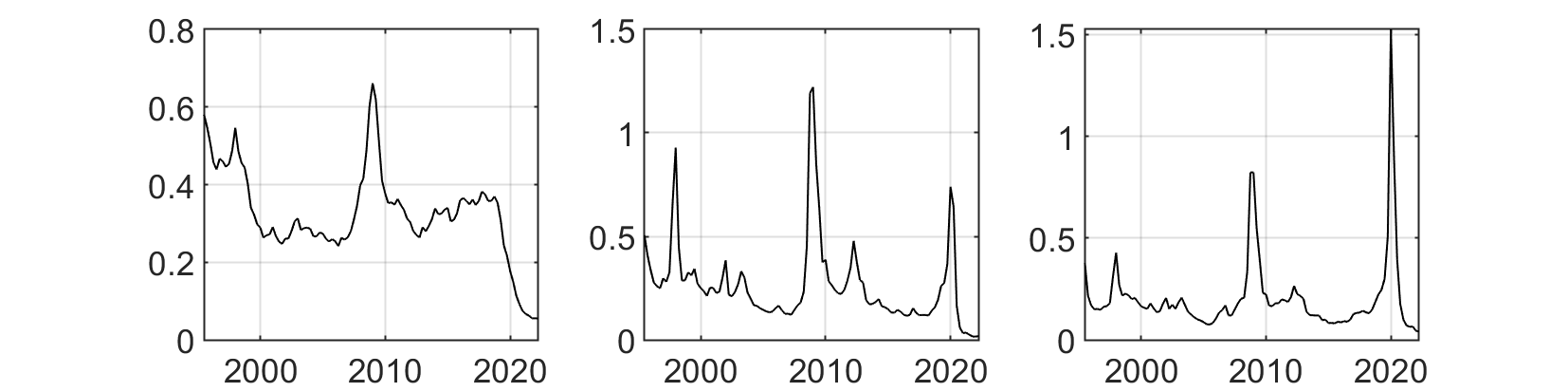

Supplement: Supplementary file 6 [file Data_Sheet_4.ZIP › BM_CHN (2).tif]

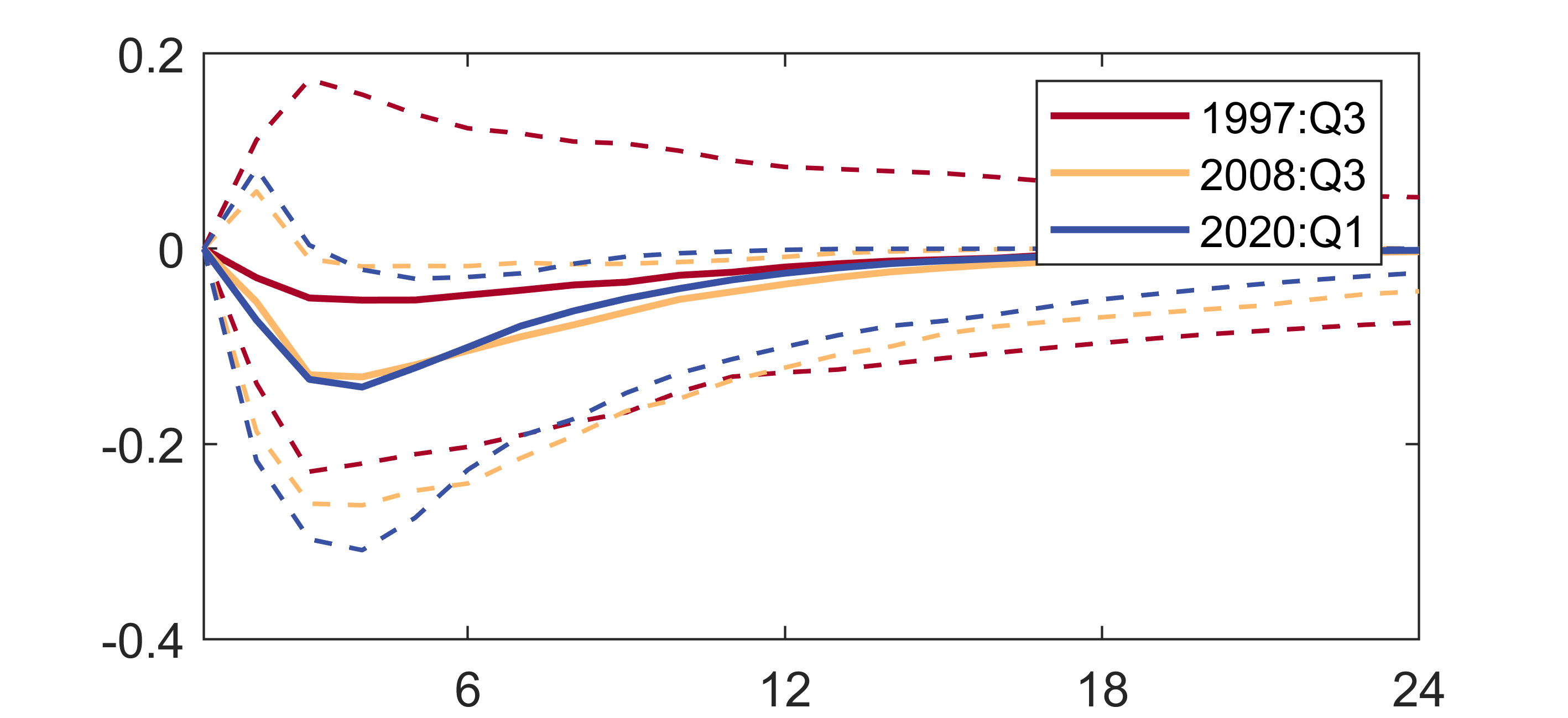

Supplement: Supplementary file 6 [file Data_Sheet_4.ZIP › BM_CHN (3).tif]

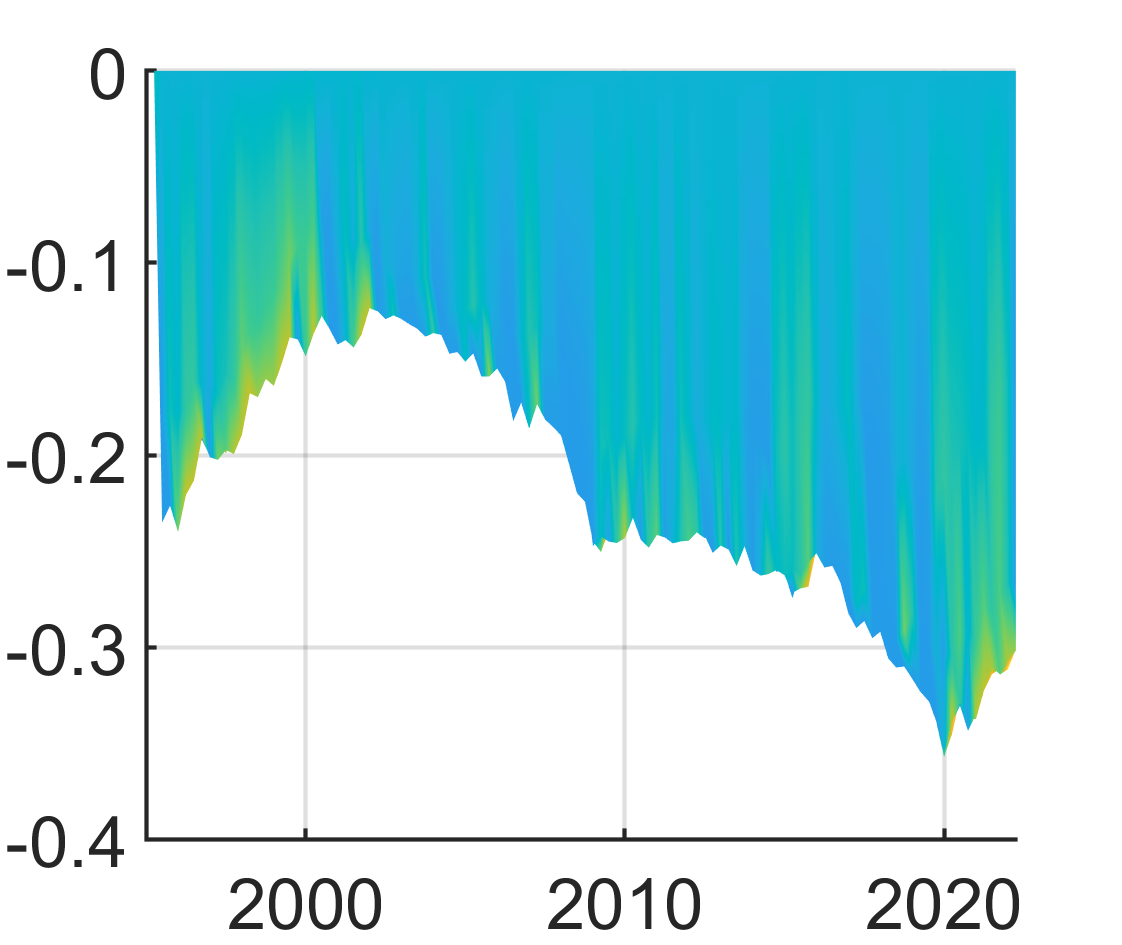

Supplement: Supplementary file 6 [file Data_Sheet_4.ZIP › BM_HK (1).tif]
