# Supplementary figures and images for: Research on the dynamic spillover of stock markets under COVID-19—Taking the stock markets of China, Japan, and South Korea as an example (part 3 of 3)
Source: Front Public Health. 2022 Nov 11;10:1008348. doi: 10.3389/fpubh.2022.1008348 (PMC9691647; doi:10.3389/fpubh.2022.1008348)

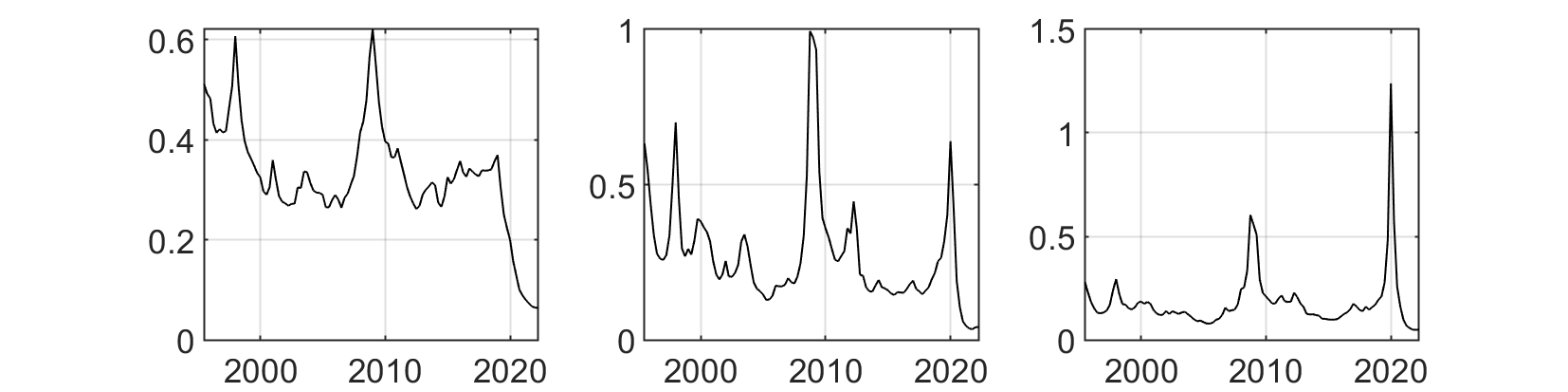

Supplement: Supplementary file 6 [file Data_Sheet_4.ZIP › BM_HK (2).tif]

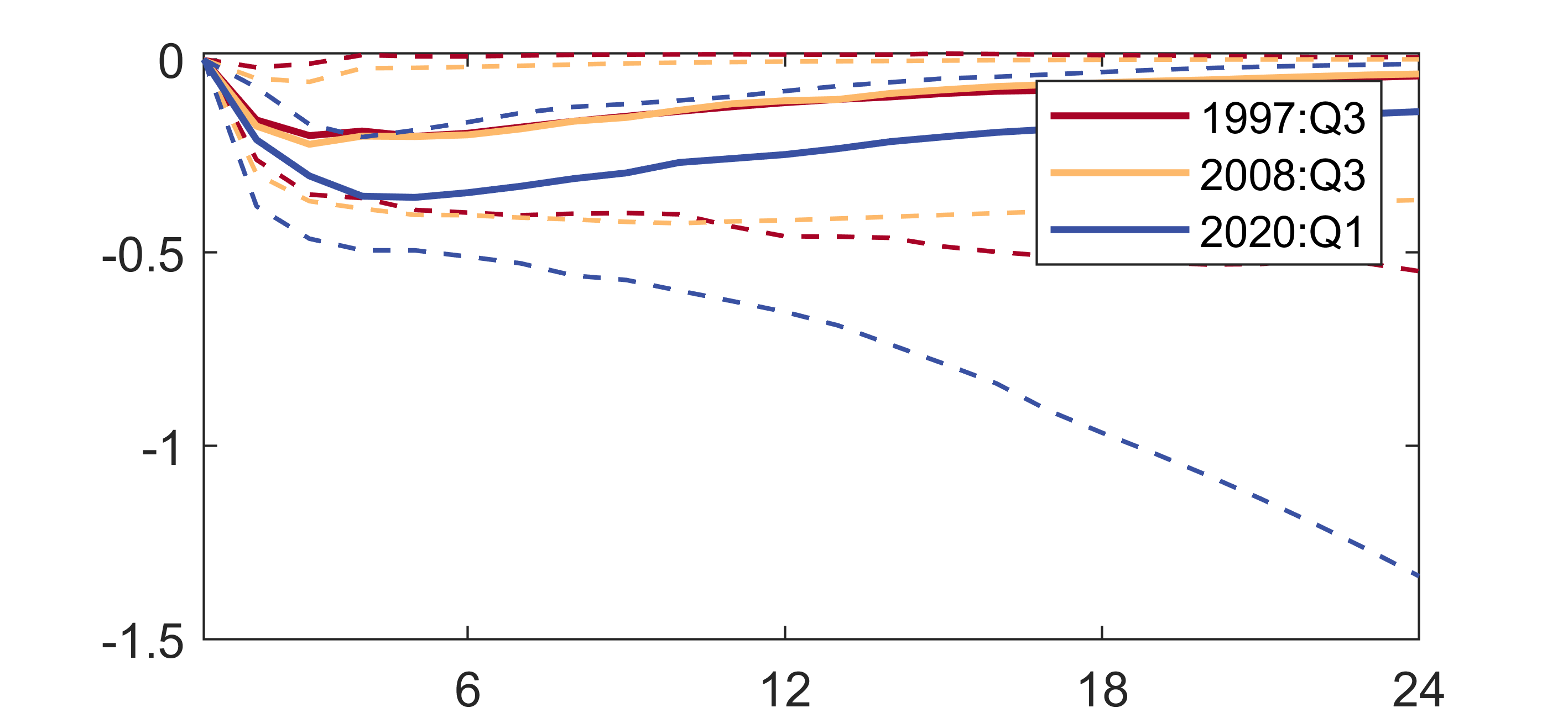

Supplement: Supplementary file 6 [file Data_Sheet_4.ZIP › BM_HK (3).tif]

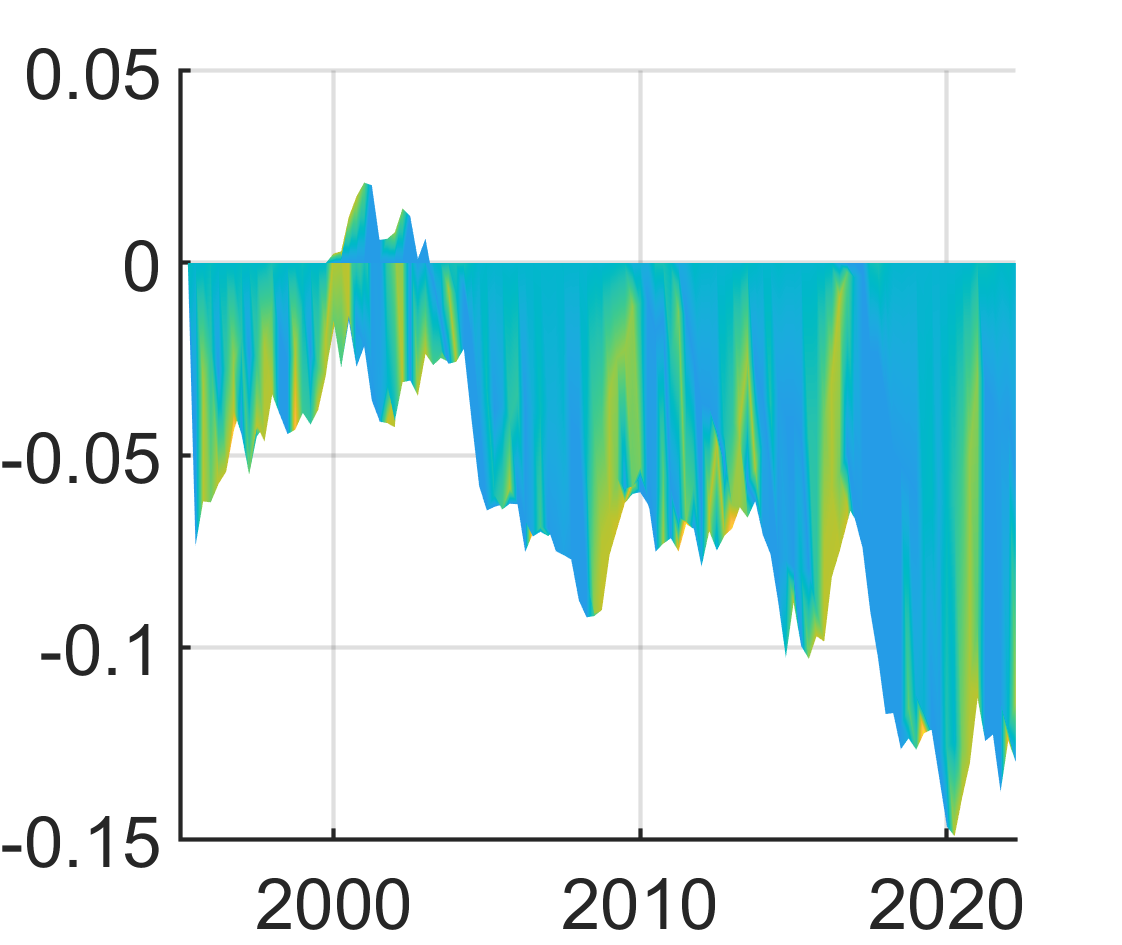

Supplement: Supplementary file 6 [file Data_Sheet_4.ZIP › BM_JPN (1).tif]

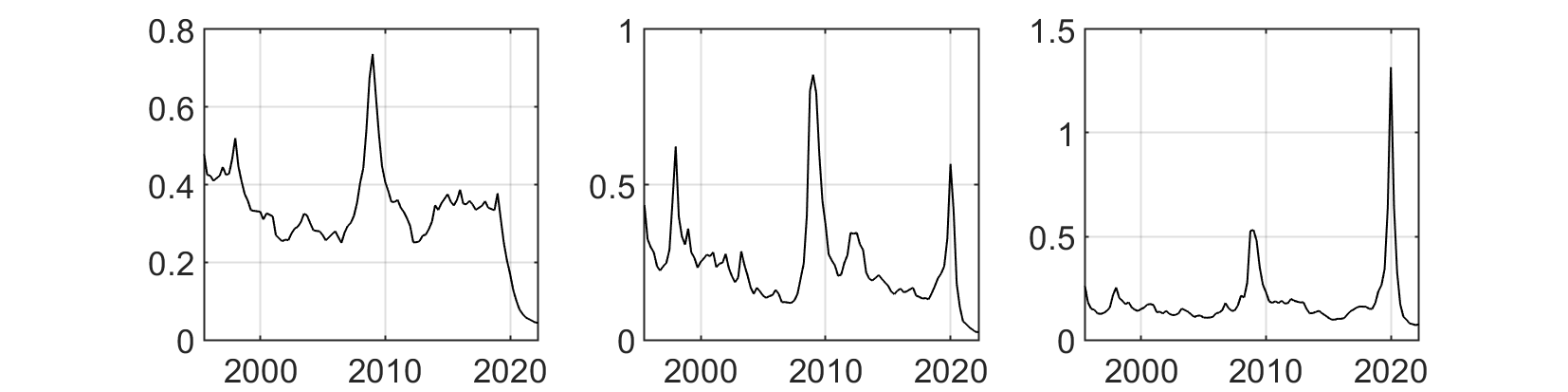

Supplement: Supplementary file 6 [file Data_Sheet_4.ZIP › BM_JPN (2).tif]

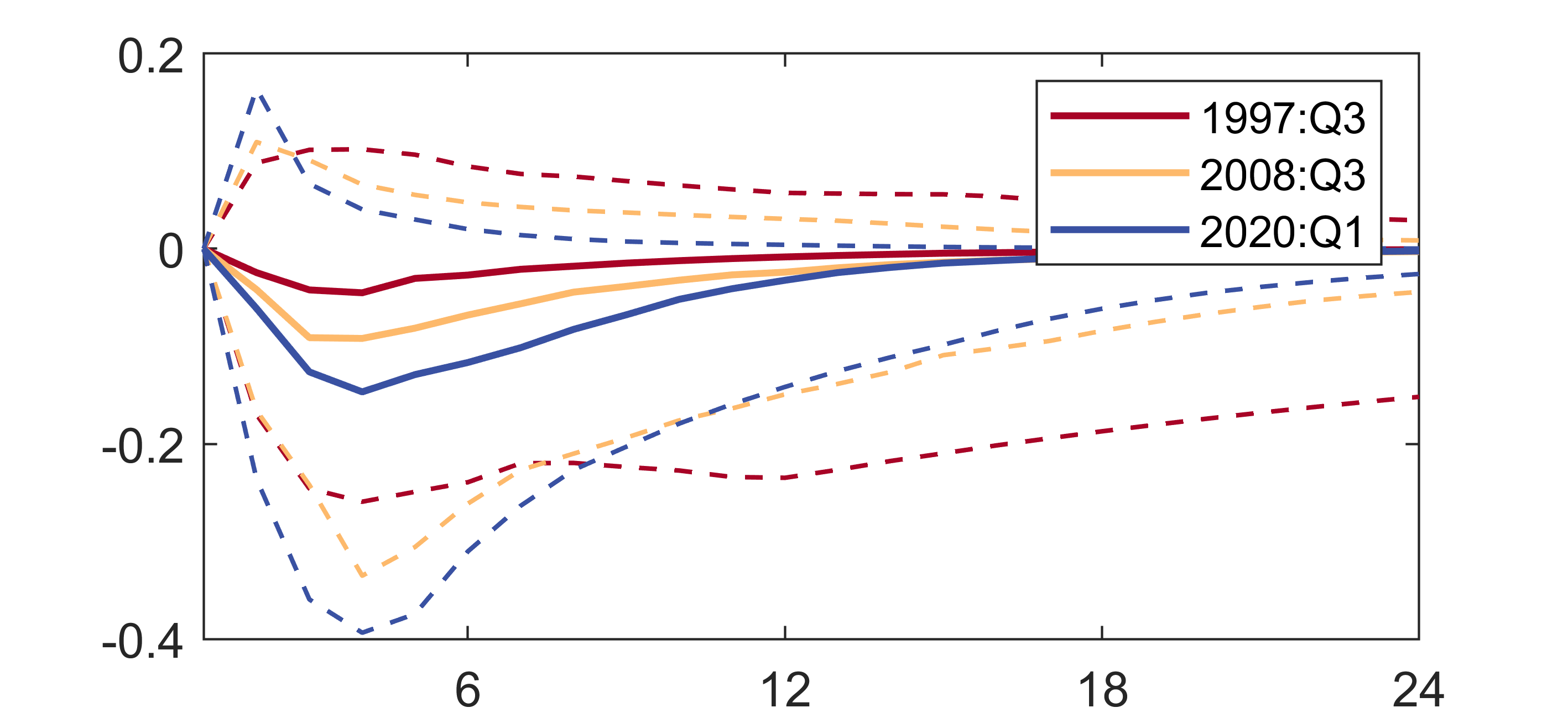

Supplement: Supplementary file 6 [file Data_Sheet_4.ZIP › BM_JPN (3).tif]

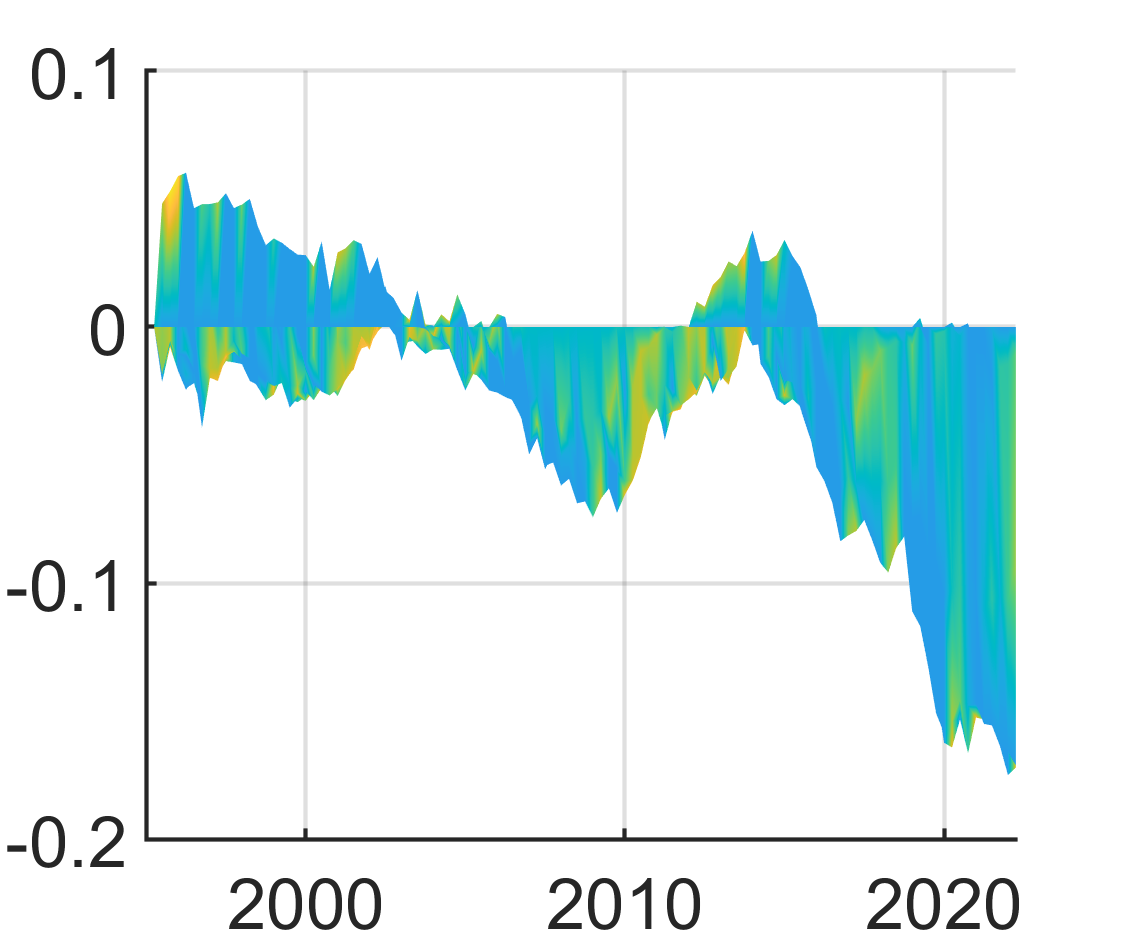

Supplement: Supplementary file 6 [file Data_Sheet_4.ZIP › BM_KR (1).tif]

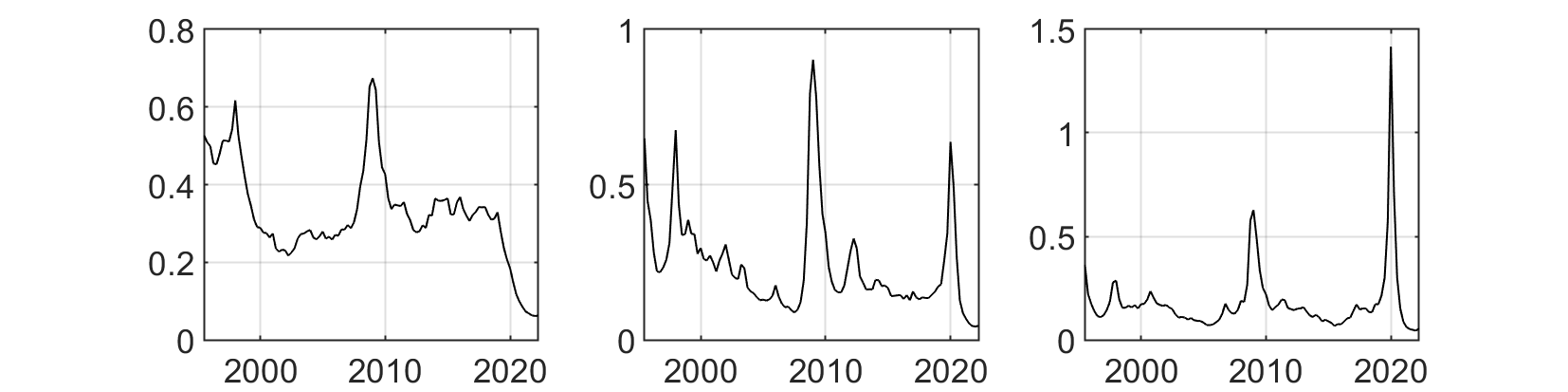

Supplement: Supplementary file 6 [file Data_Sheet_4.ZIP › BM_KR (2).tif]

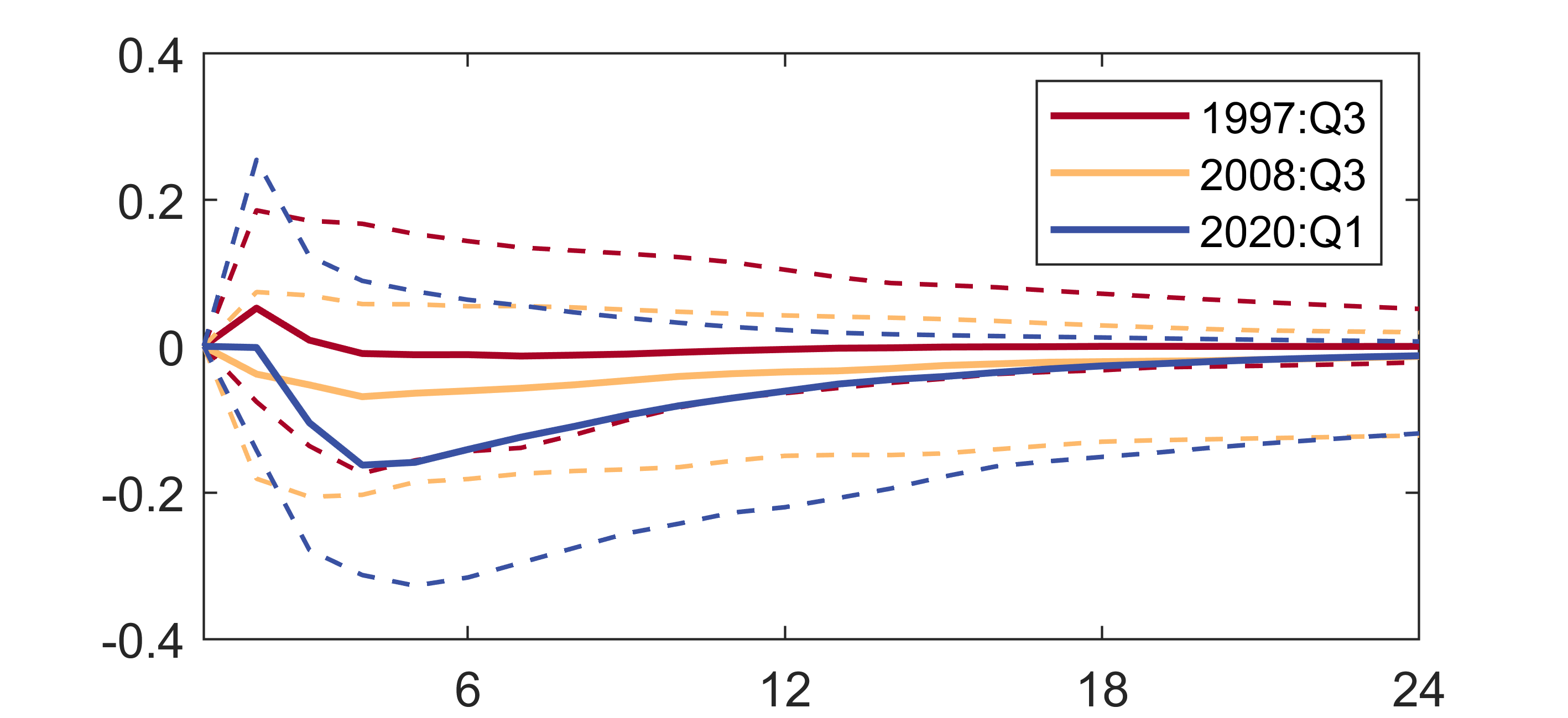

Supplement: Supplementary file 6 [file Data_Sheet_4.ZIP › BM_KR (3).tif]

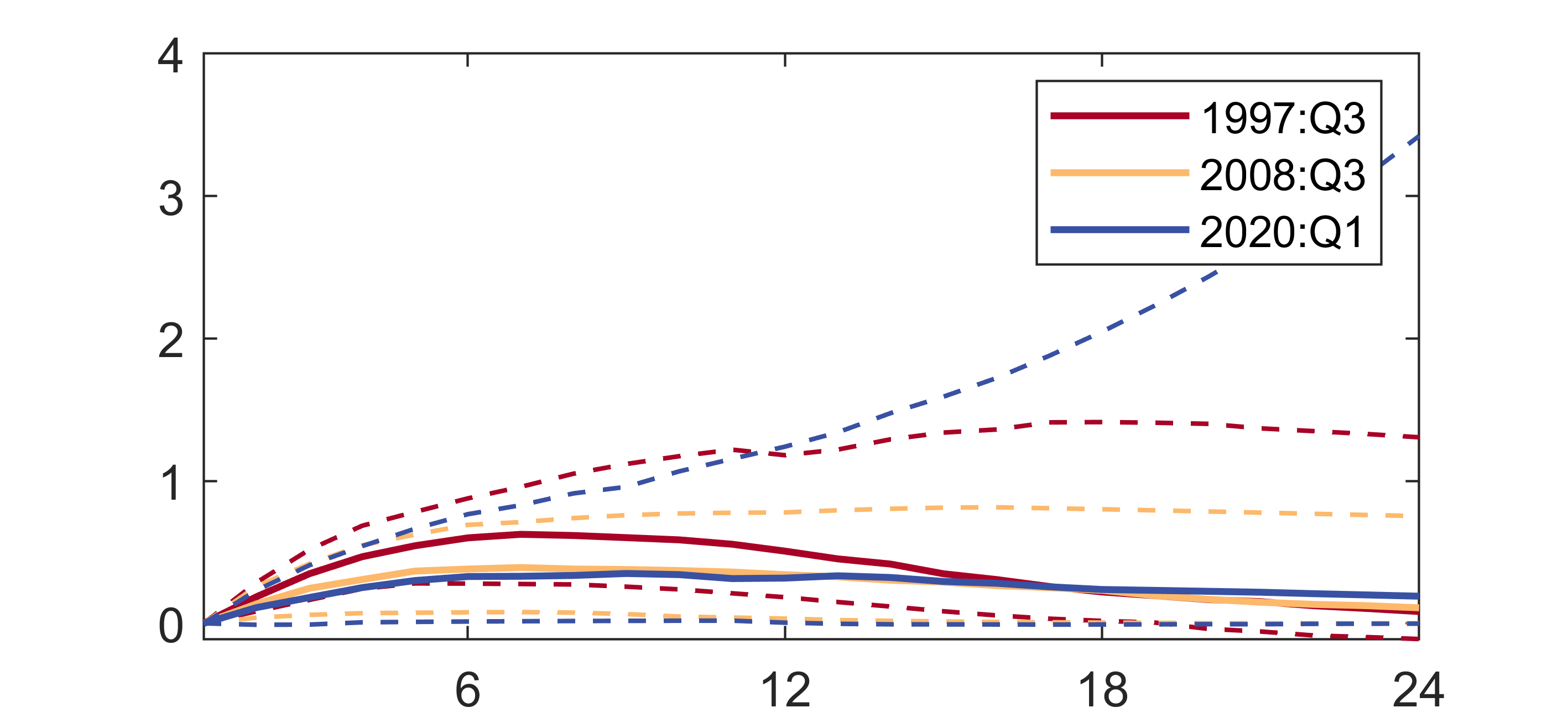

Supplement: Supplementary file 6 [file Data_Sheet_4.ZIP › China_to_HK(3).tif]

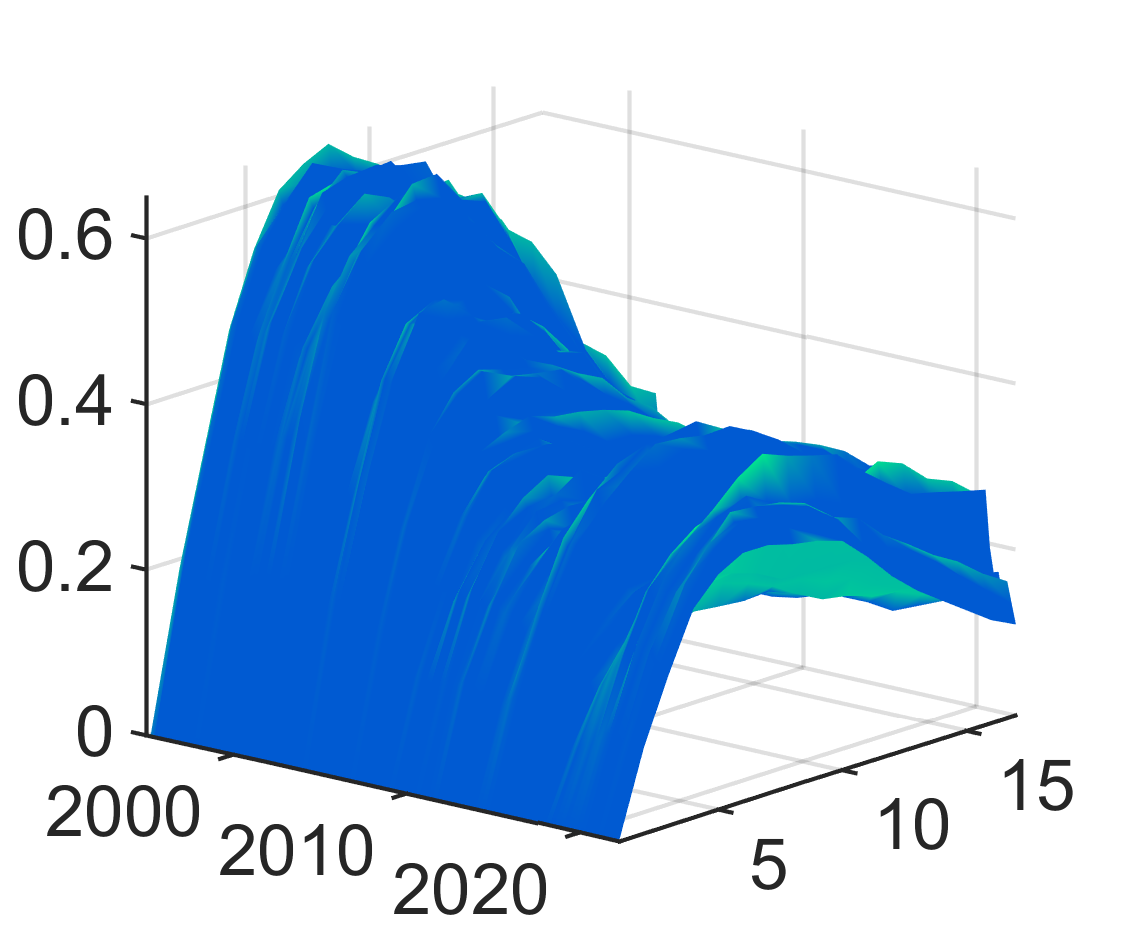

Supplement: Supplementary file 6 [file Data_Sheet_4.ZIP › China_to_HK(4).tif]

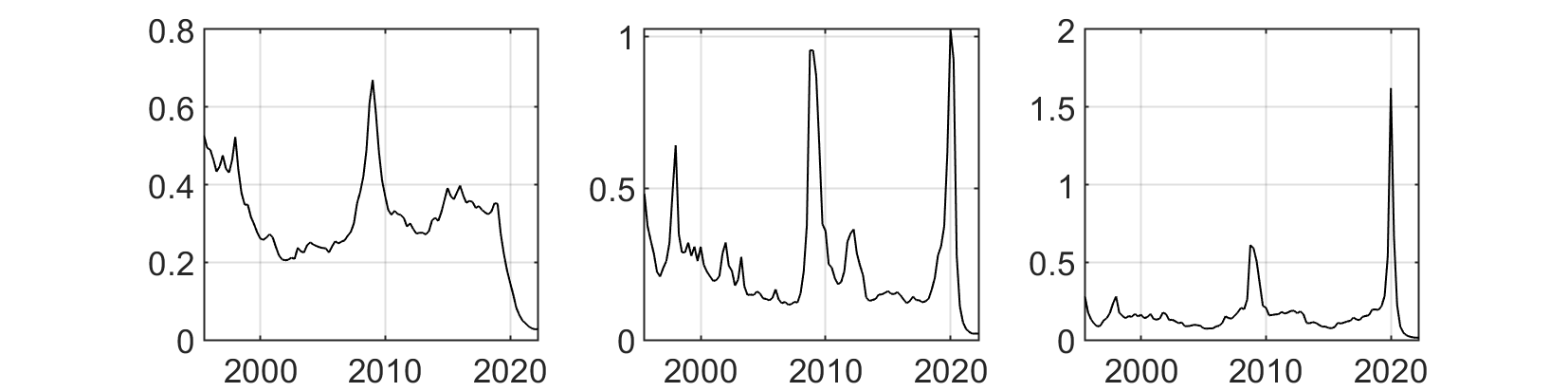

Supplement: Supplementary file 6 [file Data_Sheet_4.ZIP › China_to_HK(5).tif]

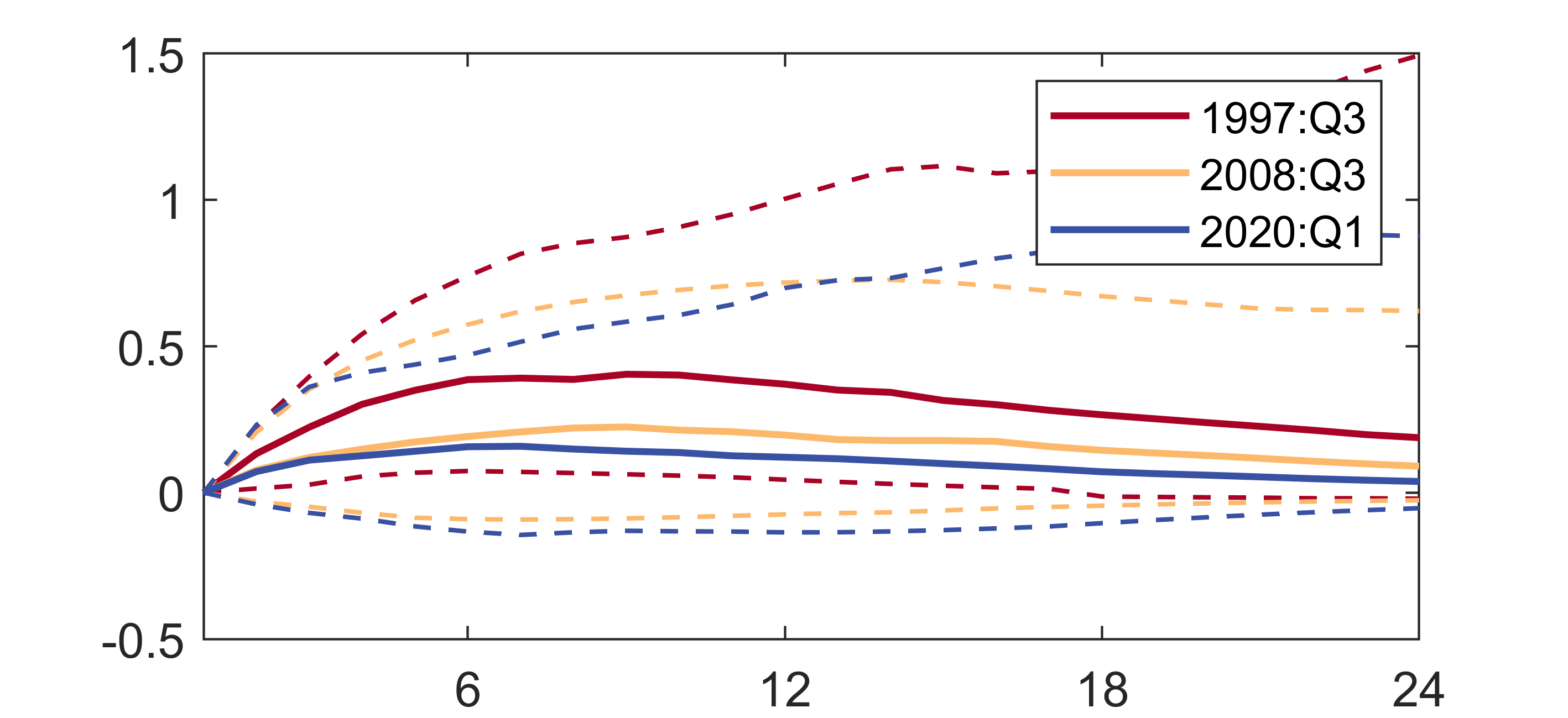

Supplement: Supplementary file 6 [file Data_Sheet_4.ZIP › CHN_to_JPN (1).tif]

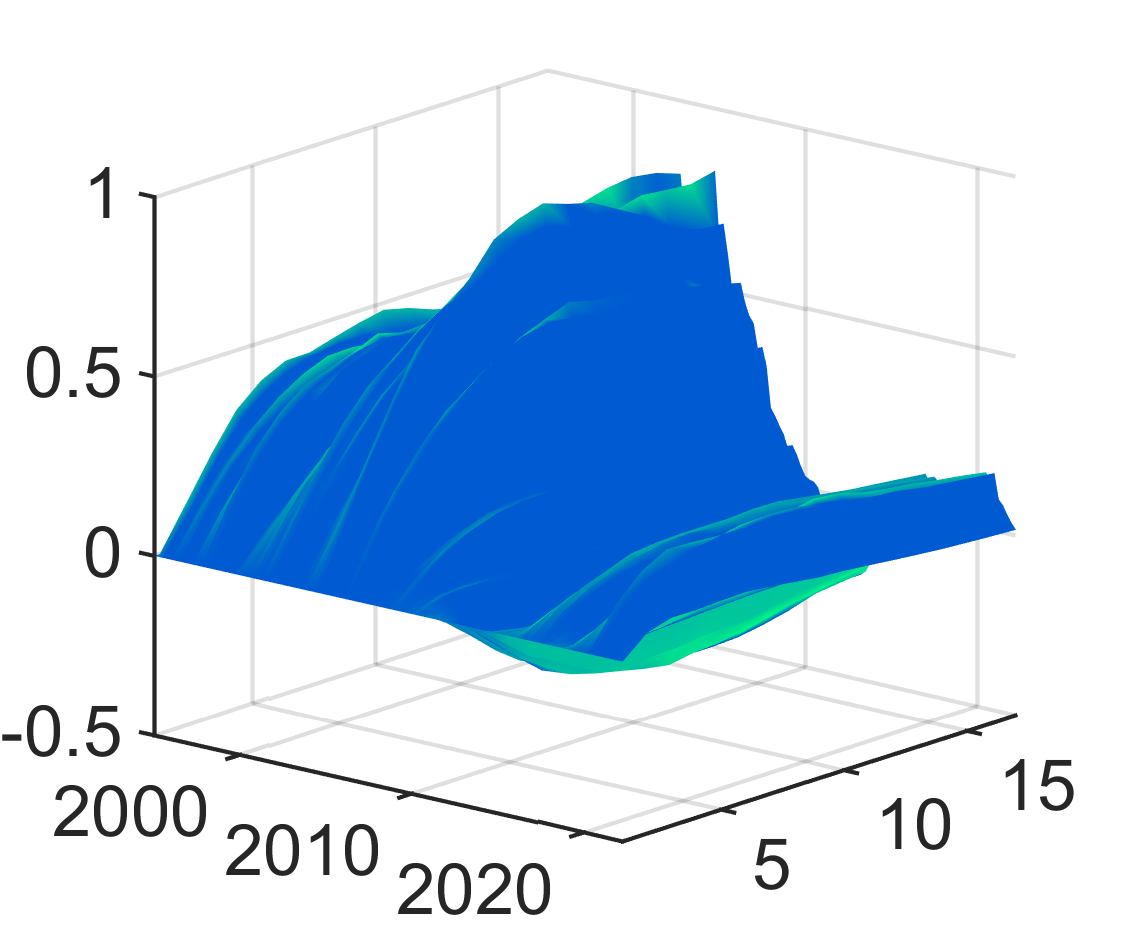

Supplement: Supplementary file 6 [file Data_Sheet_4.ZIP › CHN_to_JPN (2).tif]

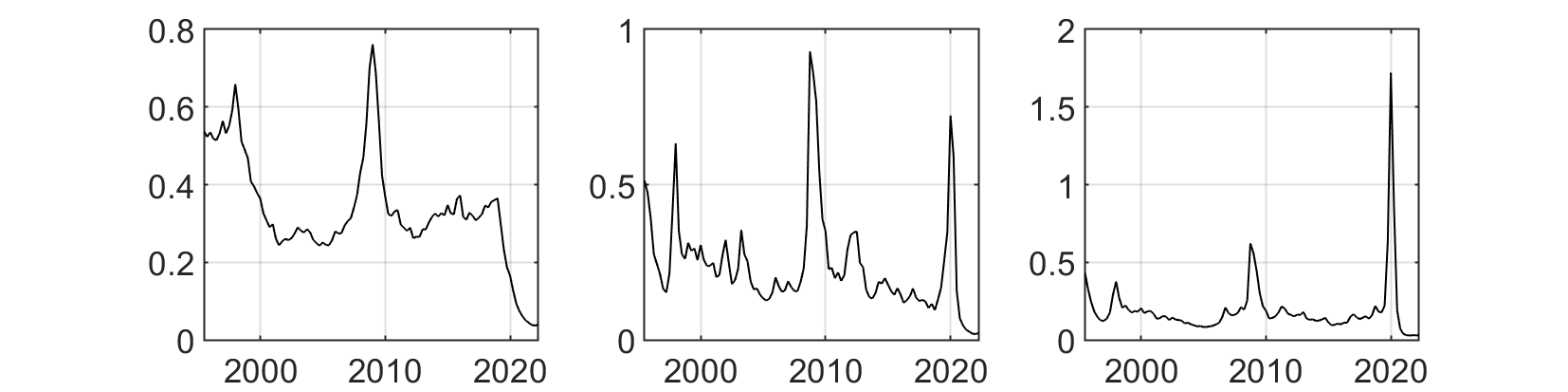

Supplement: Supplementary file 6 [file Data_Sheet_4.ZIP › CHN_to_JPN (3).tif]

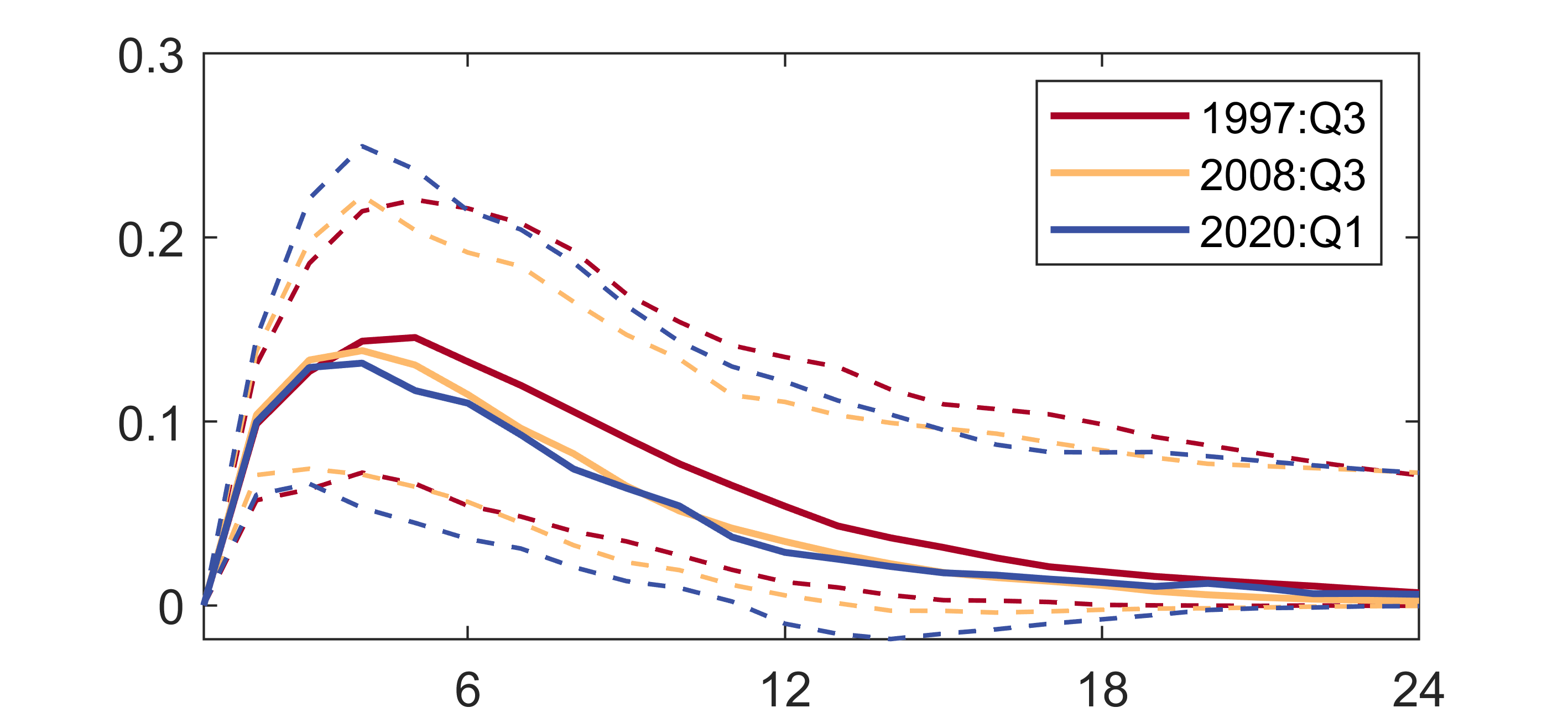

Supplement: Supplementary file 6 [file Data_Sheet_4.ZIP › CHN_to_KR (1).tif]

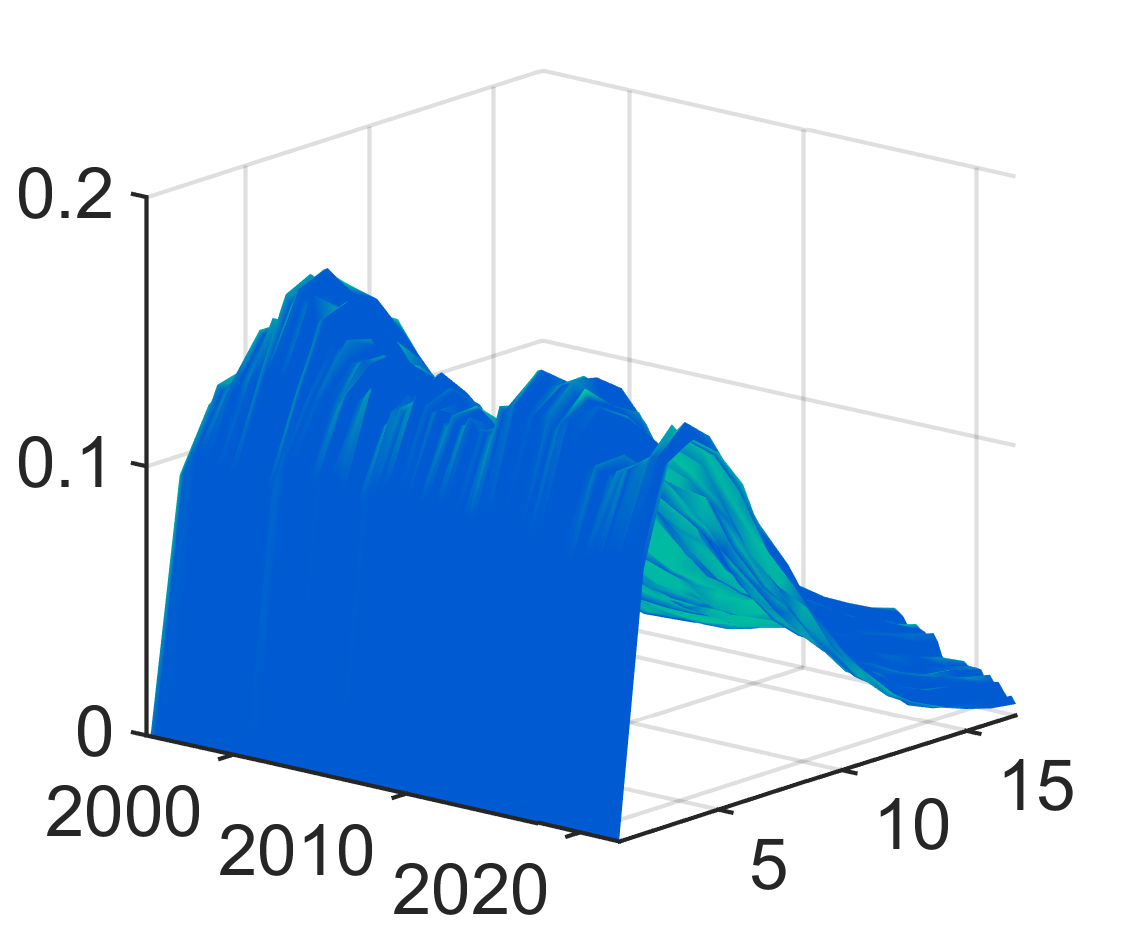

Supplement: Supplementary file 6 [file Data_Sheet_4.ZIP › CHN_to_KR (2).tif]

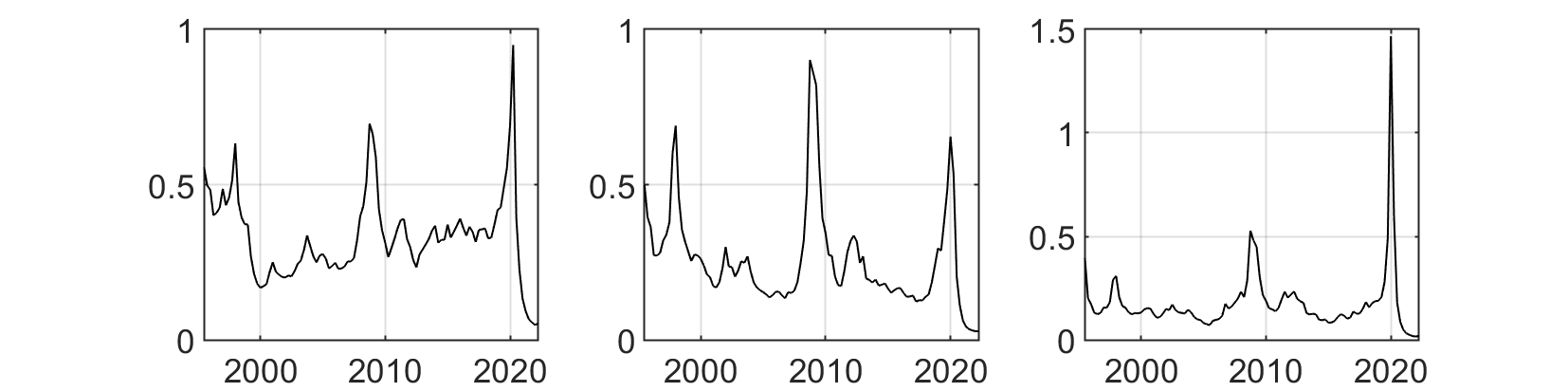

Supplement: Supplementary file 6 [file Data_Sheet_4.ZIP › CHN_to_KR (3).tif]

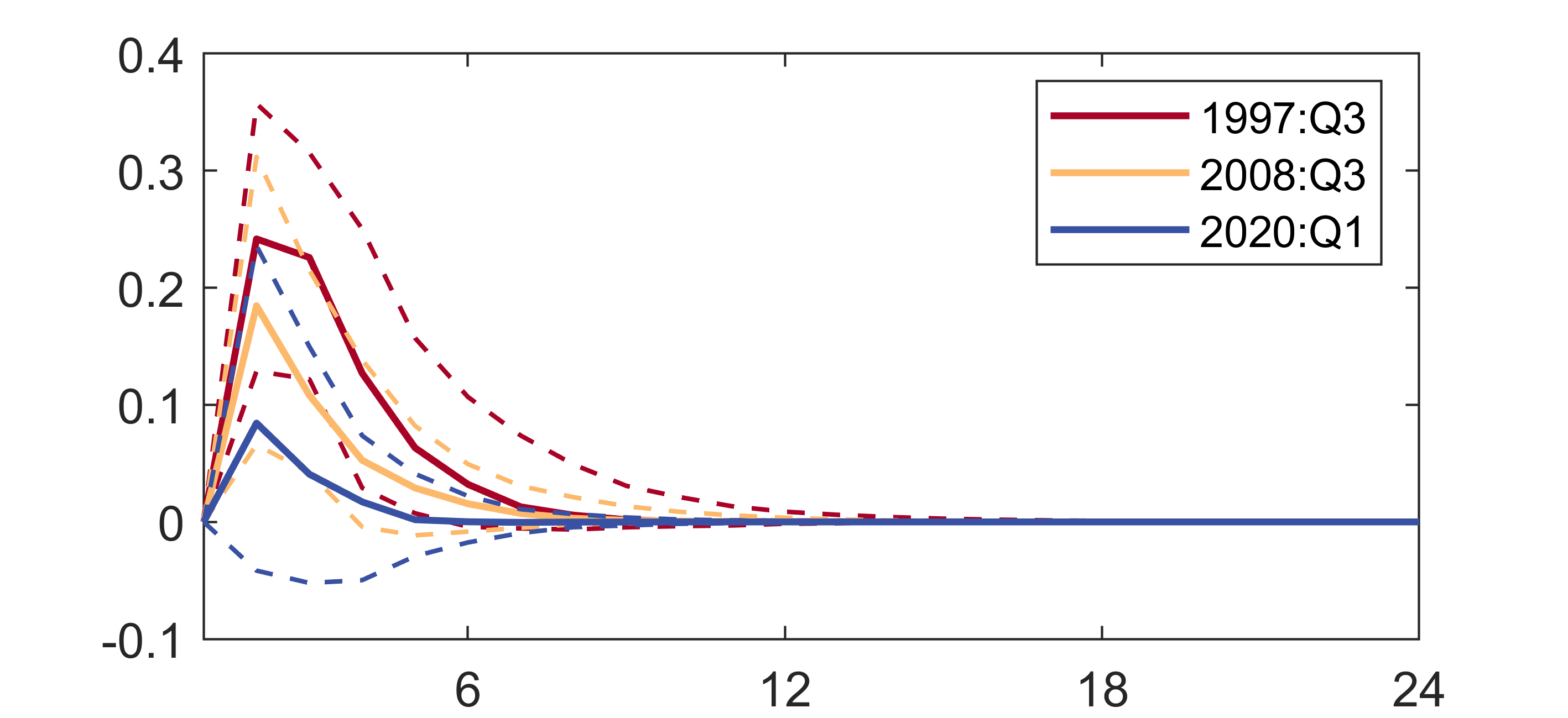

Supplement: Supplementary file 6 [file Data_Sheet_4.ZIP › CM_CHN (1).tif]

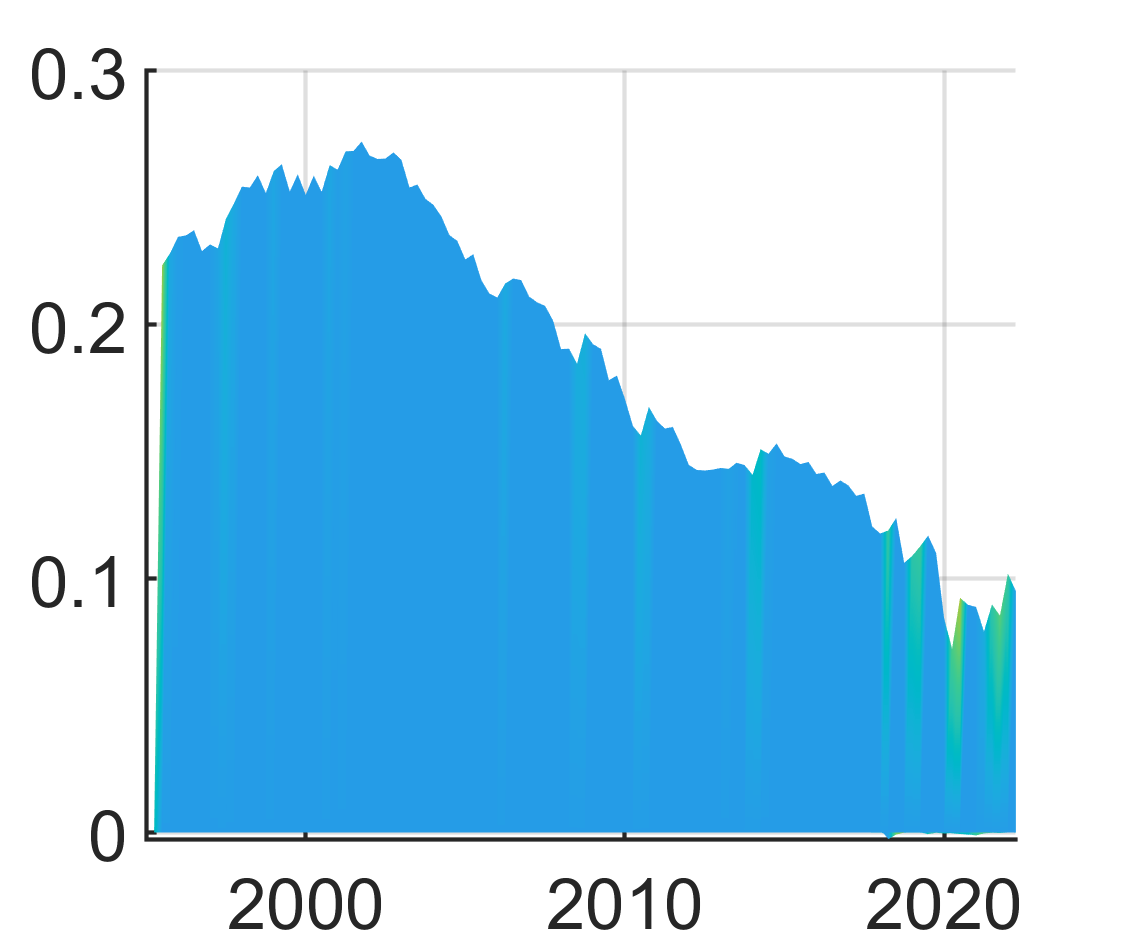

Supplement: Supplementary file 6 [file Data_Sheet_4.ZIP › CM_CHN (2).tif]

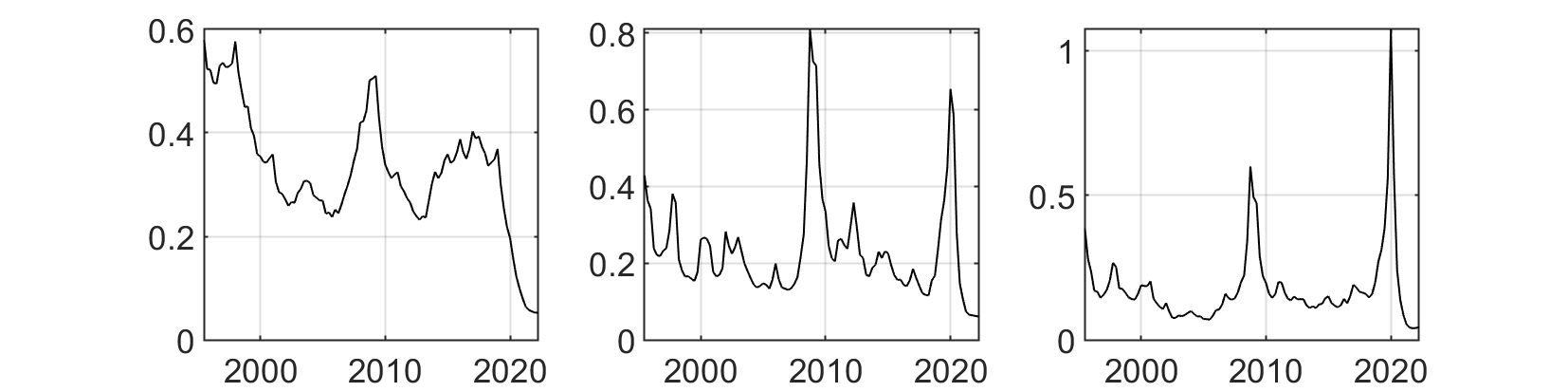

Supplement: Supplementary file 6 [file Data_Sheet_4.ZIP › CM_CHN (3).tif]

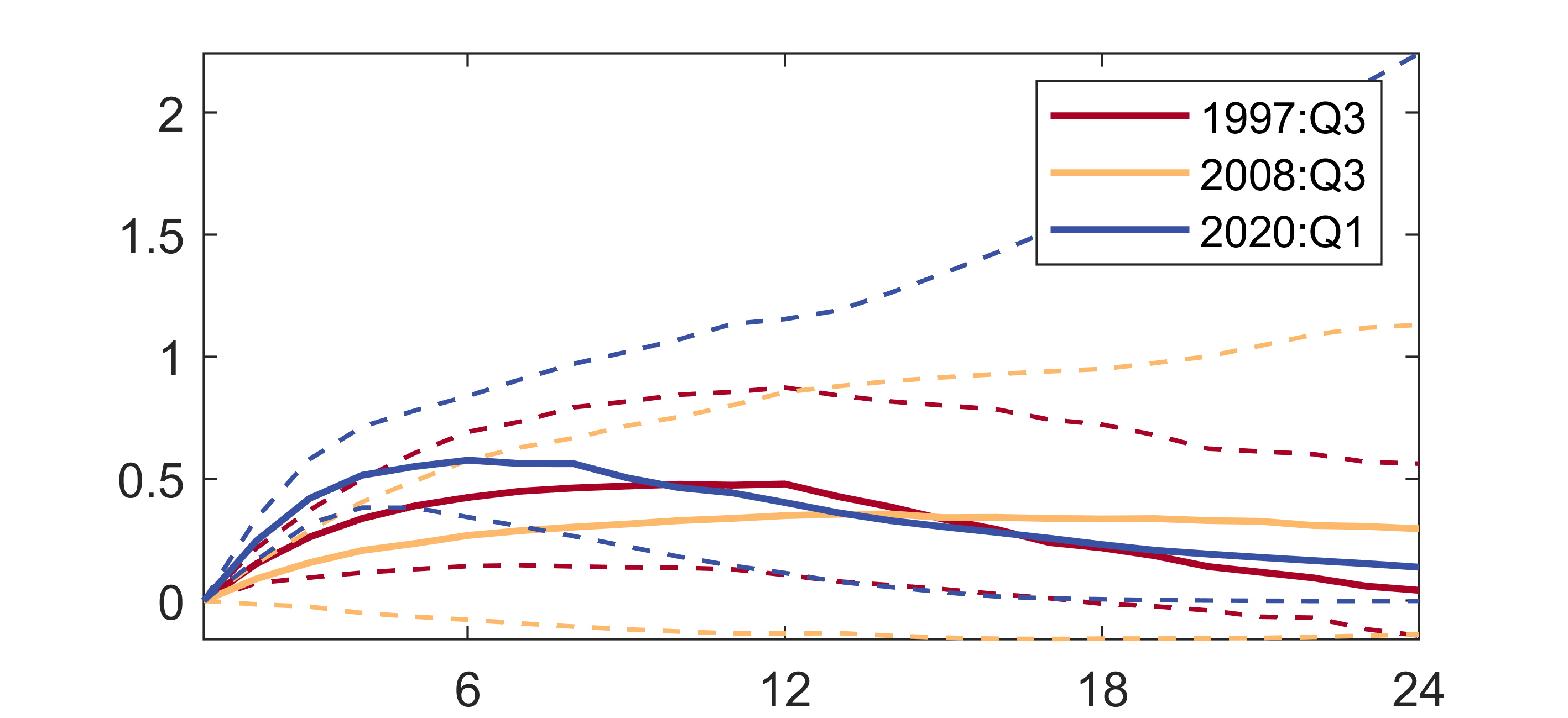

Supplement: Supplementary file 6 [file Data_Sheet_4.ZIP › CM_HK (1).tif]

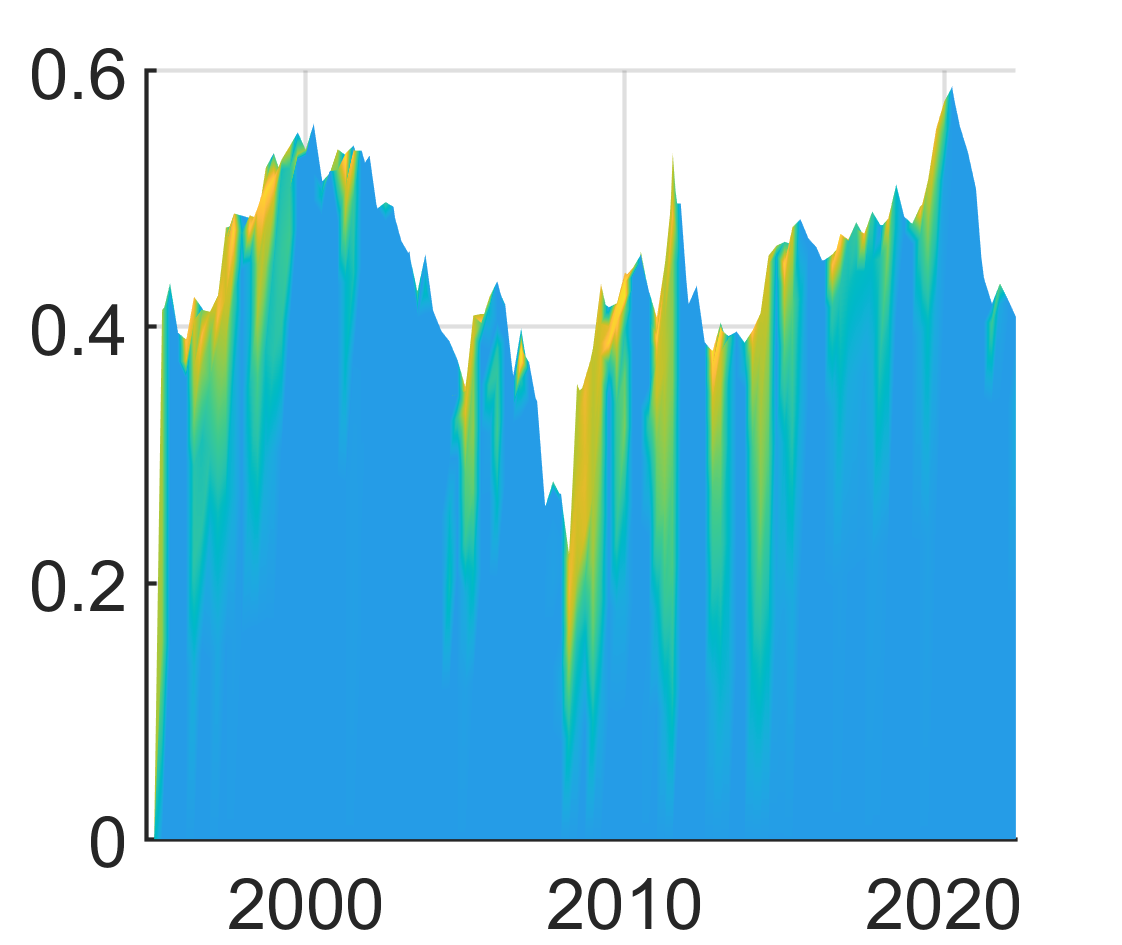

Supplement: Supplementary file 6 [file Data_Sheet_4.ZIP › CM_HK (2).tif]

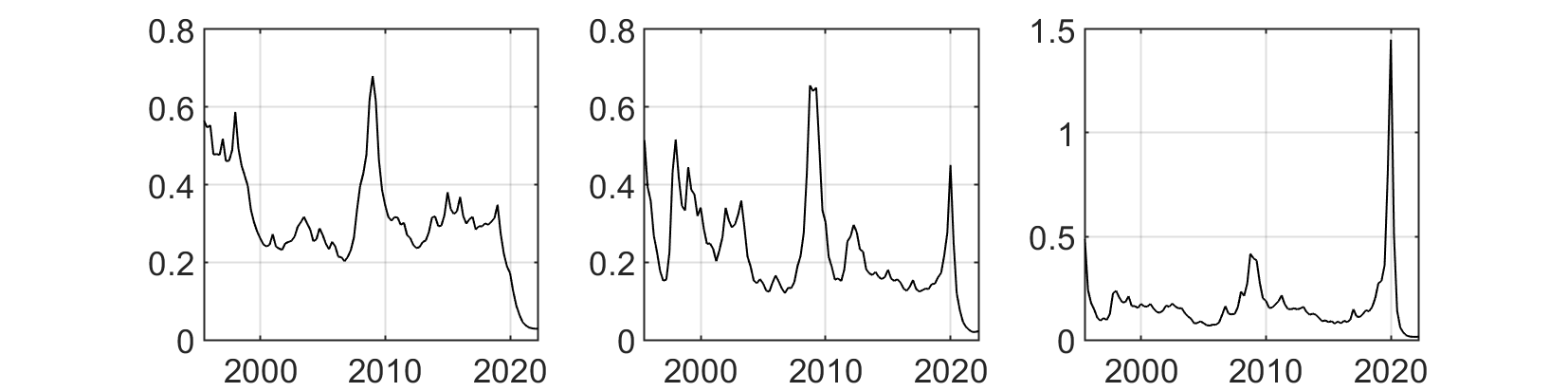

Supplement: Supplementary file 6 [file Data_Sheet_4.ZIP › CM_HK (3).tif]

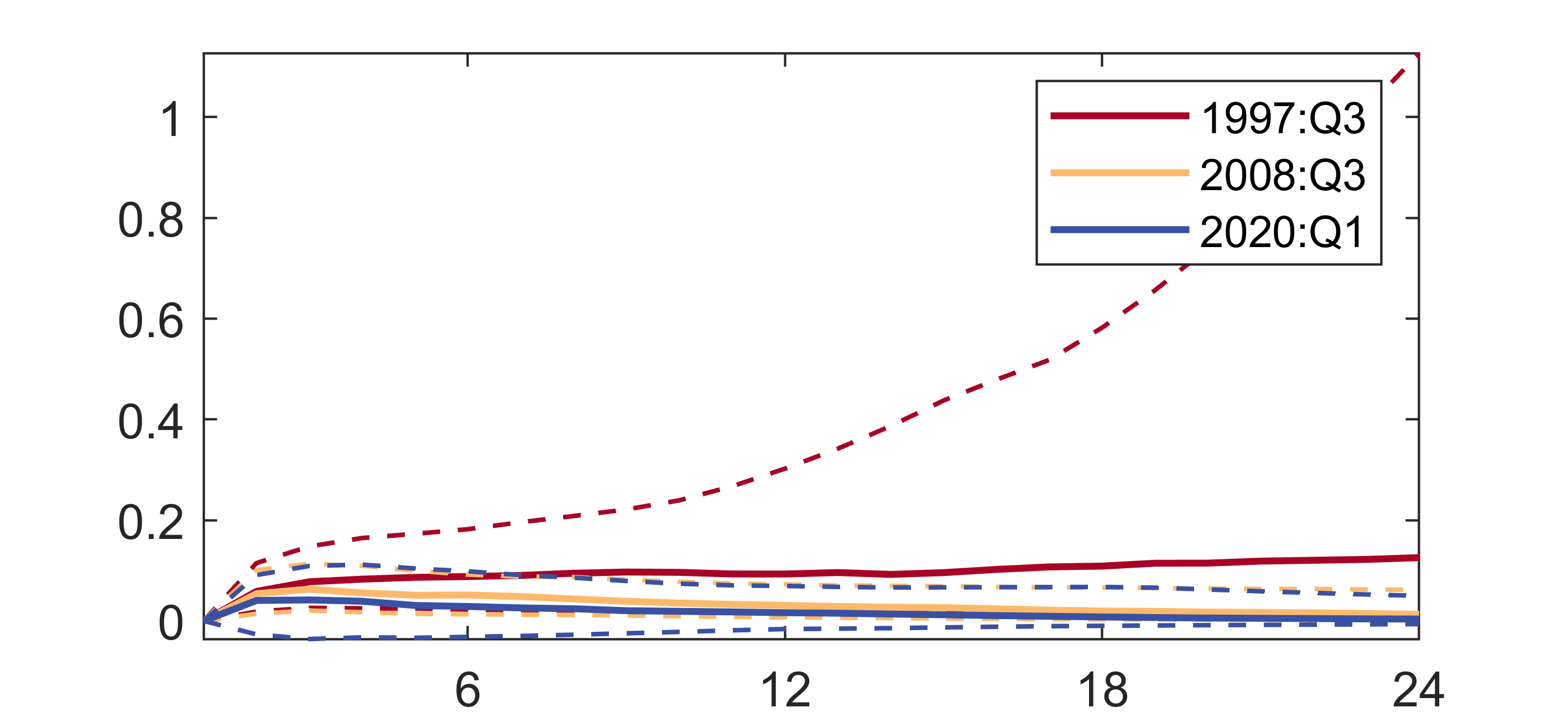

Supplement: Supplementary file 6 [file Data_Sheet_4.ZIP › CM_JPN (1).tif]

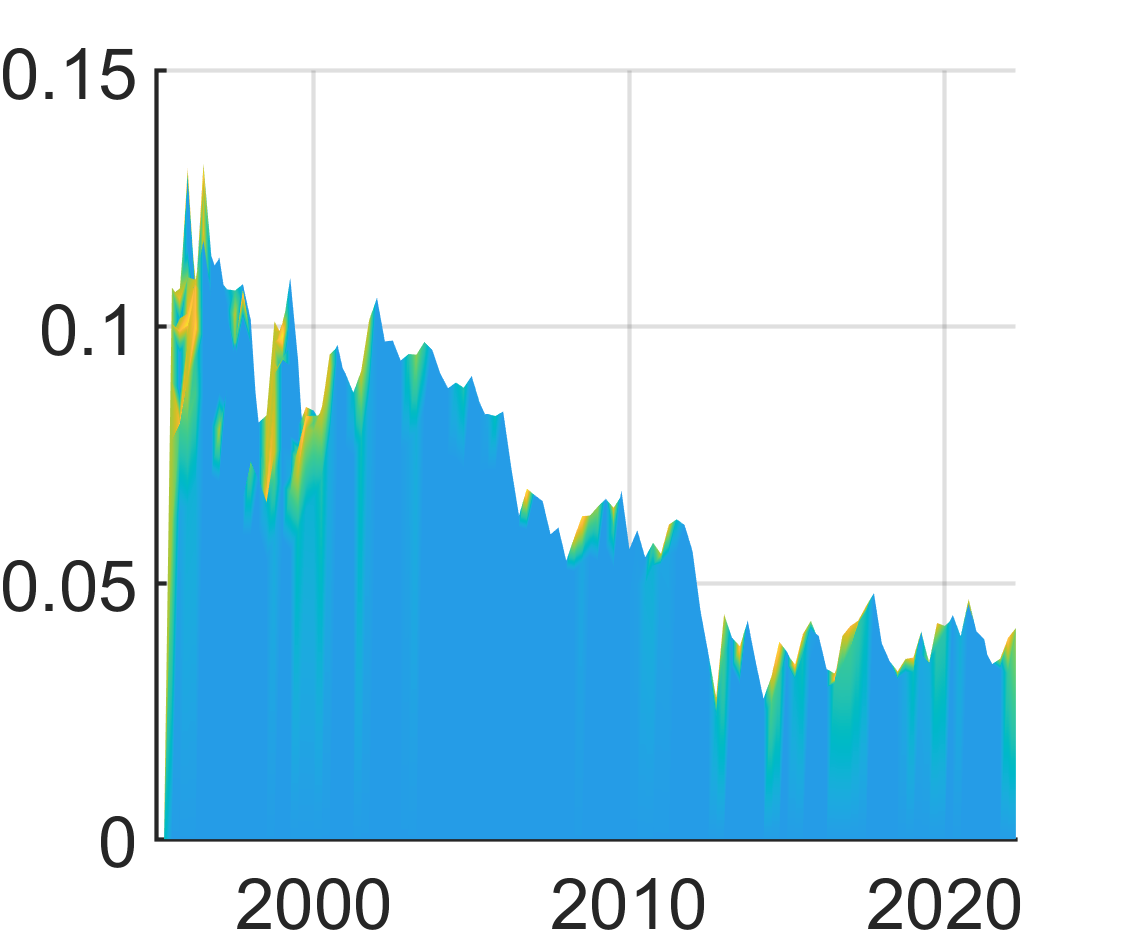

Supplement: Supplementary file 6 [file Data_Sheet_4.ZIP › CM_JPN (2).tif]

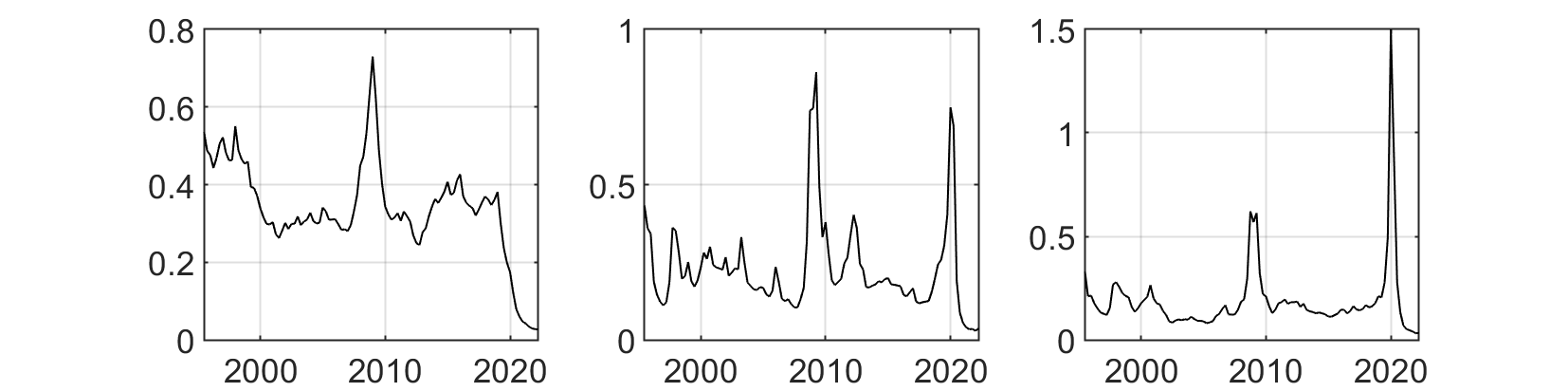

Supplement: Supplementary file 6 [file Data_Sheet_4.ZIP › CM_JPN (3).tif]

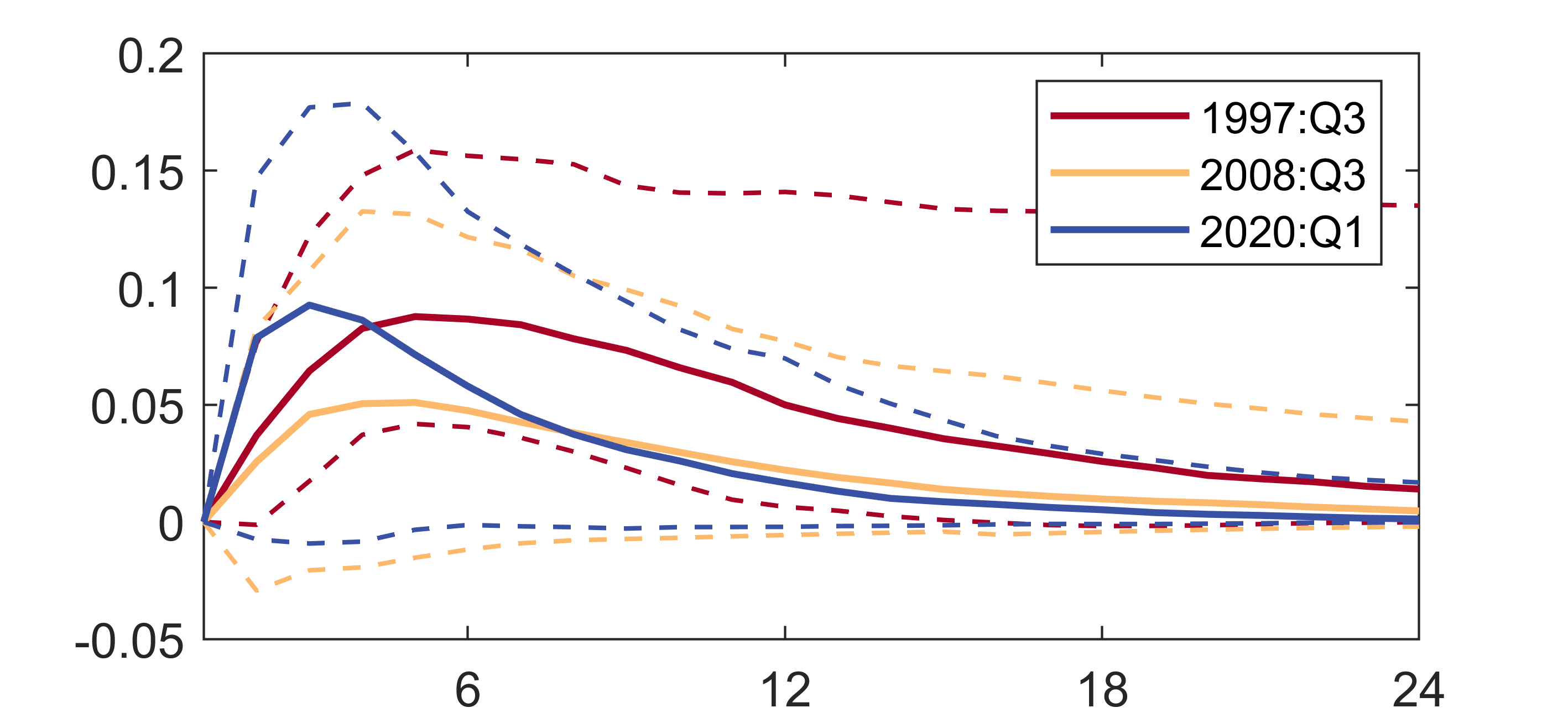

Supplement: Supplementary file 6 [file Data_Sheet_4.ZIP › CM_KR (1).tif]

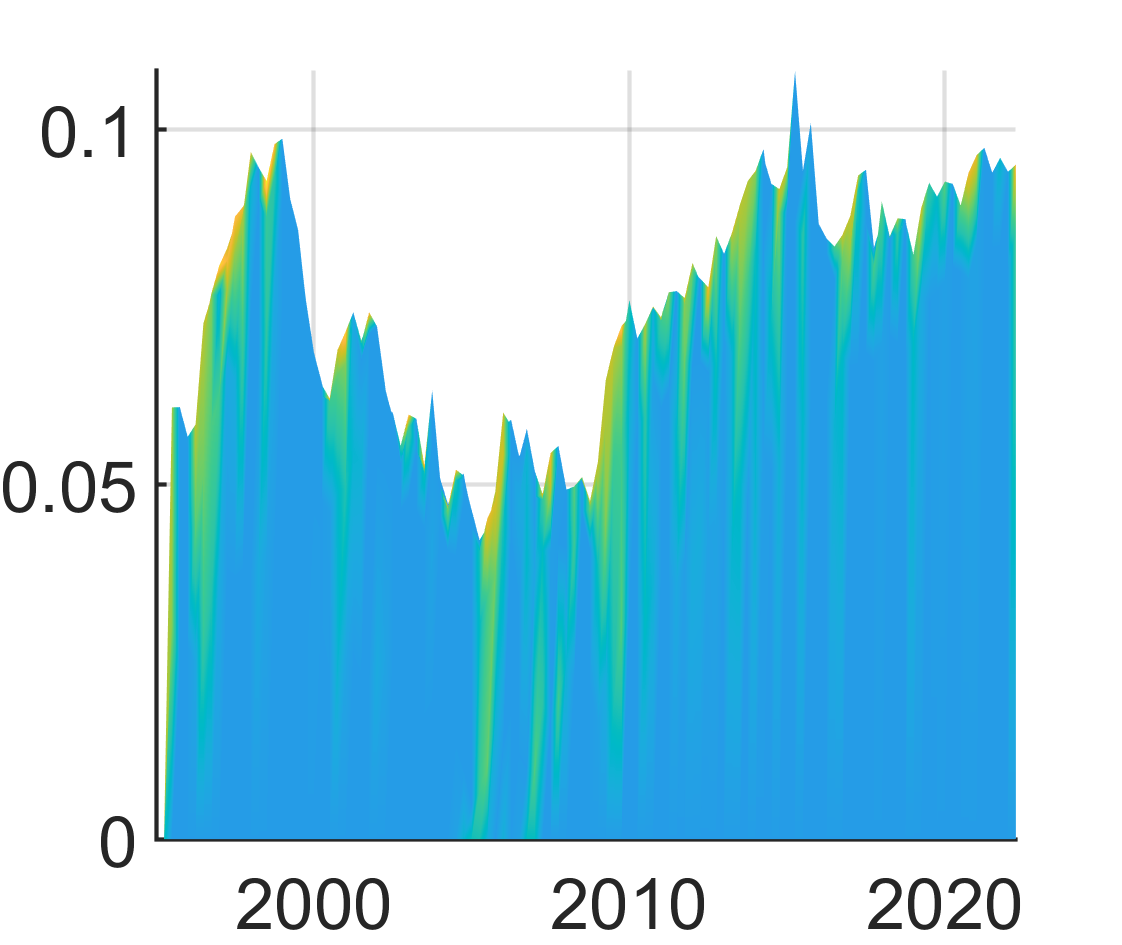

Supplement: Supplementary file 6 [file Data_Sheet_4.ZIP › CM_KR (2).tif]

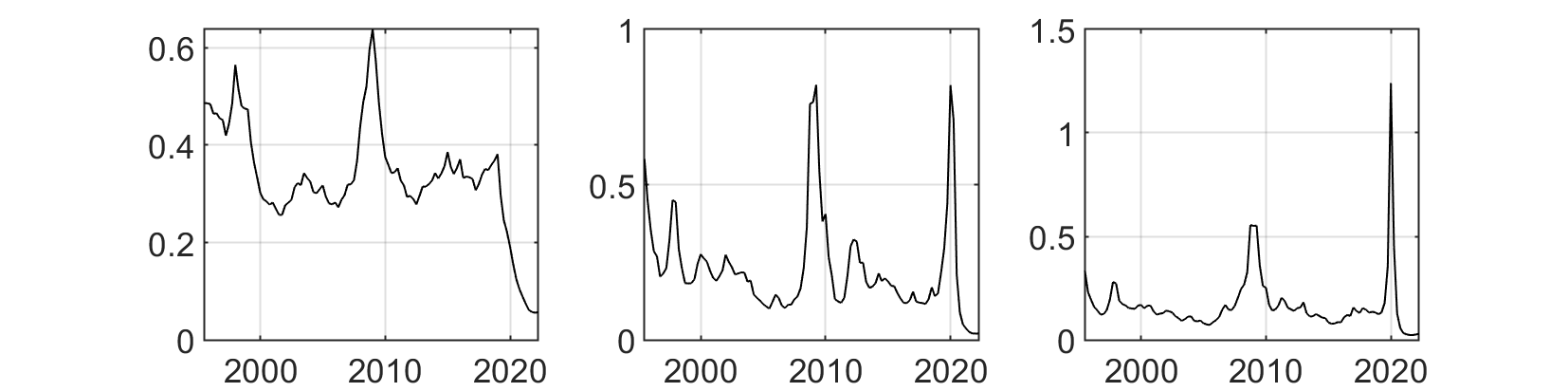

Supplement: Supplementary file 6 [file Data_Sheet_4.ZIP › CM_KR (3).tif]

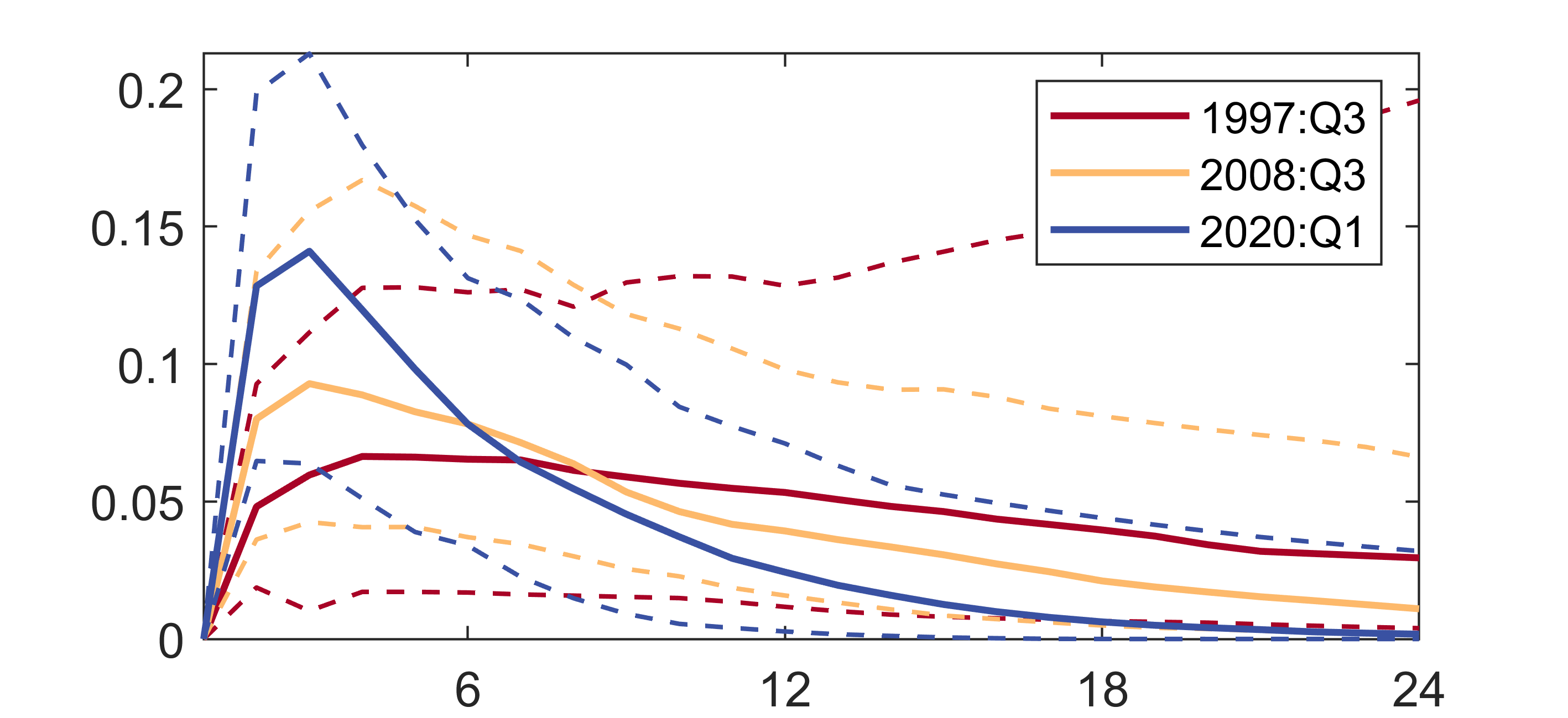

Supplement: Supplementary file 6 [file Data_Sheet_4.ZIP › COM_CHN (3).tif]

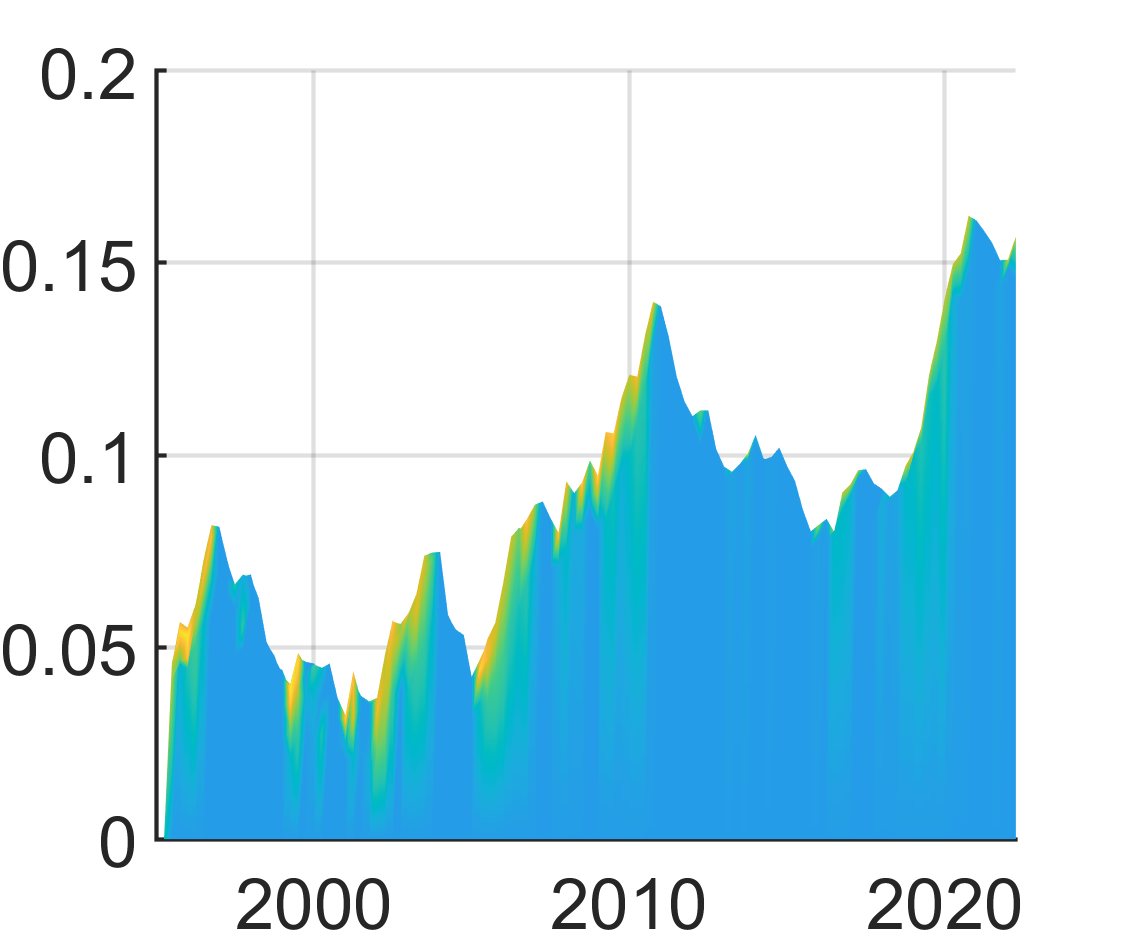

Supplement: Supplementary file 6 [file Data_Sheet_4.ZIP › COM_CHN (4).tif]

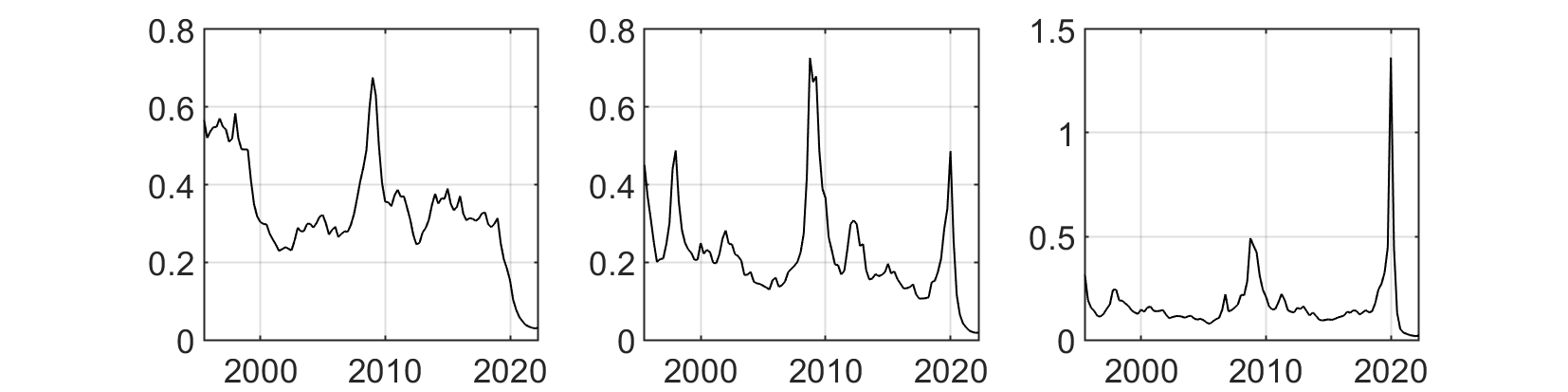

Supplement: Supplementary file 6 [file Data_Sheet_4.ZIP › COM_CHN (5).tif]

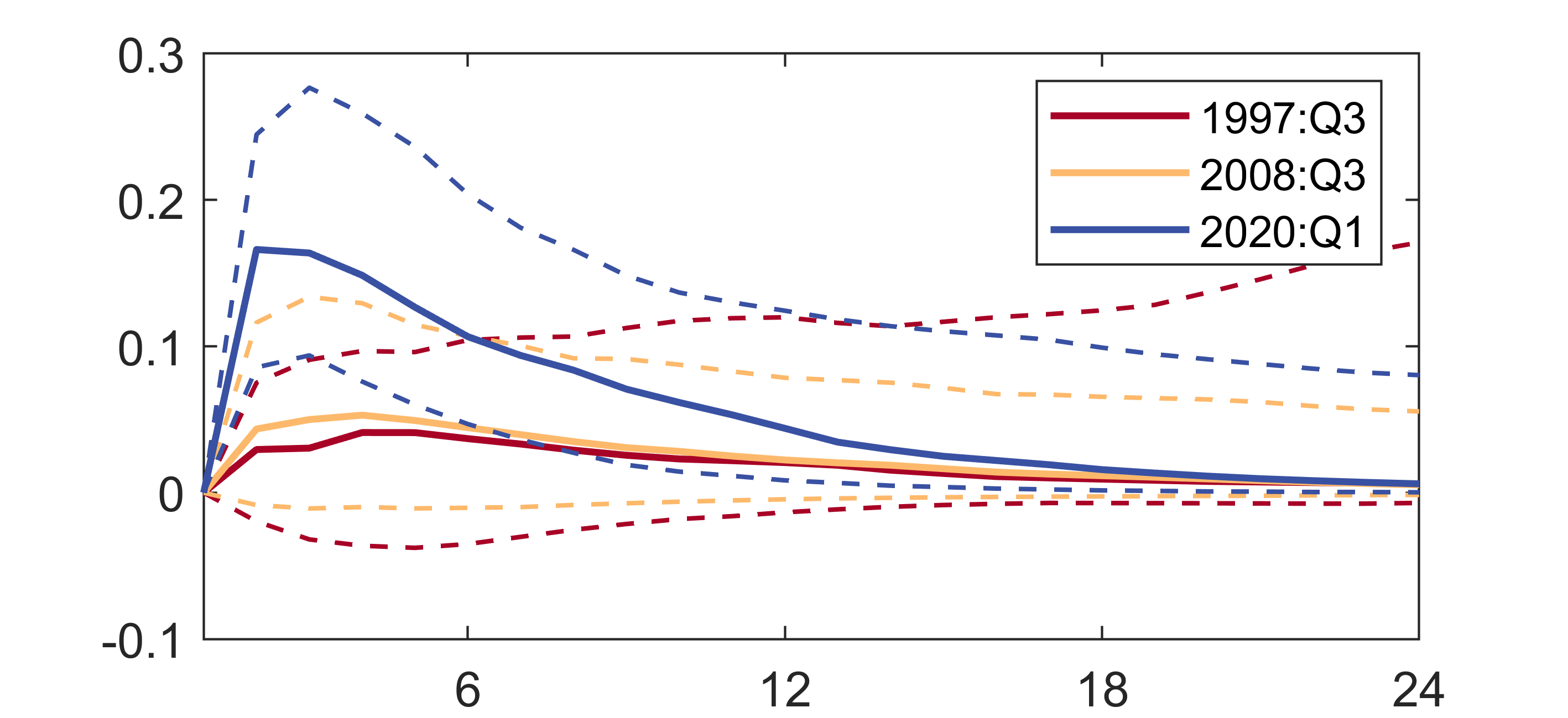

Supplement: Supplementary file 6 [file Data_Sheet_4.ZIP › COM_HK (3).tif]

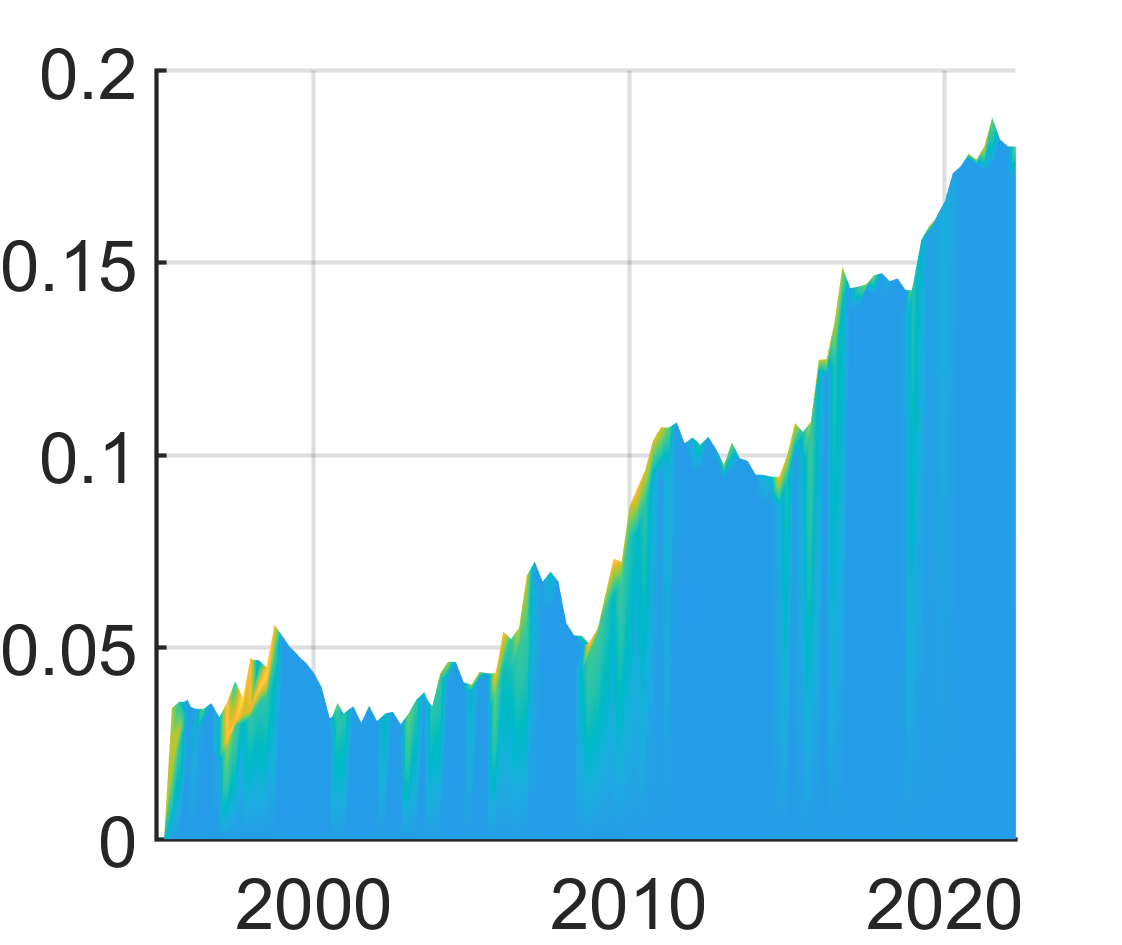

Supplement: Supplementary file 6 [file Data_Sheet_4.ZIP › COM_HK (4).tif]

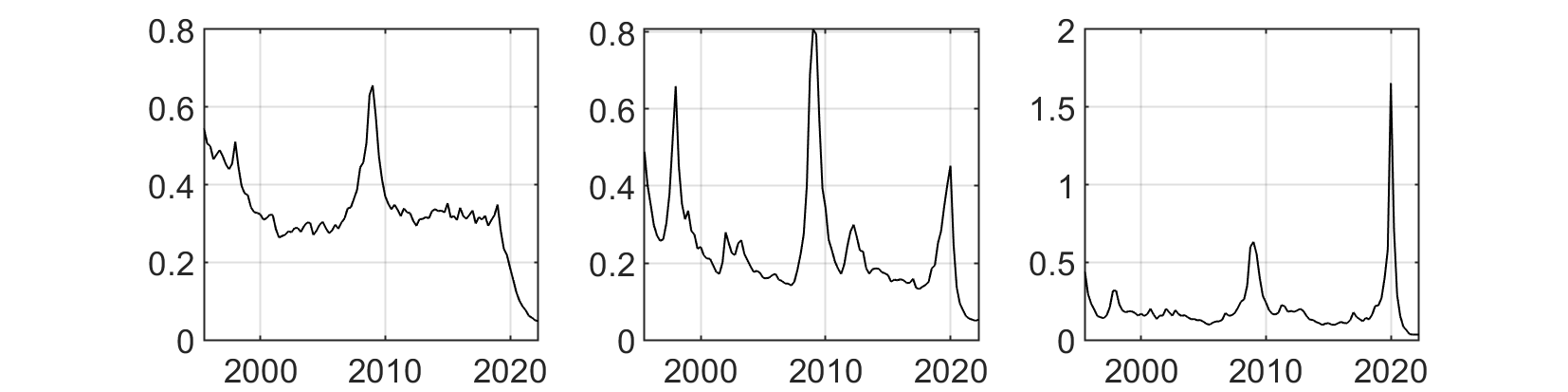

Supplement: Supplementary file 6 [file Data_Sheet_4.ZIP › COM_HK (5).tif]

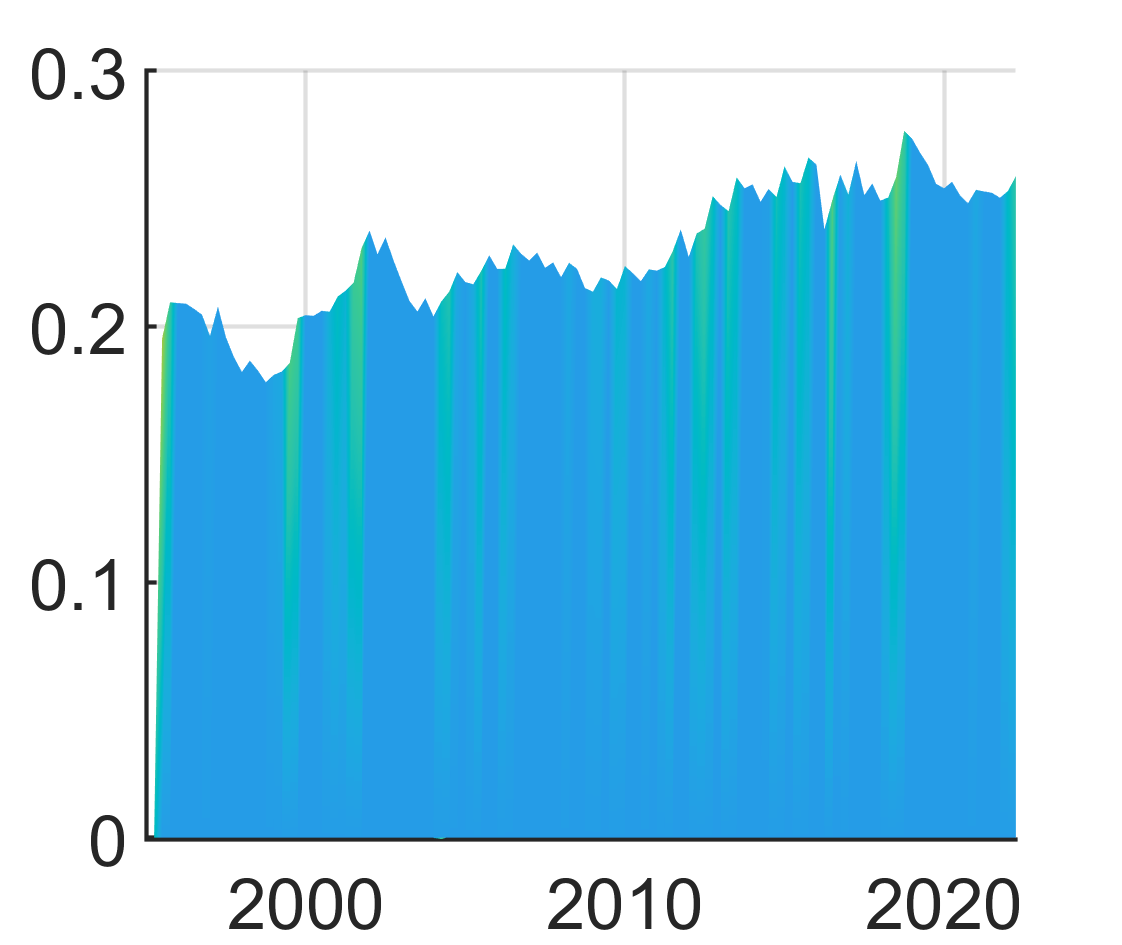

Supplement: Supplementary file 6 [file Data_Sheet_4.ZIP › COM_JPN (1).tif]

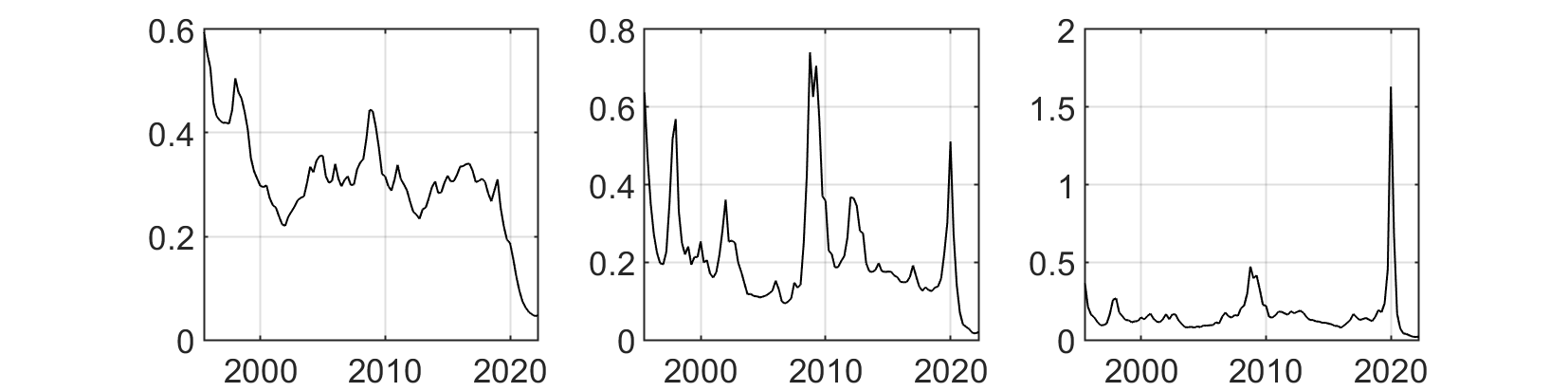

Supplement: Supplementary file 6 [file Data_Sheet_4.ZIP › COM_JPN (2).tif]

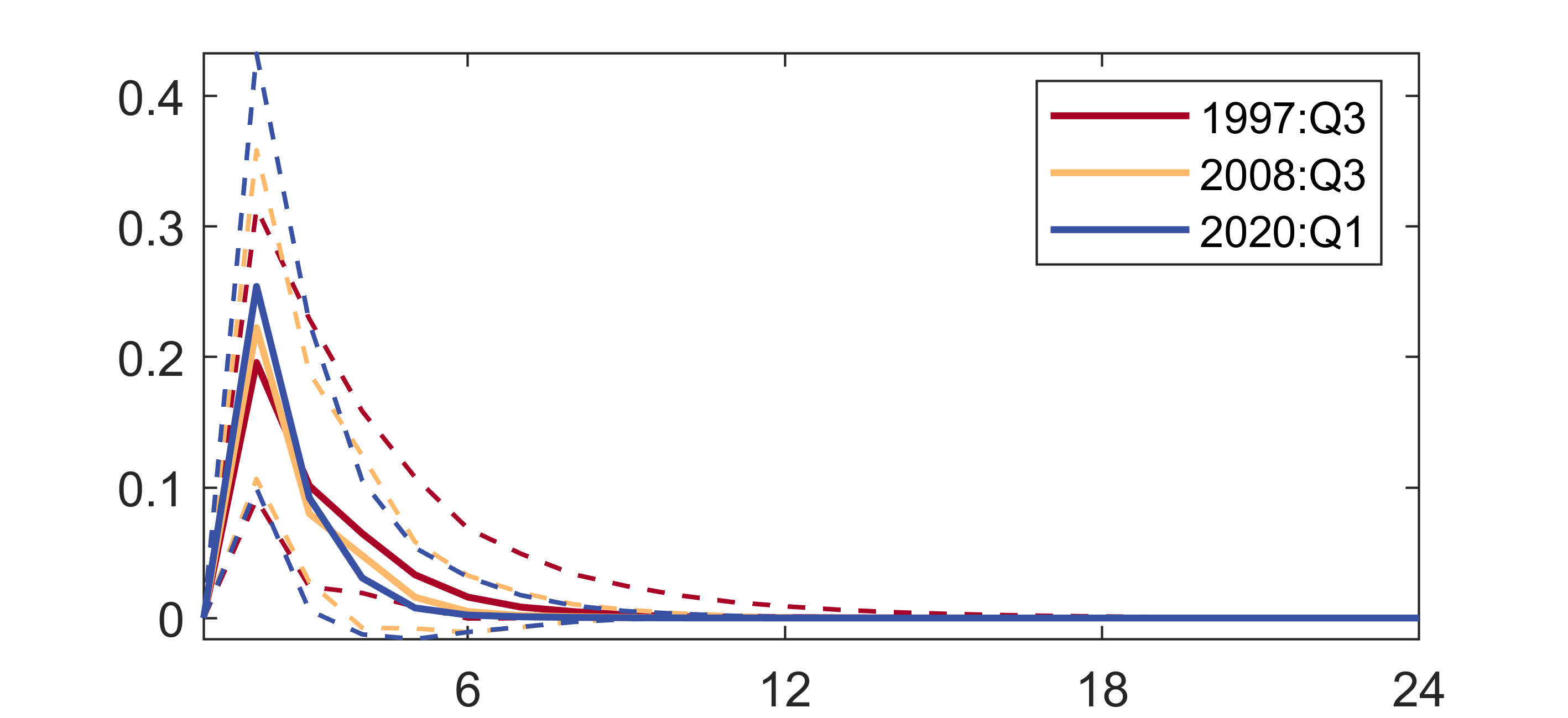

Supplement: Supplementary file 6 [file Data_Sheet_4.ZIP › COM_JPN (3).tif]

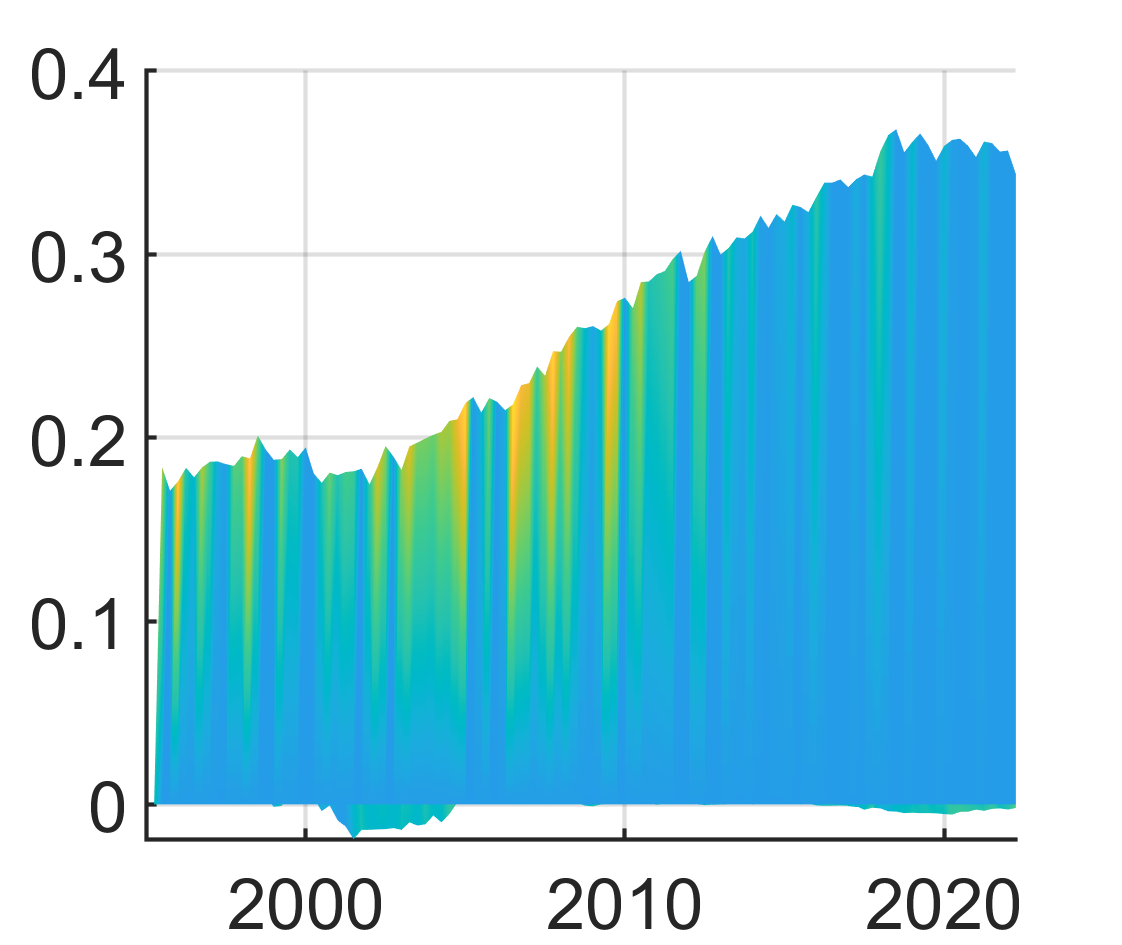

Supplement: Supplementary file 6 [file Data_Sheet_4.ZIP › COM_KR (1).tif]

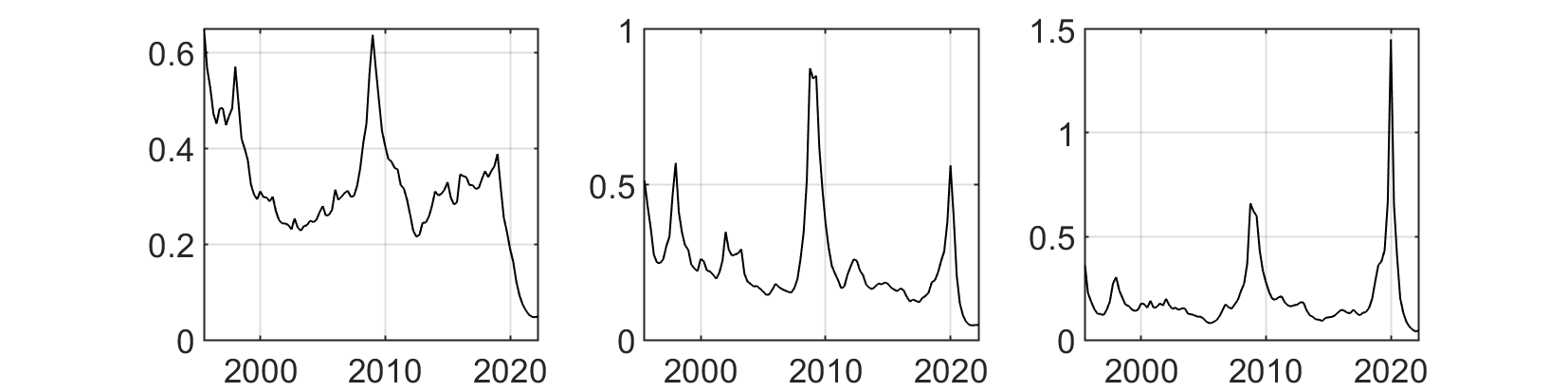

Supplement: Supplementary file 6 [file Data_Sheet_4.ZIP › COM_KR (2).tif]

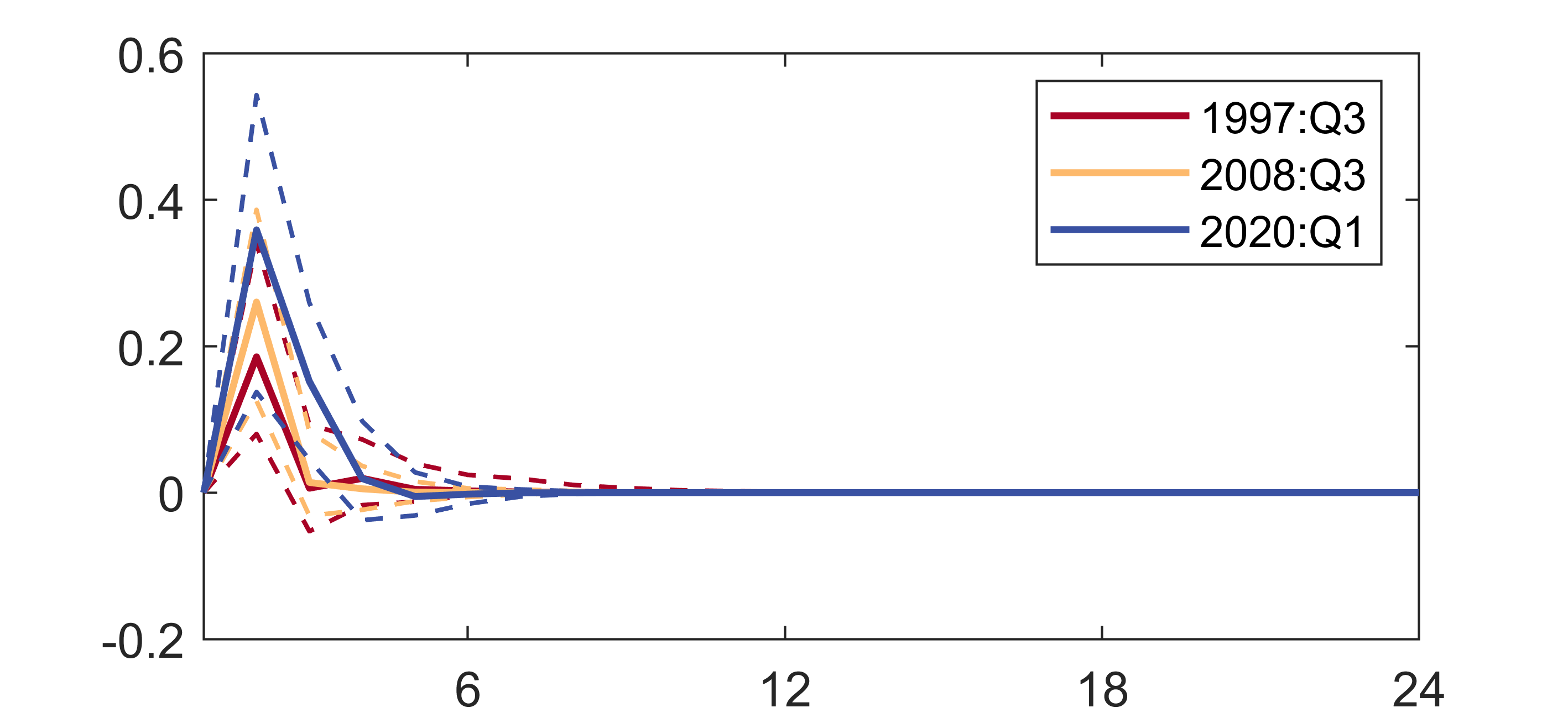

Supplement: Supplementary file 6 [file Data_Sheet_4.ZIP › COM_KR (3).tif]

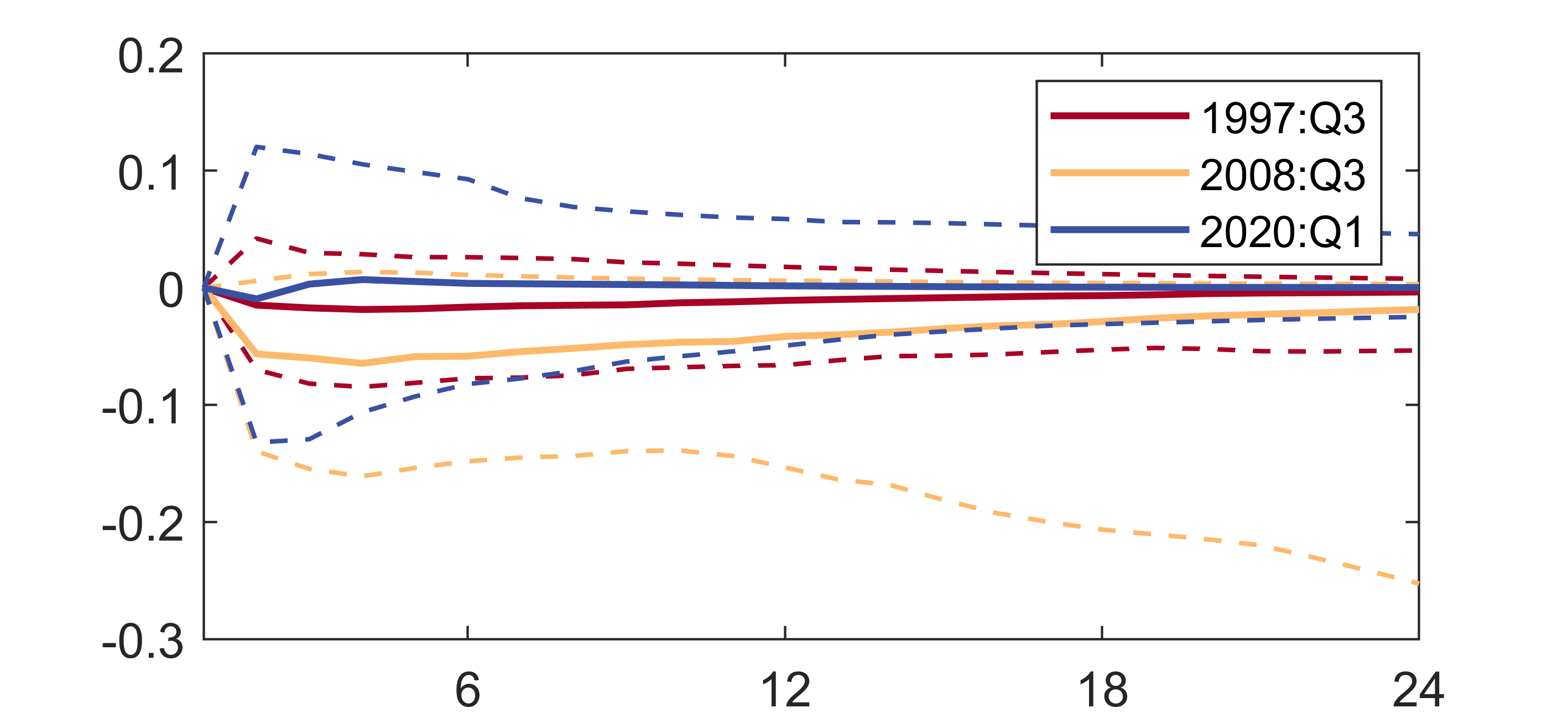

Supplement: Supplementary file 6 [file Data_Sheet_4.ZIP › FEM_CHN (1).tif]

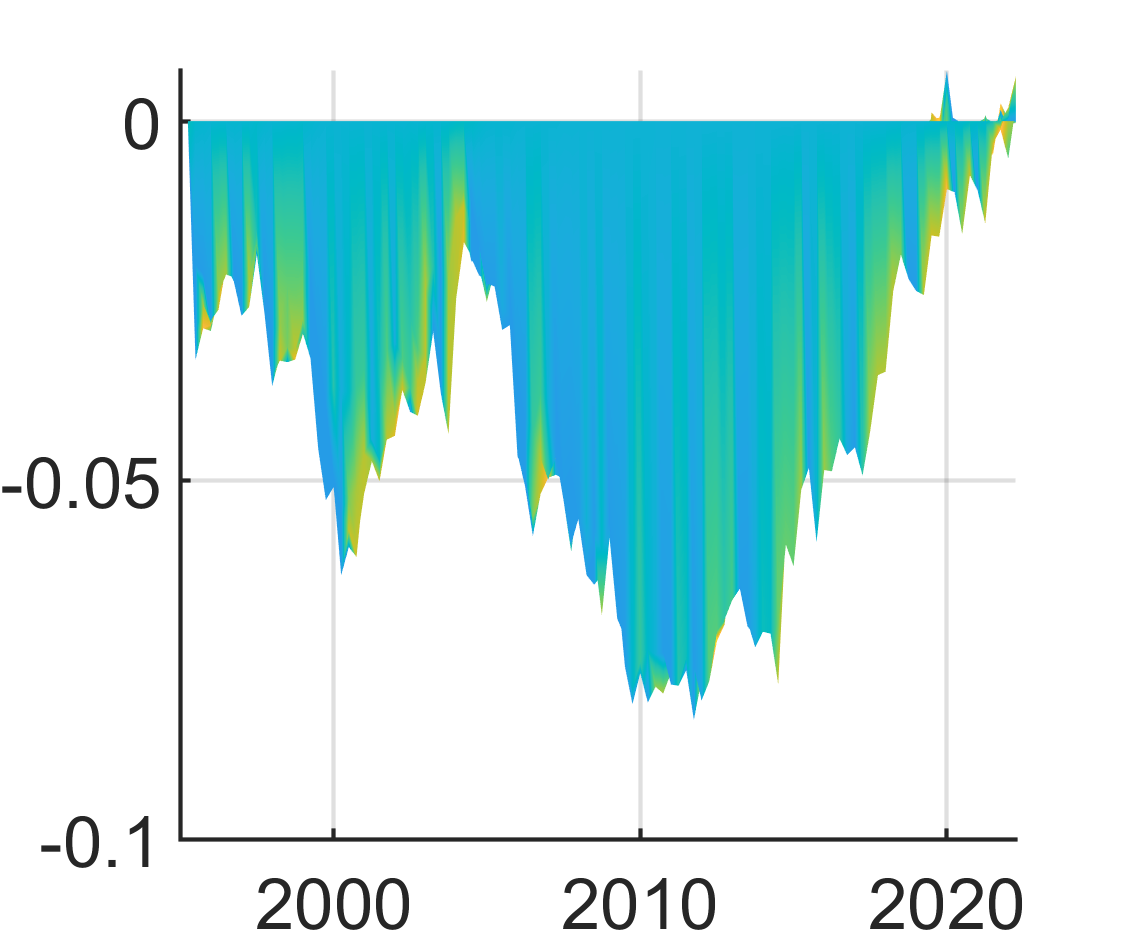

Supplement: Supplementary file 6 [file Data_Sheet_4.ZIP › FEM_CHN (2).tif]

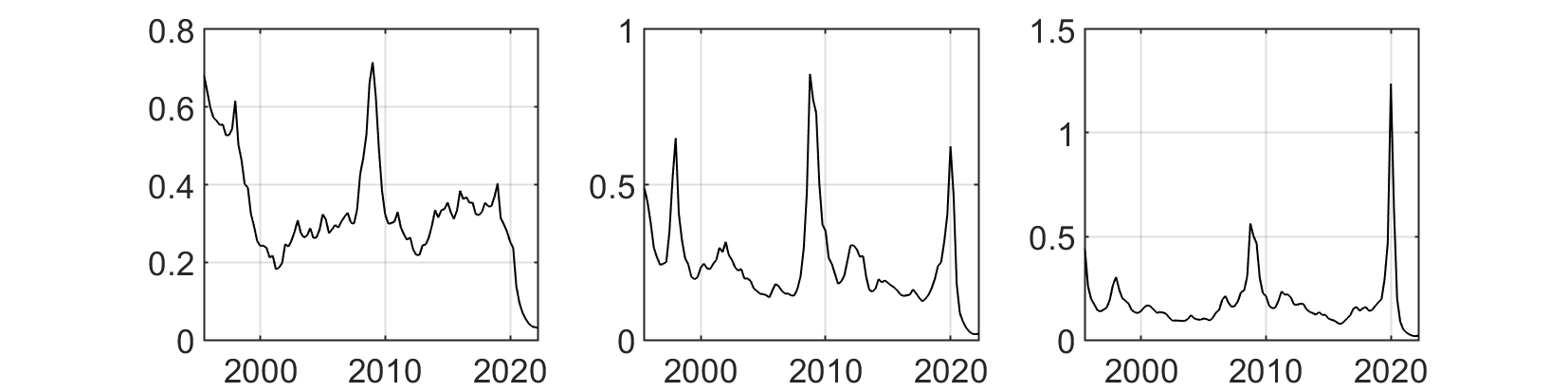

Supplement: Supplementary file 6 [file Data_Sheet_4.ZIP › FEM_CHN (3).tif]

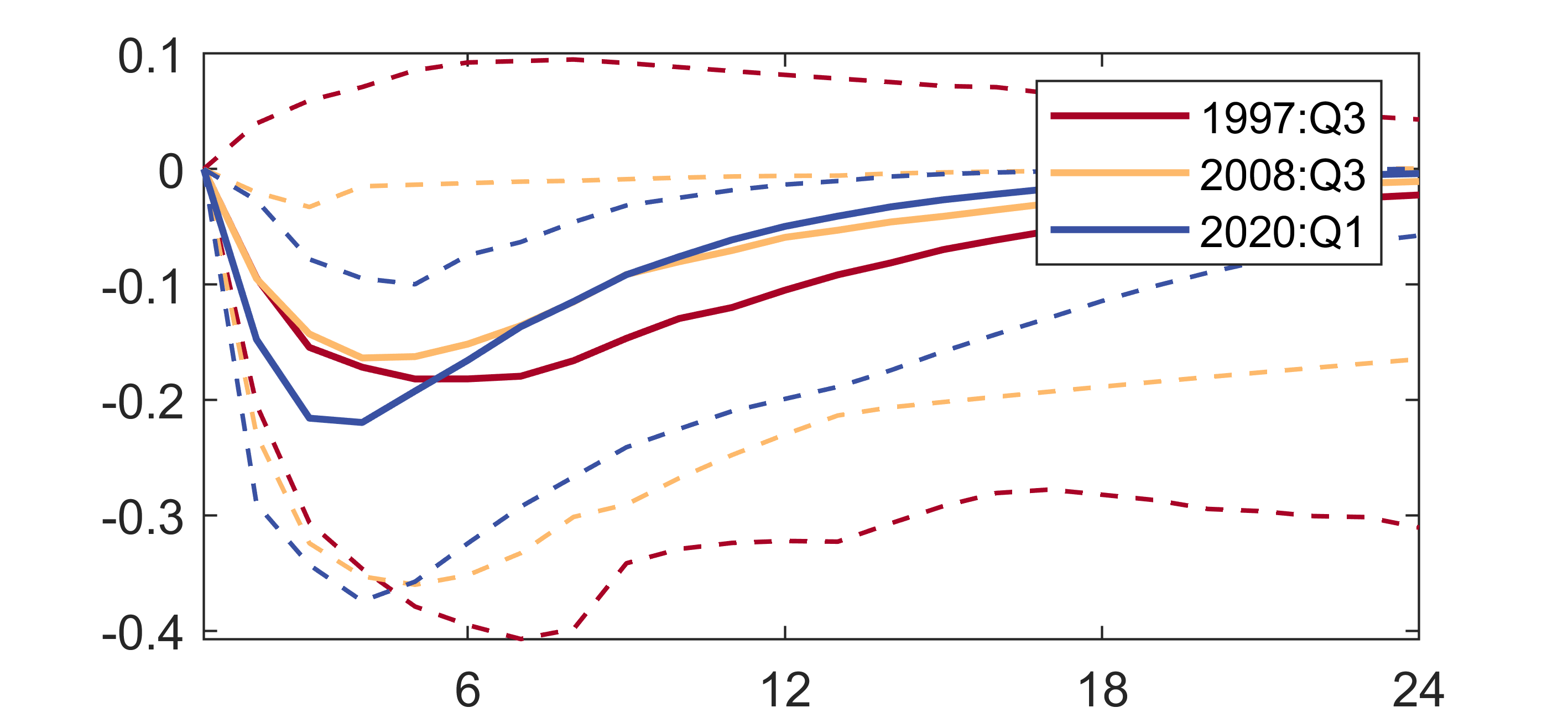

Supplement: Supplementary file 6 [file Data_Sheet_4.ZIP › FEM_HK (1).tif]

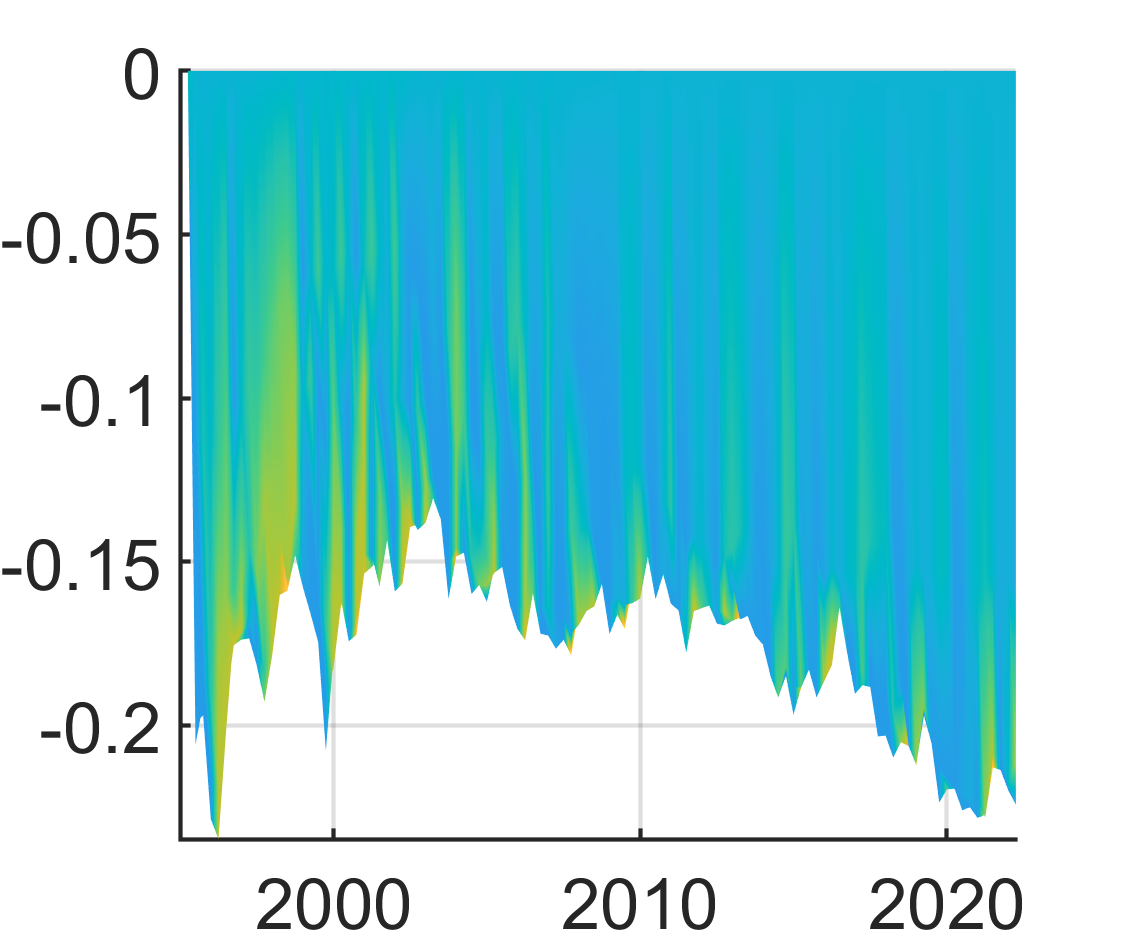

Supplement: Supplementary file 6 [file Data_Sheet_4.ZIP › FEM_HK (2).tif]

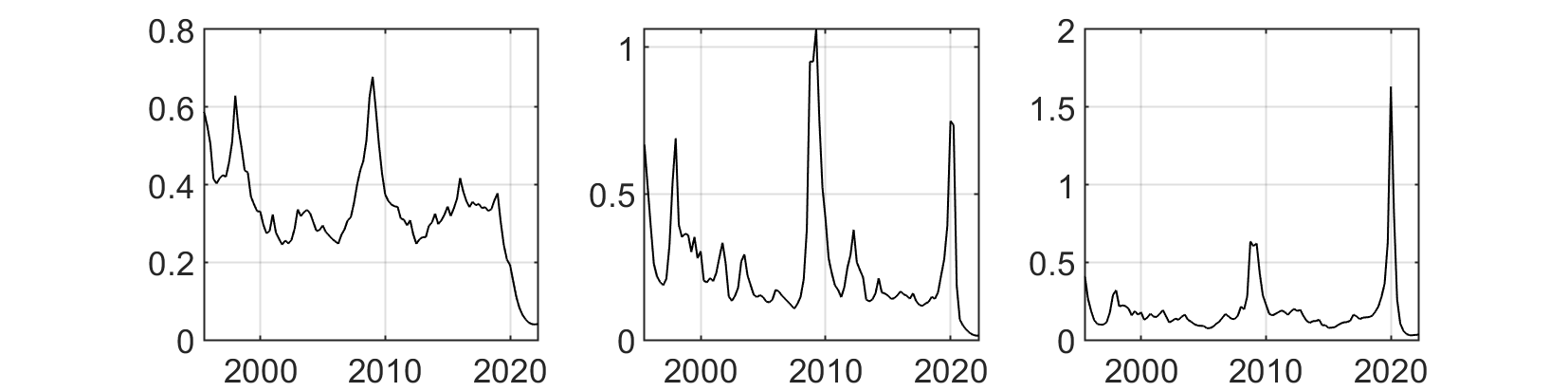

Supplement: Supplementary file 6 [file Data_Sheet_4.ZIP › FEM_HK (3).tif]

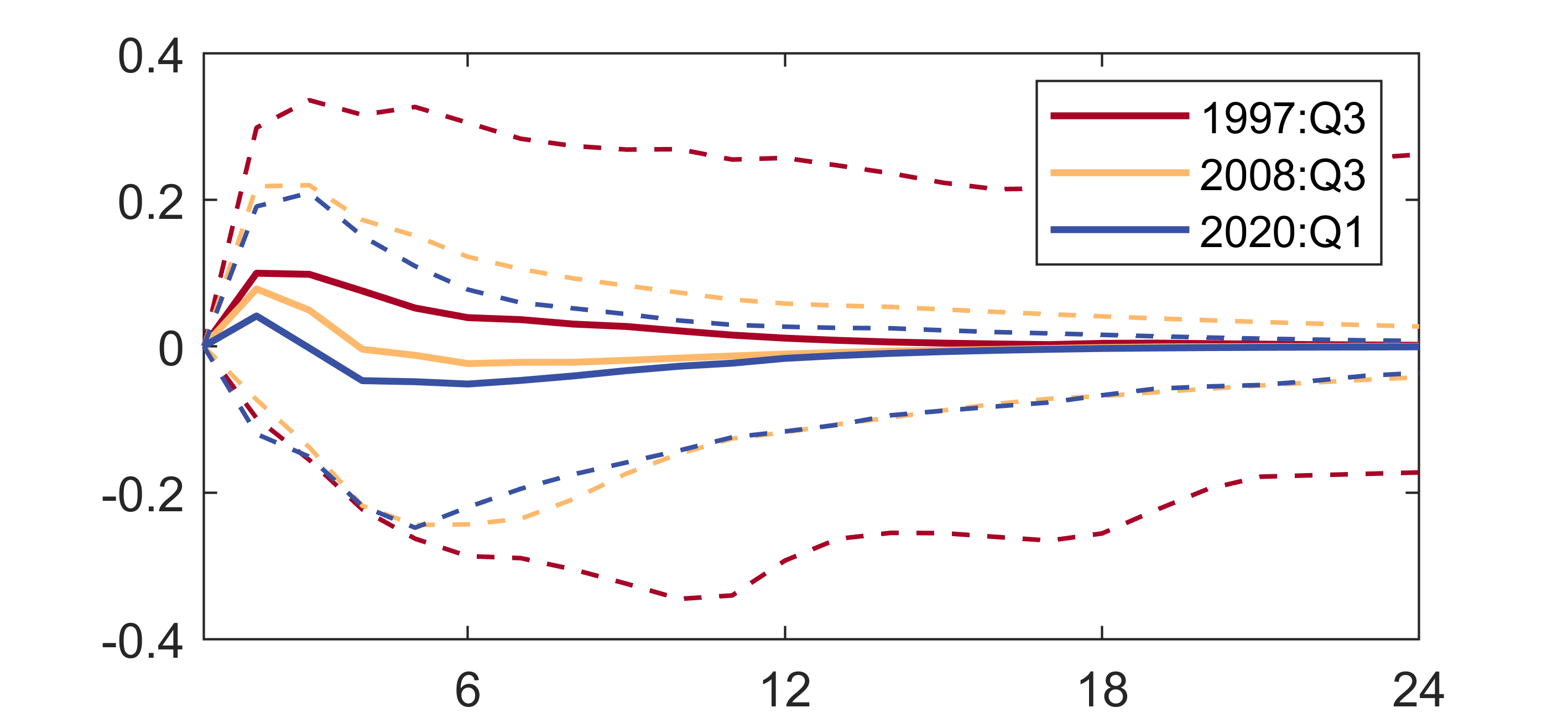

Supplement: Supplementary file 6 [file Data_Sheet_4.ZIP › FEM_JPN (1).tif]

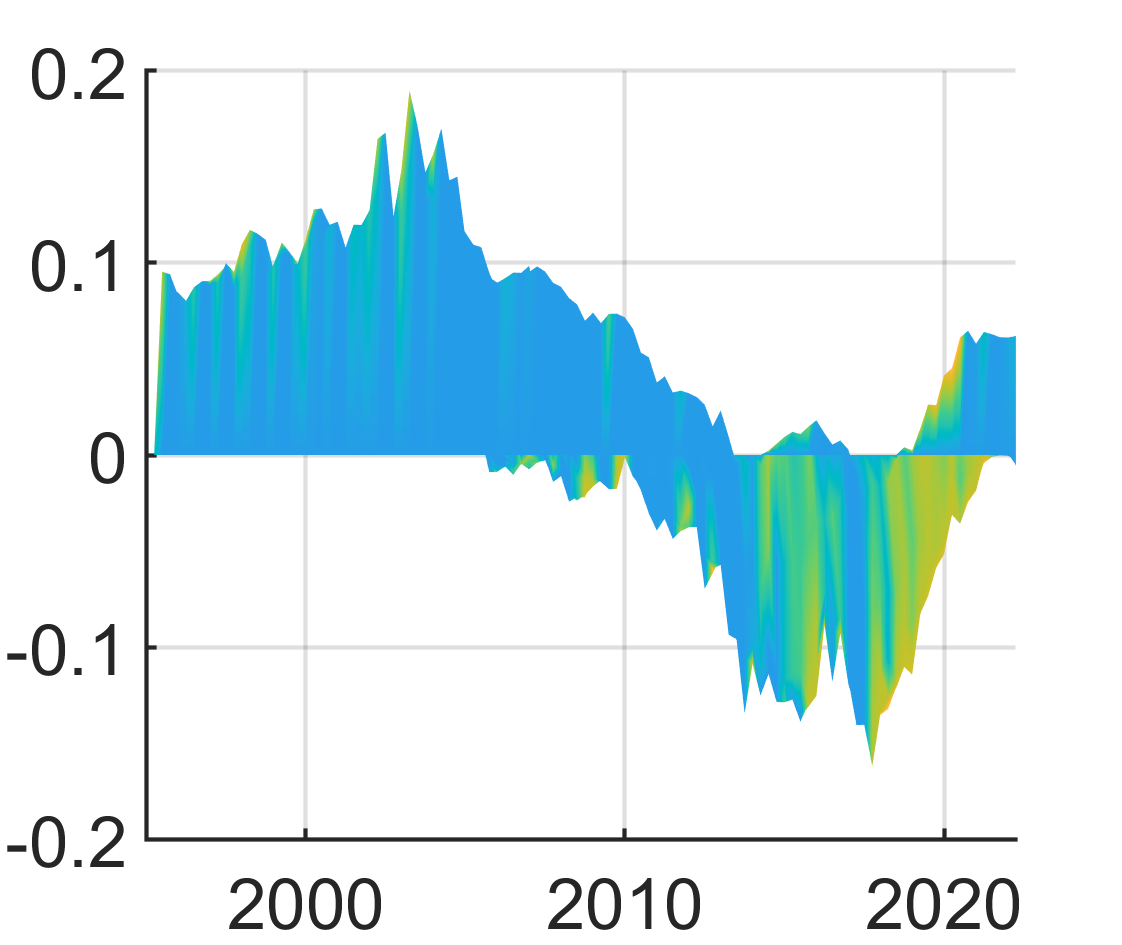

Supplement: Supplementary file 6 [file Data_Sheet_4.ZIP › FEM_JPN (2).tif]

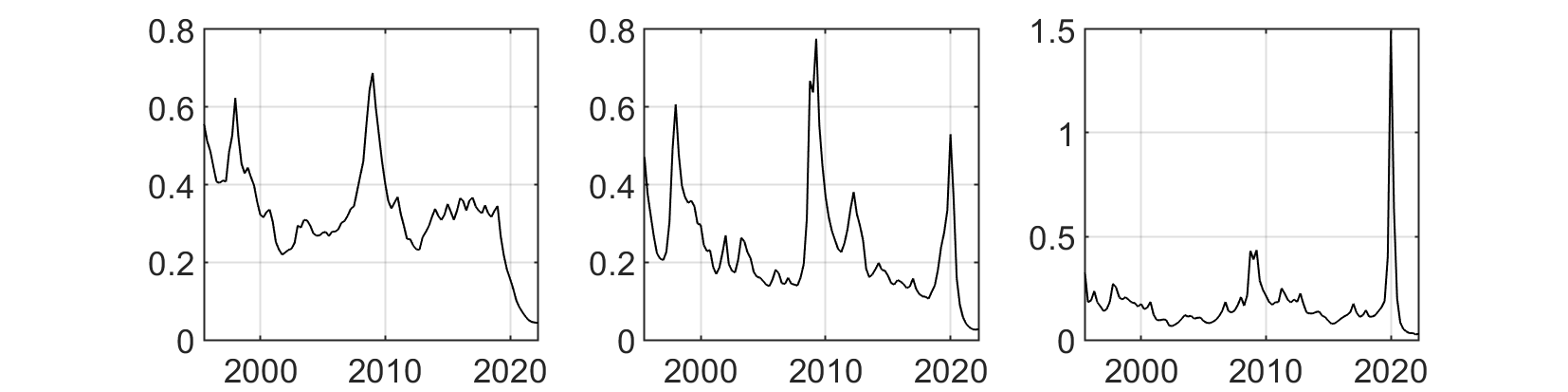

Supplement: Supplementary file 6 [file Data_Sheet_4.ZIP › FEM_JPN (3).tif]

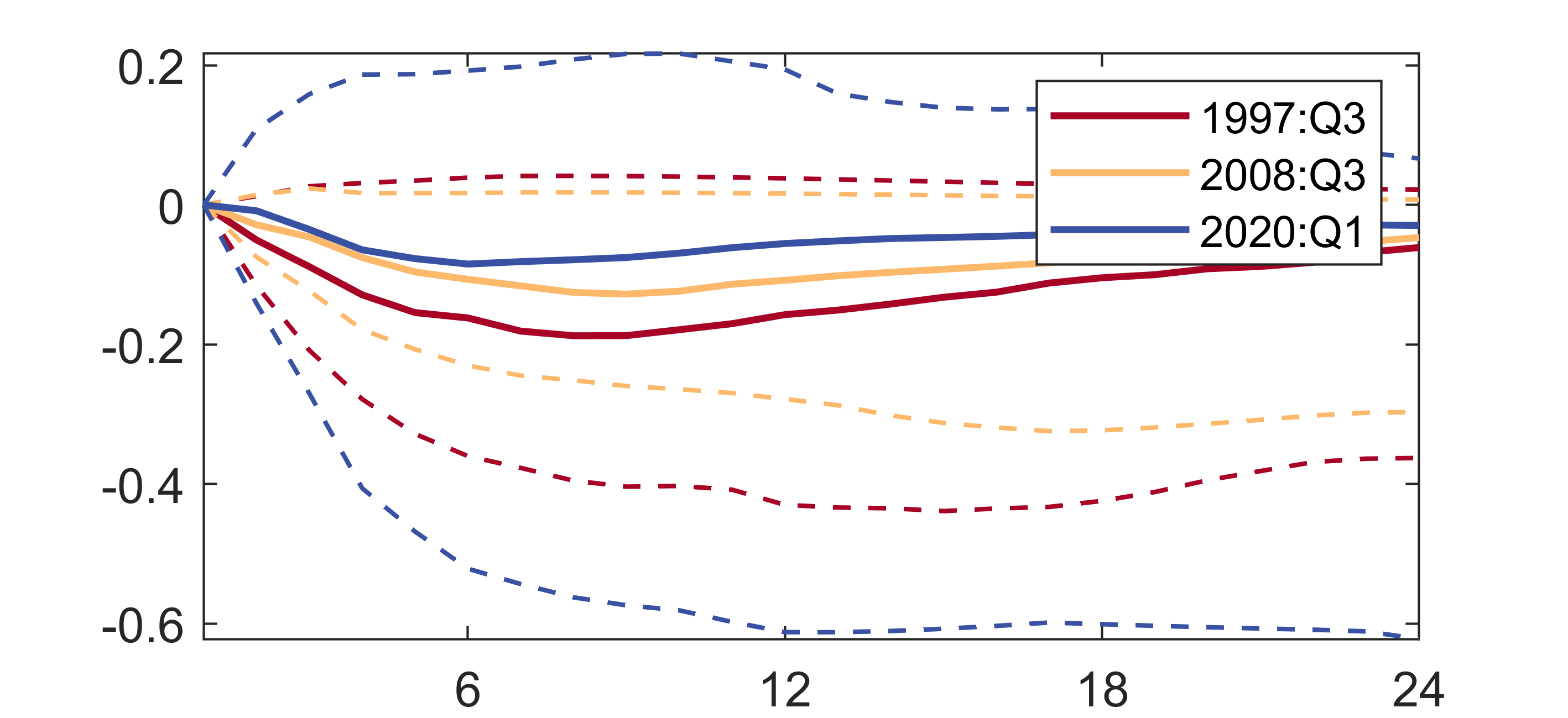

Supplement: Supplementary file 6 [file Data_Sheet_4.ZIP › FEM_KR (1).tif]

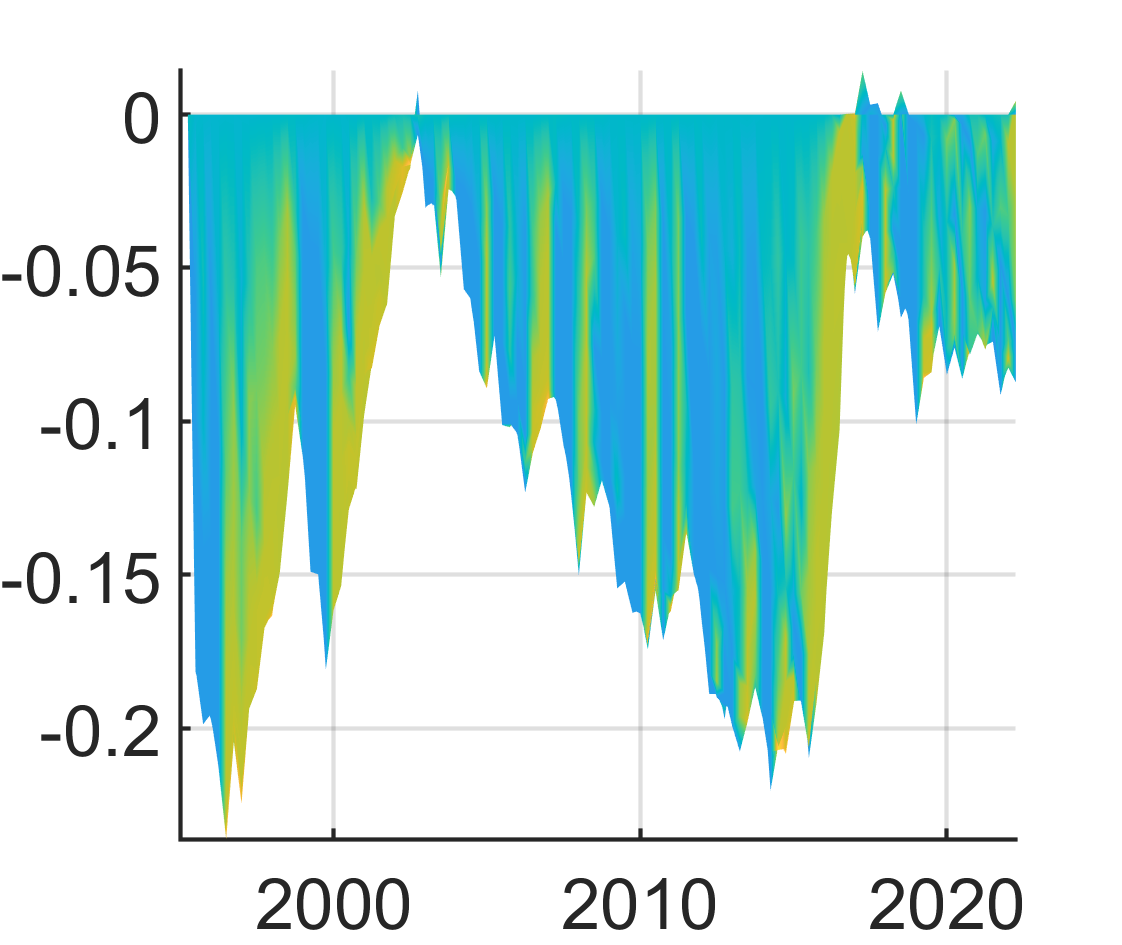

Supplement: Supplementary file 6 [file Data_Sheet_4.ZIP › FEM_KR (2).tif]

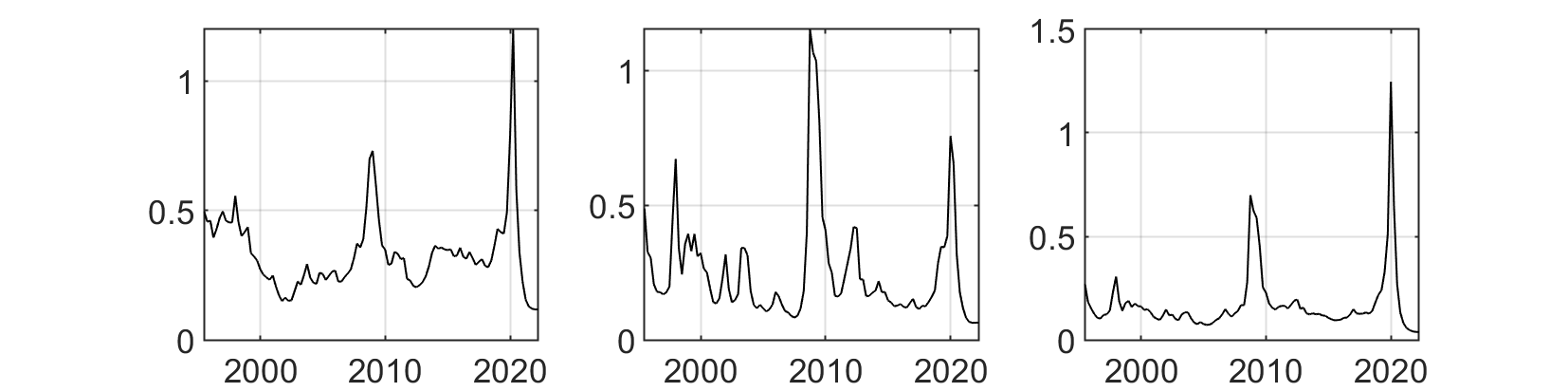

Supplement: Supplementary file 6 [file Data_Sheet_4.ZIP › FEM_KR (3).tif]

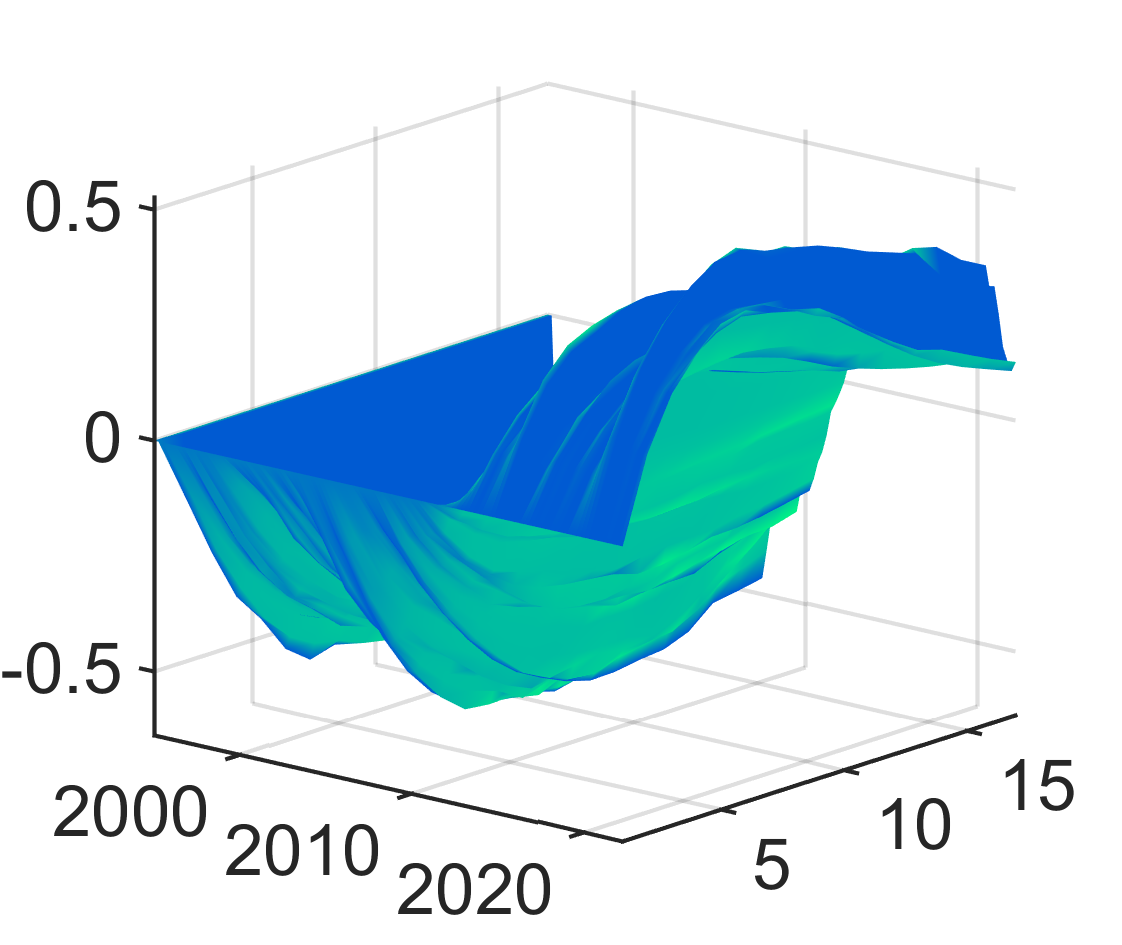

Supplement: Supplementary file 6 [file Data_Sheet_4.ZIP › HK_JPN (1).tif]

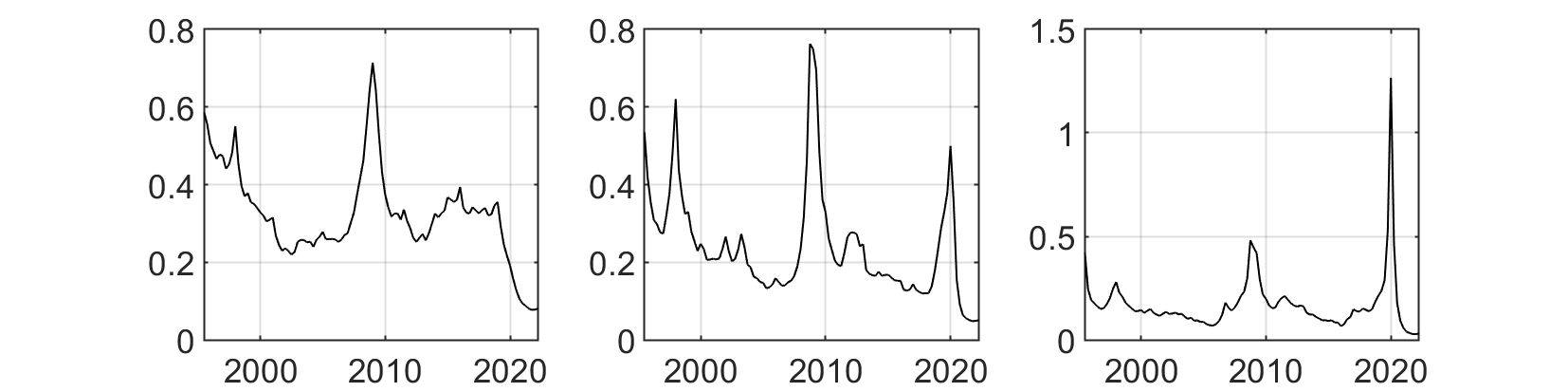

Supplement: Supplementary file 6 [file Data_Sheet_4.ZIP › HK_JPN (2).tif]

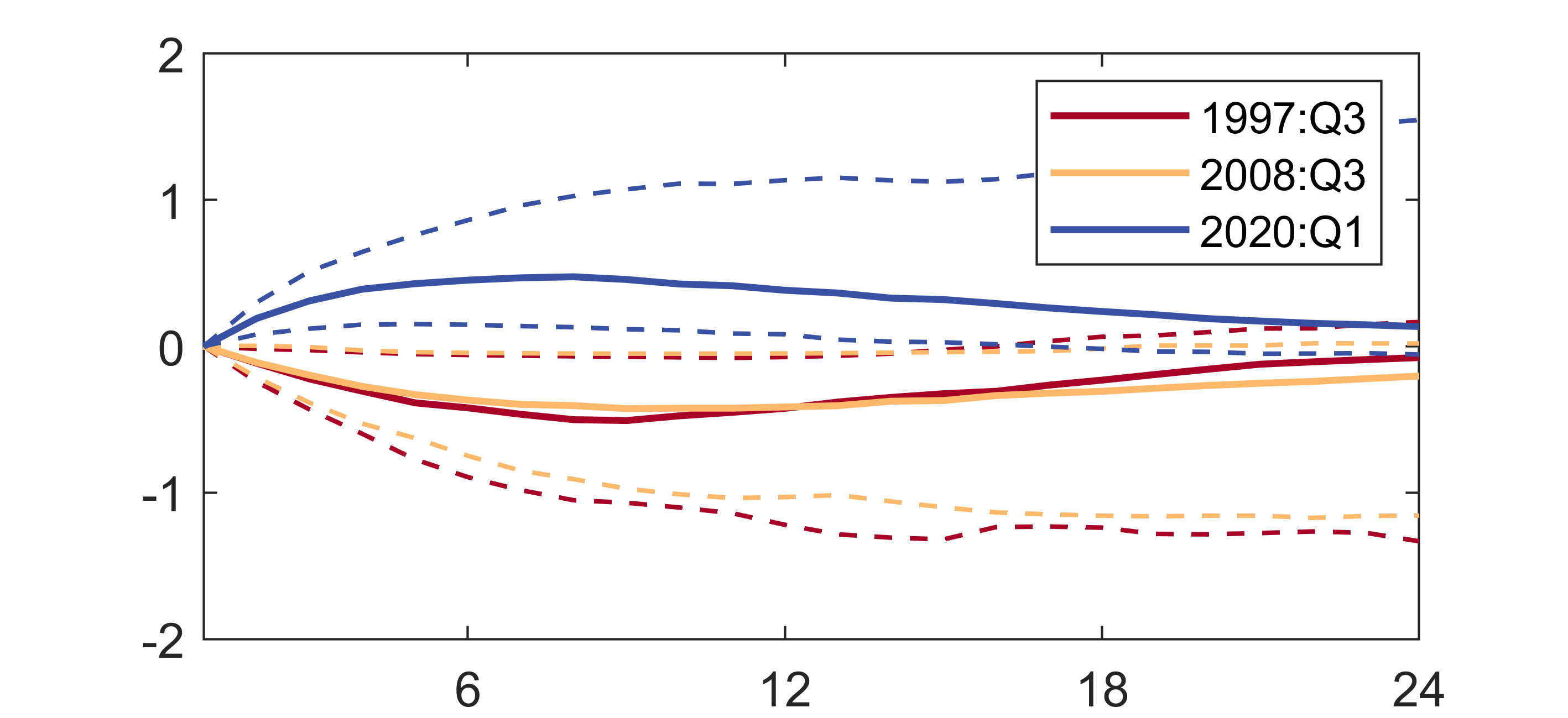

Supplement: Supplementary file 6 [file Data_Sheet_4.ZIP › HK_JPN (3).tif]

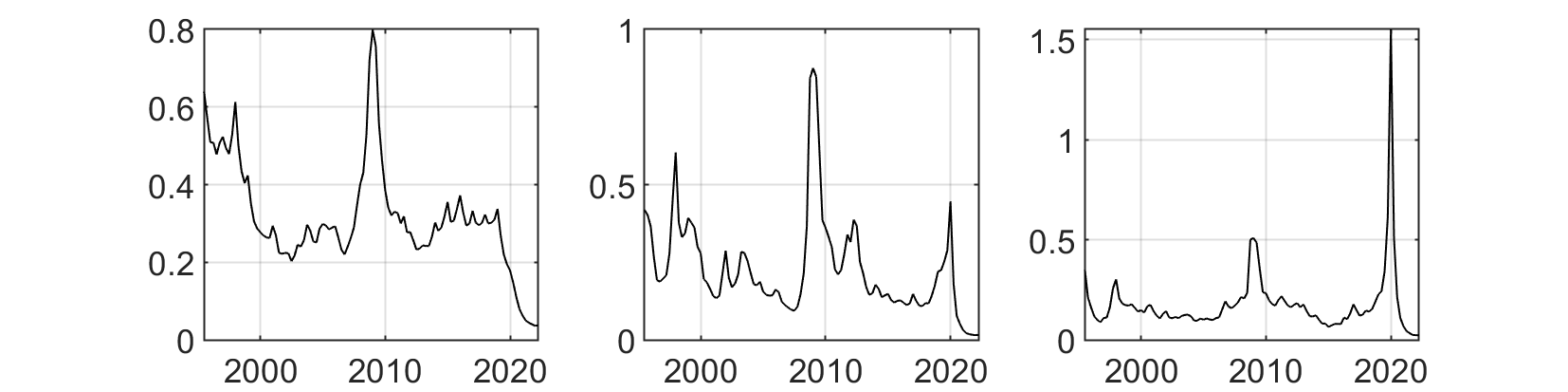

Supplement: Supplementary file 6 [file Data_Sheet_4.ZIP › HK_to_China (1).tif]

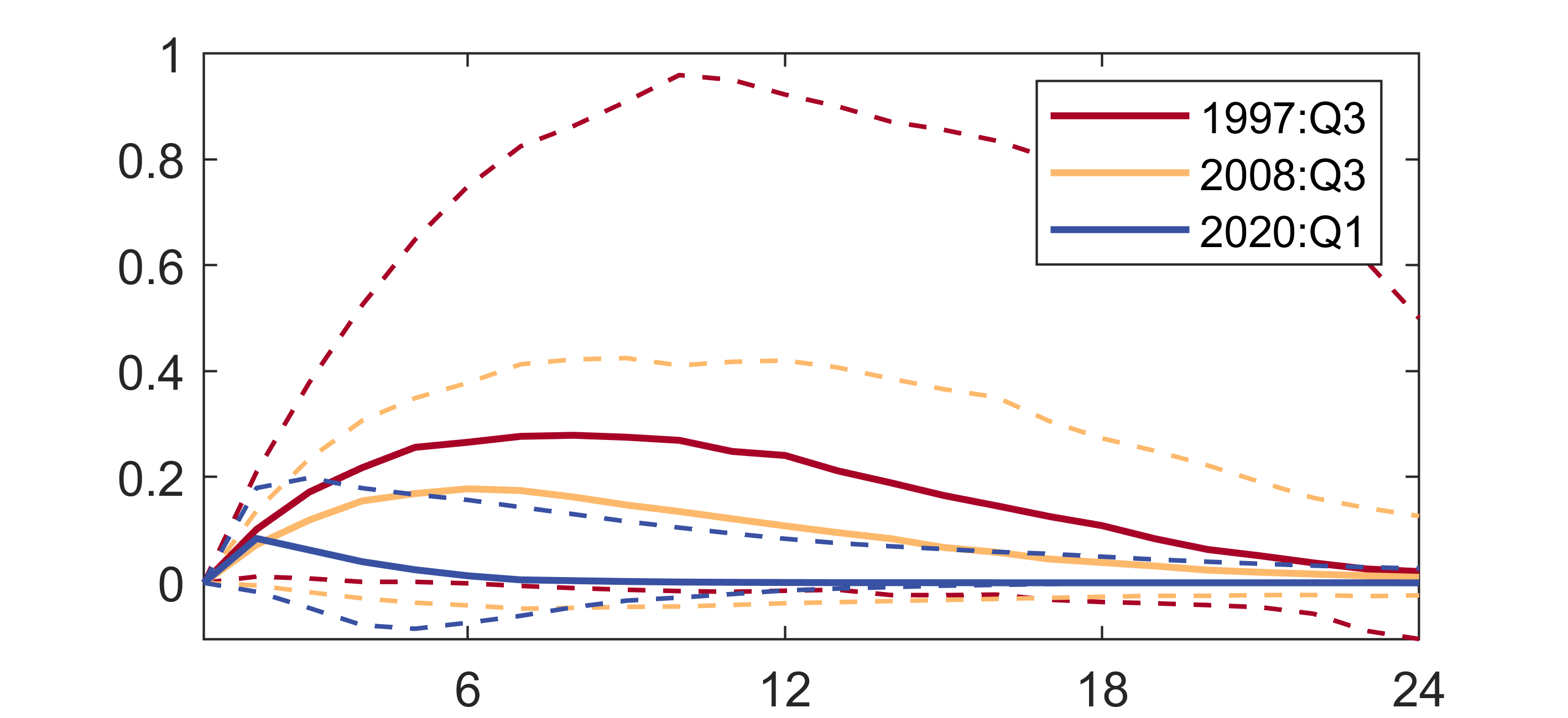

Supplement: Supplementary file 6 [file Data_Sheet_4.ZIP › HK_to_China (2).tif]

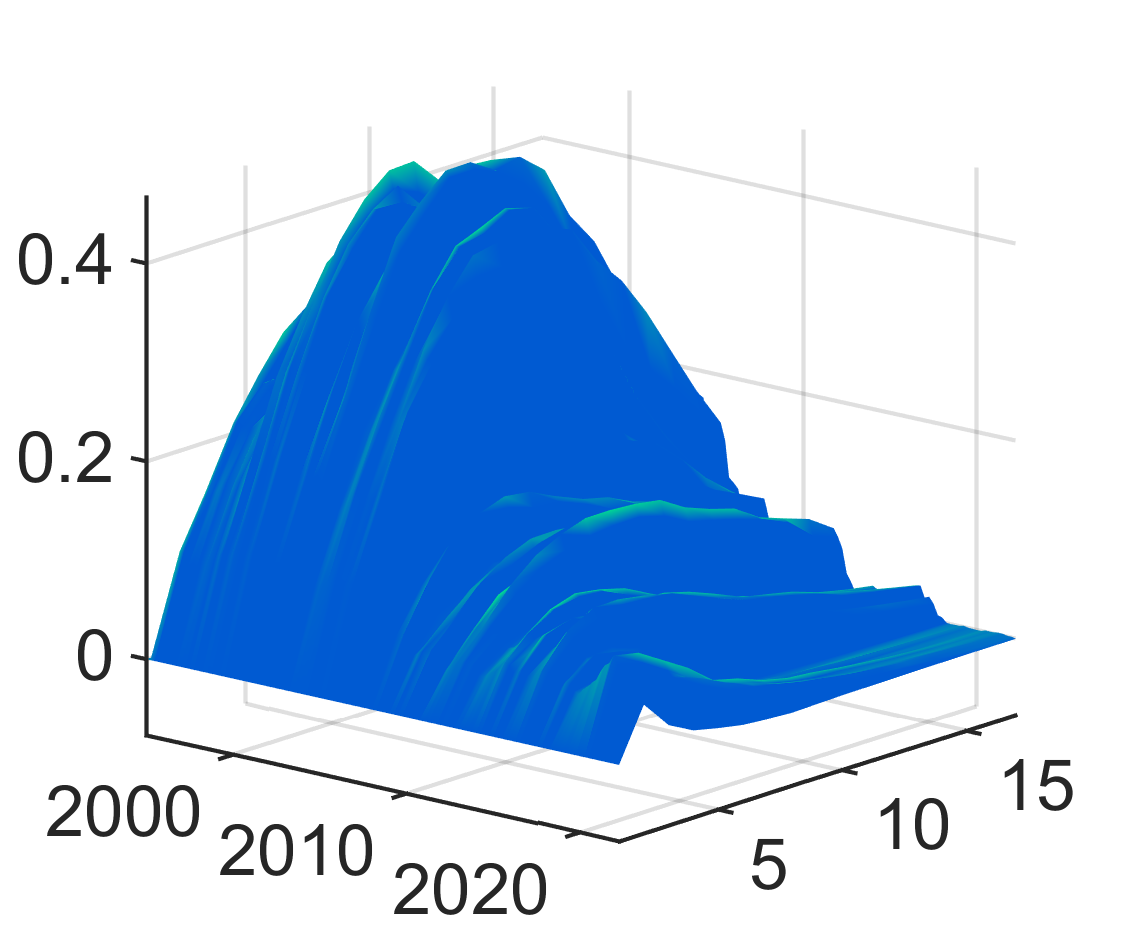

Supplement: Supplementary file 6 [file Data_Sheet_4.ZIP › HK_to_China (3).tif]

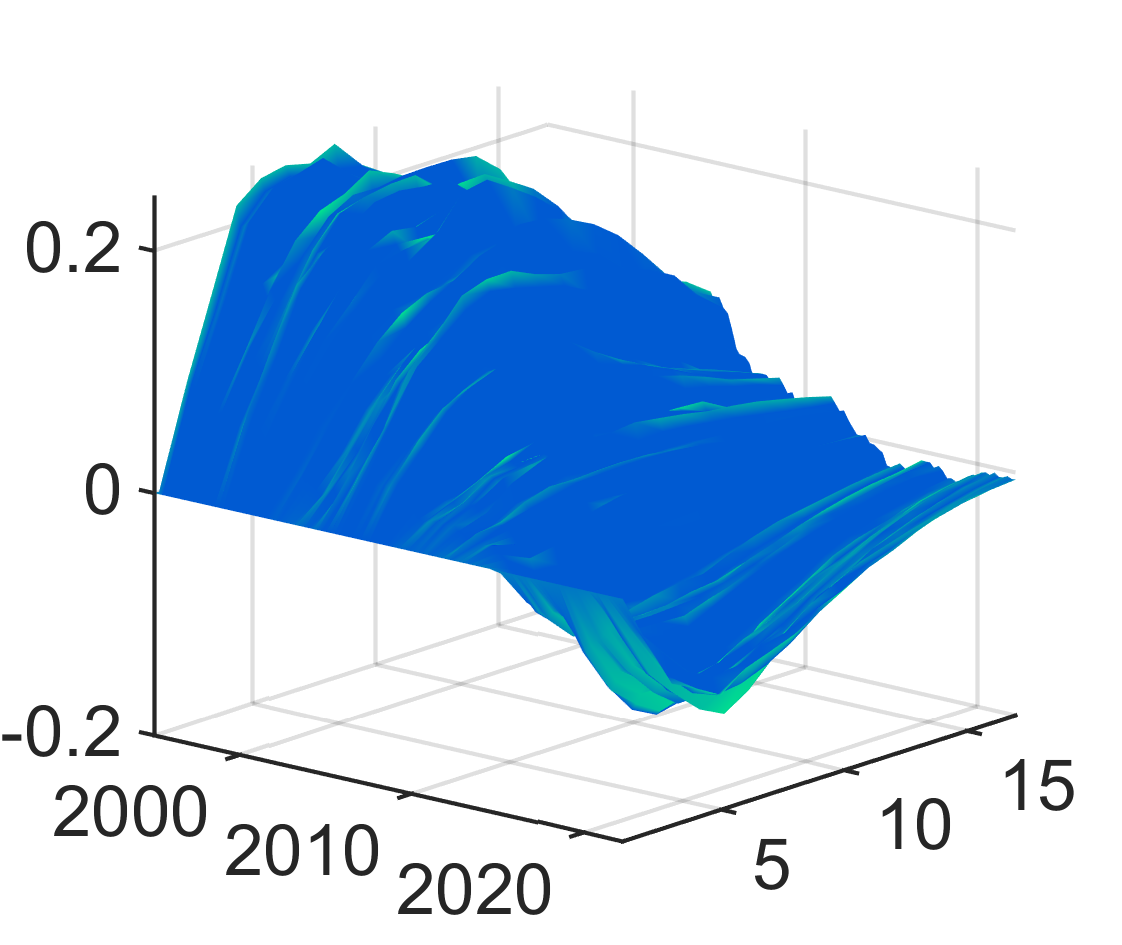

Supplement: Supplementary file 6 [file Data_Sheet_4.ZIP › HR_KR (1).tif]

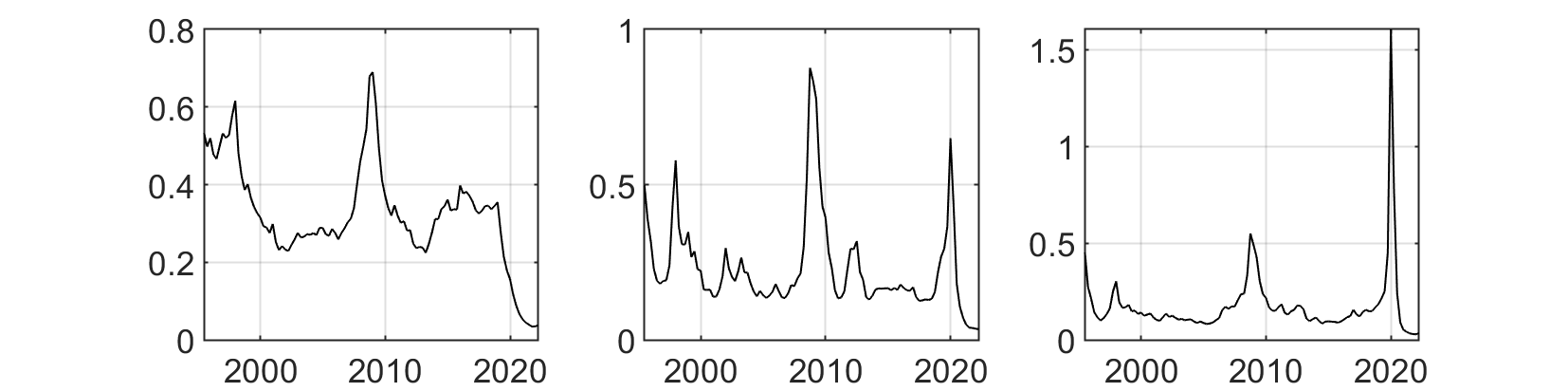

Supplement: Supplementary file 6 [file Data_Sheet_4.ZIP › HR_KR (2).tif]

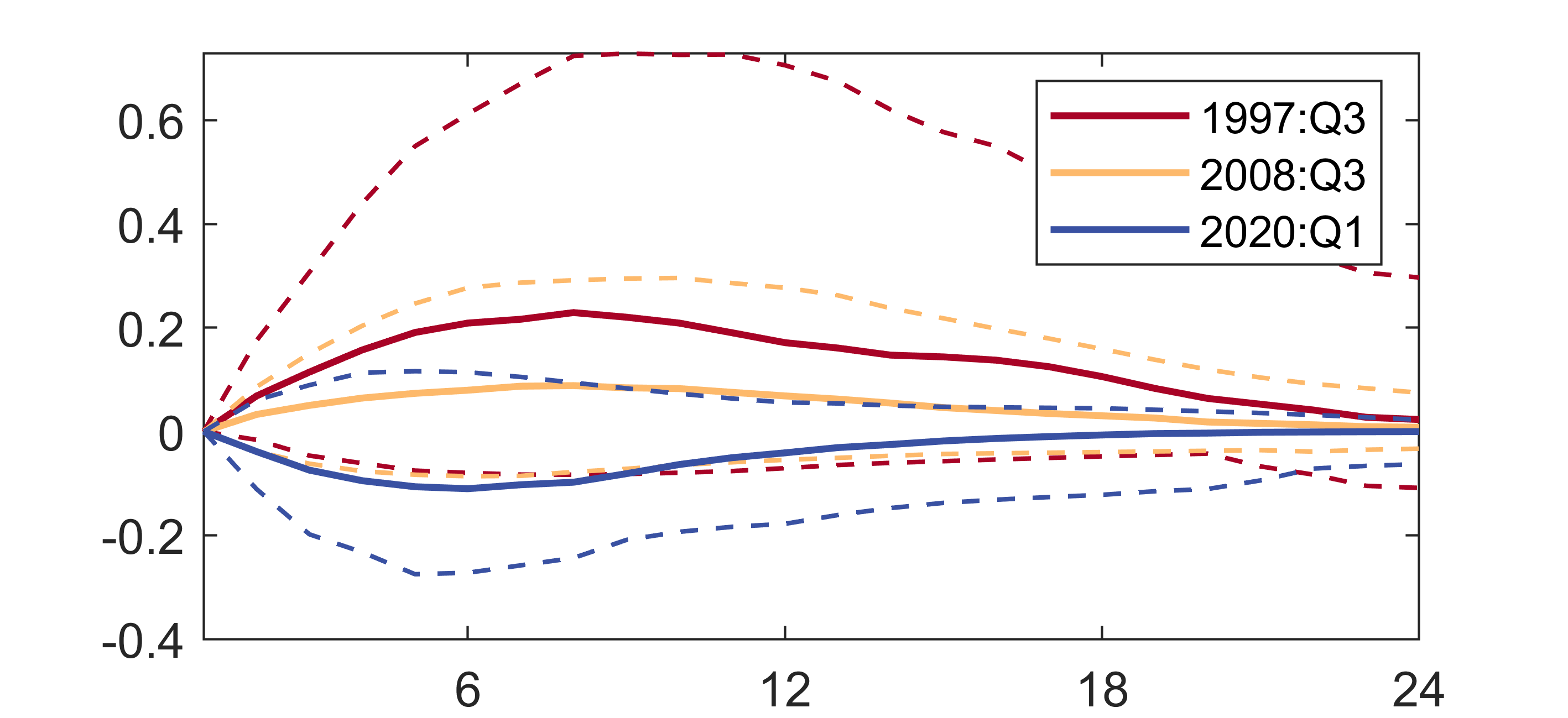

Supplement: Supplementary file 6 [file Data_Sheet_4.ZIP › HR_KR (3).tif]

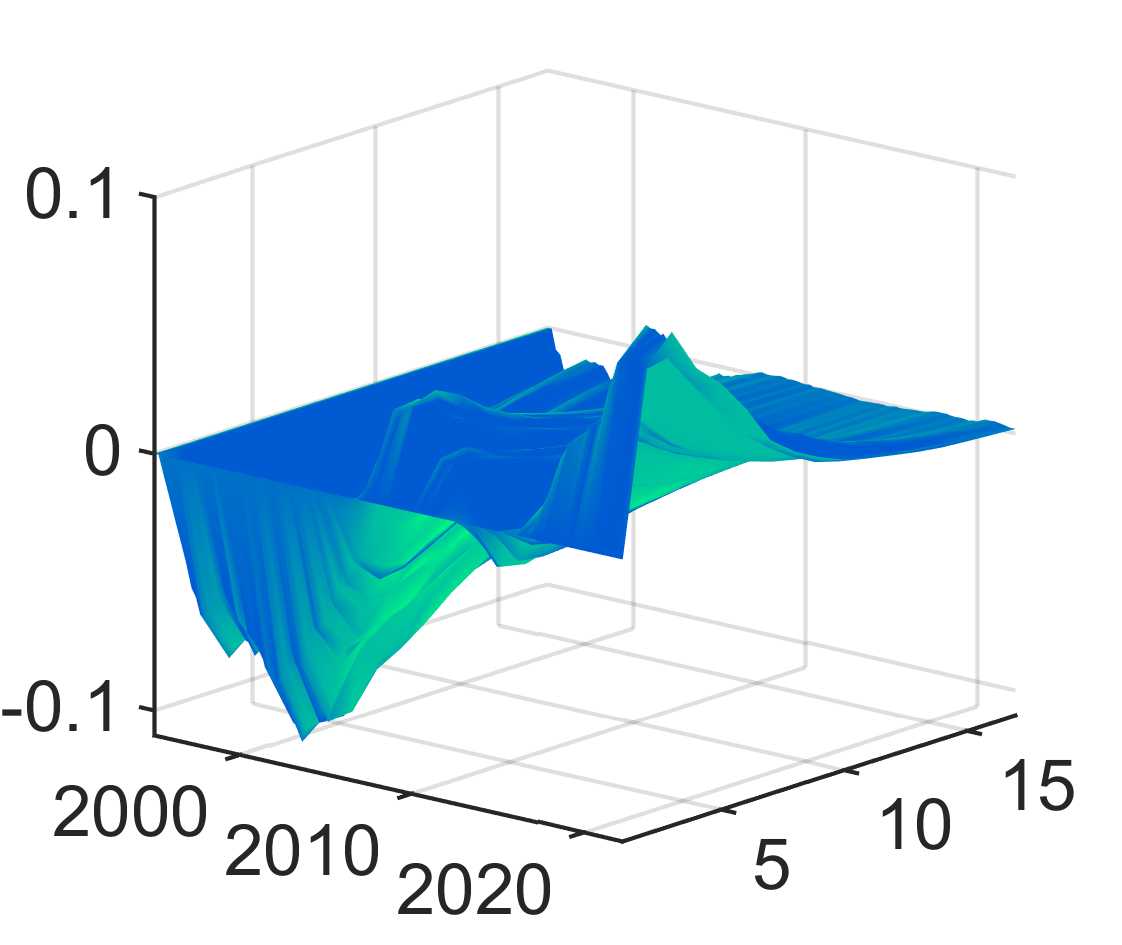

Supplement: Supplementary file 6 [file Data_Sheet_4.ZIP › JPN_CHN (1).tif]

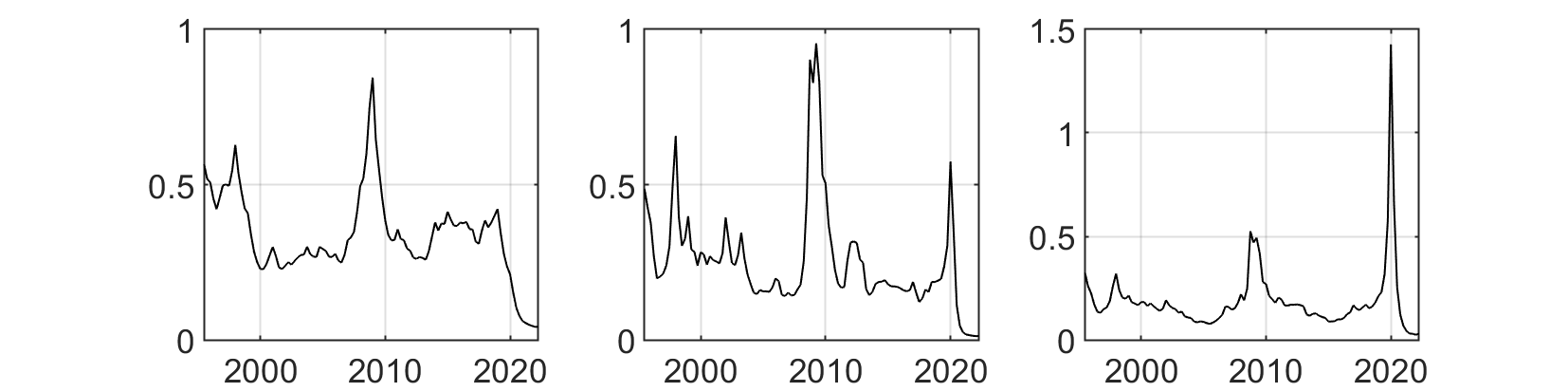

Supplement: Supplementary file 6 [file Data_Sheet_4.ZIP › JPN_CHN (2).tif]

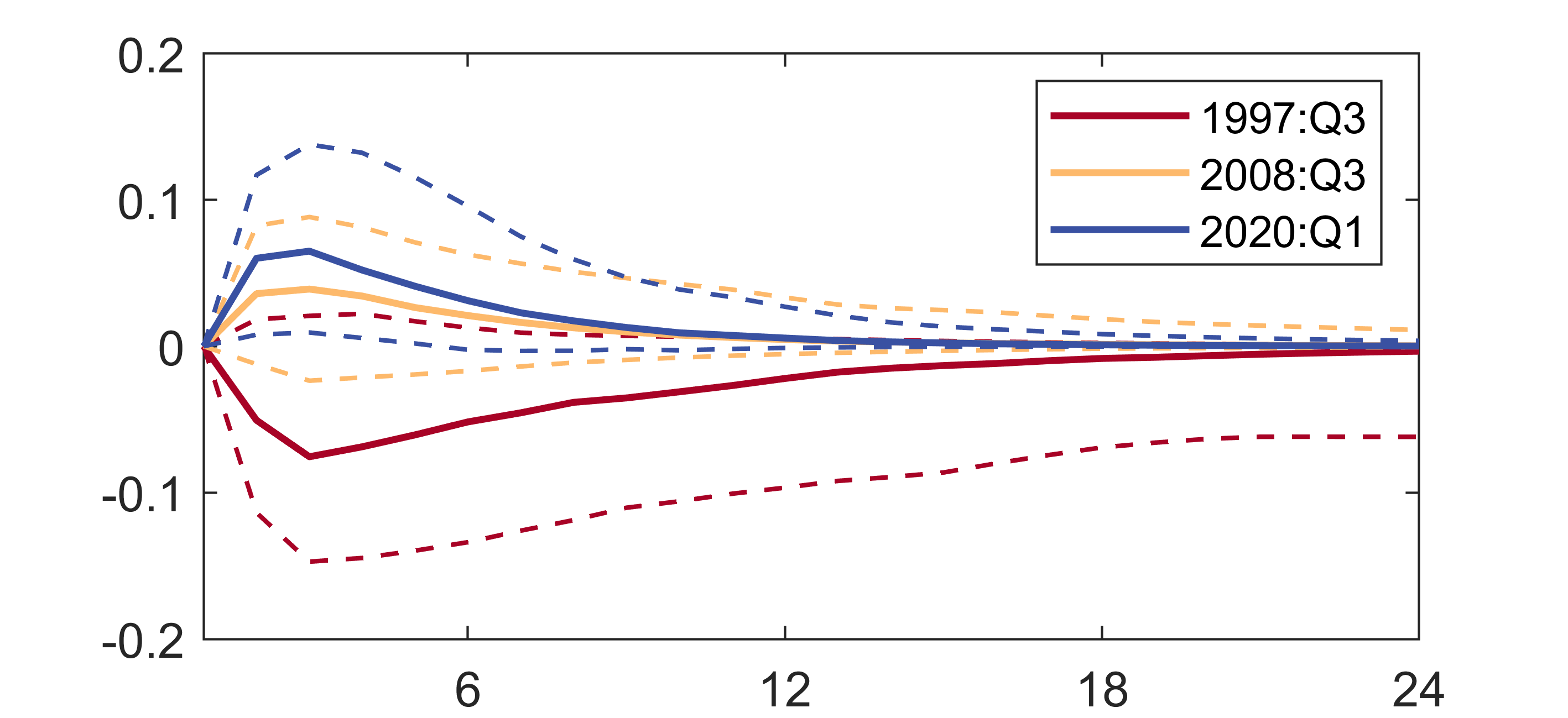

Supplement: Supplementary file 6 [file Data_Sheet_4.ZIP › JPN_CHN (3).tif]

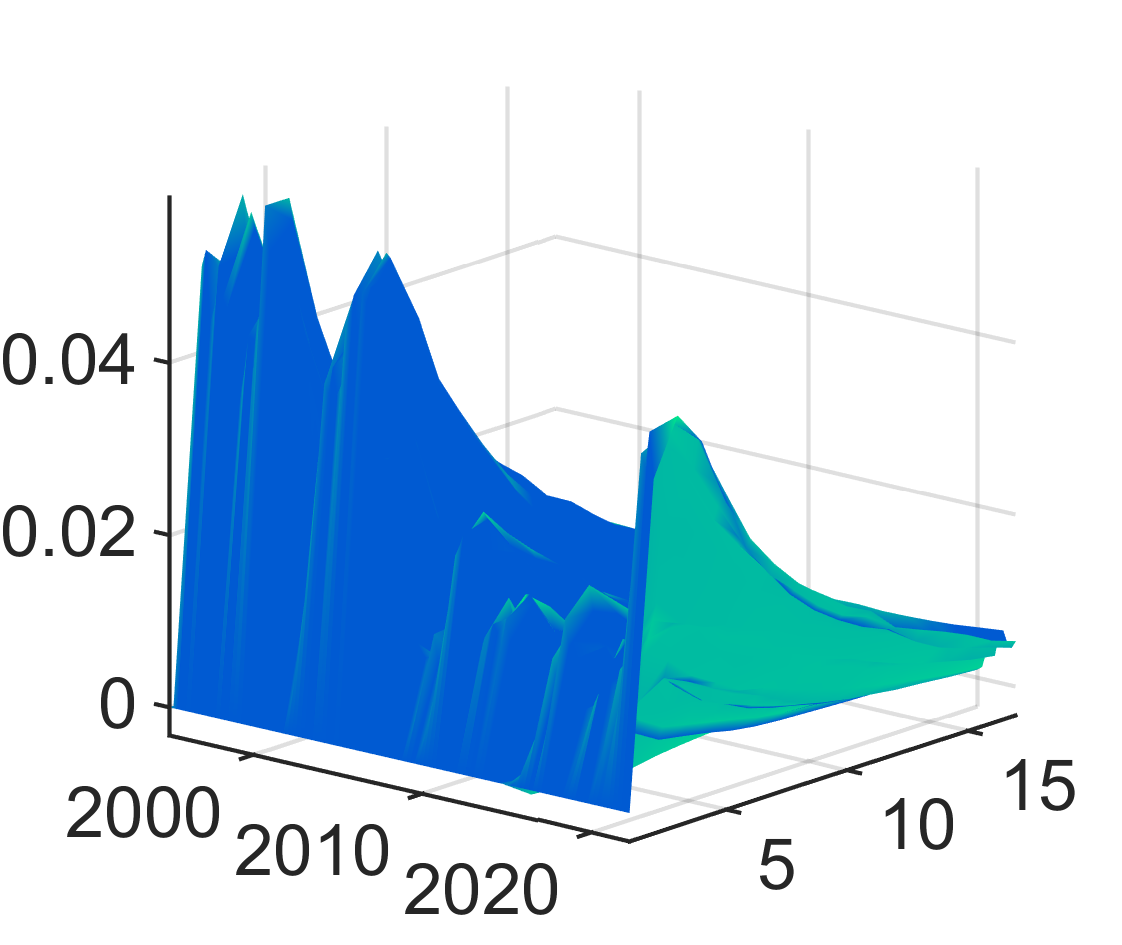

Supplement: Supplementary file 6 [file Data_Sheet_4.ZIP › JPN_HK (1).tif]

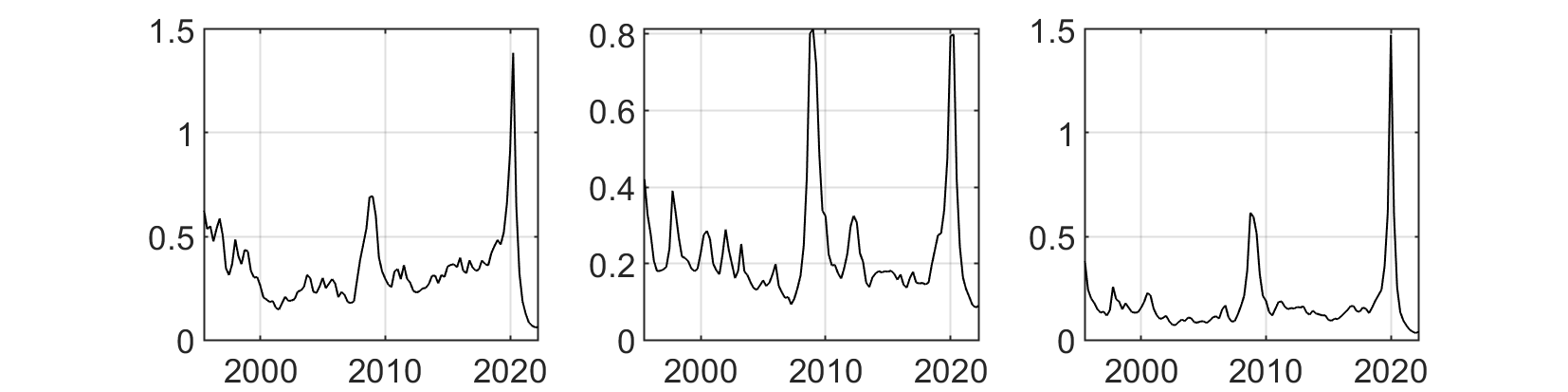

Supplement: Supplementary file 6 [file Data_Sheet_4.ZIP › JPN_HK (2).tif]

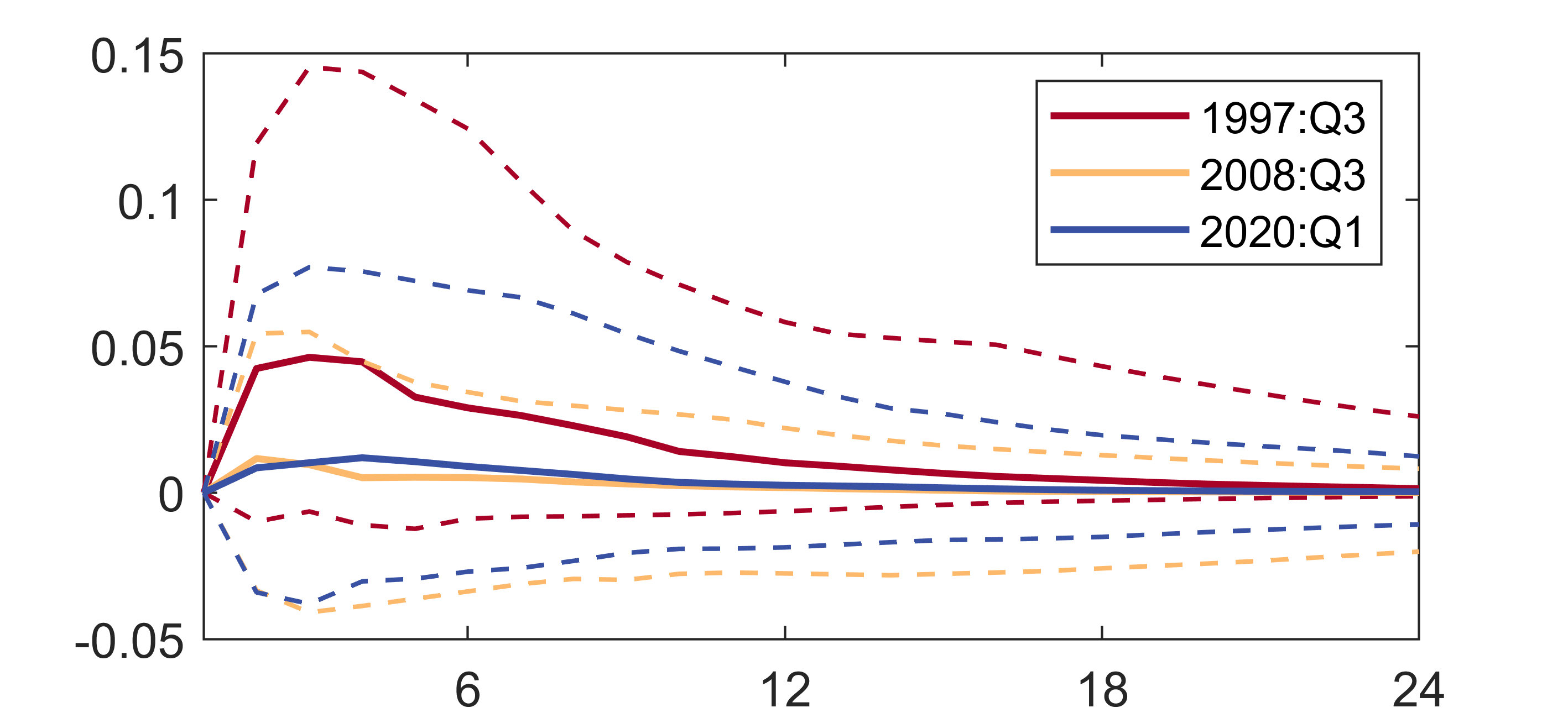

Supplement: Supplementary file 6 [file Data_Sheet_4.ZIP › JPN_HK (3).tif]

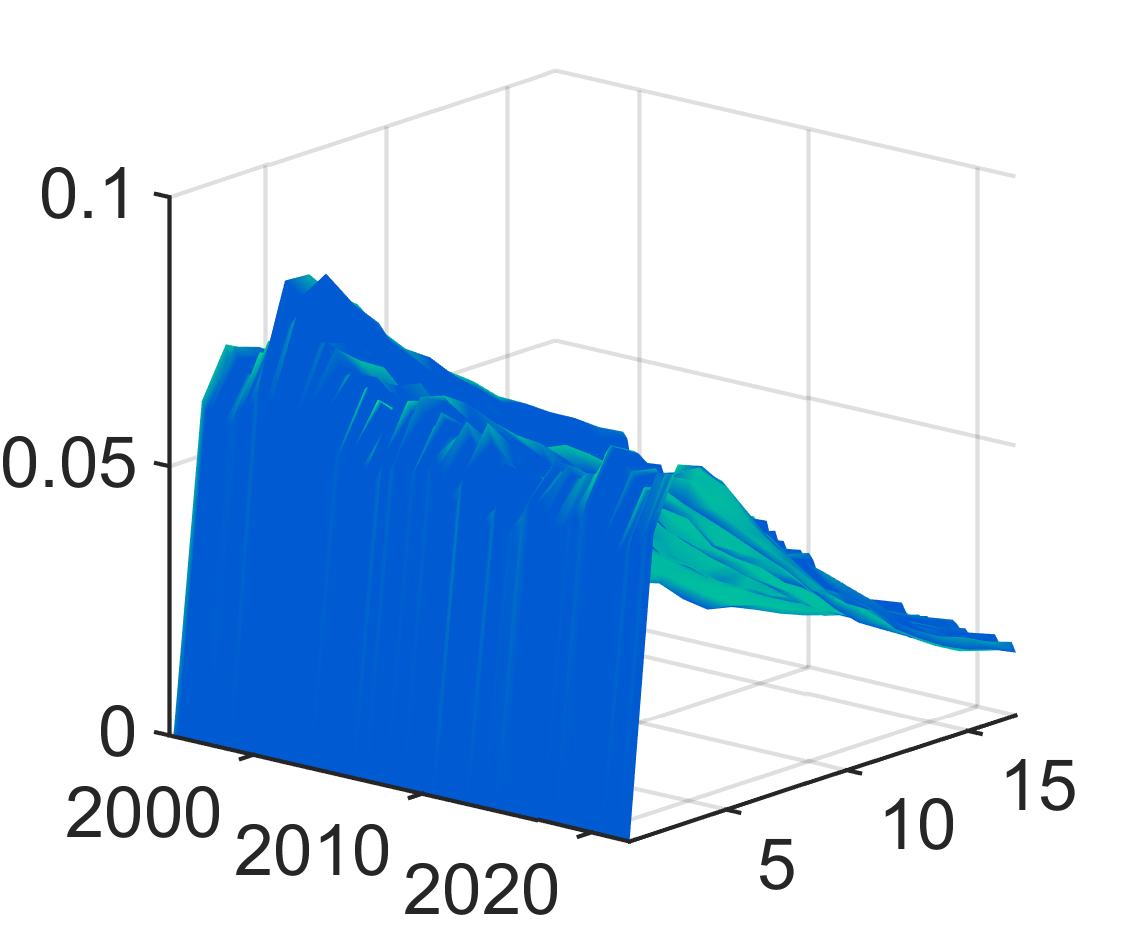

Supplement: Supplementary file 6 [file Data_Sheet_4.ZIP › JPN_KR (1).tif]

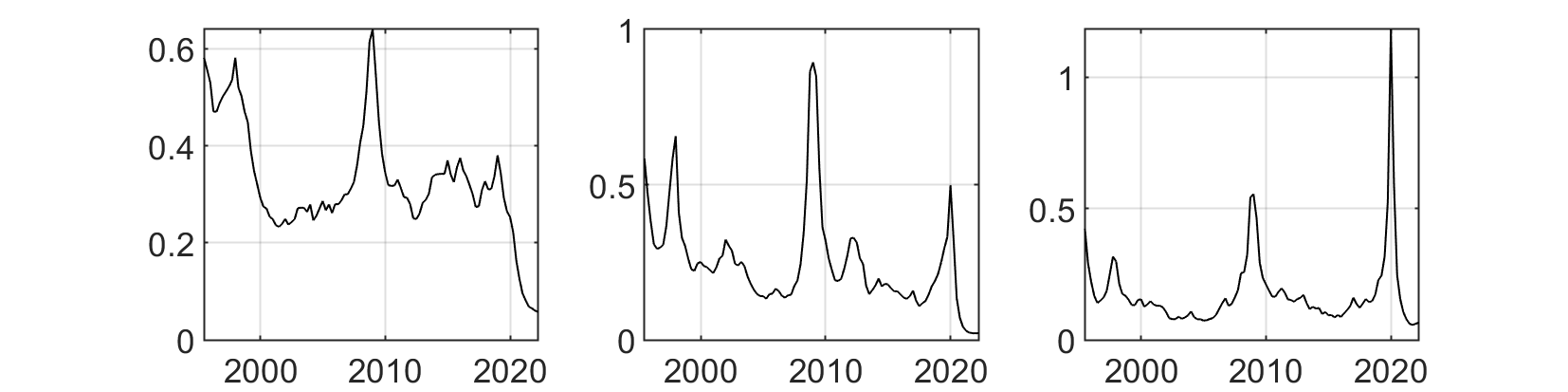

Supplement: Supplementary file 6 [file Data_Sheet_4.ZIP › JPN_KR (2).tif]

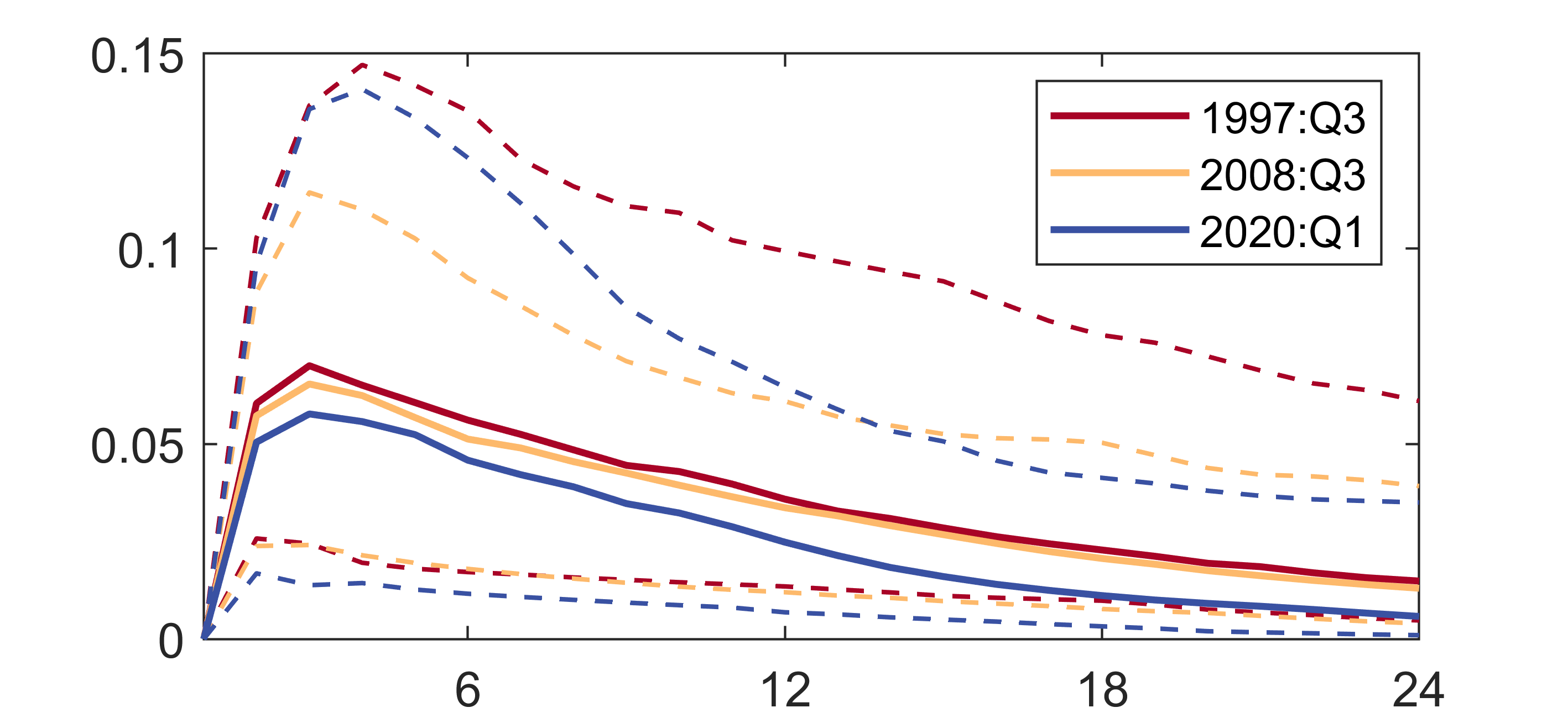

Supplement: Supplementary file 6 [file Data_Sheet_4.ZIP › JPN_KR (3).tif]

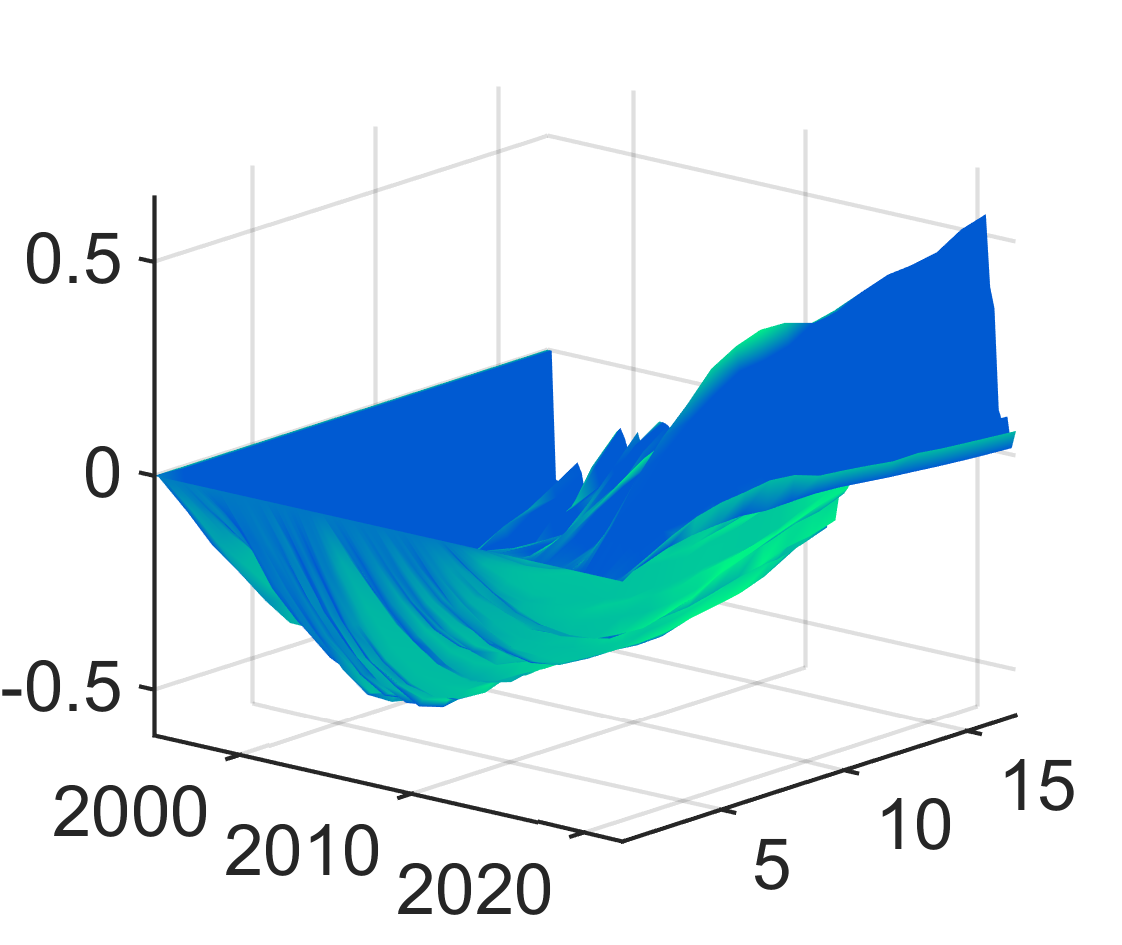

Supplement: Supplementary file 6 [file Data_Sheet_4.ZIP › KR_CHN (1).tif]

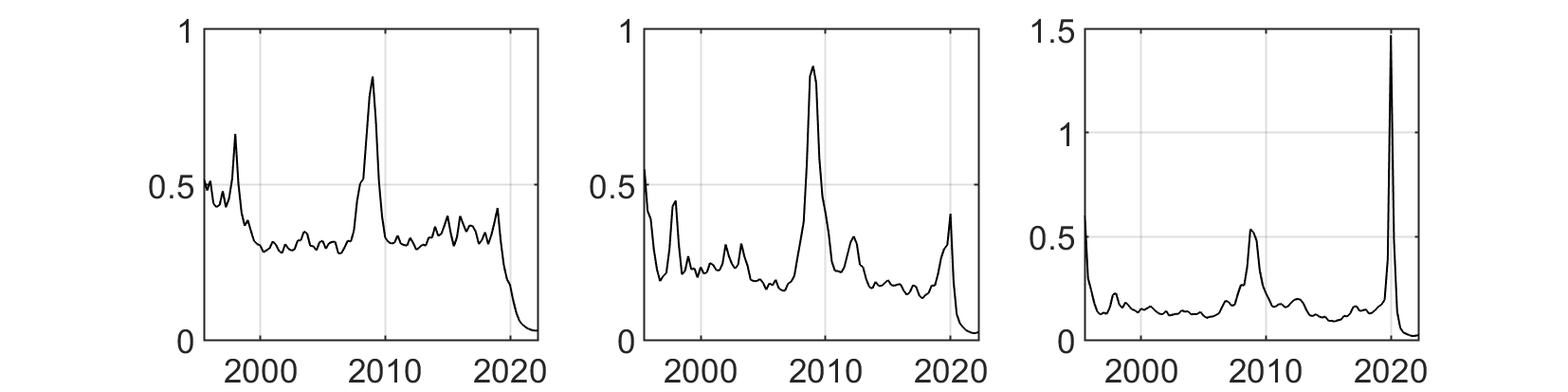

Supplement: Supplementary file 6 [file Data_Sheet_4.ZIP › KR_CHN (2).tif]

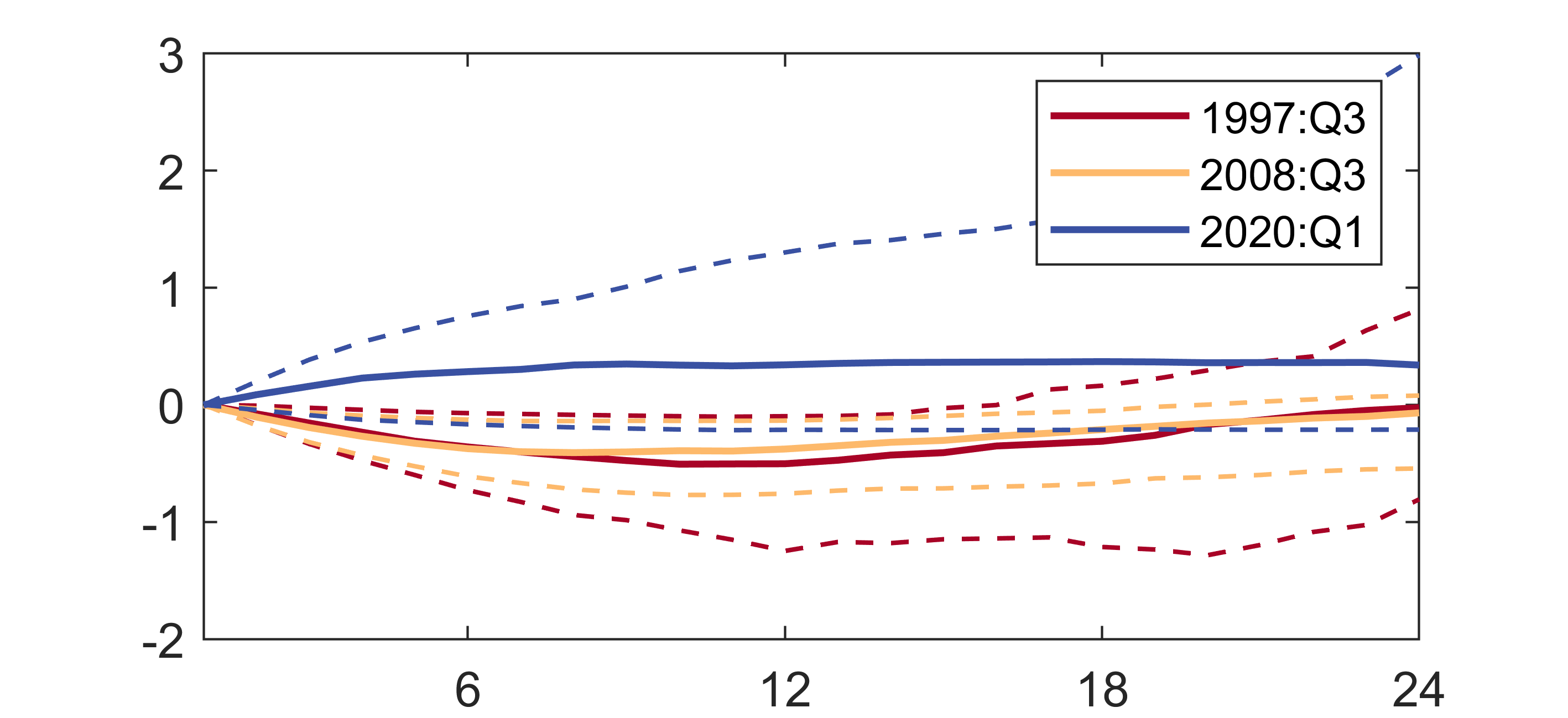

Supplement: Supplementary file 6 [file Data_Sheet_4.ZIP › KR_CHN (3).tif]

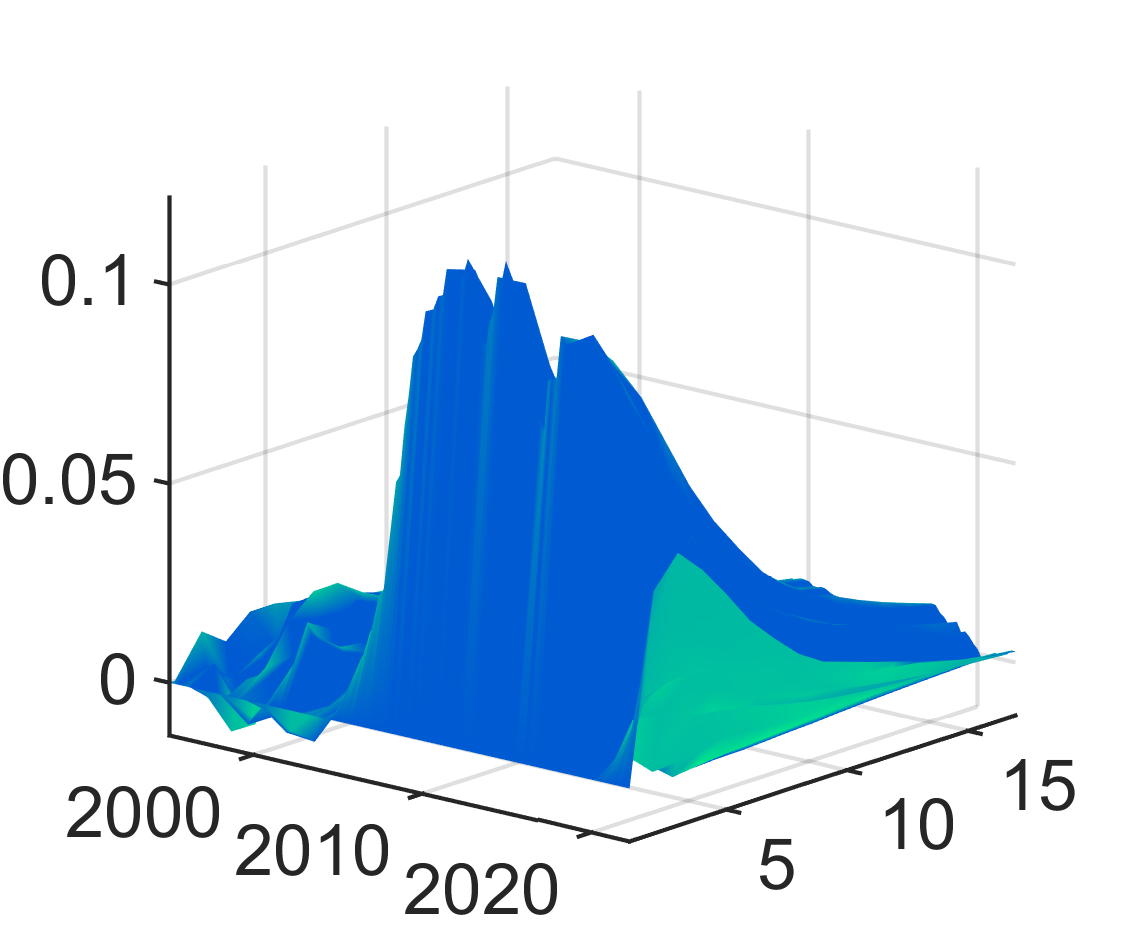

Supplement: Supplementary file 6 [file Data_Sheet_4.ZIP › KR_HK (1).tif]

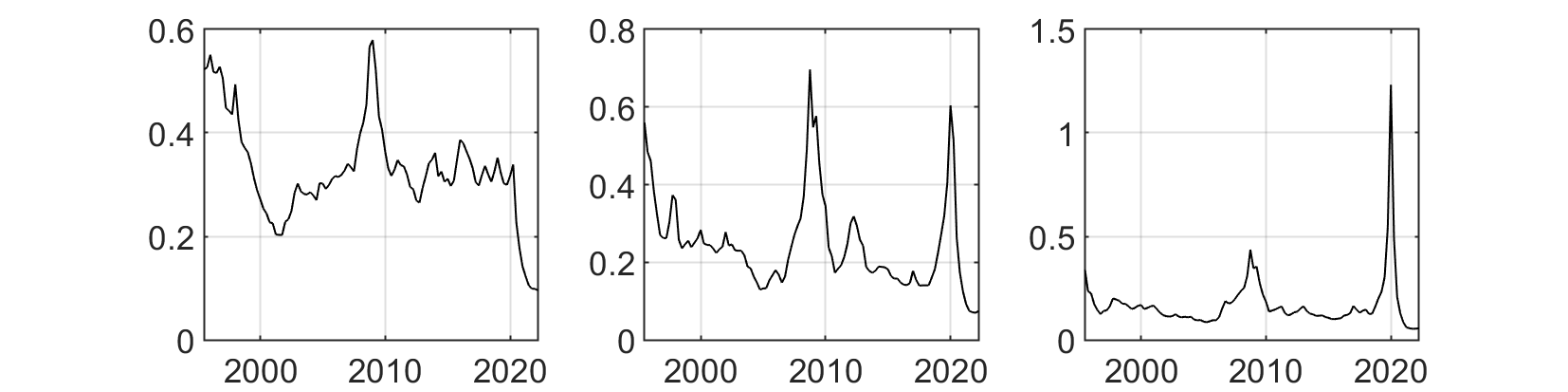

Supplement: Supplementary file 6 [file Data_Sheet_4.ZIP › KR_HK (2).tif]

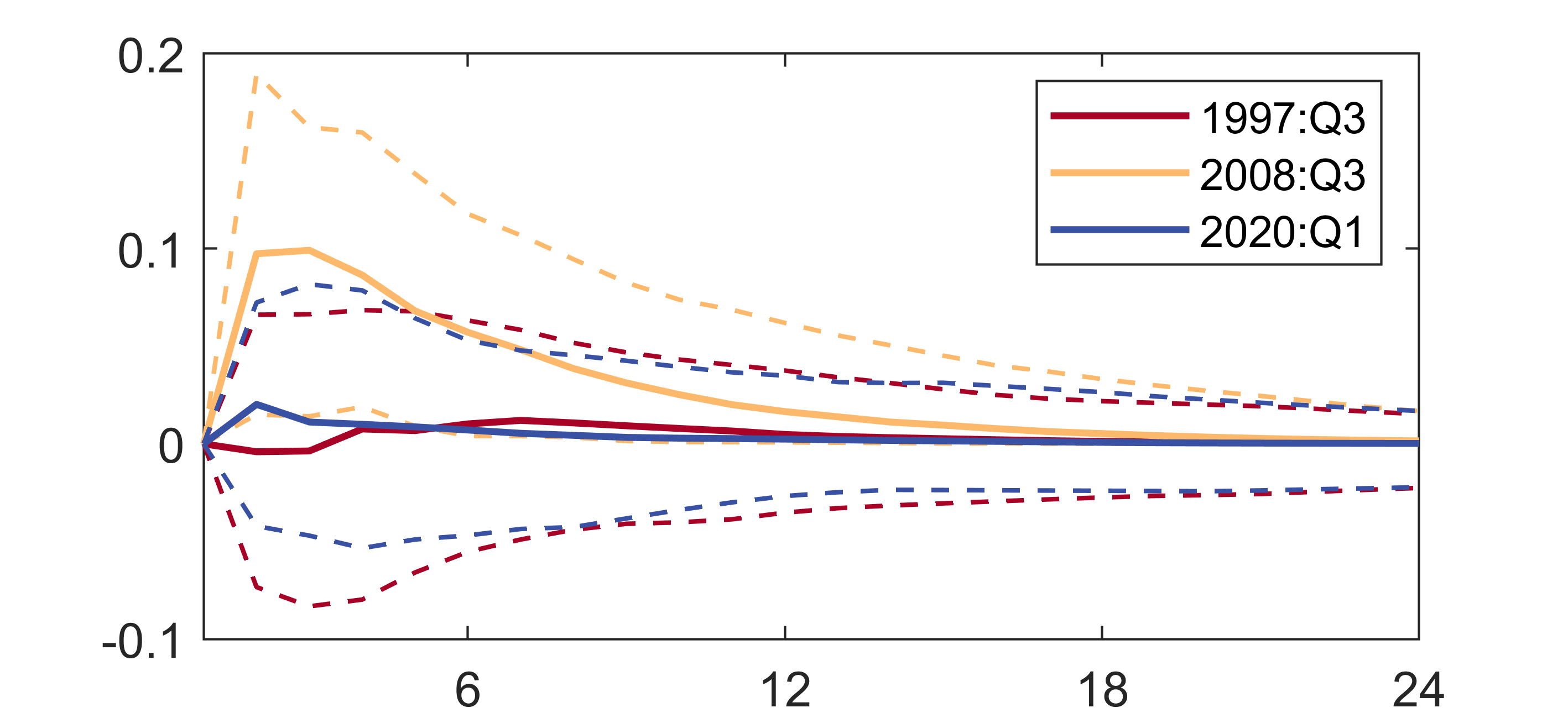

Supplement: Supplementary file 6 [file Data_Sheet_4.ZIP › KR_HK (3).tif]

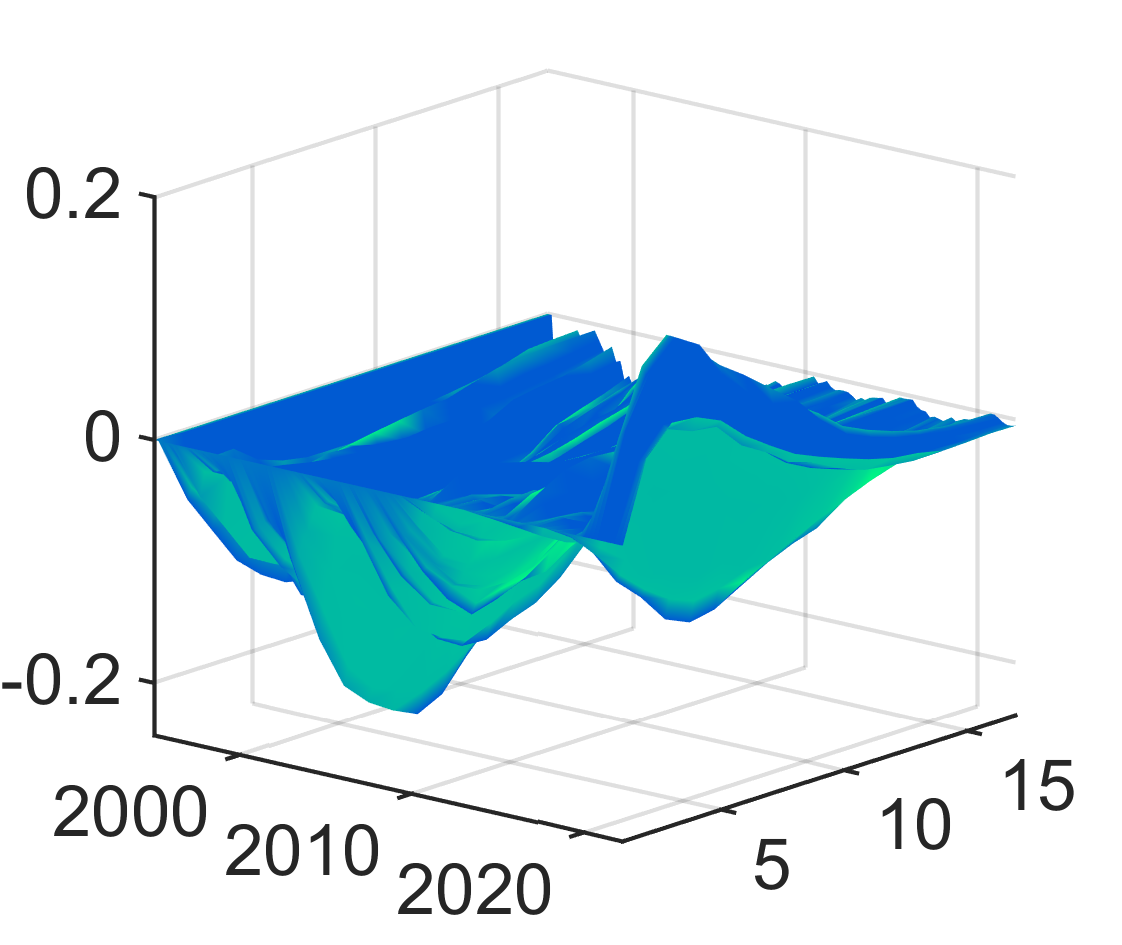

Supplement: Supplementary file 6 [file Data_Sheet_4.ZIP › KR_JPN (1).tif]

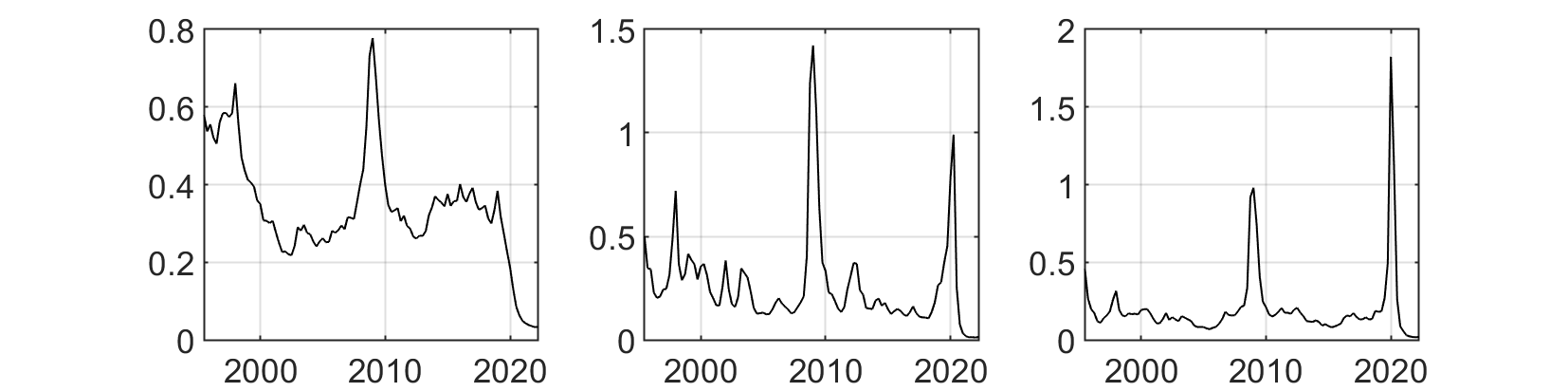

Supplement: Supplementary file 6 [file Data_Sheet_4.ZIP › KR_JPN (2).tif]

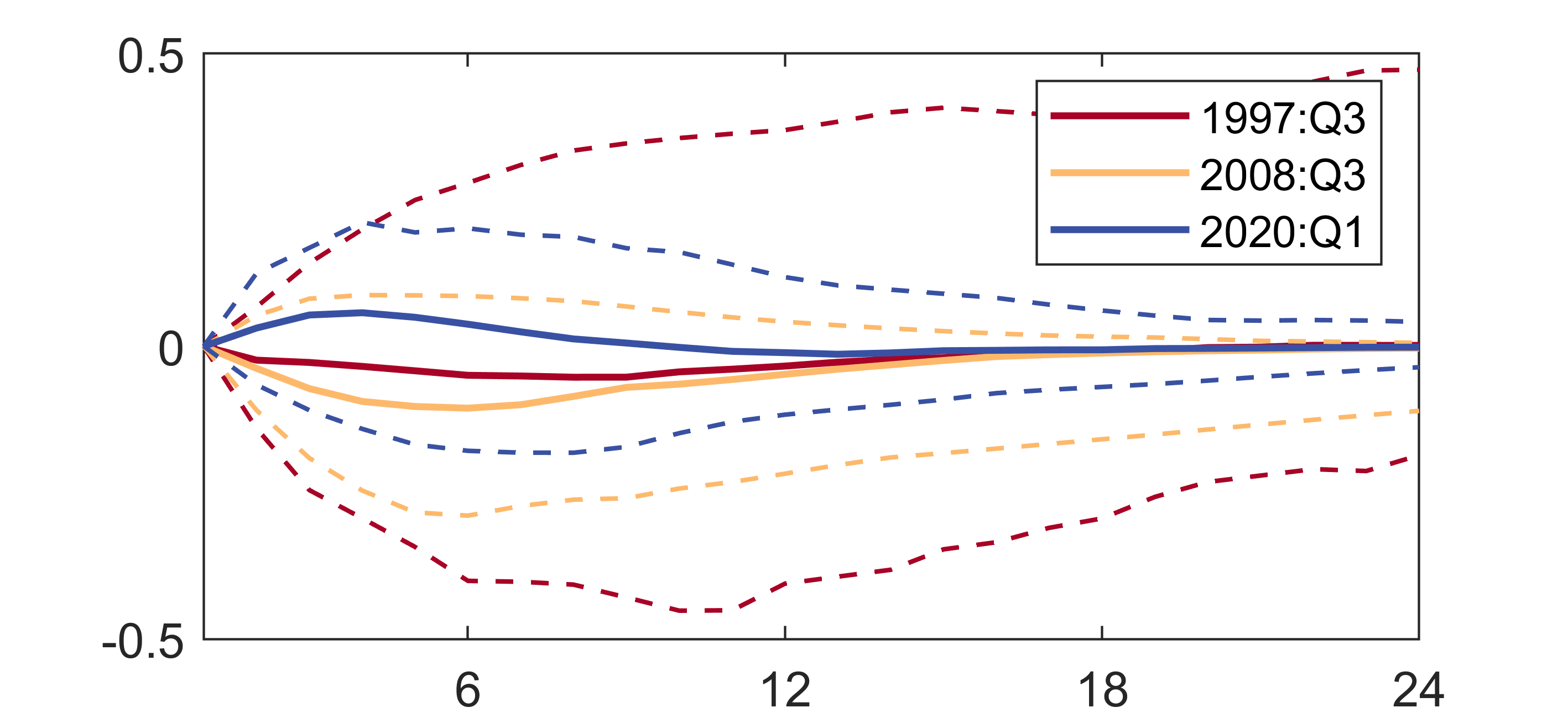

Supplement: Supplementary file 6 [file Data_Sheet_4.ZIP › KR_JPN (3).tif]
